# Detection of regional disparity in cerebrovascular reactivity using a custom whole brain functional near-infrared spectroscopy based mapping system: A prospective observational study

**DOI:** 10.1371/journal.pdig.0001349

**Published:** 2026-04-15

**Authors:** Amanjyot Singh Sainbhi, Nuray Vakitbilir, Tobias Bergmann, Kevin Y. Stein, Rakibul Hasan, Noah Silvaggio, Isuru Herath, Mansoor Hayat, Jaewoong Moon, Frederick A. Zeiler

**Affiliations:** 1 Department of Biomedical Engineering, Price Faculty of Engineering, University of Manitoba, Winnipeg, Manitoba, Canada; 2 Undergraduate Medicine, Rady Faculty of Health Sciences, University of Manitoba, Winnipeg, Manitoba, Canada; 3 Department of Human Anatomy and Cell Science, Rady Faculty of Health Sciences, University of Manitoba, Winnipeg, Manitoba, Canada; 4 Section of Neurosurgery, Department of Surgery, Rady Faculty of Health Sciences, University of Manitoba, Winnipeg, Manitoba, Canada; 5 Pan Am Clinic Foundation, Winnipeg, Manitoba, Canada; 6 Department of Clinical Neurosciences, Karolinska Institutet, Stockholm, Sweden; 7 Division of Anaesthesia, Department of Medicine, Addenbrooke’s Hospital, University of Cambridge, Cambridge, United Kingdom; Beth Israel Deaconess Medical Center, UNITED STATES OF AMERICA

## Abstract

There is limited literature on the ability of high-frequency cerebral functional near-infrared spectroscopy (fNIRS) systems to characterize cerebral autoregulation/cerebrovascular reactivity (CA/CVR) regional disparity than other low-frequency commercial systems. To overcome temporal and spatial limitations of existing commercial NIRS systems, we created a custom-built whole brain CVR mapping system using fNIRS. We preliminarily evaluated regional hemispheric disparity in CA/CVR using various fNIRS derived metrics based on relative hemoglobin concentrations. Healthy volunteer data was recorded for approximately 90 minutes in a block-trial fashion with baseline and perturbation testing. Five types of hemoglobin-based indices were derived using 1 Hz and 250 Hz sampled data. Regional hemispheric disparity between brain lobar regions was evaluated based on median and median absolute deviation metrics. Multi-variate cerebral physiologic relationships between hemispheres were assessed via optimal autoregressive integrative moving average (ARIMA), vector ARIMA impulse response functions, and Granger causality analyses. Data from 50 healthy control volunteers were prospectively analyzed. Our system was able to detect subtle differences between corresponding right and left brain regions through all statistical methods employed, demonstrating the ability of our novel system to detect changes in regional variation of fNIRS and derived CVR measures. These were present and magnified during perturbation testing compared to baseline recordings. However, given the healthy nature of the study population, large differences in CVR measures between brain regions and extreme CVR derangements were not seen. Our custom built fNIRS whole brain CVR mapping system was able to detect subtle regional differences in CVR measures across various time-domain analytic techniques. These findings are in alignment with prior literature, supporting the notion that research-grade fNIRS systems may be adequate for regional disparity analysis of CVR in humans. Future work in diseased/injured human cohorts is required to further quantify the sensitivity of our custom-built system to detect regional variations and disturbances in CVR.

## Author summary

Cerebrovascular reactivity (CVR) metrics quantify the effectiveness of brain’s blood vessels response to physiological stimuli to regulate cerebral blood flow and provide indirect measurements of cerebral autoregulation. Near-infrared spectroscopy has been successful in deriving non-invasive CVR metrics based on recorded hemoglobin concentrations by correlation with arterial blood pressure. Research-grade high-frequency (≥100 Hz) functional NIRS (fNIRS) can successfully provide full waveform data of the measured hemoglobin concentrations along with deriving non-invasive hemoglobin-based CVR indices. Our custom fNIRS system (250 Hz) was able to discern subtle regional hemispheric disparities between brain lobes in healthy population that went through baseline and multiple perturbation testing. This prospective study showed that CVR metrics derived from high-frequency fNIRS system shows huge potential to provide early detection and future intervention than CVR metrics derived from low-frequency commercial NIRS systems (<100 Hz; often ~1 Hz). Although future work is needed to fully evaluate our findings using high-frequency multi-center datasets of healthy and cranial trauma populations.

## 1. Introduction

Cerebral autoregulation (CA) is a physiologic process which ensures optimal brain function over a wide range of systemic arterial blood pressures (ABP) by maintaining a relatively constant cerebral blood flow (CBF) [1,2]. Cerebrovascular reactivity (CVR) is a mechanism behind the maintenance of constant CBF achieved by constriction and dilation of cerebral blood vessels [1,2]. It should be noted as CVR is a broader term for CA used to describe the physiologic process, these terms are not entirely interchangeable since CVR can occur outside the limits of CA. The upper and lower limits of autoregulation (ULA and LLA, respectively) can get shifted and/or the CBF plateau region can get narrowed [1–3] due to various neuropathological states with documented impairments in CA, such as traumatic brain injury (TBI) [4–10]. The literature suggests that a significant driver of poor long-term outcomes in various neurological conditions results from the exposure to impaired CA (i.e., hypoperfusion and hyperperfusion) [6,7,9,11–13]. To help prevent prolonged states of pressure passive flow, continuous monitoring of CA at the patient bedside has potential at early detection and future intervention.

CVR metrics are surrogate measures of CA and help to obtain indirect measurements of CA at bedside [4,14,15]. These metrics assess the association between slow-wave (i.e., 0.05–0.005 Hz) [16,17] vasogenic fluctuations in ABP and a surrogate for CBF or cerebral blood volume (CBv). The most established method for continuous time domain CVR assessment is based on intracranial pressure (ICP) [4,18–20] but the downside is that invasive ICP is monitored typically at a single region to derive a global CVR metric. For non-invasive monitoring of cerebral physiology, near-infrared spectroscopy (NIRS) systems have been leveraged to obtain relative oxygenated and deoxygenated hemoglobin concentrations in the brain tissue to derive NIRS-based CVR indices, and have been described as substitute for ICP-based indices in literature [21–27]. Similar to ICP-based CVR indices, the NIRS-based CVR indices are derived using Pearson correlation of five minute data updated every 10 seconds between cerebral perfusion pressure (CPP)/ABP and pulsatile CBF/CBv surrogates [23,24,26,28–36]. The advantage of non-invasive NIRS-based signals is that it can be acquired from different populations in a multi-channel capacity, which overcomes the issue of spatial resolution associated with ICP-based signals. Neuroimaging and transcranial Doppler literatures suggests that regional disparity in CBF and CA/CVR exists in different healthy and pathologic states [37].

A surrogate of CBv is the NIRS-based regional cerebral oxygen saturation (rSO_2_) signal which can be leveraged to derive the cerebral oximetry index using CPP (COx) and ABP (COx-a). It is often seen that commercial cerebral NIRS systems have a low sampling rate (<100 Hz; often ~1 Hz) compared to research-grade cerebral function NIRS (fNIRS) systems with high sampling rate (≥100 Hz) [38]. A previously conducted scoping review by our group found varying results when discerning regional hemispheric disparity in both health and disease in the current literature, suggesting that many commercially available systems lack the ability to discern hemispheric differences in CA/CVR, particularly in healthy volunteer cohorts [37]. Subsequent retrospective work using a commercial NIRS system, with 1 Hz sampling rate, failed to detect regional hemispheric disparity using continuously derived COx/COx-a in a TBI, operative, and healthy control population cohorts. These results further supports the narrative that commercial NIRS systems may not suffice for detailed regional CVR assessments [39]. In parallel, a prospective observational study was conducted by our group using signals from a commercial and research grade system NIRS systems (1 Hz low-frequency vs 250 Hz high-frequency), finding raw signals from both devices to be grossly different, although they contained similar information at a fundamental level [38]. This raised the question regarding the potential utility of high-frequency research grade fNIRS systems for regional disparity assessments of CA/CVR. At present, the literature surrounding the ability of research-grade cerebral fNIRS systems to characterize CA/CVR regional disparity is limited. It remains unknown if these high-frequency fNIRS systems are able to better discern regional or hemispheric differences in CA/CVR compared to low-frequency commercial NIRS systems. The goal of this prospective observational study was to build and test a custom whole brain fNIRS CVR mapping system and explore its ability to detect regional/hemispheric disparity in CA/CVR during baseline and various perturbation testing.

## 2. Materials and methods

### 2.1. Ethics statement

Data were collected following full approval by the University of Manitoba Health Research Ethics Board (B2022:051) and the Shared Health/Health Sciences Centre Research Impact Committee (SH2022-210). Additionally, the study is registered on ClinicalTrials.gov (ID: NCT05433129). Written informed consent was obtained during the original data collection from the healthy volunteer (HVA) population for the purpose of this analysis.

### 2.2. Study design and human populations

This study was conducted at the University of Manitoba Multi-omic Analytics and Integrative Neuroinformatics in the HUman Brain (MAIN-HUB) Lab as a registered prospective observational study (ClinicalTrials.gov ID: NCT05433129) [38,40,41], with data analysis conducted similar to previous works from our group [38,39,42]. The detailed study protocol can be seen in our previously published manuscript [40]. In short, all the HVAs were 18 years of age or older, with no history of cardiovascular or neurological conditions. In a block-trial fashion, the prospective observational study entailed multi-channel high-frequency cerebral fNIRS recording during baseline and various perturbation testing. Perturbations included assessments of vascular chemo-reactivity through breathing challenges (with end-tidal carbon dioxide [EtCO_2_] monitoring), orthostatic position changes, and neurovascular coupling assessments through formal cognitive testing using the Clinical Toolkit test battery in the Automated Neuropsychological Assessment Metrics (ANAM) test system software (Version 4.5.0.6, Vista Life Sciences, Parker, CO, USA).

The objective of these perturbations was to disturb the CA in healthy volunteers. These perturbations paradigms were selected to probe the complementary and physiologically distinct components of cerebrovascular regulation. The breathing challenges were included to provide controlled hypercapnic and hypocapnic reactivity through slow and fast breathing exercises, respectively. The bidirectional modulation of vascular chemo-reactivity, with concurrent EtCO_2_ monitoring, enabled the assessment on how these states influence regional hemispheric disparity of CVR. The orthostatic position changes of lying-to-sit and sit-to-stand induced transient alterations in systemic blood pressure to enable the assessment on how it influences regional hemispheric disparity of CVR. The cognitive testing using various tests (performance, manikin, pursuit tracking, switching, and Stroop tests) provides neurovascular coupling for the assessment of their influence on the regional hemispheric disparity of CVR. With continuously recording of various fNIRS-based CVR indices at each brain lobe and hemisphere, they can be used as a “gold standard” in healthy volunteers for future comparisons to cranial trauma population data recorded using similar higher-frequency fNIRS systems. All continuous recordings occurred over approximately 90 minutes for each subject.

### 2.3. Data collection

**Fig 1:**
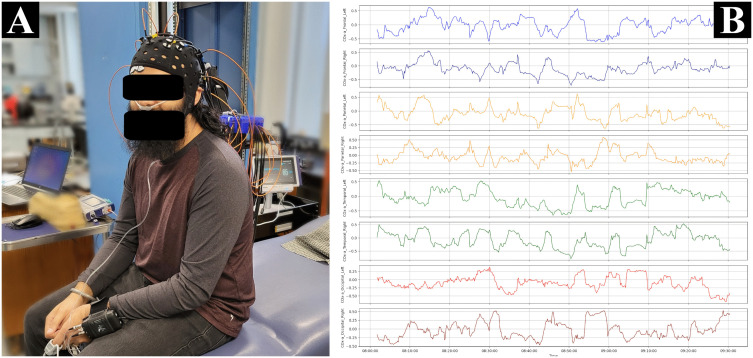
Overview of custom fNIRS system with multi-channel COx-a plot. (A) Displays all sensors for the custom multi-channel fNIRS system on human. (B) Full recording example of multi-channel COx-a signals. COx-a, cerebral oximetry index with arterial blood pressure; fNIRS, functional near-infrared spectroscopy.

**Fig 2:**
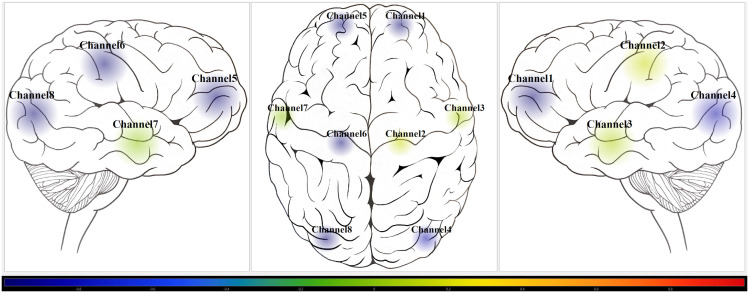
Screenshot of COx-a heat map from custom fNIRS system. Example screenshot of heat map visually displaying the COx-a autoregulation index of a multi-channel recording with colour bar representing the CA/CVR index scale from Blue (-1; intact CVR) to Red (+1; impaired CA/CVR). CA, cerebral autoregulation; COx-a, cerebral oximetry index with arterial blood pressure; CVR, cerebrovascular reactivity; fNIRS, functional near-infrared spectroscopy.

Utilizing a custom multi-channel fNIRS system (OxyMon Mk III; Artinis Medical Systems, Elst, Netherlands), four types of hemoglobin signals were recorded at 250 Hz and the fNIRS cap was used to hold the optodes for short (transmitter and receiver optode distance of 10mm) and normal channels (transmitter and receiver optode distance of 30mm) on a human head [41]. To ensure optimal fNIRS cap fit and increase maximum optode-to-scalp contact at all channels. each volunteer’s head circumference was measured to select the proper fNIRS cap size suggested by the manufacturer (small for 53 cm to 55 cm, medium for 55 cm to 57 cm, and large for 57 cm to 59 cm). The four types of hemoglobin signals recorded were oxyhemoglobin [HbO], deoxyhemoglobin [HHb], total hemoglobin [tHb], and difference between HbO and HHb [HbDiff]. Eight channels were used to assess each of the four brain lobes (frontal, parietal, temporal, and occipital) on both hemispheres of the brain. Extracranial signal contamination was eliminated by subtracting short channel signal from normal channel and produced the differenced HbO, HHb, tHb, and HbDiff. The rSO_2_ signal was derived for each channel by expressing HHb as a percentage of tHb. Non-invasive ABP was collected using the Finapres Nova finger cuff (Finapres Medical Systems, Enschede, The Netherlands) at 100 Hz. The non-invasive EtCO_2_ and respiratory rate was collected using the Capnostream 35 portable respiratory monitor with disposable nose clip (Medtronic, Minneapolis, MN, USA). The figures, Figs 1 and 2, depicts the custom multi-channel fNIRS system on a human (Fig 1A), graph showing all eight channels of COx-a subject example (Fig 1B), and screenshot of heat map of all eight channels of COx-a subject example (Fig 2).

Utilizing a custom recording Python module, all physiological data were recorded and digitized in high-frequency resolution (up to 250 Hz where available) with analog-to-digital signal converters (DT9804 or DT9826, Data Translation, Marlboro, MA, USA), where needed. The regional disparity was looked at using data in two frequencies (1 Hz and 250 Hz), where all physiological data were either down-sampled to 1 Hz using mean or up-sampled to 250 Hz. The 1 Hz (using mean down-sampled) was chosen because many commercial NIRS systems operate at this sampling rate. It was compared with the native sampling frequency of the employed fNIRS system, 250 Hz, to assess where both sampling rates yielded similar results in discerning the regional hemispheric disparity of CVR in healthy humans.

### 2.4. Physiologic data cleaning and processing

Python 3.13.5 was used to clean and process the recorded high resolution physiologic data afterwards. All major signal artifacts were removed from data streams using erroneous label events identified during the data acquisition. These erroneous events were associated with equipment check and adjustment, malfunction of equipment, and signal quality degradation. Each patient had the ABP, rSO_2,_ HbO, HHb, tHb, and HbDiff raw signals decimated using non-overlapping moving average filters of 10-second duration aimed at isolating the slow-wave vasogenic fluctuations associated with CA [16,17,21]. The five fNIRS-based CVR indices were derived using moving Pearson correlation coefficients calculated using 30 consecutive 10-second mean windows (i.e., five minutes of data), updated every 10 seconds. S1a Appendix contains the example Python code to show how the CVR index was derived from raw fNIRS and ABP signals that were either mean down-sampled to 1 Hz or up-sampled to 250 Hz. These derived CVR indices were oxyhemoglobin index (HbOx – correlation between HbO and ABP), deoxyhemoglobin index (HHbx – correlation between HHb and ABP), total hemoglobin index (tHbx – correlation between tHb and ABP), hemoglobin difference index (HbDiffx – correlation between HbDiff and ABP), and cerebral oximetry index with ABP (COx-a – correlation between rSO_2_ and ABP). The range of these CVR indices is from -1 to +1, where higher values (closer to +1) are indictive of impaired CVR, and negative values are associated with intact CVR. Of note, recent healthy control data suggests that in awake volunteers NIRS based CVR may have values in the low positive range [21,43].

### 2.5. Statistical data analysis

All statistical analysis was performed using Python with alpha (α) set at 0.05 for statistical significance. General data distributions were summarized using median, interquartile range (IQR), number, and percentage, where appropriate. The overall analysis of hemispheric disparities (comparing left to right hemispheres) encompassed the following:

A) Median and variance differences (using entire recording periods)
B) Absolute regional hemispheric differences between left and right (using data at 1 Hz and 250 Hz)
C) Optimal time-series autocorrelative structure differences for signals and derived CVR indices (1^st^ order differenced data at 1 Hz and 250 Hz)
D) Time-series impulse response function differences for the ABP on fNIRS signals relationships (1^st^ order differenced data at 1 Hz and 250 Hz)
E) Difference in Granger causality relationships between parent signals of CVR indices (1^st^ order differenced data at 1 Hz and 250 Hz)

**Fig 3:**
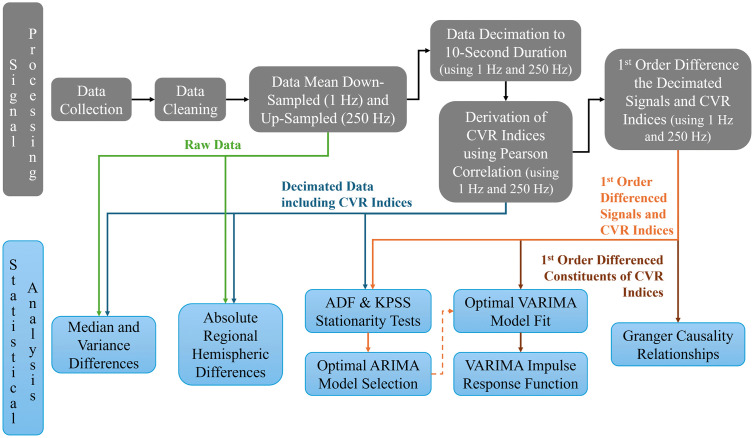
Graphical depiction of signal processing and statistical analysis dataflow. The figure depicts the signal processing and statistical analyses data flow to help in the visualization of the comprehensive regional hemispheric disparity analysis. ADF, Augmented Dickey-Fuller; ARIMA, autoregressive integrative moving average; CVR, cerebrovascular reactivity; KPSS, Kwiatkowski-Phillips-Schmidt-Shin; VARIMA, vector autoregressive integrative moving average.

In the sub-sections to follow, the individual methods will be briefly covered. Fig 3 is a graphical depiction of the data flow of raw signals and decimated signals, including CVR indices, for the employed statistical analyses.

#### 2.5.1. General statistical evaluation – Median hemispheric values.

The median, IQR, and median absolute deviation (MAD) were calculated for each fNIRS signal (rSO_2_, HbO, HHb, tHb, HbDiff, COx-a, HbOx, HHbx, tHbx, and HbDiffx) for both the left and right sides of each brain lobe over the entire recording periods of each subject along with single region measurements of ABP, EtCO_2_, and respiratory rate signals. Similarly, percent time over various literature defined threshold values were calculated for each brain lobe as follows: percent time of bilateral lobe fNIRS signals above 30, 40, 50, and 60, and percent time of bilateral lobe CVR indices above 0, + 0.2, and +0.3. Finally, comparison between left and right hemispheres for each brain lobe were conducted on these recording-based summary metrics using Wilcoxon Rank Sum test (Mann-Whitney-U testing).

#### 2.5.2. Absolute regional hemispheric difference – 1 Hz and 250 Hz sampled data.

The regional hemispheric disparity was determined in individual patients using the left and right hemispheric data of fNIRS signals and derived indices (rSO_2_, HbO, HHb, tHb, HbDiff, COx-a, HbOx, HHbx, tHbx, and HbDiffx) per brain lobe in both frequencies. The absolute value of the hemispheric difference of a signal was calculated for each patient, using the signal’s left and right brain lobe data, to determine the absolute regional hemispheric difference (ARHD). We were able to derive MAD of the ARHDs for the entire population and subgroup analyses (using common parameters and perturbation types) by finding the median of the absolute deviation of each ARHD value to the median of ARHD.

#### 2.5.3. Optimal time-series structures of fNIRS signals and CVR indices – 1 Hz and 250 Hz sampled data.

General methods for optimal time-series autoregressive integrative moving average (ARIMA) structures can be found described in S1b and S1c Appendix, and our previous work on the subject [38,39,42,44]. In brief, data stationarity and optimal autoregressive order (p-order) and moving average order (q-order) were determined for measured and derived signal sources in both frequencies using standard Box-Jenkin’s methodologies while the integrative order (d-order) was essentially held at 1 as the data was manually 1^st^ order differenced although d-order of 0 was supplied to the models [45–47]. Stationarity analysis was performed for each physiologic signal at an individual level using Augmented Dickey-Fuller (ADF; checks if a series is trend stationary) and Kwiatkowski-Phillips-Schmidt-Shin (KPSS; checks if a series is stationary around linear trend) tests [47]. Optimal ARIMA model orders were determined using lowest Akaike Information Criterion (AIC; a score used to compare ARIMA models) values for the derived ARIMA models. Comparison for difference between hemispheres in this time-series structure was conducted qualitatively, with median optimal structures tabulated for each signal across the population in both frequencies.

#### 2.5.4. Time-series impulse response function (IRF) for ABP and fNIRS signals – 1^st^ order differenced data.

A general description of vector ARIMA (VARIMA) models can be found in S1d Appendix and our previous work in the area [38,39,42,44]. In brief, VARIMA models were derived to evaluate for hemispheric differences in the multi-variate relationship between ABP and rSO_2_/HbO/HHb/tHb/HbDiff (i.e., the constituents of fNIRS-based CVR indices). The VARIMA p-order was calculated by taking the product of the optimal ARIMA p-orders of the two signals being evaluated while the VARIMA q-order was calculated by adding the optimal ARIMA q-orders of the two signals being evaluated, as suggested from past literature to ensure adequate model structure for such multivariate time-series [38,46]. These calculations were employed to determine optimal VARIMA p-order and q-order based on the univariate optimal ARIMA p-orders and q-orders as it guarantees that the autoregressive structure of each univariate time-series is embedded in the multivariate system along with accounting for higher moving average lags. IRF coefficients of the VARIMA model were generated using optimal VARIMA model for the normalized 1^st^ order differenced data, in both frequencies. The responsiveness of various models was checked to see if it was greater than an absolute value of 0.001 threshold. This threshold was chosen to look at a change in the normalized response of at least 0.1% in the majority of 5 lags after lag of 10 (lags 11–15). The number of patients showing a greater response than 0.1% were counted for each directionality relationship between two signals and it was used to compare the cerebral physiologic relationships between hemispheres of each brain lobe.

#### 2.5.5. Differences in granger causality relationships between parent signals of CVR indices – 1^st^ order differenced data.

To evaluate the interdependence between ABP and rSO_2_/HbO/HHb/tHb/HbDiff (i.e., the constituents of fNIRS-based CA/CVR indices), and any hemispheric disparities, we leveraged Granger causality testing. Granger causality testing was used to assess the ability of one signal to predict another signal beyond the ability of the signal to predict itself. Using the 1^st^ order differenced data in both frequencies, the responses of Granger Causality tests, both F-test statistic value and p-values [48], were recorded. The identification of reciprocal influences between two signals were assessed from these responses and the cerebral physiologic relationships between hemispheres of each brain lobe were compared.

#### 2.5.6. Subgroup analysis – Common parameters and perturbations.

All the above aspects of analysis for hemispheric disparity in fNIRS signals and derived CVR indices using the full recording (including all baseline/perturbations) were also conducted in various subgroups. Using both full length recordings subgrouped by common parameters and perturbation subgrouped recordings, the subdivided data was separately re-evaluated to see if any analysis produced any outlying groups. The common parameters consisted of age (<40 vs 40 – 60; moderate vs old age), biological sex (male vs female), and hand dominance (left hand vs right hand dominance). For the perturbation subgroup analysis, all recordings were subgrouped based on baseline, neurovascular coupling, orthostatic challenge, and vascular chemo-reactivity.

## 3. Results

### 3.1. Population demographics

A total of 50 HVAs were included in this study, which had a median recording duration of 89.8 minutes (IQR: 85.8 – 95.5 minutes). The median age of the HVA population was 28 years (IQR: 23.3 – 35 years), median EtCO_2_ was 35.5 mmHg (IQR: 34 – 37 mmHg), and median respiratory rate was 18 breaths per minute (bpm; IQR: 15 – 20 bpm), while most of the subjects were right handed (98%) and the representation of male and female were nearly equal (56% and 44%, respectively). Table 1 shows the summary of demographics of the HVA population including employed ANAM tests.

**Table 1:** Demographic data for HVA population.

Variable	Median (IQR) or Number (%)
Duration of Recording (minutes)	89.8 (85.8–95.5)
Number of Patients	50
Age (years)	28 (23.3–35)
Biological Sex (Male)	28 (56%)
Hand Dominance (Right)	49 (98%)
EtCO_2_ (mmHg)	35.5 (34–37)
Respiratory Rate (bpm)	18 (15–20)
ANAM – Standard Continuous Performance Test	
Median Response Time (msec)	395.5 (365.625–430.5)
Correct (%)	100 (100–100)
ANAM – Manikin Test	
Median Response Time (msec)	1385.5 (971.125–2116.5)
Correct (%)	90.62 (72.66–96.88)
ANAM – Pursuit Tracking Test	
Median Distance	8 (7–8)
Correct (%)	99.5 (98.435–99.92)
ANAM – Switching Test	
Median Response Time (msec)	1771.75 (1402.375–2213.375)
Correct (%)	92.19 (84.38–95.31)
ANAM – Stroop Test	
Interscore	13.6 (7.4325–20.88)
ANAM – Stroop Test (Word)	
Median Response Time (msec)	714.5 (642–840.625)
Correct (%)	96.71 (91.9975–99.615)
ANAM – Stroop Test (Colour)	
Median Response Time (msec)	653.25 (579–818.75)
Correct (%)	98.055 (93.5825–100)
ANAM – Stroop Test (Colour & Word)	
Median Response Time (msec)	837.5 (693.75–1188.75)
Correct (%)	94.29 (90.44–98.19)

The table depicts the summary of demographic data for the HVA population. ANAM, automated neuropsychological assessment metrics; bpm, breaths per minute; EtCO_2_, end-tidal carbon dioxide; HVA, healthy volunteer; IQR, interquartile range; mmHg, millimeters of mercury.

### 3.2. Median entire recording summary metrics – Hemispheric differences

Various population physiologic variables were examined per brain lobe including ABP, EtCO_2_, respiratory rate, bilateral lobe fNIRS signals (rSO_2_, HbO, HHb, tHb, and HbDiff), bilateral lobe fNIRS CVR indices (COx-a, HbOx, HHbx, tHbx, and HbDiffx), bilateral lobe MAD of fNIRS signals and CVR indices, and Mann-Whitney U test between the signals of each bilateral brain lobe. The percent time of each brain lobe’s bilateral fNIRS signals above 30, 40, 50, and 60, and the percent time of each brain lobe’s bilateral CVR indices above 0, + 0.2, and +0.3 were also calculated, along with performing the Mann-Whitney U test between the bilateral percent time of a signal at each brain lobe. Overall, the mean CVR measures across all brain regions was ~ 0, suggesting relatively intact CVR through the duration of testing. In general, across both frequencies, no significant p-values were reported between left and right fNIRS-based CVR indices (COx-a, HbOx, HHbx, tHbx, and HbDiffx) of each brain lobe along with their MAD and the only significant p-values found were between the occipital lobe hemispheres reported using all five CVR indices. This is in keeping with prior analysis using grand averaged data for CVR measures. Table 2 gives the hemispheric difference summary of the CVR indices with Mann-Whitney U test p-values using 1 Hz sampled data and S2a Appendix shows similar summary using 250 Hz sampled data.

**Table 2:** Median and IQR of CVR indices using 1 hz sampled data.

CVR Index	Brain Lobe	Median (IQR)		p-value
		Left Hemisphere	Right Hemisphere	
COx-a (au)	Frontal	0 (−0.22–0.19)	0 (−0.19–0.21)	0.3027
	Parietal	0 (−0.2–0.2)	−0.01 (−0.2–0.19)	0.5695
	Temporal	0 (−0.2–0.19)	0 (−0.18–0.2)	0.9643
	Occipital	0 (−0.14–0.14)	0.01 (−0.18–0.2)	0.3907
HbOx (au)	Frontal	−0.02 (−0.22–0.18)	0.01 (−0.2–0.22)	0.0557
	Parietal	0.01 (−0.2–0.22)	0.02 (−0.19–0.22)	0.6075
	Temporal	0.01 (−0.2–0.19)	0.01 (−0.18–0.21)	0.4545
	Occipital	0.02 (−0.14–0.18)	0 (−0.18–0.2)	0.4713
HHbx (au)	Frontal	0 (−0.19–0.19)	0.01 (−0.18–0.22)	0.9149
	Parietal	0 (−0.21–0.2)	0.01 (−0.17–0.21)	0.201
	Temporal	0 (−0.19–0.19)	0.01 (−0.19–0.21)	0.3574
	Occipital	0.01 (−0.14–0.17)	0.01 (−0.18–0.21)	0.5059
tHbx (au)	Frontal	−0.01 (−0.21–0.19)	0.01 (−0.18–0.22)	0.2806
	Parietal	−0.01 (−0.2–0.2)	0.01 (−0.18–0.23)	0.132
	Temporal	0 (−0.19–0.19)	0.01 (−0.19–0.21)	0.702
	Occipital	0.01 (−0.15–0.18)	0.01 (−0.18–0.21)	0.9423
HbDiffx (au)	Frontal	0 (−0.21–0.18)	0.01 (−0.18–0.23)	0.1627
	Parietal	0 (−0.19–0.2)	−0.01 (−0.21–0.19)	0.627
	Temporal	0 (−0.18–0.18)	0.01 (−0.18–0.2)	0.6666
	Occipital	0 (−0.14–0.16)	0 (−0.18–0.2)	0.8067
MAD of COx-a (au)	Frontal	0.21 (0.18–0.23)	0.21 (0.17–0.23)	0.9753
	Parietal	0.19 (0.16–0.21)	0.2 (0.17–0.22)	0.1467
	Temporal	0.2 (0.16–0.23)	0.2 (0.17–0.22)	0.8227
	Occipital	0.14 (0.13–0.16)	0.2 (0.17–0.22)	**<0.001**
MAD of HbOx (au)	Frontal	0.2 (0.17–0.22)	0.21 (0.18–0.24)	0.0782
	Parietal	0.2 (0.18–0.22)	0.2 (0.18–0.24)	0.9423
	Temporal	0.19 (0.17–0.23)	0.19 (0.17–0.22)	0.4299
	Occipital	0.16 (0.14–0.19)	0.19 (0.17–0.22)	**<0.001**
MAD of HHbx (au)	Frontal	0.2 (0.18–0.24)	0.2 (0.18–0.23)	0.9698
	Parietal	0.19 (0.17–0.23)	0.21 (0.19–0.23)	0.2963
	Temporal	0.2 (0.16–0.22)	0.19 (0.17–0.23)	0.6716
	Occipital	0.15 (0.13–0.17)	0.19 (0.17–0.23)	**<0.001**
MAD of tHbx (au)	Frontal	0.2 (0.18–0.22)	0.2 (0.18–0.23)	0.855
	Parietal	0.2 (0.17–0.23)	0.21 (0.18–0.24)	0.3832
	Temporal	0.2 (0.18–0.23)	0.2 (0.17–0.23)	0.9423
	Occipital	0.16 (0.15–0.21)	0.2 (0.17–0.22)	**<0.001**
MAD of HbDiffx (au)	Frontal	0.2 (0.18–0.23)	0.21 (0.17–0.23)	0.8442
	Parietal	0.18 (0.16–0.21)	0.2 (0.18–0.23)	0.1075
	Temporal	0.18 (0.16–0.22)	0.2 (0.16–0.22)	0.5464
	Occipital	0.14 (0.13–0.17)	0.2 (0.17–0.22)	**<0.001**

The p-values in the table are derived using Mann-Whitney U test between the bilateral signals. COx-a, cerebral oximetry index with arterial blood pressure; CVR, cerebrovascular reactivity index; HbDiffx, hemoglobin difference index; HbOx, oxyhemoglobin index; HHbx, deoxyhemoglobin index; IQR, interquartile range; MAD, median absolute deviation; tHbx, total hemoglobin index.

Across both frequencies of raw and 10-second decimated data, significant p-values were found between left and right of all four brain lobes for rSO_2_ and HbDiff signals, temporal lobes for HbO signal, and occipital lobes for HbO, HHb, and tHb signals. Between MAD of these fNIRS signals, the only significant p-values found were between the occipital lobes except for MAD of HbDiff. S2b and S2c Appendix gives summary of these hemispheric differences of the fNIRS signals with Mann-Whitney U test p-values using 1 Hz and 250 Hz of 10-second decimated data and raw data, respectively.

For all brain lobes of both hemispheres, median percent of time for rSO_2_ above 30% was 100% but it started to decrease to 0% of time for rSO_2_ above 60%. Significant p-values were seen between most brain lobes for rSO_2_ above 40% but for rSO_2_ above 30%, only frontal lobes showed significant p-values. The median percentage of time the fNIRS-based CVR indices were above zero was ~ 50%, above 0.2 was ~ 25%, and above 0.3 was ~ 15%. Most of the time, the significant p-values were seen between the occipital lobes for percentage of time a CVR index was above 0.2 or 0.3. The summary of the percent time results of rSO_2_ and CVR indices with p-values from Mann-Whitney U tests using 10-second decimated data at both frequencies is given in S2d Appendix, while the S2e Appendix shows the percent time results of rSO_2_ using raw data at both frequencies.

### 3.3. Absolute regional hemispheric differences (ARHD) – 1 Hz and 250 Hz sampled data

Overall, for all four brain lobes, the ARHD and MAD was ~ 0.22 between hemispheres for all CVR indices and their MAD of ARHD was ~ 0.14 using 10-second decimated data in 1 Hz and 250 Hz, as seen in Table 3 and S3a Appendix, respectively. This supports the ability of the custom-built system to detect subtle differences in CVR between brain regions. Regarding ARHD and MAD of fNIRS signals, rSO_2_ had the lowest ARHD ~6% between hemispheres of frontal and parietal lobe and it increased to ~9.5% for the temporal and occipital lobes, while their MAD of ARHD was ~ 1.25%. Regarding the ARHD and its MAD of other four hemoglobin signals, it varied since the measured hemoglobin concentrations were relative and not absolute. S3b Appendix has the results of ARHD and its MAD for the fNIRS signals using raw and 10-second decimated data sampled at both 1 Hz and 250 Hz.

**Table 3:** Regional hemispheric disparity analysis on CVR indices using 10-second decimated data at 1 Hz.

Physiologic Variable	Median (IQR)			
	Frontal Lobe	Parietal Lobe	Temporal Lobe	Occipital Lobe
ARHD of COx-a (au)	0.23 (0.11–0.41)	0.22 (0.1–0.44)	0.21 (0.1–0.4)	0.24 (0.11–0.42)
ARHD of HbOx (au)	0.22 (0.1–0.41)	0.21 (0.09–0.43)	0.23 (0.1–0.43)	0.23 (0.11–0.39)
ARHD of HHbx (au)	0.22 (0.09–0.42)	0.23 (0.1–0.44)	0.21 (0.1–0.45)	0.23 (0.1–0.41)
ARHD of tHbx (au)	0.23 (0.1–0.43)	0.22 (0.1–0.43)	0.23 (0.1–0.43)	0.22 (0.1–0.4)
ARHD of HbDiffx (au)	0.23 (0.11–0.4)	0.22 (0.1–0.43)	0.22 (0.1–0.41)	0.23 (0.1–0.4)
MAD of ARHD COx-a (au)	0.14 (0.12–0.17)	0.15 (0.12–0.17)	0.14 (0.12–0.15)	0.14 (0.12–0.16)
MAD of ARHD HbOx (au)	0.14 (0.12–0.17)	0.14 (0.12–0.17)	0.15 (0.12–0.17)	0.14 (0.12–0.16)
MAD of ARHD HHbx (au)	0.15 (0.12–0.18)	0.15 (0.13–0.17)	0.14 (0.12–0.17)	0.14 (0.12–0.16)
MAD of ARHD tHbx (au)	0.14 (0.12–0.16)	0.15 (0.12–0.17)	0.15 (0.13–0.17)	0.14 (0.12–0.16)
MAD of ARHD HbDiffx (au)	0.13 (0.12–0.16)	0.14 (0.12–0.17)	0.14 (0.12–0.16)	0.14 (0.12–0.16)

The table shows the absolute regional hemispheric disparity analysis in on four brain lobes of the five fNIRS-derived CVR indices using 10-second decimated data at 1 Hz sampling frequency. ARHD, absolute regional hemispheric difference; au, arbitrary units; COx-a, cerebral oximetry index with arterial blood pressure; CVR, cerebrovascular reactivity index; fNIRS, function near-infrared spectroscopy; HbDiffx, hemoglobin difference index; HbOx, oxyhemoglobin index; HHbx, deoxyhemoglobin index; IQR, interquartile range; MAD, median absolute deviation; tHbx, total hemoglobin index.

### 3.4. Hemispheric difference in optimal ARIMA structure – 1 Hz and 250 Hz sampled data

Overall, across both frequencies, there was no significant difference in fNIRS signals and their derived CVR indices regarding stationarity between left and right hemispheres for all brain lobes as all these signals demonstrated a similar level of non-stationary behaviour on ADF and KPSS testing, requiring 1^st^ order differencing to mitigate. Table 4 provides general results for ADF and KPSS testing on non-differenced and 1^st^ order differenced CVR data streams using 1 Hz sampled data, while S4a Appendix provides such analysis using 250 Hz sampled data. Similar results for the remaining fNIRS signals are provided in S4b and S4c Appendix. To note, for some patients, NA in these tables/appendices represents that stationarity could not be determined due to inadequate data points.

**Table 4:** ADF and KPSS results showing stationary vs non-stationary vs NA for calculated fNIRS CA indices at 1 Hz.

ADF results for non-differenced data																
Hemisphere	Brain Lobe	HbOx			HHbx			tHbx			HbDiffx			COx-a		
		S	NS	NA	S	NS	NA	S	NS	NA	S	NS	NA	S	NS	NA
Left	Frontal	50	0	0	49	1	0	50	0	0	49	1	0	49	1	0
	Parietal	49	1	0	47	3	0	49	1	0	48	2	0	49	1	0
	Temporal	49	1	0	49	1	0	49	1	0	50	0	0	50	0	0
	Occipital	46	4	0	50	0	0	49	1	0	48	2	0	49	1	0
Right	Frontal	48	2	0	49	1	0	50	0	0	50	0	0	50	0	0
	Parietal	48	2	0	48	2	0	50	0	0	47	3	0	47	3	0
	Temporal	49	1	0	49	1	0	50	0	0	50	0	0	49	1	0
	Occipital	50	0	0	50	0	0	50	0	0	49	1	0	49	1	0
**ADF results for 1** ^**st**^ **order differenced data**																
**Hemisphere**	**Brain Lobe**	**HbOx**			**HHbx**			**tHbx**			**HbDiffx**			**COx-a**		
		**S**	**NS**	**NA**	**S**	**NS**	**NA**	**S**	**NS**	**NA**	**S**	**NS**	**NA**	**S**	**NS**	**NA**
Left	Frontal	50	0	0	50	0	0	50	0	0	50	0	0	50	0	0
	Parietal	50	0	0	50	0	0	50	0	0	50	0	0	50	0	0
	Temporal	50	0	0	50	0	0	50	0	0	50	0	0	50	0	0
	Occipital	50	0	0	50	0	0	50	0	0	50	0	0	50	0	0
Right	Frontal	50	0	0	50	0	0	50	0	0	50	0	0	50	0	0
	Parietal	50	0	0	50	0	0	50	0	0	50	0	0	50	0	0
	Temporal	50	0	0	50	0	0	50	0	0	50	0	0	50	0	0
	Occipital	50	0	0	50	0	0	50	0	0	50	0	0	50	0	0
**KPSS results for non-differenced data**																
**Hemisphere**	**Brain Lobe**	**HbOx**			**HHbx**			**tHbx**			**HbDiffx**			**COx-a**		
		**S**	**NS**	**NA**	**S**	**NS**	**NA**	**S**	**NS**	**NA**	**S**	**NS**	**NA**	**S**	**NS**	**NA**
Left	Frontal	37	13	0	39	11	0	36	14	0	42	8	0	43	7	0
	Parietal	40	10	0	42	8	0	41	9	0	34	16	0	35	15	0
	Temporal	40	10	0	37	13	0	41	9	0	41	9	0	38	12	0
	Occipital	34	16	0	40	10	0	40	10	0	34	16	0	34	16	0
Right	Frontal	36	14	0	41	9	0	39	11	0	40	10	0	40	10	0
	Parietal	42	8	0	41	9	0	37	13	0	46	4	0	45	5	0
	Temporal	39	11	0	38	12	0	41	9	0	36	14	0	34	16	0
	Occipital	37	13	0	40	10	0	40	10	0	39	11	0	35	15	0
**KPSS results for 1** ^**st**^ **order differenced data**																
**Hemisphere**	**Brain Lobe**	**HbOx**			**HHbx**			**tHbx**			**HbDiffx**			**COx-a**		
		**S**	**NS**	**NA**	**S**	**NS**	**NA**	**S**	**NS**	**NA**	**S**	**NS**	**NA**	**S**	**NS**	**NA**
Left	Frontal	50	0	0	50	0	0	50	0	0	50	0	0	50	0	0
	Parietal	50	0	0	50	0	0	50	0	0	50	0	0	50	0	0
	Temporal	50	0	0	50	0	0	50	0	0	50	0	0	50	0	0
	Occipital	50	0	0	50	0	0	50	0	0	50	0	0	50	0	0
Right	Frontal	50	0	0	50	0	0	50	0	0	50	0	0	50	0	0
	Parietal	50	0	0	50	0	0	50	0	0	50	0	0	50	0	0
	Temporal	50	0	0	50	0	0	50	0	0	50	0	0	50	0	0
	Occipital	50	0	0	50	0	0	50	0	0	50	0	0	50	0	0

The table presents the results of the ADF and KPSS analysis with the count of subject data that was found to be stationary (S), non-stationary (NS) or unable to assess (NA) using non-differenced and 1^st^ order differenced data sampled at 1 Hz for all the calculated fNIRS indices at each brain lobe of both hemispheres. It was found from these stationarity tests that signals were stationary after 1^st^ order differencing while they were originally non-stationary. ADF, Augmented Dickey-Fuller; COx-a, cerebral oximetry index with arterial blood pressure; fNIRS, functional near-infrared spectroscopy; HbDiffx, hemoglobin difference index; HbOx, oxyhemoglobin index; HHbx, deoxyhemoglobin index; KPSS, Kwiatkowski–Phillips–Schmidt–Shin; NA, unable to assess stationarity; NS, non-stationary; S, stationary; tHbx, total hemoglobin index.

The optimal ARIMA models for all signals were found separately for both frequencies in each subject using the lowest AIC value. Then the median of the optimal ARIMA models was determined for each signal at all four brain lobes. There were subtle differences in both autoregressive and moving average orders for the optimal ARIMA models between brain regions, suggesting our custom-built system had the capacity to detect minor regional variation in CVR at a statistical signal structure level. This held true at both data frequencies analyzed. Overall, the median optimal ARIMA model tended to stay in the lower ARIMA model range for all recorded signals and derived CVR indices, which is similar to prior works in healthy populations using low-resolution commercial systems. The global median optimal ARIMA orders for CVR indices using 1 Hz and 250 Hz sampled data are given in Table 5 and S4d Appendix, with similar results for ABP and fNIRS signals given in S4f Appendix.

**Table 5:** Optimal ARIMA models based on AIC of CVR indices and their hemispheric disparity at 1 Hz.

CVR Index	Hemisphere	Optimal ARIMA Models (Median [IQR])			
		Frontal Lobe	Parietal Lobe	Temporal Lobe	Occipital Lobe
COx-a	Left	(2,1,1) [(1,1,7)–(3,1,5)]	(2,1,2) [(1,1,4)–(3,1,7)]	(2,1,6) [(1,1,5)–(4,1,8)]	(2,1,2) [(1,1,1)–(3,1,4)]
	Right	(3,1,2) [(2,1,1)–(5,1,2)]	(2,1,8) [(1,1,9)–(4,1,7)]	(2,1,6) [(1,1,6)–(4,1,3)]	(2,1,6) [(1,1,6)–(5,1,4)]
HbOx	Left	(2,1,8) [(1,1,5)–(5,1,5)]	(2,1,9) [(1,1,9)–(4,1,3)]	(2,1,8) [(1,1,6)–(4,1,4)]	(2,1,9) [(1,1,1)–(5,1,5)]
	Right	(2,1,1) [(1,1,6)–(4,1,4)]	(2,1,4) [(1,1,6)–(4,1,6)]	(2,1,1) [(1,1,4)–(3,1,4)]	(2,1,6) [(1,1,8)–(4,1,3)]
HHbx	Left	(3,1,2) [(1,1,10)–(6,1,1)]	(1,1,8) [(1,1,3)–(2,1,9)]	(2,1,4) [(1,1,6)–(3,1,8)]	(2,1,1) [(1,1,1)–(3,1,4)]
	Right	(2,1,1) [(1,1,6)–(4,1,7)]	(2,1,5) [(1,1,7)–(4,1,5)]	(2,1,10) [(1,1,5)–(4,1,9)]	(2,1,1) [(1,1,6)–(3,1,6)]
tHbx	Left	(2,1,9) [(1,1,9)–(5,1,2)]	(2,1,2) [(1,1,5)–(3,1,3)]	(3,1,1) [(1,1,7)–(4,1,0)]	(2,1,7) [(1,1,2)–(3,1,10)]
	Right	(2,1,9) [(1,1,9)–(5,1,1)]	(2,1,8) [(1,1,9)–(5,1,1)]	(2,1,7) [(1,1,6)–(4,1,6)]	(2,1,6) [(1,1,7)–(3,1,8)]
HbDiffx	Left	(1,1,9) [(1,1,2)–(3,1,1)]	(2,1,8) [(1,1,8)–(4,1,7)]	(2,1,3) [(1,1,7)–(4,1,4)]	(2,1,2) [(1,1,1)–(3,1,0)]
	Right	(2,1,3) [(1,1,4)–(5,1,2)]	(2,1,9) [(2,1,1)–(4,1,3)]	(2,1,6) [(1,1,7)–(4,1,5)]	(2,1,1) [(1,1,4)–(3,1,7)]

The table provides median and IQR of optimal ARIMA models based on AIC for CVR indices using data in 1 Hz frequency. AIC, Akaike Information Criterion; ARIMA, autoregressive integrative moving average; COx-a, cerebral oximetry index with arterial blood pressure; IQR, interquartile range; HbDiffx, hemoglobin difference index; HbOx, oxyhemoglobin index; HHbx, deoxyhemoglobin index; tHbx, total hemoglobin index.

### 3.5. Hemispheric difference in impulse response function (IRF) of ABP on fNIRS signals – 1 Hz and 250 Hz sampled data

The regional hemispheric disparity was checked in responsiveness of bivariate VARIMA IRF models. In general, across all brain lobes and both sampled frequencies tested, there were subtle differences in IRF responses of ABP on fNIRS signals (rSO_2_, HbO, HHb, tHb, and HbDiff) between left and right hemispheres. It was found that the percentage of patients showing a greater response than 0.1% were mostly above 80% of the population for either direction of the following signal combinations for left and right hemispheres of each brain lobe: ABP & rSO_2_, ABP & HbO, ABP & HHb, ABP & tHb, and ABP & HbDiff. Table 6 provides IRF results for 1 Hz sampled data with analysis for 250 Hz sampled data found in S5a Appendix.

**Table 6:** Hemispheric responsiveness using impulse response coefficients of optimal VARIMA model at 1 Hz.

Direction	Hemisphere	Frontal Lobe [% (count)]		Parietal Lobe [% (count)]		Temporal Lobe [% (count)]		Occipital Lobe [% (count)]	
		>0.1%	NA	>0.1%	NA	>0.1%	NA	>0.1%	NA
ABP → rSO_2_	Left	94% (47)	0% (0)	94% (47)	0% (0)	88% (44)	6% (3)	92% (46)	0% (0)
rSO_2_ → ABP		92% (46)	0% (0)	94% (47)	0% (0)	90% (45)	6% (3)	92% (46)	0% (0)
ABP → rSO_2_	Right	86% (43)	6% (3)	94% (47)	0% (0)	92% (46)	2% (1)	92% (46)	2% (1)
rSO_2_ → ABP		86% (43)	6% (3)	94% (47)	0% (0)	92% (46)	2% (1)	92% (46)	2% (1)
ABP → HbO	Left	92% (46)	0% (0)	86% (43)	0% (0)	84% (42)	2% (1)	94% (47)	2% (1)
HbO → ABP		96% (48)	0% (0)	84% (42)	0% (0)	86% (43)	2% (1)	96% (48)	2% (1)
ABP → HbO	Right	80% (40)	6% (3)	92% (46)	0% (0)	90% (45)	0% (0)	92% (46)	0% (0)
HbO → ABP		82% (41)	6% (3)	92% (46)	0% (0)	90% (45)	0% (0)	94% (47)	0% (0)
ABP → HHb	Left	92% (46)	0% (0)	84% (42)	4% (2)	90% (45)	0% (0)	90% (45)	4% (2)
HHb → ABP		90% (45)	0% (0)	84% (42)	4% (2)	92% (46)	0% (0)	88% (44)	4% (2)
ABP → HHb	Right	94% (47)	0% (0)	86% (43)	0% (0)	88% (44)	2% (1)	86% (43)	6% (3)
HHb → ABP		96% (48)	0% (0)	90% (45)	0% (0)	88% (44)	2% (1)	86% (43)	6% (3)
ABP → tHb	Left	90% (45)	4% (2)	88% (44)	0% (0)	94% (47)	0% (0)	88% (44)	0% (0)
tHb → ABP		92% (46)	4% (2)	88% (44)	0% (0)	94% (47)	0% (0)	84% (42)	0% (0)
ABP → tHb	Right	84% (42)	0% (0)	94% (47)	0% (0)	86% (43)	2% (1)	94% (47)	0% (0)
tHb → ABP		88% (44)	0% (0)	94% (47)	0% (0)	86% (43)	2% (1)	90% (45)	0% (0)
ABP → HbDiff	Left	98% (49)	0% (0)	90% (45)	0% (0)	86% (43)	4% (2)	92% (46)	2% (1)
HbDiff → ABP		98% (49)	0% (0)	90% (45)	0% (0)	90% (45)	4% (2)	92% (46)	2% (1)
ABP → HbDiff	Right	90% (45)	2% (1)	92% (46)	6% (3)	86% (43)	4% (2)	92% (46)	2% (1)
HbDiff → ABP		92% (46)	2% (1)	92% (46)	6% (3)	86% (43)	4% (2)	92% (46)	2% (1)

The table shows the hemispheric responsiveness of fNIRS signals using Impulse Response Coefficients of Optimal VARIMA model using 1 Hz data. ABP, arterial blood pressure; fNIRS, functional near-infrared spectroscopy; HbDiff, hemoglobin difference; HbO, oxyhemoglobin; HHb, deoxyhemoglobin; rSO_2_, regional cerebral oxygen saturation; tHb, total hemoglobin; VARIMA, vector autoregressive integrative moving average.

### 3.6. Hemispheric difference in granger causality between ABP and fNIRS signals – 1 Hz and 250 Hz sampled data

Overall, regardless of data frequency, there were subtle differences in Granger causality testing results between left and right hemispheres for each brain lobe for the ABP on fNIRS signals (HbO, HHb, tHb, HbDiff, and rSO_2_) relationships. A similar directional relationship, often favouring ABP leading to directional change in fNIRS signals was seen in both left and right hemispheres of all brain lobes. Table 7 provides analysis results for 1 Hz sampled data, with analysis for the 250 Hz sampled data found in S5b Appendix.

**Table 7:** Granger causal directionality results based on greater F-statistic at 1 Hz.

Signal	Direction	Hemisphere	Frontal Lobe [% (count)]	Parietal Lobe [% (count)]	Temporal Lobe [% (count)]	Occipital Lobe [% (count)]
ABP & rSO_2_	ABP → rSO_2_	Left	44% (22)	48% (24)	68% (34)	40% (20)
	rSO_2_ → ABP		56% (28)	52% (26)	32% (16)	60% (30)
	ABP → rSO_2_	Right	48% (24)	62% (31)	44% (22)	54% (27)
	rSO_2_ → ABP		52% (26)	38% (19)	56% (28)	46% (23)
ABP & HbO	ABP → HbO	Left	44% (22)	54% (27)	54% (27)	50% (25)
	HbO → ABP		56% (28)	46% (23)	46% (23)	50% (25)
	ABP → HbO	Right	58% (29)	56% (28)	52% (26)	60% (30)
	HbO → ABP		42% (21)	44% (22)	48% (24)	40% (20)
ABP & HHb	ABP → HHb	Left	50% (25)	52% (26)	50% (25)	52% (26)
	HHb → ABP		50% (25)	48% (24)	50% (25)	48% (24)
	ABP → HHb	Right	58% (29)	52% (26)	58% (29)	56% (28)
	HHb → ABP		42% (21)	48% (24)	42% (21)	44% (22)
ABP & tHb	ABP → tHb	Left	48% (24)	42% (21)	50% (25)	52% (26)
	tHb → ABP		52% (26)	58% (29)	50% (25)	48% (24)
	ABP → tHb	Right	44% (22)	52% (26)	50% (25)	60% (30)
	tHb → ABP		56% (28)	48% (24)	50% (25)	40% (20)
ABP & HbDiff	ABP → HbDiff	Left	44% (22)	54% (27)	46% (23)	48% (24)
	HbDiff → ABP		56% (28)	46% (23)	54% (27)	52% (26)
	ABP → HbDiff	Right	46% (23)	66% (33)	50% (25)	52% (26)
	HbDiff → BP		54% (27)	34% (17)	50% (25)	48% (24)

The table shows the Granger causal directionality results between ABP and fNIRS signals using 1 Hz data. ABP, arterial blood pressure; fNIRS, functional near-infrared spectroscopy; HbDiff, hemoglobin difference; HbO, oxyhemoglobin; HHb, deoxyhemoglobin; rSO_2_, regional cerebral oxygen saturation; tHb, total hemoglobin.

### 3.7. Subgroup analysis assessment – Common parameters and perturbations

Subgroup analysis across both frequencies occurred using the common indicators outlined in the Methods (section 2.5.6), with all results outlined in detailed tables found in S6 Appendix along with the ANAM results subgrouped using these common indicators. Overall, across all categories, subtle differences were discernable between left and right hemispheres of brain lobes within each individual sub-group analyzed, similar to the full recording analysis. In the median/IQR results of CVR indices, the significant p-values found were between parietal lobes in the Age 40 – 60 subgroup using HHbx and tHbx indices and occipital lobes using MAD of all five CVR indices. Regarding median/IQR of other physiologic signals in majority of subgroups, significant p-values were often seen between all brain lobes using rSO_2_ and HbDiff signals, temporal and occipital brain lobes for HbO, and only occipital lobes for HHb and tHb signals. Similar regional hemispheric disparities (ARHD) of signals, MAD of signals’ ARHD, median optimal ARIMA models were found amongst the subgroups. The subgrouped VARIMA IRF analysis presented that a higher population percentage (>~80%) showed a greater response than 0.1% in either direction (ABP → fNIRS signal and fNIRS signal → ABP) and often the ABP → fNIRS signal direction was slightly favoured in all subgroups. Lastly, the Granger causality results showed that, across subgroups, the hemispheric ABP on the constituents of CVR indices were generally similar and the directionality of ABP Granger causes fNIRS signals was often favoured.

Subgroup analysis using perturbation subdivisions across both frequencies occurred as outlined in the Methods (Section 2.5.6), with all results outlined in detailed tables found in S7 Appendix. Overall, across all categories, subtle differences were noticeable between left and right hemispheres, for all brain lobes, within each analyzed baseline/perturbation subgroup. Similarly, noticeable changes in CVR measures in a given brain region were seen, comparing baseline recordings to subsequent different perturbation categories, suggesting our custom systems ability to pick up live physiologic change over time. In the median/IQR results of CVR indices, the significant p-values were often found between occipital lobes for MAD of all five CVR indices in each perturbation subgroup. Regarding median/IQR of other physiologic signals in majority of perturbation subgroups, significant p-values were often seen between all brain lobes using rSO_2_ and HbDiff signals, all brain lobes excluding parietal lobes for HbO signal, and only occipital lobes for HHb and tHb signals. Similar regional hemispheric disparities were found amongst the perturbation subgroups along with their MAD. Similar regional hemispheric disparities (ARHD) of signals, MAD of signals’ ARHD, median optimal ARIMA models were found amongst the subgroups. The subgrouped VARIMA IRF analysis presented that only the neurovascular coupling subgroup had a higher population percentage (≥~80%) which showed a greater response than 0.1% in either direction (ABP → fNIRS signal and fNIRS signal → ABP), while the orthostatic challenge, vascular chemo-reactivity, and baseline subgroups showed a lower percentage (≥72%, ≥ 62%, and ≥52%, respectively). Lastly, the Granger causality results showed that, across perturbation subgroups, the hemispheric ABP on the constituents of CVR indices were generally similar and the directionality of ABP Granger causes fNIRS signals was also often favoured.

## 4. Discussion

In healthy volunteers, the multi-regional hemispheric disparity in CA/CVR was assessed using fNIRS-derived indices (COx-a, HbOx, HHbx, tHbx, and HbDiffx) which employed various statistical methods including: A. entire-recording median summary metrics for fNIRS signals (rSO_2_/HbO/HHb/tHb/HbDiff) and fNIRS-derived indices (COx-a/HbOx/HHbx/tHbx/HbDiffx), B. ARHD/MAD of fNIRS signals and derived indices data streams, C. time-series optimal ARIMA structures of fNIRS signals and derived indices data streams, D. VARIMA IRF analysis of the ABP on rSO_2_/HbO/HHb/tHb/HbDiff relationships (i.e., constituents of fNIRS-based COx-a/HbOx/HHbx/tHbx/HbDiffx indices), and E. Granger causality testing of the ABP on rSO_2_/HbO/HHb/tHb/HbDiff relationships (i.e., constituents of fNIRS-based COx-a/HbOx/HHbx/tHbx/HbDiffx indices) in two data frequencies (1 Hz and 250 Hz). These advanced time-series analyses were employed to fully characterize the temporal dynamics underlying CVR to quantify the regional hemispheric differences. ARIMA models helped capture the intrinsic autoregressive and moving average structures of the fNIRS and ABP signals. VARIMA models with IRF extended this framework to the multivariate domain, allowing the examination of how an impulse to a constituent signals of the calculated CVR indices propagates over time to influence the other signal in the regional and hemispheric space and vice versa. Granger causality analysis evaluated the bidirectional influence on the constituents signals of the calculated CVR indices to provide insight into regional hemispheric difference in the causality of a fNIRS signal Granger causing ABP and vice versa. Together, these advanced time-series methods provided complementary insights into the regional hemispheric differences of CVR by modeling signal dynamics, interactions, and causal relationships. This analytical approach represents the most comprehensive regional hemispheric disparity analysis using high-frequency cerebral fNIRS data streams and through the exhaustive analysis, some important features regarding high-resolution cerebral fNIRS data streams were highlighted.

First, subtle differences between brain lobes (regional disparity), single hemisphere (intra-hemispheric disparity), and two hemispheres (hemispheric disparity) were found in the ARHD, ARIMA, VARIMA IRF, and Granger causality analyses using COx-a, HbOx, HHbx, tHbx, and HbDiffx CA/CVR indices and its constituent signals. Although there was a lack of significant regional and hemispheric differences found in the grand median summary metrics, with a median near zero indicating intact CA/CVR, this result was not unexpected based on our prior systematic review and retrospective study on commercial NIRS system where the grand median values in healthy volunteers did not show a regional or hemispheric difference using short duration recordings [37,39]. The subtle regional and hemispheric disparities seen in the conducted array of statistical analyses held true during the common parameters subgroup but was more prevalent in the perturbation subgroup analyses which demonstrated the live change and response to each perturbation test compared to the observed baseline. This further supports the utility of our custom platform to detect subtle regional, intra-hemispheric, and inter-hemispheric differences in cerebral physiology and CA/CVR. In unilateral brain pathology population, there is potential for spatially resolved CA/CVR techniques, such as our custom platform, to discern regional hemispheric disparity as indicated in previous literature reviews [34,37,49–52], as our previous study showed that commercial, low-frequency (1 Hz) cerebral NIRS systems may not be able to reliably discern hemispheric disparity in cranial trauma (i.e., TBI) population which had been compared with healthy volunteer and elective spinal surgery (without intradural work) populations [39]. Hence, the observed regional, intra-hemispheric, and inter-hemispheric differences across perturbation tests demonstrate that the fNIRS system can detect subtle spatial variations in CVR responses to controlled perturbation in healthy population with intact CA/CVR. This supports potential clinical applicability of such fNIRS systems in cranial trauma populations, where observation of larger hemispheric and lobar disparities is expected to indicate impaired CA/CVR.

Second, using decimated data from both 1 Hz (mean down-sampled) and 250 Hz (up-sampled) sampled data, these subtle regional, intra-hemispheric, and inter-hemispheric differences held true in the thoroughly conducted signal statistical structures and modeling in full recording and subgrouped analyses. Although there were significant p-values found for the MAD of fNIRS signals/indices throughout the analyses, these could be caused due to the optodes for the brain lobe (usually the occipital lobe) failing to maintain a consistent contact with the scalp region due to poor cap conformity over that region during different movements involved while performing the perturbations. Also, the median EtCO_2_ and respiratory rate was similar for all baseline/perturbations except for the vascular chemo-reactivity perturbation group where a small decrease of these median signals was seen as it was expected and probably attributed by the normal, slow, and fast breathing challenges for the perturbation. These findings align with the prior literature which suggests that high-frequency fNIRS systems may be more useful than commercial system to detect subtle regional hemispheric variations. Also, the mean down-sampling can retain statistical features which is important in achieving more manageable data size to simplify high-frequency physiological signals and increase computational efficiency as indicated in the previous original study from our lab [38].

Third, gross derangements in CA/CVR or massive regional hemispheric differences were not seen in response to various perturbations employed. It was expected to only see subtle but consistent differences as a response to the perturbations as CA/CVR is finely regulated in the normal state of healthy cohort without any known history of cardiovascular or neurological conditions. These conditions would be prevalent in diseased/injured human cohorts where we would expect to see gross derangements in CA/CVR along with massive lobar differences. These findings emphasize that disturbing the CA/CVR in healthy population with various perturbations (neurovascular coupling, orthostatic challenge, and vascular chemo-reactivity) shows a subtle regional hemispheric disparity of CA/CVR as measured via the high-frequency fNIRS system at both sampled frequencies using our custom platform and is in keeping with established literature [35,37,53]. Now the question remains if these high-frequency research-grade fNIRS systems can show regional hemispheric disparity in cranial trauma populations where regional and hemispheric differences in injury patterns are prevalent.

Fourth, the high-frequency whole-brain fNIRS system improves upon existing commercial NIRS systems by increasing the temporal and spatial resolutions along with providing improved regional/hemispheric CVR sensitivity. Our custom whole brain fNIRS-based mapping system allows recording fNIRS signals at the high-frequency of 250 Hz compared to commercial NIRS systems that have a much lower temporal resolution of <100 Hz, but often it is seen to be near ~1 Hz. The spatial resolution has been improved to separately assess each brain lobe of both hemispheres using a total of 14 channels (8 normal and 8 short channels) and since the layouts are customizable, a 24 channel grid can be created using 8 normal channels with possibility to increase the spatial resolution by connecting additional optodes [41]. Whereas the commercial NIRS systems often employ only 2 channels to assess only the frontal brain lobes [31]. The improvement in regional/hemispheric CVR sensitivity seen using the custom system is a result of full waveform fNIRS signals being recorded which provides a deeper insight into brain hemodynamics via better statistical features present in the signals. These features seem to be retained after data decimation to focus on slow-wave vasogenic fluctuations associated with CA [38]. In comparison, commercial systems have not been able to discern hemispheric disparity for the frontal lobes in cranial trauma populations where CVR disparity should exist [39].

Lastly, our findings are consistent with the current human literature on regional hemispheric disparity in continuously measured time-domain CVR indices. In our systematic review of prior literature [37], grand median summary metrics did not show regional disparity which is consistent with results of our retrospective study using commercial NIRS [39] and results of our current study using high-frequency fNIRS system. With our high spatial and temporal setup, subtle regional, intra-hemispheric, and inter-hemispheric differences were found and compared to the current literature, often minimal to no differences have been found often using low-frequency dual-channel commercial NIRS systems probing the frontal lobes [37,53]. Although endarterectomy and subarachnoid hemorrhage studies reported significant hemispheric difference but varying results were seen in stroke, TBI, and multiple population studies often using dual-channel low-frequency commercial NIRS systems probing the frontal lobes [33–35,37,50,54]. To our knowledge, this study has conducted the most comprehensive regional hemispheric disparity analysis on between regional, intra-hemispheric, and inter-hemispheric differences of the brain lobes during perturbations in a healthy population and often, studies only look at the hemispheric disparity of the frontal lobes. To properly contextualize the observed regional hemispheric differences in CVR, a clinically interpretable metric is non-existent. Given that the CVR index ranges from -1 to +1, a 10% difference (0.2 CVR index difference) may be considered high. The ARHD was typically below 10% in our previous work with commercial NIRS system on multiple populations [39] and in contrast, the ARHD in the present study slightly exceeded this 10% threshold, indicating the subtle but measurable differences of CVR in the healthy perturbed population. As we have found subtle differences in a healthy perturbed population, the high-frequency fNIRS systems has a huge potential to reliably detect the regional hemispheric differences in various neuropathological states such as endarterectomy, subarachnoid hemorrhage, stroke, and TBI. Although it is difficult to compare regional hemispheric disparity between different modalities, given the diverse natural of cerebral vascular compartments they measure at varying depths and spatial resolutions, our lab is currently planning the next phase of system assessments. This phase will compare our fNIRS platform with field standard fixed neuroimaging systems, such as functional magnetic resonance imaging (fMRI) and/or magnetic resonance parameters (MRP), using another block-trial design. These assessment will evaluate the ability of our fNIRS systems to discern regional and hemispheric differences not only in healthy populations but also in a range of pathological states, such as post concussion, mild TBI, subacute or long-term moderate/severe TBI survivors.

## 5. Limitations

Due to the analysis exploratory approach, it entails several overarching limitations. First, although the healthy volunteer cohort was reasonably sized (>40 participants) [55] for the prospective analysis, these findings should only be considered exploratory and be taken with caution. Second, as these findings may not be generalizable to other healthy populations (with or without perturbations), it warrants validation in larger multi-center high-frequency (≥100 Hz) physiologic datasets. Third, although steps were taken to choose the optimal fNIRS cap fit by measuring a subject’s head circumference according to the manufacturer, the optode-to-scalp contact on the occipital lobe regions were poor. Either during the entire recording of the subject or during movement pertaining to the various perturbations, the cap conformity in the occipital lobe regions were suboptimal and was the major reason the results for these regions showed significant difference in MAD of all calculated CVR indices. Fourth, correction for multiple comparisons were not performed as this was an exploratory analysis of data of the prospective observational study. As large number of statistical tests performed across signals in multiple brain regions, hemispheres, and subgroups may heighten the risk of Type I error inflation, these findings should be taken with caution. Fifth, decimation of data to focus on slow-wave (i.e., 0.05–0.005 Hz) vasogenic fluctuations using non-overlapping moving average filtering provides limited control over passband characteristics, aliasing, and phase behaviour. These effects were not explicitly evaluated as this was an exploratory study. Future studies should explore techniques such as linear-phase finite impulse response low-pass filters followed by signal decimation to analyze if it is able to provide better signal features due to minimized aliasing. Sixth, although the employed linear approaches (ARIMA and VARIMA) did seem to characterize the complexity of cerebral physiologic signals during various perturbations, exploring other hybrid or nonlinear approaches for complex time-series modeling which may yield additional insights along with investigating nonlinear dynamics and causal interactions. Seventh, evaluation of the high-frequency fNIRS system on population with clear disparity and gross derangements likely exist was not evaluated as this type of dataset does not currently exist but the creation of such dataset is in works at our lab. Eighth, the recordings from a single high-frequency research-grade fNIRS system were analysed which can be a potential bias. Finally, it is challenging to verify the subtle regional hemispheric disparities detected with fNIRS against other modalities due to the lack of gold-standard CVR measurement as different modalities (fMRI, Transcranial Doppler, and NIRS/fNIRS) assess CVR using different physiological domains (perfusion/oxygenation, macrovascular flow, or microvascular oxygenation, respectively) [56]. Consequently, their measurement of regional hemispheric disparity may diverge due to differences in cerebrovascular compartment, measurement cadence, depth sensitivity, and spatial resolution; although global hemispheric disparity may be detected by all these modalities.

## 6. Conclusion

With our novel custom-built fNIRS CVR mapping system, measured and derived signals demonstrated subtle but reproducible differences in CA/CVR between brain regions and hemispheres using 1 Hz mean down-sampling and 250 Hz up-sampling of data. This was the case across several different time-domains statistical methods that were applied, and during baseline and perturbation testing. These findings are in alignment with prior literature, supporting the notion that research-grade fNIRS systems may be adequate for regional disparity analysis of CVR in humans. Future work in diseased/injured human cohorts is required to further quantify the sensitivity of our custom-built system to detect regional variations and disturbances in CVR.

## Supporting information

S1 Appendix Methodology. (DOCX)

S2 Appendix Signal median and interquartile range. (DOCX)

S3 Appendix Regional hemispheric disparity analysis. (DOCX)

S4 Appendix Autoregressive integrative moving average (ARIMA) analysis. (DOCX)

S5 Appendix VARIMA IRF and granger causality analyses. (DOCX)

S6 Appendix Common parameters subgroup analysis. (DOCX)

S7 Appendix Perturbation subgroup analysis. (DOCX)
