# Supplementary material for: Detection of regional disparity in cerebrovascular reactivity using a custom whole brain functional near-infrared spectroscopy based mapping system: A prospective observational study
Source: PLOS Digit Health. 2026 Apr 15;5(4):e0001349. doi: 10.1371/journal.pdig.0001349 (PMC13082728; doi:10.1371/journal.pdig.0001349)
Supplement: S1 Appendix — (DOCX) [file pdig.0001349.s001.docx]

**Appendix S1 – Methodology**

Appendix S1 – Table of Contents

[Appendix S1a: Evaluation of Data Stationarity 2](#_Toc213065770)

[Appendix S1b: Evaluation of optimal Autoregressive Integrated Moving Average (ARIMA) orders 3](#_Toc213065771)

[Appendix S1c: Generation of Vector Autoregressive Integrated Moving Average (VARIMA) model 4](#_Toc213065772)

[Appendix S1d: References 5](#_Toc213065773)

Appendix S1a: Python Code Example – Calculation of CVR Index

frequency = 250 # 250 Hz or 1 Hz sampling frequency

update_ma = 10; # 10 seconds

update_corr = 300; # 300 seconds; 5 minutes of data

window_ma = frequency * update_ma # calculate moving average window points based on frequency

window_corr = frequency * update_corr // window_ma # calculate correlation window based on frequency

decimated_abp = series_abp.rolling(window_ma, min_periods=window_ma //2).mean()[window_ma -1:: window_ma]

decimated_fnirs = series_fnirs_abp.rolling(window_ma, min_periods=window_ma //2).mean()[window_ma -1:: window_ma]

signal_cvr = decimated_abp.rolling(window_corr, min_periods=window_corr //2).corr(decimated_fnirs)

Appendix S1b: Evaluation of Data Stationarity

Stationarity analysis was performed for each physiologic signal at an individual level using Augmented Dickey-Fuller (ADF) and Kwiatkowski-Phillips-Schmidt-Shin (KPSS) tests for the 1 Hz and 250 Hz sampled data, in keeping with previous work from our group. The ADF test informs if the series is trend-stationary and KPSS test informs if the series is stationary around a linear trend.^1^ The ADF and KPSS tests were run on each subject’s data using the “adfuller” and “kpss” functions from the *statsmodels* package (<https://www.statsmodels.org/stable/index.html>). The data from populations were 1^st^ order differenced, and the above-mentioned stationarity analysis was re-run on each differenced data in both the 1 Hz and 250 Hz frequencies to make certain that the data was strictly stationary before any sort of data modeling.

Appendix S1c: Evaluation of optimal Autoregressive Integrated Moving Average (ARIMA) orders

Using Python, Autoregressive Integrated Moving Average (ARIMA) model fit was performed in accordance with standard Box-Jenkin’s time-series methodologies.^1–3^ Various ARIMA models were fit to each univariate physiologic time-series to model every 1^st^ order differenced signal for each subject in 1 Hz and 250 Hz sampling frequencies. The Akaike Information Criterion (AIC) value was obtained for ARIMA model fit on all physiologic variables (arterial blood pressure [ABP], regional oxygen saturation [rSO_2_], oxyhemoglobin [HbO], deoxyhemoglobin [HHb], total hemoglobin [tHb], difference between HbO and HHb [HbDiff], cerebral oximetry index derived with ABP [COx-a], HbO index [HbOx], HHb index [HHbx], tHb index [tHbx], and HbDiff index [HbDiffx]) where available since it is neither stringent or lenient as compared to other performance values.^4–7^ Based on previous research from our lab,^4–7^ the list of ARIMA models were obtained by varying the autoregressive order (p-order), and the moving average order (q-order) from 1 to 10, and 0 to 10, respectively. The integrative order (d-order) was separately set to 1 using the previously described differencing method. To find the optimal ARIMA model for each physiologic time-series in various sampled frequencies, the lowest AIC values were extracted for a subject. Finally, by reordering the optimal ARIMA models sequentially, the median optimal ARIMA model for a physiologic signal in each sampled frequency was found using AIC.

Appendix S1d: Generation of Vector Autoregressive Integrated Moving Average (VARIMA) model

To represent the relationship between two types of signals, we derived vector ARIMA (VARIMA) models. The p-order for the VARIMA model was calculated by taking a product of the previously saved optimal ARIMA p-orders for the two signals being evaluated and the VARIMA q-order was calculated by adding the previously saved optimal ARIMA q-orders for the two signals being evaluated, as suggested from past literature.^3,7^ The VARIMA d-order was equal to one since 1^st^ order differenced data was used. This created a personalized VARIMA model for each 1^st^ order differenced signal pair in a subject’s data for 1 Hz and 250 Hz sampling frequencies.

Appendix S1e: References

1. Chatfield, C. & Xing, H. *The Analysis of Time Series: An Introduction with R*. (Chapman and Hall/CRC, 2019).

2. Chatfield, C. *The Analysis of Time Series: An Introduction*. (Chapman and Hall/CRC, New York, 2003). doi:10.4324/9780203491683.

3. Lütkepohl, H. *New Introduction to Multiple Time Series Analysis*. (New York : Springer, Berlin, 2005).

4. Sainbhi, A. S. *et al.* Time-Series autocorrelative structure of cerebrovascular reactivity metrics in severe neural injury: An evaluation of the impact of data resolution. *Biomedical Signal Processing and Control* **95**, 106403 (2024).

5. Sainbhi, A. S. *et al.* Commercial NIRS May Not Detect Hemispheric Regional Disparity in Continuously Measured COx/COx-a: An Exploratory Healthy and Cranial Trauma Time-Series Analysis. *Bioengineering* **12**, 247 (2025).

6. Sainbhi, A. S. *et al.* Time-Series Autoregressive Models for Point and Interval Forecasting of Raw and Derived Commercial Near-Infrared Spectroscopy Measures: An Exploratory Cranial Trauma and Healthy Control Analysis. *Bioengineering* **12**, 682 (2025).

7. Sainbhi, A. S. *et al.* Time-Domain Analysis of Low- and High-Frequency Near-Infrared Spectroscopy Sensor Technologies for Characterization of Cerebral Pressure–Flow and Oxygen Delivery Physiology: A Prospective Observational Study. *Sensors* **25**, 5391 (2025).
