# Supplementary material for: Detection of regional disparity in cerebrovascular reactivity using a custom whole brain functional near-infrared spectroscopy based mapping system: A prospective observational study
Source: PLOS Digit Health. 2026 Apr 15;5(4):e0001349. doi: 10.1371/journal.pdig.0001349 (PMC13082728; doi:10.1371/journal.pdig.0001349)
Supplement: S2 Appendix — (DOCX) [file pdig.0001349.s002.docx]

**Appendix S2 – Signal Median and Interquartile Range**

Appendix S2 – Table of Contents

[Appendix S2a: Median and IQR of CVR Indices using 10-Second Decimated Data Sampled at 250 Hz 2](#_Toc213065925)

[Appendix S2b: Median and IQR of Physiologic Signals 10-Second Decimated Data 3](#_Toc213065926)

[Appendix S2c: Median and IQR of Physiologic Signals Raw Data 4](#_Toc213065927)

[Appendix S2d: Percent Time Results of rSO_2_ and CVR Indices Using 10-Second Decimated Data 5](#_Toc213065928)

[Appendix S2e: Percent Time Results of rSO_2_ Using Raw Data 6](#_Toc213065929)

Appendix S2a: Median and IQR of CVR Indices using 10-Second Decimated Data Sampled at 250 Hz

| **CVR Index** | **Brain Lobe** | **Median (IQR)** | | **p-value** |
| --- | --- | --- | --- | --- |
|  |  | **Left Hemisphere** | **Right Hemisphere** |  |
| COx-a (au) | Frontal | -0.01 (-0.22 – 0.18) | 0 (-0.19 – 0.21) | 0.306 |
|  | Parietal | 0 (-0.2 – 0.2) | -0.01 (-0.2 – 0.19) | 0.6516 |
|  | Temporal | 0 (-0.2 – 0.19) | 0.01 (-0.18 – 0.2) | 0.9423 |
|  | Occipital | 0.01 (-0.14 – 0.15) | -0.01 (-0.19 – 0.19) | 0.796 |
| HbOx (au) | Frontal | -0.01 (-0.21 – 0.18) | 0.01 (-0.2 – 0.22) | 0.054 |
|  | Parietal | 0 (-0.2 – 0.22) | 0.01 (-0.19 – 0.22) | 0.5979 |
|  | Temporal | 0.01 (-0.2 – 0.18) | 0.02 (-0.19 – 0.2) | 0.4299 |
|  | Occipital | 0.02 (-0.15 – 0.17) | 0.01 (-0.18 – 0.2) | 0.5464 |
| HHbx (au) | Frontal | 0 (-0.19 – 0.21) | 0.02 (-0.19 – 0.23) | 0.8605 |
|  | Parietal | 0 (-0.21 – 0.2) | 0.02 (-0.17 – 0.22) | 0.1506 |
|  | Temporal | 0 (-0.19 – 0.19) | 0.01 (-0.19 – 0.21) | 0.4713 |
|  | Occipital | 0.01 (-0.14 – 0.16) | 0.01 (-0.18 – 0.21) | 0.9478 |
| tHbx (au) | Frontal | -0.01 (-0.22 – 0.19) | 0.01 (-0.18 – 0.23) | 0.3502 |
|  | Parietal | 0 (-0.2 – 0.21) | 0 (-0.18 – 0.24) | 0.1669 |
|  | Temporal | 0 (-0.19 – 0.19) | 0.01 (-0.2 – 0.21) | 0.7277 |
|  | Occipital | 0.01 (-0.14 – 0.18) | 0 (-0.19 – 0.21) | 0.8822 |
| HbDiffx (au) | Frontal | 0 (-0.22 – 0.18) | 0.01 (-0.18 – 0.23) | 0.1356 |
|  | Parietal | 0.01 (-0.19 – 0.2) | -0.01 (-0.2 – 0.19) | 0.5602 |
|  | Temporal | 0.01 (-0.19 – 0.19) | 0.01 (-0.19 – 0.2) | 0.7226 |
|  | Occipital | 0.01 (-0.14 – 0.17) | 0 (-0.19 – 0.19) | 0.9368 |
| MAD of COx-a (au) | Frontal | 0.21 (0.18 – 0.23) | 0.21 (0.18 – 0.23) | 0.9149 |
|  | Parietal | 0.19 (0.16 – 0.21) | 0.21 (0.17 – 0.23) | 0.0747 |
|  | Temporal | 0.2 (0.16 – 0.22) | 0.2 (0.16 – 0.22) | 0.9862 |
|  | Occipital | 0.14 (0.12 – 0.16) | 0.2 (0.17 – 0.22) | **<0.001** |
| MAD of HbOx (au) | Frontal | 0.19 (0.17 – 0.22) | 0.21 (0.18 – 0.24) | 0.0523 |
|  | Parietal | 0.21 (0.18 – 0.22) | 0.21 (0.18 – 0.24) | 0.7433 |
|  | Temporal | 0.19 (0.17 – 0.23) | 0.2 (0.16 – 0.22) | 0.4971 |
|  | Occipital | 0.16 (0.13 – 0.19) | 0.19 (0.17 – 0.22) | **<0.001** |
| MAD of HHbx (au) | Frontal | 0.21 (0.18 – 0.23) | 0.2 (0.17 – 0.24) | 0.7329 |
|  | Parietal | 0.19 (0.16 – 0.23) | 0.21 (0.19 – 0.23) | 0.2426 |
|  | Temporal | 0.2 (0.16 – 0.22) | 0.19 (0.17 – 0.23) | 0.5556 |
|  | Occipital | 0.15 (0.13 – 0.17) | 0.19 (0.17 – 0.23) | **<0.001** |
| MAD of tHbx (au) | Frontal | 0.2 (0.17 – 0.22) | 0.2 (0.17 – 0.24) | 0.759 |
|  | Parietal | 0.2 (0.17 – 0.23) | 0.22 (0.18 – 0.24) | 0.4586 |
|  | Temporal | 0.2 (0.18 – 0.23) | 0.2 (0.17 – 0.23) | 0.8822 |
|  | Occipital | 0.17 (0.14 – 0.2) | 0.2 (0.17 – 0.22) | **<0.001** |
| MAD of HbDiffx (au) | Frontal | 0.2 (0.18 – 0.23) | 0.21 (0.17 – 0.23) | 0.5742 |
|  | Parietal | 0.18 (0.16 – 0.21) | 0.2 (0.17 – 0.22) | 0.0652 |
|  | Temporal | 0.19 (0.15 – 0.22) | 0.2 (0.16 – 0.22) | 0.3328 |
|  | Occipital | 0.15 (0.12 – 0.18) | 0.2 (0.17 – 0.22) | **<0.001** |
| The p-values in the table are derived using Mann-Whitney U test between the bilateral signals. *COx-a, cerebral oximetry index with arterial blood pressure; CVR, cerebrovascular reactivity index; HbDiffx, hemoglobin difference index; HbOx, oxyhemoglobin index; HHbx, deoxyhemoglobin index; IQR, interquartile range; MAD, median absolute deviation; tHbx, total hemoglobin index.* | | | | |

Appendix S2b: Median and IQR of Physiologic Signals 10-Second Decimated Data

| **Physiologic Signal** | **Brain Lobe** | **Median (IQR)** | | | | | |
| --- | --- | --- | --- | --- | --- | --- | --- |
|  |  | **1 Hz Sampled Data** | | | **250 Hz Sampled Data** | | |
|  |  | **Left Hemisphere** | **Right Hemisphere** | **p-value** | **Left Hemisphere** | **Right Hemisphere** | **p-value** |
| ABP (mmHg) | – | 95.24 (89.41 – 101.36) | | – | 95.13 (89.58 – 101.14) | | – |
| EtCO_2_ (mmHg) | – | 35.44 (34.18 – 36.91) | | – | 35.42 (34.13 – 36.93) | | – |
| RR (bpm) | – | 17.67 (14.43 – 20) | | – | 17.69 (14.46 – 20) | | – |
| rSO_2_ (%) | Frontal | 45.25 (44.59 – 46.11) | 40.58 (39.59 – 41.21) | **<0.001** | 45.25 (44.59 – 46.12) | 40.58 (39.56 – 41.21) | **<0.001** |
|  | Parietal | 48.55 (47.24 – 50.56) | 45.51 (44.18 – 47.18) | **0.0015** | 48.55 (47.24 – 50.55) | 45.5 (44.16 – 47.18) | **0.0015** |
|  | Temporal | 40.52 (38.7 – 42.04) | 48.06 (46.75 – 48.93) | **<0.001** | 40.52 (38.7 – 42.04) | 48.02 (46.75 – 48.96) | **<0.001** |
|  | Occipital | 35.93 (35.59 – 36.36) | 46.73 (46.09 – 48.08) | **<0.001** | 36.22 (35.81 – 36.62) | 46.73 (46.08 – 48.09) | **<0.001** |
| HbO (au) | Frontal | -119.79 (-128.35 – -114.25) | -127.87 (-136.29 – -123.25) | 0.0725 | -119.8 (-128.35 – -114.23) | -127.77 (-136.31 – -123.25) | 0.0759 |
|  | Parietal | -173.14 (-187.45 – -158.57) | -184.97 (-207.81 – -170.37) | 0.1526 | -173.14 (-187.45 – -158.13) | -184.92 (-208.07 – -170.47) | 0.1546 |
|  | Temporal | -211.54 (-228.61 – -201.43) | -154.98 (-166.94 – -141.81) | **0.0215** | -211.48 (-228.33 – -201.4) | -155.02 (-167.16 – -141.91) | **0.0215** |
|  | Occipital | -495.44 (-505.37 – -485.04) | -188.11 (-209.04 – -172) | **<0.001** | -495.29 (-505.41 – -484.99) | -188.15 (-208.68 – -172.02) | **<0.001** |
| HHb (au) | Frontal | -109.79 (-111.34 – -105.23) | -85.83 (-94.48 – -78.42) | 0.083 | -109.67 (-111.3 – -105.36) | -85.86 (-94.48 – -78.45) | 0.083 |
|  | Parietal | -169.5 (-195.68 – -157.24) | -163.27 (-184.2 – -147.61) | 0.7329 | -169.55 (-195.58 – -157.29) | -163.41 (-184.22 – -147.66) | 0.7485 |
|  | Temporal | -143.81 (-161.42 – -134.16) | -148.25 (-157.19 – -131.58) | 0.5147 | -143.53 (-161.39 – -134.15) | -148.36 (-157.14 – -131.55) | 0.5147 |
|  | Occipital | -269.41 (-278.85 – -263.84) | -167.23 (-183.89 – -154.55) | **<0.001** | -269.07 (-278.97 – -263.88) | -167.18 (-183.56 – -154.55) | **<0.001** |
| tHb (au) | Frontal | -226.5 (-241.38 – -214.39) | -215.44 (-231.77 – -198.82) | 0.7226 | -226.51 (-241.41 – -214.38) | -215.18 (-231.84 – -198.74) | 0.7174 |
|  | Parietal | -340.49 (-381.42 – -318.28) | -346.95 (-388.74 – -323.38) | 0.5789 | -340.67 (-381.34 – -317.94) | -346.92 (-387.91 – -323.36) | 0.5789 |
|  | Temporal | -355.86 (-383.91 – -340.3) | -308.52 (-323.22 – -272.97) | 0.29 | -355.82 (-383.99 – -340.29) | -308.56 (-323.29 – -272.69) | 0.2931 |
|  | Occipital | -763.32 (-792.43 – -750.19) | -354.88 (-394.9 – -335.19) | **<0.001** | -763.26 (-792.49 – -750.24) | -354.98 (-394.74 – -335.21) | **<0.001** |
| HbDiff (au) | Frontal | -19.39 (-21.85 – -14.01) | -44.33 (-48.95 – -38.37) | **<0.001** | -19.35 (-21.84 – -13.98) | -44.32 (-48.93 – -38.43) | **<0.001** |
|  | Parietal | -8.67 (-17.28 – 2.79) | -29.3 (-35.94 – -19.15) | **0.002** | -8.7 (-17.07 – 2.78) | -29.29 (-35.96 – -19.1) | **0.002** |
|  | Temporal | -59.68 (-71.94 – -50.37) | -8.08 (-18.22 – -5.57) | **<0.001** | -59.73 (-71.93 – -50.35) | -8.08 (-18.23 – -5.55) | **<0.001** |
|  | Occipital | -220.22 (-227.55 – -212.04) | -21.55 (-25.81 – -11.94) | **<0.001** | -220.7 (-227.39 – -212.03) | -21.56 (-25.85 – -11.94) | **<0.001** |
| MAD of ABP (mmHg) | – | 5.61 (4.87 – 6.7) | | – | 5.5 (4.82 – 6.63) | | – |
| MAD of EtCO_2_ (mmHg) | – | 1.22 (1 – 1.54) | | – | 1.23 (1 – 1.56) | | – |
| MAD of RR (bpm) | – | 2.3 (1.99 – 2.98) | | – | 2.29 (1.99 – 2.99) | | – |
| MAD of rSO_2_ (%) | Frontal | 0.71 (0.47 – 0.92) | 0.8 (0.59 – 1.12) | 0.1487 | 0.71 (0.48 – 0.95) | 0.81 (0.59 – 1.15) | 0.1546 |
|  | Parietal | 1.09 (0.71 – 1.75) | 0.95 (0.7 – 2.04) | 0.5602 | 1.1 (0.71 – 1.79) | 0.94 (0.7 – 2.09) | 0.5602 |
|  | Temporal | 0.86 (0.52 – 1.44) | 0.85 (0.51 – 1.38) | 0.9478 | 0.85 (0.51 – 1.38) | 0.84 (0.49 – 1.4) | 0.9588 |
|  | Occipital | 0.36 (0.3 – 0.47) | 0.68 (0.47 – 1.22) | **<0.001** | 0.36 (0.31 – 0.48) | 0.68 (0.48 – 1.28) | **<0.001** |
| MAD of HbO (au) | Frontal | 3.65 (2.51 – 6.94) | 4.43 (2.83 – 8.12) | 0.2995 | 3.64 (2.5 – 6.9) | 4.44 (2.82 – 8.05) | 0.3125 |
|  | Parietal | 11.17 (4.39 – 20.58) | 10.26 (6.29 – 16.97) | 0.9259 | 11.16 (4.27 – 20.76) | 10.39 (6.28 – 17.18) | 0.9423 |
|  | Temporal | 8.1 (3.57 – 12.53) | 6.17 (3.69 – 11.69) | 0.5237 | 8.1 (3.6 – 12.65) | 6.14 (3.68 – 11.67) | 0.5327 |
|  | Occipital | 5.59 (3.73 – 9.02) | 10.25 (5.06 – 22.23) | **0.0017** | 5.62 (3.71 – 9.19) | 10.27 (5.11 – 22.2) | **0.0018** |
| MAD of HHb (au) | Frontal | 3.21 (1.48 – 5.35) | 4.4 (2.08 – 8.19) | 0.0566 | 3.25 (1.48 – 5.27) | 4.44 (2.08 – 8.09) | 0.0632 |
|  | Parietal | 11.29 (3.41 – 17.75) | 8.23 (4.21 – 17.56) | 0.8876 | 11.36 (3.35 – 17.83) | 8.22 (4.19 – 17.46) | 0.8713 |
|  | Temporal | 6.18 (2.53 – 11.61) | 4.86 (2.84 – 8.04) | 0.4462 | 6.16 (2.49 – 11.72) | 4.86 (2.91 – 7.89) | 0.4628 |
|  | Occipital | 3.17 (2.12 – 5.46) | 8.15 (3.66 – 13.96) | **<0.001** | 3.25 (2.18 – 5.31) | 8.18 (3.67 – 13.79) | **<0.001** |
| MAD of tHb (au) | Frontal | 6 (3.6 – 10.59) | 6.94 (4.33 – 15.05) | 0.4219 | 6.14 (3.56 – 10.47) | 6.94 (4.25 – 14.7) | 0.4299 |
|  | Parietal | 18.84 (6.43 – 37.36) | 15.67 (7.66 – 27.13) | 0.5015 | 18.76 (6.59 – 37.3) | 15.45 (7.54 – 27.01) | 0.4884 |
|  | Temporal | 11.03 (5.2 – 24.36) | 11.3 (6.8 – 15.07) | 0.812 | 11.16 (5.38 – 24.66) | 11.39 (6.82 – 15.11) | 0.796 |
|  | Occipital | 6.93 (2.84 – 11.69) | 20.39 (8.9 – 35.35) | **<0.001** | 6.83 (2.92 – 11.49) | 20.41 (8.92 – 34.64) | **<0.001** |
| MAD of HbDiff (au) | Frontal | 3.34 (2.26 – 4.82) | 3.68 (2.54 – 5.6) | 0.2211 | 3.31 (2.27 – 4.88) | 3.71 (2.54 – 5.66) | 0.2109 |
|  | Parietal | 7.31 (4.67 – 12.2) | 6.51 (4.43 – 15.54) | 0.9588 | 7.34 (4.7 – 12.26) | 6.46 (4.37 – 15.66) | 0.9698 |
|  | Temporal | 5.42 (2.95 – 10.19) | 5.46 (3.18 – 11.03) | 0.6716 | 5.43 (2.89 – 9.72) | 5.39 (3.2 – 10.99) | 0.7123 |
|  | Occipital | 5.79 (4.81 – 7.55) | 4.5 (3.42 – 8.9) | 0.0817 | 5.77 (4.88 – 7.61) | 4.39 (3.39 – 9) | 0.0714 |
| The p-values in the table are derived using Mann-Whitney U test between the bilateral signals. *ABP, arterial blood pressure; bpm, beats per minute; EtCO_2_, end-tidal carbon dioxide; HbDiff, hemoglobin difference; HbO, oxyhemoglobin; HHb, deoxyhemoglobin; IQR, interquartile range; MAD, median absolute deviation; mmHg, millimeters of mercury; RR, respiratory rate; rSO_2_, regional cerebral oxygen saturation; tHb, total hemoglobin.* | | | | | | | |

Appendix S2c: Median and IQR of Physiologic Signals Raw Data

| **Physiologic Signal** | **Brain Lobe** | **Median (IQR)** | | | | | |
| --- | --- | --- | --- | --- | --- | --- | --- |
|  |  | **1 Hz Sampled Data** | | | **250 Hz Sampled Data** | | |
|  |  | **Left Hemisphere** | **Right Hemisphere** | **p-value** | **Left Hemisphere** | **Right Hemisphere** | **p-value** |
| ABP (mmHg) | – | 94.44 (87.95 – 101.16) | | – | 92.7 (80.49 – 108.37) | | – |
| EtCO_2_ (mmHg) | – | 35.79 (34 – 37) | | – | 35.5 (34 – 37) | | – |
| RR (bpm) | – | 17.83 (15 – 20) | | – | 18 (15 – 20) | | – |
| rSO_2_ (%) | Frontal | 45.29 (44.61 – 46.11) | 40.55 (39.33 – 41.2) | **<0.001** | 45.31 (44.24 – 46.13) | 40.74 (39.31 – 41.65) | **<0.001** |
|  | Parietal | 48.6 (47.22 – 50.54) | 45.53 (44.18 – 47.16) | **0.0018** | 48.39 (46.05 – 51.41) | 45.61 (43.93 – 47.25) | **0.0034** |
|  | Temporal | 40.42 (38.72 – 42.04) | 48.19 (46.78 – 48.92) | **<0.001** | 40.32 (38.1 – 42.98) | 47.99 (46.06 – 48.87) | **<0.001** |
|  | Occipital | 35.98 (35.08 – 36.81) | 46.83 (46.08 – 48.08) | **<0.001** | 36.38 (30.16 – 41.33) | 46.85 (45.61 – 48.39) | **<0.001** |
| HbO (au) | Frontal | -119.85 (-128.57 – -114.15) | -128.46 (-136.45 – -123.29) | 0.0805 | -119.85 (-128.61 – -114.14) | -128.44 (-137.61 – -123.29) | 0.0817 |
|  | Parietal | -172.88 (-192.91 – -155.09) | -183.81 (-208.34 – -170.25) | 0.1586 | -173.03 (-193.07 – -156.58) | -183.51 (-208.23 – -170.26) | 0.1648 |
|  | Temporal | -210.81 (-229.58 – -201.28) | -154.86 (-163.13 – -141.22) | **0.0211** | -210.89 (-229.74 – -197.89) | -154.87 (-162.73 – -139.89) | **0.0223** |
|  | Occipital | -493.88 (-506.53 – -482.29) | -186.75 (-208.19 – -170.66) | **<0.001** | -509.12 (-546.15 – -431.87) | -187.35 (-208.67 – -170.67) | **<0.001** |
| HHb (au) | Frontal | -110.22 (-111.29 – -105.89) | -86.98 (-94.56 – -78.56) | 0.0805 | -110.2 (-111.31 – -105.07) | -86.95 (-94.54 – -78.58) | 0.0793 |
|  | Parietal | -172.14 (-196.13 – -157.48) | -163.23 (-184.06 – -146.51) | 0.6867 | -174.17 (-198.66 – -156.46) | -163.28 (-184.71 – -146.35) | 0.6817 |
|  | Temporal | -143.02 (-159.02 – -133.73) | -149 (-158.38 – -131.74) | 0.4713 | -143.31 (-159.42 – -134.59) | -149.28 (-158.5 – -131.94) | 0.4503 |
|  | Occipital | -269.51 (-279.87 – -261.48) | -164.19 (-182.13 – -154.85) | **<0.001** | -282.8 (-308.95 – -227.39) | -164.57 (-182.56 – -155.31) | **<0.001** |
| tHb (au) | Frontal | -227.73 (-242.17 – -214.31) | -216.95 (-232.11 – -198.91) | 0.7071 | -226.99 (-242.16 – -214.28) | -216.9 (-232.08 – -198.87) | 0.7071 |
|  | Parietal | -339.54 (-381.28 – -311.42) | -346.02 (-385.13 – -321.2) | 0.6075 | -339.97 (-381.95 – -312.12) | -346.01 (-386.11 – -321.38) | 0.5932 |
|  | Temporal | -355.54 (-382.11 – -341.67) | -309.23 (-324.26 – -272.12) | 0.306 | -356.42 (-382.64 – -341.51) | -309.24 (-324.26 – -274.68) | 0.3259 |
|  | Occipital | -761.89 (-792.89 – -749.94) | -353.22 (-391.32 – -333.93) | **<0.001** | -766.83 (-803.17 – -729.66) | -353.58 (-391.35 – -333.83) | **<0.001** |
| HbDiff (au) | Frontal | -19.6 (-21.89 – -13.88) | -44.9 (-49.68 – -37.81) | **<0.001** | -19.66 (-22.76 – -13.85) | -44.83 (-49.96 – -38.3) | **<0.001** |
|  | Parietal | -8.41 (-16.83 – 2.68) | -29.25 (-35.48 – -19.41) | **0.0027** | -10.66 (-22.52 – 10) | -28.84 (-37.46 – -16.84) | **0.0059** |
|  | Temporal | -55.47 (-72.07 – -50.54) | -8.5 (-18.07 – -5.47) | **<0.001** | -60.4 (-73.13 – -48.11) | -13.89 (-24.68 – -5.42) | **<0.001** |
|  | Occipital | -218.53 (-232.31 – -205.37) | -21.5 (-25.55 – -11.82) | **<0.001** | -228.91 (-309.34 – -126.29) | -20.02 (-28.37 – -9.9) | **<0.001** |
| MAD of ABP (mmHg) | – | 6.5 (5.57 – 7.52) | | – | 13.31 (11.9 – 14.34) | | – |
| MAD of EtCO_2_ (mmHg) | – | 1 (1 – 2) | | – | 1 (1 – 2) | | – |
| MAD of RR (bpm) | – | 2 (2 – 3) | | – | 2 (2 – 3) | | – |
| MAD of rSO_2_ (%) | Frontal | 0.8 (0.51 – 0.97) | 0.88 (0.58 – 1.2) | 0.2263 | 0.84 (0.57 – 1.11) | 0.92 (0.61 – 1.39) | 0.2317 |
|  | Parietal | 1.26 (0.87 – 1.83) | 1.04 (0.75 – 1.85) | 0.2837 | 3.17 (1.58 – 5.18) | 1.63 (0.87 – 3.96) | **0.0094** |
|  | Temporal | 0.98 (0.67 – 1.59) | 1 (0.7 – 1.67) | 0.702 | 1.4 (0.76 – 4.87) | 1.34 (0.78 – 5.51) | 0.9478 |
|  | Occipital | 0.88 (0.69 – 1) | 0.79 (0.55 – 1.41) | 0.9588 | 5.52 (4.27 – 6.24) | 1.31 (0.67 – 5.14) | **<0.001** |
| MAD of HbO (au) | Frontal | 4.18 (2.93 – 7.74) | 4.88 (3.19 – 8.32) | 0.414 | 4.2 (2.96 – 7.74) | 5.41 (3.19 – 8.95) | 0.3158 |
|  | Parietal | 12.77 (6.15 – 18.88) | 10.05 (7.18 – 17.04) | 0.5742 | 18.47 (12.73 – 39.77) | 14.63 (8.66 – 34.7) | 0.1566 |
|  | Temporal | 8.99 (4.08 – 17.35) | 8.17 (4.17 – 12.21) | 0.5695 | 11.2 (5.66 – 33.13) | 9.26 (4.18 – 36) | 0.5059 |
|  | Occipital | 10.09 (8.57 – 12.19) | 11.5 (6.04 – 22.26) | 0.5979 | 57.62 (43.68 – 61.02) | 14.22 (7.24 – 34.33) | **<0.001** |
| MAD of HHb (au) | Frontal | 3.39 (1.56 – 6.3) | 4.58 (2.7 – 8.39) | 0.0714 | 3.43 (1.57 – 6.81) | 4.54 (2.74 – 8.64) | 0.0867 |
|  | Parietal | 13.4 (4.81 – 19.33) | 9.03 (6.02 – 15.15) | 0.5979 | 18.89 (10.22 – 31.55) | 14.35 (7.14 – 28.32) | 0.1845 |
|  | Temporal | 6.81 (4.13 – 12.58) | 6.48 (4.64 – 9.18) | 0.5418 | 12.21 (5.78 – 32.06) | 8.7 (4.69 – 38.97) | 0.7226 |
|  | Occipital | 7.25 (6.25 – 8.74) | 9.67 (4.3 – 15.79) | 0.1234 | 40.92 (33.77 – 42.2) | 15 (4.71 – 30.18) | **<0.001** |
| MAD of tHb (au) | Frontal | 6.9 (3.49 – 11.99) | 7.5 (5.15 – 14.73) | 0.2454 | 7.06 (3.51 – 14.17) | 8.35 (5.28 – 15.69) | 0.2868 |
|  | Parietal | 24.14 (7.87 – 40.19) | 15.51 (7.44 – 32.89) | 0.361 | 27.91 (15.7 – 44.35) | 21.84 (11.44 – 35.83) | 0.1822 |
|  | Temporal | 12.57 (5.84 – 21.83) | 11.77 (6.85 – 17.79) | 0.8822 | 22.12 (8.32 – 34.71) | 15.4 (8.5 – 35.76) | 0.8659 |
|  | Occipital | 8.71 (5.62 – 15.43) | 18.57 (8.81 – 36.77) | **<0.001** | 35.09 (27.16 – 38.8) | 26.33 (10.41 – 50.15) | 0.077 |
| MAD of HbDiff (au) | Frontal | 3.59 (2.47 – 5.22) | 4.13 (2.69 – 6.35) | 0.3984 | 3.81 (2.43 – 5.37) | 4.13 (2.69 – 7.24) | 0.3757 |
|  | Parietal | 8.14 (5.49 – 13.44) | 9.16 (4.74 – 17.5) | 0.9862 | 19.55 (7.48 – 55.43) | 11.31 (6.08 – 32.65) | 0.0906 |
|  | Temporal | 6.5 (3.48 – 14.48) | 7.86 (3.41 – 15.11) | 0.6319 | 7.83 (3.74 – 49.83) | 7.45 (3.73 – 59.93) | 0.8713 |
|  | Occipital | 14.48 (11.75 – 15.75) | 6.02 (3.68 – 13.56) | **<0.001** | 87.56 (71.11 – 95.24) | 8.92 (4.1 – 35.34) | **<0.001** |
| The p-values in the table are derived using Mann-Whitney U test between the bilateral signals. *ABP, arterial blood pressure; bpm, beats per minute; EtCO_2_, end-tidal carbon dioxide; HbDiff, hemoglobin difference; HbO, oxyhemoglobin; HHb, deoxyhemoglobin; IQR, interquartile range; MAD, median absolute deviation; mmHg, millimeters of mercury; RR, respiratory rate; rSO_2_, regional cerebral oxygen saturation; tHb, total hemoglobin.* | | | | | | | |

Appendix S2d: Percent Time Results of rSO_2_ and CVR Indices Using 10-Second Decimated Data

| **Physiologic Variable** | **Brain Lobe** | **Median (IQR; MAD)** | | | | | |
| --- | --- | --- | --- | --- | --- | --- | --- |
|  |  | **1 Hz Sampled Data** | | | **250 Hz Sampled Data** | | |
|  |  | **Left Hemisphere** | **Right Hemisphere** | **p-value** | **Left Hemisphere** | **Right Hemisphere** | **p-value** |
| % time rSO_2_ > 30% | Frontal | 100 (100 – 100; 0) | 100 (100 – 100; 0) | **0.0259** | 100 (100 – 100; 0) | 100 (100 – 100; 0) | **0.0072** |
|  | Parietal | 100 (100 – 100; 0) | 100 (100 – 100; 0) | 0.7026 | 100 (100 – 100; 0) | 100 (100 – 100; 0) | 0.7026 |
|  | Temporal | 100 (100 – 100; 0) | 100 (100 – 100; 0) | 0.5836 | 100 (100 – 100; 0) | 100 (100 – 100; 0) | 0.3297 |
|  | Occipital | 100 (100 – 100; 0) | 100 (100 – 100; 0) | 0.5676 | 100 (100 – 100; 0) | 100 (100 – 100; 0) | 0.5676 |
| % time rSO_2_ > 40% | Frontal | 100 (100 – 100; 0) | 69.73 (1.06 – 98.94; 30.27) | **<0.001** | 100 (100 – 100; 0) | 69.83 (0.96 – 98.94; 30.17) | **<0.001** |
|  | Parietal | 100 (74.5 – 100; 0) | 100 (89.16 – 100; 0) | 0.8527 | 100 (74.49 – 100; 0) | 100 (89.4 – 100; 0) | 0.8467 |
|  | Temporal | 58.53 (7.59 – 94.84; 39.48) | 100 (100 – 100; 0) | **<0.001** | 58.54 (7.63 – 94.84; 39.31) | 100 (100 – 100; 0) | **<0.001** |
|  | Occipital | 0 (0 – 0; 0) | 100 (93.41 – 100; 0) | **<0.001** | 0 (0 – 0; 0) | 100 (93.51 – 100; 0) | **<0.001** |
| % time rSO_2_ > 50% | Frontal | 0 (0 – 72.62; 0) | 0 (0 – 0; 0) | **0.0021** | 0 (0 – 73.02; 0) | 0 (0 – 0; 0) | **0.0107** |
|  | Parietal | 37.58 (2.26 – 88.35; 37.49) | 0.14 (0 – 18.78; 0.14) | **<0.001** | 37.33 (2.26 – 88.51; 37.06) | 0.07 (0 – 18.78; 0.07) | **<0.001** |
|  | Temporal | 0 (0 – 0; 0) | 1.19 (0 – 47.54; 1.19) | **<0.001** | 0 (0 – 0; 0) | 1.29 (0 – 47.75; 1.29) | **<0.001** |
|  | Occipital | 0 (0 – 0; 0) | 1.6 (0 – 22.66; 1.6) | **<0.001** | 0 (0 – 0; 0) | 1.69 (0 – 22.64; 1.69) | **<0.001** |
| % time rSO_2_ > 60% | Frontal | 0 (0 – 0; 0) | 0 (0 – 0; 0) | 0.3198 | 0 (0 – 0; 0) | 0 (0 – 0; 0) | 0.9733 |
|  | Parietal | 0 (0 – 0; 0) | 0 (0 – 0; 0) | 0.1448 | 0 (0 – 0; 0) | 0 (0 – 0; 0) | 0.0863 |
|  | Temporal | 0 (0 – 0; 0) | 0 (0 – 0; 0) | 0.0907 | 0 (0 – 0; 0) | 0 (0 – 0; 0) | **0.0487** |
|  | Occipital | 0 (0 – 0; 0) | 0 (0 – 0; 0) | **0.0231** | 0 (0 – 0; 0) | 0 (0 – 0; 0) | **0.0231** |
| % time COx-a > 0 | Frontal | 50.06 (39.99 – 54.35; 6.61) | 49.47 (43.43 – 56.02; 6.55) | 0.3832 | 49.49 (38.66 – 54.93; 7.43) | 49.53 (44.44 – 56.88; 5.72) | 0.3293 |
|  | Parietal | 50.16 (44.06 – 54.66; 5.72) | 46.78 (40.94 – 56.9; 8.46) | 0.4299 | 49.82 (43.59 – 53.8; 6.04) | 48.64 (42.08 – 57.33; 7.98) | 0.6368 |
|  | Temporal | 49.91 (45.73 – 56.33; 5.93) | 49.72 (41.81 – 58.51; 8.2) | 0.9917 | 49.85 (44.88 – 56.8; 5.68) | 51.58 (41.61 – 58.66; 8.97) | 0.9643 |
|  | Occipital | 50.34 (44.13 – 54.82; 6.06) | 50.85 (44.88 – 58.28; 7.21) | 0.3647 | 50.93 (43.74 – 57.14; 6.95) | 49.29 (44.16 – 59.03; 7.38) | 0.9149 |
| % time COx-a > 0.2 | Frontal | 23.08 (16.12 – 29.38; 6.9) | 25.53 (21 – 33.6; 5.97) | 0.0939 | 22.74 (16.47 – 29.65; 6.69) | 26.35 (21.17 – 34.81; 6.46) | 0.0753 |
|  | Parietal | 24.54 (17.76 – 28.38; 4.33) | 24.09 (17.81 – 31.77; 7.59) | 0.5372 | 24.5 (17.67 – 28.01; 4.92) | 24.15 (17.73 – 31.91; 7.22) | 0.5602 |
|  | Temporal | 23.18 (17.97 – 30.58; 5.71) | 25.01 (16.48 – 32.45; 8.42) | 0.8335 | 23.78 (18.11 – 29.39; 5.71) | 25.09 (16.72 – 31.94; 7.32) | 0.6691 |
|  | Occipital | 17.24 (12.85 – 22.45; 5.02) | 24.58 (19.66 – 32.41; 6.05) | **<0.001** | 17.76 (13.45 – 24.15; 5.67) | 23.75 (18.54 – 32.54; 6.26) | **<0.001** |
| % time COx-a > 0.3 | Frontal | 14.23 (8.39 – 20; 6.07) | 16.12 (11.78 – 23.19; 5.11) | 0.0731 | 13.09 (8.38 – 18.08; 4.85) | 16.47 (11.7 – 22.98; 4.88) | 0.0747 |
|  | Parietal | 14.94 (9.34 – 18.41; 4.62) | 15.12 (9.87 – 20.87; 5.76) | 0.9012 | 14.78 (9.45 – 19.05; 4.42) | 14.77 (9.24 – 21.28; 5.91) | 0.9423 |
|  | Temporal | 12.84 (9.01 – 18.93; 4.99) | 15.29 (8.84 – 20.26; 5.91) | 0.6417 | 13.38 (8.65 – 18.26; 4.81) | 15.28 (9.61 – 19.73; 5.27) | 0.4819 |
|  | Occipital | 8.09 (4.13 – 12.08; 4.12) | 16.21 (11.37 – 21.44; 5.12) | **<0.001** | 7.86 (5.22 – 11.71; 3.31) | 14.78 (11.17 – 23.36; 5.71) | **<0.001** |
| % time HbOx > 0 | Frontal | 47.7 (40.58 – 56.19; 7.82) | 51.08 (45.69 – 58.75; 6.87) | 0.0515 | 48.66 (40.78 – 55.11; 7.16) | 50.96 (46.57 – 58.14; 6.36) | **0.0483** |
|  | Parietal | 51.56 (44.43 – 55.29; 6.29) | 51.98 (44.63 – 59.3; 7.44) | 0.6172 | 50.85 (44.15 – 55.58; 6.14) | 52.28 (45.18 – 59.39; 7.16) | 0.5884 |
|  | Temporal | 51.07 (43.41 – 58.42; 7.63) | 52.13 (41.69 – 59.66; 9.02) | 0.6969 | 51.31 (42.52 – 56.87; 7.09) | 53.29 (41.6 – 59.37; 7.76) | 0.5214 |
|  | Occipital | 53.05 (45.84 – 57.56; 6.31) | 49 (43.67 – 57.58; 7.43) | 0.6417 | 53.61 (46.29 – 57.16; 5.53) | 51.03 (44.69 – 57.34; 6.35) | 0.5214 |
| % time HbOx > 0.2 | Frontal | 22.29 (17.9 – 28.49; 5.04) | 27.63 (22.53 – 32.14; 5.07) | **0.0042** | 22.96 (17.56 – 27.16; 4.83) | 27.15 (22.33 – 31.89; 4.88) | **0.0056** |
|  | Parietal | 27.17 (19.82 – 32.91; 6.47) | 27.03 (22.04 – 35.26; 5.84) | 0.4734 | 27.42 (19.74 – 32.7; 7) | 26.33 (21.8 – 35.02; 6.9) | 0.3397 |
|  | Temporal | 23.42 (18.26 – 29.94; 5.9) | 26.47 (18.84 – 33.27; 7.51) | 0.3485 | 22.98 (18.21 – 29.89; 5.22) | 26.08 (19.26 – 32.15; 6.6) | 0.2931 |
|  | Occipital | 21.53 (15.11 – 28.65; 6.95) | 25.57 (19.77 – 31.44; 6) | **0.0323** | 21.66 (17.13 – 28.74; 6.27) | 25.41 (20.57 – 30.53; 5.08) | 0.0731 |
| % time HbOx > 0.3 | Frontal | 13.69 (8.67 – 17.2; 3.66) | 16.88 (13.03 – 23.18; 4.24) | **0.0049** | 13.42 (9.19 – 16.81; 3.96) | 16.92 (13.15 – 23.82; 4.81) | **0.0038** |
|  | Parietal | 17.12 (11.25 – 22.59; 5.81) | 17.8 (13.91 – 22.73; 4.18) | 0.5015 | 16.78 (11.21 – 21.92; 5.56) | 17.56 (13.78 – 21.77; 4.16) | 0.4841 |
|  | Temporal | 14.54 (9.57 – 18.41; 4.26) | 15.25 (9.8 – 22.06; 5.96) | 0.6491 | 14.47 (9.91 – 17.86; 4.39) | 16.5 (9.93 – 21.89; 6.07) | 0.5015 |
|  | Occipital | 11.1 (7.37 – 17.17; 4.87) | 17.35 (11.86 – 20.69; 4.94) | **0.0038** | 12.62 (7.02 – 18.18; 5.64) | 17.01 (11.66 – 21.02; 4.79) | **0.0105** |
| % time HHbx > 0 | Frontal | 49.59 (44.51 – 58.55; 6.82) | 51.62 (42.99 – 56.36; 7.79) | 0.8686 | 49.88 (44.59 – 57.73; 5.64) | 52.11 (42.63 – 56.9; 7.3) | 0.8415 |
|  | Parietal | 49.66 (44.04 – 55.4; 5.83) | 51.46 (46.7 – 58.48; 6.19) | 0.1856 | 49.14 (43.47 – 55.2; 5.73) | 52.69 (45.94 – 57.85; 6.63) | 0.1506 |
|  | Temporal | 49.16 (42.06 – 55.99; 7.2) | 50.84 (45.03 – 57.99; 6.69) | 0.2611 | 50.14 (42.63 – 56.41; 7.08) | 50.79 (45.02 – 57.84; 6) | 0.4884 |
|  | Occipital | 52.6 (45.8 – 58.36; 6.51) | 51.92 (45.96 – 55.13; 5.2) | 0.2597 | 53.19 (47.44 – 57.83; 5.69) | 51.72 (45.73 – 57.02; 5.98) | 0.4042 |
| % time HHbx > 0.2 | Frontal | 24.23 (20.48 – 31.81; 5.57) | 26.93 (22.47 – 33.27; 4.93) | 0.3027 | 25.58 (20.16 – 31.33; 5.66) | 27.38 (22.6 – 32.83; 5.07) | 0.2776 |
|  | Parietal | 25.04 (18.86 – 29.02; 5.77) | 26.7 (21.85 – 32.86; 5.73) | 0.0919 | 25.24 (18.5 – 29.88; 6.21) | 26.62 (21.89 – 32.91; 5.84) | 0.1506 |
|  | Temporal | 24.16 (18.16 – 30.28; 6.18) | 26.4 (18.95 – 32.54; 6.67) | 0.4884 | 24.52 (18.18 – 30.2; 6.07) | 26.27 (19.21 – 32.23; 6.61) | 0.551 |
|  | Occipital | 20.93 (14.89 – 26.79; 6.12) | 25.64 (20.15 – 32.53; 6.13) | **0.008** | 20.03 (14.47 – 26.09; 6.02) | 26.1 (19.99 – 32.18; 6.24) | **0.0025** |
| % time HHbx > 0.3 | Frontal | 14.89 (11.61 – 21.48; 4.19) | 18.03 (13.63 – 21.96; 4.39) | 0.1811 | 15.25 (11.99 – 21.93; 4.2) | 17.97 (14.22 – 22.98; 4.29) | 0.1712 |
|  | Parietal | 16.36 (10.52 – 20.24; 5.48) | 16.84 (13.38 – 22.98; 4.75) | 0.1467 | 16.36 (8.84 – 19.66; 6.6) | 17.91 (13.23 – 24.67; 5.09) | 0.0946 |
|  | Temporal | 14.84 (9.33 – 19.68; 5.09) | 16.98 (9.02 – 23.69; 7.38) | 0.3242 | 15.08 (9.35 – 20.31; 5.61) | 16.74 (9.68 – 23.72; 7.22) | 0.2995 |
|  | Occipital | 10.02 (5.21 – 14.98; 5) | 15.39 (10.25 – 22.1; 5.66) | **<0.001** | 9.85 (5.39 – 13.54; 4.46) | 15.98 (11.51 – 21.88; 5.83) | **<0.001** |
| % time tHbx > 0 | Frontal | 49.26 (43.61 – 56.57; 6.52) | 50.63 (44.51 – 59.13; 7.59) | 0.3538 | 48.54 (44.2 – 57.35; 7.14) | 51.08 (44.22 – 58.9; 7.74) | 0.3869 |
|  | Parietal | 49.75 (43.77 – 53.76; 5.32) | 51.72 (45.67 – 60.64; 7.15) | 0.2071 | 49.6 (45.14 – 53.74; 4.39) | 51.79 (46.41 – 59.22; 6.48) | 0.2303 |
|  | Temporal | 50.32 (43.74 – 58.78; 7.73) | 51.45 (44.6 – 57.3; 5.88) | 0.8415 | 50.76 (44.52 – 58.07; 6.79) | 52.56 (44.31 – 56.47; 6.26) | 0.9396 |
|  | Occipital | 51.04 (47.41 – 57.41; 5.52) | 50.82 (45.09 – 59.53; 6.31) | 0.812 | 51.31 (46.57 – 56.93; 5.48) | 51.39 (44.66 – 58.67; 7.25) | 0.8442 |
| % time tHbx > 0.2 | Frontal | 24.26 (18.33 – 31.74; 6.93) | 26.89 (21.23 – 34.61; 6.88) | 0.1506 | 23.98 (18.75 – 31.98; 6) | 27.56 (20.04 – 33.84; 7.03) | 0.1891 |
|  | Parietal | 25.2 (20.4 – 33.71; 7.81) | 28.57 (23.24 – 33.6; 5.39) | 0.3984 | 25.9 (19.45 – 33.61; 7.6) | 29.13 (23.2 – 33.75; 5.25) | 0.4462 |
|  | Temporal | 25.15 (20.18 – 32.12; 5.42) | 26.13 (19.86 – 31.8; 5.9) | 0.8335 | 25.08 (19.69 – 30.54; 5.5) | 26.59 (19.71 – 31.52; 6.05) | 0.8254 |
|  | Occipital | 22.77 (18.78 – 29.19; 5.1) | 26.84 (20.08 – 31.84; 5.58) | 0.1268 | 23.17 (19.24 – 29.86; 5.8) | 26.58 (19.44 – 32.63; 6.62) | 0.1669 |
| % time tHbx > 0.3 | Frontal | 14.6 (8.84 – 20.37; 5.85) | 16.82 (13.69 – 23.3; 5.79) | 0.0531 | 13.88 (9.37 – 20.51; 5.35) | 17.36 (13.12 – 23.45; 5.7) | **0.037** |
|  | Parietal | 17.34 (11.99 – 23.56; 6.06) | 18.43 (12.69 – 24.64; 6.11) | 0.5259 | 17.65 (11.26 – 23.48; 6.29) | 18.86 (12.72 – 24.01; 6.02) | 0.4755 |
|  | Temporal | 15.9 (11.95 – 20.95; 4.48) | 18.54 (10.11 – 21.25; 6.39) | 0.6817 | 14.98 (11.87 – 21; 3.87) | 18.26 (9.66 – 21.82; 6.97) | 0.6442 |
|  | Occipital | 14.6 (10.54 – 19.58; 4.95) | 17.32 (12.48 – 21.11; 4.59) | 0.0782 | 14.1 (10.61 – 19.4; 4.02) | 17.3 (12.82 – 22.11; 4.74) | 0.0867 |
| % time HbDiffx > 0 | Frontal | 50.31 (41.54 – 56.07; 6.83) | 51.71 (45.3 – 59.01; 7.3) | 0.1566 | 49.07 (40.42 – 55.94; 7.63) | 51.52 (45.47 – 57.44; 6.1) | 0.1938 |
|  | Parietal | 50.42 (43.99 – 54.53; 4.91) | 48.53 (40.71 – 56.75; 8.12) | 0.4971 | 50.28 (43.72 – 55.8; 5.86) | 48.89 (43.28 – 57.05; 7.84) | 0.6124 |
|  | Temporal | 50.84 (45.59 – 58.33; 7.09) | 51.04 (42.89 – 58.17; 7.52) | 0.7226 | 51.17 (45.41 – 57.72; 6.51) | 52.14 (42.13 – 57.37; 8.88) | 0.7801 |
|  | Occipital | 49.86 (45.99 – 57.45; 5.14) | 50.29 (44.75 – 60.48; 7.94) | 0.7907 | 51.76 (46.27 – 56.86; 5.25) | 49.48 (44.69 – 59.9; 8.05) | 0.6842 |
| % time HbDiffx > 0.2 | Frontal | 21.63 (16.97 – 30.79; 6.05) | 27.42 (21.51 – 32.59; 5.88) | **0.0211** | 22.9 (16.62 – 30.2; 6.42) | 28.58 (22.09 – 32.41; 5.32) | **0.0189** |
|  | Parietal | 24.44 (18.05 – 28.39; 5.65) | 23.88 (17.44 – 31.63; 6.95) | 0.5649 | 24.66 (17.91 – 28.59; 6.54) | 24.56 (17.76 – 31.21; 6.77) | 0.5395 |
|  | Temporal | 22.99 (18.43 – 28.77; 5) | 25.13 (16.88 – 31.75; 7.8) | 0.6343 | 23.68 (17.64 – 28.89; 5.41) | 25.04 (16.97 – 31.8; 6.85) | 0.4841 |
|  | Occipital | 19.67 (13.67 – 25.69; 6.17) | 25.41 (18.17 – 34.42; 7.34) | **0.0027** | 19.94 (14.35 – 26.59; 6.19) | 24.39 (17.98 – 35.1; 6.72) | **0.0109** |
| % time HbDiffx > 0.3 | Frontal | 14.29 (9.07 – 20.34; 5.44) | 17.88 (14.01 – 22.25; 4.26) | **0.0318** | 13.11 (8.69 – 18.03; 4.52) | 17.93 (14.2 – 21.69; 3.78) | **0.0155** |
|  | Parietal | 16.18 (11.08 – 18.5; 3.99) | 14.58 (9.62 – 20; 5.01) | 0.8659 | 16.27 (10.67 – 18.81; 5.03) | 14.97 (9.97 – 20.75; 5.33) | 0.9396 |
|  | Temporal | 13.41 (8.68 – 18.8; 5.21) | 15.7 (8.43 – 20.55; 6.44) | 0.6196 | 12.61 (8.27 – 17.89; 4.57) | 16.08 (9.29 – 20.33; 5.83) | 0.438 |
|  | Occipital | 9.4 (5.28 – 14.24; 4.32) | 16.43 (10.43 – 24.27; 6.25) | **<0.001** | 9.82 (5.57 – 14.55; 4.7) | 16.08 (9.77 – 24.62; 6.73) | **<0.001** |
| The p-values in the table are derived using Mann-Whitney U test between the bilateral signals. *COx-a, cerebral oximetry index with arterial blood pressure; CVR, cerebrovascular reactivity index; HbDiffx, hemoglobin difference index; HbOx, oxyhemoglobin index; HHbx, deoxyhemoglobin index; IQR, interquartile range; MAD, median absolute deviation; rSO_2_, regional cerebral oxygen saturation; tHbx, total hemoglobin index.* | | | | | | | |

Appendix S2e: Percent Time Results of rSO_2_ Using Raw Data

| **Physiologic Variable** | **Brain Lobe** | **Median (IQR; MAD)** | | | | | |
| --- | --- | --- | --- | --- | --- | --- | --- |
|  |  | **1 Hz Sampled Data** | | | **250 Hz Sampled Data** | | |
|  |  | **Left Hemisphere** | **Right Hemisphere** | **p-value** | **Left Hemisphere** | **Right Hemisphere** | **p-value** |
| % time rSO_2_ > 30% | Frontal | 100 (100 – 100; 0) | 100 (100 – 100; 0) | **0.0291** | 100 (100 – 100; 0) | 100 (99.82 – 100; 0) | **0.0316** |
|  | Parietal | 100 (99.98 – 100; 0) | 100 (100 – 100; 0) | 0.1297 | 99.96 (93.12 – 100; 0.04) | 100 (99.13 – 100; 0) | 0.0824 |
|  | Temporal | 100 (100 – 100; 0) | 100 (100 – 100; 0) | 0.2801 | 99.96 (93.13 – 100; 0.04) | 100 (98.83 – 100; 0) | 0.2927 |
|  | Occipital | 99.96 (99.87 – 100; 0.04) | 100 (100 – 100; 0) | **<0.001** | 75.29 (73.96 – 76.75; 1.44) | 99.99 (97.36 – 100; 0.01) | **<0.001** |
| % time rSO_2_ > 40% | Frontal | 100 (100 – 100; 0) | 65.08 (2.27 – 98.66; 34.92) | **<0.001** | 100 (99.8 – 100; 0) | 65.16 (2.37 – 98.12; 34.77) | **<0.001** |
|  | Parietal | 100 (73.47 – 100; 0) | 99.92 (85.97 – 100; 0.08) | 0.7753 | 95.86 (72.13 – 99.92; 4.13) | 98.81 (84.6 – 99.99; 1.19) | 0.3678 |
|  | Temporal | 57.33 (7.78 – 94.73; 39.63) | 100 (99.3 – 100; 0) | **<0.001** | 53.42 (34.45 – 89.4; 25.54) | 99.96 (92.79 – 100; 0.04) | **<0.001** |
|  | Occipital | 0.45 (0.02 – 2.2; 0.45) | 100 (93.17 – 100; 0) | **<0.001** | 29.09 (22.19 – 33.8; 5.91) | 98.2 (82.78 – 100; 1.8) | **<0.001** |
| % time rSO_2_ > 50% | Frontal | 0 (0 – 70.73; 0) | 0 (0 – 0.01; 0) | **0.0062** | 0.27 (0 – 65.93; 0.27) | 0 (0 – 0.24; 0) | **0.0083** |
|  | Parietal | 37.2 (3.81 – 87.56; 36.22) | 0.55 (0 – 19.39; 0.55) | **<0.001** | 36.88 (12.95 – 69.98; 29.67) | 6.85 (0.89 – 24.09; 6.85) | **<0.001** |
|  | Temporal | 0 (0 – 0.02; 0) | 2.17 (0 – 44.43; 2.17) | **<0.001** | 0.27 (0 – 12.57; 0.27) | 16.32 (1.15 – 41.81; 16.3) | **<0.001** |
|  | Occipital | 0 (0 – 0; 0) | 1.77 (0 – 22.98; 1.77) | **<0.001** | 7.29 (2.76 – 10.55; 4.42) | 15.52 (0.29 – 28.08; 14.77) | 0.1075 |
| % time rSO_2_ > 60% | Frontal | 0 (0 – 0; 0) | 0 (0 – 0; 0) | 0.8089 | 0 (0 – 0; 0) | 0 (0 – 0.03; 0) | 0.6233 |
|  | Parietal | 0 (0 – 0.12; 0) | 0 (0 – 0; 0) | **0.0012** | 1.35 (0.03 – 10.32; 1.35) | 0.03 (0 – 0.64; 0.03) | **<0.001** |
|  | Temporal | 0 (0 – 0; 0) | 0 (0 – 0; 0) | 0.0744 | 0 (0 – 1.04; 0) | 0.05 (0 – 2.74; 0.05) | 0.2068 |
|  | Occipital | 0 (0 – 0; 0) | 0 (0 – 0; 0) | **0.0018** | 0.05 (0 – 0.78; 0.05) | 0.31 (0 – 3.1; 0.31) | 0.3559 |
| The p-values in the table are derived using Mann-Whitney U test between the bilateral signals. *IQR, interquartile range; MAD, median absolute deviation; rSO_2_, regional cerebral oxygen saturation.* | | | | | | | |
