# Supplementary material for: Detection of regional disparity in cerebrovascular reactivity using a custom whole brain functional near-infrared spectroscopy based mapping system: A prospective observational study
Source: PLOS Digit Health. 2026 Apr 15;5(4):e0001349. doi: 10.1371/journal.pdig.0001349 (PMC13082728; doi:10.1371/journal.pdig.0001349)
Supplement: S3 Appendix — (DOCX) [file pdig.0001349.s003.docx]

**Appendix S3 – Regional Hemispheric Disparity Analysis**

Appendix S3 – Table of Contents

[Appendix S3a: Regional Hemispheric Disparity Analysis on CVR Indices Using 10-Second Decimated Data at 250 Hz 2](#_Toc213065866)

[Appendix S3b: Regional Hemispheric Disparity Analysis on Physiologic Signals 3](#_Toc213065867)

Appendix S3a: Regional Hemispheric Disparity Analysis on CVR Indices Using 10-Second Decimated Data at 250 Hz

| **Physiologic Variable** | **Median (IQR)** | | | |
| --- | --- | --- | --- | --- |
|  | **Frontal Lobe** | **Parietal Lobe** | **Temporal Lobe** | **Occipital Lobe** |
| ARHD of COx-a (au) | 0.23 (0.11 – 0.41) | 0.22 (0.1 – 0.45) | 0.22 (0.1 – 0.4) | 0.24 (0.11 – 0.41) |
| ARHD of HbOx (au) | 0.22 (0.09 – 0.41) | 0.21 (0.09 – 0.42) | 0.23 (0.11 – 0.43) | 0.24 (0.11 – 0.41) |
| ARHD of HHbx (au) | 0.22 (0.09 – 0.42) | 0.23 (0.1 – 0.44) | 0.22 (0.1 – 0.45) | 0.24 (0.11 – 0.42) |
| ARHD of tHbx (au) | 0.23 (0.1 – 0.42) | 0.22 (0.1 – 0.44) | 0.23 (0.1 – 0.43) | 0.22 (0.1 – 0.39) |
| ARHD of HbDiffx (au) | 0.23 (0.11 – 0.4) | 0.22 (0.1 – 0.42) | 0.23 (0.1 – 0.39) | 0.23 (0.11 – 0.4) |
| MAD of ARHD COx-a (au) | 0.14 (0.12 – 0.17) | 0.15 (0.13 – 0.16) | 0.13 (0.12 – 0.15) | 0.14 (0.12 – 0.16) |
| MAD of ARHD HbOx (au) | 0.14 (0.12 – 0.17) | 0.14 (0.12 – 0.17) | 0.15 (0.13 – 0.16) | 0.14 (0.12 – 0.17) |
| MAD of ARHD HHbx (au) | 0.14 (0.12 – 0.18) | 0.15 (0.13 – 0.17) | 0.14 (0.12 – 0.16) | 0.14 (0.12 – 0.16) |
| MAD of ARHD tHbx (au) | 0.15 (0.12 – 0.16) | 0.15 (0.12 – 0.17) | 0.15 (0.13 – 0.17) | 0.14 (0.12 – 0.17) |
| MAD of ARHD HbDiffx (au) | 0.14 (0.12 – 0.15) | 0.14 (0.12 – 0.17) | 0.14 (0.12 – 0.16) | 0.14 (0.12 – 0.15) |
| The table shows the absolute regional hemispheric disparity analysis in on four brain lobes of the five fNIRS-derived CVR indices using 10-second decimated data at 250 Hz sampling frequency. *ARHD, absolute regional hemispheric difference; au, arbitrary units; COx-a, cerebral oximetry index with arterial blood pressure; CVR, cerebrovascular reactivity index; fNIRS, function near-infrared spectroscopy; HbDiffx, hemoglobin difference index; HbOx, oxyhemoglobin index; HHbx, deoxyhemoglobin index; IQR, interquartile range; MAD, median absolute deviation; tHbx, total hemoglobin index.* | | | | |

Appendix S3b: Regional Hemispheric Disparity Analysis on Physiologic Signals

| **Physiologic Variable** | **Median (IQR)** | | | |
| --- | --- | --- | --- | --- |
|  | **Frontal Lobe** | **Parietal Lobe** | **Temporal Lobe** | **Occipital Lobe** |
| **Raw 1 Hz Sampled Data** | | | | |
| ARHD of rSO_2_ (%) | 7.07 (5.9 – 8.99) | 5.05 (3.08 – 7.89) | 8.1 (6.28 – 9.76) | 10.58 (9.56 – 12.9) |
| ARHD of HbO (au) | 24.76 (16.36 – 33.06) | 46.4 (30.94 – 73.22) | 62.29 (43.23 – 75.94) | 316.63 (297.4 – 333.63) |
| ARHD of HHb (au) | 28.07 (22.92 – 36.44) | 28.15 (14.66 – 45.39) | 32.58 (19.07 – 49.9) | 111.6 (90.69 – 128.49) |
| ARHD of tHb (au) | 37.81 (25.64 – 46.78) | 73.06 (45.39 – 116) | 92.59 (52.63 – 122.14) | 430.74 (391.63 – 459.79) |
| ARHD of HbDiff (au) | 28.63 (23.45 – 37.2) | 29.67 (20.26 – 62.27) | 55.32 (40.71 – 66.52) | 198.52 (183.6 – 218.31) |
| MAD of ARHD rSO_2_ (%) | 1.18 (0.8 – 1.59) | 1.7 (1.13 – 2.56) | 1.37 (0.99 – 2.12) | 1.12 (0.97 – 1.74) |
| MAD of ARHD HbO (au) | 7.24 (4.66 – 11.79) | 15.46 (8.55 – 31.15) | 12.17 (8.78 – 24.32) | 15.89 (12.37 – 25.84) |
| MAD of ARHD HHb (au) | 6.41 (3.86 – 11.86) | 12.6 (6.51 – 19.2) | 8.9 (6.75 – 16.73) | 11.68 (8.46 – 16.38) |
| MAD of ARHD tHb (au) | 11.29 (7.36 – 21.04) | 24.06 (12.07 – 48.42) | 18.57 (9.87 – 33.95) | 23.29 (13.66 – 34.1) |
| MAD of ARHD HbDiff (au) | 5.14 (3.53 – 8.54) | 12.74 (7.24 – 25.64) | 14.26 (5.95 – 21.86) | 15.66 (13.71 – 19.92) |
| **Raw 250 Hz Sampled Data** | | | | |
| ARHD of rSO_2_ (%) | 7.42 (5.85 – 9.29) | 6.85 (3.53 – 11.16) | 9.07 (5.01 – 13.39) | 11.15 (6.72 – 17.55) |
| ARHD of HbO (au) | 25.33 (17.09 – 36.53) | 52.06 (30.89 – 86.13) | 82.79 (44.68 – 114.77) | 334.58 (250.39 – 373.04) |
| ARHD of HHb (au) | 30.68 (22.75 – 37.74) | 42.13 (20.97 – 71.84) | 47.6 (28.01 – 83.03) | 117.98 (63.72 – 153.43) |
| ARHD of tHb (au) | 39.64 (25.99 – 47.58) | 75.76 (40.29 – 120.39) | 93.63 (52.68 – 124.67) | 426.44 (369.4 – 475.73) |
| ARHD of HbDiff (au) | 28.35 (24.27 – 41) | 46.9 (26.04 – 80.97) | 66.28 (46.52 – 90.24) | 207.05 (113.3 – 290.51) |
| MAD of ARHD rSO_2_ (%) | 1.22 (0.84 – 1.9) | 3.39 (1.63 – 4.61) | 3.71 (1.22 – 5.05) | 5.1 (4.32 – 6.17) |
| MAD of ARHD HbO (au) | 7.34 (4.7 – 13.65) | 24.2 (10.99 – 45.42) | 26.96 (10.06 – 53.36) | 56.54 (46.2 – 66.66) |
| MAD of ARHD HHb (au) | 6.42 (4 – 14.19) | 21.44 (10.81 – 34.29) | 22.98 (7.77 – 38.77) | 38.35 (34.87 – 42.86) |
| MAD of ARHD tHb (au) | 12.36 (7.49 – 25.45) | 27.04 (18.36 – 52.55) | 31.08 (16.47 – 41.69) | 43.48 (33.47 – 57.01) |
| MAD of ARHD HbDiff (au) | 5.2 (3.56 – 8.92) | 22.27 (9.34 – 60.99) | 27.13 (7.15 – 67.33) | 84.66 (71.58 – 92.12) |
| **10-Second Decimated 1 Hz Sampled Data** | | | | |
| ARHD of rSO_2_ (%) | 7.11 (5.86 – 8.9) | 5.22 (2.86 – 7.47) | 8 (6.21 – 9.42) | 10.37 (9.95 – 12.89) |
| ARHD of HbO (au) | 24.94 (16.56 – 32.02) | 46.15 (29.55 – 74.18) | 63.9 (43.45 – 76.43) | 314.96 (298.24 – 330.47) |
| ARHD of HHb (au) | 28.13 (22.86 – 36.7) | 26.83 (14.17 – 45.52) | 31.57 (19.24 – 47.01) | 108.54 (90.88 – 128.3) |
| ARHD of tHb (au) | 36.25 (26.86 – 46.72) | 73.71 (42.87 – 116.79) | 100.22 (52.51 – 124.46) | 422.37 (395.21 – 460.37) |
| ARHD of HbDiff (au) | 28.11 (22.9 – 38.06) | 29.74 (21.14 – 48.13) | 53.55 (41.12 – 65.06) | 197.46 (189.17 – 213.21) |
| MAD of ARHD rSO_2_ (%) | 1.08 (0.73 – 1.51) | 1.43 (1.07 – 2.21) | 1.25 (0.9 – 1.74) | 0.78 (0.61 – 1.42) |
| MAD of ARHD HbO (au) | 6.11 (4.02 – 10.99) | 14.85 (7.83 – 32.3) | 10.9 (6.07 – 24.74) | 14.15 (7.64 – 23.66) |
| MAD of ARHD HHb (au) | 5.45 (3.88 – 11.13) | 11.65 (5.7 – 15.38) | 8.42 (5.14 – 17.37) | 10.21 (5.44 – 13.71) |
| MAD of ARHD tHb (au) | 11.36 (6.24 – 19.05) | 25.67 (12.12 – 46.88) | 17.48 (9.61 – 36.39) | 21.29 (13.17 – 31.97) |
| MAD of ARHD HbDiff (au) | 4.86 (3.23 – 7.5) | 10.47 (6.39 – 23.31) | 9.21 (6.08 – 18.68) | 8.62 (6.67 – 13.18) |
| **10-Second Decimated 250 Hz Sampled Data** | | | | |
| ARHD of rSO_2_ (%) | 7.1 (5.86 – 8.78) | 5.24 (2.82 – 7.31) | 7.94 (6.18 – 9.32) | 10.13 (9.67 – 12.75) |
| ARHD of HbO (au) | 24.93 (16.52 – 32.15) | 45.93 (29.77 – 74.15) | 63.98 (43.45 – 76.5) | 314.92 (298.59 – 330.41) |
| ARHD of HHb (au) | 28.07 (22.85 – 36.73) | 26.82 (13.75 – 45.57) | 31.61 (18.9 – 47.04) | 108.82 (90.63 – 128.41) |
| ARHD of tHb (au) | 36.21 (26.79 – 46.76) | 74.11 (43.31 – 117.22) | 100.42 (52.52 – 124.54) | 421.67 (394.95 – 460.77) |
| ARHD of HbDiff (au) | 28.03 (22.96 – 37.96) | 29.81 (20.84 – 48.74) | 53.51 (41.63 – 65.14) | 198.01 (189.04 – 212.46) |
| MAD of ARHD rSO_2_ (%) | 1.08 (0.74 – 1.52) | 1.45 (1.05 – 2.31) | 1.23 (0.9 – 1.69) | 0.81 (0.62 – 1.41) |
| MAD of ARHD HbO (au) | 6.11 (4.23 – 10.97) | 14.74 (7.7 – 32.06) | 11 (5.98 – 24.42) | 14.35 (7.44 – 24) |
| MAD of ARHD HHb (au) | 5.57 (3.84 – 11.13) | 11.54 (5.66 – 15.51) | 8.37 (5.11 – 17.41) | 10.21 (5.24 – 13.56) |
| MAD of ARHD tHb (au) | 11.04 (6.35 – 18.96) | 25.97 (11.99 – 47.4) | 17.47 (9.64 – 36.57) | 21.23 (13.25 – 31.59) |
| MAD of ARHD HbDiff (au) | 4.93 (3.26 – 7.51) | 10.4 (6.47 – 22.67) | 9.12 (6.13 – 18.8) | 8.69 (6.42 – 13.17) |
| The table shows the absolute regional hemispheric disparity analysis in on four brain lobes of the five fNIRS signals using both raw and 10-second decimated data at 1 Hz and 250 Hz sampling frequencies. *ARHD, absolute regional hemispheric difference; au, arbitrary units; fNIRS, function near-infrared spectroscopy; HbDiff, hemoglobin difference; HbO, oxyhemoglobin; HHb, deoxyhemoglobin; IQR, interquartile range; MAD, median absolute deviation; rSO_2_, regional cerebral oxygen saturation; tHb, total hemoglobin.* | | | | |
