# Supplementary material for: Detection of regional disparity in cerebrovascular reactivity using a custom whole brain functional near-infrared spectroscopy based mapping system: A prospective observational study
Source: PLOS Digit Health. 2026 Apr 15;5(4):e0001349. doi: 10.1371/journal.pdig.0001349 (PMC13082728; doi:10.1371/journal.pdig.0001349)
Supplement: S4 Appendix — (DOCX) [file pdig.0001349.s004.docx]

**Appendix S4 – Autoregressive Integrative Moving Average (ARIMA) Analysis**

Appendix S4 – Table of Contents

[Appendix S4a: ADF and KPSS Results Showing Stationary vs Non-Stationary vs NA for fNIRS Physiologic Signals at 1 Hz 2](#_Toc213066046)

[Appendix S4b: ADF and KPSS Results Showing Stationary vs Non-Stationary vs NA for Calculated fNIRS CA Indices at 250 Hz 3](#_Toc213066047)

[Appendix S4c: ADF and KPSS Results Showing Stationary vs Non-Stationary vs NA for fNIRS Physiologic Signals at 250 Hz 4](#_Toc213066048)

[Appendix S4d: Optimal ARIMA Models Based on AIC of CVR Indices and their Hemispheric Disparity at 250 Hz 5](#_Toc213066049)

[Appendix S4e: Optimal ARIMA Models Based on AIC of Physiologic Signals and their Hemispheric Disparity 6](#_Toc213066050)

Appendix S4a: ADF and KPSS Results Showing Stationary vs Non-Stationary vs NA for fNIRS Physiologic Signals at 1 Hz

| **ADF results for non-differenced data** | | | | | | | | | | | | | | | | |
| --- | --- | --- | --- | --- | --- | --- | --- | --- | --- | --- | --- | --- | --- | --- | --- | --- |
| **Hemisphere** | **Brain Lobe** | **HbO** | | | **HHb** | | | **tHb** | | | **HbDiff** | | | **rSO_2_** | | |
|  |  | **S** | **NS** | **NA** | **S** | **NS** | **NA** | **S** | **NS** | **NA** | **S** | **NS** | **NA** | **S** | **NS** | **NA** |
| Left | Frontal | 34 | 16 | 0 | 30 | 20 | 0 | 33 | 17 | 0 | 35 | 15 | 0 | 34 | 16 | 0 |
|  | Parietal | 31 | 19 | 0 | 30 | 20 | 0 | 29 | 21 | 0 | 33 | 17 | 0 | 35 | 15 | 0 |
|  | Temporal | 33 | 17 | 0 | 26 | 24 | 0 | 32 | 18 | 0 | 40 | 10 | 0 | 35 | 15 | 0 |
|  | Occipital | 44 | 6 | 0 | 47 | 3 | 0 | 37 | 13 | 0 | 49 | 1 | 0 | 48 | 2 | 0 |
| Right | Frontal | 42 | 8 | 0 | 34 | 16 | 0 | 38 | 12 | 0 | 42 | 8 | 0 | 38 | 12 | 0 |
|  | Parietal | 39 | 11 | 0 | 33 | 17 | 0 | 34 | 16 | 0 | 41 | 9 | 0 | 39 | 11 | 0 |
|  | Temporal | 34 | 16 | 0 | 31 | 19 | 0 | 29 | 21 | 0 | 32 | 18 | 0 | 34 | 16 | 0 |
|  | Occipital | 38 | 12 | 0 | 40 | 10 | 0 | 36 | 14 | 0 | 40 | 10 | 0 | 40 | 10 | 0 |
| **ADF results for 1^st^ order differenced data** | | | | | | | | | | | | | | | | |
| **Hemisphere** | **Brain Lobe** | **HbO** | | | **HHb** | | | **tHb** | | | **HbDiff** | | | **rSO_2_** | | |
|  |  | **S** | **NS** | **NA** | **S** | **NS** | **NA** | **S** | **NS** | **NA** | **S** | **NS** | **NA** | **S** | **NS** | **NA** |
| Left | Frontal | 50 | 0 | 0 | 50 | 0 | 0 | 50 | 0 | 0 | 50 | 0 | 0 | 50 | 0 | 0 |
|  | Parietal | 50 | 0 | 0 | 50 | 0 | 0 | 50 | 0 | 0 | 50 | 0 | 0 | 50 | 0 | 0 |
|  | Temporal | 50 | 0 | 0 | 50 | 0 | 0 | 50 | 0 | 0 | 50 | 0 | 0 | 50 | 0 | 0 |
|  | Occipital | 50 | 0 | 0 | 50 | 0 | 0 | 50 | 0 | 0 | 50 | 0 | 0 | 50 | 0 | 0 |
| Right | Frontal | 50 | 0 | 0 | 50 | 0 | 0 | 50 | 0 | 0 | 50 | 0 | 0 | 50 | 0 | 0 |
|  | Parietal | 50 | 0 | 0 | 50 | 0 | 0 | 50 | 0 | 0 | 50 | 0 | 0 | 50 | 0 | 0 |
|  | Temporal | 50 | 0 | 0 | 50 | 0 | 0 | 50 | 0 | 0 | 50 | 0 | 0 | 50 | 0 | 0 |
|  | Occipital | 50 | 0 | 0 | 50 | 0 | 0 | 50 | 0 | 0 | 50 | 0 | 0 | 50 | 0 | 0 |
| **KPSS results for non-differenced data** | | | | | | | | | | | | | | | | |
| **Hemisphere** | **Brain Lobe** | **HbO** | | | **HHb** | | | **tHb** | | | **HbDiff** | | | **rSO_2_** | | |
|  |  | **S** | **NS** | **NA** | **S** | **NS** | **NA** | **S** | **NS** | **NA** | **S** | **NS** | **NA** | **S** | **NS** | **NA** |
| Left | Frontal | 8 | 42 | 0 | 11 | 39 | 0 | 10 | 40 | 0 | 6 | 44 | 0 | 7 | 43 | 0 |
|  | Parietal | 4 | 46 | 0 | 10 | 40 | 0 | 6 | 44 | 0 | 8 | 42 | 0 | 11 | 39 | 0 |
|  | Temporal | 12 | 38 | 0 | 12 | 38 | 0 | 8 | 42 | 0 | 10 | 40 | 0 | 12 | 38 | 0 |
|  | Occipital | 14 | 36 | 0 | 14 | 36 | 0 | 11 | 39 | 0 | 16 | 34 | 0 | 17 | 33 | 0 |
| Right | Frontal | 12 | 38 | 0 | 10 | 40 | 0 | 13 | 37 | 0 | 16 | 34 | 0 | 8 | 42 | 0 |
|  | Parietal | 12 | 38 | 0 | 12 | 38 | 0 | 10 | 40 | 0 | 14 | 36 | 0 | 13 | 37 | 0 |
|  | Temporal | 8 | 42 | 0 | 5 | 45 | 0 | 7 | 43 | 0 | 9 | 41 | 0 | 9 | 41 | 0 |
|  | Occipital | 15 | 35 | 0 | 16 | 34 | 0 | 16 | 34 | 0 | 12 | 38 | 0 | 13 | 37 | 0 |
| **KPSS results for 1^st^ order differenced data** | | | | | | | | | | | | | | | | |
| **Hemisphere** | **Brain Lobe** | **HbO** | | | **HHb** | | | **tHb** | | | **HbDiff** | | | **rSO_2_** | | |
|  |  | **S** | **NS** | **NA** | **S** | **NS** | **NA** | **S** | **NS** | **NA** | **S** | **NS** | **NA** | **S** | **NS** | **NA** |
| Left | Frontal | 50 | 0 | 0 | 49 | 1 | 0 | 49 | 1 | 0 | 50 | 0 | 0 | 49 | 1 | 0 |
|  | Parietal | 50 | 0 | 0 | 50 | 0 | 0 | 50 | 0 | 0 | 49 | 1 | 0 | 48 | 2 | 0 |
|  | Temporal | 49 | 1 | 0 | 49 | 1 | 0 | 50 | 0 | 0 | 50 | 0 | 0 | 50 | 0 | 0 |
|  | Occipital | 50 | 0 | 0 | 50 | 0 | 0 | 50 | 0 | 0 | 48 | 2 | 0 | 48 | 2 | 0 |
| Right | Frontal | 50 | 0 | 0 | 50 | 0 | 0 | 50 | 0 | 0 | 50 | 0 | 0 | 50 | 0 | 0 |
|  | Parietal | 49 | 1 | 0 | 50 | 0 | 0 | 50 | 0 | 0 | 50 | 0 | 0 | 50 | 0 | 0 |
|  | Temporal | 49 | 1 | 0 | 49 | 1 | 0 | 49 | 1 | 0 | 50 | 0 | 0 | 50 | 0 | 0 |
|  | Occipital | 50 | 0 | 0 | 50 | 0 | 0 | 50 | 0 | 0 | 50 | 0 | 0 | 50 | 0 | 0 |
| The table presents the results of the ADF and KPSS analysis with the count of subject data that was found to be stationary (S), non-stationary (NS) or unable to assess (NA) using non-differenced and 1^st^ order differenced data sampled at 1 Hz for all the calculated fNIRS signals at each brain lobe of both hemispheres. It was found from these stationarity tests that signals were stationary after 1^st^ order differencing while they were originally non-stationary. *ADF, Augmented Dickey-Fuller; fNIRS, functional near-infrared spectroscopy; HbDiff, hemoglobin difference; HbO, oxyhemoglobin; HHb, deoxyhemoglobin; KPSS, Kwiatkowski–Phillips–Schmidt–Shin; NA, unable to assess stationarity; NS, non-stationary; rSO_2_, regional oxygen saturation; S, stationary; tHb, total hemoglobin.* | | | | | | | | | | | | | | | | |

Appendix S4b: ADF and KPSS Results Showing Stationary vs Non-Stationary vs NA for Calculated fNIRS CA Indices at 250 Hz

| **ADF results for non-differenced data** | | | | | | | | | | | | | | | | |
| --- | --- | --- | --- | --- | --- | --- | --- | --- | --- | --- | --- | --- | --- | --- | --- | --- |
| **Hemisphere** | **Brain Lobe** | **HbOx** | | | **HHbx** | | | **tHbx** | | | **HbDiffx** | | | **COx-a** | | |
|  |  | **S** | **NS** | **NA** | **S** | **NS** | **NA** | **S** | **NS** | **NA** | **S** | **NS** | **NA** | **S** | **NS** | **NA** |
| Left | Frontal | 50 | 0 | 0 | 48 | 2 | 0 | 50 | 0 | 0 | 49 | 1 | 0 | 49 | 1 | 0 |
|  | Parietal | 49 | 1 | 0 | 48 | 2 | 0 | 49 | 1 | 0 | 48 | 2 | 0 | 48 | 2 | 0 |
|  | Temporal | 49 | 1 | 0 | 49 | 1 | 0 | 49 | 1 | 0 | 50 | 0 | 0 | 50 | 0 | 0 |
|  | Occipital | 43 | 7 | 0 | 50 | 0 | 0 | 48 | 2 | 0 | 45 | 5 | 0 | 49 | 1 | 0 |
| Right | Frontal | 48 | 2 | 0 | 50 | 0 | 0 | 50 | 0 | 0 | 50 | 0 | 0 | 48 | 2 | 0 |
|  | Parietal | 49 | 1 | 0 | 48 | 2 | 0 | 50 | 0 | 0 | 46 | 4 | 0 | 46 | 4 | 0 |
|  | Temporal | 49 | 1 | 0 | 49 | 1 | 0 | 50 | 0 | 0 | 50 | 0 | 0 | 50 | 0 | 0 |
|  | Occipital | 50 | 0 | 0 | 49 | 1 | 0 | 49 | 1 | 0 | 49 | 1 | 0 | 49 | 1 | 0 |
| **ADF results for 1^st^ order differenced data** | | | | | | | | | | | | | | | | |
| **Hemisphere** | **Brain Lobe** | **HbOx** | | | **HHbx** | | | **tHbx** | | | **HbDiffx** | | | **COx-a** | | |
|  |  | **S** | **NS** | **NA** | **S** | **NS** | **NA** | **S** | **NS** | **NA** | **S** | **NS** | **NA** | **S** | **NS** | **NA** |
| Left | Frontal | 50 | 0 | 0 | 50 | 0 | 0 | 50 | 0 | 0 | 50 | 0 | 0 | 50 | 0 | 0 |
|  | Parietal | 50 | 0 | 0 | 50 | 0 | 0 | 50 | 0 | 0 | 50 | 0 | 0 | 50 | 0 | 0 |
|  | Temporal | 50 | 0 | 0 | 50 | 0 | 0 | 50 | 0 | 0 | 50 | 0 | 0 | 50 | 0 | 0 |
|  | Occipital | 50 | 0 | 0 | 50 | 0 | 0 | 50 | 0 | 0 | 50 | 0 | 0 | 50 | 0 | 0 |
| Right | Frontal | 50 | 0 | 0 | 50 | 0 | 0 | 50 | 0 | 0 | 50 | 0 | 0 | 50 | 0 | 0 |
|  | Parietal | 50 | 0 | 0 | 50 | 0 | 0 | 50 | 0 | 0 | 50 | 0 | 0 | 50 | 0 | 0 |
|  | Temporal | 50 | 0 | 0 | 50 | 0 | 0 | 50 | 0 | 0 | 50 | 0 | 0 | 50 | 0 | 0 |
|  | Occipital | 50 | 0 | 0 | 50 | 0 | 0 | 50 | 0 | 0 | 50 | 0 | 0 | 50 | 0 | 0 |
| **KPSS results for non-differenced data** | | | | | | | | | | | | | | | | |
| **Hemisphere** | **Brain Lobe** | **HbOx** | | | **HHbx** | | | **tHbx** | | | **HbDiffx** | | | **COx-a** | | |
|  |  | **S** | **NS** | **NA** | **S** | **NS** | **NA** | **S** | **NS** | **NA** | **S** | **NS** | **NA** | **S** | **NS** | **NA** |
| Left | Frontal | 38 | 12 | 0 | 39 | 11 | 0 | 37 | 13 | 0 | 41 | 9 | 0 | 41 | 9 | 0 |
|  | Parietal | 40 | 10 | 0 | 41 | 9 | 0 | 40 | 10 | 0 | 37 | 13 | 0 | 37 | 13 | 0 |
|  | Temporal | 38 | 12 | 0 | 36 | 14 | 0 | 37 | 13 | 0 | 39 | 11 | 0 | 38 | 12 | 0 |
|  | Occipital | 35 | 15 | 0 | 40 | 10 | 0 | 40 | 10 | 0 | 35 | 15 | 0 | 37 | 13 | 0 |
| Right | Frontal | 34 | 16 | 0 | 41 | 9 | 0 | 39 | 11 | 0 | 40 | 10 | 0 | 40 | 10 | 0 |
|  | Parietal | 43 | 7 | 0 | 42 | 8 | 0 | 38 | 12 | 0 | 44 | 6 | 0 | 45 | 5 | 0 |
|  | Temporal | 44 | 6 | 0 | 38 | 12 | 0 | 43 | 7 | 0 | 38 | 12 | 0 | 35 | 15 | 0 |
|  | Occipital | 38 | 12 | 0 | 40 | 10 | 0 | 39 | 11 | 0 | 40 | 10 | 0 | 37 | 13 | 0 |
| **KPSS results for 1^st^ order differenced data** | | | | | | | | | | | | | | | | |
| **Hemisphere** | **Brain Lobe** | **HbOx** | | | **HHbx** | | | **tHbx** | | | **HbDiffx** | | | **COx-a** | | |
|  |  | **S** | **NS** | **NA** | **S** | **NS** | **NA** | **S** | **NS** | **NA** | **S** | **NS** | **NA** | **S** | **NS** | **NA** |
| Left | Frontal | 50 | 0 | 0 | 50 | 0 | 0 | 50 | 0 | 0 | 50 | 0 | 0 | 50 | 0 | 0 |
|  | Parietal | 50 | 0 | 0 | 50 | 0 | 0 | 50 | 0 | 0 | 50 | 0 | 0 | 50 | 0 | 0 |
|  | Temporal | 50 | 0 | 0 | 50 | 0 | 0 | 50 | 0 | 0 | 50 | 0 | 0 | 50 | 0 | 0 |
|  | Occipital | 50 | 0 | 0 | 50 | 0 | 0 | 50 | 0 | 0 | 50 | 0 | 0 | 50 | 0 | 0 |
| Right | Frontal | 50 | 0 | 0 | 50 | 0 | 0 | 50 | 0 | 0 | 50 | 0 | 0 | 50 | 0 | 0 |
|  | Parietal | 50 | 0 | 0 | 50 | 0 | 0 | 50 | 0 | 0 | 50 | 0 | 0 | 50 | 0 | 0 |
|  | Temporal | 50 | 0 | 0 | 50 | 0 | 0 | 50 | 0 | 0 | 50 | 0 | 0 | 50 | 0 | 0 |
|  | Occipital | 50 | 0 | 0 | 50 | 0 | 0 | 50 | 0 | 0 | 50 | 0 | 0 | 50 | 0 | 0 |
| The table presents the results of the ADF and KPSS analysis with the count of subject data that was found to be stationary (S), non-stationary (NS) or unable to assess (NA) using non-differenced and 1^st^ order differenced data sampled at 250 Hz for all the calculated fNIRS indices at each brain lobe of both hemispheres. It was found from these stationarity tests that signals were stationary after 1^st^ order differencing while they were originally non-stationary. *ADF, Augmented Dickey-Fuller; COx-a, cerebral oximetry index with arterial blood pressure; fNIRS, functional near-infrared spectroscopy; HbDiffx, hemoglobin difference index; HbOx, oxyhemoglobin index; HHbx, deoxyhemoglobin index; KPSS, Kwiatkowski–Phillips–Schmidt–Shin; NA, unable to assess stationarity; NS, non-stationary; S, stationary; tHbx, total hemoglobin index.* | | | | | | | | | | | | | | | | |

Appendix S4c: ADF and KPSS Results Showing Stationary vs Non-Stationary vs NA for fNIRS Physiologic Signals at 250 Hz

| **ADF results for non-differenced data** | | | | | | | | | | | | | | | | |
| --- | --- | --- | --- | --- | --- | --- | --- | --- | --- | --- | --- | --- | --- | --- | --- | --- |
| **Hemisphere** | **Brain Lobe** | **HbO** | | | **HHb** | | | **tHb** | | | **HbDiff** | | | **rSO_2_** | | |
|  |  | **S** | **NS** | **NA** | **S** | **NS** | **NA** | **S** | **NS** | **NA** | **S** | **NS** | **NA** | **S** | **NS** | **NA** |
| Left | Frontal | 34 | 16 | 0 | 29 | 21 | 0 | 32 | 18 | 0 | 32 | 18 | 0 | 33 | 17 | 0 |
|  | Parietal | 31 | 19 | 0 | 30 | 20 | 0 | 29 | 21 | 0 | 34 | 16 | 0 | 36 | 14 | 0 |
|  | Temporal | 33 | 17 | 0 | 26 | 24 | 0 | 32 | 18 | 0 | 40 | 10 | 0 | 35 | 15 | 0 |
|  | Occipital | 44 | 6 | 0 | 46 | 4 | 0 | 37 | 13 | 0 | 49 | 1 | 0 | 48 | 2 | 0 |
| Right | Frontal | 43 | 7 | 0 | 34 | 16 | 0 | 38 | 12 | 0 | 42 | 8 | 0 | 39 | 11 | 0 |
|  | Parietal | 39 | 11 | 0 | 34 | 16 | 0 | 36 | 14 | 0 | 41 | 9 | 0 | 39 | 11 | 0 |
|  | Temporal | 35 | 15 | 0 | 31 | 19 | 0 | 30 | 20 | 0 | 33 | 17 | 0 | 33 | 17 | 0 |
|  | Occipital | 37 | 13 | 0 | 40 | 10 | 0 | 37 | 13 | 0 | 40 | 10 | 0 | 38 | 12 | 0 |
| **ADF results for 1^st^ order differenced data** | | | | | | | | | | | | | | | | |
| **Hemisphere** | **Brain Lobe** | **HbO** | | | **HHb** | | | **tHb** | | | **HbDiff** | | | **rSO_2_** | | |
|  |  | **S** | **NS** | **NA** | **S** | **NS** | **NA** | **S** | **NS** | **NA** | **S** | **NS** | **NA** | **S** | **NS** | **NA** |
| Left | Frontal | 50 | 0 | 0 | 50 | 0 | 0 | 50 | 0 | 0 | 50 | 0 | 0 | 50 | 0 | 0 |
|  | Parietal | 50 | 0 | 0 | 50 | 0 | 0 | 50 | 0 | 0 | 50 | 0 | 0 | 50 | 0 | 0 |
|  | Temporal | 50 | 0 | 0 | 50 | 0 | 0 | 50 | 0 | 0 | 50 | 0 | 0 | 50 | 0 | 0 |
|  | Occipital | 50 | 0 | 0 | 50 | 0 | 0 | 50 | 0 | 0 | 50 | 0 | 0 | 50 | 0 | 0 |
| Right | Frontal | 50 | 0 | 0 | 50 | 0 | 0 | 50 | 0 | 0 | 50 | 0 | 0 | 50 | 0 | 0 |
|  | Parietal | 50 | 0 | 0 | 50 | 0 | 0 | 50 | 0 | 0 | 50 | 0 | 0 | 50 | 0 | 0 |
|  | Temporal | 50 | 0 | 0 | 50 | 0 | 0 | 50 | 0 | 0 | 50 | 0 | 0 | 50 | 0 | 0 |
|  | Occipital | 50 | 0 | 0 | 50 | 0 | 0 | 50 | 0 | 0 | 50 | 0 | 0 | 50 | 0 | 0 |
| **KPSS results for non-differenced data** | | | | | | | | | | | | | | | | |
| **Hemisphere** | **Brain Lobe** | **HbO** | | | **HHb** | | | **tHb** | | | **HbDiff** | | | **rSO_2_** | | |
|  |  | **S** | **NS** | **NA** | **S** | **NS** | **NA** | **S** | **NS** | **NA** | **S** | **NS** | **NA** | **S** | **NS** | **NA** |
| Left | Frontal | 8 | 42 | 0 | 11 | 39 | 0 | 11 | 39 | 0 | 6 | 44 | 0 | 7 | 43 | 0 |
|  | Parietal | 4 | 46 | 0 | 10 | 40 | 0 | 6 | 44 | 0 | 8 | 42 | 0 | 11 | 39 | 0 |
|  | Temporal | 12 | 38 | 0 | 12 | 38 | 0 | 8 | 42 | 0 | 10 | 40 | 0 | 13 | 37 | 0 |
|  | Occipital | 14 | 36 | 0 | 14 | 36 | 0 | 11 | 39 | 0 | 16 | 34 | 0 | 16 | 34 | 0 |
| Right | Frontal | 12 | 38 | 0 | 10 | 40 | 0 | 13 | 37 | 0 | 17 | 33 | 0 | 8 | 42 | 0 |
|  | Parietal | 12 | 38 | 0 | 12 | 38 | 0 | 10 | 40 | 0 | 14 | 36 | 0 | 13 | 37 | 0 |
|  | Temporal | 7 | 43 | 0 | 5 | 45 | 0 | 6 | 44 | 0 | 9 | 41 | 0 | 9 | 41 | 0 |
|  | Occipital | 15 | 35 | 0 | 16 | 34 | 0 | 16 | 34 | 0 | 12 | 38 | 0 | 13 | 37 | 0 |
| **KPSS results for 1^st^ order differenced data** | | | | | | | | | | | | | | | | |
| **Hemisphere** | **Brain Lobe** | **HbO** | | | **HHb** | | | **tHb** | | | **HbDiff** | | | **rSO_2_** | | |
|  |  | **S** | **NS** | **NA** | **S** | **NS** | **NA** | **S** | **NS** | **NA** | **S** | **NS** | **NA** | **S** | **NS** | **NA** |
| Left | Frontal | 50 | 0 | 0 | 49 | 1 | 0 | 49 | 1 | 0 | 50 | 0 | 0 | 50 | 0 | 0 |
|  | Parietal | 50 | 0 | 0 | 49 | 1 | 0 | 50 | 0 | 0 | 49 | 1 | 0 | 49 | 1 | 0 |
|  | Temporal | 50 | 0 | 0 | 50 | 0 | 0 | 49 | 1 | 0 | 50 | 0 | 0 | 50 | 0 | 0 |
|  | Occipital | 49 | 1 | 0 | 49 | 1 | 0 | 50 | 0 | 0 | 48 | 2 | 0 | 48 | 2 | 0 |
| Right | Frontal | 50 | 0 | 0 | 50 | 0 | 0 | 50 | 0 | 0 | 50 | 0 | 0 | 50 | 0 | 0 |
|  | Parietal | 50 | 0 | 0 | 49 | 1 | 0 | 50 | 0 | 0 | 49 | 1 | 0 | 49 | 1 | 0 |
|  | Temporal | 50 | 0 | 0 | 49 | 1 | 0 | 49 | 1 | 0 | 50 | 0 | 0 | 50 | 0 | 0 |
|  | Occipital | 50 | 0 | 0 | 50 | 0 | 0 | 50 | 0 | 0 | 50 | 0 | 0 | 50 | 0 | 0 |
| The table presents the results of the ADF and KPSS analysis with the count of subject data that was found to be stationary (S), non-stationary (NS) or unable to assess (NA) using non-differenced and 1^st^ order differenced data sampled at 250 Hz for all the calculated fNIRS signals at each brain lobe of both hemispheres. It was found from these stationarity tests that signals were stationary after 1^st^ order differencing while they were originally non-stationary. *ADF, Augmented Dickey-Fuller; fNIRS, functional near-infrared spectroscopy; HbDiff, hemoglobin difference; HbO, oxyhemoglobin; HHb, deoxyhemoglobin; KPSS, Kwiatkowski–Phillips–Schmidt–Shin; NA, unable to assess stationarity; NS, non-stationary; rSO_2_, regional oxygen saturation; S, stationary; tHb, total hemoglobin.* | | | | | | | | | | | | | | | | |

Appendix S4d: Optimal ARIMA Models Based on AIC of CVR Indices and their Hemispheric Disparity at 250 Hz

| **CVR Index** | **Hemisphere** | **Optimal ARIMA Models (Median [IQR])** | | | |
| --- | --- | --- | --- | --- | --- |
|  |  | **Frontal Lobe** | **Parietal Lobe** | **Temporal Lobe** | **Occipital Lobe** |
| COx-a | Left | (2,1,5) [(1,1,5) – (4,1,1)] | (2,1,2) [(1,1,7) – (4,1,4)] | (2,1,8) [(1,1,6) – (4,1,3)] | (2,1,0) [(1,1,1) – (4,1,0)] |
|  | Right | (2,1,7) [(1,1,9) – (3,1,8)] | (2,1,6) [(1,1,10) – (4,1,6)] | (2,1,7) [(1,1,6) – (3,1,6)] | (2,1,2) [(1,1,6) – (3,1,6)] |
| HbOx | Left | (3,1,2) [(1,1,7) – (5,1,2)] | (2,1,6) [(1,1,7) – (4,1,2)] | (2,1,3) [(1,1,5) – (4,1,8)] | (2,1,5) [(1,1,2) – (3,1,5)] |
|  | Right | (2,1,3) [(1,1,8) – (3,1,8)] | (2,1,3) [(1,1,2) – (4,1,2)] | (2,1,4) [(1,1,7) – (4,1,2)] | (2,1,5) [(1,1,6) – (4,1,5)] |
| HHbx | Left | (2,1,2) [(1,1,7) – (5,1,2)] | (2,1,6) [(1,1,7) – (4,1,4)] | (2,1,1) [(1,1,6) – (3,1,3)] | (2,1,2) [(1,1,1) – (4,1,0)] |
|  | Right | (2,1,1) [(1,1,7) – (3,1,8)] | (2,1,5) [(1,1,7) – (4,1,7)] | (2,1,6) [(1,1,6) – (4,1,8)] | (2,1,2) [(1,1,5) – (3,1,7)] |
| tHbx | Left | (2,1,2) [(1,1,7) – (4,1,2)] | (2,1,8) [(1,1,6) – (4,1,1)] | (3,1,1) [(1,1,6) – (4,1,5)] | (2,1,5) [(1,1,7) – (3,1,8)] |
|  | Right | (2,1,2) [(1,1,9) – (3,1,7)] | (2,1,8) [(2,1,0) – (4,1,4)] | (2,1,4) [(1,1,6) – (3,1,4)] | (2,1,6) [(1,1,7) – (4,1,1)] |
| HbDiffx | Left | (2,1,0) [(1,1,2) – (3,1,9)] | (2,1,0) [(1,1,6) – (3,1,5)] | (2,1,0) [(1,1,5) – (3,1,9)] | (2,1,2) [(1,1,1) – (3,1,10)] |
|  | Right | (2,1,4) [(1,1,5) – (3,1,9)] | (2,1,3) [(1,1,6) – (4,1,3)] | (3,1,2) [(1,1,7) – (5,1,5)] | (2,1,3) [(1,1,6) – (3,1,9)] |
| The table provides median and IQR of optimal ARIMA models based on AIC for CVR indices using data in 250 Hz frequency. *AIC, Akaike Information Criterion; ARIMA, autoregressive integrative moving average; COx-a, cerebral oximetry index with arterial blood pressure; IQR, interquartile range; HbDiffx, hemoglobin difference index; HbOx, oxyhemoglobin index; HHbx, deoxyhemoglobin index; tHbx, total hemoglobin index.* | | | | | |

Appendix S4e: Optimal ARIMA Models Based on AIC of Physiologic Signals and their Hemispheric Disparity

| **Physiologic Variable** | **Hemisphere** | **Optimal ARIMA Models (Median [IQR])** | | | |
| --- | --- | --- | --- | --- | --- |
|  |  | **Frontal Lobe** | **Parietal Lobe** | **Temporal Lobe** | **Occipital Lobe** |
| **1 Hz Sampled Data** | | | | | |
| ABP | – | (4,1,4) [(2,1,8) – (6,1,3)] | | | |
| rSO_2_ | Left | (2,1,5) [(1,1,3) – (4,1,3)] | (3,1,6) [(1,1,10) – (5,1,5)] | (3,1,3) [(2,1,3) – (5,1,4)] | (2,1,5) [(1,1,3) – (5,1,1)] |
|  | Right | (3,1,1) [(2,1,1) – (5,1,6)] | (4,1,4) [(2,1,8) – (7,1,8)] | (3,1,3) [(1,1,5) – (6,1,5)] | (3,1,4) [(2,1,1) – (5,1,2)] |
| HbO | Left | (3,1,9) [(2,1,4) – (7,1,7)] | (3,1,6) [(2,1,2) – (5,1,9)] | (3,1,8) [(1,1,10) – (5,1,5)] | (4,1,3) [(2,1,1) – (5,1,8)] |
|  | Right | (3,1,4) [(2,1,0) – (6,1,5)] | (5,1,5) [(2,1,8) – (6,1,9)] | (3,1,5) [(2,1,5) – (6,1,4)] | (4,1,9) [(2,1,4) – (6,1,7)] |
| HHb | Left | (3,1,3) [(2,1,0) – (7,1,4)] | (3,1,4) [(1,1,7) – (5,1,10)] | (2,1,9) [(2,1,2) – (4,1,9)] | (3,1,4) [(2,1,3) – (5,1,9)] |
|  | Right | (3,1,7) [(2,1,2) – (5,1,8)] | (3,1,4) [(2,1,1) – (7,1,7)] | (3,1,6) [(1,1,9) – (7,1,5)] | (4,1,9) [(2,1,5) – (6,1,7)] |
| tHb | Left | (4,1,5) [(2,1,4) – (6,1,9)] | (3,1,6) [(1,1,7) – (4,1,10)] | (3,1,5) [(1,1,8) – (6,1,5)] | (3,1,3) [(2,1,1) – (6,1,8)] |
|  | Right | (3,1,10) [(1,1,7) – (6,1,5)] | (4,1,7) [(3,1,3) – (7,1,9)] | (3,1,5) [(1,1,10) – (5,1,3)] | (4,1,4) [(2,1,2) – (6,1,5)] |
| HbDiff | Left | (4,1,3) [(3,1,1) – (6,1,0)] | (3,1,4) [(2,1,1) – (7,1,1)] | (4,1,4) [(2,1,3) – (6,1,5)] | (3,1,2) [(1,1,4) – (5,1,1)] |
|  | Right | (3,1,7) [(2,1,0) – (5,1,7)] | (4,1,8) [(2,1,8) – (7,1,8)] | (3,1,4) [(2,1,3) – (6,1,4)] | (3,1,9) [(2,1,3) – (6,1,8)] |
| **250 Hz Sampled Data** | | | | | |
| ABP | – | (5,1,1) [(3,1,3) – (7,1,1)] | | | |
| rSO_2_ | Left | (3,1,6) [(2,1,1) – (6,1,1)] | (3,1,3) [(1,1,10) – (5,1,3)] | (3,1,3) [(2,1,0) – (6,1,7)] | (2,1,3) [(1,1,2) – (4,1,2)] |
|  | Right | (3,1,3) [(2,1,0) – (5,1,1)] | (3,1,8) [(2,1,1) – (6,1,8)] | (2,1,4) [(1,1,3) – (4,1,6)] | (3,1,6) [(2,1,1) – (5,1,1)] |
| HbO | Left | (3,1,7) [(2,1,0) – (5,1,7)] | (3,1,5) [(1,1,4) – (5,1,8)] | (2,1,10) [(1,1,10) – (4,1,7)] | (3,1,3) [(1,1,7) – (6,1,1)] |
|  | Right | (3,1,3) [(1,1,6) – (6,1,1)] | (4,1,5) [(2,1,8) – (6,1,7)] | (4,1,3) [(2,1,3) – (7,1,6)] | (4,1,6) [(2,1,1) – (7,1,7)] |
| HHb | Left | (4,1,1) [(2,1,3) – (7,1,10)] | (3,1,3) [(1,1,10) – (7,1,3)] | (3,1,4) [(2,1,1) – (5,1,9)] | (3,1,1) [(1,1,6) – (5,1,6)] |
|  | Right | (3,1,2) [(2,1,1) – (6,1,1)] | (4,1,1) [(2,1,3) – (7,1,1)] | (3,1,4) [(2,1,2) – (6,1,9)] | (4,1,7) [(3,1,3) – (7,1,1)] |
| tHb | Left | (3,1,4) [(2,1,2) – (6,1,8)] | (3,1,4) [(2,1,0) – (6,1,6)] | (3,1,2) [(2,1,2) – (5,1,5)] | (3,1,6) [(1,1,5) – (7,1,8)] |
|  | Right | (3,1,3) [(1,1,7) – (6,1,5)] | (4,1,4) [(2,1,7) – (7,1,5)] | (3,1,3) [(1,1,3) – (5,1,3)] | (3,1,8) [(2,1,0) – (6,1,5)] |
| HbDiff | Left | (4,1,1) [(2,1,6) – (7,1,2)] | (3,1,5) [(2,1,1) – (6,1,3)] | (4,1,2) [(2,1,3) – (6,1,7)] | (2,1,3) [(1,1,2) – (4,1,4)] |
|  | Right | (3,1,6) [(1,1,6) – (5,1,6)] | (3,1,9) [(2,1,5) – (5,1,9)] | (3,1,6) [(2,1,3) – (5,1,6)] | (3,1,3) [(2,1,1) – (7,1,7)] |
| The table provides median and IQR of optimal ARIMA models based on AIC for physiologic signals using data in 1 Hz and 250 Hz frequencies. *ABP, arterial blood pressure; AIC, Akaike Information Criterion; ARIMA, autoregressive integrative moving average; IQR, interquartile range; HbDiff, hemoglobin difference; HbO, oxyhemoglobin; HHb, deoxyhemoglobin; rSO_2_, regional cerebral oxygen saturation; tHb, total hemoglobin.* | | | | | |
