# Supplementary material for: Detection of regional disparity in cerebrovascular reactivity using a custom whole brain functional near-infrared spectroscopy based mapping system: A prospective observational study
Source: PLOS Digit Health. 2026 Apr 15;5(4):e0001349. doi: 10.1371/journal.pdig.0001349 (PMC13082728; doi:10.1371/journal.pdig.0001349)
Supplement: S5 Appendix — (DOCX) [file pdig.0001349.s005.docx]

**Appendix S5 – VARIMA IRF and Granger Causality Analyses**

Appendix S5 – Table of Contents

[Appendix S5a: Hemispheric Responsiveness using Impulse Response Coefficients of Optimal VARIMA Model at 250 Hz 2](#_Toc213066162)

[Appendix S5b: Granger Causal Directionality Results Based on Greater F-Statistic at 250 Hz 3](#_Toc213066163)

Appendix S5a: Hemispheric Responsiveness using Impulse Response Coefficients of Optimal VARIMA Model at 250 Hz

| **Direction** | **Hemisphere** | **Frontal Lobe [% (count)]** | | **Parietal Lobe [% (count)]** | | **Temporal Lobe [% (count)]** | | **Occipital Lobe [% (count)]** | |
| --- | --- | --- | --- | --- | --- | --- | --- | --- | --- |
|  |  | **>0.1%** | **NA** | **>0.1%** | **NA** | **>0.1%** | **NA** | **>0.1%** | **NA** |
| ABP ® rSO_2_ | Left | 84% (42) | 0% (0) | 90% (45) | 0% (0) | 96% (48) | 2% (1) | 88% (44) | 0% (0) |
| rSO_2_ ® ABP |  | 84% (42) | 0% (0) | 92% (46) | 0% (0) | 94% (47) | 2% (1) | 86% (43) | 0% (0) |
| ABP ® rSO_2_ | Right | 98% (49) | 0% (0) | 90% (45) | 4% (2) | 86% (43) | 6% (3) | 94% (47) | 4% (2) |
| rSO_2_ ® ABP |  | 100% (50) | 0% (0) | 90% (45) | 4% (2) | 86% (43) | 6% (3) | 92% (46) | 4% (2) |
| ABP ® HbO | Left | 98% (49) | 0% (0) | 84% (42) | 4% (2) | 82% (41) | 0% (0) | 82% (41) | 2% (1) |
| HbO ® ABP |  | 98% (49) | 0% (0) | 82% (41) | 4% (2) | 88% (44) | 0% (0) | 84% (42) | 2% (1) |
| ABP ® HbO | Right | 94% (47) | 0% (0) | 96% (48) | 2% (1) | 88% (44) | 2% (1) | 98% (49) | 0% (0) |
| HbO ® ABP |  | 92% (46) | 0% (0) | 96% (48) | 2% (1) | 90% (45) | 2% (1) | 100% (50) | 0% (0) |
| ABP ® HHb | Left | 84% (42) | 6% (3) | 94% (47) | 0% (0) | 84% (42) | 0% (0) | 82% (41) | 4% (2) |
| HHb ® ABP |  | 84% (42) | 6% (3) | 96% (48) | 0% (0) | 88% (44) | 0% (0) | 80% (40) | 4% (2) |
| ABP ® HHb | Right | 80% (40) | 6% (3) | 94% (47) | 0% (0) | 96% (48) | 0% (0) | 78% (39) | 6% (3) |
| HHb ® ABP |  | 80% (40) | 6% (3) | 94% (47) | 0% (0) | 96% (48) | 0% (0) | 80% (40) | 6% (3) |
| ABP ® tHb | Left | 88% (44) | 0% (0) | 88% (44) | 0% (0) | 90% (45) | 4% (2) | 86% (43) | 6% (3) |
| tHb ® ABP |  | 88% (44) | 0% (0) | 88% (44) | 0% (0) | 88% (44) | 4% (2) | 86% (43) | 6% (3) |
| ABP ® tHb | Right | 92% (46) | 0% (0) | 90% (45) | 2% (1) | 88% (44) | 0% (0) | 92% (46) | 0% (0) |
| tHb ® ABP |  | 90% (45) | 0% (0) | 90% (45) | 2% (1) | 92% (46) | 0% (0) | 92% (46) | 0% (0) |
| ABP ® HbDiff | Left | 90% (45) | 2% (1) | 86% (43) | 0% (0) | 88% (44) | 4% (2) | 88% (44) | 4% (2) |
| HbDiff ® ABP |  | 92% (46) | 2% (1) | 88% (44) | 0% (0) | 86% (43) | 4% (2) | 82% (41) | 4% (2) |
| ABP ® HbDiff | Right | 96% (48) | 0% (0) | 94% (47) | 2% (1) | 94% (47) | 0% (0) | 94% (47) | 0% (0) |
| HbDiff ® ABP |  | 96% (48) | 0% (0) | 94% (47) | 2% (1) | 96% (48) | 0% (0) | 94% (47) | 0% (0) |
| The table shows the hemispheric responsiveness of fNIRS signals using Impulse Response Coefficients of Optimal VARIMA model using 250 Hz data. *ABP, arterial blood pressure; HbDiff, fNIRS, functional near-infrared spectroscopy; hemoglobin difference; HbO, oxyhemoglobin; HHb, deoxyhemoglobin; rSO_2_, regional cerebral oxygen saturation; tHb, total hemoglobin; VARIMA, vector autoregressive integrative moving average.* | | | | | | | | | |

Appendix S5b: Granger Causal Directionality Results Based on Greater F-Statistic at 250 Hz

| **Signal** | **Direction** | **Hemisphere** | **Frontal Lobe [% (count)]** | **Parietal Lobe [% (count)]** | **Temporal Lobe [% (count)]** | **Occipital Lobe [% (count)]** |
| --- | --- | --- | --- | --- | --- | --- |
| ABP & rSO_2_ | ABP ® rSO_2_ | Left | 56% (28) | 58% (29) | 62% (31) | 46% (23) |
|  | rSO_2_ ® ABP |  | 44% (22) | 42% (21) | 38% (19) | 54% (27) |
|  | ABP ® rSO_2_ | Right | 52% (26) | 60% (30) | 54% (27) | 52% (26) |
|  | rSO_2_ ® ABP |  | 48% (24) | 40% (20) | 46% (23) | 48% (24) |
| ABP & HbO | ABP ® HbO | Left | 60% (30) | 62% (31) | 42% (21) | 60% (30) |
|  | HbO ® ABP |  | 40% (20) | 38% (19) | 58% (29) | 40% (20) |
|  | ABP ® HbO | Right | 58% (29) | 54% (27) | 62% (31) | 52% (26) |
|  | HbO ® ABP |  | 42% (21) | 46% (23) | 38% (19) | 48% (24) |
| ABP & HHb | ABP ® HHb | Left | 56% (28) | 50% (25) | 60% (30) | 48% (24) |
|  | HHb ® ABP |  | 44% (22) | 50% (25) | 40% (20) | 52% (26) |
|  | ABP ® HHb | Right | 58% (29) | 62% (31) | 62% (31) | 48% (24) |
|  | HHb ® ABP |  | 42% (21) | 38% (19) | 38% (19) | 52% (26) |
| ABP & tHb | ABP ® tHb | Left | 58% (29) | 44% (22) | 48% (24) | 46% (23) |
|  | tHb ® ABP |  | 42% (21) | 56% (28) | 52% (26) | 54% (27) |
|  | ABP ® tHb | Right | 50% (25) | 50% (25) | 72% (36) | 58% (29) |
|  | tHb ® ABP |  | 50% (25) | 50% (25) | 28% (14) | 42% (21) |
| ABP & HbDiff | ABP ® HbDiff | Left | 50% (25) | 48% (24) | 48% (24) | 46% (23) |
|  | HbDiff ® ABP |  | 50% (25) | 52% (26) | 52% (26) | 54% (27) |
|  | ABP ® HbDiff | Right | 54% (27) | 58% (29) | 56% (28) | 52% (26) |
|  | HbDiff ® ABP |  | 46% (23) | 42% (21) | 44% (22) | 48% (24) |
| The table shows the Granger causal directionality results between ABP and fNIRS signals using 250 Hz data. *ABP, arterial blood pressure; fNIRS, functional near-infrared spectroscopy; HbDiff, hemoglobin difference; HbO, oxyhemoglobin; HHb, deoxyhemoglobin; rSO_2_, regional cerebral oxygen saturation; tHb, total hemoglobin.* | | | | | | |
