# Supplementary material for: Detection of regional disparity in cerebrovascular reactivity using a custom whole brain functional near-infrared spectroscopy based mapping system: A prospective observational study
Source: PLOS Digit Health. 2026 Apr 15;5(4):e0001349. doi: 10.1371/journal.pdig.0001349 (PMC13082728; doi:10.1371/journal.pdig.0001349)
Supplement: S6 Appendix — (DOCX) [file pdig.0001349.s006.docx]

**Appendix S6 – Common Parameters Subgroup Analysis**

Appendix S6 – Table of Contents

[Appendix S6a: Subgrouped ANAM Results 2](#_Toc213066393)

[Appendix S6b: Subgrouped CVR Indices Results Using 10-Second Decimated Data 3](#_Toc213066394)

[Appendix S6c: Subgrouped Physiologic Results Using 10-Second Decimated Data 5](#_Toc213066395)

[Appendix S6d: Subgrouped Physiologic Results Using Raw Data 8](#_Toc213066396)

[Appendix S6e: Subgrouped Percent Time Results of rSO_2_ and CVR Indices Using 10-Second Decimated Data 11](#_Toc213066397)

[Appendix S6f: Subgrouped Percent Time Results of rSO_2_ Using Raw Data 15](#_Toc213066398)

[Appendix S6g: Subgrouped Regional Hemispheric Disparity Analysis on CVR Indices Using 10-Second Decimated Data 16](#_Toc213066399)

[Appendix S6h: Subgrouped Regional Hemispheric Disparity Analysis on Physiologic Signals Using 10-Second Decimated Data 17](#_Toc213066400)

[Appendix S6i: Subgrouped Regional Hemispheric Disparity Analysis on Physiologic Signals Using Raw Data 18](#_Toc213066401)

[Appendix S6j: Subgrouped Optimal ARIMA Models Based on AIC of Physiologic Signals and their Hemispheric Disparity 19](#_Toc213066402)

[Appendix S6k: Subgrouped Hemispheric Responsiveness using Impulse Response Coefficients of Optimal VARIMA Model 23](#_Toc213066403)

[Appendix S6l: Subgrouped Granger Causal Directionality Results Based on Greater F-Statistic 25](#_Toc213066404)

Appendix S6a: Subgrouped ANAM Results

| **Variable** | **Subgrouped ANAM [Median (IQR)]** | | | | | |
| --- | --- | --- | --- | --- | --- | --- |
|  | **Age < 40 [n=38]** | **Age 40 – 60 [n=12]** | **Males [n=28]** | **Females [n=22]** | **Left Hand Dominance [n=1]** | **Right Hand Dominance [n=49]** |
| Standard Continuous Performance Test | | | | | | |
| Median Response Time (msec) | 391 (359.13 – 422.63) | 428.25 (379.88 – 459.75) | 398.75 (364.13 – 432.13) | 392.5 (369.13 – 426.75) | 423 (423 – 423) | 394 (365 – 430.5) |
| Correct (%) | 100 (100 – 100) | 100 (99.38 – 100) | 100 (100 – 100) | 100 (100 – 100) | 100 (100 – 100) | 100 (100 – 100) |
| Manikin Test | | | | | | |
| Median Response Time (msec) | 1242.25 (919.5 – 2059.25) | 1835.75 (1147 – 2156) | 1331.25 (980.38 – 2258.88) | 1494 (933.13 – 2055.75) | 1517 (1517 – 1517) | 1378 (966.5 – 2126) |
| Correct (%) | 93.75 (76.56 – 96.88) | 79.69 (61.72 – 90.62) | 89.06 (74.22 – 96.88) | 93.75 (67.97 – 96.88) | 100 (100 – 100) | 90.62 (71.88 – 96.88) |
| Pursuit Tracking Test | | | | | | |
| Median Distance | 7 (6.25 – 8) | 8 (8 – 9.5) | 8 (7 – 8) | 8 (7 – 9.75) | 5 (5 – 5) | 8 (7 – 8) |
| Correct (%) | 99.54 (98.85 – 99.92) | 99.21 (97.69 – 99.79) | 99.54 (98.92 – 99.92) | 99.38 (97.81 – 99.9) | 99.83 (99.83 – 99.83) | 99.5 (98.33 – 99.92) |
| Switching Test | | | | | | |
| Median Response Time (msec) | 1574 (1353.13 – 2275.25) | 2018.5 (1952.5 – 2174.88) | 1839.25 (1413.13 – 2240.13) | 1693 (1400.5 – 2152) | 1548 (1548 – 1548) | 1798 (1397 – 2219.5) |
| Correct (%) | 92.97 (85.94 – 95.31) | 87.5 (80.47 – 92.97) | 92.97 (85.55 – 95.7) | 92.19 (80.86 – 93.75) | 96.88 (96.88 – 96.88) | 92.19 (84.38 – 95.31) |
| Stroop Test | | | | | | |
| Interscore | 14.21 (7.88 – 21.13) | 10.49 (4.96 – 15.66) | 14.71 (7.7 – 21.06) | 12.31 (6.26 – 16.33) | 7.3 (7.3 – 7.3) | 13.63 (7.83 – 21) |
| Stroop Test (Word) | | | | | | |
| Median Response Time (msec) | 676.5 (613.88 – 785.13) | 894.25 (791.25 – 1072.5) | 695 (637.5 – 799.38) | 768.25 (654.25 – 913.25) | 691 (691 – 691) | 730 (641 – 843.5) |
| Correct (%) | 96.08 (91.22 – 98.31) | 97.53 (96.01 – 100) | 95.38 (91.77 – 98.69) | 97.4 (93.7 – 99.62) | 100 (100 – 100) | 96.36 (91.8 – 98.46) |
| Stroop Test (Colour) | | | | | | |
| Median Response Time (msec) | 627.25 (563.63 – 675.63) | 857.25 (818.25 – 951.38) | 653.25 (594 – 754.75) | 653.5 (571.5 – 843.63) | 638.5 (638.5 – 638.5) | 656.5 (579 – 828) |
| Correct (%) | 96.88 (93.58 – 100) | 100 (97.99 – 100) | 96.69 (93.97 – 100) | 99.26 (93.97 – 100) | 100 (100 – 100) | 97.75 (93.33 – 100) |
| Stroop Test (Colour & Word) | | | | | | |
| Median Response Time (msec) | 808.5 (655.75 – 1051) | 1193.5 (934.5 – 1522.75) | 797.5 (676.75 – 1076) | 1014.5 (772.25 – 1195.75) | 1052 (1052 – 1052) | 836 (691 – 1189) |
| Correct (%) | 93.29 (88.88 – 95.64) | 97.28 (95.04 – 100) | 94.92 (90.39 – 98.73) | 93.43 (90.44 – 96.66) | 100 (100 – 100) | 93.75 (90.38 – 97.83) |
| This table shows the ANAM scores for various tests subgrouped by common parameters. *ANAM, automated neuropsychological assessment metrics; IQR, interquartile range.* | | | | | | |

Appendix S6b: Subgrouped CVR Indices Results Using 10-Second Decimated Data

| **CVR Index** | **Brain Lobe** | **Hemisphere** | **Subgroups** | | | | | | | | | | | |
| --- | --- | --- | --- | --- | --- | --- | --- | --- | --- | --- | --- | --- | --- | --- |
|  |  |  | **Age < 40 [n=38]** | | **Age 40 – 60 [n=12]** | | **Males [n=28]** | | **Females [n=22]** | | **Left Hand Dominance [n=1]** | | **Right Hand Dominance [n=49]** | |
|  |  |  | **Median (IQR)** | **p-value** | **Median (IQR)** | **p-value** | **Median (IQR)** | **p-value** | **Median (IQR)** | **p-value** | **Median (IQR)** | **p-value** | **Median (IQR)** | **p-value** |
| **1 Hz Sampled Data** | | | | | | | | | | | | | | |
| COx-a (au) | Frontal | Left | -0.01 (-0.23 – 0.17) | 0.1888 | 0.02 (-0.22 – 0.2) | 0.6236 | -0.01 (-0.22 – 0.16) | 0.2831 | 0.01 (-0.23 – 0.22) | 0.7336 | 0.01 (-0.19 – 0.2) | 1 | 0 (-0.23 – 0.19) | 0.2439 |
|  |  | Right | 0 (-0.18 – 0.22) |  | -0.02 (-0.24 – 0.2) |  | 0 (-0.18 – 0.19) |  | 0.01 (-0.22 – 0.22) |  | -0.06 (-0.2 – 0.12) |  | 0 (-0.19 – 0.21) |  |
|  | Parietal | Left | 0 (-0.19 – 0.2) | 0.7278 | 0 (-0.2 – 0.19) | 0.5067 | 0.01 (-0.2 – 0.19) | 0.4965 | -0.01 (-0.2 – 0.2) | 1 | 0 (-0.22 – 0.15) | 1 | 0 (-0.19 – 0.2) | 0.696 |
|  |  | Right | -0.01 (-0.2 – 0.19) |  | -0.03 (-0.18 – 0.18) |  | -0.02 (-0.2 – 0.18) |  | 0 (-0.2 – 0.22) |  | -0.11 (-0.26 – 0.09) |  | -0.01 (-0.19 – 0.19) |  |
|  | Temporal | Left | -0.01 (-0.2 – 0.19) | 0.7356 | 0.02 (-0.16 – 0.18) | 0.5444 | 0 (-0.2 – 0.17) | 0.4363 | 0.02 (-0.16 – 0.21) | 0.3538 | 0 (-0.13 – 0.15) | 1 | 0 (-0.2 – 0.19) | 0.8368 |
|  |  | Right | -0.01 (-0.18 – 0.2) |  | 0.01 (-0.19 – 0.18) |  | 0.01 (-0.18 – 0.21) |  | -0.01 (-0.2 – 0.17) |  | 0.11 (0 – 0.26) |  | -0.01 (-0.18 – 0.2) |  |
|  | Occipital | Left | 0 (-0.15 – 0.14) | 0.6144 | 0.01 (-0.14 – 0.14) | 0.5834 | 0 (-0.15 – 0.14) | 0.5281 | 0.01 (-0.14 – 0.15) | 0.6138 | -0.11 (-0.24 – 0.02) | 1 | 0 (-0.14 – 0.14) | 0.3822 |
|  |  | Right | 0 (-0.19 – 0.2) |  | 0.01 (-0.16 – 0.19) |  | -0.01 (-0.2 – 0.19) |  | 0.01 (-0.17 – 0.21) |  | -0.11 (-0.22 – 0.06) |  | 0.01 (-0.18 – 0.2) |  |
| HbOx (au) | Frontal | Left | -0.02 (-0.22 – 0.18) | 0.0553 | -0.01 (-0.23 – 0.17) | 0.8399 | -0.02 (-0.21 – 0.17) | 0.0868 | -0.01 (-0.23 – 0.18) | 0.3538 | 0.11 (-0.04 – 0.25) | 1 | -0.02 (-0.22 – 0.18) | **0.033** |
|  |  | Right | 0.02 (-0.19 – 0.22) |  | -0.01 (-0.22 – 0.21) |  | 0 (-0.18 – 0.22) |  | 0.02 (-0.22 – 0.23) |  | -0.03 (-0.15 – 0.15) |  | 0.01 (-0.2 – 0.22) |  |
|  | Parietal | Left | 0.01 (-0.2 – 0.23) | 0.9627 | -0.03 (-0.23 – 0.19) | 0.2602 | 0.01 (-0.18 – 0.21) | 0.6288 | 0.01 (-0.21 – 0.25) | 0.6812 | 0 (-0.17 – 0.13) | 1 | 0.01 (-0.2 – 0.23) | 0.629 |
|  |  | Right | 0.01 (-0.19 – 0.22) |  | 0.02 (-0.19 – 0.22) |  | 0.02 (-0.17 – 0.21) |  | 0.01 (-0.21 – 0.23) |  | 0.02 (-0.2 – 0.14) |  | 0.02 (-0.18 – 0.22) |  |
|  | Temporal | Left | 0.02 (-0.21 – 0.2) | 0.1623 | 0 (-0.19 – 0.16) | 0.3123 | 0.01 (-0.2 – 0.19) | 0.2413 | 0.02 (-0.2 – 0.18) | 0.8419 | 0.01 (-0.13 – 0.18) | 1 | 0.01 (-0.21 – 0.19) | 0.5459 |
|  |  | Right | 0.04 (-0.17 – 0.22) |  | -0.05 (-0.24 – 0.18) |  | 0.04 (-0.18 – 0.22) |  | -0.04 (-0.19 – 0.18) |  | 0.13 (-0.03 – 0.3) |  | 0 (-0.18 – 0.21) |  |
|  | Occipital | Left | 0.02 (-0.16 – 0.19) | 0.4767 | 0.02 (-0.13 – 0.14) | 0.931 | 0.02 (-0.15 – 0.21) | 0.3461 | 0.02 (-0.14 – 0.15) | 0.9345 | -0.03 (-0.18 – 0.1) | 1 | 0.02 (-0.14 – 0.18) | 0.4907 |
|  |  | Right | 0 (-0.18 – 0.2) |  | 0 (-0.19 – 0.21) |  | -0.02 (-0.2 – 0.2) |  | 0.02 (-0.17 – 0.22) |  | -0.03 (-0.19 – 0.11) |  | 0 (-0.18 – 0.21) |  |
| HHbx (au) | Frontal | Left | 0 (-0.19 – 0.21) | 0.9959 | -0.01 (-0.21 – 0.19) | 0.931 | 0 (-0.18 – 0.19) | 0.6288 | 0 (-0.22 – 0.22) | 0.7872 | 0.08 (-0.06 – 0.22) | 1 | 0 (-0.19 – 0.19) | 0.9151 |
|  |  | Right | 0.01 (-0.18 – 0.2) |  | 0.01 (-0.18 – 0.23) |  | 0.01 (-0.16 – 0.2) |  | -0.01 (-0.22 – 0.23) |  | 0.08 (-0.13 – 0.21) |  | 0.01 (-0.18 – 0.22) |  |
|  | Parietal | Left | 0 (-0.19 – 0.2) | 0.8233 | -0.01 (-0.25 – 0.18) | **0.0404** | 0 (-0.2 – 0.2) | 0.4268 | 0 (-0.23 – 0.21) | 0.3185 | 0 (-0.18 – 0.2) | 1 | 0 (-0.22 – 0.2) | 0.2439 |
|  |  | Right | 0.01 (-0.18 – 0.2) |  | 0.06 (-0.16 – 0.26) |  | 0.01 (-0.18 – 0.2) |  | 0.01 (-0.17 – 0.26) |  | 0.09 (-0.09 – 0.24) |  | 0.01 (-0.18 – 0.21) |  |
|  | Temporal | Left | 0.01 (-0.18 – 0.21) | 0.5161 | -0.04 (-0.21 – 0.15) | 0.665 | 0.01 (-0.17 – 0.23) | 0.9935 | -0.03 (-0.21 – 0.16) | 0.2267 | 0 (-0.14 – 0.16) | 1 | -0.01 (-0.19 – 0.2) | 0.3669 |
|  |  | Right | 0.01 (-0.19 – 0.22) |  | -0.02 (-0.22 – 0.18) |  | 0.01 (-0.19 – 0.22) |  | 0 (-0.19 – 0.2) |  | 0.01 (-0.11 – 0.15) |  | 0.01 (-0.19 – 0.22) |  |
|  | Occipital | Left | 0.01 (-0.14 – 0.17) | 0.6439 | -0.01 (-0.14 – 0.13) | 0.7075 | 0.02 (-0.12 – 0.18) | 0.3896 | 0 (-0.15 – 0.16) | 0.8419 | 0.13 (0.02 – 0.25) | 1 | 0.01 (-0.14 – 0.17) | 0.5318 |
|  |  | Right | 0.02 (-0.17 – 0.22) |  | 0 (-0.19 – 0.19) |  | 0.02 (-0.17 – 0.22) |  | -0.01 (-0.19 – 0.2) |  | 0.06 (-0.14 – 0.21) |  | 0.01 (-0.18 – 0.21) |  |
| tHbx (au) | Frontal | Left | -0.01 (-0.21 – 0.19) | 0.2965 | -0.01 (-0.21 – 0.19) | 0.977 | -0.02 (-0.21 – 0.18) | 0.1381 | 0 (-0.21 – 0.2) | 0.8603 | 0.11 (-0.06 – 0.27) | 1 | -0.01 (-0.21 – 0.19) | 0.2137 |
|  |  | Right | 0.01 (-0.18 – 0.22) |  | -0.02 (-0.23 – 0.22) |  | 0.01 (-0.16 – 0.23) |  | 0 (-0.19 – 0.21) |  | -0.01 (-0.12 – 0.14) |  | 0.01 (-0.18 – 0.22) |  |
|  | Parietal | Left | 0.01 (-0.2 – 0.22) | 0.8314 | -0.04 (-0.24 – 0.18) | **0.0086** | 0.01 (-0.19 – 0.21) | 0.5389 | -0.02 (-0.23 – 0.2) | 0.1361 | -0.02 (-0.19 – 0.12) | 1 | -0.01 (-0.21 – 0.2) | 0.1725 |
|  |  | Right | 0.01 (-0.18 – 0.22) |  | 0.05 (-0.19 – 0.25) |  | 0.01 (-0.18 – 0.22) |  | 0.02 (-0.2 – 0.25) |  | 0.09 (-0.14 – 0.22) |  | 0.01 (-0.18 – 0.23) |  |
|  | Temporal | Left | 0.01 (-0.18 – 0.21) | 0.4576 | -0.03 (-0.21 – 0.15) | 0.1749 | 0 (-0.19 – 0.19) | 0.5718 | -0.01 (-0.2 – 0.19) | 0.9532 | 0.03 (-0.11 – 0.18) | 1 | 0 (-0.2 – 0.2) | 0.7654 |
|  |  | Right | 0.03 (-0.18 – 0.22) |  | -0.06 (-0.24 – 0.18) |  | 0.03 (-0.18 – 0.21) |  | -0.01 (-0.22 – 0.2) |  | 0.08 (-0.1 – 0.22) |  | 0.01 (-0.19 – 0.21) |  |
|  | Occipital | Left | 0.01 (-0.15 – 0.2) | 0.8885 | 0.01 (-0.15 – 0.16) | 0.795 | 0.03 (-0.14 – 0.21) | 0.7743 | 0 (-0.16 – 0.17) | 0.8235 | 0.05 (-0.09 – 0.23) | 1 | 0.01 (-0.15 – 0.18) | 0.9717 |
|  |  | Right | 0.01 (-0.18 – 0.21) |  | 0.01 (-0.19 – 0.22) |  | 0.01 (-0.19 – 0.21) |  | 0 (-0.18 – 0.22) |  | 0.02 (-0.18 – 0.15) |  | 0.01 (-0.18 – 0.21) |  |
| HbDiffx (au) | Frontal | Left | -0.01 (-0.19 – 0.17) | 0.1131 | 0.01 (-0.22 – 0.18) | 0.977 | -0.01 (-0.19 – 0.17) | 0.216 | 0.01 (-0.22 – 0.2) | 0.474 | 0.03 (-0.15 – 0.19) | 1 | 0 (-0.21 – 0.18) | 0.1231 |
|  |  | Right | 0.02 (-0.18 – 0.23) |  | 0 (-0.25 – 0.18) |  | 0.01 (-0.18 – 0.22) |  | 0.02 (-0.2 – 0.23) |  | -0.05 (-0.18 – 0.14) |  | 0.01 (-0.18 – 0.23) |  |
|  | Parietal | Left | 0.01 (-0.19 – 0.2) | 0.5642 | 0 (-0.22 – 0.19) | 0.7508 | 0.01 (-0.19 – 0.2) | 0.4659 | 0 (-0.19 – 0.2) | 0.7872 | 0 (-0.22 – 0.15) | 1 | 0 (-0.19 – 0.2) | 0.7492 |
|  |  | Right | -0.01 (-0.21 – 0.19) |  | -0.02 (-0.19 – 0.2) |  | -0.02 (-0.2 – 0.19) |  | 0.03 (-0.21 – 0.22) |  | -0.12 (-0.26 – 0.09) |  | -0.01 (-0.21 – 0.19) |  |
|  | Temporal | Left | 0 (-0.18 – 0.18) | 0.971 | 0.01 (-0.17 – 0.18) | 0.5067 | 0 (-0.19 – 0.18) | 0.6523 | 0.01 (-0.17 – 0.21) | 0.2649 | 0.01 (-0.13 – 0.16) | 1 | 0 (-0.18 – 0.18) | 0.5412 |
|  |  | Right | 0.02 (-0.18 – 0.21) |  | -0.03 (-0.22 – 0.15) |  | 0.02 (-0.18 – 0.21) |  | 0 (-0.19 – 0.18) |  | 0.14 (0.02 – 0.28) |  | 0 (-0.18 – 0.2) |  |
|  | Occipital | Left | 0 (-0.15 – 0.16) | 0.8967 | 0 (-0.13 – 0.13) | 0.5444 | 0 (-0.15 – 0.17) | 0.8122 | 0.01 (-0.12 – 0.15) | 0.5652 | -0.09 (-0.22 – 0.04) | 1 | 0 (-0.14 – 0.16) | 0.8091 |
|  |  | Right | -0.01 (-0.19 – 0.2) |  | 0.01 (-0.17 – 0.19) |  | -0.02 (-0.2 – 0.2) |  | 0.02 (-0.17 – 0.23) |  | -0.06 (-0.19 – 0.05) |  | 0.01 (-0.18 – 0.2) |  |
| MAD of COx-a (au) | Frontal | Left | 0.21 (0.18 – 0.24) | 0.6588 | 0.2 (0.18 – 0.22) | 0.3408 | 0.2 (0.17 – 0.22) | 0.8249 | 0.22 (0.2 – 0.23) | 0.7513 | 0.19 (0.19 – 0.19) | 1 | 0.21 (0.18 – 0.23) | 0.9603 |
|  |  | Right | 0.2 (0.17 – 0.23) |  | 0.22 (0.19 – 0.23) |  | 0.2 (0.17 – 0.23) |  | 0.21 (0.18 – 0.26) |  | 0.16 (0.16 – 0.16) |  | 0.21 (0.18 – 0.23) |  |
|  | Parietal | Left | 0.19 (0.16 – 0.21) | 0.2965 | 0.19 (0.17 – 0.2) | 0.2366 | 0.18 (0.16 – 0.2) | 0.1176 | 0.19 (0.19 – 0.23) | 0.7336 | 0.17 (0.17 – 0.17) | 1 | 0.19 (0.16 – 0.21) | 0.1357 |
|  |  | Right | 0.2 (0.17 – 0.24) |  | 0.2 (0.19 – 0.21) |  | 0.19 (0.17 – 0.22) |  | 0.21 (0.19 – 0.23) |  | 0.17 (0.17 – 0.17) |  | 0.2 (0.17 – 0.22) |  |
|  | Temporal | Left | 0.2 (0.16 – 0.23) | 0.8967 | 0.17 (0.16 – 0.24) | 0.977 | 0.19 (0.16 – 0.22) | 0.4268 | 0.22 (0.17 – 0.24) | 0.3418 | 0.14 (0.14 – 0.14) | 1 | 0.2 (0.16 – 0.23) | 0.8368 |
|  |  | Right | 0.21 (0.16 – 0.23) |  | 0.18 (0.17 – 0.22) |  | 0.21 (0.17 – 0.22) |  | 0.18 (0.16 – 0.22) |  | 0.12 (0.12 – 0.12) |  | 0.2 (0.17 – 0.23) |  |
|  | Occipital | Left | 0.14 (0.13 – 0.16) | **<0.001** | 0.13 (0.12 – 0.15) | **<0.001** | 0.14 (0.13 – 0.16) | **<0.001** | 0.13 (0.12 – 0.16) | **<0.001** | 0.13 (0.13 – 0.13) | 1 | 0.14 (0.13 – 0.16) | **<0.001** |
|  |  | Right | 0.21 (0.17 – 0.22) |  | 0.19 (0.16 – 0.22) |  | 0.21 (0.17 – 0.22) |  | 0.19 (0.17 – 0.22) |  | 0.14 (0.14 – 0.14) |  | 0.2 (0.17 – 0.22) |  |
| MAD of HbOx (au) | Frontal | Left | 0.2 (0.17 – 0.22) | 0.1959 | 0.2 (0.18 – 0.21) | 0.2855 | 0.19 (0.16 – 0.21) | 0.2831 | 0.21 (0.19 – 0.22) | 0.1242 | 0.14 (0.14 – 0.14) | 1 | 0.2 (0.17 – 0.22) | 0.07 |
|  |  | Right | 0.21 (0.18 – 0.23) |  | 0.21 (0.19 – 0.25) |  | 0.2 (0.18 – 0.23) |  | 0.23 (0.19 – 0.25) |  | 0.14 (0.14 – 0.14) |  | 0.21 (0.19 – 0.24) |  |
|  | Parietal | Left | 0.2 (0.18 – 0.22) | 0.5572 | 0.21 (0.18 – 0.22) | 0.3123 | 0.19 (0.16 – 0.21) | 0.7618 | 0.22 (0.19 – 0.23) | 0.8787 | 0.14 (0.14 – 0.14) | 1 | 0.2 (0.18 – 0.22) | 0.949 |
|  |  | Right | 0.21 (0.18 – 0.25) |  | 0.19 (0.18 – 0.2) |  | 0.19 (0.17 – 0.23) |  | 0.21 (0.18 – 0.26) |  | 0.15 (0.15 – 0.15) |  | 0.2 (0.18 – 0.24) |  |
|  | Temporal | Left | 0.2 (0.18 – 0.24) | 0.1888 | 0.19 (0.15 – 0.2) | 0.5067 | 0.19 (0.18 – 0.23) | 0.4268 | 0.19 (0.16 – 0.23) | 0.7872 | 0.16 (0.16 – 0.16) | 1 | 0.2 (0.18 – 0.23) | 0.4179 |
|  |  | Right | 0.19 (0.17 – 0.21) |  | 0.19 (0.16 – 0.24) |  | 0.19 (0.17 – 0.21) |  | 0.19 (0.15 – 0.24) |  | 0.17 (0.17 – 0.17) |  | 0.19 (0.17 – 0.22) |  |
|  | Occipital | Left | 0.16 (0.14 – 0.19) | **0.0023** | 0.15 (0.12 – 0.16) | **<0.001** | 0.16 (0.15 – 0.19) | **0.0058** | 0.15 (0.14 – 0.18) | **<0.001** | 0.14 (0.14 – 0.14) | 1 | 0.16 (0.14 – 0.19) | **<0.001** |
|  |  | Right | 0.19 (0.17 – 0.23) |  | 0.19 (0.18 – 0.21) |  | 0.19 (0.17 – 0.23) |  | 0.19 (0.18 – 0.22) |  | 0.15 (0.15 – 0.15) |  | 0.19 (0.17 – 0.22) |  |
| MAD of HHbx (au) | Frontal | Left | 0.21 (0.18 – 0.24) | 0.9462 | 0.2 (0.19 – 0.23) | 0.7508 | 0.19 (0.17 – 0.22) | 0.7618 | 0.21 (0.19 – 0.24) | 0.5186 | 0.14 (0.14 – 0.14) | 1 | 0.2 (0.18 – 0.24) | 0.9943 |
|  |  | Right | 0.19 (0.17 – 0.24) |  | 0.21 (0.2 – 0.22) |  | 0.19 (0.16 – 0.22) |  | 0.22 (0.19 – 0.25) |  | 0.16 (0.16 – 0.16) |  | 0.2 (0.18 – 0.23) |  |
|  | Parietal | Left | 0.19 (0.16 – 0.23) | 0.1888 | 0.22 (0.18 – 0.25) | 0.8399 | 0.18 (0.16 – 0.22) | 0.1983 | 0.22 (0.18 – 0.25) | 0.9345 | 0.19 (0.19 – 0.19) | 1 | 0.19 (0.17 – 0.23) | 0.2801 |
|  |  | Right | 0.21 (0.19 – 0.23) |  | 0.21 (0.19 – 0.22) |  | 0.2 (0.18 – 0.22) |  | 0.22 (0.2 – 0.23) |  | 0.16 (0.16 – 0.16) |  | 0.21 (0.19 – 0.23) |  |
|  | Temporal | Left | 0.2 (0.17 – 0.21) | 0.7831 | 0.19 (0.15 – 0.23) | 0.665 | 0.2 (0.16 – 0.21) | 0.4079 | 0.2 (0.16 – 0.23) | 0.8419 | 0.15 (0.15 – 0.15) | 1 | 0.2 (0.16 – 0.22) | 0.6493 |
|  |  | Right | 0.19 (0.16 – 0.24) |  | 0.19 (0.18 – 0.22) |  | 0.2 (0.18 – 0.23) |  | 0.19 (0.16 – 0.24) |  | 0.13 (0.13 – 0.13) |  | 0.19 (0.17 – 0.23) |  |
|  | Occipital | Left | 0.15 (0.14 – 0.18) | **<0.001** | 0.14 (0.13 – 0.16) | **<0.001** | 0.15 (0.14 – 0.17) | **<0.001** | 0.16 (0.13 – 0.17) | **<0.001** | 0.11 (0.11 – 0.11) | 1 | 0.15 (0.14 – 0.17) | **<0.001** |
|  |  | Right | 0.2 (0.18 – 0.23) |  | 0.18 (0.17 – 0.2) |  | 0.19 (0.17 – 0.22) |  | 0.19 (0.18 – 0.23) |  | 0.17 (0.17 – 0.17) |  | 0.19 (0.17 – 0.23) |  |
| MAD of tHbx (au) | Frontal | Left | 0.2 (0.18 – 0.23) | 0.6664 | 0.2 (0.17 – 0.21) | 0.141 | 0.2 (0.17 – 0.22) | 0.8505 | 0.21 (0.2 – 0.22) | 0.8053 | 0.17 (0.17 – 0.17) | 1 | 0.2 (0.18 – 0.22) | 0.8091 |
|  |  | Right | 0.2 (0.17 – 0.24) |  | 0.21 (0.19 – 0.23) |  | 0.2 (0.18 – 0.23) |  | 0.21 (0.18 – 0.25) |  | 0.13 (0.13 – 0.13) |  | 0.21 (0.18 – 0.24) |  |
|  | Parietal | Left | 0.19 (0.17 – 0.23) | 0.403 | 0.21 (0.2 – 0.22) | 0.7508 | 0.18 (0.17 – 0.21) | 0.6057 | 0.22 (0.19 – 0.23) | 0.5035 | 0.15 (0.15 – 0.15) | 1 | 0.2 (0.18 – 0.23) | 0.3631 |
|  |  | Right | 0.21 (0.18 – 0.24) |  | 0.21 (0.19 – 0.23) |  | 0.19 (0.17 – 0.22) |  | 0.23 (0.21 – 0.24) |  | 0.15 (0.15 – 0.15) |  | 0.21 (0.18 – 0.24) |  |
|  | Temporal | Left | 0.2 (0.18 – 0.23) | 0.5926 | 0.19 (0.17 – 0.21) | 0.2145 | 0.2 (0.18 – 0.23) | 0.7995 | 0.2 (0.18 – 0.23) | 0.7692 | 0.14 (0.14 – 0.14) | 1 | 0.2 (0.18 – 0.23) | 0.9434 |
|  |  | Right | 0.2 (0.17 – 0.23) |  | 0.21 (0.17 – 0.23) |  | 0.19 (0.17 – 0.22) |  | 0.21 (0.16 – 0.25) |  | 0.15 (0.15 – 0.15) |  | 0.2 (0.17 – 0.23) |  |
|  | Occipital | Left | 0.18 (0.15 – 0.21) | **0.0478** | 0.15 (0.14 – 0.17) | **0.0017** | 0.16 (0.15 – 0.2) | **0.0113** | 0.17 (0.13 – 0.21) | **0.062** | 0.16 (0.16 – 0.16) | 1 | 0.17 (0.14 – 0.21) | **0.0017** |
|  |  | Right | 0.2 (0.17 – 0.23) |  | 0.19 (0.18 – 0.21) |  | 0.2 (0.17 – 0.23) |  | 0.19 (0.18 – 0.22) |  | 0.15 (0.15 – 0.15) |  | 0.2 (0.18 – 0.22) |  |
| MAD of HbDiffx (au) | Frontal | Left | 0.2 (0.18 – 0.24) | 0.9959 | 0.19 (0.17 – 0.22) | 0.665 | 0.19 (0.17 – 0.22) | 0.5943 | 0.22 (0.19 – 0.23) | 0.8419 | 0.17 (0.17 – 0.17) | 1 | 0.2 (0.18 – 0.23) | 0.8201 |
|  |  | Right | 0.21 (0.17 – 0.23) |  | 0.21 (0.17 – 0.21) |  | 0.2 (0.17 – 0.23) |  | 0.22 (0.18 – 0.24) |  | 0.15 (0.15 – 0.15) |  | 0.21 (0.17 – 0.23) |  |
|  | Parietal | Left | 0.18 (0.16 – 0.22) | 0.2302 | 0.19 (0.17 – 0.2) | 0.1572 | 0.18 (0.15 – 0.2) | 0.0963 | 0.19 (0.18 – 0.23) | 0.5495 | 0.18 (0.18 – 0.18) | 1 | 0.18 (0.16 – 0.21) | 0.0993 |
|  |  | Right | 0.2 (0.17 – 0.23) |  | 0.2 (0.19 – 0.22) |  | 0.2 (0.17 – 0.22) |  | 0.2 (0.18 – 0.25) |  | 0.17 (0.17 – 0.17) |  | 0.2 (0.18 – 0.23) |  |
|  | Temporal | Left | 0.19 (0.16 – 0.22) | 0.6739 | 0.17 (0.16 – 0.2) | 0.5067 | 0.18 (0.15 – 0.21) | 0.3058 | 0.2 (0.17 – 0.22) | 0.7872 | 0.14 (0.14 – 0.14) | 1 | 0.18 (0.16 – 0.22) | 0.5179 |
|  |  | Right | 0.21 (0.16 – 0.22) |  | 0.2 (0.16 – 0.21) |  | 0.21 (0.16 – 0.22) |  | 0.19 (0.16 – 0.22) |  | 0.13 (0.13 – 0.13) |  | 0.2 (0.16 – 0.22) |  |
|  | Occipital | Left | 0.15 (0.13 – 0.17) | **<0.001** | 0.13 (0.12 – 0.15) | **<0.001** | 0.14 (0.13 – 0.18) | **<0.001** | 0.14 (0.12 – 0.16) | **<0.001** | 0.13 (0.13 – 0.13) | 1 | 0.14 (0.13 – 0.17) | **<0.001** |
|  |  | Right | 0.2 (0.17 – 0.22) |  | 0.19 (0.17 – 0.21) |  | 0.2 (0.17 – 0.22) |  | 0.2 (0.17 – 0.22) |  | 0.13 (0.13 – 0.13) |  | 0.2 (0.17 – 0.22) |  |
| **250 Hz Sampled Data** | | | | | | | | | | | | | | |
| COx-a (au) | Frontal | Left | -0.01 (-0.22 – 0.18) | 0.2262 | 0.02 (-0.22 – 0.19) | 0.7075 | -0.01 (-0.2 – 0.17) | 0.4079 | 0.01 (-0.24 – 0.21) | 0.5339 | 0.01 (-0.2 – 0.18) | 1 | -0.01 (-0.23 – 0.18) | 0.2439 |
|  |  | Right | 0.01 (-0.18 – 0.23) |  | -0.02 (-0.23 – 0.19) |  | -0.01 (-0.18 – 0.2) |  | 0.02 (-0.22 – 0.23) |  | -0.08 (-0.21 – 0.12) |  | 0 (-0.19 – 0.21) |  |
|  | Parietal | Left | -0.01 (-0.2 – 0.2) | 0.8152 | 0 (-0.19 – 0.19) | 0.5444 | 0.01 (-0.18 – 0.19) | 0.5069 | -0.02 (-0.21 – 0.2) | 0.9532 | 0.01 (-0.2 – 0.16) | 1 | 0 (-0.19 – 0.2) | 0.7763 |
|  |  | Right | 0 (-0.2 – 0.19) |  | -0.03 (-0.19 – 0.17) |  | -0.02 (-0.2 – 0.18) |  | 0 (-0.2 – 0.21) |  | -0.1 (-0.27 – 0.08) |  | -0.01 (-0.19 – 0.19) |  |
|  | Temporal | Left | -0.01 (-0.2 – 0.19) | 0.6588 | 0.03 (-0.15 – 0.19) | 0.5444 | -0.01 (-0.2 – 0.16) | 0.2348 | 0.03 (-0.16 – 0.2) | 0.2359 | -0.02 (-0.13 – 0.14) | 1 | 0 (-0.2 – 0.19) | 0.9095 |
|  |  | Right | 0.01 (-0.18 – 0.2) |  | -0.01 (-0.2 – 0.19) |  | 0.03 (-0.18 – 0.21) |  | -0.02 (-0.19 – 0.19) |  | 0.11 (0 – 0.26) |  | 0.01 (-0.18 – 0.2) |  |
|  | Occipital | Left | 0 (-0.14 – 0.14) | 0.9379 | 0.03 (-0.13 – 0.17) | 0.8399 | 0 (-0.15 – 0.14) | 0.6405 | 0.02 (-0.12 – 0.15) | 0.8603 | -0.07 (-0.21 – 0.05) | 1 | 0.01 (-0.14 – 0.15) | 0.7817 |
|  |  | Right | -0.01 (-0.2 – 0.2) |  | 0 (-0.16 – 0.17) |  | -0.01 (-0.21 – 0.18) |  | 0 (-0.17 – 0.2) |  | -0.1 (-0.22 – 0.07) |  | 0 (-0.18 – 0.19) |  |
| HbOx (au) | Frontal | Left | -0.01 (-0.21 – 0.19) | 0.0594 | -0.01 (-0.23 – 0.17) | 0.5834 | -0.02 (-0.21 – 0.17) | 0.0728 | 0 (-0.23 – 0.19) | 0.4181 | 0.1 (-0.05 – 0.25) | 1 | -0.01 (-0.22 – 0.18) | **0.0313** |
|  |  | Right | 0.02 (-0.19 – 0.22) |  | -0.01 (-0.22 – 0.21) |  | 0 (-0.18 – 0.22) |  | 0.02 (-0.22 – 0.23) |  | -0.04 (-0.16 – 0.14) |  | 0.01 (-0.2 – 0.22) |  |
|  | Parietal | Left | 0.01 (-0.2 – 0.24) | 0.9876 | -0.03 (-0.23 – 0.18) | 0.2366 | 0.01 (-0.19 – 0.21) | 0.9152 | 0 (-0.21 – 0.24) | 0.5495 | 0.01 (-0.16 – 0.16) | 1 | 0 (-0.2 – 0.22) | 0.5843 |
|  |  | Right | 0.01 (-0.19 – 0.22) |  | 0.01 (-0.18 – 0.22) |  | 0.01 (-0.16 – 0.21) |  | 0.01 (-0.22 – 0.24) |  | 0.01 (-0.2 – 0.15) |  | 0.01 (-0.18 – 0.22) |  |
|  | Temporal | Left | 0 (-0.21 – 0.19) | 0.2144 | 0.02 (-0.19 – 0.16) | 0.5067 | 0 (-0.2 – 0.18) | 0.1338 | 0.03 (-0.2 – 0.16) | 0.7513 | 0 (-0.12 – 0.18) | 1 | 0.01 (-0.2 – 0.18) | 0.5271 |
|  |  | Right | 0.04 (-0.17 – 0.21) |  | -0.05 (-0.24 – 0.18) |  | 0.04 (-0.17 – 0.22) |  | -0.04 (-0.2 – 0.18) |  | 0.13 (-0.05 – 0.3) |  | 0.02 (-0.19 – 0.2) |  |
|  | Occipital | Left | 0.01 (-0.15 – 0.17) | 0.6892 | 0.03 (-0.14 – 0.17) | 0.5834 | 0.02 (-0.16 – 0.19) | 0.3987 | 0.03 (-0.14 – 0.17) | 0.9159 | 0 (-0.15 – 0.12) | 1 | 0.03 (-0.15 – 0.18) | 0.604 |
|  |  | Right | 0.01 (-0.18 – 0.19) |  | 0.01 (-0.19 – 0.21) |  | -0.01 (-0.2 – 0.19) |  | 0.02 (-0.18 – 0.22) |  | -0.04 (-0.2 – 0.12) |  | 0.01 (-0.18 – 0.2) |  |
| HHbx (au) | Frontal | Left | 0.01 (-0.18 – 0.21) | 0.9876 | -0.02 (-0.21 – 0.18) | 0.7075 | 0 (-0.18 – 0.19) | 0.5607 | -0.01 (-0.21 – 0.21) | 0.5812 | 0.08 (-0.06 – 0.23) | 1 | 0 (-0.19 – 0.2) | 0.887 |
|  |  | Right | 0.01 (-0.19 – 0.21) |  | 0.03 (-0.18 – 0.24) |  | 0.02 (-0.15 – 0.21) |  | -0.01 (-0.23 – 0.24) |  | 0.1 (-0.14 – 0.22) |  | 0.02 (-0.19 – 0.24) |  |
|  | Parietal | Left | 0 (-0.2 – 0.22) | 0.6664 | -0.02 (-0.24 – 0.18) | **0.0262** | 0 (-0.2 – 0.2) | 0.3058 | -0.01 (-0.22 – 0.22) | 0.2751 | 0 (-0.2 – 0.18) | 1 | 0 (-0.22 – 0.2) | 0.1887 |
|  |  | Right | 0.01 (-0.18 – 0.2) |  | 0.04 (-0.17 – 0.26) |  | 0.02 (-0.18 – 0.19) |  | 0.02 (-0.17 – 0.26) |  | 0.08 (-0.08 – 0.24) |  | 0.02 (-0.17 – 0.22) |  |
|  | Temporal | Left | 0.01 (-0.17 – 0.21) | 0.6968 | -0.04 (-0.21 – 0.14) | 0.6236 | 0.02 (-0.16 – 0.22) | 0.7369 | -0.02 (-0.21 – 0.18) | 0.1927 | 0 (-0.11 – 0.16) | 1 | 0 (-0.19 – 0.2) | 0.473 |
|  |  | Right | 0.01 (-0.19 – 0.21) |  | -0.03 (-0.23 – 0.17) |  | 0.01 (-0.19 – 0.23) |  | 0 (-0.2 – 0.19) |  | 0.01 (-0.11 – 0.17) |  | 0.01 (-0.2 – 0.21) |  |
|  | Occipital | Left | 0.01 (-0.14 – 0.16) | 0.8476 | 0.02 (-0.16 – 0.14) | 0.6236 | 0.03 (-0.14 – 0.18) | 0.3377 | -0.01 (-0.16 – 0.15) | 0.2962 | 0.1 (0.01 – 0.24) | 1 | 0.01 (-0.14 – 0.16) | 0.9717 |
|  |  | Right | 0.02 (-0.17 – 0.22) |  | -0.01 (-0.19 – 0.18) |  | 0.02 (-0.15 – 0.22) |  | 0 (-0.18 – 0.21) |  | 0.05 (-0.15 – 0.19) |  | 0 (-0.18 – 0.22) |  |
| tHbx (au) | Frontal | Left | -0.01 (-0.22 – 0.19) | 0.3914 | -0.02 (-0.23 – 0.19) | 0.8399 | -0.01 (-0.22 – 0.19) | 0.1712 | 0.01 (-0.23 – 0.19) | 0.8973 | 0.1 (-0.07 – 0.27) | 1 | -0.01 (-0.22 – 0.19) | 0.2677 |
|  |  | Right | 0.02 (-0.17 – 0.22) |  | -0.02 (-0.24 – 0.23) |  | 0.01 (-0.16 – 0.23) |  | -0.01 (-0.21 – 0.2) |  | -0.02 (-0.13 – 0.14) |  | 0.01 (-0.18 – 0.23) |  |
|  | Parietal | Left | 0 (-0.19 – 0.23) | 0.7911 | -0.03 (-0.23 – 0.19) | **0.0086** | 0.01 (-0.18 – 0.21) | 0.7493 | -0.02 (-0.23 – 0.21) | 0.0933 | -0.01 (-0.2 – 0.12) | 1 | 0 (-0.21 – 0.21) | 0.2085 |
|  |  | Right | 0 (-0.18 – 0.22) |  | 0.04 (-0.2 – 0.25) |  | 0.01 (-0.17 – 0.22) |  | 0 (-0.2 – 0.25) |  | 0.08 (-0.13 – 0.21) |  | 0 (-0.18 – 0.24) |  |
|  | Temporal | Left | 0.02 (-0.17 – 0.2) | 0.4832 | -0.02 (-0.21 – 0.15) | 0.2855 | 0.01 (-0.19 – 0.19) | 0.4659 | 0 (-0.2 – 0.18) | 0.8603 | 0.02 (-0.12 – 0.19) | 1 | 0 (-0.2 – 0.19) | 0.8036 |
|  |  | Right | 0.03 (-0.18 – 0.22) |  | -0.05 (-0.25 – 0.18) |  | 0.03 (-0.18 – 0.22) |  | -0.02 (-0.23 – 0.2) |  | 0.08 (-0.09 – 0.23) |  | 0.01 (-0.2 – 0.21) |  |
|  | Occipital | Left | 0.01 (-0.14 – 0.2) | 0.7991 | 0.02 (-0.15 – 0.17) | 0.3708 | 0.02 (-0.14 – 0.2) | 0.6172 | 0.01 (-0.17 – 0.17) | 0.8603 | 0.06 (-0.09 – 0.25) | 1 | 0.01 (-0.15 – 0.18) | 0.9208 |
|  |  | Right | 0.01 (-0.18 – 0.21) |  | 0 (-0.19 – 0.22) |  | 0 (-0.19 – 0.21) |  | 0 (-0.19 – 0.22) |  | 0.02 (-0.18 – 0.15) |  | 0 (-0.19 – 0.22) |  |
| HbDiffx (au) | Frontal | Left | -0.01 (-0.2 – 0.18) | 0.1018 | 0.01 (-0.23 – 0.18) | 0.8852 | -0.01 (-0.19 – 0.16) | 0.2041 | 0.01 (-0.24 – 0.19) | 0.4455 | 0.03 (-0.15 – 0.19) | 1 | -0.01 (-0.23 – 0.18) | 0.1022 |
|  |  | Right | 0.02 (-0.18 – 0.24) |  | 0 (-0.24 – 0.17) |  | 0 (-0.18 – 0.22) |  | 0.02 (-0.2 – 0.24) |  | -0.06 (-0.19 – 0.13) |  | 0.01 (-0.18 – 0.23) |  |
|  | Parietal | Left | 0.01 (-0.19 – 0.21) | 0.5365 | 0 (-0.19 – 0.19) | 0.931 | 0.01 (-0.19 – 0.2) | 0.3377 | 0 (-0.2 – 0.19) | 0.7872 | 0.01 (-0.21 – 0.16) | 1 | 0.01 (-0.19 – 0.2) | 0.6803 |
|  |  | Right | -0.01 (-0.2 – 0.19) |  | -0.02 (-0.2 – 0.2) |  | -0.01 (-0.2 – 0.19) |  | 0.02 (-0.21 – 0.22) |  | -0.12 (-0.27 – 0.07) |  | -0.01 (-0.2 – 0.19) |  |
|  | Temporal | Left | 0 (-0.19 – 0.19) | 0.9131 | 0.02 (-0.16 – 0.18) | 0.4705 | 0 (-0.19 – 0.18) | 0.5175 | 0.02 (-0.16 – 0.21) | 0.1848 | -0.02 (-0.12 – 0.15) | 1 | 0.01 (-0.19 – 0.19) | 0.5649 |
|  |  | Right | 0.02 (-0.18 – 0.21) |  | -0.04 (-0.22 – 0.19) |  | 0.03 (-0.18 – 0.22) |  | 0 (-0.19 – 0.19) |  | 0.14 (0.02 – 0.28) |  | 0.01 (-0.19 – 0.2) |  |
|  | Occipital | Left | 0.01 (-0.15 – 0.17) | 0.72 | 0.02 (-0.13 – 0.16) | 0.8399 | 0 (-0.15 – 0.17) | 0.8634 | 0.03 (-0.12 – 0.16) | 0.9532 | -0.05 (-0.17 – 0.06) | 1 | 0.01 (-0.14 – 0.17) | 0.9717 |
|  |  | Right | -0.01 (-0.19 – 0.21) |  | 0.01 (-0.17 – 0.19) |  | -0.01 (-0.2 – 0.19) |  | 0.01 (-0.17 – 0.22) |  | -0.05 (-0.19 – 0.06) |  | 0 (-0.19 – 0.2) |  |
| MAD of COx-a (au) | Frontal | Left | 0.21 (0.18 – 0.24) | 0.8721 | 0.2 (0.17 – 0.22) | 0.5834 | 0.19 (0.18 – 0.22) | 0.9152 | 0.22 (0.19 – 0.23) | 0.8603 | 0.19 (0.19 – 0.19) | 1 | 0.21 (0.18 – 0.23) | 0.859 |
|  |  | Right | 0.2 (0.18 – 0.23) |  | 0.21 (0.2 – 0.22) |  | 0.2 (0.17 – 0.23) |  | 0.22 (0.19 – 0.25) |  | 0.16 (0.16 – 0.16) |  | 0.21 (0.18 – 0.23) |  |
|  | Parietal | Left | 0.19 (0.16 – 0.22) | 0.2425 | 0.19 (0.16 – 0.2) | 0.069 | 0.18 (0.15 – 0.2) | 0.0838 | 0.2 (0.17 – 0.23) | 0.4317 | 0.17 (0.17 – 0.17) | 1 | 0.19 (0.16 – 0.21) | 0.0722 |
|  |  | Right | 0.2 (0.17 – 0.24) |  | 0.21 (0.19 – 0.21) |  | 0.2 (0.17 – 0.22) |  | 0.21 (0.18 – 0.24) |  | 0.17 (0.17 – 0.17) |  | 0.21 (0.17 – 0.23) |  |
|  | Temporal | Left | 0.2 (0.16 – 0.22) | 0.9876 | 0.17 (0.14 – 0.24) | 0.7508 | 0.18 (0.16 – 0.22) | 0.3296 | 0.21 (0.16 – 0.24) | 0.33 | 0.13 (0.13 – 0.13) | 1 | 0.2 (0.16 – 0.23) | 0.983 |
|  |  | Right | 0.2 (0.17 – 0.23) |  | 0.19 (0.16 – 0.22) |  | 0.2 (0.17 – 0.23) |  | 0.19 (0.16 – 0.22) |  | 0.13 (0.13 – 0.13) |  | 0.2 (0.17 – 0.22) |  |
|  | Occipital | Left | 0.14 (0.13 – 0.16) | **<0.001** | 0.14 (0.12 – 0.15) | **<0.001** | 0.14 (0.13 – 0.17) | **<0.001** | 0.14 (0.12 – 0.15) | **<0.001** | 0.13 (0.13 – 0.13) | 1 | 0.14 (0.12 – 0.16) | **<0.001** |
|  |  | Right | 0.2 (0.17 – 0.22) |  | 0.19 (0.16 – 0.22) |  | 0.2 (0.18 – 0.22) |  | 0.2 (0.17 – 0.22) |  | 0.14 (0.14 – 0.14) |  | 0.2 (0.17 – 0.22) |  |
| MAD of HbOx (au) | Frontal | Left | 0.19 (0.17 – 0.22) | 0.1592 | 0.19 (0.18 – 0.21) | 0.1939 | 0.19 (0.16 – 0.21) | 0.1871 | 0.21 (0.18 – 0.22) | 0.098 | 0.15 (0.15 – 0.15) | 1 | 0.19 (0.17 – 0.22) | **0.0459** |
|  |  | Right | 0.21 (0.18 – 0.24) |  | 0.22 (0.19 – 0.24) |  | 0.2 (0.17 – 0.23) |  | 0.24 (0.19 – 0.26) |  | 0.15 (0.15 – 0.15) |  | 0.21 (0.19 – 0.24) |  |
|  | Parietal | Left | 0.2 (0.18 – 0.22) | 0.4897 | 0.21 (0.19 – 0.22) | 0.3123 | 0.19 (0.16 – 0.21) | 0.7743 | 0.22 (0.2 – 0.24) | 0.8603 | 0.16 (0.16 – 0.16) | 1 | 0.21 (0.18 – 0.22) | 0.7492 |
|  |  | Right | 0.2 (0.18 – 0.25) |  | 0.21 (0.18 – 0.21) |  | 0.19 (0.17 – 0.23) |  | 0.22 (0.18 – 0.26) |  | 0.17 (0.17 – 0.17) |  | 0.21 (0.18 – 0.24) |  |
|  | Temporal | Left | 0.2 (0.17 – 0.23) | 0.2342 | 0.18 (0.15 – 0.2) | 0.5834 | 0.19 (0.17 – 0.23) | 0.7618 | 0.19 (0.16 – 0.23) | 0.5974 | 0.14 (0.14 – 0.14) | 1 | 0.19 (0.17 – 0.23) | 0.4429 |
|  |  | Right | 0.2 (0.16 – 0.21) |  | 0.19 (0.16 – 0.22) |  | 0.2 (0.17 – 0.21) |  | 0.18 (0.16 – 0.23) |  | 0.18 (0.18 – 0.18) |  | 0.2 (0.16 – 0.22) |  |
|  | Occipital | Left | 0.16 (0.14 – 0.19) | **0.0013** | 0.16 (0.12 – 0.17) | **0.0017** | 0.17 (0.15 – 0.19) | **0.0156** | 0.14 (0.12 – 0.17) | **<0.001** | 0.14 (0.14 – 0.14) | 1 | 0.16 (0.13 – 0.19) | **<0.001** |
|  |  | Right | 0.2 (0.17 – 0.23) |  | 0.19 (0.18 – 0.2) |  | 0.2 (0.17 – 0.23) |  | 0.19 (0.18 – 0.22) |  | 0.16 (0.16 – 0.16) |  | 0.19 (0.17 – 0.22) |  |
| MAD of HHbx (au) | Frontal | Left | 0.21 (0.18 – 0.23) | 0.7911 | 0.2 (0.19 – 0.22) | 0.5444 | 0.19 (0.16 – 0.22) | 0.9022 | 0.21 (0.19 – 0.23) | 0.2962 | 0.14 (0.14 – 0.14) | 1 | 0.21 (0.18 – 0.23) | 0.7438 |
|  |  | Right | 0.19 (0.17 – 0.25) |  | 0.21 (0.19 – 0.23) |  | 0.19 (0.16 – 0.23) |  | 0.23 (0.19 – 0.26) |  | 0.15 (0.15 – 0.15) |  | 0.2 (0.18 – 0.24) |  |
|  | Parietal | Left | 0.19 (0.16 – 0.23) | 0.1562 | 0.22 (0.18 – 0.25) | 0.931 | 0.19 (0.15 – 0.23) | 0.2284 | 0.23 (0.18 – 0.24) | 0.7336 | 0.19 (0.19 – 0.19) | 1 | 0.19 (0.16 – 0.23) | 0.2326 |
|  |  | Right | 0.21 (0.19 – 0.23) |  | 0.21 (0.19 – 0.23) |  | 0.2 (0.17 – 0.22) |  | 0.22 (0.2 – 0.24) |  | 0.16 (0.16 – 0.16) |  | 0.21 (0.19 – 0.23) |  |
|  | Temporal | Left | 0.2 (0.17 – 0.22) | 0.8071 | 0.19 (0.16 – 0.23) | 0.5067 | 0.2 (0.17 – 0.21) | 0.3215 | 0.19 (0.16 – 0.24) | 0.8973 | 0.13 (0.13 – 0.13) | 1 | 0.2 (0.17 – 0.22) | 0.5601 |
|  |  | Right | 0.19 (0.17 – 0.24) |  | 0.19 (0.18 – 0.23) |  | 0.21 (0.17 – 0.23) |  | 0.19 (0.16 – 0.23) |  | 0.14 (0.14 – 0.14) |  | 0.19 (0.17 – 0.23) |  |
|  | Occipital | Left | 0.15 (0.13 – 0.17) | **<0.001** | 0.14 (0.12 – 0.16) | **0.0014** | 0.15 (0.13 – 0.17) | **<0.001** | 0.14 (0.13 – 0.16) | **<0.001** | 0.11 (0.11 – 0.11) | 1 | 0.15 (0.13 – 0.17) | **<0.001** |
|  |  | Right | 0.19 (0.17 – 0.23) |  | 0.18 (0.17 – 0.2) |  | 0.19 (0.17 – 0.22) |  | 0.19 (0.17 – 0.23) |  | 0.16 (0.16 – 0.16) |  | 0.19 (0.17 – 0.23) |  |
| MAD of tHbx (au) | Frontal | Left | 0.2 (0.17 – 0.23) | 0.7911 | 0.19 (0.17 – 0.21) | 0.3123 | 0.19 (0.17 – 0.21) | 0.676 | 0.21 (0.19 – 0.23) | 0.6812 | 0.17 (0.17 – 0.17) | 1 | 0.2 (0.17 – 0.22) | 0.7012 |
|  |  | Right | 0.2 (0.17 – 0.24) |  | 0.21 (0.19 – 0.23) |  | 0.2 (0.18 – 0.22) |  | 0.22 (0.18 – 0.25) |  | 0.13 (0.13 – 0.13) |  | 0.2 (0.18 – 0.24) |  |
|  | Parietal | Left | 0.19 (0.17 – 0.23) | 0.4328 | 0.21 (0.19 – 0.23) | 0.977 | 0.18 (0.16 – 0.21) | 0.5389 | 0.22 (0.19 – 0.24) | 0.6641 | 0.15 (0.15 – 0.15) | 1 | 0.2 (0.17 – 0.23) | 0.4643 |
|  |  | Right | 0.22 (0.18 – 0.24) |  | 0.21 (0.18 – 0.23) |  | 0.2 (0.17 – 0.23) |  | 0.22 (0.21 – 0.24) |  | 0.16 (0.16 – 0.16) |  | 0.22 (0.18 – 0.24) |  |
|  | Temporal | Left | 0.2 (0.18 – 0.23) | 0.7435 | 0.19 (0.18 – 0.21) | 0.2602 | 0.2 (0.18 – 0.23) | 0.9152 | 0.2 (0.18 – 0.23) | 0.7872 | 0.16 (0.16 – 0.16) | 1 | 0.2 (0.18 – 0.23) | 0.8758 |
|  |  | Right | 0.19 (0.17 – 0.23) |  | 0.22 (0.17 – 0.23) |  | 0.19 (0.17 – 0.22) |  | 0.21 (0.17 – 0.24) |  | 0.16 (0.16 – 0.16) |  | 0.2 (0.17 – 0.23) |  |
|  | Occipital | Left | 0.18 (0.15 – 0.21) | 0.0856 | 0.16 (0.14 – 0.18) | **0.0073** | 0.17 (0.15 – 0.2) | 0.0606 | 0.17 (0.13 – 0.21) | 0.0557 | 0.16 (0.16 – 0.16) | 1 | 0.17 (0.14 – 0.2) | **0.0053** |
|  |  | Right | 0.2 (0.17 – 0.23) |  | 0.2 (0.17 – 0.21) |  | 0.19 (0.17 – 0.23) |  | 0.2 (0.18 – 0.22) |  | 0.16 (0.16 – 0.16) |  | 0.2 (0.17 – 0.22) |  |
| MAD of HbDiffx (au) | Frontal | Left | 0.2 (0.18 – 0.23) | 0.7356 | 0.19 (0.17 – 0.22) | 0.6236 | 0.19 (0.17 – 0.22) | 0.5069 | 0.22 (0.19 – 0.23) | 0.9532 | 0.17 (0.17 – 0.17) | 1 | 0.2 (0.18 – 0.23) | 0.5459 |
|  |  | Right | 0.21 (0.17 – 0.23) |  | 0.2 (0.17 – 0.22) |  | 0.2 (0.17 – 0.23) |  | 0.22 (0.19 – 0.24) |  | 0.16 (0.16 – 0.16) |  | 0.21 (0.17 – 0.23) |  |
|  | Parietal | Left | 0.18 (0.16 – 0.23) | 0.2032 | 0.18 (0.17 – 0.2) | 0.069 | 0.17 (0.16 – 0.2) | 0.1101 | 0.19 (0.17 – 0.24) | 0.4181 | 0.17 (0.17 – 0.17) | 1 | 0.18 (0.16 – 0.21) | 0.0711 |
|  |  | Right | 0.2 (0.17 – 0.23) |  | 0.21 (0.19 – 0.21) |  | 0.2 (0.17 – 0.22) |  | 0.21 (0.18 – 0.25) |  | 0.17 (0.17 – 0.17) |  | 0.2 (0.18 – 0.22) |  |
|  | Temporal | Left | 0.19 (0.16 – 0.22) | 0.4703 | 0.18 (0.15 – 0.2) | 0.5067 | 0.18 (0.15 – 0.2) | 0.1176 | 0.19 (0.16 – 0.22) | 0.7513 | 0.13 (0.13 – 0.13) | 1 | 0.19 (0.16 – 0.22) | 0.3268 |
|  |  | Right | 0.21 (0.16 – 0.22) |  | 0.2 (0.17 – 0.22) |  | 0.21 (0.17 – 0.22) |  | 0.2 (0.16 – 0.22) |  | 0.13 (0.13 – 0.13) |  | 0.21 (0.17 – 0.22) |  |
|  | Occipital | Left | 0.15 (0.13 – 0.18) | **<0.001** | 0.15 (0.12 – 0.16) | **0.0024** | 0.16 (0.13 – 0.18) | **<0.001** | 0.14 (0.12 – 0.16) | **<0.001** | 0.12 (0.12 – 0.12) | 1 | 0.15 (0.13 – 0.18) | **<0.001** |
|  |  | Right | 0.2 (0.17 – 0.22) |  | 0.2 (0.16 – 0.21) |  | 0.2 (0.18 – 0.22) |  | 0.2 (0.16 – 0.22) |  | 0.13 (0.13 – 0.13) |  | 0.2 (0.17 – 0.22) |  |
| The p-values in the table are derived using Mann-Whitney U test between the subgrouped bilateral CVR signals. *COx-a, cerebral oximetry index with arterial blood pressure; CVR, cerebrovascular reactivity index; HbDiffx, hemoglobin difference index; HbOx, oxyhemoglobin index; HHbx, deoxyhemoglobin index; IQR, interquartile range; MAD, median absolute deviation; tHbx, total hemoglobin index.* | | | | | | | | | | | | | | |

Appendix S6c: Subgrouped Physiologic Results Using 10-Second Decimated Data

| **Physiologic Signal** | **Brain Lobe** | **Hemisphere** | **Subgroups** | | | | | | | | | | | |
| --- | --- | --- | --- | --- | --- | --- | --- | --- | --- | --- | --- | --- | --- | --- |
|  |  |  | **Age < 40 [n=38]** | | **Age 40 – 60 [n=12]** | | **Males [n=28]** | | **Females [n=22]** | | **Left Hand Dominance [n=1]** | | **Right Hand Dominance [n=49]** | |
|  |  |  | **Median (IQR)** | **p-value** | **Median (IQR)** | **p-value** | **Median (IQR)** | **p-value** | **Median (IQR)** | **p-value** | **Median (IQR)** | **p-value** | **Median (IQR)** | **p-value** |
| **1 Hz Sampled Data** | | | | | | | | | | | | | | |
| ABP (mmHg) | – | – | 95.07 (89.75 – 101.06) | – | 95.63 (89.21 – 102.58) | – | 93.53 (89.3 – 99.4) | – | 95.83 (90 – 103.31) | – | 82.15 (78.69 – 86.4) | – | 95.32 (89.57 – 101.61) | – |
| EtCO₂ (mmHg) | – | – | 35.44 (34.41 – 36.91) | – | 35.69 (33.27 – 36.94) | – | 36.32 (34.38 – 37.64) | – | 34.86 (33.5 – 35.92) | – | 30.36 (23.89 – 33.92) | – | 35.57 (34.32 – 36.99) | – |
| RR (bpm) | – | – | 18.17 (14.97 – 20.6) | – | 16.31 (13.45 – 18.56) | – | 17.46 (14.38 – 20) | – | 18.52 (14.7 – 21.24) | – | 14.01 (11 – 18) | – | 17.82 (14.48 – 20) | – |
| rSO_2_ (%) | Frontal | Left | 46.3 (45.67 – 47.09) | **<0.001** | 44.32 (43.86 – 44.71) | **<0.001** | 45.25 (44.59 – 46.42) | **<0.001** | 45.26 (44.62 – 45.95) | **<0.001** | 42.92 (42.51 – 43.85) | 1 | 45.33 (44.66 – 46.12) | **<0.001** |
|  |  | Right | 40.88 (39.86 – 41.55) |  | 39.39 (38.96 – 40.3) |  | 40.88 (40.24 – 42.03) |  | 38.92 (37.59 – 40.14) |  | 37.35 (35.93 – 44.09) |  | 40.77 (39.69 – 41.19) |  |
|  | Parietal | Left | 50.08 (48.01 – 50.88) | **0.0214** | 47.86 (45.99 – 49.24) | **0.0051** | 50.66 (48.2 – 51.36) | **0.0055** | 47.99 (45.99 – 49.17) | 0.1625 | 53.53 (50.63 – 55.68) | 1 | 48.54 (47.24 – 50.51) | **0.0026** |
|  |  | Right | 45.69 (44.44 – 47.18) |  | 45.25 (43.25 – 46.95) |  | 45.24 (44.18 – 46.53) |  | 46.04 (44.59 – 47.72) |  | 44.72 (42.05 – 45.31) |  | 45.56 (44.19 – 47.29) |  |
|  | Temporal | Left | 40.52 (38.7 – 41.89) | **<0.001** | 40.89 (39.16 – 42.74) | **0.0061** | 39.71 (38.41 – 41.35) | **<0.001** | 41.91 (39.57 – 43.31) | 0.098 | 38.29 (37.51 – 41.14) | 1 | 40.83 (38.8 – 42.18) | **<0.001** |
|  |  | Right | 48.74 (47.62 – 49.63) |  | 45.88 (45.1 – 46.68) |  | 48.78 (47.38 – 49.55) |  | 46.18 (45.19 – 47.47) |  | 46.69 (46.09 – 48.84) |  | 48.1 (46.95 – 49.03) |  |
|  | Occipital | Left | 36.04 (35.72 – 36.43) | **<0.001** | 35.43 (34.91 – 36.03) | **<0.001** | 36.04 (35.69 – 36.36) | **<0.001** | 35.8 (35.29 – 36.48) | **<0.001** | 36.41 (35.98 – 36.95) | 1 | 35.85 (35.49 – 36.34) | **<0.001** |
|  |  | Right | 46.83 (46.24 – 48.46) |  | 45.52 (44.69 – 47.31) |  | 46.89 (46.54 – 47.82) |  | 46 (45.14 – 48.32) |  | 45.82 (45.42 – 46.52) |  | 46.77 (46.14 – 48.19) |  |
| HbO (au) | Frontal | Left | -117.37 (-127.45 – -113.44) | 0.0765 | -126.62 (-128.35 – -119.95) | 0.5444 | -130.06 (-139.95 – -122.93) | 0.5718 | -107.58 (-114.04 – -99.4) | **0.05** | -137.58 (-140.06 – -131.61) | 1 | -119.12 (-125.28 – -114.12) | 0.0895 |
|  |  | Right | -127.87 (-136.29 – -119.59) |  | -129.41 (-133.64 – -127.81) |  | -131.86 (-141.73 – -126.66) |  | -126.86 (-132.54 – -120.78) |  | -523.78 (-533.56 – -168.74) |  | -125.66 (-135.95 – -121.73) |  |
|  | Parietal | Left | -173.14 (-192.99 – -158.57) | 0.2731 | -161.86 (-181.47 – -145.66) | 0.2366 | -171.98 (-184.52 – -160.92) | 0.21 | -176.4 (-189.94 – -145.49) | 0.5974 | -154.68 (-158.68 – -149.08) | 1 | -173.54 (-190.5 – -159.57) | 0.1935 |
|  |  | Right | -184.97 (-204.73 – -170.28) |  | -186.11 (-219.09 – -174.02) |  | -191.19 (-207.81 – -173.92) |  | -177.4 (-206.67 – -168.53) |  | -231.48 (-248.18 – -224.46) |  | -182.43 (-205.9 – -170.28) |  |
|  | Temporal | Left | -203.9 (-209.03 – -195.32) | 0.0622 | -245.13 (-287.41 – -228.11) | 0.1749 | -198.68 (-206.5 – -186.28) | **0.0021** | -224.98 (-246.59 – -222.18) | 0.9345 | -447.67 (-456.5 – -402.9) | 1 | -207.41 (-223.65 – -200.45) | **0.0281** |
|  |  | Right | -152.34 (-161.71 – -139.06) |  | -168.44 (-184.59 – -145.83) |  | -145.31 (-157.59 – -133.86) |  | -229.19 (-260.94 – -212.9) |  | -183.57 (-242.89 – -145.12) |  | -154.75 (-165.56 – -139.94) |  |
|  | Occipital | Left | -496.47 (-505.32 – -491.41) | **<0.001** | -495.44 (-506.44 – -474.86) | **<0.001** | -504.37 (-508.78 – -497.68) | **<0.001** | -487.24 (-504.45 – -464.32) | **<0.001** | -465.69 (-484.43 – -444.84) | 1 | -495.49 (-506.23 – -486.38) | **<0.001** |
|  |  | Right | -181.56 (-206.1 – -159.53) |  | -207.72 (-221.8 – -187.28) |  | -171.18 (-187.29 – -159.53) |  | -213 (-235.31 – -198.57) |  | -245.42 (-256.34 – -236.92) |  | -186.95 (-206.57 – -167.41) |  |
| HHb (au) | Frontal | Left | -112.48 (-116.75 – -110.26) | 0.2384 | -97.34 (-102.78 – -91.75) | 0.1572 | -114.43 (-117.44 – -112.58) | 0.1215 | -103.97 (-110.04 – -95.51) | 0.3787 | -102.12 (-103.83 – -99.98) | 1 | -110.14 (-111.61 – -107.9) | 0.0524 |
|  |  | Right | -86.51 (-94.48 – -78.42) |  | -81.98 (-91.33 – -77.06) |  | -87.85 (-100.64 – -81.68) |  | -80.74 (-88.55 – -72.46) |  | -292.94 (-300.01 – -131.87) |  | -84.49 (-92.68 – -77.49) |  |
|  | Parietal | Left | -169.5 (-191.15 – -157.24) | 0.6144 | -172.52 (-204.66 – -158.75) | 0.8852 | -172.11 (-191.15 – -162.11) | 0.5498 | -159.33 (-199.52 – -143.72) | 0.9719 | -164.87 (-193.1 – -160.54) | 1 | -169.91 (-198.25 – -156.6) | 0.7171 |
|  |  | Right | -163.27 (-183.32 – -147.32) |  | -168.37 (-204.65 – -159.41) |  | -160.33 (-179.35 – -147.61) |  | -166.93 (-191.52 – -152.29) |  | -182.32 (-185.02 – -167.57) |  | -158.36 (-183.39 – -145.49) |  |
|  | Temporal | Left | -135.09 (-151.16 – -133.09) | 0.3914 | -163.05 (-235.97 – -157.37) | 1 | -134.95 (-145.88 – -131.2) | 1 | -152.03 (-169.56 – -141.94) | 0.2092 | -275.09 (-279.29 – -270.05) | 1 | -138.76 (-160.8 – -133.78) | 0.422 |
|  |  | Right | -145.37 (-154.72 – -130.57) |  | -163.54 (-167.84 – -146.28) |  | -134.93 (-142.64 – -128.85) |  | -243.96 (-261.33 – -238.83) |  | -147.44 (-231.62 – -123.87) |  | -149.05 (-157.09 – -132.52) |  |
|  | Occipital | Left | -270.59 (-277.9 – -267.76) | **<0.001** | -268.59 (-279.72 – -259.88) | **0.002** | -277.73 (-281.05 – -274.54) | **<0.001** | -261.07 (-277.13 – -258.42) | **<0.001** | -263.46 (-276.19 – -258.77) | 1 | -269.45 (-279.6 – -267.16) | **<0.001** |
|  |  | Right | -163.22 (-181.63 – -150.7) |  | -192.79 (-207.91 – -176.01) |  | -157.72 (-168.63 – -144.09) |  | -199.43 (-237.34 – -178.97) |  | -211.25 (-223.07 – -200.59) |  | -163.83 (-183.72 – -154.11) |  |
| tHb (au) | Frontal | Left | -225.29 (-243.29 – -213.04) | 0.9793 | -226.5 (-240.7 – -215.04) | 0.4705 | -242.57 (-246.07 – -234.31) | 0.476 | -207.25 (-227.24 – -196.47) | 0.7336 | -240.23 (-243.45 – -231.03) | 1 | -226.28 (-241.35 – -210.52) | 0.6089 |
|  |  | Right | -215.44 (-231.77 – -198.82) |  | -213.26 (-223.37 – -204.58) |  | -221.86 (-242.78 – -205.77) |  | -211.98 (-221.5 – -197.63) |  | -826.82 (-834.61 – -301.45) |  | -214.61 (-228.64 – -196.43) |  |
|  | Parietal | Left | -340.49 (-375.35 – -318.28) | 0.7514 | -333.88 (-385.64 – -304.53) | 0.5067 | -344.21 (-375.35 – -322.9) | 0.7246 | -333.72 (-398.28 – -282.8) | 0.8235 | -322.95 (-348.71 – -304.8) | 1 | -342.53 (-385.27 – -321.32) | 0.624 |
|  |  | Right | -346.95 (-369.96 – -321.65) |  | -353.14 (-415.93 – -341.03) |  | -341.83 (-384.42 – -323.38) |  | -354.87 (-388.74 – -325.42) |  | -407.16 (-417.18 – -400.47) |  | -344.02 (-379.35 – -318.86) |  |
|  | Temporal | Left | -338.99 (-359.15 – -334.06) | 0.439 | -401.96 (-524.81 – -379.42) | 0.4705 | -332.04 (-350.78 – -318.34) | 0.0996 | -383.12 (-423.07 – -366.44) | 0.5812 | -724.63 (-730.86 – -682.54) | 1 | -355.73 (-369.53 – -335.53) | 0.3557 |
|  |  | Right | -304.55 (-319.22 – -270.59) |  | -329.42 (-343.21 – -310.18) |  | -279.16 (-295.33 – -263.29) |  | -509.3 (-541.85 – -467.53) |  | -330.04 (-475.12 – -269.18) |  | -305.75 (-321 – -273.93) |  |
|  | Occipital | Left | -763.32 (-794.15 – -755.66) | **<0.001** | -764.81 (-786.46 – -731.48) | **<0.001** | -792.88 (-798.59 – -779.64) | **<0.001** | -745.39 (-779.6 – -717.33) | **<0.001** | -724.25 (-762.6 – -704.54) | 1 | -765.78 (-793.45 – -754.59) | **<0.001** |
|  |  | Right | -353.21 (-390.51 – -318.14) |  | -399.03 (-426.67 – -363.6) |  | -327.2 (-357.21 – -302.78) |  | -409.62 (-488.06 – -379.19) |  | -457.45 (-476.09 – -437.67) |  | -353.37 (-391.45 – -333.3) |  |
| HbDiff (au) | Frontal | Left | -14.01 (-17.17 – -11.68) | **<0.001** | -26.6 (-29.24 – -22.83) | **<0.001** | -20.49 (-23.47 – -14.01) | **<0.001** | -17.48 (-20.27 – -15.53) | **<0.001** | -33.93 (-36.11 – -28.84) | 1 | -18.7 (-21.54 – -12.86) | **<0.001** |
|  |  | Right | -41.89 (-45.59 – -35.8) |  | -46.78 (-50.98 – -42.69) |  | -41.89 (-45.36 – -35.8) |  | -47.21 (-50.52 – -42.69) |  | -210.84 (-233.83 – -35.5) |  | -44.01 (-48.37 – -38.49) |  |
|  | Parietal | Left | 1.14 (-14.69 – 4.25) | **0.0197** | -12.27 (-23.04 – -4.78) | **0.0102** | 3.09 (-10.13 – 7.99) | **0.0052** | -12.71 (-23.04 – -6.05) | 0.2008 | 21.94 (4.06 – 37.66) | 1 | -9.68 (-18.92 – 2.34) | **0.0035** |
|  |  | Right | -24.54 (-33.35 – -18.57) |  | -32.37 (-39.32 – -23.81) |  | -32.9 (-40.79 – -24.93) |  | -24.54 (-32.93 – -10.77) |  | -43.28 (-65.48 – -38.19) |  | -29.28 (-35.6 – -18.89) |  |
|  | Temporal | Left | -56.01 (-63.38 – -50.25) | **<0.001** | -68.02 (-83.88 – -56.37) | **0.0166** | -61.89 (-71.94 – -53.36) | **<0.001** | -52.04 (-65.99 – -48.63) | 0.13 | -169.76 (-182.55 – -121.91) | 1 | -58.05 (-71.69 – -48.69) | **<0.001** |
|  |  | Right | -6.18 (-13.79 – -2.03) |  | -22.72 (-26.22 – -17.17) |  | -6.18 (-13.79 – -2.16) |  | -20.39 (-26.93 – -12.42) |  | -19.65 (-21.64 – -10.86) |  | -7.91 (-17.24 – -5.4) |  |
|  | Occipital | Left | -219.81 (-226.11 – -212.04) | **<0.001** | -221.1 (-229.76 – -212.01) | **<0.001** | -221.48 (-228.46 – -214.1) | **<0.001** | -218.61 (-226.94 – -208.08) | **<0.001** | -201.46 (-209.72 – -185.62) | 1 | -220.34 (-228.38 – -212.54) | **<0.001** |
|  |  | Right | -20.1 (-23.23 – -9.01) |  | -28.8 (-33.12 – -18.35) |  | -18.85 (-22.73 – -13.09) |  | -32.12 (-37.08 – -9.45) |  | -37.64 (-40.74 – -31.08) |  | -20.63 (-23.24 – -11.32) |  |
| MAD of ABP (mmHg) | – | – | 5.61 (4.94 – 6.48) | – | 5.56 (4.74 – 6.85) | – | 5.29 (4.77 – 6.28) | – | 6.16 (5.11 – 6.99) | – | 3.65 (3.65 – 3.65) | – | 5.64 (4.93 – 6.78) | – |
| MAD of EtCO₂ (mmHg) | – | – | 1.18 (1 – 1.44) | – | 1.45 (1.14 – 1.67) | – | 1.38 (1 – 1.72) | – | 1.13 (1 – 1.32) | – | 4.64 (4.64 – 4.64) | – | 1.21 (1 – 1.54) | – |
| MAD of RR (bpm) | – | – | 2.3 (1.69 – 2.98) | – | 2.3 (2.01 – 2.96) | – | 2.07 (1.62 – 3.01) | – | 2.46 (2.05 – 2.88) | – | 3.58 (3.58 – 3.58) | – | 2.22 (1.99 – 2.91) | – |
| MAD of rSO_2_ (%) | Frontal | Left | 0.71 (0.51 – 0.98) | 0.1592 | 0.65 (0.45 – 0.79) | 0.6236 | 0.67 (0.38 – 0.86) | 0.3136 | 0.74 (0.65 – 1.05) | 0.2649 | 0.57 (0.57 – 0.57) | 1 | 0.71 (0.47 – 0.93) | 0.219 |
|  |  | Right | 0.85 (0.59 – 1.25) |  | 0.75 (0.53 – 0.9) |  | 0.74 (0.44 – 1.16) |  | 0.89 (0.74 – 1.05) |  | 2.05 (2.05 – 2.05) |  | 0.8 (0.59 – 1.08) |  |
|  | Parietal | Left | 1.09 (0.68 – 1.75) | 0.4207 | 1.12 (0.84 – 1.66) | 0.7508 | 0.95 (0.53 – 1.72) | 0.7869 | 1.17 (0.95 – 1.78) | 0.6138 | 2.59 (2.59 – 2.59) | 1 | 1.07 (0.71 – 1.7) | 0.5554 |
|  |  | Right | 0.9 (0.57 – 1.63) |  | 1.4 (0.84 – 2.33) |  | 0.87 (0.53 – 1.62) |  | 1.05 (0.78 – 2.36) |  | 2.14 (2.14 – 2.14) |  | 0.94 (0.69 – 1.74) |  |
|  | Temporal | Left | 0.79 (0.48 – 1.4) | 0.8557 | 1 (0.6 – 1.45) | 0.8399 | 0.79 (0.46 – 1.17) | 0.7618 | 0.97 (0.63 – 1.51) | 0.6985 | 1.01 (1.01 – 1.01) | 1 | 0.86 (0.51 – 1.46) | 0.9208 |
|  |  | Right | 0.87 (0.51 – 1.35) |  | 0.79 (0.58 – 1.4) |  | 0.79 (0.54 – 1.23) |  | 0.9 (0.48 – 1.47) |  | 0.86 (0.86 – 0.86) |  | 0.83 (0.49 – 1.38) |  |
|  | Occipital | Left | 0.34 (0.3 – 0.4) | **<0.001** | 0.38 (0.32 – 0.48) | **0.012** | 0.35 (0.3 – 0.39) | **<0.001** | 0.36 (0.32 – 0.49) | **<0.001** | 0.48 (0.48 – 0.48) | 1 | 0.36 (0.3 – 0.46) | **<0.001** |
|  |  | Right | 0.67 (0.46 – 1.22) |  | 0.69 (0.5 – 1.22) |  | 0.57 (0.45 – 1) |  | 0.82 (0.5 – 1.69) |  | 0.52 (0.52 – 0.52) |  | 0.69 (0.47 – 1.22) |  |
| MAD of HbO (au) | Frontal | Left | 3.43 (2.65 – 7.12) | 0.2467 | 3.77 (2.22 – 6.49) | 0.8852 | 4.45 (2.91 – 7.05) | 0.3631 | 3.06 (2.2 – 5.87) | 0.4455 | 3.21 (3.21 – 3.21) | 1 | 3.76 (2.48 – 7.01) | 0.3939 |
|  |  | Right | 4.54 (2.91 – 8.47) |  | 4.31 (2.39 – 7.37) |  | 5.22 (3.62 – 8.81) |  | 3.76 (2.65 – 5.03) |  | 16.45 (16.45 – 16.45) |  | 4.32 (2.81 – 8.04) |  |
|  | Parietal | Left | 8.74 (4.16 – 20.25) | 0.8967 | 12.97 (10.46 – 21.65) | 0.8399 | 10.34 (4.34 – 21.21) | 0.8763 | 11.43 (4.74 – 19.33) | 0.8603 | 4.42 (4.42 – 4.42) | 1 | 11.69 (4.38 – 20.66) | 0.9943 |
|  |  | Right | 9.91 (6.06 – 15.05) |  | 14.86 (7.92 – 35.17) |  | 10.38 (5.7 – 15.48) |  | 10.06 (7.05 – 28.95) |  | 9.89 (9.89 – 9.89) |  | 10.55 (6.09 – 17.14) |  |
|  | Temporal | Left | 7.46 (3.57 – 12.45) | 0.5784 | 10.62 (4.73 – 20.75) | 0.931 | 7.56 (4.73 – 12.49) | 0.2348 | 8.92 (3.17 – 16.59) | 0.5974 | 12.4 (12.4 – 12.4) | 1 | 8 (3.55 – 12.56) | 0.4643 |
|  |  | Right | 6.17 (3.46 – 11.52) |  | 6.23 (3.98 – 16.96) |  | 6.03 (3.55 – 8.38) |  | 6.38 (3.91 – 19.81) |  | 42.86 (42.86 – 42.86) |  | 6.09 (3.62 – 11.63) |  |
|  | Occipital | Left | 5.5 (3.62 – 8.2) | **0.0273** | 6.64 (4.27 – 10.26) | **0.012** | 5.76 (4.81 – 8.33) | **0.0232** | 4.64 (3.06 – 11.08) | **0.0116** | 19.77 (19.77 – 19.77) | 1 | 5.54 (3.7 – 8.71) | **0.0014** |
|  |  | Right | 9.77 (4.62 – 18.58) |  | 18.51 (6.67 – 31.62) |  | 9.77 (6.34 – 15.57) |  | 17.15 (4.77 – 37.74) |  | 9.96 (9.96 – 9.96) |  | 10.36 (4.88 – 22.65) |  |
| MAD of HHb (au) | Frontal | Left | 3.21 (1.45 – 5.35) | 0.1062 | 3.21 (1.65 – 4.24) | 0.2855 | 2.73 (1.45 – 5.83) | 0.3215 | 3.59 (1.59 – 5.07) | 0.098 | 2.04 (2.04 – 2.04) | 1 | 3.42 (1.45 – 5.41) | 0.0895 |
|  |  | Right | 4.37 (2.05 – 8.44) |  | 4.74 (3.21 – 5.8) |  | 4.83 (1.95 – 8.63) |  | 4.3 (3.36 – 6.92) |  | 20.76 (20.76 – 20.76) |  | 4.34 (2.06 – 7.45) |  |
|  | Parietal | Left | 10.45 (2.93 – 17.15) | 0.8557 | 13.78 (7.43 – 19.86) | 0.7508 | 10.45 (3.36 – 14.87) | 0.676 | 14.87 (3.69 – 18.45) | 0.8053 | 10.5 (10.5 – 10.5) | 1 | 11.97 (3.38 – 17.79) | 0.9321 |
|  |  | Right | 7.74 (4.21 – 12.47) |  | 15.07 (5.84 – 27.9) |  | 7.36 (3.76 – 12.15) |  | 10.53 (6.28 – 22.91) |  | 8.62 (8.62 – 8.62) |  | 8.08 (3.84 – 17.84) |  |
|  | Temporal | Left | 5.71 (2.81 – 10.78) | 0.5503 | 11.05 (2.25 – 17.48) | 0.8399 | 5.47 (2.29 – 10.8) | 0.8505 | 7.54 (3.36 – 11.78) | 0.4047 | 4.52 (4.52 – 4.52) | 1 | 6.26 (2.48 – 11.67) | 0.3199 |
|  |  | Right | 4.89 (3.04 – 8.04) |  | 4.74 (2.81 – 10.72) |  | 5.18 (2.88 – 7.83) |  | 4.78 (3.01 – 8.51) |  | 27.37 (27.37 – 27.37) |  | 4.85 (2.82 – 7.72) |  |
|  | Occipital | Left | 3.32 (2.08 – 5.25) | **<0.001** | 3 (2.6 – 8.37) | **0.0404** | 3.32 (2.72 – 4.89) | **0.002** | 3.06 (2.06 – 5.57) | **0.0024** | 6.73 (6.73 – 6.73) | 1 | 3.09 (2.08 – 5.36) | **<0.001** |
|  |  | Right | 8.59 (3.54 – 12.38) |  | 7.04 (3.67 – 16.06) |  | 7.58 (3.62 – 12.79) |  | 8.78 (3.75 – 15.2) |  | 10.91 (10.91 – 10.91) |  | 8.02 (3.66 – 14.13) |  |
| MAD of tHb (au) | Frontal | Left | 6.18 (3.72 – 12.87) | 0.4328 | 6 (3.06 – 9.55) | 0.7508 | 6.78 (4.3 – 12.36) | 0.9282 | 5.42 (2.71 – 10.04) | 0.2453 | 4.88 (4.88 – 4.88) | 1 | 6.01 (3.59 – 10.6) | 0.5133 |
|  |  | Right | 6.94 (4.73 – 15.15) |  | 6.43 (3.83 – 11.24) |  | 6.94 (4.76 – 16.14) |  | 7.11 (4.09 – 10.68) |  | 15.04 (15.04 – 15.04) |  | 6.9 (4.22 – 15.05) |  |
|  | Parietal | Left | 17.13 (6.29 – 36.45) | 0.4767 | 26.01 (16.34 – 38.74) | 0.7508 | 17.74 (6.65 – 37.38) | 0.3987 | 26.17 (7.3 – 37.36) | 0.8973 | 25.08 (25.08 – 25.08) | 1 | 18 (6.29 – 37.6) | 0.5843 |
|  |  | Right | 13.24 (7.59 – 23.77) |  | 28.54 (8.08 – 61.75) |  | 11.54 (7.09 – 24.11) |  | 17.3 (8.84 – 49.82) |  | 7.82 (7.82 – 7.82) |  | 16.87 (7.61 – 27.75) |  |
|  | Temporal | Left | 10.76 (5.2 – 19.13) | 0.9959 | 18.42 (7.75 – 28.64) | 0.7508 | 11.67 (5.39 – 23.87) | 0.7369 | 10.76 (5.5 – 24.37) | 0.9719 | 8.37 (8.37 – 8.37) | 1 | 11.12 (5.05 – 24.61) | 0.6544 |
|  |  | Right | 12 (7.02 – 14.52) |  | 10.41 (5.94 – 17.26) |  | 11.62 (6.86 – 14.74) |  | 11.3 (6.04 – 21.07) |  | 69.67 (69.67 – 69.67) |  | 11.05 (6.77 – 14.57) |  |
|  | Occipital | Left | 7.22 (2.86 – 10.37) | **<0.001** | 5.55 (3.21 – 14.35) | **0.0051** | 6.93 (4.13 – 10.97) | **<0.001** | 6.87 (1.89 – 12.88) | **0.0019** | 26.34 (26.34 – 26.34) | 1 | 6.91 (2.6 – 10.61) | **<0.001** |
|  |  | Right | 15.79 (6.3 – 28.94) |  | 29.64 (10.07 – 40.44) |  | 17.84 (8.98 – 27.1) |  | 27.34 (7.55 – 41.99) |  | 19.55 (19.55 – 19.55) |  | 21.23 (8.86 – 36.21) |  |
| MAD of HbDiff (au) | Frontal | Left | 3.24 (2.27 – 5.06) | 0.1655 | 3.52 (2.2 – 4.04) | 0.977 | 3.54 (1.93 – 5.37) | 0.3896 | 3.04 (2.4 – 4.51) | 0.3787 | 3.15 (3.15 – 3.15) | 1 | 3.35 (2.22 – 4.83) | 0.2996 |
|  |  | Right | 4.02 (2.6 – 5.82) |  | 3.18 (1.8 – 4.81) |  | 3.74 (2.58 – 6.21) |  | 3.54 (2.44 – 5.44) |  | 34.87 (34.87 – 34.87) |  | 3.66 (2.53 – 5.51) |  |
|  | Parietal | Left | 7.64 (4.67 – 12.2) | 0.5434 | 6.67 (4.9 – 9.36) | 0.2602 | 6.6 (3.16 – 12.15) | 0.7869 | 7.64 (6.12 – 12.22) | 0.8419 | 17.08 (17.08 – 17.08) | 1 | 7.25 (4.65 – 12.13) | 0.983 |
|  |  | Right | 6.25 (4.01 – 11.84) |  | 13.49 (6.46 – 17.28) |  | 6.51 (4.54 – 12.9) |  | 8.65 (4.25 – 16.4) |  | 15.42 (15.42 – 15.42) |  | 6.45 (4.37 – 15.58) |  |
|  | Temporal | Left | 5.64 (2.54 – 8.16) | 0.5784 | 5.41 (4.55 – 14.74) | 1 | 5.21 (2.78 – 9.39) | 1 | 6.58 (3.18 – 10.19) | 0.5186 | 16.39 (16.39 – 16.39) | 1 | 5.4 (2.9 – 8.24) | 0.5318 |
|  |  | Right | 5.38 (3.27 – 11.16) |  | 5.46 (3.1 – 8.91) |  | 4.91 (3.06 – 7.7) |  | 7.17 (4.78 – 16.1) |  | 3.59 (3.59 – 3.59) |  | 5.57 (3.17 – 11.1) |  |
|  | Occipital | Left | 5.52 (4.71 – 7.15) | 0.1416 | 6.79 (5.55 – 8.16) | 0.4357 | 5.95 (5.01 – 7.36) | **0.009** | 5.39 (4.74 – 7.55) | 0.6138 | 10.8 (10.8 – 10.8) | 1 | 5.79 (4.79 – 7.47) | 0.1131 |
|  |  | Right | 4.5 (3.32 – 8.48) |  | 5.13 (3.62 – 14.51) |  | 4.23 (3.35 – 5.96) |  | 7.62 (3.9 – 26.67) |  | 4.49 (4.49 – 4.49) |  | 4.5 (3.38 – 8.95) |  |
| **250 Hz Sampled Data** | | | | | | | | | | | | | | |
| ABP (mmHg) | – | – | 95.08 (89.74 – 100.84) | – | 95.77 (89.44 – 102.62) | – | 93.7 (89.31 – 99.33) | – | 96.2 (89.9 – 103.34) | – | 82.15 (78.69 – 86.29) | – | 95.16 (89.64 – 101.42) | – |
| EtCO₂ (mmHg) | – | – | 35.42 (34.42 – 36.93) | – | 35.67 (33.28 – 37.02) | – | 36.29 (34.3 – 37.65) | – | 34.87 (33.5 – 35.95) | – | 30.43 (23.9 – 33.97) | – | 35.56 (34.23 – 37) | – |
| RR (bpm) | – | – | 18.17 (15 – 20.59) | – | 16.33 (13.44 – 18.36) | – | 17.44 (14.41 – 20) | – | 18.54 (14.7 – 21.37) | – | 14.04 (11 – 18) | – | 17.86 (14.49 – 20) | – |
| rSO_2_ (%) | Frontal | Left | 46.3 (45.66 – 47.09) | **<0.001** | 44.32 (43.85 – 44.7) | **<0.001** | 45.25 (44.59 – 46.43) | **<0.001** | 45.26 (44.62 – 45.96) | **<0.001** | 42.91 (42.51 – 43.85) | 1 | 45.33 (44.66 – 46.13) | **<0.001** |
|  |  | Right | 40.87 (39.84 – 41.55) |  | 39.39 (38.97 – 40.3) |  | 40.87 (40.24 – 42.03) |  | 38.93 (37.59 – 40.13) |  | 37.61 (36.12 – 44.09) |  | 40.78 (39.65 – 41.18) |  |
|  | Parietal | Left | 50.11 (48.03 – 50.87) | **0.0214** | 47.85 (45.97 – 49.28) | **0.0051** | 50.67 (48.2 – 51.39) | **0.0052** | 47.95 (45.97 – 49.21) | 0.1697 | 53.64 (50.77 – 55.89) | 1 | 48.53 (47.23 – 50.51) | **0.0027** |
|  |  | Right | 45.68 (44.43 – 47.18) |  | 45.23 (43.3 – 46.94) |  | 45.24 (44.14 – 46.53) |  | 46.04 (44.56 – 47.7) |  | 44.71 (42.05 – 45.32) |  | 45.58 (44.18 – 47.28) |  |
|  | Temporal | Left | 40.52 (38.7 – 41.88) | **<0.001** | 40.88 (39.23 – 42.75) | **0.0061** | 39.71 (38.4 – 41.56) | **<0.001** | 41.92 (39.64 – 43.45) | 0.098 | 38.66 (37.84 – 41.56) | 1 | 40.83 (38.81 – 42.18) | **<0.001** |
|  |  | Right | 48.74 (47.6 – 49.64) |  | 45.88 (45.09 – 46.69) |  | 48.77 (47.35 – 49.55) |  | 46.17 (45.18 – 47.48) |  | 46.69 (46.09 – 48.88) |  | 48.02 (46.95 – 49.03) |  |
|  | Occipital | Left | 36.27 (35.92 – 36.66) | **<0.001** | 35.71 (35.15 – 36.28) | **<0.001** | 36.27 (35.92 – 36.62) | **<0.001** | 36.1 (35.55 – 36.77) | **<0.001** | 36.7 (36.29 – 37.27) | 1 | 36.2 (35.71 – 36.58) | **<0.001** |
|  |  | Right | 46.82 (46.23 – 48.45) |  | 45.62 (44.67 – 47.31) |  | 46.88 (46.55 – 47.86) |  | 46.03 (45.09 – 48.31) |  | 45.81 (45.41 – 46.51) |  | 46.77 (46.12 – 48.2) |  |
| HbO (au) | Frontal | Left | -117.33 (-127.41 – -113.44) | 0.08 | -126.65 (-128.35 – -119.92) | 0.5444 | -130 (-139.94 – -122.86) | 0.583 | -108.02 (-113.9 – -99.41) | 0.0528 | -137.61 (-140.1 – -131.64) | 1 | -119.14 (-125.27 – -114.12) | 0.0936 |
|  |  | Right | -127.77 (-136.31 – -119.61) |  | -129.44 (-133.61 – -127.84) |  | -131.74 (-141.73 – -126.71) |  | -126.85 (-132.49 – -120.79) |  | -523.03 (-533.15 – -168.7) |  | -125.41 (-135.97 – -121.74) |  |
|  | Parietal | Left | -173.14 (-193.01 – -158.13) | 0.2776 | -161.83 (-181.51 – -145.58) | 0.2366 | -172.04 (-184.52 – -160.47) | 0.21 | -176.26 (-189.99 – -145.59) | 0.5974 | -154.8 (-158.73 – -148.87) | 1 | -173.41 (-190.47 – -158.7) | 0.196 |
|  |  | Right | -184.92 (-205.29 – -170.39) |  | -186.25 (-218.91 – -173.92) |  | -190.66 (-208.07 – -173.94) |  | -177.6 (-206.52 – -168.49) |  | -231.57 (-248.22 – -224.46) |  | -182.32 (-206.56 – -170.27) |  |
|  | Temporal | Left | -203.93 (-209.16 – -195.29) | 0.0622 | -245.13 (-287.45 – -228.09) | 0.1749 | -198.74 (-206.57 – -186.46) | **0.0021** | -224.98 (-247.15 – -222.14) | 0.9345 | -448.03 (-456.5 – -401.85) | 1 | -207.39 (-223.46 – -200.38) | **0.0281** |
|  |  | Right | -152.36 (-161.59 – -139.07) |  | -168.48 (-184.7 – -145.93) |  | -145.3 (-157.58 – -134.03) |  | -228.65 (-260.93 – -213.01) |  | -183.25 (-242.87 – -145.32) |  | -154.78 (-165.29 – -140.15) |  |
|  | Occipital | Left | -496.29 (-505.24 – -491.42) | **<0.001** | -495.29 (-506.5 – -474.32) | **<0.001** | -504.36 (-508.73 – -497.83) | **<0.001** | -486.89 (-504.48 – -464.33) | **<0.001** | -466.18 (-484.72 – -444.71) | 1 | -495.55 (-506.17 – -486.28) | **<0.001** |
|  |  | Right | -181.57 (-205.63 – -159.47) |  | -207.78 (-221.74 – -187.5) |  | -171.3 (-187.4 – -159.47) |  | -213.06 (-234.94 – -197.49) |  | -245.26 (-256.33 – -236.89) |  | -186.98 (-205.84 – -167.38) |  |
| HHb (au) | Frontal | Left | -112.46 (-116.7 – -110.27) | 0.2384 | -97.38 (-102.75 – -91.75) | 0.1572 | -114.41 (-117.45 – -112.58) | 0.1215 | -104.01 (-109.96 – -95.58) | 0.3787 | -102.14 (-103.82 – -99.96) | 1 | -109.88 (-111.6 – -107.91) | 0.0524 |
|  |  | Right | -86.53 (-94.48 – -78.45) |  | -81.98 (-91.33 – -77.03) |  | -87.83 (-100.64 – -81.71) |  | -80.78 (-88.69 – -72.44) |  | -293.04 (-300.4 – -131.84) |  | -84.56 (-92.69 – -77.49) |  |
|  | Parietal | Left | -169.55 (-191.06 – -157.29) | 0.6364 | -172.45 (-204.65 – -158.68) | 0.8852 | -172.17 (-191.06 – -162.21) | 0.5607 | -159.21 (-199.5 – -143.84) | 0.9719 | -164.94 (-192.93 – -160.51) | 1 | -170.03 (-198.23 – -156.58) | 0.7331 |
|  |  | Right | -163.41 (-183.31 – -147.36) |  | -168.64 (-204.34 – -160.06) |  | -160.36 (-179.22 – -147.66) |  | -167.05 (-191.47 – -152.26) |  | -182.29 (-185.06 – -167.36) |  | -158.6 (-183.38 – -145.52) |  |
|  | Temporal | Left | -135.29 (-151.16 – -133.11) | 0.3914 | -163.43 (-235.97 – -157.35) | 1 | -134.91 (-146.05 – -131.2) | 1 | -152.37 (-169.74 – -142.1) | 0.2092 | -275.27 (-279.4 – -269.72) | 1 | -138.24 (-160.79 – -133.79) | 0.422 |
|  |  | Right | -145.4 (-154.8 – -130.51) |  | -163.52 (-167.89 – -146.25) |  | -135.01 (-142.6 – -128.85) |  | -243.9 (-261.3 – -238.97) |  | -147.51 (-231.77 – -124.01) |  | -149.2 (-157.05 – -132.53) |  |
|  | Occipital | Left | -270.6 (-277.98 – -267.79) | **<0.001** | -268.11 (-279.79 – -259.78) | **0.002** | -277.71 (-281.02 – -274.46) | **<0.001** | -261.01 (-277.36 – -258.25) | **<0.001** | -263.41 (-276.11 – -259) | 1 | -269.34 (-279.58 – -267.13) | **<0.001** |
|  |  | Right | -163.28 (-181.5 – -150.71) |  | -193.42 (-207.96 – -175.96) |  | -157.74 (-168.64 – -144.06) |  | -199.93 (-237.6 – -178.75) |  | -211.05 (-222.5 – -200.6) |  | -163.74 (-183.38 – -154.15) |  |
| tHb (au) | Frontal | Left | -225.29 (-243.25 – -213.03) | 0.9793 | -226.51 (-240.65 – -215.02) | 0.4705 | -242.59 (-246.06 – -234.41) | 0.4659 | -207.13 (-227.2 – -196.54) | 0.7336 | -240.24 (-243.44 – -231.01) | 1 | -226.23 (-241.34 – -210.5) | 0.604 |
|  |  | Right | -215.18 (-231.84 – -198.74) |  | -213.2 (-223.36 – -204.59) |  | -221.54 (-242.78 – -205.69) |  | -212.01 (-221.56 – -197.63) |  | -827.01 (-834.86 – -301.1) |  | -214 (-228.77 – -196.28) |  |
|  | Parietal | Left | -340.67 (-375.16 – -317.94) | 0.7514 | -334.24 (-385.7 – -304.66) | 0.5067 | -344.38 (-375.16 – -320.96) | 0.7246 | -334.05 (-398.3 – -282.83) | 0.8235 | -322.98 (-348.71 – -305.2) | 1 | -342.86 (-385.36 – -320.63) | 0.624 |
|  |  | Right | -346.92 (-369.94 – -321.61) |  | -353.18 (-415.25 – -341.21) |  | -341.72 (-384.39 – -323.36) |  | -354.94 (-387.91 – -325.41) |  | -407.16 (-417.45 – -400.69) |  | -343.92 (-379.38 – -318.9) |  |
|  | Temporal | Left | -338.96 (-359.16 – -334.07) | 0.4451 | -401.63 (-525.12 – -379.35) | 0.4705 | -332.46 (-350.97 – -318.45) | 0.103 | -382.88 (-422.8 – -366.42) | 0.5812 | -724.62 (-730.8 – -682.81) | 1 | -355.67 (-369.67 – -335.54) | 0.3594 |
|  |  | Right | -304.54 (-319.28 – -270.35) |  | -329.3 (-343.2 – -310.17) |  | -279.15 (-295.52 – -263.33) |  | -509.33 (-541.86 – -467.46) |  | -330.1 (-475.33 – -269.25) |  | -305.84 (-321.04 – -273.93) |  |
|  | Occipital | Left | -763.26 (-794.03 – -755.68) | **<0.001** | -764.89 (-786.77 – -730.8) | **<0.001** | -792.74 (-798.48 – -779.59) | **<0.001** | -745 (-779.67 – -717.49) | **<0.001** | -724.62 (-762.67 – -704.56) | 1 | -765.69 (-793.3 – -754.62) | **<0.001** |
|  |  | Right | -353.23 (-390.39 – -318.09) |  | -399.05 (-426.79 – -363.72) |  | -327.17 (-357.22 – -302.76) |  | -409.65 (-488.97 – -379.24) |  | -457.19 (-476.01 – -437.73) |  | -353.4 (-391.1 – -333.33) |  |
| HbDiff (au) | Frontal | Left | -14.01 (-17.17 – -11.73) | **<0.001** | -26.56 (-29.23 – -22.87) | **<0.001** | -20.48 (-23.47 – -13.98) | **<0.001** | -17.46 (-20.28 – -15.51) | **<0.001** | -33.91 (-36.14 – -28.77) | 1 | -18.65 (-21.53 – -12.84) | **<0.001** |
|  |  | Right | -41.9 (-45.55 – -35.83) |  | -46.79 (-50.97 – -42.69) |  | -41.9 (-45.28 – -35.83) |  | -47.2 (-50.54 – -42.69) |  | -210.7 (-233.91 – -35.44) |  | -44.01 (-48.37 – -38.61) |  |
|  | Parietal | Left | 1.25 (-14.65 – 4.25) | **0.0197** | -12.31 (-23.05 – -4.79) | **0.012** | 3.15 (-10.17 – 7.97) | **0.0052** | -12.82 (-23.05 – -6.09) | 0.2008 | 21.98 (3.94 – 37.67) | 1 | -9.7 (-18.9 – 2.34) | **0.0036** |
|  |  | Right | -24.64 (-33.37 – -18.58) |  | -32.33 (-39.13 – -23.75) |  | -32.89 (-40.75 – -24.85) |  | -24.64 (-32.91 – -10.69) |  | -44.18 (-65.55 – -38.13) |  | -29.27 (-35.61 – -18.86) |  |
|  | Temporal | Left | -56.03 (-63.34 – -50.23) | **<0.001** | -68.37 (-83.5 – -56.38) | **0.0194** | -61.91 (-71.93 – -53.39) | **<0.001** | -51.99 (-66.01 – -48.58) | 0.13 | -170.26 (-182.46 – -122.13) | 1 | -58.09 (-71.69 – -48.65) | **<0.001** |
|  |  | Right | -6.15 (-13.82 – -1.98) |  | -22.72 (-26.26 – -17.12) |  | -6.15 (-13.82 – -2.08) |  | -20.34 (-27.03 – -12.43) |  | -19.61 (-21.6 – -10.66) |  | -7.9 (-17.23 – -5.38) |  |
|  | Occipital | Left | -219.98 (-225.99 – -212.03) | **<0.001** | -221.05 (-229.36 – -211.75) | **<0.001** | -221.13 (-228.02 – -214.06) | **<0.001** | -218.78 (-226.69 – -207.89) | **<0.001** | -201.49 (-209.21 – -185.53) | 1 | -220.78 (-228.14 – -212.37) | **<0.001** |
|  |  | Right | -20.05 (-23.24 – -9.05) |  | -28.85 (-33.14 – -18.5) |  | -18.84 (-22.44 – -13.05) |  | -32.22 (-37.21 – -9.46) |  | -37.4 (-40.74 – -31.22) |  | -20.58 (-23.24 – -11.34) |  |
| MAD of ABP (mmHg) | – | – | 5.51 (4.82 – 6.54) | – | 5.37 (4.85 – 6.73) | – | 5.31 (4.79 – 6.49) | – | 6 (5.03 – 6.9) | – | 3.74 (3.74 – 3.74) | – | 5.54 (4.82 – 6.65) | – |
| MAD of EtCO₂ (mmHg) | – | – | 1.17 (1 – 1.42) | – | 1.48 (1.14 – 1.68) | – | 1.39 (1 – 1.75) | – | 1.14 (1 – 1.31) | – | 4.57 (4.57 – 4.57) | – | 1.21 (1 – 1.56) | – |
| MAD of RR (bpm) | – | – | 2.29 (1.68 – 2.99) | – | 2.26 (2.04 – 2.94) | – | 2.11 (1.61 – 3.04) | – | 2.44 (2.15 – 2.89) | – | 3.51 (3.51 – 3.51) | – | 2.25 (1.99 – 2.92) | – |
| MAD of rSO_2_ (%) | Frontal | Left | 0.71 (0.52 – 0.98) | 0.1655 | 0.65 (0.45 – 0.77) | 0.665 | 0.67 (0.39 – 0.87) | 0.2981 | 0.74 (0.64 – 1.03) | 0.2855 | 0.58 (0.58 – 0.58) | 1 | 0.71 (0.48 – 0.95) | 0.2271 |
|  |  | Right | 0.85 (0.59 – 1.25) |  | 0.75 (0.53 – 0.9) |  | 0.74 (0.43 – 1.19) |  | 0.89 (0.73 – 1.05) |  | 2.08 (2.08 – 2.08) |  | 0.8 (0.59 – 1.09) |  |
|  | Parietal | Left | 1.1 (0.65 – 1.79) | 0.4089 | 1.12 (0.81 – 1.65) | 0.665 | 0.93 (0.55 – 1.78) | 0.7995 | 1.2 (0.94 – 1.8) | 0.6304 | 2.61 (2.61 – 2.61) | 1 | 1.06 (0.7 – 1.77) | 0.5554 |
|  |  | Right | 0.93 (0.57 – 1.64) |  | 1.39 (0.85 – 2.29) |  | 0.9 (0.54 – 1.62) |  | 1.06 (0.8 – 2.33) |  | 2.22 (2.22 – 2.22) |  | 0.94 (0.69 – 1.75) |  |
|  | Temporal | Left | 0.8 (0.49 – 1.38) | 0.8557 | 1.02 (0.58 – 1.35) | 0.8852 | 0.8 (0.47 – 1.17) | 0.7869 | 1 (0.64 – 1.51) | 0.7872 | 1.05 (1.05 – 1.05) | 1 | 0.84 (0.5 – 1.42) | 0.9377 |
|  |  | Right | 0.86 (0.49 – 1.34) |  | 0.78 (0.59 – 1.42) |  | 0.78 (0.53 – 1.22) |  | 0.89 (0.51 – 1.45) |  | 0.87 (0.87 – 0.87) |  | 0.83 (0.47 – 1.4) |  |
|  | Occipital | Left | 0.36 (0.31 – 0.42) | **<0.001** | 0.41 (0.35 – 0.5) | **0.012** | 0.36 (0.3 – 0.42) | **<0.001** | 0.36 (0.34 – 0.49) | **<0.001** | 0.47 (0.47 – 0.47) | 1 | 0.36 (0.31 – 0.49) | **<0.001** |
|  |  | Right | 0.67 (0.46 – 1.28) |  | 0.69 (0.51 – 1.21) |  | 0.58 (0.45 – 1) |  | 0.81 (0.5 – 1.73) |  | 0.52 (0.52 – 0.52) |  | 0.69 (0.48 – 1.29) |  |
| MAD of HbO (au) | Frontal | Left | 3.38 (2.62 – 7.19) | 0.2641 | 3.81 (2.21 – 6.59) | 0.931 | 4.46 (2.92 – 7.04) | 0.3545 | 3.11 (2.22 – 5.75) | 0.5035 | 3.2 (3.2 – 3.2) | 1 | 3.81 (2.47 – 6.96) | 0.4139 |
|  |  | Right | 4.53 (2.93 – 8.35) |  | 4.35 (2.43 – 7.34) |  | 5.12 (3.63 – 8.76) |  | 3.81 (2.64 – 5.03) |  | 17.45 (17.45 – 17.45) |  | 4.3 (2.82 – 8.01) |  |
|  | Parietal | Left | 8.89 (4.17 – 20.32) | 0.9296 | 13.05 (10.43 – 21.83) | 0.8399 | 11.02 (4.38 – 21.2) | 0.7995 | 11.46 (4.49 – 19.51) | 0.7872 | 4.46 (4.46 – 4.46) | 1 | 11.7 (4.21 – 20.9) | 0.983 |
|  |  | Right | 9.8 (5.93 – 14.95) |  | 14.95 (8 – 34.96) |  | 10.39 (5.73 – 15.31) |  | 10.18 (7.06 – 28.88) |  | 9.99 (9.99 – 9.99) |  | 10.79 (6.08 – 17.44) |  |
|  | Temporal | Left | 7.35 (3.6 – 12.42) | 0.5784 | 10.55 (4.56 – 20.98) | 0.977 | 7.37 (4.87 – 12.62) | 0.2548 | 8.97 (3.14 – 16.61) | 0.5974 | 11.87 (11.87 – 11.87) | 1 | 8.05 (3.54 – 12.67) | 0.4686 |
|  |  | Right | 6.14 (3.45 – 11.55) |  | 6.25 (4.34 – 16.86) |  | 6.1 (3.53 – 8.4) |  | 6.34 (3.89 – 20.04) |  | 42.61 (42.61 – 42.61) |  | 6.11 (3.62 – 11.6) |  |
|  | Occipital | Left | 5.49 (3.56 – 8.33) | **0.0266** | 6.89 (4.34 – 10.58) | **0.0166** | 5.83 (4.82 – 8.53) | **0.0287** | 4.67 (3.04 – 11.13) | **0.0116** | 19.44 (19.44 – 19.44) | 1 | 5.52 (3.7 – 8.82) | **0.0014** |
|  |  | Right | 9.75 (4.64 – 18.51) |  | 18.01 (6.64 – 31.18) |  | 9.75 (6.28 – 15.55) |  | 17.05 (4.76 – 38.51) |  | 9.9 (9.9 – 9.9) |  | 10.34 (4.95 – 22.59) |  |
| MAD of HHb (au) | Frontal | Left | 3.24 (1.44 – 5.27) | 0.1131 | 3.25 (1.62 – 4.21) | 0.3123 | 2.71 (1.44 – 5.75) | 0.3461 | 3.59 (1.58 – 5.1) | 0.1131 | 2.06 (2.06 – 2.06) | 1 | 3.43 (1.45 – 5.3) | 0.0964 |
|  |  | Right | 4.38 (2.03 – 8.37) |  | 4.79 (3.23 – 5.77) |  | 4.86 (1.95 – 8.58) |  | 4.31 (3.4 – 6.95) |  | 20.65 (20.65 – 20.65) |  | 4.37 (2.05 – 7.41) |  |
|  | Parietal | Left | 10.45 (2.98 – 17.17) | 0.8476 | 13.69 (7.92 – 19.57) | 0.7508 | 10.45 (3.41 – 14.48) | 0.676 | 14.71 (3.62 – 18.13) | 0.8053 | 10.66 (10.66 – 10.66) | 1 | 12.07 (3.29 – 17.86) | 0.9208 |
|  |  | Right | 7.72 (4.19 – 12.69) |  | 14.97 (5.87 – 27.86) |  | 7.34 (3.74 – 12.17) |  | 10.45 (6.09 – 22.85) |  | 8.66 (8.66 – 8.66) |  | 8.1 (3.81 – 17.71) |  |
|  | Temporal | Left | 5.67 (2.8 – 10.7) | 0.5572 | 10.77 (2.23 – 17.37) | 0.8852 | 5.43 (2.37 – 10.63) | 0.8763 | 7.53 (3.38 – 11.8) | 0.4181 | 4.45 (4.45 – 4.45) | 1 | 6.19 (2.43 – 11.74) | 0.3339 |
|  |  | Right | 4.9 (3.12 – 7.89) |  | 4.76 (2.85 – 10.57) |  | 5.18 (2.88 – 7.79) |  | 4.8 (3.13 – 8.51) |  | 27.42 (27.42 – 27.42) |  | 4.84 (2.88 – 7.74) |  |
|  | Occipital | Left | 3.33 (2.16 – 5.03) | **<0.001** | 2.94 (2.49 – 8.25) | **0.0404** | 3.33 (2.61 – 4.81) | **0.0018** | 3.11 (2.13 – 5.59) | **0.0037** | 6.36 (6.36 – 6.36) | 1 | 3.2 (2.18 – 5.26) | **<0.001** |
|  |  | Right | 8.63 (3.5 – 12.32) |  | 6.93 (3.76 – 16.37) |  | 7.63 (3.61 – 12.77) |  | 8.53 (3.77 – 15.07) |  | 10.62 (10.62 – 10.62) |  | 8.03 (3.66 – 13.92) |  |
| MAD of tHb (au) | Frontal | Left | 6.28 (3.66 – 12.8) | 0.4513 | 5.97 (2.95 – 9.55) | 0.7508 | 6.74 (4.35 – 12.31) | 0.9152 | 5.45 (2.68 – 10.02) | 0.2453 | 4.75 (4.75 – 4.75) | 1 | 6.21 (3.54 – 10.51) | 0.5271 |
|  |  | Right | 6.94 (4.73 – 15.03) |  | 6.43 (3.82 – 11.15) |  | 6.94 (4.77 – 16.02) |  | 7.09 (4.06 – 10.89) |  | 14.33 (14.33 – 14.33) |  | 6.88 (4.12 – 14.83) |  |
|  | Parietal | Left | 17.13 (6.3 – 36.24) | 0.4576 | 25.82 (16.17 – 39.1) | 0.7508 | 17.73 (6.66 – 37.34) | 0.3987 | 26.13 (7.49 – 37.3) | 0.9159 | 25.01 (25.01 – 25.01) | 1 | 18.12 (6.5 – 37.6) | 0.5746 |
|  |  | Right | 13.29 (7.49 – 23.86) |  | 28.01 (8.07 – 61.28) |  | 11.51 (7.02 – 24.14) |  | 16.8 (8.84 – 49.91) |  | 7.74 (7.74 – 7.74) |  | 16.44 (7.47 – 27.59) |  |
|  | Temporal | Left | 10.86 (5.38 – 19.17) | 1 | 18.57 (8 – 30.73) | 0.7508 | 11.7 (5.3 – 24.14) | 0.7618 | 10.86 (5.7 – 24.12) | 1 | 8.19 (8.19 – 8.19) | 1 | 11.18 (5.32 – 24.92) | 0.634 |
|  |  | Right | 12.19 (7.06 – 14.51) |  | 10.46 (5.96 – 17.35) |  | 11.48 (6.9 – 14.72) |  | 11.39 (6.03 – 21.04) |  | 69.73 (69.73 – 69.73) |  | 11.04 (6.78 – 14.53) |  |
|  | Occipital | Left | 7.12 (2.92 – 10.52) | **<0.001** | 5.62 (3.34 – 14.34) | **0.0043** | 6.83 (4.17 – 11.23) | **<0.001** | 7.01 (1.95 – 12.89) | **0.0017** | 26.34 (26.34 – 26.34) | 1 | 6.73 (2.69 – 11.1) | **<0.001** |
|  |  | Right | 15.23 (6.25 – 28.92) |  | 29.66 (9.98 – 40.58) |  | 17.74 (8.99 – 27.06) |  | 27.1 (7.46 – 42.3) |  | 19.34 (19.34 – 19.34) |  | 21.49 (8.88 – 35.38) |  |
| MAD of HbDiff (au) | Frontal | Left | 3.24 (2.27 – 5.06) | 0.1655 | 3.5 (2.21 – 4.01) | 0.977 | 3.54 (1.95 – 5.3) | 0.3545 | 3.02 (2.42 – 4.64) | 0.3916 | 3.16 (3.16 – 3.16) | 1 | 3.32 (2.22 – 4.89) | 0.2801 |
|  |  | Right | 4.09 (2.61 – 5.72) |  | 3.14 (1.77 – 4.84) |  | 3.82 (2.57 – 6.23) |  | 3.58 (2.42 – 5.38) |  | 35.15 (35.15 – 35.15) |  | 3.7 (2.52 – 5.52) |  |
|  | Parietal | Left | 7.69 (4.75 – 12.26) | 0.5161 | 6.6 (4.66 – 9.43) | 0.2366 | 6.66 (3.15 – 12.21) | 0.7743 | 7.75 (6.13 – 12.3) | 0.8235 | 17.16 (17.16 – 17.16) | 1 | 7.33 (4.63 – 12.18) | 0.9943 |
|  |  | Right | 6.22 (4.14 – 11.87) |  | 13.51 (6.44 – 17.34) |  | 6.46 (4.4 – 12.94) |  | 8.48 (4.4 – 16.53) |  | 15.7 (15.7 – 15.7) |  | 6.43 (4.36 – 15.53) |  |
|  | Temporal | Left | 5.67 (2.51 – 8.1) | 0.5926 | 5.38 (4.49 – 14.79) | 1 | 5.18 (2.71 – 9.23) | 0.9673 | 6.51 (3.18 – 9.72) | 0.5186 | 15.96 (15.96 – 15.96) | 1 | 5.42 (2.82 – 8.18) | 0.5746 |
|  |  | Right | 5.27 (3.29 – 11.12) |  | 5.46 (3.14 – 8.93) |  | 4.9 (3.07 – 7.66) |  | 7.27 (4.54 – 16.1) |  | 3.72 (3.72 – 3.72) |  | 5.42 (3.18 – 11.08) |  |
|  | Occipital | Left | 5.5 (4.65 – 7.14) | 0.1333 | 6.78 (5.43 – 8.09) | 0.4025 | 6.01 (5.02 – 7.18) | **0.0085** | 5.37 (4.83 – 7.72) | 0.6812 | 10.16 (10.16 – 10.16) | 1 | 5.65 (4.88 – 7.28) | 0.1068 |
|  |  | Right | 4.39 (3.27 – 8.58) |  | 5.07 (3.63 – 14.2) |  | 4.24 (3.31 – 5.99) |  | 7.39 (3.97 – 26.45) |  | 4.36 (4.36 – 4.36) |  | 4.43 (3.35 – 9.07) |  |
| The p-values in the table are derived using Mann-Whitney U test between the bilateral signals. *ABP, arterial blood pressure; bpm, beats per minute; EtCO_2_, end-tidal carbon dioxide; HbDiff, hemoglobin difference; HbO, oxyhemoglobin; HHb, deoxyhemoglobin; IQR, interquartile range; MAD, median absolute deviation; mmHg, millimeters of mercury; RR, respiratory rate; rSO_2_, regional cerebral oxygen saturation; tHb, total hemoglobin.* | | | | | | | | | | | | | | |

Appendix S6d: Subgrouped Physiologic Results Using Raw Data

| **Physiologic Signal** | **Brain Lobe** | **Hemisphere** | **Subgroups** | | | | | | | | | | | |
| --- | --- | --- | --- | --- | --- | --- | --- | --- | --- | --- | --- | --- | --- | --- |
|  |  |  | **Age < 40 [n=38]** | | **Age 40 – 60 [n=12]** | | **Males [n=28]** | | **Females [n=22]** | | **Left Hand Dominance [n=1]** | | **Right Hand Dominance [n=49]** | |
|  |  |  | **Median (IQR)** | **p-value** | **Median (IQR)** | **p-value** | **Median (IQR)** | **p-value** | **Median (IQR)** | **p-value** | **Median (IQR)** | **p-value** | **Median (IQR)** | **p-value** |
| **1 Hz Sampled Data** | | | | | | | | | | | | | | |
| ABP (mmHg) | – | – | 94.44 (87.84 – 101.05) | – | 94.27 (87.98 – 102.36) | – | 92.5 (87.31 – 99.53) | – | 94.71 (88.41 – 103.64) | – | 81.04 (76.36 – 85.95) | – | 94.63 (88.06 – 101.24) | – |
| EtCO₂ (mmHg) | – | – | 35.79 (34.5 – 37) | – | 35.5 (33 – 37) | – | 36 (34.18 – 38) | – | 35 (34 – 36) | – | 29 (21 – 34.19) | – | 36 (34 – 37) | – |
| RR (bpm) | – | – | 18 (15 – 20.5) | – | 16 (13.05 – 18.5) | – | 17.33 (14.91 – 20) | – | 18.5 (15 – 21.5) | – | 15 (11 – 19) | – | 18 (15 – 20) | – |
| rSO_2_ (%) | Frontal | Left | 46.25 (45.51 – 47.08) | **<0.001** | 44.37 (43.89 – 44.79) | **<0.001** | 45.29 (44.61 – 46.44) | **<0.001** | 45.27 (44.46 – 46.02) | **<0.001** | 43.1 (42.56 – 44.15) | 1 | 45.34 (44.66 – 46.12) | **<0.001** |
|  |  | Right | 40.8 (39.72 – 41.64) |  | 39.41 (39 – 40.35) |  | 40.86 (40.22 – 42.09) |  | 39.03 (37.62 – 40.34) |  | 37.21 (35.92 – 43.96) |  | 40.73 (39.42 – 41.17) |  |
|  | Parietal | Left | 49.55 (47.98 – 50.89) | **0.0232** | 47.8 (45.4 – 49.38) | **0.0073** | 50.7 (48.28 – 51.42) | **0.0055** | 47.89 (45.4 – 49.23) | 0.1927 | 54.09 (50.84 – 55.83) | 1 | 48.58 (47.18 – 50.52) | **0.003** |
|  |  | Right | 45.7 (44.46 – 47.28) |  | 45.01 (43.37 – 46.43) |  | 45.34 (44.09 – 46.54) |  | 46.07 (44.71 – 48.34) |  | 44.49 (42.17 – 45.23) |  | 45.55 (44.23 – 47.21) |  |
|  | Temporal | Left | 40.42 (38.72 – 41.82) | **<0.001** | 40.84 (38.91 – 42.66) | **0.0061** | 39.73 (38.37 – 41.18) | **<0.001** | 41.88 (39.37 – 43.11) | 0.098 | 38.42 (37.1 – 40.83) | 1 | 40.63 (38.83 – 42.21) | **<0.001** |
|  |  | Right | 48.77 (47.5 – 49.63) |  | 45.89 (45.19 – 46.67) |  | 48.7 (47.3 – 49.53) |  | 46.33 (45.19 – 47.48) |  | 46.75 (46.06 – 48.55) |  | 48.35 (46.96 – 48.96) |  |
|  | Occipital | Left | 36.05 (35.19 – 36.87) | **<0.001** | 35.45 (34.46 – 36.42) | **<0.001** | 36.05 (35.17 – 36.81) | **<0.001** | 35.82 (34.84 – 36.87) | **<0.001** | 36.4 (35.45 – 37.39) | 1 | 35.9 (35.01 – 36.78) | **<0.001** |
|  |  | Right | 46.96 (46.27 – 48.42) |  | 45.51 (44.68 – 47.4) |  | 46.99 (46.48 – 47.91) |  | 45.96 (45.07 – 48.23) |  | 45.96 (45.4 – 46.86) |  | 46.86 (46.13 – 48.11) |  |
| HbO (au) | Frontal | Left | -117.85 (-127.87 – -113.21) | 0.0875 | -125.82 (-128.57 – -119.99) | 0.5444 | -129.78 (-139.76 – -123.14) | 0.5718 | -110.21 (-114.87 – -99.43) | **0.0473** | -136.66 (-139.75 – -127.83) | 1 | -119.16 (-125.51 – -114.08) | 0.0908 |
|  |  | Right | -128.46 (-136.45 – -119.37) |  | -129.32 (-134.61 – -127.05) |  | -133.87 (-142.15 – -127.04) |  | -127.1 (-132.65 – -120.58) |  | -193.48 (-531.31 – -165.64) |  | -126.72 (-136.2 – -121.82) |  |
|  | Parietal | Left | -172.88 (-192.91 – -155.09) | 0.2731 | -163.5 (-220.43 – -146.26) | 0.2366 | -171.76 (-185.21 – -159.37) | 0.1983 | -176.33 (-202.58 – -145.22) | 0.5186 | -154.26 (-159.54 – -147.73) | 1 | -172.95 (-195.41 – -158.24) | 0.2009 |
|  |  | Right | -183.81 (-204.88 – -170.25) |  | -186.75 (-218.54 – -173.6) |  | -191.71 (-208.34 – -173.92) |  | -179.89 (-204.92 – -167.65) |  | -230.14 (-247.66 – -224.68) |  | -182.46 (-206.99 – -170.2) |  |
|  | Temporal | Left | -204.31 (-209.18 – -197.33) | 0.0667 | -244.38 (-284.57 – -229.65) | 0.1749 | -198.88 (-206.25 – -187.48) | **0.0022** | -225.79 (-248.86 – -222.29) | 0.9906 | -447.03 (-460.54 – -412.9) | 1 | -206.46 (-222.44 – -200.19) | **0.0292** |
|  |  | Right | -152.18 (-162.71 – -139.3) |  | -169.14 (-184.63 – -145.05) |  | -145.73 (-157.83 – -133.91) |  | -233.15 (-271.96 – -213.2) |  | -174.63 (-241.44 – -144.52) |  | -154.63 (-160.82 – -138.62) |  |
|  | Occipital | Left | -496.46 (-506.37 – -488.93) | **<0.001** | -491.73 (-507.72 – -472.86) | **<0.001** | -503.21 (-510.78 – -494.61) | **<0.001** | -479.28 (-505.34 – -461.51) | **<0.001** | -471.5 (-487.3 – -448.1) | 1 | -496.11 (-507.55 – -485.81) | **<0.001** |
|  |  | Right | -181.85 (-205.08 – -158.31) |  | -204.77 (-222.05 – -187.09) |  | -169.24 (-185.49 – -158.31) |  | -214.95 (-233.6 – -192.23) |  | -249.63 (-261.37 – -237.67) |  | -184.73 (-205.38 – -165.99) |  |
| HHb (au) | Frontal | Left | -112.6 (-117.54 – -110.29) | 0.2342 | -97.42 (-102.67 – -91.89) | 0.1572 | -114.46 (-117.54 – -112.58) | 0.1065 | -103.75 (-110.04 – -95.79) | 0.3916 | -101.66 (-103.62 – -99.33) | 1 | -111.03 (-111.6 – -107.85) | 0.0578 |
|  |  | Right | -87.37 (-94.56 – -78.56) |  | -82.67 (-91.42 – -77.12) |  | -88.69 (-100.61 – -81.86) |  | -81.34 (-87.35 – -72.48) |  | -160.4 (-300.35 – -129.81) |  | -85.65 (-92.78 – -77.5) |  |
|  | Parietal | Left | -172.14 (-192.9 – -157.48) | 0.6144 | -174.2 (-222.28 – -159.46) | 1 | -172.21 (-192.9 – -161.46) | 0.4965 | -159.86 (-200.57 – -143.78) | 0.9719 | -172.18 (-193.51 – -160.52) | 1 | -172.11 (-198.75 – -156.42) | 0.6699 |
|  |  | Right | -163.23 (-183.3 – -146.09) |  | -169.83 (-203.33 – -160.15) |  | -158.66 (-178.73 – -146.51) |  | -167.96 (-192.46 – -153.88) |  | -182.62 (-184.47 – -169.43) |  | -160.7 (-183.66 – -146.06) |  |
|  | Temporal | Left | -134.71 (-148.08 – -132.66) | 0.38 | -160.64 (-238.02 – -157) | 0.977 | -133.82 (-144.43 – -131.23) | 0.9673 | -150.3 (-171.05 – -141.64) | 0.2178 | -274.71 (-282.34 – -265.91) | 1 | -140.49 (-156.67 – -133.2) | 0.3899 |
|  |  | Right | -145.68 (-158.13 – -130.57) |  | -163.22 (-174.21 – -145.91) |  | -134.94 (-141.84 – -128.99) |  | -244.73 (-267.33 – -237.34) |  | -147.79 (-230.61 – -122.81) |  | -150.21 (-158.25 – -132.62) |  |
|  | Occipital | Left | -270.14 (-280.78 – -264.34) | **<0.001** | -269.21 (-279.87 – -258.37) | **0.0011** | -277.43 (-283.28 – -271.98) | **<0.001** | -263.66 (-276.45 – -254.81) | **<0.001** | -268.19 (-277.16 – -258.75) | 1 | -269.7 (-281.02 – -263) | **<0.001** |
|  |  | Right | -161.01 (-180.34 – -150.83) |  | -192.95 (-207.8 – -176.06) |  | -156.49 (-164.05 – -142.86) |  | -202.65 (-238.06 – -178.7) |  | -213.29 (-238.81 – -201.27) |  | -162.74 (-181.16 – -154.61) |  |
| tHb (au) | Frontal | Left | -230.27 (-242.88 – -212.97) | 0.9627 | -227.08 (-240.59 – -215.29) | 0.5067 | -242.18 (-245.87 – -232.22) | 0.4659 | -207.15 (-226.99 – -198.16) | 0.7336 | -239.32 (-243.01 – -227.4) | 1 | -227.61 (-241.58 – -210.63) | 0.624 |
|  |  | Right | -216.95 (-232.11 – -198.91) |  | -215.13 (-226.88 – -204.79) |  | -224.37 (-242.84 – -206.02) |  | -212.3 (-221.75 – -197.68) |  | -353.32 (-833.37 – -296.62) |  | -216.72 (-229.09 – -196.61) |  |
|  | Parietal | Left | -339.54 (-373.99 – -311.42) | 0.7592 | -337.85 (-442.99 – -308.41) | 0.5834 | -342.61 (-373.99 – -317.79) | 0.8122 | -333.99 (-415.04 – -283.34) | 0.716 | -330.02 (-349.27 – -307.19) | 1 | -339.61 (-387.56 – -315.64) | 0.6442 |
|  |  | Right | -346.02 (-369.87 – -320.18) |  | -351.96 (-412.76 – -340.88) |  | -337.87 (-384.07 – -321.2) |  | -354.84 (-385.13 – -324.98) |  | -407.72 (-414.56 – -401.79) |  | -343.15 (-372.94 – -318.74) |  |
|  | Temporal | Left | -338.93 (-358.85 – -334.39) | 0.4513 | -403.66 (-522.18 – -379.75) | 0.5067 | -331.35 (-348.76 – -317.05) | 0.103 | -383.78 (-425.71 – -366.7) | 0.5652 | -724.76 (-733.86 – -693.39) | 1 | -354.97 (-367.17 – -335.64) | 0.3745 |
|  |  | Right | -305.69 (-324.02 – -268.62) |  | -330.15 (-356.85 – -309.39) |  | -279.36 (-294.59 – -263.4) |  | -505.11 (-557.76 – -467.24) |  | -330.32 (-473.12 – -267.09) |  | -306.33 (-320.69 – -274.1) |  |
|  | Occipital | Left | -762.24 (-795.7 – -754.71) | **<0.001** | -761.89 (-786.36 – -731.24) | **<0.001** | -790.66 (-797.65 – -779.7) | **<0.001** | -737.43 (-778.57 – -714.2) | **<0.001** | -735.13 (-763.1 – -707.09) | 1 | -762.33 (-795.03 – -754.05) | **<0.001** |
|  |  | Right | -351.34 (-389.51 – -317.74) |  | -399.27 (-427.28 – -362.77) |  | -324.68 (-355.39 – -300.69) |  | -414.55 (-486.69 – -378.5) |  | -464.25 (-498.19 – -438.71) |  | -351.8 (-390.15 – -333.89) |  |
| HbDiff (au) | Frontal | Left | -13.97 (-17.3 – -11.72) | **<0.001** | -26 (-28.95 – -22.78) | **<0.001** | -20.34 (-23.5 – -13.88) | **<0.001** | -17.76 (-20.72 – -15.61) | **<0.001** | -33.14 (-35.84 – -27.4) | 1 | -18.81 (-21.75 – -12.91) | **<0.001** |
|  |  | Right | -42.48 (-46.73 – -36.19) |  | -46.99 (-51.78 – -42.98) |  | -41.82 (-46.83 – -36.19) |  | -47.03 (-51.21 – -42.91) |  | -55.17 (-229.26 – -35.98) |  | -44.01 (-49.43 – -38.04) |  |
|  | Parietal | Left | -3.39 (-11.06 – 4.27) | **0.0202** | -12.41 (-26.78 – -3.94) | **0.0166** | 3.12 (-8.06 – 8.37) | **0.0047** | -12.41 (-26.78 – -5.77) | 0.2359 | 26.4 (5.57 – 39) | 1 | -9.62 (-18.86 – 2.41) | **0.0048** |
|  |  | Right | -24.82 (-32.95 – -18.54) |  | -32.16 (-38.28 – -26.19) |  | -32.36 (-40.58 – -23.48) |  | -24.82 (-32.87 – -10.18) |  | -45.74 (-63.67 – -39.22) |  | -28.88 (-35.12 – -18.81) |  |
|  | Temporal | Left | -55.47 (-63.28 – -50.54) | **<0.001** | -63.89 (-79.82 – -54.84) | **0.0141** | -60.06 (-72.07 – -53.07) | **<0.001** | -51.94 (-65.8 – -48.88) | 0.13 | -168.13 (-188.71 – -128.21) | 1 | -53.33 (-71.69 – -49.03) | **<0.001** |
|  |  | Right | -6.34 (-14.34 – -2.04) |  | -21.43 (-26.16 – -17.09) |  | -6.4 (-14.34 – -2.25) |  | -18.01 (-28.03 – -11.07) |  | -19.44 (-21.93 – -13.87) |  | -8.27 (-17.31 – -5.46) |  |
|  | Occipital | Left | -218.44 (-229.82 – -206.21) | **<0.001** | -220.08 (-237.38 – -202.55) | **<0.001** | -221.4 (-235.19 – -206.72) | **<0.001** | -217.55 (-228.05 – -202.23) | **<0.001** | -201.53 (-217.48 – -183.98) | 1 | -219 (-233.03 – -205.62) | **<0.001** |
|  |  | Right | -19.95 (-23.19 – -9.57) |  | -28.66 (-32.8 – -18.76) |  | -18.6 (-22.05 – -13.46) |  | -31.76 (-37.27 – -9.48) |  | -36.73 (-41.88 – -29.29) |  | -20.54 (-23.2 – -10.88) |  |
| MAD of ABP (mmHg) | – | – | 6.5 (5.74 – 7.28) | – | 6.44 (5.45 – 7.68) | – | 6.21 (5.56 – 6.66) | – | 6.8 (6.02 – 8.13) | – | 4.79 (4.79 – 4.79) | – | 6.5 (5.6 – 7.57) | – |
| MAD of EtCO₂ (mmHg) | – | – | 1 (1 – 1.52) | – | 1.52 (1 – 2) | – | 1.02 (1 – 2) | – | 1 (1 – 1.18) | – | 6 (6 – 6) | – | 1 (1 – 2) | – |
| MAD of RR (bpm) | – | – | 2 (2 – 3) | – | 2.5 (2 – 3.12) | – | 2 (2 – 3) | – | 2.3 (2 – 3) | – | 4 (4 – 4) | – | 2 (2 – 3) | – |
| MAD of rSO_2_ (%) | Frontal | Left | 0.84 (0.63 – 1.08) | 0.2823 | 0.65 (0.47 – 0.81) | 0.6236 | 0.76 (0.49 – 0.94) | 0.5281 | 0.83 (0.68 – 1.12) | 0.2855 | 0.74 (0.74 – 0.74) | 1 | 0.81 (0.51 – 0.97) | 0.2996 |
|  |  | Right | 0.91 (0.63 – 1.26) |  | 0.71 (0.5 – 0.97) |  | 0.76 (0.48 – 1.23) |  | 0.99 (0.78 – 1.17) |  | 2.2 (2.2 – 2.2) |  | 0.88 (0.57 – 1.18) |  |
|  | Parietal | Left | 1.27 (0.87 – 1.83) | 0.2032 | 1.25 (0.91 – 1.8) | 0.977 | 1.14 (0.75 – 1.87) | 0.4659 | 1.33 (1.05 – 1.8) | 0.5339 | 2.33 (2.33 – 2.33) | 1 | 1.25 (0.85 – 1.82) | 0.2963 |
|  |  | Right | 0.98 (0.69 – 1.68) |  | 1.44 (0.88 – 2.71) |  | 0.9 (0.71 – 1.56) |  | 1.19 (0.84 – 2.64) |  | 1.6 (1.6 – 1.6) |  | 1.04 (0.74 – 1.86) |  |
|  | Temporal | Left | 0.91 (0.6 – 1.56) | 0.5095 | 1.16 (0.87 – 1.62) | 0.8399 | 0.86 (0.61 – 1.32) | 0.6881 | 1.12 (0.82 – 1.78) | 0.7692 | 1.6 (1.6 – 1.6) | 1 | 0.96 (0.66 – 1.55) | 0.624 |
|  |  | Right | 1 (0.76 – 1.49) |  | 0.99 (0.67 – 1.86) |  | 0.93 (0.64 – 1.38) |  | 1.07 (1 – 1.77) |  | 0.91 (0.91 – 0.91) |  | 1.01 (0.68 – 1.73) |  |
|  | Occipital | Left | 0.86 (0.61 – 1) | 0.6739 | 0.97 (0.86 – 1.02) | 0.3408 | 0.85 (0.69 – 0.95) | 0.2348 | 0.97 (0.74 – 1.03) | 0.2359 | 0.97 (0.97 – 0.97) | 1 | 0.88 (0.69 – 1) | 0.949 |
|  |  | Right | 0.8 (0.55 – 1.41) |  | 0.74 (0.56 – 1.33) |  | 0.67 (0.47 – 1.03) |  | 1 (0.7 – 2.2) |  | 0.67 (0.67 – 0.67) |  | 0.81 (0.54 – 1.45) |  |
| MAD of HbO (au) | Frontal | Left | 4.18 (3.05 – 8.12) | 0.2917 | 4.29 (2.27 – 6.08) | 1 | 5.07 (3.28 – 8.73) | 0.583 | 3.83 (2.62 – 7.58) | 0.5035 | 4.3 (4.3 – 4.3) | 1 | 4.06 (2.89 – 7.75) | 0.5225 |
|  |  | Right | 5.22 (3.48 – 8.69) |  | 4.48 (2.16 – 7.17) |  | 6.05 (3.99 – 9.06) |  | 4.65 (3.06 – 5.87) |  | 69.35 (69.35 – 69.35) |  | 4.76 (3.15 – 8.24) |  |
|  | Parietal | Left | 11.94 (5.99 – 18.32) | 0.5784 | 14.54 (11.05 – 21.04) | 1 | 12.4 (5.83 – 19.51) | 0.583 | 13.26 (9 – 18.35) | 0.8787 | 5.87 (5.87 – 5.87) | 1 | 12.82 (6.36 – 18.99) | 0.5365 |
|  |  | Right | 9.5 (6.74 – 15.28) |  | 13.61 (9.7 – 32.68) |  | 10.05 (6.59 – 14.64) |  | 9.92 (7.9 – 29.13) |  | 8.16 (8.16 – 8.16) |  | 10.06 (7.06 – 17.36) |  |
|  | Temporal | Left | 8.93 (3.7 – 13.66) | 0.8152 | 11.46 (8.09 – 20.97) | 0.5834 | 8.74 (4.48 – 14.77) | 0.1516 | 10.05 (4.09 – 19.29) | 0.5495 | 17.67 (17.67 – 17.67) | 1 | 8.96 (3.89 – 16.93) | 0.5179 |
|  |  | Right | 8.1 (4.5 – 11.43) |  | 8.79 (3.87 – 17.61) |  | 6.57 (3.83 – 9.65) |  | 9.73 (6.01 – 22.13) |  | 33.69 (33.69 – 33.69) |  | 7.97 (4.07 – 11.58) |  |
|  | Occipital | Left | 10.07 (7.68 – 11.39) | 0.7831 | 10.94 (9.32 – 12.31) | 0.5834 | 10.14 (7.55 – 12.15) | 0.9412 | 9.4 (8.85 – 13.97) | 0.3538 | 18.54 (18.54 – 18.54) | 1 | 10.08 (8.53 – 12.13) | 0.599 |
|  |  | Right | 11 (5.35 – 20.78) |  | 16.84 (7.52 – 35.79) |  | 9.84 (5.92 – 15.54) |  | 18.05 (7.18 – 37.84) |  | 11.93 (11.93 – 11.93) |  | 11.21 (6.02 – 22.45) |  |
| MAD of HHb (au) | Frontal | Left | 3.65 (1.57 – 6.83) | 0.1155 | 3.05 (1.55 – 4.48) | 0.3708 | 2.68 (1.49 – 7.23) | 0.3215 | 3.65 (1.57 – 5.93) | 0.1489 | 2.19 (2.19 – 2.19) | 1 | 3.49 (1.56 – 6.38) | 0.1147 |
|  |  | Right | 4.58 (2.7 – 8.48) |  | 4.52 (2.96 – 7.13) |  | 4.49 (2.04 – 8.48) |  | 4.93 (3.35 – 7.47) |  | 91.41 (91.41 – 91.41) |  | 4.58 (2.62 – 8.13) |  |
|  | Parietal | Left | 12.25 (4.26 – 18.17) | 0.5365 | 13.79 (8.64 – 19.99) | 0.795 | 10.01 (5.17 – 17.68) | 0.4461 | 14.68 (4.81 – 19.33) | 1 | 17.01 (17.01 – 17.01) | 1 | 13.26 (4.75 – 19.47) | 0.7224 |
|  |  | Right | 8.53 (6.2 – 12.62) |  | 14.37 (5.88 – 29.67) |  | 7.51 (5.31 – 12.06) |  | 11.36 (7.62 – 23.15) |  | 4.32 (4.32 – 4.32) |  | 9.09 (6.18 – 15.74) |  |
|  | Temporal | Left | 6.71 (3.91 – 11.67) | 0.7751 | 10.18 (5.64 – 14.72) | 0.7508 | 6.48 (3.37 – 11.33) | 0.7618 | 8.16 (6.21 – 14.87) | 0.6304 | 8.12 (8.12 – 8.12) | 1 | 6.75 (4.06 – 12.75) | 0.4556 |
|  |  | Right | 6.48 (4.64 – 8.85) |  | 6.39 (5.07 – 14.18) |  | 6.39 (3.63 – 8.8) |  | 7.14 (5.47 – 12.3) |  | 27.32 (27.32 – 27.32) |  | 6.4 (4.53 – 8.87) |  |
|  | Occipital | Left | 7.27 (6.17 – 8.38) | 0.1062 | 6.75 (6.32 – 9.06) | 0.795 | 7.27 (6.07 – 8.48) | 0.5175 | 6.91 (6.3 – 9.06) | 0.098 | 9.2 (9.2 – 9.2) | 1 | 7.24 (6.23 – 8.71) | 0.1574 |
|  |  | Right | 9.67 (4.34 – 15.24) |  | 9.84 (4.07 – 17.54) |  | 7.33 (4.09 – 14.28) |  | 11.54 (6.15 – 17.26) |  | 13.8 (13.8 – 13.8) |  | 9.51 (4.29 – 15.81) |  |
| MAD of tHb (au) | Frontal | Left | 6.8 (4.34 – 13.48) | 0.2384 | 7.56 (2.64 – 8.94) | 0.5834 | 7.19 (4.34 – 13.32) | 0.6641 | 6.07 (2.72 – 11.05) | 0.1556 | 6.41 (6.41 – 6.41) | 1 | 6.93 (3.44 – 12.04) | 0.3303 |
|  |  | Right | 7.5 (5.36 – 14.73) |  | 7.66 (3.84 – 11.9) |  | 7.5 (4.96 – 16.39) |  | 7.84 (5.33 – 12.3) |  | 160.42 (160.42 – 160.42) |  | 7.35 (5.11 – 14.69) |  |
|  | Parietal | Left | 18.07 (7.87 – 35.04) | 0.4328 | 29.79 (18.69 – 40.82) | 0.931 | 19.19 (8.36 – 36.03) | 0.2348 | 30.62 (6.85 – 40.19) | 0.9532 | 19.97 (19.97 – 19.97) | 1 | 25.56 (7.62 – 40.44) | 0.4139 |
|  |  | Right | 13.62 (8.05 – 26.44) |  | 27.25 (6.99 – 58.72) |  | 12.34 (5.96 – 25.27) |  | 17.37 (9.82 – 49.88) |  | 6.58 (6.58 – 6.58) |  | 17.09 (7.47 – 33.76) |  |
|  | Temporal | Left | 11.89 (5.84 – 18.59) | 0.6071 | 17.25 (6.98 – 26.02) | 0.8399 | 12.15 (5.6 – 20.64) | 0.9412 | 13.14 (6.18 – 24.28) | 0.7513 | 11.98 (11.98 – 11.98) | 1 | 12.83 (5.75 – 21.99) | 0.9943 |
|  |  | Right | 12.75 (7.14 – 16.77) |  | 8.6 (6.67 – 21) |  | 11.77 (6.86 – 15.29) |  | 12.41 (6.36 – 26.97) |  | 68.97 (68.97 – 68.97) |  | 11.49 (6.84 – 17.12) |  |
|  | Occipital | Left | 9.18 (5.5 – 15.24) | **0.0064** | 7.63 (6.06 – 15.08) | **0.0073** | 8.71 (6.59 – 15.08) | **0.0081** | 8.48 (5.36 – 16.74) | **0.0083** | 27.98 (27.98 – 27.98) | 1 | 8.46 (5.58 – 14.91) | **<0.001** |
|  |  | Right | 17.26 (7.32 – 36.37) |  | 28.29 (10.73 – 39.91) |  | 17.09 (9.21 – 30.83) |  | 27 (7.94 – 41.2) |  | 27.67 (27.67 – 27.67) |  | 17.52 (8.61 – 36.83) |  |
| MAD of HbDiff (au) | Frontal | Left | 3.71 (2.57 – 5.44) | 0.2869 | 3.49 (2.15 – 4.58) | 0.795 | 3.93 (2.48 – 5.31) | 0.6057 | 3.47 (2.47 – 4.73) | 0.6138 | 3.95 (3.95 – 3.95) | 1 | 3.5 (2.44 – 5.24) | 0.4907 |
|  |  | Right | 4.38 (2.76 – 7.1) |  | 2.96 (1.84 – 4.48) |  | 4.13 (2.74 – 6.36) |  | 4.02 (2.49 – 6.31) |  | 25.25 (25.25 – 25.25) |  | 4.11 (2.69 – 6.06) |  |
|  | Parietal | Left | 8.47 (5.49 – 13.6) | 0.5229 | 7.62 (5.67 – 12.27) | 0.3123 | 7.52 (4.26 – 12.88) | 0.8634 | 8.47 (7.3 – 13.44) | 0.9906 | 15.86 (15.86 – 15.86) | 1 | 8.04 (5.32 – 13.44) | 0.9773 |
|  |  | Right | 7.24 (4.4 – 14.31) |  | 17.46 (7.37 – 19.1) |  | 7.87 (5.55 – 13.79) |  | 12.06 (4.4 – 17.99) |  | 12.1 (12.1 – 12.1) |  | 8.21 (4.58 – 17.55) |  |
|  | Temporal | Left | 6.5 (3.27 – 13.13) | 0.4207 | 9.28 (5.05 – 15.34) | 0.7508 | 5.88 (3.57 – 13.73) | 0.7618 | 8.03 (3.28 – 14.48) | 0.2751 | 25.26 (25.26 – 25.26) | 1 | 6.24 (3.47 – 13.97) | 0.4599 |
|  |  | Right | 7.86 (3.6 – 15.23) |  | 7.63 (3.09 – 14.98) |  | 5.32 (3.32 – 10.96) |  | 14.97 (5.03 – 19.24) |  | 3.34 (3.34 – 3.34) |  | 8.33 (3.56 – 15.13) |  |
|  | Occipital | Left | 14.39 (10.95 – 15.21) | **<0.001** | 15.45 (14.2 – 16.35) | **0.0404** | 14.59 (11.58 – 16.22) | **<0.001** | 14.44 (12.33 – 15.27) | 0.4047 | 16.7 (16.7 – 16.7) | 1 | 14.47 (11.75 – 15.73) | **<0.001** |
|  |  | Right | 6.02 (3.77 – 12.99) |  | 5.84 (3.63 – 18.16) |  | 4.64 (3.64 – 6.34) |  | 12.93 (4.2 – 28.62) |  | 6.12 (6.12 – 6.12) |  | 6.01 (3.66 – 14.19) |  |
| **250 Hz Sampled Data** | | | | | | | | | | | | | | |
| ABP (mmHg) | – | – | 92.7 (80.51 – 108.26) | – | 92.48 (80.49 – 111.48) | – | 90.85 (80.26 – 105.59) | – | 93.67 (80.87 – 109.94) | – | 79.19 (70.71 – 90.85) | – | 92.74 (80.51 – 108.4) | – |
| EtCO₂ (mmHg) | – | – | 35.5 (34.5 – 37) | – | 35.5 (33 – 37) | – | 36 (34 – 38) | – | 35 (34 – 36) | – | 29 (21 – 35) | – | 36 (34 – 37) | – |
| RR (bpm) | – | – | 18 (15 – 20.5) | – | 16 (13 – 18.5) | – | 17.5 (15 – 20) | – | 18.5 (15 – 21.5) | – | 15 (11 – 19) | – | 18 (15 – 20) | – |
| rSO_2_ (%) | Frontal | Left | 46.24 (44.81 – 47.11) | **<0.001** | 44.37 (43.9 – 44.79) | **<0.001** | 45.31 (44.63 – 46.47) | **<0.001** | 45.23 (43.92 – 46.03) | **<0.001** | 43.1 (42.55 – 44.12) | 1 | 45.38 (44.56 – 46.13) | **<0.001** |
|  |  | Right | 40.9 (39.69 – 42.33) |  | 39.4 (38.99 – 40.38) |  | 41.18 (40.22 – 42.09) |  | 39.04 (37.61 – 40.76) |  | 42.74 (35.85 – 44.55) |  | 40.74 (39.39 – 41.23) |  |
|  | Parietal | Left | 49.33 (46.73 – 51.62) | **0.0319** | 47.45 (44.91 – 50.44) | **0.0194** | 50.28 (47.53 – 52.01) | **0.0067** | 47.46 (44.91 – 50.45) | 0.2855 | 53.17 (47.99 – 58.7) | 1 | 48.29 (45.97 – 51.36) | **0.0057** |
|  |  | Right | 45.98 (44.25 – 47.25) |  | 44.81 (43.12 – 47.67) |  | 45.52 (42.98 – 46.72) |  | 45.97 (44.55 – 49.41) |  | 44.37 (42.09 – 45.31) |  | 45.63 (44.08 – 47.38) |  |
|  | Temporal | Left | 40.32 (37.96 – 42.59) | **<0.001** | 40.79 (38.25 – 43.16) | **0.0086** | 39.72 (37.84 – 42.21) | **<0.001** | 41.44 (38.71 – 44.34) | 0.113 | 39.06 (34.03 – 46.29) | 1 | 40.45 (38.13 – 42.95) | **<0.001** |
|  |  | Right | 48.63 (46.41 – 49.62) |  | 45.89 (45.13 – 46.71) |  | 48.49 (46.52 – 49.55) |  | 46.35 (44.9 – 47.48) |  | 47.12 (45.96 – 48.61) |  | 48.01 (46.16 – 48.94) |  |
|  | Occipital | Left | 36.37 (30.34 – 41.33) | **<0.001** | 36.46 (29.71 – 41.47) | **<0.001** | 36.38 (30.2 – 41.02) | **<0.001** | 36.35 (30.03 – 42.12) | **<0.001** | 36.38 (30.49 – 42.89) | 1 | 36.38 (30.12 – 41.21) | **<0.001** |
|  |  | Right | 47.02 (45.72 – 48.48) |  | 45.42 (44.45 – 48.13) |  | 46.99 (46.37 – 48.23) |  | 46.59 (44.36 – 49.52) |  | 46.05 (44.5 – 47.48) |  | 46.86 (45.71 – 48.46) |  |
| HbO (au) | Frontal | Left | -118.47 (-127.86 – -113.18) | 0.0934 | -125.84 (-128.61 – -119.98) | 0.5444 | -129.83 (-139.74 – -123.14) | 0.5718 | -110.26 (-114.94 – -99.41) | 0.0528 | -136.56 (-139.74 – -127.79) | 1 | -119.16 (-125.55 – -114.06) | 0.0922 |
|  |  | Right | -128.44 (-137.61 – -119.36) |  | -129.31 (-134.62 – -127.06) |  | -133.81 (-142.13 – -127.11) |  | -127.6 (-136.46 – -120.58) |  | -193.7 (-559.67 – -165.71) |  | -126.67 (-136.72 – -121.8) |  |
|  | Parietal | Left | -173.31 (-193.07 – -156.58) | 0.2641 | -162.37 (-222.62 – -144.66) | 0.3123 | -172.06 (-187.37 – -158.63) | 0.1983 | -176.88 (-205.62 – -148.27) | 0.5339 | -157.56 (-176.98 – -130.3) | 1 | -173.07 (-195.58 – -158.52) | 0.2009 |
|  |  | Right | -183.51 (-199.4 – -170.26) |  | -186.74 (-216.72 – -173.43) |  | -192.7 (-208.23 – -174.34) |  | -179.73 (-204.77 – -167.69) |  | -230.53 (-247.11 – -224.72) |  | -182.26 (-203 – -170.2) |  |
|  | Temporal | Left | -203.85 (-212 – -193.9) | 0.0699 | -243.19 (-280.97 – -229.39) | 0.1748 | -198.91 (-209.64 – -187.2) | **0.0019** | -226.75 (-248.14 – -221.58) | 0.9532 | -415.44 (-509.54 – -332.77) | 1 | -206.38 (-222.33 – -193.93) | **0.0286** |
|  |  | Right | -152.27 (-162.34 – -139.48) |  | -167.11 (-182.54 – -143.52) |  | -145.81 (-157.91 – -134.24) |  | -234.02 (-278.18 – -203.51) |  | -172.48 (-241.06 – -146.49) |  | -154.73 (-160.67 – -135.72) |  |
|  | Occipital | Left | -508.46 (-547.71 – -440.79) | **<0.001** | -511.36 (-543.54 – -420.31) | **<0.001** | -521.76 (-550.52 – -450.1) | **<0.001** | -496.1 (-541.65 – -420.07) | **<0.001** | -488.89 (-525.19 – -400.36) | 1 | -511.55 (-547.66 – -435.15) | **<0.001** |
|  |  | Right | -181.96 (-206.48 – -158.3) |  | -202.45 (-223.91 – -187.47) |  | -169.09 (-187.17 – -158.3) |  | -212.15 (-237.63 – -196.05) |  | -248.63 (-267.34 – -235.55) |  | -185.61 (-206.66 – -166.01) |  |
| HHb (au) | Frontal | Left | -113.52 (-117.59 – -110.28) | 0.2302 | -97.44 (-102.71 – -91.87) | 0.1572 | -114.42 (-117.59 – -112.58) | 0.1065 | -103.75 (-110.02 – -89.78) | 0.3787 | -101.64 (-103.62 – -99.33) | 1 | -110.99 (-111.64 – -107.85) | 0.0569 |
|  |  | Right | -87.31 (-94.54 – -78.58) |  | -82.67 (-91.41 – -77.12) |  | -88.67 (-100.57 – -81.87) |  | -81.3 (-87.34 – -72.5) |  | -160.75 (-315.16 – -129.73) |  | -85.59 (-92.75 – -77.52) |  |
|  | Parietal | Left | -174.17 (-196.17 – -156.46) | 0.5998 | -175.4 (-213.71 – -155.51) | 0.931 | -176.01 (-196.17 – -158.03) | 0.5069 | -159.63 (-201.23 – -141.32) | 0.9906 | -175.84 (-196.91 – -157.22) | 1 | -172.5 (-200.41 – -155.69) | 0.6699 |
|  |  | Right | -163.28 (-183.33 – -146.06) |  | -169.3 (-203.21 – -155.71) |  | -158.77 (-179.44 – -146.35) |  | -168.1 (-192.92 – -153.61) |  | -182.29 (-185.52 – -169.64) |  | -160.75 (-183.9 – -145.93) |  |
|  | Temporal | Left | -134.91 (-150.87 – -132.3) | 0.3688 | -161.08 (-222.34 – -156.67) | 1 | -134.47 (-144.5 – -130.91) | 0.9543 | -151.59 (-173.95 – -141.67) | 0.177 | -278.46 (-319.98 – -219.55) | 1 | -140.51 (-157.92 – -133.2) | 0.3744 |
|  |  | Right | -145.89 (-158.3 – -130.18) |  | -162.73 (-174.53 – -145.91) |  | -135.23 (-144.99 – -127.41) |  | -246.06 (-277.87 – -206.48) |  | -148.2 (-229.6 – -124.95) |  | -150.35 (-158.34 – -133.16) |  |
|  | Occipital | Left | -281.51 (-310.41 – -228.12) | **<0.001** | -284.01 (-307.09 – -222.51) | **<0.001** | -291.9 (-315.25 – -235.37) | **<0.001** | -274.87 (-307.4 – -222.52) | **<0.001** | -280.99 (-307.41 – -221.33) | 1 | -283.23 (-309.86 – -228.02) | **<0.001** |
|  |  | Right | -160.4 (-180.45 – -151.33) |  | -189.95 (-210.86 – -176.4) |  | -156.61 (-164.73 – -142.5) |  | -198.11 (-233.1 – -179.31) |  | -213.32 (-233.67 – -200.67) |  | -162.22 (-181.42 – -154.37) |  |
| tHb (au) | Frontal | Left | -228.14 (-242.88 – -212.96) | 0.9627 | -226.99 (-240.58 – -215.29) | 0.5067 | -242.15 (-245.87 – -232.28) | 0.4659 | -207.15 (-226.98 – -199.41) | 0.7336 | -239.28 (-243.04 – -227.37) | 1 | -226.38 (-241.59 – -210.61) | 0.624 |
|  |  | Right | -216.9 (-232.08 – -198.87) |  | -215.15 (-226.84 – -204.76) |  | -224.38 (-242.82 – -205.97) |  | -212.28 (-221.73 – -197.66) |  | -353.43 (-844.68 – -296.62) |  | -216.66 (-229.07 – -196.56) |  |
|  | Parietal | Left | -339.97 (-374.11 – -312.12) | 0.7278 | -337.75 (-441.95 – -307.33) | 0.5834 | -342.5 (-374.11 – -317.95) | 0.7995 | -334.61 (-415.46 – -286.59) | 0.7336 | -327.68 (-354.7 – -308.37) | 1 | -340.36 (-389.17 – -315.87) | 0.629 |
|  |  | Right | -346.01 (-369.79 – -320.4) |  | -351.94 (-413.84 – -340.74) |  | -338.43 (-384.37 – -321.38) |  | -354.8 (-386.11 – -325.01) |  | -408.19 (-415.51 – -401.61) |  | -343.16 (-373.62 – -318.65) |  |
|  | Temporal | Left | -338.97 (-358.87 – -334.33) | 0.4832 | -402.83 (-521.13 – -379.85) | 0.5444 | -331.39 (-348.63 – -316.91) | 0.1101 | -383.88 (-425.85 – -366.89) | 0.5186 | -701.98 (-766 – -650.82) | 1 | -355.01 (-368.27 – -335.68) | 0.3899 |
|  |  | Right | -304.14 (-324.03 – -270.91) |  | -330.05 (-357.38 – -309.51) |  | -280.09 (-294.28 – -263.36) |  | -499.82 (-562.58 – -460.71) |  | -329.93 (-472.28 – -267.73) |  | -306.33 (-320.7 – -275.25) |  |
|  | Occipital | Left | -772.42 (-801.85 – -736.88) | **<0.001** | -763.17 (-803.17 – -715.04) | **<0.001** | -795.12 (-824.59 – -753.1) | **<0.001** | -749.43 (-787 – -705.06) | **<0.001** | -740.23 (-780.72 – -693.88) | 1 | -767.72 (-806.45 – -736.17) | **<0.001** |
|  |  | Right | -351.5 (-389.9 – -316.15) |  | -397.36 (-428.35 – -363.05) |  | -324.51 (-355.6 – -300.68) |  | -413.23 (-483.24 – -378.77) |  | -463.32 (-500.19 – -438.77) |  | -351.98 (-390.24 – -333.64) |  |
| HbDiff (au) | Frontal | Left | -14.05 (-18.94 – -11.61) | **<0.001** | -25.98 (-28.99 – -22.74) | **<0.001** | -20.37 (-23.81 – -13.85) | **<0.001** | -17.76 (-22.61 – -15.53) | **<0.001** | -33.09 (-35.88 – -27.27) | 1 | -18.92 (-22.38 – -12.92) | **<0.001** |
|  |  | Right | -42.31 (-46.92 – -35.99) |  | -47.03 (-51.66 – -42.99) |  | -41.66 (-47.29 – -35.99) |  | -47.08 (-51.84 – -42.88) |  | -53.11 (-239.96 – -34.96) |  | -44.01 (-49.92 – -38.81) |  |
|  | Parietal | Left | -3.83 (-17.72 – 10.09) | **0.0295** | -12.68 (-36.15 – 2.39) | **0.0351** | 1.83 (-14.1 – 12.44) | **0.007** | -13.06 (-37.61 – 3.73) | 0.3787 | 21.3 (-13.49 – 56.04) | 1 | -11.96 (-23.06 – 9.91) | **0.0099** |
|  |  | Right | -25.76 (-37.07 – -16.84) |  | -32.21 (-38.39 – -15.06) |  | -31.72 (-43.37 – -22.46) |  | -26.45 (-33 – -5.5) |  | -46.69 (-64.66 – -38.96) |  | -28.69 (-37.18 – -15.19) |  |
|  | Temporal | Left | -60.4 (-69.29 – -47.55) | **<0.001** | -65.07 (-78.23 – -48.86) | **0.0166** | -65.16 (-73.13 – -50.73) | **<0.001** | -53.39 (-70.04 – -43.71) | 0.1554 | -143.09 (-235.31 – -47.02) | 1 | -57.87 (-72.88 – -48.14) | **<0.001** |
|  |  | Right | -7.99 (-20.56 – -2.05) |  | -21.67 (-26.25 – -17.04) |  | -7.21 (-19.75 – -2.26) |  | -21.41 (-32.35 – -11.01) |  | -18.42 (-24.99 – -10.87) |  | -13.38 (-24.37 – -5.3) |  |
|  | Occipital | Left | -226.7 (-302.69 – -129.34) | **<0.001** | -234.77 (-311.39 – -121.04) | **<0.001** | -230.1 (-310.98 – -131.1) | **<0.001** | -222.2 (-295.05 – -118.43) | **<0.001** | -215.84 (-289.47 – -100.56) | 1 | -229.22 (-310.04 – -126.52) | **<0.001** |
|  |  | Right | -18.46 (-27.5 – -9.41) |  | -28.65 (-32.83 – -12.81) |  | -18.66 (-24.57 – -12.14) |  | -25.06 (-41.46 – -4.51) |  | -36.11 (-51.91 – -23.07) |  | -19.57 (-27.9 – -9.86) |  |
| MAD of ABP (mmHg) | – | – | 12.83 (11.71 – 13.98) | – | 14.19 (13.43 – 15.34) | – | 12.34 (11.38 – 13.69) | – | 13.72 (12.99 – 15.23) | – | 9.58 (9.58 – 9.58) | – | 13.34 (11.99 – 14.4) | – |
| MAD of EtCO₂ (mmHg) | – | – | 1 (1 – 2) | – | 1.5 (1 – 2) | – | 1 (1 – 2) | – | 1 (1 – 1) | – | 6 (6 – 6) | – | 1 (1 – 2) | – |
| MAD of RR (bpm) | – | – | 2 (2 – 3) | – | 2.5 (2 – 3) | – | 2 (2 – 3) | – | 2 (2 – 3) | – | 4 (4 – 4) | – | 2 (2 – 3) | – |
| MAD of rSO_2_ (%) | Frontal | Left | 0.88 (0.66 – 1.18) | 0.2641 | 0.65 (0.46 – 0.87) | 0.7075 | 0.8 (0.48 – 0.99) | 0.6405 | 0.86 (0.69 – 1.18) | 0.2178 | 0.75 (0.75 – 0.75) | 1 | 0.84 (0.56 – 1.11) | 0.3029 |
|  |  | Right | 0.95 (0.65 – 1.7) |  | 0.77 (0.51 – 0.97) |  | 0.83 (0.49 – 1.32) |  | 0.99 (0.87 – 1.61) |  | 4.4 (4.4 – 4.4) |  | 0.91 (0.6 – 1.35) |  |
|  | Parietal | Left | 3.77 (1.4 – 5.33) | **0.0162** | 2.82 (1.94 – 3.69) | 0.4705 | 2.87 (1.37 – 5.02) | 0.0996 | 3.27 (1.78 – 5.18) | **0.0423** | 5.35 (5.35 – 5.35) | 1 | 3.08 (1.56 – 4.95) | **0.0148** |
|  |  | Right | 1.28 (0.84 – 3.51) |  | 2.62 (1.34 – 4.29) |  | 1.48 (0.86 – 4.22) |  | 1.9 (0.87 – 3.3) |  | 1.5 (1.5 – 1.5) |  | 1.76 (0.86 – 4.11) |  |
|  | Temporal | Left | 1.4 (0.75 – 4.56) | 0.8395 | 1.57 (0.91 – 4.97) | 0.6236 | 1.28 (0.74 – 5.13) | 0.5175 | 1.85 (1.2 – 4.18) | 0.4455 | 6.37 (6.37 – 6.37) | 1 | 1.38 (0.76 – 4.63) | 0.9434 |
|  |  | Right | 1.39 (0.85 – 4.9) |  | 1.13 (0.68 – 5.98) |  | 1 (0.68 – 1.81) |  | 4.83 (1.1 – 6.4) |  | 1.3 (1.3 – 1.3) |  | 1.37 (0.76 – 5.68) |  |
|  | Occipital | Left | 5.08 (4.27 – 6.32) | **<0.001** | 5.93 (4.33 – 6.17) | 0.1124 | 4.95 (4.22 – 6) | **<0.001** | 6.05 (4.45 – 6.43) | 0.4455 | 6.24 (6.24 – 6.24) | 1 | 5.21 (4.25 – 6.24) | **<0.001** |
|  |  | Right | 1.31 (0.69 – 5.11) |  | 1.56 (0.57 – 4.57) |  | 0.79 (0.57 – 1.87) |  | 3.3 (1.18 – 6.57) |  | 1.49 (1.49 – 1.49) |  | 1.31 (0.67 – 5.55) |  |
| MAD of HbO (au) | Frontal | Left | 4.2 (3.04 – 8.08) | 0.2262 | 4.26 (2.27 – 6.47) | 1 | 5.13 (3.32 – 8.66) | 0.5498 | 3.89 (2.63 – 7.56) | 0.3538 | 4.38 (4.38 – 4.38) | 1 | 4.07 (2.95 – 7.76) | 0.4098 |
|  |  | Right | 5.48 (3.52 – 10.77) |  | 4.48 (2.28 – 6.82) |  | 6.32 (4.02 – 9.27) |  | 4.66 (3.03 – 7.57) |  | 69.56 (69.56 – 69.56) |  | 5.39 (3.15 – 8.75) |  |
|  | Parietal | Left | 17.84 (10.34 – 44.3) | 0.128 | 19.78 (13.85 – 30.98) | 0.931 | 16.38 (12.73 – 41.83) | 0.2548 | 20.32 (10.47 – 34.07) | 0.474 | 22.45 (22.45 – 22.45) | 1 | 17.93 (12.73 – 40.59) | 0.1984 |
|  |  | Right | 12.62 (7.72 – 31.14) |  | 27.75 (11.74 – 37.02) |  | 14.63 (8.69 – 34.07) |  | 14.12 (7.83 – 34.66) |  | 8.64 (8.64 – 8.64) |  | 15.64 (8.71 – 35.02) |  |
|  | Temporal | Left | 10.64 (4.37 – 28.28) | 0.7592 | 22.65 (8.69 – 39.75) | 0.5834 | 10.12 (6.22 – 32.68) | 0.0781 | 12.7 (6.06 – 33.13) | 0.3662 | 91.08 (91.08 – 91.08) | 1 | 10.83 (5.3 – 33.07) | 0.5554 |
|  |  | Right | 9.26 (4.42 – 32.75) |  | 11.24 (3.93 – 48.96) |  | 7.04 (3.9 – 10.89) |  | 32.47 (5.99 – 58.76) |  | 32.39 (32.39 – 32.39) |  | 8.55 (4.1 – 36.16) |  |
|  | Occipital | Left | 56.47 (43.08 – 61.02) | **<0.001** | 59.06 (51.01 – 60.33) | 0.0531 | 56.47 (43.42 – 60.48) | **<0.001** | 58.92 (50.68 – 61.67) | 0.13 | 60.54 (60.54 – 60.54) | 1 | 57 (43.66 – 61.15) | **<0.001** |
|  |  | Right | 14.22 (7.24 – 31.25) |  | 16.58 (7.53 – 42.36) |  | 12.17 (6.58 – 19.08) |  | 28.11 (8.83 – 64.63) |  | 14.83 (14.83 – 14.83) |  | 13.6 (7.19 – 34.59) |  |
| MAD of HHb (au) | Frontal | Left | 3.69 (1.58 – 7.69) | 0.136 | 3.08 (1.55 – 4.72) | 0.3408 | 2.71 (1.51 – 7.94) | 0.3545 | 3.69 (1.58 – 6.12) | 0.1697 | 2.18 (2.18 – 2.18) | 1 | 3.53 (1.57 – 6.98) | 0.1394 |
|  |  | Right | 4.54 (2.74 – 9.05) |  | 4.5 (2.98 – 7.23) |  | 4.42 (2.41 – 8.66) |  | 4.96 (3.38 – 8.55) |  | 75.83 (75.83 – 75.83) |  | 4.54 (2.65 – 8.61) |  |
|  | Parietal | Left | 19.32 (9.97 – 30.77) | 0.1108 | 18.82 (11.15 – 34.89) | 0.7075 | 19.88 (9.82 – 31.69) | 0.1425 | 18.78 (12.46 – 26.16) | 0.6641 | 19.8 (19.8 – 19.8) | 1 | 18.85 (9.84 – 31.57) | 0.2271 |
|  |  | Right | 11.27 (7.14 – 25.86) |  | 27.76 (12.02 – 38.2) |  | 11.8 (6.35 – 27.7) |  | 15.4 (8.51 – 28.8) |  | 5.52 (5.52 – 5.52) |  | 14.81 (7.36 – 28.36) |  |
|  | Temporal | Left | 11.67 (5.22 – 28.83) | 0.7831 | 15.02 (8.85 – 34.16) | 0.931 | 10.61 (5.43 – 34.06) | 0.2041 | 15.02 (7.8 – 31.17) | 0.2267 | 52.29 (52.29 – 52.29) | 1 | 11.88 (5.61 – 31.72) | 0.7872 |
|  |  | Right | 8.7 (4.69 – 37.49) |  | 14.76 (5 – 41.62) |  | 6.75 (3.63 – 10.69) |  | 37.34 (6.23 – 43.07) |  | 28.8 (28.8 – 28.8) |  | 8.67 (4.56 – 39.41) |  |
|  | Occipital | Left | 40.8 (31.63 – 42.24) | **<0.001** | 41.41 (40.19 – 42.06) | **0.0464** | 40.71 (31.26 – 42.06) | **<0.001** | 41.36 (36.1 – 42.2) | 0.0588 | 41.38 (41.38 – 41.38) | 1 | 40.83 (33.65 – 42.2) | **<0.001** |
|  |  | Right | 14.07 (5.42 – 29.11) |  | 18.33 (4.02 – 33.71) |  | 10.5 (4.41 – 17.89) |  | 27.64 (10.12 – 43.2) |  | 15.23 (15.23 – 15.23) |  | 14.77 (4.55 – 30.41) |  |
| MAD of tHb (au) | Frontal | Left | 6.88 (4.34 – 17.2) | 0.2965 | 7.6 (2.66 – 9.12) | 0.5834 | 7.24 (4.35 – 18.26) | 0.7869 | 6.84 (2.73 – 13.46) | 0.1625 | 6.42 (6.42 – 6.42) | 1 | 7.18 (3.46 – 14.24) | 0.386 |
|  |  | Right | 8.37 (5.57 – 16.09) |  | 7.75 (3.85 – 11.81) |  | 8.01 (4.96 – 16.42) |  | 8.69 (5.57 – 15) |  | 160.51 (160.51 – 160.51) |  | 8.22 (5.27 – 15.25) |  |
|  | Parietal | Left | 27.91 (15.44 – 44.01) | 0.1687 | 29.61 (21.98 – 44.39) | 0.8852 | 25.81 (15.93 – 46.38) | 0.1338 | 31.69 (12.83 – 41.58) | 0.8419 | 23.27 (23.27 – 23.27) | 1 | 28.17 (15.59 – 45) | 0.2217 |
|  |  | Right | 21.21 (11.44 – 29.51) |  | 31.26 (13.25 – 56.1) |  | 21.21 (10.93 – 29.03) |  | 24.22 (12.96 – 47.41) |  | 6.97 (6.97 – 6.97) |  | 22.4 (12.06 – 36.51) |  |
|  | Temporal | Left | 18.6 (7.24 – 34.14) | 0.8967 | 26.87 (18.64 – 38.27) | 0.7508 | 20.91 (8.78 – 34.9) | 0.216 | 24.43 (8.37 – 34.14) | 0.2855 | 59.31 (59.31 – 59.31) | 1 | 21.68 (8.03 – 34.41) | 0.859 |
|  |  | Right | 15.4 (9.95 – 34.28) |  | 22.16 (7.88 – 41.51) |  | 13.72 (8.02 – 18.2) |  | 33.55 (11.36 – 38.91) |  | 68.65 (68.65 – 68.65) |  | 15.14 (8.32 – 34.62) |  |
|  | Occipital | Left | 34.69 (26.67 – 38.22) | 0.0502 | 36.31 (29.17 – 41.35) | 0.931 | 33.71 (26.99 – 38.66) | **0.0047** | 35.57 (30.02 – 38.73) | 0.5974 | 42.72 (42.72 – 42.72) | 1 | 35.08 (27.15 – 38.23) | 0.095 |
|  |  | Right | 25.51 (10.45 – 40.63) |  | 35.2 (10.73 – 60.97) |  | 18.83 (9.77 – 30.98) |  | 38.07 (14.78 – 69.57) |  | 27.39 (27.39 – 27.39) |  | 26.17 (10.19 – 52.43) |  |
| MAD of HbDiff (au) | Frontal | Left | 3.85 (2.49 – 5.37) | 0.2641 | 3.64 (2.18 – 4.73) | 0.7508 | 3.94 (2.5 – 5.49) | 0.583 | 3.47 (2.43 – 4.76) | 0.5652 | 4 (4 – 4) | 1 | 3.8 (2.42 – 5.38) | 0.4556 |
|  |  | Right | 4.38 (2.83 – 7.93) |  | 3.04 (1.93 – 4.48) |  | 4.13 (2.78 – 6.46) |  | 4.09 (2.48 – 7.26) |  | 22.46 (22.46 – 22.46) |  | 4.13 (2.69 – 6.46) |  |
|  | Parietal | Left | 19.9 (7.48 – 55.43) | 0.0683 | 15.42 (8.02 – 40.7) | 0.977 | 19.4 (6.86 – 64.32) | 0.2831 | 22.06 (7.86 – 33.58) | 0.1489 | 34.77 (34.77 – 34.77) | 1 | 19.44 (7.42 – 60.37) | 0.1052 |
|  |  | Right | 9.86 (5.15 – 28.49) |  | 17.21 (7.28 – 40.75) |  | 10.43 (6.48 – 36.35) |  | 14.36 (4.85 – 20.86) |  | 11.69 (11.69 – 11.69) |  | 10.93 (5.95 – 33.49) |  |
|  | Temporal | Left | 7.83 (3.6 – 33.74) | 0.9296 | 9.76 (4.93 – 62.86) | 0.6236 | 6.84 (4.01 – 45.29) | 0.4268 | 10.7 (3.95 – 47.13) | 0.4887 | 94.06 (94.06 – 94.06) | 1 | 7.63 (3.61 – 36.43) | 0.9887 |
|  |  | Right | 8.2 (4.25 – 56.27) |  | 6.2 (3.1 – 63.2) |  | 5.86 (3.25 – 13.4) |  | 58.38 (5.48 – 91.22) |  | 6.99 (6.99 – 6.99) |  | 7.51 (3.58 – 61.48) |  |
|  | Occipital | Left | 86.06 (68.87 – 95.24) | **<0.001** | 91.45 (82.19 – 93.65) | **0.0141** | 86.01 (69.19 – 93.56) | **<0.001** | 91.57 (78.52 – 95.24) | **0.0142** | 95.37 (95.37 – 95.37) | 1 | 87.5 (71.1 – 95.24) | **<0.001** |
|  |  | Right | 8.66 (4.38 – 33.04) |  | 12.35 (3.64 – 54.05) |  | 5.71 (3.92 – 13.54) |  | 27.1 (8.32 – 93.7) |  | 14.25 (14.25 – 14.25) |  | 8.81 (4.04 – 35.99) |  |
| The p-values in the table are derived using Mann-Whitney U test between the bilateral signals. *ABP, arterial blood pressure; bpm, beats per minute; EtCO_2_, end-tidal carbon dioxide; HbDiff, hemoglobin difference; HbO, oxyhemoglobin; HHb, deoxyhemoglobin; IQR, interquartile range; MAD, median absolute deviation; mmHg, millimeters of mercury; RR, respiratory rate; rSO_2_, regional cerebral oxygen saturation; tHb, total hemoglobin.* | | | | | | | | | | | | | | |

Appendix S6e: Subgrouped Percent Time Results of rSO_2_ and CVR Indices Using 10-Second Decimated Data

| **Physiologic Variable** | **Brain Lobe** | **Hemisphere** | **Subgroups** | | | | | | | | | | | |
| --- | --- | --- | --- | --- | --- | --- | --- | --- | --- | --- | --- | --- | --- | --- |
|  |  |  | **Age < 40 [n=38]** | | **Age 40 – 60 [n=12]** | | **Males [n=28]** | | **Females [n=22]** | | **Left Hand Dominance [n=1]** | | **Right Hand Dominance [n=49]** | |
|  |  |  | **Median (IQR; MAD)** | **p-value** | **Median (IQR; MAD)** | **p-value** | **Median (IQR; MAD)** | **p-value** | **Median (IQR; MAD)** | **p-value** | **Median (IQR; MAD)** | **p-value** | **Median (IQR; MAD)** | **p-value** |
| **1 Hz Sampled Data** | | | | | | | | | | | | | | |
| % time rSO_2_ > 30% | Frontal | Left | 100 (100 – 100; 0) | **0.0457** | 100 (100 – 100; 0) | 0.3593 | 100 (100 – 100; 0) | 0.5426 | 100 (100 – 100; 0) | **0.0203** | 100 (100 – 100; 0) | 1 | 100 (100 – 100; 0) | **0.0258** |
|  |  | Right | 100 (100 – 100; 0) |  | 100 (100 – 100; 0) |  | 100 (100 – 100; 0) |  | 100 (100 – 100; 0) |  | 100 (100 – 100; 0) |  | 100 (100 – 100; 0) |  |
|  | Parietal | Left | 100 (100 – 100; 0) | 1 | 100 (100 – 100; 0) | 0.5807 | 100 (100 – 100; 0) | 0.3218 | 100 (100 – 100; 0) | 0.6099 | 100 (100 – 100; 0) | 1 | 100 (100 – 100; 0) | 0.7025 |
|  |  | Right | 100 (100 – 100; 0) |  | 100 (100 – 100; 0) |  | 100 (100 – 100; 0) |  | 100 (100 – 100; 0) |  | 100 (100 – 100; 0) |  | 100 (100 – 100; 0) |  |
|  | Temporal | Left | 100 (100 – 100; 0) | 1 | 100 (100 – 100; 0) | 0.3593 | 100 (100 – 100; 0) | 0.1611 | 100 (100 – 100; 0) | 0.3398 | 100 (100 – 100; 0) | 1 | 100 (100 – 100; 0) | 0.584 |
|  |  | Right | 100 (100 – 100; 0) |  | 100 (100 – 100; 0) |  | 100 (100 – 100; 0) |  | 100 (100 – 100; 0) |  | 100 (100 – 100; 0) |  | 100 (100 – 100; 0) |  |
|  | Occipital | Left | 100 (100 – 100; 0) | 0.5689 | 100 (100 – 100; 0) | 1 | 100 (100 – 100; 0) | 0.3349 | 100 (100 – 100; 0) | 0.1621 | 100 (100 – 100; 0) | 1 | 100 (100 – 100; 0) | 0.5677 |
|  |  | Right | 100 (100 – 100; 0) |  | 100 (100 – 100; 0) |  | 100 (100 – 100; 0) |  | 100 (100 – 100; 0) |  | 100 (100 – 100; 0) |  | 100 (100 – 100; 0) |  |
| % time rSO_2_ > 40% | Frontal | Left | 100 (100 – 100; 0) | **<0.001** | 100 (99.96 – 100; 0) | **0.0031** | 100 (100 – 100; 0) | **<0.001** | 100 (100 – 100; 0) | **<0.001** | 100 (100 – 100; 0) | 1 | 100 (100 – 100; 0) | **<0.001** |
|  |  | Right | 74.07 (2.38 – 98.94; 25.93) |  | 18.74 (0.75 – 98.85; 18.74) |  | 79.35 (3.97 – 99.06; 20.65) |  | 23.65 (1.06 – 96.23; 23.65) |  | 46.4 (46.4 – 46.4; 0) |  | 71.23 (0.94 – 98.95; 28.77) |  |
|  | Parietal | Left | 100 (63.99 – 100; 0) | 0.6753 | 100 (82.14 – 100; 0) | 0.3529 | 100 (82.87 – 100; 0) | 0.7029 | 100 (74.5 – 100; 0) | 0.8557 | 100 (100 – 100; 0) | 1 | 100 (72.51 – 100; 0) | 0.9904 |
|  |  | Right | 100 (92.71 – 100; 0) |  | 90.97 (84.57 – 99.86; 8.94) |  | 100 (86.6 – 100; 0) |  | 100 (89.49 – 100; 0) |  | 79 (79 – 79; 0) |  | 100 (89.25 – 100; 0) |  |
|  | Temporal | Left | 58.53 (17.03 – 98.4; 40.88) | **<0.001** | 63.93 (0 – 88.53; 32.38) | **0.0182** | 37.6 (0 – 84.24; 37.6) | **<0.001** | 70.88 (37.52 – 98.4; 28.65) | 0.0829 | 30 (30 – 30; 0) | 1 | 58.84 (4.81 – 94.84; 40.54) | **<0.001** |
|  |  | Right | 100 (100 – 100; 0) |  | 100 (82.73 – 100; 0) |  | 100 (100 – 100; 0) |  | 100 (0.48 – 100; 0) |  | 100 (100 – 100; 0) |  | 100 (100 – 100; 0) |  |
|  | Occipital | Left | 0 (0 – 0; 0) | **<0.001** | 0 (0 – 0; 0) | **<0.001** | 0 (0 – 0; 0) | **<0.001** | 0 (0 – 0; 0) | **<0.001** | 0 (0 – 0; 0) | 1 | 0 (0 – 0; 0) | **<0.001** |
|  |  | Right | 100 (94.3 – 100; 0) |  | 98.66 (92.45 – 100; 1.34) |  | 100 (99.96 – 100; 0) |  | 95.93 (65.97 – 100; 4.07) |  | 100 (100 – 100; 0) |  | 100 (93.23 – 100; 0) |  |
| % time rSO_2_ > 50% | Frontal | Left | 0 (0 – 88.74; 0) | **0.0076** | 0 (0 – 4.98; 0) | 0.0786 | 0 (0 – 51.18; 0) | **0.0041** | 0 (0 – 87.99; 0) | 0.166 | 0 (0 – 0; 0) | 1 | 0 (0 – 79.5; 0) | **0.002** |
|  |  | Right | 0 (0 – 0; 0) |  | 0 (0 – 0; 0) |  | 0 (0 – 0; 0) |  | 0 (0 – 0; 0) |  | 0 (0 – 0; 0) |  | 0 (0 – 0; 0) |  |
|  | Parietal | Left | 51.83 (0.68 – 88.72; 42.52) | **<0.001** | 17.4 (8.25 – 45.4; 17.4) | 0.0575 | 59.15 (7.23 – 88.66; 37.15) | **<0.001** | 17.4 (2.26 – 57.95; 17.4) | 0.4144 | 92.6 (92.6 – 92.6; 0) | 1 | 34.08 (2.17 – 87.55; 34.08) | **<0.001** |
|  |  | Right | 0.07 (0 – 23.55; 0.07) |  | 5.66 (0 – 16.71; 5.66) |  | 0 (0 – 0.24; 0) |  | 12.03 (0.83 – 25.85; 12.03) |  | 0.2 (0.2 – 0.2; 0) |  | 0.13 (0 – 19.33; 0.13) |  |
|  | Temporal | Left | 0 (0 – 0; 0) | **<0.001** | 0 (0 – 0; 0) | 0.1058 | 0 (0 – 0; 0) | **<0.001** | 0 (0 – 0; 0) | **0.0336** | 0 (0 – 0; 0) | 1 | 0 (0 – 0; 0) | **<0.001** |
|  |  | Right | 16.59 (0 – 52.46; 16.59) |  | 0 (0 – 4.57; 0) |  | 16.59 (0 – 50.22; 16.59) |  | 0.09 (0 – 31.32; 0.09) |  | 9.8 (9.8 – 9.8; 0) |  | 1.16 (0 – 49.1; 1.16) |  |
|  | Occipital | Left | 0 (0 – 0; 0) | **<0.001** | 0 (0 – 0; 0) | **0.0165** | 0 (0 – 0; 0) | **<0.001** | 0 (0 – 0; 0) | **<0.001** | 0 (0 – 0; 0) | 1 | 0 (0 – 0; 0) | **<0.001** |
|  |  | Right | 2.81 (0 – 26.07; 2.81) |  | 0 (0 – 6.81; 0) |  | 0.54 (0 – 14.35; 0.54) |  | 4.41 (0 – 27.79; 4.41) |  | 0 (0 – 0; 0) |  | 1.93 (0 – 23.66; 1.93) |  |
| % time rSO_2_ > 60% | Frontal | Left | 0 (0 – 0; 0) | 0.3206 | 0 (0 – 0; 0) | 1 | 0 (0 – 0; 0) | 0.1611 | 0 (0 – 0; 0) | 1 | 0 (0 – 0; 0) | 1 | 0 (0 – 0; 0) | 0.3198 |
|  |  | Right | 0 (0 – 0; 0) |  | 0 (0 – 0; 0) |  | 0 (0 – 0; 0) |  | 0 (0 – 0; 0) |  | 0 (0 – 0; 0) |  | 0 (0 – 0; 0) |  |
|  | Parietal | Left | 0 (0 – 0; 0) | 0.2504 | 0 (0 – 0; 0) | 0.3593 | 0 (0 – 0; 0) | 0.4364 | 0 (0 – 0; 0) | 0.1621 | 0 (0 – 0; 0) | 1 | 0 (0 – 0; 0) | 0.1446 |
|  |  | Right | 0 (0 – 0; 0) |  | 0 (0 – 0; 0) |  | 0 (0 – 0; 0) |  | 0 (0 – 0; 0) |  | 0 (0 – 0; 0) |  | 0 (0 – 0; 0) |  |
|  | Temporal | Left | 0 (0 – 0; 0) | **0.0427** | 0 (0 – 0; 0) | 1 | 0 (0 – 0; 0) | 0.5708 | 0 (0 – 0; 0) | **0.0809** | 0 (0 – 0; 0) | 1 | 0 (0 – 0; 0) | 0.0905 |
|  |  | Right | 0 (0 – 0; 0) |  | 0 (0 – 0; 0) |  | 0 (0 – 0; 0) |  | 0 (0 – 0; 0) |  | 0 (0 – 0; 0) |  | 0 (0 – 0; 0) |  |
|  | Occipital | Left | 0 (0 – 0; 0) | **0.0223** | 0 (0 – 0; 0) | 1 | 0 (0 – 0; 0) | 0.1611 | 0 (0 – 0; 0) | **0.0809** | 0 (0 – 0; 0) | 1 | 0 (0 – 0; 0) | **0.023** |
|  |  | Right | 0 (0 – 0; 0) |  | 0 (0 – 0; 0) |  | 0 (0 – 0; 0) |  | 0 (0 – 0; 0) |  | 0 (0 – 0; 0) |  | 0 (0 – 0; 0) |  |
| % time COx-a > 0 | Frontal | Left | 48.56 (39.33 – 54.26; 8.33) | 0.2302 | 52.9 (47 – 55.02; 3.37) | 0.665 | 48.78 (39.96 – 53.68; 6.95) | 0.4559 | 52.21 (40.3 – 55.79; 4.74) | 0.6304 | 51.85 (51.85 – 51.85; 0) | 1 | 49.9 (39.92 – 54.38; 6.95) | 0.3096 |
|  |  | Right | 50.34 (42.86 – 56.02; 6.77) |  | 46.96 (45.28 – 52.92; 3.95) |  | 49.36 (44.89 – 54.77; 5.65) |  | 51.6 (42.82 – 57.53; 8.18) |  | 39.71 (39.71 – 39.71; 0) |  | 49.56 (44.59 – 56.2; 6.52) |  |
|  | Parietal | Left | 49.24 (43.54 – 57.18; 7.05) | 0.5998 | 51.32 (44.56 – 53.25; 3.11) | 0.5444 | 51.14 (40.15 – 55.1; 6.44) | 0.481 | 48.63 (44.39 – 53.59; 4.48) | 0.7424 | 51.23 (51.23 – 51.23; 0) | 1 | 49.63 (43.96 – 54.67; 5.67) | 0.5271 |
|  |  | Right | 48.29 (42.5 – 56.9; 8.29) |  | 45.43 (39.66 – 52.37; 5.96) |  | 46 (41.37 – 55.63; 5.71) |  | 48.83 (40.8 – 57.69; 8.9) |  | 37.45 (37.45 – 37.45; 0) |  | 46.94 (41.59 – 56.91; 8.22) |  |
|  | Temporal | Left | 49.17 (44.33 – 55.85; 6.05) | 0.7911 | 53.16 (49.04 – 58.27; 5.1) | 0.6236 | 49.31 (44.17 – 54.62; 5.41) | 0.5069 | 53.16 (47.21 – 57.8; 5.42) | 0.4597 | 50.21 (50.21 – 50.21; 0) | 1 | 49.72 (45.66 – 56.38; 6.15) | 0.8534 |
|  |  | Right | 48.45 (42.15 – 57.93; 8.28) |  | 51.35 (40.77 – 61.17; 10.42) |  | 52.01 (41.78 – 59.03; 9.56) |  | 48.45 (41.96 – 58.04; 8.18) |  | 75.72 (75.72 – 75.72; 0) |  | 48.5 (41.8 – 58.09; 8.61) |  |
|  | Occipital | Left | 50.2 (44.23 – 54.62; 5.93) | 0.5784 | 51.27 (42.45 – 55; 6.35) | 0.4705 | 49.85 (43.95 – 53.56; 5.11) | 0.4268 | 51.69 (46.36 – 58.35; 6.32) | 0.6812 | 28.19 (28.19 – 28.19; 0) | 1 | 50.68 (44.2 – 54.92; 5.75) | 0.3745 |
|  |  | Right | 49.35 (42.78 – 59.77; 7.31) |  | 50.85 (49.8 – 57.83; 3.66) |  | 48.77 (43.29 – 58.38; 7.07) |  | 51.47 (46.72 – 58.28; 6.2) |  | 32.92 (32.92 – 32.92; 0) |  | 51.02 (44.91 – 58.37; 7) |  |
| % time COx-a > 0.2 | Frontal | Left | 21.82 (16.01 – 31.45; 6.97) | 0.1029 | 25.05 (20.91 – 28.4; 3.72) | 0.8852 | 21.09 (15.8 – 26.5; 5.29) | 0.0768 | 27.6 (19.69 – 31.45; 6.06) | 0.6304 | 24.07 (24.07 – 24.07; 0) | 1 | 22.98 (16.06 – 29.49; 6.92) | 0.0751 |
|  |  | Right | 26.2 (21 – 35; 7.09) |  | 25.14 (21.52 – 27.45; 4.06) |  | 24.34 (20.54 – 30.72; 5.43) |  | 25.91 (22.02 – 34.42; 6.91) |  | 17.7 (17.7 – 17.7; 0) |  | 25.87 (21.49 – 34.14; 6.1) |  |
|  | Parietal | Left | 24.54 (16.42 – 28.55; 5.1) | 0.6144 | 24.06 (18.63 – 26.41; 3.51) | 0.7508 | 23.6 (15.92 – 28.43; 5.25) | 0.7995 | 24.75 (19.73 – 28.17; 3.64) | 0.5035 | 17.7 (17.7 – 17.7; 0) | 1 | 24.68 (17.95 – 28.38; 4.11) | 0.4774 |
|  |  | Right | 24.09 (19.09 – 32.85; 7.73) |  | 23.16 (17.1 – 29.34; 6.34) |  | 22.24 (15.83 – 31.01; 7.5) |  | 26.65 (20.3 – 31.77; 5.88) |  | 10.7 (10.7 – 10.7; 0) |  | 24.18 (19.08 – 31.84; 7.38) |  |
|  | Temporal | Left | 23.59 (17.97 – 30.75; 5.79) | 0.6071 | 22.51 (18.17 – 29.4; 5.43) | 0.5444 | 22.23 (17.85 – 28.8; 4.98) | 0.0996 | 25.91 (18.76 – 30.85; 6.83) | 0.2267 | 17.7 (17.7 – 17.7; 0) | 1 | 23.21 (18.04 – 30.62; 5.78) | 0.9773 |
|  |  | Right | 25.14 (16.5 – 32.45; 8.05) |  | 21.6 (15.99 – 29.67; 6.81) |  | 26.07 (20.71 – 32.68; 6.26) |  | 20.24 (14.86 – 28.7; 8.02) |  | 33.95 (33.95 – 33.95; 0) |  | 24.87 (16.48 – 32.1; 8.3) |  |
|  | Occipital | Left | 17.24 (13.79 – 22.3; 4.46) | **<0.001** | 16.65 (11.19 – 22.92; 5.88) | **0.0194** | 15.54 (12.5 – 20.83; 3.97) | **<0.001** | 18 (13.32 – 23.31; 5.11) | **0.0077** | 2.67 (2.67 – 2.67; 0) | 1 | 17.47 (12.94 – 22.49; 4.74) | **<0.001** |
|  |  | Right | 25.44 (19.43 – 32.48; 6.66) |  | 24.39 (21.21 – 29.65; 4.23) |  | 24.2 (19.49 – 30.83; 5.36) |  | 25.73 (21.37 – 32.41; 6.23) |  | 10.08 (10.08 – 10.08; 0) |  | 24.76 (20.08 – 32.87; 5.5) |  |
| % time COx-a > 0.3 | Frontal | Left | 12.39 (8 – 21.4; 5.31) | 0.128 | 14.89 (10.69 – 16.04; 1.2) | 0.4528 | 11.13 (8.09 – 16.5; 4.06) | 0.1587 | 15.5 (10.74 – 20.92; 5.37) | 0.3185 | 12.96 (12.96 – 12.96; 0) | 1 | 14.74 (8.18 – 20.77; 6.24) | 0.052 |
|  |  | Right | 16.46 (11.78 – 24.65; 7.2) |  | 15.05 (11.99 – 18.57; 3.42) |  | 15.12 (11.29 – 20; 4.66) |  | 16.46 (12.77 – 25.14; 6.5) |  | 5.76 (5.76 – 5.76; 0) |  | 16.14 (12.24 – 23.42; 4.99) |  |
|  | Parietal | Left | 14.94 (9.2 – 19.74; 5.26) | 0.6701 | 15.65 (11.43 – 17.74; 2.7) | 0.8399 | 15.23 (8 – 19.39; 5.81) | 0.7932 | 14.94 (12.82 – 17.93; 2.88) | 0.4596 | 5.56 (5.56 – 5.56; 0) | 1 | 15.03 (9.65 – 18.46; 4.19) | 0.9067 |
|  |  | Right | 15.35 (10.15 – 23.1; 7) |  | 11.63 (8.88 – 18.64; 5.81) |  | 11.8 (7.52 – 18.64; 5.44) |  | 16.33 (12.01 – 20.87; 4.81) |  | 6.17 (6.17 – 6.17; 0) |  | 15.21 (10.09 – 21.31; 5.42) |  |
|  | Temporal | Left | 13.41 (9.08 – 19.09; 5.12) | 0.5572 | 11.67 (8.86 – 17.51; 4.44) | 1 | 12.28 (8.89 – 17.47; 3.82) | 0.1176 | 16.41 (9.28 – 21.32; 7.06) | 0.3787 | 5.97 (5.97 – 5.97; 0) | 1 | 12.94 (9.22 – 18.94; 4.83) | 0.7817 |
|  |  | Right | 16.28 (10.03 – 20.94; 5.88) |  | 12.35 (8.25 – 18.4; 5.58) |  | 15.87 (11.04 – 20.63; 4.92) |  | 12.39 (7.05 – 19.57; 6.9) |  | 18.72 (18.72 – 18.72; 0) |  | 15.04 (8.7 – 20.47; 6.06) |  |
|  | Occipital | Left | 7.97 (4.8 – 12.08; 3.81) | **<0.001** | 8.33 (3.18 – 11.26; 4.33) | **0.002** | 7.98 (3.96 – 11.77; 4.05) | **<0.001** | 8.2 (4.62 – 12.16; 4.04) | **0.0012** | 0.41 (0.41 – 0.41; 0) | 1 | 8.1 (4.54 – 12.23; 4.11) | **<0.001** |
|  |  | Right | 15.89 (11.47 – 21.41; 5.03) |  | 16.56 (11.66 – 21.31; 4.81) |  | 14.8 (11.73 – 20.59; 4.52) |  | 16.67 (11.26 – 21.44; 5.3) |  | 5.97 (5.97 – 5.97; 0) |  | 16.23 (11.91 – 21.51; 5.04) |  |
| % time HbOx > 0 | Frontal | Left | 47.47 (40 – 56.22; 8.55) | 0.0594 | 48.88 (43.29 – 51.41; 3.81) | 0.8399 | 46.99 (39.92 – 56.73; 7.56) | 0.0754 | 48.88 (44.86 – 55.05; 5.98) | 0.3072 | 68.52 (68.52 – 68.52; 0) | 1 | 47.5 (40.56 – 56.14; 7.54) | **0.0276** |
|  |  | Right | 51.81 (45.69 – 60.69; 7.4) |  | 48.59 (46.38 – 53.65; 5.02) |  | 50.23 (46.27 – 59.46; 5.82) |  | 51.81 (45.08 – 58.22; 6.63) |  | 45.88 (45.88 – 45.88; 0) |  | 51.45 (45.62 – 58.8; 6.88) |  |
|  | Parietal | Left | 52 (46.39 – 59.13; 6.69) | 0.7831 | 46.01 (36.88 – 52.02; 7.31) | 0.141 | 51.2 (42.93 – 59.1; 7.91) | 0.7493 | 51.94 (45.22 – 54.45; 4.8) | 0.7336 | 49.79 (49.79 – 49.79; 0) | 1 | 51.81 (44.29 – 55.34; 6.98) | 0.6544 |
|  |  | Right | 51.69 (44.02 – 60.24; 8.14) |  | 51.98 (46.65 – 54.27; 3.65) |  | 52.22 (44.69 – 56.88; 7.02) |  | 51.54 (43.85 – 59.74; 8.14) |  | 52.26 (52.26 – 52.26; 0) |  | 51.79 (44.6 – 59.48; 7.68) |  |
|  | Temporal | Left | 51.35 (43.82 – 58.42; 7.43) | 0.3211 | 49.94 (43.01 – 57.15; 7.3) | 0.3408 | 50.5 (43.48 – 57.67; 7.09) | 0.4363 | 52.8 (42.39 – 59.02; 8.04) | 0.9345 | 51.65 (51.65 – 51.65; 0) | 1 | 51.06 (43.37 – 58.55; 7.69) | 0.8201 |
|  |  | Right | 55.08 (43.32 – 61.04; 7.64) |  | 44.1 (38.95 – 50.87; 7.29) |  | 54.78 (44.1 – 59.3; 7.08) |  | 47.63 (40.69 – 59.66; 10.2) |  | 70.58 (70.58 – 70.58; 0) |  | 50 (41.46 – 59.4; 8.76) |  |
|  | Occipital | Left | 53.28 (46.03 – 55.99; 5.88) | 0.6144 | 52.12 (43.74 – 57.84; 7.19) | 0.931 | 51.93 (45.83 – 57.36; 5.94) | 0.3896 | 53.28 (45.87 – 57.26; 6.17) | 0.8603 | 45.88 (45.88 – 45.88; 0) | 1 | 53.27 (45.82 – 57.67; 6.01) | 0.6596 |
|  |  | Right | 49 (42.27 – 58.16; 7.98) |  | 49.98 (46.71 – 55.62; 5.02) |  | 47.49 (43.96 – 56.24; 7.33) |  | 53.02 (43.67 – 57.9; 9.04) |  | 44.86 (44.86 – 44.86; 0) |  | 49.06 (43.36 – 57.83; 7.74) |  |
| % time HbOx > 0.2 | Frontal | Left | 22.62 (17.47 – 29.96; 6.4) | **0.0166** | 22.03 (20.39 – 25.76; 2.74) | **0.0464** | 21.78 (16.44 – 26.47; 5.47) | **0.0096** | 23.66 (20.73 – 28.49; 4.46) | 0.1556 | 32.92 (32.92 – 32.92; 0) | 1 | 22.28 (17.89 – 27.42; 4.95) | **0.0019** |
|  |  | Right | 28.13 (22.73 – 35.82; 6.17) |  | 25.7 (22.49 – 28.94; 3.21) |  | 26.83 (22.49 – 31.3; 4.34) |  | 28.49 (23.26 – 34.08; 5.92) |  | 19.55 (19.55 – 19.55; 0) |  | 27.89 (22.63 – 32.27; 5.26) |  |
|  | Parietal | Left | 28.56 (20.65 – 34.45; 6.37) | 0.6551 | 24.19 (18.16 – 28.45; 5.39) | 0.3123 | 26.5 (16.82 – 33.03; 7.66) | 0.583 | 28.49 (22.7 – 31.98; 5.62) | 0.6221 | 15.23 (15.23 – 15.23; 0) | 1 | 27.63 (20.39 – 32.93; 6.22) | 0.4708 |
|  |  | Right | 27.32 (22.04 – 36.81; 7.1) |  | 26.6 (23.7 – 31.96; 4.97) |  | 25.85 (21.73 – 33.84; 7.36) |  | 29.01 (22.68 – 35.26; 6.39) |  | 16.05 (16.05 – 16.05; 0) |  | 27.06 (22.44 – 35.5; 5.53) |  |
|  | Temporal | Left | 25.16 (20.11 – 30.28; 5.26) | 0.2893 | 18.59 (16.28 – 24.53; 4.39) | 0.931 | 23.42 (18.77 – 28.58; 5.06) | 0.2253 | 24.14 (18.14 – 30.48; 6.3) | 0.9345 | 22.02 (22.02 – 22.02; 0) | 1 | 23.6 (18.25 – 30.1; 5.88) | 0.4664 |
|  |  | Right | 28.6 (21.32 – 36.12; 7.7) |  | 23.88 (14.51 – 26.88; 6.26) |  | 27.66 (22.47 – 32.4; 5.61) |  | 25.62 (15.32 – 33.05; 9.94) |  | 39.3 (39.3 – 39.3; 0) |  | 26.32 (18.64 – 31.8; 7.45) |  |
|  | Occipital | Left | 21.97 (17.13 – 28.65; 6.45) | 0.1444 | 16.55 (13.12 – 26.33; 6.48) | 0.0885 | 23.95 (16.95 – 29.71; 6.51) | 0.5069 | 18.23 (12.92 – 25.26; 6.41) | **0.0161** | 6.79 (6.79 – 6.79; 0) | 1 | 21.8 (15.45 – 28.68; 6.81) | **0.0325** |
|  |  | Right | 25.76 (19.12 – 30.91; 6.53) |  | 25.5 (21.74 – 31.79; 5.1) |  | 25.17 (20.24 – 29.34; 4.47) |  | 26.46 (19.8 – 32.41; 6.37) |  | 13.79 (13.79 – 13.79; 0) |  | 25.61 (20.65 – 31.47; 5.86) |  |
| % time HbOx > 0.3 | Frontal | Left | 14.49 (8.1 – 18.97; 6) | **0.0248** | 13.28 (11.39 – 17.02; 3.03) | **0.0194** | 11.46 (6.67 – 17.22; 5.59) | **0.0113** | 15.63 (12.69 – 17.13; 2.52) | 0.1392 | 17.28 (17.28 – 17.28; 0) | 1 | 13.55 (8.57 – 17.18; 3.83) | **0.0027** |
|  |  | Right | 16.46 (12.68 – 24.46; 5.98) |  | 17.1 (15.19 – 18.93; 2.05) |  | 15.39 (13.24 – 23.08; 4.43) |  | 17.1 (13.4 – 23.8; 4.35) |  | 8.44 (8.44 – 8.44; 0) |  | 17.01 (13.35 – 23.23; 4.14) |  |
|  | Parietal | Left | 17.74 (11.69 – 23.93; 6.23) | 0.6071 | 15.84 (10.43 – 19.6; 4.91) | 0.4357 | 16.77 (9.71 – 19.85; 6.51) | 0.5607 | 18.86 (14.65 – 22.96; 4.25) | 0.8235 | 5.56 (5.56 – 5.56; 0) | 1 | 17.14 (11.42 – 22.7; 5.72) | 0.473 |
|  |  | Right | 17.8 (13.6 – 26.96; 6.27) |  | 17.44 (15.71 – 20.51; 2.21) |  | 17.53 (12.49 – 22.07; 4.94) |  | 18.73 (15.25 – 22.73; 3.92) |  | 4.12 (4.12 – 4.12; 0) |  | 18 (14.71 – 23.08; 4.35) |  |
|  | Temporal | Left | 15.72 (12.42 – 20.86; 3.82) | 0.6035 | 10.41 (7.55 – 14; 3.03) | 0.8852 | 14.51 (9.28 – 18.25; 4.61) | 0.3419 | 15.01 (10.81 – 18.67; 4.26) | 0.6472 | 9.26 (9.26 – 9.26; 0) | 1 | 14.82 (10.28 – 18.46; 4.3) | 0.8064 |
|  |  | Right | 18.03 (10.58 – 22.77; 6.79) |  | 13.02 (6.92 – 16.31; 4.59) |  | 18.38 (11.79 – 22.37; 4.81) |  | 13.91 (9.8 – 17.27; 4.04) |  | 24.9 (24.9 – 24.9; 0) |  | 15.14 (9.73 – 21.28; 5.78) |  |
|  | Occipital | Left | 11.1 (7.71 – 19.4; 5.06) | **0.0402** | 11.16 (4.14 – 13.1; 3.84) | **0.0141** | 12.47 (8.04 – 19.73; 4.84) | 0.1101 | 9.8 (5.8 – 15.59; 5.32) | **0.0116** | 0.82 (0.82 – 0.82; 0) | 1 | 11.22 (7.58 – 17.46; 4.85) | **0.0037** |
|  |  | Right | 16.53 (11.02 – 21.67; 5.59) |  | 18.64 (12.29 – 19.63; 2.04) |  | 17.64 (12.26 – 19.79; 3.9) |  | 16.5 (10.97 – 20.91; 5.22) |  | 7.2 (7.2 – 7.2; 0) |  | 17.38 (11.88 – 20.71; 4.95) |  |
| % time HHbx > 0 | Frontal | Left | 50.69 (43.18 – 59.75; 7.99) | 0.9255 | 48.86 (45.3 – 51.5; 3.46) | 0.8852 | 49.35 (42.79 – 61.17; 8.99) | 0.451 | 50.21 (45.09 – 54.26; 5.12) | 0.6472 | 65.23 (65.23 – 65.23; 0) | 1 | 49.59 (44.32 – 57.91; 6.79) | 0.8229 |
|  |  | Right | 51.62 (42.99 – 57.68; 8.06) |  | 51.93 (43.54 – 55.3; 7.92) |  | 52.07 (42.91 – 58.87; 8.31) |  | 48.29 (43.22 – 55.35; 6.17) |  | 58.64 (58.64 – 58.64; 0) |  | 51.52 (42.94 – 56.3; 8.03) |  |
|  | Parietal | Left | 50.41 (43.98 – 57.62; 7.06) | 0.7632 | 48.39 (46.2 – 49.65; 1.39) | 0.0531 | 50.41 (41.5 – 58.23; 8.78) | 0.422 | 49.34 (47.7 – 51.42; 2.07) | 0.2855 | 50.41 (50.41 – 50.41; 0) | 1 | 49.61 (43.88 – 56.28; 5.83) | 0.2257 |
|  |  | Right | 50.67 (45.99 – 57.52; 6.02) |  | 57.14 (50.88 – 61.46; 4.9) |  | 51.64 (45.43 – 57.87; 6.44) |  | 51.06 (47.32 – 58.76; 5.77) |  | 65.43 (65.43 – 65.43; 0) |  | 51.33 (46.61 – 58.11; 6.3) |  |
|  | Temporal | Left | 51.01 (43.67 – 57.38; 7.1) | 0.4832 | 42.26 (40.15 – 46.68; 2.83) | 0.4528 | 51.19 (44.41 – 56.94; 6.43) | 0.7307 | 44.66 (40.74 – 52.89; 4.6) | 0.2649 | 50.82 (50.82 – 50.82; 0) | 1 | 48.76 (41.86 – 56.26; 7.01) | 0.2723 |
|  |  | Right | 51.89 (46.43 – 58.56; 5.97) |  | 47.6 (40.68 – 52.49; 7.18) |  | 51.42 (46.24 – 56.71; 5.36) |  | 49.77 (41.84 – 59.88; 9.41) |  | 52.47 (52.47 – 52.47; 0) |  | 50.58 (44.8 – 58.13; 6.77) |  |
|  | Occipital | Left | 53.32 (47.3 – 58.83; 5.95) | 0.3471 | 48.65 (40.6 – 58.16; 9.39) | 0.6236 | 55.55 (48.38 – 61.35; 7.3) | 0.1764 | 50.83 (43.79 – 57.32; 7.34) | 0.9159 | 79.22 (79.22 – 79.22; 0) | 1 | 52.57 (45.75 – 58.23; 6.41) | 0.2897 |
|  |  | Right | 52.22 (46.58 – 56.61; 5.61) |  | 48.86 (43.74 – 52.46; 3.79) |  | 52.09 (45.8 – 57.19; 5.68) |  | 48.86 (45.96 – 54.23; 4.35) |  | 58.02 (58.02 – 58.02; 0) |  | 51.89 (45.76 – 55.02; 5.11) |  |
| % time HHbx > 0.2 | Frontal | Left | 25.86 (20.48 – 32.2; 5.93) | 0.5783 | 24.06 (21.53 – 26.47; 2.98) | 0.1123 | 23.62 (19.97 – 31.25; 5.38) | 0.2905 | 26.63 (22.74 – 31.91; 5.02) | 0.6641 | 29.42 (29.42 – 29.42; 0) | 1 | 24.14 (20.44 – 32.09; 5.57) | 0.2833 |
|  |  | Right | 25.51 (22.19 – 33.8; 4.88) |  | 27.42 (25.35 – 30.48; 2.34) |  | 25.78 (22.44 – 31.11; 3.87) |  | 27.42 (22.71 – 33.46; 5.66) |  | 26.75 (26.75 – 26.75; 0) |  | 27.02 (22.46 – 33.46; 5.15) |  |
|  | Parietal | Left | 25.5 (18.86 – 33.29; 7.12) | 0.4962 | 23.39 (20.3 – 26.43; 3.24) | **0.0102** | 24.82 (16.68 – 27.76; 6.43) | 0.2041 | 25.55 (21.5 – 31.58; 4.67) | 0.1771 | 24.69 (24.69 – 24.69; 0) | 1 | 25.13 (18.74 – 29.25; 5.89) | 0.1147 |
|  |  | Right | 25.86 (21.22 – 32.43; 5.36) |  | 30.69 (26.57 – 36.88; 5.74) |  | 25.86 (20.74 – 31; 5.13) |  | 30.21 (24.4 – 36.73; 6.3) |  | 32.72 (32.72 – 32.72; 0) |  | 26.32 (21.36 – 32.91; 5.55) |  |
|  | Temporal | Left | 26.38 (19.29 – 31.22; 5.91) | 0.7831 | 19.69 (17.34 – 25.63; 4.31) | 0.4025 | 27.48 (20.72 – 30.54; 6.46) | 0.768 | 20.82 (17.73 – 27.08; 5.31) | 0.3418 | 20.99 (20.99 – 20.99; 0) | 1 | 24.7 (18.15 – 30.5; 6.46) | 0.4345 |
|  |  | Right | 27.89 (19.02 – 32.91; 5.78) |  | 21.68 (19.01 – 29.05; 4.29) |  | 29.05 (18.46 – 32.79; 5.46) |  | 25.51 (19.79 – 29.15; 5.43) |  | 16.26 (16.26 – 16.26; 0) |  | 26.67 (19.5 – 32.72; 6.63) |  |
|  | Occipital | Left | 21.3 (16.9 – 28.25; 5.45) | **0.0127** | 15.77 (13.11 – 25.35; 5.03) | 0.3123 | 22.23 (16.39 – 29.04; 6.81) | 0.1215 | 20.2 (13.53 – 24.11; 5.81) | **0.0195** | 34.77 (34.77 – 34.77; 0) | 1 | 20.79 (14.84 – 25.93; 5.94) | **0.0055** |
|  |  | Right | 26.78 (21.42 – 32.77; 5.99) |  | 24.52 (17 – 26.53; 6.38) |  | 26.44 (19.51 – 32.05; 6.33) |  | 24.58 (20.48 – 32.35; 4.36) |  | 26.75 (26.75 – 26.75; 0) |  | 25.18 (20.08 – 32.76; 6.56) |  |
| % time HHbx > 0.3 | Frontal | Left | 15.33 (11.64 – 21.9; 4.89) | 0.6327 | 13.73 (12.16 – 17.71; 3.34) | **0.0351** | 13.9 (12.16 – 18.22; 2.88) | 0.1381 | 17.65 (11.66 – 24.66; 6.56) | 0.6556 | 13.99 (13.99 – 13.99; 0) | 1 | 15.1 (11.33 – 21.65; 4.63) | 0.1462 |
|  |  | Right | 16.15 (12.92 – 23.18; 4.64) |  | 18.93 (17.83 – 21.32; 1.55) |  | 16.8 (13.57 – 21.29; 3.94) |  | 18.89 (14.31 – 24.75; 5.74) |  | 8.85 (8.85 – 8.85; 0) |  | 18.15 (13.65 – 21.96; 4.5) |  |
|  | Parietal | Left | 16.36 (10.52 – 22; 5.78) | 0.5297 | 15.03 (12.02 – 17.58; 3.02) | **0.0262** | 13.69 (9.12 – 18.69; 5.09) | 0.1983 | 17.35 (12.98 – 21.92; 4.54) | 0.3662 | 11.32 (11.32 – 11.32; 0) | 1 | 16.4 (10.45 – 20.41; 5.6) | 0.1637 |
|  |  | Right | 16.52 (13.38 – 21.59; 4.36) |  | 21.75 (15.8 – 23.57; 3.14) |  | 16.05 (12.66 – 21.57; 4.3) |  | 21.04 (15.18 – 26.02; 5.85) |  | 15.64 (15.64 – 15.64; 0) |  | 16.92 (13.32 – 23.25; 4.77) |  |
|  | Temporal | Left | 16.44 (9.99 – 19.92; 5.27) | 0.6071 | 12 (8.44 – 15.83; 4.28) | 0.2726 | 18.1 (12.16 – 19.97; 3.83) | 0.4363 | 12.33 (8.99 – 17.91; 4.23) | 0.511 | 8.23 (8.23 – 8.23; 0) | 1 | 14.87 (9.83 – 19.68; 5.04) | 0.3113 |
|  |  | Right | 17.71 (9.02 – 24.11; 6.88) |  | 15.37 (10.01 – 20.6; 5.52) |  | 18.32 (10.09 – 24.07; 6.15) |  | 15.37 (8.9 – 21.16; 6.45) |  | 8.02 (8.02 – 8.02; 0) |  | 17.09 (9.18 – 24.06; 7.03) |  |
|  | Occipital | Left | 11.12 (6.88 – 15.08; 4.22) | **0.003** | 7.36 (3.21 – 9.95; 3.69) | **0.0141** | 11.88 (6.61 – 16.39; 4.63) | **0.0483** | 8.25 (4.79 – 10.63; 3.44) | **<0.001** | 16.46 (16.46 – 16.46; 0) | 1 | 9.88 (5.01 – 14.91; 5.03) | **<0.001** |
|  |  | Right | 16.12 (11.71 – 22.27; 5.54) |  | 13.09 (9.71 – 21.86; 4.15) |  | 16.12 (9.85 – 22.52; 6.35) |  | 14.59 (12.26 – 21.03; 4.66) |  | 9.88 (9.88 – 9.88; 0) |  | 15.67 (10.99 – 22.11; 5.9) |  |
| % time tHbx > 0 | Frontal | Left | 48.8 (42.32 – 59.47; 7.9) | 0.3211 | 49.26 (46.47 – 50.72; 2.5) | 0.8852 | 47.01 (41.32 – 57.84; 9.01) | 0.1215 | 50.33 (46.25 – 54.58; 4.28) | 0.7336 | 67.7 (67.7 – 67.7; 0) | 1 | 49.11 (43.57 – 54.91; 5.8) | 0.2646 |
|  |  | Right | 52.26 (45.65 – 60.47; 7.58) |  | 47.86 (43.82 – 56.16; 6.23) |  | 52.26 (46.6 – 59.44; 6.44) |  | 49.71 (41.7 – 58.76; 8.37) |  | 48.97 (48.97 – 48.97; 0) |  | 50.77 (44.18 – 59.19; 8.01) |  |
|  | Parietal | Left | 50.78 (44.93 – 58.67; 6.69) | 0.9917 | 45.84 (36.82 – 48.47; 5.6) | 0.0051 | 50.78 (44.52 – 61.31; 8.97) | 0.7369 | 48.41 (43.77 – 52; 3.95) | 0.1028 | 46.71 (46.71 – 46.71; 0) | 1 | 50 (43.44 – 53.89; 5.4) | 0.2631 |
|  |  | Right | 50.94 (44.73 – 61.17; 7.55) |  | 56.31 (49.18 – 58.2; 6.93) |  | 50.94 (46.23 – 59.43; 6.69) |  | 53.52 (45.26 – 60.76; 7.76) |  | 61.52 (61.52 – 61.52; 0) |  | 51.44 (45.66 – 59.41; 6.85) |  |
|  | Temporal | Left | 51.7 (43.78 – 60.39; 8.64) | 0.6107 | 46.86 (43.98 – 49.84; 3.08) | 0.4025 | 50.44 (45.44 – 59.4; 7.06) | 0.623 | 48.91 (42.23 – 58.23; 8.28) | 0.9159 | 54.12 (54.12 – 54.12; 0) | 1 | 50 (43.6 – 58.9; 8.23) | 0.9349 |
|  |  | Right | 55.09 (48.61 – 58.69; 5.35) |  | 44.02 (41.59 – 49.62; 4.71) |  | 55.09 (47.77 – 57.5; 4.55) |  | 48.72 (43.92 – 55.68; 5.48) |  | 66.05 (66.05 – 66.05; 0) |  | 51.05 (44.24 – 57.22; 6.28) |  |
|  | Occipital | Left | 51.04 (47.17 – 59.23; 5.71) | 0.9959 | 52.37 (48.04 – 56.88; 4.55) | 0.7075 | 55.06 (47.49 – 61.27; 6.68) | 0.4461 | 50.11 (47.23 – 56.16; 4.59) | 0.8235 | 61.32 (61.32 – 61.32; 0) | 1 | 50.83 (47.37 – 56.97; 5.46) | 0.8646 |
|  |  | Right | 50.88 (45.09 – 61.01; 6.85) |  | 50.73 (47.47 – 55.09; 4.67) |  | 51.08 (44.28 – 57.88; 7.44) |  | 50.03 (46.21 – 59.92; 5.79) |  | 53.5 (53.5 – 53.5; 0) |  | 50.75 (44.93 – 60.36; 6.31) |  |
| % time tHbx > 0.2 | Frontal | Left | 24.62 (17.71 – 33; 7.52) | 0.3914 | 23.85 (18.82 – 24.85; 4.09) | 0.0464 | 22.19 (17.43 – 29.98; 5.5) | 0.0702 | 25.18 (20.48 – 32.96; 6.79) | 0.8603 | 38.68 (38.68 – 38.68; 0) | 1 | 24.02 (18.29 – 31.57; 6.72) | 0.0817 |
|  |  | Right | 26.77 (19.99 – 35.78; 7.56) |  | 27.15 (23 – 30.57; 4.11) |  | 29.28 (21.46 – 32.8; 6.97) |  | 25.85 (19.71 – 35.32; 6.63) |  | 14.61 (14.61 – 14.61; 0) |  | 27.09 (21.24 – 34.86; 6.79) |  |
|  | Parietal | Left | 27.47 (21.01 – 34.29; 6.82) | 0.8639 | 23.37 (17.35 – 25.84; 5.63) | **0.0226** | 25.96 (18.53 – 34.46; 8.56) | 0.9022 | 25.14 (21.01 – 31.32; 6.06) | 0.2267 | 13.17 (13.17 – 13.17; 0) | 1 | 25.43 (20.46 – 33.76; 7.71) | 0.4862 |
|  |  | Right | 28 (20.53 – 32.7; 6.24) |  | 30.98 (28.53 – 33.95; 3.09) |  | 28.09 (20.85 – 32.65; 5.44) |  | 29.69 (25.05 – 34.19; 4.66) |  | 28.19 (28.19 – 28.19; 0) |  | 28.95 (23.05 – 33.82; 5.37) |  |
|  | Temporal | Left | 27.27 (21.31 – 35.48; 6.31) | 0.8395 | 20.64 (16.1 – 26.24; 5.38) | 0.931 | 25.15 (20.86 – 29.88; 4.55) | 0.6172 | 25.42 (20.05 – 32.48; 6.13) | 0.8419 | 22.22 (22.22 – 22.22; 0) | 1 | 25.72 (20.18 – 32.27; 5.88) | 0.9151 |
|  |  | Right | 28.02 (23.33 – 34.63; 6.54) |  | 23.21 (15.44 – 26.95; 7.12) |  | 26.99 (21.69 – 31.58; 4.99) |  | 25.51 (18.81 – 31.88; 6.78) |  | 28.19 (28.19 – 28.19; 0) |  | 26.12 (19.48 – 31.91; 6.03) |  |
|  | Occipital | Left | 24.67 (20.95 – 29.93; 4.69) | 0.4832 | 19.11 (17.3 – 22.53; 3.14) | 0.0999 | 25.74 (20.49 – 32.16; 6.04) | 0.7743 | 22.29 (17.13 – 27.8; 6.09) | 0.0689 | 27.78 (27.78 – 27.78; 0) | 1 | 22.67 (18.73 – 29.2; 5.27) | 0.0868 |
|  |  | Right | 26.23 (20.08 – 30.96; 5.78) |  | 27.18 (22.51 – 32.25; 5.23) |  | 25.67 (18.79 – 31.55; 6.9) |  | 26.88 (21.69 – 31.42; 5.24) |  | 17.49 (17.49 – 17.49; 0) |  | 26.85 (21.6 – 32.07; 5.25) |  |
| % time tHbx > 0.3 | Frontal | Left | 15.77 (8.84 – 23.18; 7.63) | 0.2731 | 12.98 (9.23 – 15.88; 3.58) | **0.0029** | 12.96 (7.78 – 18.37; 5.23) | **0.0325** | 16.28 (12.1 – 23.98; 7.65) | 0.6304 | 19.55 (19.55 – 19.55; 0) | 1 | 14 (8.67 – 20.37; 5.78) | **0.0325** |
|  |  | Right | 16.14 (11.81 – 24.53; 6.81) |  | 18.81 (15.91 – 22.55; 3.75) |  | 16.14 (13.83 – 23.39; 6.42) |  | 17.84 (12.76 – 23.18; 5.55) |  | 7.61 (7.61 – 7.61; 0) |  | 17.15 (13.89 – 23.31; 5.4) |  |
|  | Parietal | Left | 18.41 (12.17 – 24.86; 6.52) | 0.8762 | 14.76 (11.47 – 18.27; 3.54) | **0.0464** | 18.07 (9.85 – 21; 6.42) | 0.8957 | 16.62 (12.3 – 23.77; 5.62) | 0.2962 | 2.47 (2.47 – 2.47; 0) | 1 | 18.05 (12.1 – 23.58; 5.78) | 0.5341 |
|  |  | Right | 17.48 (11.99 – 24.64; 7.04) |  | 20.31 (15.7 – 22.7; 4.48) |  | 16.63 (10.47 – 21.02; 5.73) |  | 20.27 (14.13 – 24.8; 4.77) |  | 8.44 (8.44 – 8.44; 0) |  | 18.56 (13.45 – 24.66; 6.09) |  |
|  | Temporal | Left | 17.2 (12.81 – 22.83; 4.66) | 0.9214 | 10.48 (7.55 – 16.26; 4.27) | 0.4705 | 15.9 (10.55 – 20.83; 5.1) | 0.4559 | 16.2 (12.81 – 20.83; 3.9) | 0.8787 | 7.82 (7.82 – 7.82; 0) | 1 | 15.96 (12.22 – 21.05; 4.22) | 0.7654 |
|  |  | Right | 19.06 (12.03 – 24.51; 6.01) |  | 12.82 (7.79 – 18.72; 5.73) |  | 18.55 (10.15 – 20.51; 5.71) |  | 17.44 (8.81 – 23.36; 7.2) |  | 15.23 (15.23 – 15.23; 0) |  | 18.54 (10.08 – 21.26; 6.55) |  |
|  | Occipital | Left | 15.34 (11.46 – 19.62; 4.22) | 0.2731 | 11.5 (8.72 – 14.44; 2.85) | 0.0885 | 15.34 (11.98 – 19.73; 4.22) | 0.3987 | 12.62 (8.62 – 16.19; 3.95) | 0.0689 | 15.23 (15.23 – 15.23; 0) | 1 | 14.26 (10.42 – 19.61; 5.25) | 0.0551 |
|  |  | Right | 17.32 (12.94 – 21.87; 4.59) |  | 17 (11.52 – 19.61; 4.69) |  | 18.05 (12.95 – 21.38; 4.76) |  | 16.37 (12.11 – 20.77; 4.47) |  | 6.79 (6.79 – 6.79; 0) |  | 17.34 (12.93 – 21.14; 4.41) |  |
| % time HbDiffx > 0 | Frontal | Left | 49.12 (39.73 – 55.1; 7.97) | 0.0996 | 50.79 (45.37 – 56.52; 5.87) | 0.8399 | 48.3 (39.29 – 54.99; 7.82) | 0.2905 | 50.79 (43.33 – 56.58; 6.65) | 0.3787 | 54.94 (54.94 – 54.94; 0) | 1 | 50.27 (41.26 – 56.37; 6.88) | 0.1164 |
|  |  | Right | 52.14 (46.7 – 59.01; 5.56) |  | 49.57 (41.72 – 54.82; 8.21) |  | 51.29 (46.13 – 55.68; 4.72) |  | 52.14 (43.96 – 60.88; 9.2) |  | 43.62 (43.62 – 43.62; 0) |  | 51.94 (46.55 – 59.48; 7.05) |  |
|  | Parietal | Left | 50.19 (44.31 – 56.6; 6.53) | 0.4513 | 50.77 (44.48 – 52.4; 2.99) | 0.931 | 50.65 (42.3 – 56.45; 7.31) | 0.3631 | 50.42 (46.62 – 53.94; 3.81) | 0.8787 | 50 (50 – 50; 0) | 1 | 50.46 (43.62 – 54.58; 5.36) | 0.5819 |
|  |  | Right | 48.53 (41.78 – 57.29; 8.23) |  | 47.13 (40.12 – 53.77; 7.01) |  | 47.28 (41.26 – 52.48; 5.9) |  | 53.01 (41.2 – 58.37; 8.36) |  | 37.45 (37.45 – 37.45; 0) |  | 48.88 (41.54 – 56.77; 7.89) |  |
|  | Temporal | Left | 50.84 (44.54 – 57.98; 7.09) | 0.9462 | 51.07 (49.19 – 58.97; 6.72) | 0.4357 | 50.65 (43.7 – 57.38; 6.99) | 0.7123 | 51.11 (48.3 – 58.62; 6.77) | 0.3662 | 52.47 (52.47 – 52.47; 0) | 1 | 50.49 (44.91 – 58.44; 7.23) | 0.5941 |
|  |  | Right | 52.57 (43.35 – 57.5; 5.89) |  | 47.39 (42.05 – 61.33; 10.53) |  | 53.49 (42.75 – 57.89; 9.81) |  | 49.21 (43.17 – 57.6; 7.28) |  | 78.4 (78.4 – 78.4; 0) |  | 49.7 (42.83 – 57.7; 7.92) |  |
|  | Occipital | Left | 49.66 (46.2 – 57.45; 5.04) | 0.8639 | 50.4 (45.18 – 55.42; 5.4) | 0.5444 | 48.82 (45.34 – 56.75; 4.84) | 0.7995 | 51.42 (46.38 – 57.45; 5.65) | 0.5035 | 31.89 (31.89 – 31.89; 0) | 1 | 50 (46.14 – 57.51; 5.19) | 0.8036 |
|  |  | Right | 48.78 (42.59 – 60.06; 7.8) |  | 51.54 (48.64 – 60.31; 5.92) |  | 47.34 (42.08 – 57.18; 8.13) |  | 53.13 (47.62 – 60.94; 7.36) |  | 36.01 (36.01 – 36.01; 0) |  | 51.01 (45.32 – 60.56; 7.01) |  |
| % time HbDiffx > 0.2 | Frontal | Left | 21.52 (16.76 – 31.89; 6.94) | **0.0467** | 22.62 (20.8 – 26.4; 2.92) | 0.5834 | 20.62 (16.53 – 27.46; 5.06) | **0.0213** | 25.3 (20.31 – 32.39; 6.5) | 0.3418 | 23.87 (23.87 – 23.87; 0) | 1 | 21.1 (16.95 – 30.96; 5.87) | **0.017** |
|  |  | Right | 28.34 (23.05 – 34.37; 5.83) |  | 23.4 (20.82 – 29.57; 3.9) |  | 27.8 (21.18 – 30.87; 6.26) |  | 27.33 (23.45 – 34.18; 5.01) |  | 18.52 (18.52 – 18.52; 0) |  | 27.43 (21.6 – 32.94; 5.83) |  |
|  | Parietal | Left | 24.64 (18.05 – 29.63; 6.34) | 0.6815 | 24.16 (19.77 – 25.4; 2.63) | 0.5444 | 24.04 (15.78 – 27.93; 5.95) | 0.8122 | 24.76 (22.26 – 28.78; 3.85) | 0.5652 | 18.31 (18.31 – 18.31; 0) | 1 | 24.48 (17.97 – 28.4; 5.38) | 0.4952 |
|  |  | Right | 23.88 (18.19 – 32.65; 7.7) |  | 23.92 (17.2 – 30.48; 6.57) |  | 23.7 (16.01 – 28.92; 7.7) |  | 27.87 (20.19 – 31.63; 7.52) |  | 8.44 (8.44 – 8.44; 0) |  | 24 (17.61 – 31.99; 6.62) |  |
|  | Temporal | Left | 23.51 (18.49 – 29.03; 5.51) | 0.5399 | 22.92 (18.43 – 24.86; 3.99) | 0.7508 | 22.09 (18.65 – 27.99; 4.94) | 0.1493 | 25.94 (18.32 – 30.35; 7.57) | 0.4455 | 18.72 (18.72 – 18.72; 0) | 1 | 23.11 (18.42 – 29.02; 4.89) | 0.7954 |
|  |  | Right | 25.51 (17.22 – 31.75; 7.32) |  | 19.43 (16.74 – 29.17; 5.5) |  | 26.45 (19.83 – 31.98; 6.32) |  | 21.94 (16.06 – 30.03; 6.77) |  | 39.92 (39.92 – 39.92; 0) |  | 24.95 (16.85 – 31.65; 7.62) |  |
|  | Occipital | Left | 20.16 (15.07 – 25.69; 5.38) | **0.0208** | 16.72 (11.08 – 26.33; 7.43) | **0.0404** | 22.11 (14.53 – 25.81; 5.26) | 0.0584 | 18.72 (13.67 – 22.09; 4.72) | **0.0317** | 3.91 (3.91 – 3.91; 0) | 1 | 19.76 (14.66 – 25.78; 6.02) | **0.0021** |
|  |  | Right | 25.41 (18.17 – 34.95; 7.34) |  | 24.31 (18.58 – 32.13; 6.56) |  | 24.28 (18.12 – 32.84; 6.31) |  | 27.43 (19.1 – 34.25; 8.08) |  | 9.67 (9.67 – 9.67; 0) |  | 26.25 (18.2 – 35.31; 8.09) |  |
| % time HbDiffx > 0.3 | Frontal | Left | 12.95 (8.83 – 21.18; 5.72) | 0.0683 | 15.44 (9.45 – 16.84; 2.44) | 0.4705 | 11.23 (8.88 – 17.73; 4) | **0.0382** | 16.47 (11.1 – 20.87; 5.29) | 0.4181 | 12.76 (12.76 – 12.76; 0) | 1 | 15.36 (8.99 – 20.56; 5.98) | **0.0248** |
|  |  | Right | 19.25 (14.51 – 23.14; 4.5) |  | 15.27 (12.57 – 17.75; 2.69) |  | 17.01 (13.45 – 20.22; 3.56) |  | 18.63 (14.48 – 22.7; 4.19) |  | 6.79 (6.79 – 6.79; 0) |  | 18.21 (14.06 – 22.36; 4.15) |  |
|  | Parietal | Left | 15.81 (11.08 – 18.79; 4.2) | 0.9131 | 16.48 (11.47 – 17.77; 1.43) | 0.6236 | 15.91 (8.44 – 19.04; 5.6) | 0.8122 | 16.18 (13.98 – 18.39; 2.31) | 0.9345 | 6.17 (6.17 – 6.17; 0) | 1 | 16.32 (11.38 – 18.5; 3.92) | 0.8758 |
|  |  | Right | 14.86 (9.83 – 22.16; 5.25) |  | 12.05 (9.13 – 17.57; 4.71) |  | 11.45 (7.53 – 19.76; 4.86) |  | 16.38 (12.96 – 21.69; 3.76) |  | 3.5 (3.5 – 3.5; 0) |  | 14.71 (9.63 – 20.12; 5.09) |  |
|  | Temporal | Left | 14.79 (8.68 – 19.88; 5.24) | 0.5748 | 12.53 (9.52 – 14.67; 3.28) | 0.8852 | 12.54 (9.65 – 16.94; 4.33) | 0.0868 | 17.29 (8.67 – 22.3; 7.71) | 0.3128 | 8.02 (8.02 – 8.02; 0) | 1 | 13.67 (10.11 – 18.92; 5.25) | 0.7681 |
|  |  | Right | 16.23 (10.37 – 21.5; 5.75) |  | 12.18 (7.32 – 17.96; 5.4) |  | 15.92 (11.19 – 21.92; 5.36) |  | 13.37 (7.17 – 19.18; 6.05) |  | 22.84 (22.84 – 22.84; 0) |  | 15.62 (8.4 – 19.48; 6.08) |  |
|  | Occipital | Left | 9.4 (6.43 – 14.71; 4.32) | **0.0023** | 8.96 (4.5 – 13.26; 4.44) | **0.0086** | 10.38 (6.94 – 14.89; 4.55) | **0.0038** | 7.97 (5.26 – 12.13; 3.2) | **0.0067** | 0.41 (0.41 – 0.41; 0) | 1 | 9.48 (5.72 – 14.29; 4.37) | **<0.001** |
|  |  | Right | 16.1 (10.28 – 24.27; 6.33) |  | 18.45 (11.12 – 22.27; 7.1) |  | 15.36 (10.79 – 23.29; 5.06) |  | 17.63 (10.14 – 24.44; 7.67) |  | 5.76 (5.76 – 5.76; 0) |  | 16.67 (10.74 – 24.76; 6.34) |  |
| **250 Hz Sampled Data** | | | | | | | | | | | | | | |
| % time rSO_2_ > 30% | Frontal | Left | 100 (100 – 100; 0) | **0.0031** | 100 (100 – 100; 0) | 1 | 100 (100 – 100; 0) | 0.0814 | 100 (100 – 100; 0) | **0.034** | 100 (100 – 100; 0) | 1 | 100 (100 – 100; 0) | **0.0071** |
|  |  | Right | 100 (100 – 100; 0) |  | 100 (100 – 100; 0) |  | 100 (100 – 100; 0) |  | 100 (99.85 – 100; 0) |  | 100 (100 – 100; 0) |  | 100 (100 – 100; 0) |  |
|  | Parietal | Left | 100 (100 – 100; 0) | 1 | 100 (100 – 100; 0) | 0.5807 | 100 (100 – 100; 0) | 0.3218 | 100 (100 – 100; 0) | 0.6099 | 100 (100 – 100; 0) | 1 | 100 (100 – 100; 0) | 0.7025 |
|  |  | Right | 100 (100 – 100; 0) |  | 100 (100 – 100; 0) |  | 100 (100 – 100; 0) |  | 100 (100 – 100; 0) |  | 100 (100 – 100; 0) |  | 100 (100 – 100; 0) |  |
|  | Temporal | Left | 100 (100 – 100; 0) | 0.59 | 100 (100 – 100; 0) | 0.3593 | 100 (100 – 100; 0) | 0.0814 | 100 (100 – 100; 0) | 0.3398 | 100 (100 – 100; 0) | 1 | 100 (100 – 100; 0) | 0.33 |
|  |  | Right | 100 (100 – 100; 0) |  | 100 (100 – 100; 0) |  | 100 (100 – 100; 0) |  | 100 (100 – 100; 0) |  | 100 (100 – 100; 0) |  | 100 (100 – 100; 0) |  |
|  | Occipital | Left | 100 (100 – 100; 0) | 0.5689 | 100 (100 – 100; 0) | 1 | 100 (100 – 100; 0) | 0.3349 | 100 (100 – 100; 0) | 0.1621 | 100 (100 – 100; 0) | 1 | 100 (100 – 100; 0) | 0.5677 |
|  |  | Right | 100 (100 – 100; 0) |  | 100 (100 – 100; 0) |  | 100 (100 – 100; 0) |  | 100 (100 – 100; 0) |  | 100 (100 – 100; 0) |  | 100 (100 – 100; 0) |  |
| % time rSO_2_ > 40% | Frontal | Left | 100 (100 – 100; 0) | **<0.001** | 100 (99.96 – 100; 0) | **0.0031** | 100 (100 – 100; 0) | **<0.001** | 100 (100 – 100; 0) | **<0.001** | 100 (100 – 100; 0) | 1 | 100 (100 – 100; 0) | **<0.001** |
|  |  | Right | 74.04 (2.6 – 98.94; 25.96) |  | 18.64 (0.66 – 98.85; 18.64) |  | 79.48 (4.29 – 99.06; 20.52) |  | 23.81 (0.96 – 96.38; 23.81) |  | 46.39 (46.39 – 46.39; 0) |  | 71.37 (0.75 – 98.95; 28.63) |  |
|  | Parietal | Left | 100 (66.14 – 100; 0) | 0.6842 | 100 (82.27 – 100; 0) | 0.3227 | 100 (83.7 – 100; 0) | 0.6892 | 100 (74.49 – 100; 0) | 0.8557 | 100 (100 – 100; 0) | 1 | 100 (72.5 – 100; 0) | 0.9841 |
|  |  | Right | 100 (92.51 – 100; 0) |  | 91.19 (85.28 – 99.86; 8.71) |  | 100 (86.44 – 100; 0) |  | 100 (89.93 – 100; 0) |  | 78.92 (78.92 – 78.92; 0) |  | 100 (89.71 – 100; 0) |  |
|  | Temporal | Left | 58.54 (17.24 – 98.38; 40.77) | **<0.001** | 64.22 (0 – 90.11; 32.38) | **0.0114** | 37.62 (0 – 84.4; 37.62) | **<0.001** | 71.66 (41.18 – 98.38; 27.84) | 0.0677 | 31.33 (31.33 – 31.33; 0) | 1 | 59.03 (4.81 – 94.85; 40.22) | **<0.001** |
|  |  | Right | 100 (100 – 100; 0) |  | 100 (84.94 – 100; 0) |  | 100 (100 – 100; 0) |  | 100 (4.43 – 100; 0) |  | 100 (100 – 100; 0) |  | 100 (100 – 100; 0) |  |
|  | Occipital | Left | 0 (0 – 0; 0) | **<0.001** | 0 (0 – 0.04; 0) | **<0.001** | 0 (0 – 0; 0) | **<0.001** | 0 (0 – 0; 0) | **<0.001** | 0 (0 – 0; 0) | 1 | 0 (0 – 0; 0) | **<0.001** |
|  |  | Right | 100 (94.19 – 100; 0) |  | 99.04 (93.41 – 100; 0.96) |  | 100 (99.96 – 100; 0) |  | 96.38 (71.58 – 100; 3.62) |  | 100 (100 – 100; 0) |  | 100 (93.43 – 100; 0) |  |
| % time rSO_2_ > 50% | Frontal | Left | 0 (0 – 89.16; 0) | **0.031** | 0 (0 – 4.89; 0) | 0.0786 | 0 (0 – 50.46; 0) | **0.0152** | 0 (0 – 88.4; 0) | 0.2855 | 0 (0 – 0; 0) | 1 | 0 (0 – 79.5; 0) | **0.0101** |
|  |  | Right | 0 (0 – 0.17; 0) |  | 0 (0 – 0; 0) |  | 0 (0 – 0; 0) |  | 0 (0 – 0.13; 0) |  | 0 (0 – 0; 0) |  | 0 (0 – 0; 0) |  |
|  | Parietal | Left | 52.31 (0.81 – 88.77; 43.91) | **<0.001** | 17.51 (7.88 – 45.66; 17.51) | 0.0746 | 59.15 (6.8 – 88.76; 38.84) | **<0.001** | 17.51 (2.26 – 58.26; 17.51) | 0.3759 | 95.98 (95.98 – 95.98; 0) | 1 | 34.08 (2.18 – 87.77; 34.08) | **<0.001** |
|  |  | Right | 0 (0 – 23.48; 0) |  | 5.85 (0 – 16.77; 5.85) |  | 0 (0 – 0.24; 0) |  | 12.07 (0.83 – 25.72; 12.07) |  | 0.2 (0.2 – 0.2; 0) |  | 0 (0 – 19.33; 0) |  |
|  | Temporal | Left | 0 (0 – 0; 0) | **<0.001** | 0 (0 – 0; 0) | 0.1058 | 0 (0 – 0; 0) | **<0.001** | 0 (0 – 0; 0) | **0.0204** | 0 (0 – 0; 0) | 1 | 0 (0 – 0; 0) | **<0.001** |
|  |  | Right | 16.54 (0 – 52.25; 16.54) |  | 0 (0 – 4.76; 0) |  | 16.54 (0 – 50.33; 16.54) |  | 0.09 (0 – 31.36; 0.09) |  | 9.44 (9.44 – 9.44; 0) |  | 1.22 (0 – 49.37; 1.22) |  |
|  | Occipital | Left | 0 (0 – 0; 0) | **<0.001** | 0 (0 – 0; 0) | **0.0165** | 0 (0 – 0; 0) | **<0.001** | 0 (0 – 0; 0) | **<0.001** | 0 (0 – 0; 0) | 1 | 0 (0 – 0; 0) | **<0.001** |
|  |  | Right | 2.81 (0 – 25.93; 2.81) |  | 0 (0 – 6.42; 0) |  | 0.54 (0 – 14.26; 0.54) |  | 4.22 (0 – 27.59; 4.22) |  | 0 (0 – 0; 0) |  | 1.93 (0 – 23.66; 1.93) |  |
| % time rSO_2_ > 60% | Frontal | Left | 0 (0 – 0; 0) | 0.9646 | 0 (0 – 0; 0) | 1 | 0 (0 – 0; 0) | 0.9561 | 0 (0 – 0; 0) | 1 | 0 (0 – 0; 0) | 1 | 0 (0 – 0; 0) | 0.9727 |
|  |  | Right | 0 (0 – 0; 0) |  | 0 (0 – 0; 0) |  | 0 (0 – 0; 0) |  | 0 (0 – 0; 0) |  | 0 (0 – 0; 0) |  | 0 (0 – 0; 0) |  |
|  | Parietal | Left | 0 (0 – 0; 0) | 0.1517 | 0 (0 – 0; 0) | 0.3593 | 0 (0 – 0; 0) | 0.2722 | 0 (0 – 0; 0) | 0.1621 | 0 (0 – 0; 0) | 1 | 0 (0 – 0; 0) | 0.0862 |
|  |  | Right | 0 (0 – 0; 0) |  | 0 (0 – 0; 0) |  | 0 (0 – 0; 0) |  | 0 (0 – 0; 0) |  | 0 (0 – 0; 0) |  | 0 (0 – 0; 0) |  |
|  | Temporal | Left | 0 (0 – 0; 0) | **0.0223** | 0 (0 – 0; 0) | 1 | 0 (0 – 0; 0) | 0.3042 | 0 (0 – 0; 0) | 0.0809 | 0 (0 – 0; 0) | 1 | 0 (0 – 0; 0) | **0.0485** |
|  |  | Right | 0 (0 – 0; 0) |  | 0 (0 – 0; 0) |  | 0 (0 – 0; 0) |  | 0 (0 – 0; 0) |  | 0 (0 – 0; 0) |  | 0 (0 – 0; 0) |  |
|  | Occipital | Left | 0 (0 – 0; 0) | **0.0223** | 0 (0 – 0; 0) | 1 | 0 (0 – 0; 0) | 0.1611 | 0 (0 – 0; 0) | 0.0809 | 0 (0 – 0; 0) | 1 | 0 (0 – 0; 0) | **0.023** |
|  |  | Right | 0 (0 – 0; 0) |  | 0 (0 – 0; 0) |  | 0 (0 – 0; 0) |  | 0 (0 – 0; 0) |  | 0 (0 – 0; 0) |  | 0 (0 – 0; 0) |  |
| % time COx-a > 0 | Frontal | Left | 49.06 (38.14 – 54.78; 9.52) | 0.2069 | 52.92 (47.22 – 54.69; 2.71) | 0.7075 | 48.81 (38.31 – 54.15; 6.93) | 0.476 | 50.97 (40.23 – 55.31; 7.66) | 0.5339 | 50.62 (50.62 – 50.62; 0) | 1 | 49.44 (38.4 – 55.1; 7.61) | 0.2646 |
|  |  | Right | 51.16 (44.68 – 56.88; 6.43) |  | 47.95 (44.38 – 52.09; 3.69) |  | 49.19 (45.21 – 54.76; 4.72) |  | 52.58 (42.14 – 57.7; 8.42) |  | 38.22 (38.22 – 38.22; 0) |  | 49.65 (44.51 – 57.4; 5.63) |  |
|  | Parietal | Left | 49.06 (43.59 – 56.4; 5.78) | 0.7278 | 50.53 (43.89 – 52.32; 4.29) | 0.6236 | 50.85 (43.93 – 55.31; 6.46) | 0.4559 | 48.22 (43.47 – 52.61; 4.71) | 0.9345 | 51.86 (51.86 – 51.86; 0) | 1 | 49.35 (43.58 – 53.9; 5.75) | 0.7438 |
|  |  | Right | 49.67 (43.42 – 57.33; 7.41) |  | 45.44 (41 – 53.38; 6.47) |  | 47.88 (42.96 – 55.42; 6.77) |  | 50.68 (41.31 – 57.66; 7.41) |  | 38.22 (38.22 – 38.22; 0) |  | 48.68 (43.34 – 57.57; 7.91) |  |
|  | Temporal | Left | 48.62 (44.47 – 56.09; 5.01) | 0.7435 | 54.95 (49.81 – 58.18; 5.71) | 0.665 | 48.54 (43.56 – 53.06; 5.01) | 0.2348 | 54.95 (47.44 – 59.26; 6.69) | 0.1489 | 47.11 (47.11 – 47.11; 0) | 1 | 50.38 (44.54 – 56.93; 5.96) | 0.7981 |
|  |  | Right | 51.89 (42.56 – 57.91; 8.67) |  | 49.92 (41.17 – 61.82; 10.23) |  | 53.85 (42.07 – 59.46; 10) |  | 47.47 (41.66 – 57.56; 7.81) |  | 74.79 (74.79 – 74.79; 0) |  | 51.2 (41.3 – 57.96; 8.69) |  |
|  | Occipital | Left | 50.78 (44.18 – 56.48; 6.34) | 0.9462 | 54.44 (39.49 – 59.6; 9.44) | 0.7508 | 49.6 (43.48 – 54.48; 5.47) | 0.5718 | 54.27 (46.89 – 60.88; 7.23) | 0.6138 | 34.92 (34.92 – 34.92; 0) | 1 | 50.99 (44.02 – 57.36; 6.93) | 0.9095 |
|  |  | Right | 49.02 (43.04 – 59.03; 8.5) |  | 50.23 (47.06 – 57.76; 4.72) |  | 49.17 (42.7 – 58.08; 8.38) |  | 50.23 (45.95 – 59.43; 5.17) |  | 33.26 (33.26 – 33.26; 0) |  | 49.35 (44.67 – 59.5; 7.14) |  |
| % time COx-a > 0.2 | Frontal | Left | 22.39 (16 – 31.29; 6.79) | 0.0996 | 23.47 (21 – 28.12; 3.63) | 0.7728 | 21.25 (15.69 – 27.67; 5.91) | 0.0883 | 27.1 (19.58 – 31.29; 5.65) | 0.4317 | 23.14 (23.14 – 23.14; 0) | 1 | 22.35 (16.41 – 29.78; 6.57) | 0.0555 |
|  |  | Right | 27.42 (21.18 – 35.82; 7.88) |  | 24.53 (20.92 – 28.29; 3.94) |  | 24.56 (20.78 – 30.97; 5.6) |  | 28.26 (21.48 – 35.82; 7.44) |  | 15.08 (15.08 – 15.08; 0) |  | 26.38 (21.24 – 35.46; 6.42) |  |
|  | Parietal | Left | 24.83 (16.55 – 28.17; 6.51) | 0.6217 | 24.13 (18.24 – 27.13; 3.37) | 0.7508 | 24.46 (15.29 – 28.4; 6.14) | 0.8763 | 24.5 (19.13 – 27.26; 3.26) | 0.4181 | 18.6 (18.6 – 18.6; 0) | 1 | 24.75 (17.56 – 28.1; 4.53) | 0.4952 |
|  |  | Right | 24.15 (18.78 – 32.26; 7.52) |  | 23.08 (17.08 – 29.21; 6.47) |  | 22.08 (16.03 – 30.44; 7.07) |  | 26.79 (20.51 – 31.91; 6.27) |  | 10.54 (10.54 – 10.54; 0) |  | 24.4 (18.29 – 32.05; 7.07) |  |
|  | Temporal | Left | 23.78 (18.05 – 29.35; 5.73) | 0.5748 | 23.54 (18.64 – 29.44; 5.46) | 0.7508 | 22.13 (17.89 – 27.22; 4.51) | 0.1083 | 25.36 (18.79 – 33.81; 6.88) | 0.33 | 15.7 (15.7 – 15.7; 0) | 1 | 24.14 (18.16 – 29.5; 5.98) | 0.8229 |
|  |  | Right | 25.76 (17.08 – 31.94; 6.59) |  | 23.69 (15.93 – 29.34; 7.87) |  | 26.44 (21.24 – 32.24; 5.63) |  | 22.81 (15.55 – 29.15; 7.24) |  | 35.12 (35.12 – 35.12; 0) |  | 24.9 (16.7 – 31.68; 7.22) |  |
|  | Occipital | Left | 16.78 (13.87 – 23.61; 4.83) | **0.001** | 20.92 (10.71 – 25.31; 6.05) | 0.2039 | 17.76 (12.65 – 23.72; 5.95) | **0.0069** | 17.56 (14.28 – 24.15; 4.79) | **0.0228** | 2.69 (2.69 – 2.69; 0) | 1 | 17.87 (13.76 – 24.32; 5.65) | **<0.001** |
|  |  | Right | 24.51 (18.54 – 36.24; 6.81) |  | 22.73 (19.77 – 28.53; 5.31) |  | 22.88 (19.02 – 30.39; 6.44) |  | 24.51 (18.1 – 33.22; 6.92) |  | 10.12 (10.12 – 10.12; 0) |  | 23.93 (18.97 – 33.08; 6.21) |  |
| % time COx-a > 0.3 | Frontal | Left | 12.3 (7.99 – 21.17; 5.12) | 0.1229 | 14.65 (10.58 – 15.87; 1.8) | 0.5067 | 11.4 (8.22 – 16.13; 3.79) | 0.1296 | 15.84 (9.92 – 19.84; 5.68) | 0.3418 | 12.6 (12.6 – 12.6; 0) | 1 | 13.56 (8.32 – 18.16; 5.24) | 0.0533 |
|  |  | Right | 17.29 (12.65 – 24.33; 6.59) |  | 15.09 (11.16 – 18.4; 4.27) |  | 15.53 (11.16 – 20.73; 5.27) |  | 17.29 (13.63 – 25.17; 6.59) |  | 4.55 (4.55 – 4.55; 0) |  | 16.77 (11.78 – 23.58; 5.09) |  |
|  | Parietal | Left | 15.43 (9.19 – 19.34; 4.62) | 0.7751 | 14.78 (10.23 – 17.8; 3.98) | 0.8852 | 15.44 (8.05 – 19.18; 5.93) | 0.7246 | 14.77 (11.25 – 17.75; 3.48) | 0.474 | 7.23 (7.23 – 7.23; 0) | 1 | 14.82 (10.62 – 19.11; 4.28) | 0.9264 |
|  |  | Right | 15.49 (9.42 – 23.71; 6.38) |  | 12.82 (8.29 – 18.25; 5.98) |  | 12.02 (7.9 – 18.39; 5.46) |  | 16.01 (12.99 – 21.28; 4.77) |  | 6.2 (6.2 – 6.2; 0) |  | 15.34 (9.25 – 21.43; 6.09) |  |
|  | Temporal | Left | 14.08 (8.94 – 18.26; 5.01) | 0.4735 | 10.07 (7.02 – 17.43; 4.51) | 0.8399 | 11.69 (8.76 – 16.96; 3.34) | 0.0768 | 15.43 (7.85 – 20.65; 7.2) | 0.4887 | 5.79 (5.79 – 5.79; 0) | 1 | 13.88 (8.81 – 18.39; 4.9) | 0.6065 |
|  |  | Right | 16.41 (10.04 – 20.26; 5.81) |  | 11.92 (8.71 – 18.97; 6.29) |  | 17.02 (10.62 – 20.27; 5.24) |  | 12.47 (7.65 – 19.27; 6.33) |  | 17.98 (17.98 – 17.98; 0) |  | 15.21 (9.49 – 19.83; 5.21) |  |
|  | Occipital | Left | 7.86 (5.41 – 11.7; 3.01) | **<0.001** | 8.09 (4.38 – 12.19; 3.87) | **0.0051** | 8.5 (5.97 – 13.33; 3.24) | **<0.001** | 6.3 (4.98 – 11.15; 2.65) | **<0.001** | 0.21 (0.21 – 0.21; 0) | 1 | 7.89 (5.35 – 11.73; 3.28) | **<0.001** |
|  |  | Right | 14.09 (11.17 – 23.93; 5.73) |  | 15.46 (11.93 – 21.52; 5.43) |  | 14.38 (12.24 – 20.22; 4.2) |  | 16.12 (9.37 – 24.14; 7.8) |  | 5.99 (5.99 – 5.99; 0) |  | 15.07 (11.67 – 24.11; 5.58) |  |
| % time HbOx > 0 | Frontal | Left | 48.7 (39.9 – 55.9; 8.59) | **0.0484** | 48.39 (43.7 – 49.66; 3.17) | 0.6033 | 47.42 (39.13 – 55.45; 8.07) | 0.0677 | 49.19 (44.92 – 50.97; 3.72) | 0.4181 | 68.6 (68.6 – 68.6; 0) | 1 | 48.21 (40.54 – 54.12; 7.23) | **0.0248** |
|  |  | Right | 51.7 (46.57 – 60.44; 7.3) |  | 49.11 (46.57 – 52.74; 4.04) |  | 50.32 (47.21 – 59.07; 5.95) |  | 51.6 (44.79 – 56.06; 5.89) |  | 43.8 (43.8 – 43.8; 0) |  | 51.28 (47.16 – 58.16; 6.33) |  |
|  | Parietal | Left | 52.04 (46.65 – 58.79; 6.42) | 0.8967 | 47.31 (36.62 – 52.17; 8.09) | 0.1749 | 51.35 (43.43 – 59.4; 8.33) | 0.9543 | 49.59 (45.57 – 54.85; 5.19) | 0.4317 | 52.27 (52.27 – 52.27; 0) | 1 | 50.82 (43.79 – 55.67; 6.71) | 0.5843 |
|  |  | Right | 52.29 (45.18 – 59.71; 7.26) |  | 52.14 (48.15 – 53.96; 2.28) |  | 52.14 (46.79 – 57.39; 5.92) |  | 52.28 (43.77 – 59.53; 7.41) |  | 51.03 (51.03 – 51.03; 0) |  | 52.77 (45.09 – 59.4; 7.04) |  |
|  | Temporal | Left | 50.69 (43.07 – 57.33; 7.05) | 0.2183 | 53.69 (41.7 – 56.79; 5.5) | 0.4188 | 49.35 (42.35 – 56.55; 7.14) | 0.1403 | 53.69 (43.07 – 57.34; 5.96) | 0.6985 | 51.03 (51.03 – 51.03; 0) | 1 | 51.6 (42.4 – 56.91; 7.44) | 0.6315 |
|  |  | Right | 54.6 (43.7 – 59.58; 7.22) |  | 44.48 (38.19 – 51.82; 8.47) |  | 55.11 (45.48 – 59.82; 7.22) |  | 47.25 (39.82 – 57.22; 8.83) |  | 67.98 (67.98 – 67.98; 0) |  | 53.03 (41.39 – 59.08; 7.44) |  |
|  | Occipital | Left | 52.62 (46.73 – 57.16; 4.64) | 0.6439 | 54.02 (41.21 – 56.84; 6.86) | 0.5834 | 53.47 (46.12 – 56; 4.26) | 0.2905 | 53.83 (46.73 – 58.59; 5.89) | 0.8973 | 48.76 (48.76 – 48.76; 0) | 1 | 53.62 (46.28 – 57.19; 5.62) | 0.5673 |
|  |  | Right | 51.03 (44.18 – 57.78; 6.95) |  | 50.74 (45.98 – 54.21; 4.47) |  | 48.52 (43.24 – 56.44; 7.63) |  | 53.24 (45.09 – 57.78; 8.09) |  | 44.01 (44.01 – 44.01; 0) |  | 51.06 (44.72 – 57.37; 6.35) |  |
| % time HbOx > 0.2 | Frontal | Left | 22.96 (16.97 – 29.07; 6.13) | **0.0197** | 22.83 (19.92 – 25.23; 3.21) | 0.0885 | 22.07 (16.32 – 27.09; 5.43) | **0.0074** | 24.02 (21.47 – 27.77; 3.38) | 0.2549 | 32.85 (32.85 – 32.85; 0) | 1 | 22.72 (17.3 – 27.11; 4.45) | **0.0024** |
|  |  | Right | 27.82 (22.89 – 37.24; 6.75) |  | 25.8 (21.87 – 29.19; 3.72) |  | 26.53 (22.92 – 32.01; 4.78) |  | 28.14 (21.75 – 31.81; 6.04) |  | 17.56 (17.56 – 17.56; 0) |  | 27.27 (22.8 – 31.94; 4.66) |  |
|  | Parietal | Left | 27.87 (20.32 – 34.54; 6.81) | 0.5713 | 23.39 (17.39 – 28.5; 6.37) | 0.1939 | 25.97 (17.3 – 34.16; 8.47) | 0.5069 | 27.97 (22.43 – 31.93; 5.06) | 0.4597 | 19.63 (19.63 – 19.63; 0) | 1 | 27.46 (20.08 – 32.72; 6.89) | 0.3268 |
|  |  | Right | 27.01 (21.71 – 37.08; 8.09) |  | 26.13 (22.53 – 33; 4.91) |  | 25.8 (22.12 – 34.41; 5.97) |  | 29.01 (21.86 – 35.02; 6.93) |  | 16.74 (16.74 – 16.74; 0) |  | 26.65 (22.32 – 35.36; 6.69) |  |
|  | Temporal | Left | 24.09 (19.53 – 30.23; 5.55) | 0.2685 | 20.01 (17.85 – 23.94; 2.2) | 0.8399 | 23.37 (19.41 – 27.45; 4.24) | 0.1176 | 21.33 (17.74 – 30.84; 5.93) | 0.9719 | 21.49 (21.49 – 21.49; 0) | 1 | 23.02 (18.09 – 29.96; 5.29) | 0.4018 |
|  |  | Right | 27.4 (21.77 – 35.7; 8.29) |  | 22.94 (14.47 – 27.24; 4.55) |  | 27.63 (22.54 – 32.61; 5.3) |  | 22.94 (16.34 – 30.87; 8.07) |  | 40.5 (40.5 – 40.5; 0) |  | 25.96 (19.01 – 31.99; 6.24) |  |
|  | Occipital | Left | 21.96 (17.47 – 29.48; 5.89) | 0.2164 | 21.03 (13.11 – 26.89; 7.68) | 0.1124 | 23.77 (18.15 – 30.14; 6.02) | 0.6405 | 20.57 (15.1 – 26.9; 6.1) | **0.0346** | 15.08 (15.08 – 15.08; 0) | 1 | 21.92 (17.44 – 28.76; 6.19) | 0.0673 |
|  |  | Right | 24.97 (19.93 – 30.47; 5.51) |  | 25.93 (22.37 – 30.56; 4.85) |  | 24.63 (20.93 – 30.24; 5.56) |  | 27.07 (20.57 – 31; 5.86) |  | 13.64 (13.64 – 13.64; 0) |  | 25.55 (21 – 30.59; 5.04) |  |
| % time HbOx > 0.3 | Frontal | Left | 14.4 (8.51 – 18.94; 5.87) | **0.0226** | 13.06 (10.01 – 16.18; 3.25) | **0.0194** | 10.67 (6.11 – 17.19; 5.77) | **0.0108** | 15.71 (12.41 – 16.68; 2.8) | 0.098 | 17.77 (17.77 – 17.77; 0) | 1 | 13.4 (9.12 – 16.7; 3.87) | **0.0021** |
|  |  | Right | 16.75 (12.67 – 24.57; 6.54) |  | 17.05 (15.17 – 18.37; 1.84) |  | 15.78 (12.48 – 23.66; 4.77) |  | 17.63 (13.68 – 23.89; 4.36) |  | 9.5 (9.5 – 9.5; 0) |  | 17.02 (13.5 – 23.9; 4.75) |  |
|  | Parietal | Left | 17.1 (11.34 – 23; 5.98) | 0.629 | 15.85 (10.34 – 19.67; 4.48) | 0.4025 | 16.3 (9.73 – 20.58; 5.79) | 0.676 | 17.63 (15.01 – 22.62; 4.54) | 0.8603 | 7.02 (7.02 – 7.02; 0) | 1 | 17.02 (11.23 – 21.93; 5.39) | 0.4514 |
|  |  | Right | 17.56 (13.18 – 26.43; 6.25) |  | 17.91 (15.53 – 20.6; 2.7) |  | 17.29 (12.6 – 22.33; 5.55) |  | 20.26 (14.57 – 21.42; 5.5) |  | 3.72 (3.72 – 3.72; 0) |  | 17.72 (14.29 – 21.81; 4.09) |  |
|  | Temporal | Left | 15.02 (11.65 – 19.87; 4.32) | 0.4864 | 10.29 (7.5 – 13.87; 3.1) | 0.931 | 14.81 (9.69 – 17.94; 4.75) | 0.2513 | 14.02 (10.47 – 17.86; 3.97) | 0.6726 | 7.23 (7.23 – 7.23; 0) | 1 | 14.79 (9.93 – 17.88; 4.57) | 0.6493 |
|  |  | Right | 17.5 (11.63 – 23.29; 5.94) |  | 13.41 (5.28 – 16.75; 4.14) |  | 17.66 (12.13 – 22.23; 4.89) |  | 13.92 (8.24 – 17.39; 5.15) |  | 25.41 (25.41 – 25.41; 0) |  | 16.37 (9.81 – 21.07; 6.07) |  |
|  | Occipital | Left | 13.3 (7.35 – 20.61; 6.32) | 0.0748 | 9.91 (6.31 – 12.98; 4.02) | **0.0209** | 13.73 (9.31 – 20.31; 5.28) | 0.2513 | 10.63 (4.8 – 14.99; 4.58) | **0.0124** | 2.27 (2.27 – 2.27; 0) | 1 | 12.89 (7.11 – 18.36; 5.77) | **0.0111** |
|  |  | Right | 16.77 (11.1 – 22.65; 5.98) |  | 18.17 (13.27 – 20.53; 2.92) |  | 16.96 (12.41 – 20.17; 3.95) |  | 17.87 (11.04 – 21.35; 5.79) |  | 7.23 (7.23 – 7.23; 0) |  | 17.04 (11.85 – 21.11; 4.44) |  |
| % time HHbx > 0 | Frontal | Left | 51.57 (45.2 – 58.63; 6.91) | 0.971 | 47.31 (44.32 – 51.28; 3.59) | 0.7075 | 50.01 (43.89 – 60.27; 8.56) | 0.4363 | 49.12 (45.2 – 53.78; 4.63) | 0.6304 | 63.84 (63.84 – 63.84; 0) | 1 | 49.2 (44.42 – 56.18; 5.14) | 0.8064 |
|  |  | Right | 52.06 (42.63 – 56.99; 7.31) |  | 53.26 (43.37 – 55.98; 6.33) |  | 53.1 (42.56 – 59.3; 6.27) |  | 48.47 (42.91 – 56.3; 7.14) |  | 59.3 (59.3 – 59.3; 0) |  | 52.08 (42.63 – 56.79; 7.35) |  |
|  | Parietal | Left | 50.39 (42.28 – 58.13; 8.35) | 0.5365 | 48.4 (46.4 – 49; 1.04) | 0.0531 | 50.1 (41.12 – 57.43; 9.03) | 0.3215 | 48.65 (46.39 – 51.97; 2.65) | 0.2549 | 49.79 (49.79 – 49.79; 0) | 1 | 48.86 (43.44 – 56.02; 5.47) | 0.1863 |
|  |  | Right | 51.36 (45.41 – 57.85; 6.21) |  | 55.07 (50.87 – 58.54; 4.46) |  | 53.32 (45.26 – 57.62; 7.69) |  | 52.69 (46.34 – 58.2; 6.12) |  | 64.67 (64.67 – 64.67; 0) |  | 52.48 (45.91 – 57.7; 6.42) |  |
|  | Temporal | Left | 51.77 (45.14 – 57.3; 6.05) | 0.7045 | 43.52 (40.93 – 47.9; 3.23) | 0.4025 | 52.02 (45.79 – 56.78; 5.68) | 0.8505 | 45.61 (41.34 – 52.67; 5.58) | 0.2962 | 50.62 (50.62 – 50.62; 0) | 1 | 49.9 (42.5 – 56.5; 7.27) | 0.4818 |
|  |  | Right | 51.13 (46.64 – 58.38; 5.62) |  | 47.05 (41.71 – 52.23; 5.7) |  | 51.01 (46.44 – 56.73; 5.16) |  | 49.94 (41.51 – 59.84; 9.71) |  | 51.03 (51.03 – 51.03; 0) |  | 50.58 (44.83 – 58.19; 6.22) |  |
|  | Occipital | Left | 52.32 (47.73 – 58.69; 5.29) | 0.6513 | 54.16 (40.39 – 56.94; 9.86) | 0.3555 | 55.09 (49.17 – 60.22; 6.17) | 0.1195 | 49.25 (40.88 – 55.74; 7.55) | 0.5652 | 76.03 (76.03 – 76.03; 0) | 1 | 52.41 (47.4 – 57.37; 5.01) | 0.4366 |
|  |  | Right | 52.19 (45.95 – 58.84; 6.59) |  | 48.68 (44.15 – 53.45; 5.07) |  | 52.37 (45.45 – 57.32; 6.21) |  | 51.03 (45.73 – 56.78; 5.69) |  | 58.88 (58.88 – 58.88; 0) |  | 51.59 (45.71 – 56.8; 5.8) |  |
| % time HHbx > 0.2 | Frontal | Left | 26.15 (20.09 – 32.01; 6.14) | 0.5926 | 23.37 (20.76 – 26.64; 2.96) | 0.126 | 23.67 (19.78 – 31.7; 5.08) | 0.2617 | 26.06 (21.88 – 30.64; 4.58) | 0.6641 | 28.93 (28.93 – 28.93; 0) | 1 | 25.41 (19.96 – 31.55; 5.53) | 0.2739 |
|  |  | Right | 27.37 (22.26 – 34.28; 5.6) |  | 27.68 (25.29 – 30.83; 2.55) |  | 27.27 (22.67 – 30.93; 4.68) |  | 27.5 (23.12 – 33.28; 5.27) |  | 29.13 (29.13 – 29.13; 0) |  | 26.88 (22.55 – 32.86; 4.73) |  |
|  | Parietal | Left | 26.83 (17.29 – 33.07; 7.8) | 0.7356 | 23.77 (19.82 – 25.38; 2.85) | **0.0086** | 25.11 (16.61 – 27.89; 8.34) | 0.3987 | 26.37 (21 – 32.24; 6.19) | 0.2267 | 21.9 (21.9 – 21.9; 0) | 1 | 25.26 (18.48 – 30.14; 6.71) | 0.1935 |
|  |  | Right | 25 (21.38 – 32.58; 4.81) |  | 29.83 (27.88 – 36.16; 4.7) |  | 24.8 (21.07 – 30.31; 4.54) |  | 29.77 (25.54 – 36.02; 6.12) |  | 31.82 (31.82 – 31.82; 0) |  | 26.29 (21.65 – 32.91; 5.85) |  |
|  | Temporal | Left | 26.56 (21.04 – 30.5; 5.39) | 0.8639 | 19.42 (16.48 – 25.59; 5.24) | 0.4025 | 26.96 (21.2 – 30.54; 5.26) | 0.8377 | 21.88 (16.9 – 27.52; 5.63) | 0.4887 | 21.28 (21.28 – 21.28; 0) | 1 | 24.69 (18.15 – 30.49; 5.99) | 0.5179 |
|  |  | Right | 26.88 (19.21 – 32.57; 6.39) |  | 21.95 (19.46 – 28.96; 5.72) |  | 29.07 (18.91 – 32.45; 4.99) |  | 23.79 (20.43 – 29.96; 5.67) |  | 18.18 (18.18 – 18.18; 0) |  | 26.52 (19.4 – 32.4; 6.24) |  |
|  | Occipital | Left | 20.25 (16.38 – 26.09; 4.82) | **0.0041** | 14.79 (11.47 – 25.18; 5.95) | 0.2145 | 21.68 (15.12 – 27.82; 6.35) | 0.0809 | 17.95 (14.47 – 22; 3.99) | **0.0035** | 28.31 (28.31 – 28.31; 0) | 1 | 19.85 (14.15 – 25.77; 5.92) | **0.0019** |
|  |  | Right | 26.86 (21.39 – 32.43; 5.61) |  | 23.13 (16.86 – 27.67; 5.71) |  | 26.1 (19.5 – 32.14; 6.06) |  | 26.17 (20.11 – 32.88; 7.23) |  | 24.17 (24.17 – 24.17; 0) |  | 26.14 (19.72 – 32.2; 6.36) |  |
| % time HHbx > 0.3 | Frontal | Left | 15.68 (12.03 – 22.57; 4.64) | 0.5677 | 14.13 (11.65 – 17.61; 3.45) | **0.0282** | 13.83 (11.99 – 18.71; 2.82) | 0.1381 | 17.65 (12.02 – 24.21; 6.52) | 0.5262 | 14.46 (14.46 – 14.46; 0) | 1 | 15.41 (11.98 – 22.2; 4.39) | 0.1375 |
|  |  | Right | 16.44 (13.4 – 23.37; 6.65) |  | 18.73 (17.9 – 21.87; 2.2) |  | 16.3 (14.11 – 21.29; 4.38) |  | 18.73 (16.11 – 24.71; 5.56) |  | 7.85 (7.85 – 7.85; 0) |  | 17.98 (14.42 – 23.18; 4.17) |  |
|  | Parietal | Left | 16.85 (8.84 – 23.29; 7.14) | 0.5229 | 15.14 (10.9 – 16.6; 2.2) | **0.0102** | 13.63 (7.89 – 18.21; 5.31) | 0.147 | 17.58 (13.85 – 22.5; 4.64) | 0.2649 | 8.06 (8.06 – 8.06; 0) | 1 | 16.38 (9.45 – 19.74; 6.57) | 0.1197 |
|  |  | Right | 16.15 (13.23 – 22.57; 3.7) |  | 21.9 (17.06 – 24.75; 3.14) |  | 15.42 (12.81 – 21.84; 3.76) |  | 21.57 (15.03 – 26.89; 5.96) |  | 16.74 (16.74 – 16.74; 0) |  | 17.95 (13.21 – 24.7; 5.14) |  |
|  | Temporal | Left | 15.78 (9.97 – 20.95; 5.47) | 0.5297 | 12.06 (8.13 – 16.68; 5.31) | 0.4025 | 16.3 (11.77 – 21.23; 4.95) | 0.4412 | 12.93 (8.84 – 17.33; 4.28) | 0.4668 | 8.88 (8.88 – 8.88; 0) | 1 | 15.29 (9.85 – 20.4; 5.45) | 0.2801 |
|  |  | Right | 17.3 (10.01 – 24.27; 7.05) |  | 15.32 (9.81 – 20.23; 5.09) |  | 18.28 (9.93 – 24.05; 5.95) |  | 15.32 (9.64 – 21.28; 6.28) |  | 8.47 (8.47 – 8.47; 0) |  | 16.81 (10.05 – 24.01; 7.2) |  |
|  | Occipital | Left | 10.22 (5.71 – 14.74; 4.72) | **<0.001** | 7.17 (2.25 – 12.77; 5.03) | **0.0304** | 12.37 (5.92 – 15.64; 5.5) | **0.0264** | 8.53 (3.46 – 11.86; 4.15) | **<0.001** | 18.8 (18.8 – 18.8; 0) | 1 | 9.83 (5.38 – 13.24; 4.42) | **<0.001** |
|  |  | Right | 17.17 (11.92 – 21.88; 5.04) |  | 13.18 (9.49 – 21.32; 4.55) |  | 16.79 (10.1 – 22.32; 6) |  | 15.98 (12.13 – 21.58; 5.27) |  | 11.78 (11.78 – 11.78; 0) |  | 16 (11.46 – 21.9; 5.84) |  |
| % time tHbx > 0 | Frontal | Left | 49.27 (43.04 – 59.16; 8.66) | 0.3366 | 47.72 (45.06 – 51.48; 3.31) | 0.977 | 47.54 (39.9 – 58.24; 8.97) | 0.1661 | 50.39 (45.42 – 56.14; 5.43) | 0.8053 | 67.77 (67.77 – 67.77; 0) | 1 | 48.12 (44.2 – 56.35; 6.91) | 0.2897 |
|  |  | Right | 53.06 (46.34 – 60.74; 7.59) |  | 47.28 (42.49 – 54.59; 6.43) |  | 52.12 (46.66 – 57.63; 5.54) |  | 49.21 (40.84 – 60.17; 10.12) |  | 47.52 (47.52 – 47.52; 0) |  | 51.35 (43.8 – 59.28; 7.93) |  |
|  | Parietal | Left | 50.73 (45.66 – 57.75; 5.87) | 0.9669 | 47.02 (41.59 – 49.37; 3.68) | **0.0102** | 51.11 (45.27 – 63.88; 7.4) | 0.7932 | 48.69 (44.48 – 51; 3.83) | 0.098 | 46.69 (46.69 – 46.69; 0) | 1 | 49.6 (45.08 – 53.78; 4.52) | 0.2946 |
|  |  | Right | 50.26 (45.82 – 59.18; 6.11) |  | 55.08 (48.53 – 59.28; 5.72) |  | 51.75 (47.53 – 58.95; 6.29) |  | 52.25 (46.23 – 59.57; 6.66) |  | 59.3 (59.3 – 59.3; 0) |  | 50.29 (46.4 – 59.01; 5.93) |  |
|  | Temporal | Left | 51.76 (44.37 – 59.6; 7.8) | 0.8152 | 47.36 (45.17 – 53.68; 4.33) | 0.2366 | 50.95 (45.17 – 57.83; 6.15) | 0.5718 | 49.82 (43.07 – 58.07; 7.79) | 0.4455 | 52.27 (52.27 – 52.27; 0) | 1 | 50.66 (44.4 – 58.12; 7.27) | 0.8395 |
|  |  | Right | 54.11 (48.34 – 58.43; 5.12) |  | 44.24 (40.83 – 49.53; 4.85) |  | 54.11 (48.1 – 58.67; 5.01) |  | 48.31 (43.75 – 54.58; 6.1) |  | 62.19 (62.19 – 62.19; 0) |  | 52.44 (44.13 – 56.2; 6.19) |  |
|  | Occipital | Left | 50.92 (46.57 – 58.89; 5.52) | 0.7831 | 54.27 (49.43 – 55.33; 2.6) | 0.3408 | 53.37 (47.3 – 59.75; 6.62) | 0.5069 | 50.75 (46.57 – 54.92; 4.54) | 0.6985 | 62.4 (62.4 – 62.4; 0) | 1 | 51.07 (46.18 – 56.79; 5.24) | 0.9208 |
|  |  | Right | 52.1 (44.66 – 61.13; 7.95) |  | 50.65 (47.12 – 54.26; 4.32) |  | 51.05 (43.5 – 57.95; 7.59) |  | 51.55 (45.37 – 58.86; 6.68) |  | 52.48 (52.48 – 52.48; 0) |  | 51.38 (44.44 – 59.03; 7.53) |  |
| % time tHbx > 0.2 | Frontal | Left | 24.3 (18.44 – 33.39; 7.63) | 0.4513 | 23.85 (18.75 – 24.42; 4.13) | **0.0404** | 23.5 (17.2 – 28.93; 5.96) | 0.0677 | 24.53 (22.19 – 32.96; 5.96) | 0.9532 | 39.26 (39.26 – 39.26; 0) | 1 | 23.95 (18.75 – 30.68; 5.87) | 0.1068 |
|  |  | Right | 27.36 (19.63 – 36.24; 8.21) |  | 27.56 (21.86 – 30.45; 4.43) |  | 29.28 (22.02 – 32.57; 6.59) |  | 25.93 (19.63 – 35.1; 7.48) |  | 14.88 (14.88 – 14.88; 0) |  | 27.73 (20.27 – 33.86; 6.6) |  |
|  | Parietal | Left | 27.51 (20.33 – 35.46; 7.77) | 0.7991 | 23.37 (17.18 – 26.55; 5.45) | **0.0141** | 25.84 (18.15 – 34.8; 8.48) | 0.8763 | 25.9 (20.73 – 31.69; 5.72) | 0.1771 | 14.67 (14.67 – 14.67; 0) | 1 | 25.92 (20.12 – 33.63; 7.56) | 0.5365 |
|  |  | Right | 28.1 (20.27 – 33.39; 5.81) |  | 31.2 (28.72 – 34.03; 3.02) |  | 28.28 (21.42 – 31.46; 4.9) |  | 30.33 (26.23 – 34.93; 4.48) |  | 28.1 (28.1 – 28.1; 0) |  | 29.13 (23.02 – 33.85; 5.39) |  |
|  | Temporal | Left | 27.1 (21.19 – 30.83; 5.77) | 0.7162 | 19.72 (17.58 – 26.3; 3.61) | 0.7949 | 25.08 (20.51 – 29.99; 5.12) | 0.4509 | 24.31 (19.69 – 31.9; 5.06) | 0.7513 | 23.76 (23.76 – 23.76; 0) | 1 | 26.06 (19.6 – 30.6; 6.1) | 0.9349 |
|  |  | Right | 27.94 (22.47 – 34.17; 6.22) |  | 22.05 (15.38 – 26.87; 6.52) |  | 27.67 (23.03 – 31.42; 3.96) |  | 24.69 (16.69 – 31.43; 8.09) |  | 30.37 (30.37 – 30.37; 0) |  | 26.59 (19.47 – 31.62; 6.17) |  |
|  | Occipital | Left | 24.4 (19.72 – 31.04; 5.89) | 0.4328 | 21.63 (18.6 – 25.08; 3.8) | 0.1332 | 24.62 (20.17 – 31.59; 5.81) | 0.7062 | 21.89 (17.85 – 28.71; 4.92) | 0.098 | 30.58 (30.58 – 30.58; 0) | 1 | 22.63 (19.15 – 29.43; 5.66) | 0.1052 |
|  |  | Right | 26.58 (19.44 – 32.67; 7.1) |  | 26.27 (22.96 – 30.8; 5.18) |  | 25.79 (18.94 – 32.37; 6.81) |  | 27.23 (21.37 – 32.15; 6.01) |  | 16.53 (16.53 – 16.53; 0) |  | 27.21 (19.57 – 32.77; 6.41) |  |
| % time tHbx > 0.3 | Frontal | Left | 15.89 (9.62 – 22.37; 6.64) | 0.2965 | 12.5 (9.24 – 14.81; 3.32) | **<0.001** | 12.36 (7.79 – 18.54; 4.79) | **0.0137** | 16.79 (11.47 – 23.6; 6.57) | 0.6472 | 20.66 (20.66 – 20.66; 0) | 1 | 13.81 (9.3 – 20.05; 5.2) | **0.0198** |
|  |  | Right | 16.94 (10.19 – 25.44; 7.36) |  | 19.13 (16.24 – 22.11; 3.38) |  | 17.28 (13.9 – 23.37; 5.57) |  | 18.33 (12.19 – 23.77; 6.31) |  | 7.23 (7.23 – 7.23; 0) |  | 17.36 (13.58 – 23.48; 5.42) |  |
|  | Parietal | Left | 18.45 (11.26 – 25.11; 6.87) | 0.9462 | 15.02 (11.3 – 18.28; 3.36) | **0.0404** | 17.65 (9.08 – 21.34; 7.19) | 0.9804 | 17.83 (11.98 – 24.17; 6.1) | 0.3072 | 3.1 (3.1 – 3.1; 0) | 1 | 17.76 (11.55 – 23.5; 6.22) | 0.4818 |
|  |  | Right | 17.66 (11.31 – 24.01; 6.51) |  | 20.35 (16.85 – 23.21; 3.72) |  | 16.49 (10.19 – 20.45; 5.06) |  | 20.8 (15.57 – 24.6; 4.69) |  | 8.06 (8.06 – 8.06; 0) |  | 18.92 (12.72 – 24.14; 5.83) |  |
|  | Temporal | Left | 16.22 (12.72 – 21.7; 4.35) | 0.7791 | 10.87 (7.48 – 15.7; 4.05) | 0.665 | 15.22 (11.32 – 21.35; 5.04) | 0.5017 | 14.98 (12.23 – 19.86; 3.26) | 0.9532 | 11.36 (11.36 – 11.36; 0) | 1 | 15.22 (11.88 – 21.28; 3.93) | 0.6933 |
|  |  | Right | 18.57 (11.23 – 24.8; 6.69) |  | 12.88 (7.84 – 18.87; 5.76) |  | 18.26 (10.2 – 20.88; 5.84) |  | 17.62 (8.56 – 22.65; 7.49) |  | 16.32 (16.32 – 16.32; 0) |  | 18.53 (9.47 – 21.82; 6.72) |  |
|  | Occipital | Left | 14.59 (11.42 – 20.85; 4.32) | 0.2467 | 13.18 (8.61 – 14.81; 3.88) | 0.0885 | 14.9 (13.1 – 21.27; 4.1) | 0.5069 | 13.65 (8.81 – 16.2; 4.82) | **0.05** | 18.8 (18.8 – 18.8; 0) | 1 | 14.01 (10.6 – 19.58; 3.9) | 0.0533 |
|  |  | Right | 17.3 (13.16 – 22.11; 4.37) |  | 17.22 (11.74 – 20.43; 5.2) |  | 16.81 (13.28 – 21.7; 3.84) |  | 17.49 (12.38 – 22.06; 5.1) |  | 6.82 (6.82 – 6.82; 0) |  | 17.35 (13.14 – 22.34; 4.64) |  |
| % time HbDiffx > 0 | Frontal | Left | 48.5 (39.84 – 56.55; 8.55) | 0.1179 | 52.31 (44.54 – 55.19; 3.98) | 0.6236 | 48.39 (39.22 – 55.47; 7.96) | 0.248 | 51.68 (41.74 – 56.47; 6.37) | 0.5339 | 53.93 (53.93 – 53.93; 0) | 1 | 48.71 (40.24 – 56.03; 7.95) | 0.1394 |
|  |  | Right | 53.19 (47.57 – 59.18; 6.27) |  | 49.82 (41.34 – 53.5; 7.8) |  | 50.32 (46.73 – 55.08; 4.82) |  | 52.52 (44.42 – 58.64; 6.88) |  | 39.88 (39.88 – 39.88; 0) |  | 51.77 (46.15 – 57.45; 5.68) |  |
|  | Parietal | Left | 50.42 (43.72 – 57.05; 7.08) | 0.5572 | 49.73 (44.59 – 52.16; 4.04) | 1 | 51.26 (43 – 56.49; 6.57) | 0.3807 | 49.75 (45.58 – 53.94; 4.3) | 0.8053 | 52.07 (52.07 – 52.07; 0) | 1 | 50.19 (43.18 – 56.08; 6.02) | 0.7224 |
|  |  | Right | 49.07 (43.28 – 57.52; 8.04) |  | 46.63 (42.77 – 54.34; 7.8) |  | 48.69 (43.91 – 53.14; 4.72) |  | 53.41 (41.83 – 57.9; 8.83) |  | 37.6 (37.6 – 37.6; 0) |  | 49 (44.13 – 57.14; 7.75) |  |
|  | Temporal | Left | 51.09 (44.31 – 57.5; 6.8) | 0.8313 | 54 (49.96 – 58.21; 4.28) | 0.5444 | 50.3 (43.27 – 57.24; 7.09) | 0.4126 | 54 (48.83 – 59.12; 5.59) | 0.1967 | 47.73 (47.73 – 47.73; 0) | 1 | 51.19 (45.21 – 57.76; 6.57) | 0.6239 |
|  |  | Right | 52.3 (42.13 – 56.77; 7) |  | 46.31 (41.89 – 60.9; 9.78) |  | 54.2 (42.02 – 57.62; 9.05) |  | 48.88 (42.14 – 54.79; 6.61) |  | 78.1 (78.1 – 78.1; 0) |  | 52.09 (42.01 – 57.14; 8.38) |  |
|  | Occipital | Left | 51.23 (46.79 – 56.79; 5.14) | 0.5028 | 54.22 (38.92 – 58.28; 9.37) | 0.8173 | 50.33 (44.53 – 54.31; 4.78) | 0.9347 | 55.38 (48.65 – 60.45; 6.28) | 0.6641 | 37.19 (37.19 – 37.19; 0) | 1 | 52.08 (46.51 – 56.9; 5.5) | 0.6673 |
|  |  | Right | 49.02 (43.31 – 59.9; 8.75) |  | 50.83 (46.06 – 59.66; 7.2) |  | 47.39 (42.25 – 57.11; 8.84) |  | 50.83 (45.47 – 60.25; 8.81) |  | 36.78 (36.78 – 36.78; 0) |  | 49.85 (44.74 – 59.92; 8.02) |  |
| % time HbDiffx > 0.2 | Frontal | Left | 22.7 (16.6 – 31.68; 7.1) | **0.0402** | 23.29 (20.31 – 25.1; 2.91) | 0.7508 | 19.85 (16.16 – 27.52; 4.8) | **0.0275** | 24.29 (21.75 – 32.99; 6.15) | 0.2962 | 22.93 (22.93 – 22.93; 0) | 1 | 22.87 (16.6 – 30.22; 6.52) | **0.0157** |
|  |  | Right | 28.91 (25.29 – 33.55; 4.51) |  | 22.94 (19.6 – 28.82; 5.05) |  | 28.54 (21.22 – 30.69; 6.24) |  | 28.72 (24.07 – 32.98; 4.89) |  | 16.94 (16.94 – 16.94; 0) |  | 28.62 (22.73 – 32.47; 5.25) |  |
|  | Parietal | Left | 25.1 (16.8 – 30.77; 6.95) | 0.6035 | 24.49 (20.05 – 25.55; 2.29) | 0.7075 | 24.79 (15.18 – 27.92; 6.93) | 0.7185 | 24.66 (21.37 – 28.59; 4.1) | 0.5974 | 18.18 (18.18 – 18.18; 0) | 1 | 24.82 (17.85 – 28.89; 6.45) | 0.4752 |
|  |  | Right | 24.56 (18.25 – 32.42; 7.16) |  | 25.08 (16.85 – 30.26; 6.29) |  | 23.07 (16.46 – 28.72; 6.99) |  | 27.89 (19.89 – 31.21; 7.67) |  | 8.88 (8.88 – 8.88; 0) |  | 24.6 (17.87 – 31.25; 6.73) |  |
|  | Temporal | Left | 24.03 (17.5 – 29.07; 6.3) | 0.4962 | 22.27 (18.16 – 25.89; 4.25) | 0.931 | 22.04 (17.58 – 27.05; 4.51) | 0.1215 | 25.31 (17.87 – 32.09; 7.71) | 0.5974 | 16.74 (16.74 – 16.74; 0) | 1 | 23.95 (17.73 – 28.9; 5.26) | 0.6391 |
|  |  | Right | 25.81 (17.29 – 31.8; 6.16) |  | 23.11 (17.64 – 29.5; 6.2) |  | 26.82 (19.54 – 31.85; 6.09) |  | 23.5 (15.75 – 29.83; 7.53) |  | 40.08 (40.08 – 40.08; 0) |  | 24.52 (16.51 – 31.74; 7.3) |  |
|  | Occipital | Left | 20.9 (14.59 – 26.59; 6.05) | **0.0311** | 19.28 (11.92 – 26.98; 7.5) | 0.1939 | 22.54 (14.47 – 26.74; 6.44) | 0.1296 | 19.47 (14.06 – 22.55; 5.19) | **0.05** | 3.51 (3.51 – 3.51; 0) | 1 | 20.4 (14.54 – 26.72; 6.12) | **0.0095** |
|  |  | Right | 25.71 (18.13 – 37.27; 7.82) |  | 23.47 (17.21 – 31.3; 6.83) |  | 23.47 (18.38 – 32.35; 5.48) |  | 26.78 (17.75 – 36.1; 9.32) |  | 9.09 (9.09 – 9.09; 0) |  | 25 (18 – 35.33; 7.19) |  |
| % time HbDiffx > 0.3 | Frontal | Left | 11.97 (8.45 – 21.07; 5.01) | **0.0397** | 14.88 (10.23 – 16.69; 3) | 0.3708 | 11.39 (8.6 – 17.27; 3.97) | **0.0338** | 16.21 (10.15 – 19.33; 5.71) | 0.2359 | 11.78 (11.78 – 11.78; 0) | 1 | 14.05 (8.68 – 18.27; 5.37) | **0.0111** |
|  |  | Right | 19.15 (14.62 – 23.59; 4.64) |  | 14.89 (12.6 – 17.91; 2.91) |  | 17.56 (13.38 – 20.41; 3.91) |  | 18.57 (14.71 – 22.73; 4.15) |  | 5.99 (5.99 – 5.99; 0) |  | 17.96 (14.36 – 21.71; 3.75) |  |
|  | Parietal | Left | 16.53 (10.88 – 18.99; 5.03) | 0.9751 | 15.52 (10.04 – 17.84; 2.98) | 0.8399 | 15.61 (8.02 – 18.62; 5.87) | 0.7307 | 16.91 (12.83 – 18.81; 2.32) | 0.8235 | 7.44 (7.44 – 7.44; 0) | 1 | 16.4 (10.83 – 18.92; 5.04) | 0.9745 |
|  |  | Right | 14.97 (10.15 – 20.75; 5.19) |  | 13.96 (8.91 – 18.28; 5.56) |  | 11.87 (7.21 – 19.95; 5.66) |  | 16.38 (12.78 – 22.63; 5.25) |  | 3.51 (3.51 – 3.51; 0) |  | 15.2 (10.14 – 20.91; 5.29) |  |
|  | Temporal | Left | 14.69 (8.65 – 18.52; 4.87) | 0.4864 | 11.12 (7.65 – 15.15; 4.24) | 0.729 | 11.58 (8.76 – 16.52; 3.93) | **0.0422** | 15.69 (7.95 – 21.99; 7.67) | 0.3599 | 7.23 (7.23 – 7.23; 0) | 1 | 12.99 (8.6 – 18.2; 4.83) | 0.5649 |
|  |  | Right | 16.39 (10 – 21.13; 5.59) |  | 12.43 (6.45 – 17.81; 6.09) |  | 16.46 (10.71 – 21.33; 5.19) |  | 13.36 (6.67 – 17.72; 5.99) |  | 21.49 (21.49 – 21.49; 0) |  | 15.96 (9.25 – 19.25; 5.94) |  |
|  | Occipital | Left | 10.76 (5.98 – 14.55; 4.53) | **0.0082** | 8.42 (3.84 – 13.26; 4.81) | **0.0141** | 12.18 (6.6 – 14.85; 3.86) | **0.0397** | 6.8 (4.65 – 12.24; 3.33) | **0.0032** | 0 (0 – 0; 0) | 1 | 9.83 (5.7 – 14.56; 4.66) | **<0.001** |
|  |  | Right | 14.77 (9.77 – 25.13; 6.83) |  | 17.77 (11.31 – 22.79; 6.42) |  | 14.98 (10.56 – 23.21; 5.45) |  | 17.39 (8.51 – 25.23; 8.05) |  | 5.58 (5.58 – 5.58; 0) |  | 16.49 (10 – 24.71; 6.86) |  |
| The p-values in the table are derived using Mann-Whitney U test between the subgrouped percent time results. *COx-a, cerebral oximetry index with arterial blood pressure; CVR, cerebrovascular reactivity index; HbDiffx, hemoglobin difference index; HbOx, oxyhemoglobin index; HHbx, deoxyhemoglobin index; IQR, interquartile range; MAD, median absolute deviation; rSO_2_, regional cerebral oxygen saturation; tHbx, total hemoglobin index.* | | | | | | | | | | | | | | |

Appendix S6f: Subgrouped Percent Time Results of rSO_2_ Using Raw Data

| **Physiologic Variable** | **Brain Lobe** | **Hemisphere** | **Subgroups** | | | | | | | | | | | |
| --- | --- | --- | --- | --- | --- | --- | --- | --- | --- | --- | --- | --- | --- | --- |
|  |  |  | **Age < 40 [n=38]** | | **Age 40 – 60 [n=12]** | | **Males [n=28]** | | **Females [n=22]** | | **Left Hand Dominance [n=1]** | | **Right Hand Dominance [n=49]** | |
|  |  |  | **Median (IQR; MAD)** | **p-value** | **Median (IQR; MAD)** | **p-value** | **Median (IQR; MAD)** | **p-value** | **Median (IQR; MAD)** | **p-value** | **Median (IQR; MAD)** | **p-value** | **Median (IQR; MAD)** | **p-value** |
| **1 Hz Sampled Data** | | | | | | | | | | | | | | |
| % time rSO_2_ > 30% | Frontal | Left | 100 (100 – 100; 0) | **0.0191** | 100 (100 – 100; 0) | 1 | 100 (100 – 100; 0) | 0.3042 | 100 (100 – 100; 0) | **0.0457** | 100 (100 – 100; 0) | 1 | 100 (100 – 100; 0) | **0.0287** |
|  |  | Right | 100 (100 – 100; 0) |  | 100 (100 – 100; 0) |  | 100 (100 – 100; 0) |  | 100 (99.97 – 100; 0) |  | 100 (100 – 100; 0) |  | 100 (100 – 100; 0) |  |
|  | Parietal | Left | 100 (100 – 100; 0) | 0.1564 | 100 (99.92 – 100; 0) | 0.6301 | 100 (100 – 100; 0) | 0.8966 | 100 (99.95 – 100; 0) | **0.0362** | 100 (100 – 100; 0) | 1 | 100 (99.98 – 100; 0) | 0.1298 |
|  |  | Right | 100 (100 – 100; 0) |  | 100 (99.92 – 100; 0) |  | 100 (100 – 100; 0) |  | 100 (100 – 100; 0) |  | 100 (100 – 100; 0) |  | 100 (100 – 100; 0) |  |
|  | Temporal | Left | 100 (100 – 100; 0) | 0.313 | 100 (99.98 – 100; 0) | 0.7192 | 100 (100 – 100; 0) | **0.0221** | 100 (100 – 100; 0) | 0.3925 | 99.98 (99.98 – 99.98; 0) | 1 | 100 (100 – 100; 0) | 0.3919 |
|  |  | Right | 100 (100 – 100; 0) |  | 100 (100 – 100; 0) |  | 100 (100 – 100; 0) |  | 100 (100 – 100; 0) |  | 100 (100 – 100; 0) |  | 100 (100 – 100; 0) |  |
|  | Occipital | Left | 99.96 (99.9 – 100; 0.04) | **<0.001** | 99.94 (99.85 – 99.97; 0.06) | **<0.001** | 99.95 (99.89 – 100; 0.05) | **<0.001** | 99.96 (99.87 – 100; 0.04) | **0.0141** | 99.89 (99.89 – 99.89; 0) | 1 | 99.96 (99.86 – 100; 0.04) | **<0.001** |
|  |  | Right | 100 (100 – 100; 0) |  | 100 (100 – 100; 0) |  | 100 (100 – 100; 0) |  | 100 (100 – 100; 0) |  | 100 (100 – 100; 0) |  | 100 (100 – 100; 0) |  |
| % time rSO_2_ > 40% | Frontal | Left | 100 (100 – 100; 0) | **<0.001** | 100 (99.82 – 100; 0) | **0.0013** | 100 (100 – 100; 0) | **<0.001** | 100 (99.89 – 100; 0) | **<0.001** | 100 (100 – 100; 0) | 1 | 100 (100 – 100; 0) | **<0.001** |
|  |  | Right | 70.5 (3.12 – 98.66; 29.5) |  | 20.92 (0.64 – 98.22; 20.92) |  | 80.06 (4.14 – 98.82; 19.94) |  | 28.36 (2.27 – 95.7; 28.34) |  | 41.36 (41.36 – 41.36; 0) |  | 65.7 (2.26 – 98.72; 34.3) |  |
|  | Parietal | Left | 100 (67.63 – 100; 0) | 0.9025 | 99.99 (75.69 – 100; 0.01) | 0.4063 | 100 (83.92 – 100; 0) | 0.5308 | 99.99 (73.47 – 100; 0.01) | 0.7443 | 100 (100 – 100; 0) | 1 | 100 (73.41 – 100; 0) | 0.9014 |
|  |  | Right | 100 (92.54 – 100; 0) |  | 90.78 (82.64 – 99.86; 9.12) |  | 99.92 (87.18 – 100; 0.08) |  | 99.91 (86.03 – 100; 0.09) |  | 81.27 (81.27 – 81.27; 0) |  | 100 (89.15 – 100; 0) |  |
|  | Temporal | Left | 57.33 (17.15 – 97.78; 41.02) | **<0.001** | 60.27 (3.19 – 87.96; 34.38) | **0.0135** | 37.89 (2.82 – 81.63; 37.62) | **<0.001** | 68.97 (34.81 – 97.78; 30.22) | 0.0594 | 30.31 (30.31 – 30.31; 0) | 1 | 58.72 (4.84 – 94.8; 39.9) | **<0.001** |
|  |  | Right | 100 (99.41 – 100; 0) |  | 100 (83.86 – 100; 0) |  | 100 (100 – 100; 0) |  | 99.95 (19.58 – 100; 0.05) |  | 100 (100 – 100; 0) |  | 100 (99.23 – 100; 0) |  |
|  | Occipital | Left | 0.34 (0.02 – 2.02; 0.34) | **<0.001** | 0.53 (0.17 – 3.76; 0.46) | **<0.001** | 0.19 (0.01 – 0.94; 0.19) | **<0.001** | 0.72 (0.13 – 5.03; 0.72) | **<0.001** | 2.21 (2.21 – 2.21; 0) | 1 | 0.43 (0.02 – 2.17; 0.43) | **<0.001** |
|  |  | Right | 100 (93.51 – 100; 0) |  | 98.8 (91.39 – 100; 1.2) |  | 100 (99.93 – 100; 0) |  | 94.98 (67.38 – 100; 5.02) |  | 100 (100 – 100; 0) |  | 100 (93.07 – 100; 0) |  |
| % time rSO_2_ > 50% | Frontal | Left | 0.01 (0 – 87.41; 0.01) | **0.0248** | 0 (0 – 4.58; 0) | **0.0367** | 0 (0 – 54.06; 0) | **0.0161** | 0.01 (0 – 85.45; 0.01) | 0.1727 | 0 (0 – 0; 0) | 1 | 0 (0 – 79.07; 0) | **0.0057** |
|  |  | Right | 0 (0 – 0.13; 0) |  | 0 (0 – 0; 0) |  | 0 (0 – 0; 0) |  | 0 (0 – 0.29; 0) |  | 0 (0 – 0; 0) |  | 0 (0 – 0.02; 0) |  |
|  | Parietal | Left | 42.46 (2.61 – 89.47; 42.11) | **<0.001** | 16.2 (9.65 – 44.18; 16.1) | **0.0429** | 60.48 (7.43 – 88.13; 35) | **<0.001** | 16.2 (3.15 – 49.87; 16.2) | 0.3353 | 86.69 (86.69 – 86.69; 0) | 1 | 37.1 (3.27 – 87.86; 35.9) | **<0.001** |
|  |  | Right | 0.32 (0 – 24.44; 0.32) |  | 5.84 (0.01 – 15.48; 5.84) |  | 0.01 (0 – 2.06; 0.01) |  | 15.47 (0.91 – 28.21; 13.17) |  | 0.25 (0.25 – 0.25; 0) |  | 0.73 (0 – 20.38; 0.73) |  |
|  | Temporal | Left | 0 (0 – 0.01; 0) | **<0.001** | 0 (0 – 0.23; 0) | 0.2875 | 0 (0 – 0; 0) | **<0.001** | 0 (0 – 1.57; 0) | 0.054 | 0.02 (0.02 – 0.02; 0) | 1 | 0 (0 – 0.02; 0) | **<0.001** |
|  |  | Right | 15.45 (0 – 48.39; 15.45) |  | 0.06 (0 – 6.44; 0.06) |  | 15.45 (0 – 46.96; 15.45) |  | 1.25 (0 – 33.15; 1.25) |  | 8.71 (8.71 – 8.71; 0) |  | 2.12 (0 – 44.99; 2.12) |  |
|  | Occipital | Left | 0 (0 – 0; 0) | **<0.001** | 0 (0 – 0; 0) | **0.0011** | 0 (0 – 0; 0) | **<0.001** | 0 (0 – 0; 0) | **<0.001** | 0 (0 – 0; 0) | 1 | 0 (0 – 0; 0) | **<0.001** |
|  |  | Right | 2.81 (0 – 25.35; 2.81) |  | 0.47 (0 – 10.94; 0.47) |  | 1.03 (0 – 14.53; 1.03) |  | 7.2 (0.2 – 30.26; 7.2) |  | 0.55 (0.55 – 0.55; 0) |  | 2.17 (0 – 23.33; 2.17) |  |
| % time rSO_2_ > 60% | Frontal | Left | 0 (0 – 0; 0) | 0.8184 | 0 (0 – 0; 0) | 1 | 0 (0 – 0; 0) | 0.9463 | 0 (0 – 0; 0) | 0.6547 | 0 (0 – 0; 0) | 1 | 0 (0 – 0; 0) | 0.8095 |
|  |  | Right | 0 (0 – 0; 0) |  | 0 (0 – 0; 0) |  | 0 (0 – 0; 0) |  | 0 (0 – 0; 0) |  | 0 (0 – 0; 0) |  | 0 (0 – 0; 0) |  |
|  | Parietal | Left | 0 (0 – 0.13; 0) | 0.0023 | 0 (0 – 0.02; 0) | 0.3281 | 0 (0 – 0.23; 0) | **0.0224** | 0 (0 – 0.02; 0) | **0.0255** | 0.23 (0.23 – 0.23; 0) | 1 | 0 (0 – 0.09; 0) | **0.0021** |
|  |  | Right | 0 (0 – 0; 0) |  | 0 (0 – 0; 0) |  | 0 (0 – 0; 0) |  | 0 (0 – 0; 0) |  | 0 (0 – 0; 0) |  | 0 (0 – 0; 0) |  |
|  | Temporal | Left | 0 (0 – 0; 0) | **0.0457** | 0 (0 – 0; 0) | 1 | 0 (0 – 0; 0) | 0.3042 | 0 (0 – 0; 0) | 0.1536 | 0 (0 – 0; 0) | 1 | 0 (0 – 0; 0) | 0.0739 |
|  |  | Right | 0 (0 – 0; 0) |  | 0 (0 – 0; 0) |  | 0 (0 – 0; 0) |  | 0 (0 – 0; 0) |  | 0 (0 – 0; 0) |  | 0 (0 – 0; 0) |  |
|  | Occipital | Left | 0 (0 – 0; 0) | **0.0117** | 0 (0 – 0; 0) | 0.0788 | 0 (0 – 0; 0) | 0.1611 | 0 (0 – 0; 0) | **0.0048** | 0 (0 – 0; 0) | 1 | 0 (0 – 0; 0) | **0.0018** |
|  |  | Right | 0 (0 – 0; 0) |  | 0 (0 – 0; 0) |  | 0 (0 – 0; 0) |  | 0 (0 – 0.03; 0) |  | 0 (0 – 0; 0) |  | 0 (0 – 0; 0) |  |
| **250 Hz Sampled Data** | | | | | | | | | | | | | | |
| % time rSO_2_ > 30% | Frontal | Left | 100 (100 – 100; 0) | **0.0145** | 100 (100 – 100; 0) | 0.8389 | 100 (100 – 100; 0) | 0.3288 | 100 (100 – 100; 0) | **0.0386** | 100 (100 – 100; 0) | 1 | 100 (100 – 100; 0) | 0.0523 |
|  |  | Right | 100 (99.74 – 100; 0) |  | 100 (100 – 100; 0) |  | 100 (99.97 – 100; 0) |  | 99.98 (99.64 – 100; 0.02) |  | 89.36 (89.36 – 89.36; 0) |  | 100 (99.88 – 100; 0) |  |
|  | Parietal | Left | 99.98 (96.06 – 100; 0.02) | 0.0915 | 99.51 (91.01 – 100; 0.49) | 0.6531 | 100 (93.68 – 100; 0) | 0.5309 | 99.89 (93.28 – 100; 0.11) | 0.0611 | 100 (100 – 100; 0) | 1 | 99.96 (92.18 – 100; 0.04) | 0.0993 |
|  |  | Right | 100 (99.76 – 100; 0) |  | 99.97 (96.25 – 100; 0.03) |  | 100 (98.71 – 100; 0) |  | 100 (99.77 – 100; 0) |  | 100 (100 – 100; 0) |  | 100 (99 – 100; 0) |  |
|  | Temporal | Left | 99.96 (96.03 – 100; 0.04) | 0.3958 | 99.65 (90.13 – 100; 0.35) | 0.5927 | 99.93 (92.26 – 100; 0.07) | **0.0202** | 100 (95.64 – 100; 0) | 0.3247 | 82.65 (82.65 – 82.65; 0) | 1 | 99.96 (94.42 – 100; 0.04) | 0.3902 |
|  |  | Right | 100 (98.83 – 100; 0) |  | 100 (95.66 – 100; 0) |  | 100 (99.99 – 100; 0) |  | 99.68 (79.64 – 100; 0.32) |  | 100 (100 – 100; 0) |  | 100 (98.64 – 100; 0) |  |
|  | Occipital | Left | 75.45 (74.19 – 77.08; 1.54) | **<0.001** | 74.28 (73.87 – 76.47; 1.13) | **<0.001** | 75.37 (73.23 – 78.25; 2.41) | **<0.001** | 74.93 (74.16 – 76.51; 1.52) | **<0.001** | 75.79 (75.79 – 75.79; 0) | 1 | 75.22 (73.89 – 76.8; 1.41) | **<0.001** |
|  |  | Right | 99.99 (98.16 – 100; 0.01) |  | 99.61 (95.07 – 100; 0.39) |  | 100 (99.77 – 100; 0) |  | 98.57 (89.59 – 100; 1.43) |  | 99.97 (99.97 – 99.97; 0) |  | 100 (97.17 – 100; 0) |  |
| % time rSO_2_ > 40% | Frontal | Left | 100 (99.56 – 100; 0) | **<0.001** | 99.99 (99.85 – 100; 0.01) | **<0.001** | 100 (99.93 – 100; 0) | **<0.001** | 100 (99.7 – 100; 0) | **<0.001** | 100 (100 – 100; 0) | 1 | 100 (99.78 – 100; 0) | **<0.001** |
|  |  | Right | 70.37 (6.72 – 98.12; 29.49) |  | 21.28 (0.99 – 97.27; 21.27) |  | 79.91 (4.1 – 98.37; 20.03) |  | 33.29 (2.37 – 93.22; 32.95) |  | 54.65 (54.65 – 54.65; 0) |  | 65.94 (2.35 – 98.36; 34.05) |  |
|  | Parietal | Left | 96.88 (64.05 – 99.92; 3.12) | 0.2634 | 94.46 (73.01 – 99.81; 5.54) | 0.7728 | 98.82 (75.91 – 99.94; 1.18) | 0.9542 | 93.57 (72.13 – 98.55; 6.43) | 0.1694 | 95.62 (95.62 – 95.62; 0) | 1 | 96.1 (71.69 – 99.93; 3.9) | 0.3298 |
|  |  | Right | 99.4 (88.4 – 99.99; 0.6) |  | 89.22 (68.24 – 99.84; 10.78) |  | 97.62 (85.66 – 99.98; 2.38) |  | 99.22 (85.12 – 99.99; 0.78) |  | 81.14 (81.14 – 81.14; 0) |  | 98.81 (87.17 – 99.99; 1.19) |  |
|  | Temporal | Left | 53.42 (32.18 – 89.4; 25.54) | **<0.001** | 56.76 (36.09 – 83.77; 22.37) | **0.0114** | 46.65 (29.12 – 80.39; 25.75) | **<0.001** | 65.05 (45.45 – 93.78; 27.54) | 0.1124 | 47.48 (47.48 – 47.48; 0) | 1 | 53.91 (34.42 – 91.31; 25.71) | **<0.001** |
|  |  | Right | 99.94 (92.79 – 100; 0.06) |  | 100 (84.96 – 100; 0) |  | 100 (96.95 – 100; 0) |  | 95.54 (41.54 – 100; 4.46) |  | 99.95 (99.95 – 99.95; 0) |  | 99.97 (92.62 – 100; 0.03) |  |
|  | Occipital | Left | 29.21 (23.89 – 33.06; 4.87) | **<0.001** | 28.56 (21.43 – 36.12; 7.52) | **<0.001** | 28.83 (23.2 – 31.87; 3.97) | **<0.001** | 30.31 (23.05 – 37.15; 7.03) | **<0.001** | 32.05 (32.05 – 32.05; 0) | 1 | 29.01 (21.66 – 33.95; 6.6) | **<0.001** |
|  |  | Right | 98.67 (88.88 – 100; 1.33) |  | 96.15 (74.19 – 100; 3.85) |  | 99.91 (97.03 – 100; 0.09) |  | 91.6 (66.99 – 99.06; 8.4) |  | 97.25 (97.25 – 97.25; 0) |  | 98.59 (80.85 – 100; 1.41) |  |
| % time rSO_2_ > 50% | Frontal | Left | 2.87 (0 – 75.52; 2.87) | **0.0237** | 0 (0 – 3.86; 0) | 0.0578 | 0 (0 – 51.68; 0) | 0.059 | 0.74 (0 – 85.91; 0.74) | 0.1027 | 0 (0 – 0; 0) | 1 | 0.43 (0 – 72.62; 0.43) | **0.0047** |
|  |  | Right | 0.02 (0 – 2.47; 0.02) |  | 0 (0 – 0; 0) |  | 0 (0 – 0.04; 0) |  | 0 (0 – 2.47; 0) |  | 2.76 (2.76 – 2.76; 0) |  | 0 (0 – 0.23; 0) |  |
|  | Parietal | Left | 45.73 (13.2 – 72.53; 27.66) | **<0.001** | 27.7 (13.04 – 42.71; 15.97) | **0.0404** | 54.03 (16.3 – 71.59; 26.47) | **<0.001** | 27.7 (12.95 – 51.99; 20.48) | 0.2312 | 66.14 (66.14 – 66.14; 0) | 1 | 36.54 (12.48 – 70.01; 29.73) | **<0.001** |
|  |  | Right | 6.85 (0.89 – 26.72; 6.84) |  | 8.87 (1.19 – 23.74; 8.55) |  | 3.62 (0.19 – 10.95; 3.57) |  | 21.14 (3.52 – 28.46; 14.32) |  | 0.81 (0.81 – 0.81; 0) |  | 6.98 (1.12 – 24.26; 6.98) |  |
|  | Temporal | Left | 0.27 (0 – 11.76; 0.27) | **<0.001** | 3.59 (0 – 12.95; 3.59) | 0.3967 | 0.02 (0 – 9.14; 0.02) | **0.001** | 6.67 (0 – 17.14; 6.67) | 0.0713 | 15.46 (15.46 – 15.46; 0) | 1 | 0.09 (0 – 12; 0.09) | **<0.001** |
|  |  | Right | 19.84 (2.04 – 42.42; 18.88) |  | 10.44 (0 – 14.93; 9.91) |  | 19.44 (0.67 – 42.33; 19.42) |  | 15.35 (4.69 – 34.18; 14.11) |  | 10.37 (10.37 – 10.37; 0) |  | 16.56 (1.06 – 42.29; 16.55) |  |
|  | Occipital | Left | 7.03 (2.66 – 10.55; 4.33) | 0.0934 | 7.84 (3.43 – 10.48; 4.55) | 0.7506 | 7.03 (2.2 – 9.04; 3.5) | 0.5607 | 8.41 (2.87 – 14.43; 5.64) | **0.0024** | 10.49 (10.49 – 10.49; 0) | 1 | 7.22 (2.74 – 10.56; 4.43) | 0.0978 |
|  |  | Right | 15.52 (1.5 – 26.12; 13.1) |  | 13.11 (0.06 – 32.9; 13.11) |  | 4.46 (0.03 – 18.58; 4.46) |  | 21.72 (9.63 – 34.3; 13.21) |  | 7 (7 – 7; 0) |  | 16.61 (0.28 – 28.62; 16.01) |  |
| % time rSO_2_ > 60% | Frontal | Left | 0 (0 – 0; 0) | 0.3462 | 0 (0 – 0; 0) | 0.1662 | 0 (0 – 0.01; 0) | 0.4747 | 0 (0 – 0; 0) | 0.1525 | 0 (0 – 0; 0) | 1 | 0 (0 – 0; 0) | 0.621 |
|  |  | Right | 0 (0 – 0.13; 0) |  | 0 (0 – 0; 0) |  | 0 (0 – 0; 0) |  | 0 (0 – 0.13; 0) |  | 0 (0 – 0; 0) |  | 0 (0 – 0.03; 0) |  |
|  | Parietal | Left | 1.09 (0.07 – 10.97; 1.09) | **0.0021** | 2.31 (0 – 8.56; 2.31) | 0.1885 | 1.35 (0.05 – 10.63; 1.35) | **0.0066** | 1.6 (0.03 – 8.28; 1.59) | 0.0482 | 20.79 (20.79 – 20.79; 0) | 1 | 1.21 (0.01 – 9.87; 1.21) | **0.0011** |
|  |  | Right | 0.02 (0 – 0.97; 0.02) |  | 0.04 (0 – 0.24; 0.04) |  | 0 (0 – 0.48; 0) |  | 0.07 (0 – 1.86; 0.07) |  | 0 (0 – 0; 0) |  | 0.03 (0 – 0.65; 0.03) |  |
|  | Temporal | Left | 0 (0 – 0.85; 0) | 0.1248 | 0.42 (0 – 1.5; 0.42) | 0.7813 | 0 (0 – 0.82; 0) | 0.9648 | 0.34 (0 – 1.46; 0.34) | 0.0756 | 0.82 (0.82 – 0.82; 0) | 1 | 0 (0 – 1.06; 0) | 0.1804 |
|  |  | Right | 0.07 (0 – 3.25; 0.07) |  | 0.02 (0 – 0.52; 0.02) |  | 0 (0 – 0.37; 0) |  | 1.35 (0.01 – 3.28; 1.35) |  | 0.01 (0.01 – 0.01; 0) |  | 0.06 (0 – 2.91; 0.06) |  |
|  | Occipital | Left | 0.05 (0 – 0.78; 0.05) | 0.684 | 0.2 (0 – 0.75; 0.2) | 0.2765 | 0.03 (0 – 0.25; 0.03) | 0.6365 | 0.32 (0 – 2.06; 0.32) | 0.0429 | 0.69 (0.69 – 0.69; 0) | 1 | 0.05 (0 – 0.81; 0.05) | 0.3806 |
|  |  | Right | 0.19 (0 – 2.83; 0.19) |  | 1.16 (0 – 4.1; 1.16) |  | 0 (0 – 0.96; 0) |  | 2.61 (0.08 – 7.28; 2.6) |  | 0.9 (0.9 – 0.9; 0) |  | 0.26 (0 – 3.11; 0.26) |  |
| The p-values in the table are derived using Mann-Whitney U test between the subgrouped percent time results. *IQR, interquartile range; MAD, median absolute deviation; rSO_2_, regional cerebral oxygen saturation.* | | | | | | | | | | | | | | |

Appendix S6g: Subgrouped Regional Hemispheric Disparity Analysis on CVR Indices Using 10-Second Decimated Data

| **Brain Lobe** | **Physiologic Variable** | **Median (IQR)** | | | | | |
| --- | --- | --- | --- | --- | --- | --- | --- |
|  |  | **Age < 40 [n=38]** | **Age 40 – 60 [n=12]** | **Males [n=28]** | **Females [n=22]** | **Left Hand Dominance [n=1]** | **Right Hand Dominance [n=49]** |
| **1 Hz Sampled Data** | | | | | | | |
| Frontal | ARHD of COx-a (au) | 0.23 (0.11 – 0.42) | 0.23 (0.1 – 0.39) | 0.22 (0.1 – 0.38) | 0.25 (0.11 – 0.44) | 0.19 (0.09 – 0.35) | 0.24 (0.11 – 0.41) |
|  | ARHD of HbOx (au) | 0.22 (0.1 – 0.41) | 0.23 (0.1 – 0.41) | 0.21 (0.09 – 0.4) | 0.22 (0.1 – 0.43) | 0.18 (0.08 – 0.35) | 0.22 (0.1 – 0.42) |
|  | ARHD of HHbx (au) | 0.21 (0.1 – 0.41) | 0.23 (0.09 – 0.43) | 0.21 (0.09 – 0.42) | 0.24 (0.1 – 0.42) | 0.15 (0.08 – 0.29) | 0.22 (0.09 – 0.42) |
|  | ARHD of tHbx (au) | 0.23 (0.11 – 0.43) | 0.24 (0.1 – 0.42) | 0.21 (0.1 – 0.44) | 0.23 (0.11 – 0.42) | 0.17 (0.07 – 0.36) | 0.23 (0.1 – 0.43) |
|  | ARHD of HbDiffx (au) | 0.22 (0.11 – 0.4) | 0.23 (0.1 – 0.4) | 0.22 (0.11 – 0.38) | 0.24 (0.11 – 0.45) | 0.23 (0.1 – 0.38) | 0.23 (0.11 – 0.41) |
|  | MAD of ARHD COx-a (au) | 0.14 (0.12 – 0.17) | 0.13 (0.12 – 0.15) | 0.14 (0.11 – 0.15) | 0.14 (0.12 – 0.18) | 0.13 (0.13 – 0.13) | 0.14 (0.12 – 0.17) |
|  | MAD of ARHD HbOx (au) | 0.14 (0.12 – 0.18) | 0.15 (0.12 – 0.15) | 0.14 (0.12 – 0.17) | 0.15 (0.13 – 0.17) | 0.11 (0.11 – 0.11) | 0.14 (0.12 – 0.17) |
|  | MAD of ARHD HHbx (au) | 0.14 (0.11 – 0.18) | 0.16 (0.14 – 0.17) | 0.14 (0.11 – 0.17) | 0.16 (0.13 – 0.18) | 0.1 (0.1 – 0.1) | 0.15 (0.12 – 0.18) |
|  | MAD of ARHD tHbx (au) | 0.14 (0.12 – 0.16) | 0.15 (0.12 – 0.16) | 0.14 (0.12 – 0.16) | 0.14 (0.13 – 0.17) | 0.12 (0.12 – 0.12) | 0.14 (0.12 – 0.16) |
|  | MAD of ARHD HbDiffx (au) | 0.13 (0.12 – 0.15) | 0.14 (0.12 – 0.16) | 0.13 (0.12 – 0.15) | 0.15 (0.13 – 0.18) | 0.14 (0.14 – 0.14) | 0.13 (0.12 – 0.16) |
| Parietal | ARHD of COx-a (au) | 0.22 (0.1 – 0.43) | 0.26 (0.1 – 0.46) | 0.22 (0.1 – 0.43) | 0.23 (0.1 – 0.44) | 0.15 (0.05 – 0.35) | 0.23 (0.1 – 0.45) |
|  | ARHD of HbOx (au) | 0.2 (0.09 – 0.43) | 0.22 (0.11 – 0.44) | 0.21 (0.09 – 0.41) | 0.22 (0.1 – 0.44) | 0.2 (0.1 – 0.33) | 0.21 (0.09 – 0.43) |
|  | ARHD of HHbx (au) | 0.23 (0.1 – 0.44) | 0.24 (0.1 – 0.5) | 0.23 (0.1 – 0.43) | 0.24 (0.09 – 0.46) | 0.19 (0.08 – 0.33) | 0.24 (0.1 – 0.44) |
|  | ARHD of tHbx (au) | 0.21 (0.09 – 0.43) | 0.25 (0.11 – 0.45) | 0.21 (0.09 – 0.42) | 0.25 (0.1 – 0.47) | 0.17 (0.09 – 0.34) | 0.22 (0.1 – 0.44) |
|  | ARHD of HbDiffx (au) | 0.21 (0.1 – 0.42) | 0.24 (0.1 – 0.45) | 0.22 (0.09 – 0.43) | 0.22 (0.1 – 0.43) | 0.16 (0.06 – 0.34) | 0.22 (0.1 – 0.43) |
|  | MAD of ARHD COx-a (au) | 0.15 (0.13 – 0.16) | 0.16 (0.12 – 0.17) | 0.15 (0.13 – 0.17) | 0.15 (0.12 – 0.17) | 0.12 (0.12 – 0.12) | 0.15 (0.13 – 0.17) |
|  | MAD of ARHD HbOx (au) | 0.14 (0.12 – 0.17) | 0.14 (0.13 – 0.15) | 0.13 (0.12 – 0.15) | 0.15 (0.13 – 0.18) | 0.12 (0.12 – 0.12) | 0.14 (0.13 – 0.17) |
|  | MAD of ARHD HHbx (au) | 0.15 (0.13 – 0.17) | 0.17 (0.13 – 0.18) | 0.14 (0.13 – 0.16) | 0.16 (0.14 – 0.18) | 0.12 (0.12 – 0.12) | 0.15 (0.13 – 0.17) |
|  | MAD of ARHD tHbx (au) | 0.15 (0.12 – 0.17) | 0.16 (0.13 – 0.17) | 0.15 (0.11 – 0.17) | 0.17 (0.14 – 0.18) | 0.11 (0.11 – 0.11) | 0.15 (0.13 – 0.17) |
|  | MAD of ARHD HbDiffx (au) | 0.14 (0.12 – 0.16) | 0.15 (0.13 – 0.18) | 0.15 (0.13 – 0.16) | 0.14 (0.12 – 0.17) | 0.12 (0.12 – 0.12) | 0.14 (0.12 – 0.17) |
| Temporal | ARHD of COx-a (au) | 0.22 (0.1 – 0.39) | 0.21 (0.1 – 0.42) | 0.21 (0.1 – 0.41) | 0.22 (0.1 – 0.4) | 0.18 (0.08 – 0.34) | 0.22 (0.1 – 0.4) |
|  | ARHD of HbOx (au) | 0.23 (0.1 – 0.44) | 0.21 (0.09 – 0.4) | 0.21 (0.09 – 0.41) | 0.23 (0.11 – 0.46) | 0.21 (0.1 – 0.4) | 0.23 (0.1 – 0.43) |
|  | ARHD of HHbx (au) | 0.21 (0.09 – 0.43) | 0.25 (0.11 – 0.45) | 0.21 (0.1 – 0.45) | 0.21 (0.1 – 0.44) | 0.21 (0.08 – 0.32) | 0.21 (0.1 – 0.45) |
|  | ARHD of tHbx (au) | 0.24 (0.1 – 0.44) | 0.22 (0.1 – 0.42) | 0.22 (0.09 – 0.41) | 0.25 (0.1 – 0.45) | 0.21 (0.1 – 0.4) | 0.24 (0.1 – 0.43) |
|  | ARHD of HbDiffx (au) | 0.23 (0.1 – 0.4) | 0.2 (0.09 – 0.41) | 0.21 (0.1 – 0.41) | 0.23 (0.1 – 0.4) | 0.2 (0.1 – 0.36) | 0.22 (0.1 – 0.41) |
|  | MAD of ARHD COx-a (au) | 0.13 (0.12 – 0.15) | 0.14 (0.13 – 0.15) | 0.14 (0.12 – 0.15) | 0.13 (0.12 – 0.16) | 0.12 (0.12 – 0.12) | 0.14 (0.12 – 0.16) |
|  | MAD of ARHD HbOx (au) | 0.15 (0.12 – 0.17) | 0.14 (0.12 – 0.15) | 0.14 (0.12 – 0.16) | 0.15 (0.13 – 0.17) | 0.13 (0.13 – 0.13) | 0.15 (0.12 – 0.17) |
|  | MAD of ARHD HHbx (au) | 0.14 (0.11 – 0.17) | 0.14 (0.13 – 0.18) | 0.15 (0.12 – 0.16) | 0.14 (0.12 – 0.18) | 0.12 (0.12 – 0.12) | 0.14 (0.11 – 0.17) |
|  | MAD of ARHD tHbx (au) | 0.15 (0.13 – 0.17) | 0.14 (0.13 – 0.15) | 0.14 (0.13 – 0.16) | 0.15 (0.13 – 0.18) | 0.13 (0.13 – 0.13) | 0.15 (0.13 – 0.17) |
|  | MAD of ARHD HbDiffx (au) | 0.14 (0.12 – 0.16) | 0.14 (0.13 – 0.14) | 0.14 (0.12 – 0.16) | 0.14 (0.12 – 0.17) | 0.12 (0.12 – 0.12) | 0.14 (0.12 – 0.16) |
| Occipital | ARHD of COx-a (au) | 0.24 (0.11 – 0.42) | 0.23 (0.11 – 0.41) | 0.24 (0.11 – 0.42) | 0.24 (0.11 – 0.41) | 0.15 (0.07 – 0.3) | 0.24 (0.11 – 0.42) |
|  | ARHD of HbOx (au) | 0.23 (0.11 – 0.42) | 0.23 (0.1 – 0.38) | 0.22 (0.1 – 0.38) | 0.26 (0.12 – 0.42) | 0.17 (0.07 – 0.33) | 0.23 (0.11 – 0.4) |
|  | ARHD of HHbx (au) | 0.23 (0.1 – 0.42) | 0.22 (0.11 – 0.39) | 0.23 (0.1 – 0.41) | 0.23 (0.1 – 0.42) | 0.18 (0.07 – 0.37) | 0.23 (0.1 – 0.42) |
|  | ARHD of tHbx (au) | 0.22 (0.1 – 0.43) | 0.23 (0.12 – 0.39) | 0.21 (0.09 – 0.38) | 0.25 (0.12 – 0.45) | 0.16 (0.08 – 0.33) | 0.22 (0.1 – 0.41) |
|  | ARHD of HbDiffx (au) | 0.24 (0.1 – 0.4) | 0.23 (0.11 – 0.4) | 0.24 (0.11 – 0.4) | 0.23 (0.1 – 0.4) | 0.15 (0.06 – 0.27) | 0.23 (0.1 – 0.41) |
|  | MAD of ARHD COx-a (au) | 0.14 (0.12 – 0.15) | 0.14 (0.12 – 0.16) | 0.14 (0.12 – 0.16) | 0.14 (0.12 – 0.15) | 0.1 (0.1 – 0.1) | 0.14 (0.12 – 0.16) |
|  | MAD of ARHD HbOx (au) | 0.14 (0.11 – 0.17) | 0.14 (0.13 – 0.16) | 0.13 (0.11 – 0.15) | 0.15 (0.13 – 0.17) | 0.11 (0.11 – 0.11) | 0.14 (0.12 – 0.16) |
|  | MAD of ARHD HHbx (au) | 0.14 (0.12 – 0.16) | 0.13 (0.11 – 0.16) | 0.14 (0.12 – 0.15) | 0.15 (0.11 – 0.17) | 0.12 (0.12 – 0.12) | 0.14 (0.12 – 0.16) |
|  | MAD of ARHD tHbx (au) | 0.14 (0.12 – 0.16) | 0.13 (0.12 – 0.16) | 0.14 (0.12 – 0.16) | 0.15 (0.12 – 0.16) | 0.1 (0.1 – 0.1) | 0.14 (0.12 – 0.16) |
|  | MAD of ARHD HbDiffx (au) | 0.14 (0.11 – 0.16) | 0.14 (0.13 – 0.15) | 0.14 (0.11 – 0.16) | 0.14 (0.12 – 0.16) | 0.11 (0.11 – 0.11) | 0.14 (0.12 – 0.16) |
| **250 Hz Sampled Data** | | | | | | | |
| Frontal | ARHD of COx-a (au) | 0.23 (0.11 – 0.41) | 0.22 (0.11 – 0.4) | 0.22 (0.1 – 0.38) | 0.24 (0.11 – 0.44) | 0.21 (0.1 – 0.35) | 0.23 (0.11 – 0.41) |
|  | ARHD of HbOx (au) | 0.22 (0.09 – 0.41) | 0.23 (0.09 – 0.41) | 0.2 (0.09 – 0.41) | 0.23 (0.1 – 0.41) | 0.2 (0.08 – 0.34) | 0.22 (0.09 – 0.42) |
|  | ARHD of HHbx (au) | 0.22 (0.1 – 0.41) | 0.22 (0.09 – 0.43) | 0.21 (0.09 – 0.41) | 0.23 (0.1 – 0.42) | 0.17 (0.08 – 0.3) | 0.23 (0.1 – 0.42) |
|  | ARHD of tHbx (au) | 0.23 (0.11 – 0.43) | 0.22 (0.09 – 0.42) | 0.21 (0.1 – 0.44) | 0.24 (0.11 – 0.42) | 0.19 (0.08 – 0.37) | 0.23 (0.11 – 0.43) |
|  | ARHD of HbDiffx (au) | 0.23 (0.11 – 0.4) | 0.23 (0.09 – 0.4) | 0.21 (0.11 – 0.38) | 0.24 (0.12 – 0.45) | 0.24 (0.12 – 0.38) | 0.22 (0.11 – 0.41) |
|  | MAD of ARHD COx-a (au) | 0.14 (0.12 – 0.17) | 0.13 (0.12 – 0.14) | 0.14 (0.11 – 0.15) | 0.14 (0.12 – 0.17) | 0.13 (0.13 – 0.13) | 0.14 (0.12 – 0.17) |
|  | MAD of ARHD HbOx (au) | 0.14 (0.12 – 0.18) | 0.14 (0.12 – 0.15) | 0.14 (0.12 – 0.18) | 0.15 (0.13 – 0.17) | 0.13 (0.13 – 0.13) | 0.14 (0.12 – 0.17) |
|  | MAD of ARHD HHbx (au) | 0.14 (0.11 – 0.18) | 0.15 (0.13 – 0.17) | 0.14 (0.1 – 0.18) | 0.15 (0.13 – 0.19) | 0.1 (0.1 – 0.1) | 0.14 (0.12 – 0.18) |
|  | MAD of ARHD tHbx (au) | 0.14 (0.12 – 0.17) | 0.15 (0.12 – 0.16) | 0.14 (0.12 – 0.17) | 0.15 (0.12 – 0.16) | 0.12 (0.12 – 0.12) | 0.15 (0.12 – 0.16) |
|  | MAD of ARHD HbDiffx (au) | 0.13 (0.12 – 0.15) | 0.15 (0.12 – 0.15) | 0.13 (0.12 – 0.15) | 0.15 (0.12 – 0.18) | 0.13 (0.13 – 0.13) | 0.14 (0.12 – 0.15) |
| Parietal | ARHD of COx-a (au) | 0.22 (0.1 – 0.43) | 0.25 (0.11 – 0.46) | 0.22 (0.1 – 0.43) | 0.23 (0.11 – 0.45) | 0.16 (0.07 – 0.3) | 0.22 (0.1 – 0.45) |
|  | ARHD of HbOx (au) | 0.2 (0.08 – 0.42) | 0.22 (0.1 – 0.44) | 0.21 (0.08 – 0.41) | 0.22 (0.1 – 0.43) | 0.19 (0.1 – 0.34) | 0.21 (0.09 – 0.43) |
|  | ARHD of HHbx (au) | 0.23 (0.1 – 0.44) | 0.26 (0.1 – 0.49) | 0.23 (0.1 – 0.44) | 0.23 (0.1 – 0.46) | 0.18 (0.08 – 0.32) | 0.24 (0.1 – 0.44) |
|  | ARHD of tHbx (au) | 0.21 (0.09 – 0.43) | 0.25 (0.11 – 0.45) | 0.21 (0.1 – 0.41) | 0.25 (0.1 – 0.47) | 0.17 (0.08 – 0.34) | 0.22 (0.1 – 0.44) |
|  | ARHD of HbDiffx (au) | 0.21 (0.09 – 0.41) | 0.23 (0.1 – 0.45) | 0.22 (0.09 – 0.41) | 0.22 (0.1 – 0.43) | 0.16 (0.07 – 0.3) | 0.22 (0.1 – 0.43) |
|  | MAD of ARHD COx-a (au) | 0.14 (0.12 – 0.16) | 0.16 (0.14 – 0.17) | 0.14 (0.12 – 0.17) | 0.15 (0.13 – 0.16) | 0.11 (0.11 – 0.11) | 0.15 (0.13 – 0.17) |
|  | MAD of ARHD HbOx (au) | 0.14 (0.12 – 0.18) | 0.14 (0.13 – 0.16) | 0.13 (0.12 – 0.17) | 0.15 (0.12 – 0.18) | 0.11 (0.11 – 0.11) | 0.14 (0.12 – 0.17) |
|  | MAD of ARHD HHbx (au) | 0.15 (0.14 – 0.17) | 0.16 (0.13 – 0.18) | 0.14 (0.13 – 0.17) | 0.16 (0.14 – 0.19) | 0.11 (0.11 – 0.11) | 0.15 (0.14 – 0.17) |
|  | MAD of ARHD tHbx (au) | 0.15 (0.12 – 0.17) | 0.15 (0.13 – 0.19) | 0.14 (0.12 – 0.16) | 0.17 (0.15 – 0.19) | 0.11 (0.11 – 0.11) | 0.15 (0.13 – 0.18) |
|  | MAD of ARHD HbDiffx (au) | 0.14 (0.12 – 0.16) | 0.15 (0.13 – 0.17) | 0.14 (0.12 – 0.16) | 0.14 (0.12 – 0.17) | 0.1 (0.1 – 0.1) | 0.14 (0.12 – 0.17) |
| Temporal | ARHD of COx-a (au) | 0.23 (0.11 – 0.39) | 0.21 (0.1 – 0.42) | 0.21 (0.1 – 0.4) | 0.23 (0.1 – 0.4) | 0.18 (0.1 – 0.34) | 0.23 (0.1 – 0.41) |
|  | ARHD of HbOx (au) | 0.23 (0.11 – 0.44) | 0.21 (0.09 – 0.4) | 0.22 (0.1 – 0.42) | 0.24 (0.12 – 0.45) | 0.21 (0.11 – 0.4) | 0.23 (0.11 – 0.43) |
|  | ARHD of HHbx (au) | 0.21 (0.09 – 0.44) | 0.25 (0.11 – 0.45) | 0.21 (0.1 – 0.45) | 0.22 (0.1 – 0.44) | 0.21 (0.1 – 0.33) | 0.22 (0.1 – 0.45) |
|  | ARHD of tHbx (au) | 0.24 (0.1 – 0.45) | 0.22 (0.09 – 0.41) | 0.22 (0.09 – 0.41) | 0.25 (0.12 – 0.47) | 0.22 (0.1 – 0.41) | 0.23 (0.1 – 0.43) |
|  | ARHD of HbDiffx (au) | 0.23 (0.1 – 0.39) | 0.21 (0.09 – 0.39) | 0.21 (0.1 – 0.4) | 0.23 (0.1 – 0.39) | 0.2 (0.11 – 0.36) | 0.23 (0.1 – 0.39) |
|  | MAD of ARHD COx-a (au) | 0.13 (0.12 – 0.15) | 0.14 (0.13 – 0.15) | 0.13 (0.12 – 0.15) | 0.13 (0.12 – 0.15) | 0.11 (0.11 – 0.11) | 0.13 (0.12 – 0.15) |
|  | MAD of ARHD HbOx (au) | 0.15 (0.13 – 0.16) | 0.14 (0.12 – 0.15) | 0.14 (0.12 – 0.16) | 0.15 (0.14 – 0.16) | 0.13 (0.13 – 0.13) | 0.15 (0.13 – 0.16) |
|  | MAD of ARHD HHbx (au) | 0.15 (0.12 – 0.16) | 0.14 (0.13 – 0.17) | 0.15 (0.12 – 0.16) | 0.14 (0.12 – 0.17) | 0.11 (0.11 – 0.11) | 0.15 (0.12 – 0.16) |
|  | MAD of ARHD tHbx (au) | 0.16 (0.13 – 0.17) | 0.14 (0.13 – 0.15) | 0.14 (0.13 – 0.16) | 0.16 (0.14 – 0.17) | 0.14 (0.14 – 0.14) | 0.15 (0.13 – 0.17) |
|  | MAD of ARHD HbDiffx (au) | 0.13 (0.12 – 0.16) | 0.14 (0.13 – 0.15) | 0.14 (0.12 – 0.15) | 0.14 (0.12 – 0.17) | 0.11 (0.11 – 0.11) | 0.14 (0.12 – 0.16) |
| Occipital | ARHD of COx-a (au) | 0.24 (0.11 – 0.41) | 0.24 (0.12 – 0.41) | 0.25 (0.11 – 0.4) | 0.24 (0.12 – 0.41) | 0.15 (0.07 – 0.27) | 0.24 (0.11 – 0.41) |
|  | ARHD of HbOx (au) | 0.23 (0.11 – 0.41) | 0.24 (0.11 – 0.41) | 0.23 (0.11 – 0.4) | 0.25 (0.12 – 0.42) | 0.17 (0.08 – 0.38) | 0.24 (0.11 – 0.41) |
|  | ARHD of HHbx (au) | 0.24 (0.11 – 0.43) | 0.24 (0.11 – 0.41) | 0.23 (0.11 – 0.41) | 0.24 (0.11 – 0.43) | 0.17 (0.08 – 0.35) | 0.24 (0.11 – 0.42) |
|  | ARHD of tHbx (au) | 0.22 (0.1 – 0.4) | 0.24 (0.12 – 0.39) | 0.22 (0.09 – 0.38) | 0.26 (0.12 – 0.43) | 0.17 (0.08 – 0.36) | 0.23 (0.1 – 0.4) |
|  | ARHD of HbDiffx (au) | 0.24 (0.11 – 0.4) | 0.22 (0.11 – 0.4) | 0.24 (0.11 – 0.4) | 0.22 (0.11 – 0.4) | 0.13 (0.06 – 0.25) | 0.23 (0.11 – 0.4) |
|  | MAD of ARHD COx-a (au) | 0.14 (0.12 – 0.16) | 0.14 (0.12 – 0.16) | 0.14 (0.11 – 0.17) | 0.14 (0.13 – 0.16) | 0.09 (0.09 – 0.09) | 0.14 (0.12 – 0.16) |
|  | MAD of ARHD HbOx (au) | 0.14 (0.12 – 0.17) | 0.14 (0.12 – 0.16) | 0.13 (0.12 – 0.17) | 0.14 (0.12 – 0.17) | 0.12 (0.12 – 0.12) | 0.14 (0.12 – 0.17) |
|  | MAD of ARHD HHbx (au) | 0.14 (0.12 – 0.16) | 0.13 (0.11 – 0.15) | 0.14 (0.12 – 0.15) | 0.14 (0.12 – 0.16) | 0.12 (0.12 – 0.12) | 0.14 (0.12 – 0.16) |
|  | MAD of ARHD tHbx (au) | 0.14 (0.12 – 0.17) | 0.14 (0.12 – 0.16) | 0.14 (0.12 – 0.16) | 0.15 (0.12 – 0.17) | 0.11 (0.11 – 0.11) | 0.14 (0.12 – 0.17) |
|  | MAD of ARHD HbDiffx (au) | 0.14 (0.12 – 0.16) | 0.13 (0.13 – 0.15) | 0.14 (0.11 – 0.15) | 0.13 (0.12 – 0.15) | 0.08 (0.08 – 0.08) | 0.14 (0.12 – 0.15) |
| The table shows the subgrouped absolute regional hemispheric disparity analysis in on four brain lobes of NIRS-derived CVR indices using 10-second decimated data at 1 Hz and 250 Hz sampling frequencies. *ARHD, absolute regional hemispheric difference; au, arbitrary units; COx-a, cerebral oximetry index with arterial blood pressure; CVR, cerebrovascular reactivity index; HbDiffx, hemoglobin difference index; HbOx, oxyhemoglobin index; HHbx, deoxyhemoglobin index; IQR, interquartile range; MAD, median absolute deviation; tHbx, total hemoglobin index.* | | | | | | | |

Appendix S6h: Subgrouped Regional Hemispheric Disparity Analysis on Physiologic Signals Using 10-Second Decimated Data

| **Brain Lobe** | **Physiologic Variable** | **Median (IQR)** | | | | | |
| --- | --- | --- | --- | --- | --- | --- | --- |
|  |  | **Age < 40 [n=38]** | **Age 40 – 60 [n=12]** | **Males [n=28]** | **Females [n=22]** | **Left Hand Dominance [n=1]** | **Right Hand Dominance [n=49]** |
| **1 Hz Sampled Data** | | | | | | | |
| Frontal | ARHD of rSO_2_ (%) | 7.64 (6.17 – 9.22) | 6.05 (5.17 – 7.01) | 6.98 (5.97 – 8.59) | 7.23 (5.86 – 9.24) | 5.13 (1.48 – 7.5) | 7.16 (6.02 – 8.91) |
|  | ARHD of HbO (au) | 26.31 (17.64 – 36.94) | 22.35 (13.91 – 25.71) | 26.31 (19.39 – 36.94) | 22.56 (15.78 – 27.73) | 384.41 (29.43 – 401.94) | 24.82 (16.35 – 29.83) |
|  | ARHD of HHb (au) | 34.77 (25.12 – 40.33) | 23.38 (16.59 – 28.52) | 31.85 (22.86 – 38.25) | 26.4 (20.97 – 32.27) | 191.88 (29.42 – 199.96) | 27.4 (22.83 – 35.47) |
|  | ARHD of tHb (au) | 38.45 (29.45 – 48.68) | 28.66 (13.45 – 43.17) | 39.43 (28.79 – 51.76) | 33.69 (26.86 – 43.02) | 590.08 (58.49 – 600.15) | 34.13 (26.11 – 45.76) |
|  | ARHD of HbDiff (au) | 33.25 (24.92 – 42.68) | 20.59 (14.96 – 26.81) | 33.27 (24.92 – 42.96) | 22.97 (16.82 – 29.88) | 174.39 (4.58 – 204.83) | 27.83 (23.72 – 37.35) |
|  | MAD of ARHD rSO_2_ (%) | 1.15 (0.77 – 1.65) | 0.89 (0.69 – 1.12) | 0.91 (0.65 – 1.41) | 1.14 (0.89 – 1.8) | 3.01 (3.01 – 3.01) | 1.07 (0.72 – 1.44) |
|  | MAD of ARHD HbO (au) | 6.11 (4.03 – 12.61) | 5.46 (3.97 – 8.62) | 6.92 (4.69 – 12.92) | 5.42 (3.7 – 6.94) | 29.69 (29.69 – 29.69) | 6.09 (3.94 – 10.94) |
|  | MAD of ARHD HHb (au) | 5.72 (3.89 – 12.95) | 4.89 (3.02 – 7.73) | 5.79 (3.71 – 13.81) | 5.38 (3.89 – 9.04) | 19.7 (19.7 – 19.7) | 5.42 (3.88 – 10.25) |
|  | MAD of ARHD tHb (au) | 10.74 (6.32 – 22) | 11.72 (6.25 – 13.59) | 12.84 (6.76 – 23.58) | 7.91 (5.86 – 13.65) | 22.88 (22.88 – 22.88) | 11.08 (6.24 – 18.12) |
|  | MAD of ARHD HbDiff (au) | 5.43 (3.51 – 7.57) | 4.27 (3.1 – 4.64) | 4.91 (2.99 – 8.43) | 4.86 (3.59 – 7.04) | 43.33 (43.33 – 43.33) | 4.7 (3.14 – 7.34) |
| Parietal | ARHD of rSO_2_ (%) | 5.28 (3.16 – 7.09) | 4.75 (2.65 – 7.88) | 4.92 (3.36 – 6.72) | 5.47 (2.59 – 8.21) | 10.09 (8.01 – 11.64) | 5.14 (2.84 – 7.22) |
|  | ARHD of HbO (au) | 46.15 (30.78 – 70.63) | 49.95 (24.98 – 87.18) | 44.17 (25.03 – 56.82) | 54.29 (32.88 – 78.74) | 77.43 (70.25 – 90.96) | 45.19 (29.22 – 74.13) |
|  | ARHD of HHb (au) | 26.55 (15.15 – 43.43) | 36.13 (13.08 – 54.69) | 26.41 (12.45 – 43.43) | 27.13 (16.29 – 48.57) | 16.33 (11.72 – 31.35) | 26.87 (14.6 – 45.74) |
|  | ARHD of tHb (au) | 73.71 (41.06 – 116.79) | 77.94 (43.08 – 126.45) | 69.46 (29.48 – 104.24) | 84.11 (45.33 – 131.83) | 77.06 (58.21 – 110.66) | 70.36 (41.82 – 122.91) |
|  | ARHD of HbDiff (au) | 29.74 (21.27 – 48.13) | 34.7 (20.26 – 55.27) | 30.11 (22.39 – 46.46) | 29.74 (15.04 – 54.86) | 74.37 (60.24 – 88.4) | 28.89 (20.81 – 48.04) |
|  | MAD of ARHD rSO_2_ (%) | 1.4 (0.99 – 2) | 1.8 (1.31 – 2.91) | 1.4 (0.82 – 1.85) | 1.7 (1.12 – 2.83) | 1.82 (1.82 – 1.82) | 1.42 (1.07 – 2.22) |
|  | MAD of ARHD HbO (au) | 12.02 (7.58 – 24.92) | 29.13 (12.56 – 39.24) | 12.02 (8.35 – 25.28) | 19.4 (7.58 – 35.09) | 9.79 (9.79 – 9.79) | 15.82 (7.81 – 34.37) |
|  | MAD of ARHD HHb (au) | 11.65 (5.9 – 14.2) | 13.03 (5.22 – 29.55) | 9.84 (5.14 – 15.09) | 13.18 (6.66 – 18.35) | 7.2 (7.2 – 7.2) | 11.67 (5.7 – 15.53) |
|  | MAD of ARHD tHb (au) | 20.73 (11.4 – 36.25) | 41.39 (23.07 – 71.48) | 20.48 (12.22 – 38.34) | 29.48 (12.1 – 50.74) | 20.38 (20.38 – 20.38) | 25.94 (12.07 – 47.72) |
|  | MAD of ARHD HbDiff (au) | 9.42 (6.36 – 19.76) | 11.86 (6.85 – 28.3) | 10.47 (5.34 – 20.8) | 9.86 (6.42 – 23.68) | 14.11 (14.11 – 14.11) | 10.3 (6.39 – 23.53) |
| Temporal | ARHD of rSO_2_ (%) | 8.69 (7.06 – 9.84) | 4.23 (2.51 – 5.96) | 8.08 (6.67 – 9.71) | 7.01 (4.11 – 8.24) | 8.16 (6.53 – 9.9) | 7.98 (6.05 – 9.2) |
|  | ARHD of HbO (au) | 63.9 (43.45 – 76.29) | 66.48 (48.26 – 83.87) | 55.79 (41.06 – 65.1) | 78.6 (48.81 – 103.89) | 226.6 (193.18 – 302.76) | 57.85 (41.96 – 66.37) |
|  | ARHD of HHb (au) | 30.32 (17.14 – 45.4) | 44.38 (29.06 – 87.18) | 31.57 (22.89 – 47.01) | 29.25 (17.48 – 43.02) | 123.66 (39.21 – 153.89) | 30.52 (17.74 – 45.95) |
|  | ARHD of tHb (au) | 85.04 (41.95 – 122.61) | 127.4 (97.22 – 180.47) | 84.77 (39.79 – 106.66) | 120.52 (77.67 – 153.98) | 330.52 (237.77 – 457.39) | 99.8 (47.72 – 123.9) |
|  | ARHD of HbDiff (au) | 54.7 (43.83 – 70.39) | 40.18 (34.23 – 61.83) | 48.84 (43.27 – 63.28) | 62.84 (40.73 – 88.58) | 151.62 (97.4 – 164.17) | 53.52 (40.89 – 64.7) |
|  | MAD of ARHD rSO_2_ (%) | 1.19 (0.83 – 1.79) | 1.36 (1.01 – 1.54) | 1.25 (0.92 – 1.71) | 1.29 (0.84 – 2.16) | 1.7 (1.7 – 1.7) | 1.21 (0.89 – 1.75) |
|  | MAD of ARHD HbO (au) | 10.43 (5.65 – 22.62) | 17.87 (7.96 – 27.21) | 9.3 (5.88 – 22.2) | 17.84 (6.86 – 27.66) | 67.54 (67.54 – 67.54) | 10.69 (5.99 – 23.89) |
|  | MAD of ARHD HHb (au) | 7.88 (4.37 – 12.56) | 16.62 (7.03 – 22.77) | 8.64 (6.4 – 14.75) | 7.55 (3.98 – 20.56) | 36.49 (36.49 – 36.49) | 8.22 (4.98 – 16.57) |
|  | MAD of ARHD tHb (au) | 16.69 (9.4 – 28.32) | 33.73 (13.21 – 54.42) | 16.69 (10.64 – 32.08) | 20.32 (9.55 – 37.84) | 105.27 (105.27 – 105.27) | 17.18 (9.09 – 33.15) |
|  | MAD of ARHD HbDiff (au) | 9.21 (5.1 – 18.29) | 10.72 (6.85 – 21.54) | 6.81 (5.03 – 13.51) | 12.71 (6.96 – 22.99) | 18.32 (18.32 – 18.32) | 9.08 (6.02 – 18.8) |
| Occipital | ARHD of rSO_2_ (%) | 11.15 (10.36 – 13.47) | 9.95 (9.26 – 11.25) | 11.14 (10.33 – 12.3) | 9.42 (8.27 – 13.36) | 9.7 (8.87 – 10.35) | 10.63 (10.21 – 13.16) |
|  | ARHD of HbO (au) | 315.08 (298.82 – 335.94) | 314.96 (297.6 – 321.51) | 321.61 (310.09 – 343.6) | 256.92 (216.53 – 307.26) | 224.19 (203.48 – 236.93) | 316.56 (298.9 – 332.83) |
|  | ARHD of HHb (au) | 110.85 (95.45 – 130.24) | 93.1 (72.93 – 110.21) | 124.91 (111.31 – 137.14) | 65.19 (30.96 – 103.08) | 57.02 (40.98 – 67.09) | 108.95 (94.99 – 128.42) |
|  | ARHD of tHb (au) | 425.9 (398.77 – 464.32) | 421.14 (382.63 – 441.15) | 445.7 (427.48 – 481.19) | 323.79 (230.26 – 396.84) | 276.78 (253.45 – 301.73) | 422.91 (397.41 – 461.67) |
|  | ARHD of HbDiff (au) | 198.73 (189.38 – 214.53) | 192.4 (184.6 – 200.6) | 198.73 (189.98 – 211.54) | 192.68 (175.76 – 216.27) | 166.28 (151.82 – 175.65) | 197.7 (189.21 – 214.38) |
|  | MAD of ARHD rSO_2_ (%) | 0.76 (0.63 – 1.42) | 0.95 (0.6 – 1.45) | 0.71 (0.55 – 0.95) | 1.12 (0.76 – 2.16) | 0.74 (0.74 – 0.74) | 0.8 (0.61 – 1.42) |
|  | MAD of ARHD HbO (au) | 11.61 (6.97 – 18.81) | 21.11 (10.97 – 33.11) | 11.27 (7.94 – 17.07) | 19.72 (6.12 – 28.22) | 16.5 (16.5 – 16.5) | 13.68 (7.63 – 23.91) |
|  | MAD of ARHD HHb (au) | 10.21 (5.12 – 13.71) | 9.24 (6.31 – 14.2) | 9.18 (5.77 – 12.36) | 10.53 (5.14 – 16.1) | 13.15 (13.15 – 13.15) | 10 (5.44 – 13.89) |
|  | MAD of ARHD tHb (au) | 20.51 (11.25 – 30.67) | 26.79 (16.35 – 45.57) | 19.25 (12.15 – 27.9) | 25.55 (15.12 – 44.53) | 24.59 (24.59 – 24.59) | 20.73 (12.61 – 32.13) |
|  | MAD of ARHD HbDiff (au) | 7.28 (6.41 – 12.11) | 11.55 (8.5 – 21.85) | 7.26 (6.31 – 9.83) | 12.96 (7.17 – 27.62) | 10.72 (10.72 – 10.72) | 8.51 (6.64 – 13.25) |
| **250 Hz Sampled Data** | | | | | | | |
| Frontal | ARHD of rSO_2_ (%) | 7.64 (6.2 – 9.23) | 6.06 (5.17 – 7.02) | 6.97 (5.97 – 8.57) | 7.24 (5.86 – 9.25) | 4.89 (1.47 – 7.29) | 7.16 (6.02 – 8.83) |
|  | ARHD of HbO (au) | 26.38 (17.57 – 36.91) | 22.36 (13.92 – 25.71) | 26.38 (19.38 – 36.91) | 22.47 (15.55 – 27.6) | 383.65 (29.43 – 401.94) | 24.83 (16.33 – 30.11) |
|  | ARHD of HHb (au) | 34.77 (25.12 – 40.31) | 23.35 (16.57 – 28.5) | 31.78 (22.85 – 38.25) | 26.39 (20.99 – 32.26) | 191.79 (29.42 – 200.02) | 27.41 (22.83 – 35.46) |
|  | ARHD of tHb (au) | 38.59 (29.41 – 48.71) | 28.74 (13.48 – 43.18) | 39.44 (28.27 – 51.7) | 33.47 (26.79 – 43.04) | 589.84 (58.14 – 599.97) | 33.88 (25.92 – 45.81) |
|  | ARHD of HbDiff (au) | 33.19 (24.92 – 42.58) | 20.71 (15.08 – 26.83) | 33.23 (24.92 – 42.6) | 22.97 (16.79 – 29.91) | 174.76 (4.54 – 204.71) | 27.83 (23.78 – 37.36) |
|  | MAD of ARHD rSO_2_ (%) | 1.16 (0.79 – 1.65) | 0.89 (0.69 – 1.13) | 0.91 (0.66 – 1.38) | 1.16 (0.89 – 1.81) | 2.91 (2.91 – 2.91) | 1.08 (0.73 – 1.47) |
|  | MAD of ARHD HbO (au) | 6.11 (4.23 – 12.69) | 5.51 (3.98 – 8.59) | 6.94 (4.7 – 13.08) | 5.58 (3.8 – 6.91) | 30.84 (30.84 – 30.84) | 6.11 (4.23 – 10.9) |
|  | MAD of ARHD HHb (au) | 5.84 (3.89 – 12.96) | 5.01 (3 – 7.76) | 5.78 (3.72 – 13.85) | 5.57 (3.89 – 9.06) | 19.09 (19.09 – 19.09) | 5.52 (3.82 – 10.41) |
|  | MAD of ARHD tHb (au) | 10.58 (6.36 – 22.35) | 11.53 (6.4 – 13.67) | 12.97 (6.72 – 24.01) | 8.04 (5.86 – 13.78) | 23.36 (23.36 – 23.36) | 10.77 (6.32 – 17.98) |
|  | MAD of ARHD HbDiff (au) | 5.45 (3.48 – 7.7) | 4.25 (3.15 – 4.81) | 4.94 (2.99 – 8.26) | 4.93 (3.51 – 7.12) | 43.79 (43.79 – 43.79) | 4.89 (3.19 – 7.45) |
| Parietal | ARHD of rSO_2_ (%) | 5.28 (3.2 – 7.07) | 4.77 (2.54 – 7.91) | 4.92 (3.35 – 6.63) | 5.49 (2.41 – 8.29) | 10.42 (8.24 – 11.91) | 5.14 (2.8 – 7.17) |
|  | ARHD of HbO (au) | 45.93 (30.67 – 70.57) | 49.74 (24.82 – 87.16) | 44.19 (24.46 – 56.86) | 54.2 (33.5 – 78.73) | 77.38 (70 – 90.92) | 45.08 (29.58 – 74.09) |
|  | ARHD of HHb (au) | 26.47 (14.82 – 43.74) | 36.11 (13.07 – 54.39) | 26.47 (12.48 – 43.74) | 27.14 (15.75 – 48.64) | 16.18 (11.76 – 31.64) | 26.89 (13.78 – 45.75) |
|  | ARHD of tHb (au) | 74.11 (41.06 – 117.22) | 77.79 (43.56 – 126.98) | 69.86 (29.53 – 104.7) | 83.84 (46.99 – 131.72) | 77.21 (58.14 – 111.54) | 71 (42.76 – 122.89) |
|  | ARHD of HbDiff (au) | 29.81 (20.99 – 48.74) | 34.77 (19.99 – 55.39) | 30.21 (22.4 – 46.77) | 29.79 (15.15 – 55.05) | 74.29 (60.09 – 88.28) | 29.03 (20.81 – 48.69) |
|  | MAD of ARHD rSO_2_ (%) | 1.4 (1.01 – 1.94) | 1.81 (1.3 – 2.99) | 1.41 (0.83 – 1.78) | 1.72 (1.1 – 2.82) | 1.77 (1.77 – 1.77) | 1.43 (1.04 – 2.33) |
|  | MAD of ARHD HbO (au) | 12.01 (7.53 – 25.2) | 28.83 (12.53 – 38.81) | 11.96 (8.36 – 25.57) | 19.36 (7.53 – 34.92) | 10.1 (10.1 – 10.1) | 15.62 (7.68 – 34) |
|  | MAD of ARHD HHb (au) | 11.54 (5.92 – 14.21) | 13.02 (5.22 – 29.01) | 9.77 (5.12 – 15.02) | 13.23 (6.78 – 18.57) | 7.24 (7.24 – 7.24) | 11.54 (5.64 – 15.75) |
|  | MAD of ARHD tHb (au) | 20.47 (11.18 – 36.25) | 41.93 (22.88 – 70.56) | 20.31 (12.04 – 38.3) | 29.52 (11.98 – 50.97) | 20.34 (20.34 – 20.34) | 26.09 (11.97 – 48.29) |
|  | MAD of ARHD HbDiff (au) | 9.38 (6.4 – 19.33) | 12.01 (6.82 – 27.88) | 10.4 (5.54 – 20.39) | 9.94 (6.49 – 23.47) | 14.12 (14.12 – 14.12) | 10.18 (6.47 – 22.84) |
| Temporal | ARHD of rSO_2_ (%) | 8.61 (7.06 – 9.67) | 4.22 (2.44 – 5.91) | 7.95 (6.58 – 9.5) | 7.01 (4.09 – 8.24) | 7.85 (6.39 – 9.55) | 7.98 (5.99 – 9.19) |
|  | ARHD of HbO (au) | 63.98 (43.45 – 76.29) | 66.47 (48.23 – 83.86) | 55.91 (41.04 – 65.12) | 78.28 (48.76 – 103.83) | 227.39 (192.54 – 303.04) | 57.96 (41.96 – 66.49) |
|  | ARHD of HHb (au) | 30.29 (17.08 – 45.5) | 44.65 (29.14 – 87.09) | 31.61 (22.75 – 47.04) | 29.27 (17.16 – 42.81) | 123.82 (39.41 – 154.06) | 30.48 (17.7 – 46.03) |
|  | ARHD of tHb (au) | 85.31 (42.04 – 122.64) | 127.66 (97.13 – 180.47) | 84.86 (39.79 – 106.62) | 120.78 (77.69 – 154.17) | 334.41 (238.27 – 457.35) | 100.17 (47.75 – 124.01) |
|  | ARHD of HbDiff (au) | 54.67 (44.31 – 70.27) | 40.25 (34.32 – 61.95) | 48.81 (43.23 – 63.46) | 62.58 (41.04 – 88.91) | 151.75 (97.96 – 164.54) | 53.42 (41.31 – 64.5) |
|  | MAD of ARHD rSO_2_ (%) | 1.23 (0.82 – 1.72) | 1.27 (1.01 – 1.51) | 1.19 (0.91 – 1.62) | 1.32 (0.83 – 2.11) | 1.6 (1.6 – 1.6) | 1.2 (0.89 – 1.7) |
|  | MAD of ARHD HbO (au) | 10.61 (5.75 – 22.75) | 17.86 (7.88 – 27.16) | 9.37 (5.78 – 22.44) | 17.6 (6.93 – 27.65) | 66.81 (66.81 – 66.81) | 10.97 (5.83 – 22.82) |
|  | MAD of ARHD HHb (au) | 7.81 (4.33 – 12.82) | 15.81 (7.08 – 22.37) | 8.6 (6.39 – 14.93) | 7.58 (3.9 – 20.02) | 36.24 (36.24 – 36.24) | 8.24 (4.95 – 16.91) |
|  | MAD of ARHD tHb (au) | 16.44 (9.5 – 29.96) | 34.03 (13.48 – 54.83) | 16.44 (10.69 – 32.05) | 20.52 (9.6 – 38.26) | 104.44 (104.44 – 104.44) | 17.21 (9.11 – 33.26) |
|  | MAD of ARHD HbDiff (au) | 9.12 (5.05 – 18.19) | 10.77 (6.88 – 21.38) | 6.88 (4.98 – 13.64) | 12.58 (6.99 – 22.72) | 17.8 (17.8 – 17.8) | 8.71 (6.13 – 18.96) |
| Occipital | ARHD of rSO_2_ (%) | 10.91 (10.14 – 13.31) | 9.75 (8.98 – 11) | 11 (10.18 – 12.21) | 9.28 (8.11 – 13.14) | 9.38 (8.55 – 10.03) | 10.38 (9.88 – 12.95) |
|  | ARHD of HbO (au) | 315.17 (299.15 – 335.83) | 314.85 (297.89 – 321.58) | 321.43 (310.26 – 343.49) | 256.65 (218.88 – 306.77) | 224.22 (203.74 – 237.68) | 316.48 (298.96 – 332.3) |
|  | ARHD of HHb (au) | 111.12 (95.34 – 130.34) | 92.72 (73 – 110.01) | 124.82 (111.22 – 137.15) | 65.12 (31.03 – 102.95) | 56.83 (41.26 – 66.62) | 109.43 (94.59 – 128.56) |
|  | ARHD of tHb (au) | 425.34 (398.2 – 464.56) | 420.47 (383.13 – 441.14) | 445.89 (427.46 – 481.35) | 323.91 (229.87 – 396.85) | 277.37 (253.84 – 301.56) | 422.83 (397.48 – 462.16) |
|  | ARHD of HbDiff (au) | 199.36 (189.42 – 213.84) | 192.2 (184.81 – 200.9) | 199.36 (189.94 – 211.63) | 192.58 (175.75 – 216.08) | 166.38 (151.99 – 174.91) | 198.54 (189.12 – 213.15) |
|  | MAD of ARHD rSO_2_ (%) | 0.76 (0.62 – 1.41) | 0.96 (0.63 – 1.44) | 0.69 (0.55 – 0.95) | 1.12 (0.74 – 2.22) | 0.7 (0.7 – 0.7) | 0.82 (0.62 – 1.42) |
|  | MAD of ARHD HbO (au) | 11.56 (6.7 – 18.52) | 21.56 (11.17 – 33.23) | 11.46 (7.75 – 17.04) | 19.43 (6.04 – 28.22) | 16.85 (16.85 – 16.85) | 13.67 (7.38 – 24.29) |
|  | MAD of ARHD HHb (au) | 10.21 (4.8 – 13.56) | 9.24 (6.5 – 13.87) | 9.23 (5.58 – 12.5) | 10.62 (5.24 – 16.11) | 12.75 (12.75 – 12.75) | 10.13 (5.22 – 13.76) |
|  | MAD of ARHD tHb (au) | 20.53 (11.35 – 30.42) | 26.88 (16.42 – 45.13) | 19.63 (12.33 – 27.98) | 25.21 (14.75 – 43.95) | 23.81 (23.81 – 23.81) | 21 (12.82 – 31.61) |
|  | MAD of ARHD HbDiff (au) | 7.28 (6.19 – 12.08) | 11.67 (8.78 – 21.5) | 7.28 (6.19 – 9.57) | 13.12 (7.22 – 27.04) | 10.62 (10.62 – 10.62) | 8.53 (6.38 – 13.22) |
| *The table shows the perturbation subgrouped absolute regional hemispheric disparity analysis in on four brain lobes of physiologic signals using 10-second decimated data at 1 Hz and 250 Hz sampling frequencies. ARHD, absolute regional hemispheric difference; au, arbitrary units; HbDiff, hemoglobin difference; HbO, oxyhemoglobin; HHb, deoxyhemoglobin; IQR, interquartile range; MAD, median absolute deviation; rSO_2_, regional cerebral oxygen saturation; tHb, total hemoglobin.* | | | | | | | |

Appendix S6i: Subgrouped Regional Hemispheric Disparity Analysis on Physiologic Signals Using Raw Data

| **Brain Lobe** | **Physiologic Variable** | **Median (IQR)** | | | | | |
| --- | --- | --- | --- | --- | --- | --- | --- |
|  |  | **Age < 40 [n=38]** | **Age 40 – 60 [n=12]** | **Males [n=28]** | **Females [n=22]** | **Left Hand Dominance [n=1]** | **Right Hand Dominance [n=49]** |
| **1 Hz Sampled Data** | | | | | | | |
| Frontal | ARHD of rSO_2_ (%) | 7.56 (6.24 – 9.36) | 6.13 (5.26 – 7.13) | 6.86 (5.88 – 8.64) | 7.33 (5.9 – 9.37) | 5.54 (1.65 – 7.74) | 7.25 (6.09 – 9.13) |
|  | ARHD of HbO (au) | 26.43 (18.09 – 37.47) | 22.25 (13.33 – 26.56) | 26.43 (19.65 – 37.47) | 22.49 (15.06 – 31.21) | 57.86 (26.43 – 398.73) | 24.65 (15.6 – 32.91) |
|  | ARHD of HHb (au) | 32.47 (25.03 – 40.51) | 23.26 (16.55 – 28.24) | 29.93 (23.08 – 38.3) | 26.33 (20.85 – 32.57) | 52.72 (28.65 – 199.39) | 28 (22.8 – 35.25) |
|  | ARHD of tHb (au) | 39.64 (26.89 – 47.54) | 29.18 (14.16 – 45.38) | 39.64 (30.11 – 51.85) | 33.38 (25.26 – 45.38) | 108.84 (52.33 – 599.32) | 36.5 (24.74 – 46.29) |
|  | ARHD of HbDiff (au) | 33.01 (24.72 – 42.51) | 21.05 (13.8 – 26.29) | 33.05 (24.72 – 42.23) | 23.4 (17.22 – 31.49) | 29.26 (5 – 197.67) | 27.99 (24.35 – 36.55) |
|  | MAD of ARHD rSO_2_ (%) | 1.25 (0.86 – 1.72) | 0.82 (0.71 – 1.17) | 1.08 (0.77 – 1.5) | 1.2 (0.82 – 1.72) | 2.97 (2.97 – 2.97) | 1.17 (0.8 – 1.57) |
|  | MAD of ARHD HbO (au) | 7.37 (4.98 – 12.77) | 5.99 (4.27 – 8.6) | 7.83 (4.71 – 12.81) | 6.78 (4.66 – 9.39) | 57.18 (57.18 – 57.18) | 7.21 (4.66 – 11.41) |
|  | MAD of ARHD HHb (au) | 6.71 (3.97 – 13.28) | 5.01 (2.81 – 7.83) | 6.67 (3.61 – 13.95) | 6.19 (4.03 – 9.94) | 30.29 (30.29 – 30.29) | 6.25 (3.83 – 10.59) |
|  | MAD of ARHD tHb (au) | 11.29 (7.44 – 22.52) | 12.17 (7.29 – 15.13) | 12.31 (7.98 – 23.94) | 10.07 (7.36 – 15.61) | 84.65 (84.65 – 84.65) | 11.17 (7.36 – 19.22) |
|  | MAD of ARHD HbDiff (au) | 5.63 (3.6 – 8.91) | 4.24 (2.92 – 4.76) | 5.18 (3.23 – 9.8) | 5.14 (3.71 – 7.76) | 28.59 (28.59 – 28.59) | 5.03 (3.52 – 8.39) |
| Parietal | ARHD of rSO_2_ (%) | 5.05 (3.32 – 7.1) | 5.03 (2.65 – 8.07) | 4.99 (3.39 – 7.1) | 5.2 (2.4 – 8.69) | 10.44 (8.15 – 11.99) | 4.95 (2.96 – 7.84) |
|  | ARHD of HbO (au) | 46.4 (31.63 – 70.68) | 54.74 (26.21 – 87.18) | 39.09 (25.09 – 62.27) | 59.6 (34.18 – 79.64) | 77.47 (69.91 – 90.06) | 45.38 (30.56 – 72.32) |
|  | ARHD of HHb (au) | 27.59 (15.98 – 44.52) | 38.32 (13.65 – 55.76) | 28.51 (13.29 – 44.52) | 27.73 (16.14 – 49.12) | 15.62 (10.53 – 29.07) | 28.29 (15.1 – 46.03) |
|  | ARHD of tHb (au) | 73.06 (41.78 – 115.24) | 84.39 (48.15 – 129.92) | 65.57 (28.8 – 101.76) | 78.92 (50.35 – 132.26) | 71.95 (59.35 – 103.65) | 74.17 (44.99 – 123.34) |
|  | ARHD of HbDiff (au) | 29.67 (20.17 – 55.19) | 35.25 (21.65 – 69.79) | 31.18 (23.4 – 55.19) | 28.99 (16.26 – 64.13) | 78.27 (61.38 – 91.25) | 29.56 (20.03 – 61.48) |
|  | MAD of ARHD rSO_2_ (%) | 1.59 (1.1 – 2.41) | 2.24 (1.38 – 2.89) | 1.59 (0.93 – 2.04) | 2.14 (1.14 – 2.85) | 1.83 (1.83 – 1.83) | 1.66 (1.12 – 2.56) |
|  | MAD of ARHD HbO (au) | 13.23 (8 – 25.32) | 28.04 (11.38 – 43.66) | 12.65 (8.81 – 24.24) | 20.86 (8 – 35.09) | 9.31 (9.31 – 9.31) | 15.95 (8.49 – 31.99) |
|  | MAD of ARHD HHb (au) | 11.57 (6.89 – 16.26) | 13.18 (5.99 – 29.65) | 9.81 (6.09 – 16.86) | 13.33 (7.38 – 20.41) | 7.1 (7.1 – 7.1) | 12.62 (6.41 – 19.21) |
|  | MAD of ARHD tHb (au) | 20.95 (11.68 – 39.45) | 43.28 (21.63 – 74.28) | 20.3 (12.26 – 41.4) | 31.1 (13.76 – 54.61) | 16.22 (16.22 – 16.22) | 24.16 (11.98 – 48.6) |
|  | MAD of ARHD HbDiff (au) | 11.84 (6.67 – 23.02) | 13.73 (8.16 – 27.88) | 11.84 (6.85 – 21.57) | 13.18 (7.69 – 26.6) | 14.98 (14.98 – 14.98) | 12.69 (7.11 – 25.99) |
| Temporal | ARHD of rSO_2_ (%) | 8.68 (7.01 – 10.24) | 4.48 (3.03 – 5.99) | 8.27 (6.68 – 10.05) | 6.98 (4.21 – 8.22) | 8.31 (6.47 – 10.15) | 7.97 (6.16 – 9.58) |
|  | ARHD of HbO (au) | 62.29 (43.23 – 75.94) | 63.56 (46.25 – 83.11) | 56.16 (40.54 – 65.08) | 72.85 (47.95 – 102.92) | 244.28 (193.34 – 302.67) | 58.73 (41.99 – 65.71) |
|  | ARHD of HHb (au) | 31.32 (17.41 – 47.99) | 43.29 (28.57 – 86.72) | 32.58 (23.53 – 51.15) | 30.65 (16.3 – 43.35) | 124.55 (40.16 – 152.29) | 31.62 (18.84 – 48) |
|  | ARHD of tHb (au) | 78.83 (42.76 – 120.97) | 130.12 (94.87 – 180.73) | 78.83 (38.78 – 106.21) | 119.88 (76.12 – 152.2) | 385.5 (241.99 – 458.45) | 89.19 (47.9 – 121.03) |
|  | ARHD of HbDiff (au) | 56.39 (43.19 – 74.23) | 39.47 (31.62 – 57.98) | 48.42 (42.97 – 61.13) | 64.55 (39.45 – 90.17) | 149.34 (106.49 – 170.97) | 54.5 (40.53 – 64.6) |
|  | MAD of ARHD rSO_2_ (%) | 1.4 (1.04 – 2.21) | 1.22 (0.96 – 1.62) | 1.25 (0.98 – 1.97) | 1.47 (1.12 – 2.23) | 1.84 (1.84 – 1.84) | 1.35 (0.98 – 2.2) |
|  | MAD of ARHD HbO (au) | 11.74 (7.23 – 23.12) | 16.72 (9.19 – 31.76) | 10.62 (7.55 – 18.13) | 17.77 (10.28 – 30.91) | 56.27 (56.27 – 56.27) | 12.12 (8.77 – 23.29) |
|  | MAD of ARHD HHb (au) | 8.25 (6.34 – 14.78) | 14.2 (7.93 – 21.75) | 9.05 (7.36 – 16.38) | 8.5 (5.92 – 17.35) | 37.79 (37.79 – 37.79) | 8.74 (6.75 – 16.2) |
|  | MAD of ARHD tHb (au) | 17.22 (9.36 – 31.41) | 28.38 (16.3 – 52.22) | 18.33 (11.2 – 32.91) | 20.07 (9.35 – 37.04) | 86.59 (86.59 – 86.59) | 18.39 (9.83 – 33.78) |
|  | MAD of ARHD HbDiff (au) | 14.17 (4.99 – 20.44) | 14.26 (7.26 – 22.85) | 8.77 (5.27 – 17.2) | 17.31 (8.46 – 24.88) | 26.63 (26.63 – 26.63) | 14.11 (5.71 – 20.61) |
| Occipital | ARHD of rSO_2_ (%) | 11.34 (10.2 – 13.58) | 10.04 (8.78 – 11.62) | 11.24 (10.1 – 12.51) | 9.65 (8.3 – 13.08) | 9.78 (8.45 – 11.05) | 10.71 (9.69 – 13.07) |
|  | ARHD of HbO (au) | 319.22 (296.73 – 338.28) | 313.45 (297.62 – 325.79) | 322.31 (304.54 – 346.75) | 261.12 (215.7 – 312.07) | 218.32 (191.67 – 237.34) | 318.12 (297.45 – 334.44) |
|  | ARHD of HHb (au) | 114.12 (94.17 – 131.22) | 92.1 (73.12 – 109.51) | 124.14 (110.17 – 136.6) | 65.08 (32.97 – 102.32) | 52.03 (32.18 – 67.58) | 114.03 (93.65 – 130.9) |
|  | ARHD of tHb (au) | 431.95 (399.41 – 464.45) | 416.1 (383.64 – 441.81) | 443.71 (419.17 – 481.29) | 321.57 (229.77 – 398.48) | 270.8 (231.48 – 299.54) | 431.23 (394.29 – 463.75) |
|  | ARHD of HbDiff (au) | 200.09 (184.6 – 219.2) | 192.23 (175.63 – 208.15) | 200.09 (183.6 – 216.85) | 191.48 (173.56 – 218.61) | 166.36 (145.14 – 184.29) | 198.81 (184.06 – 218.9) |
|  | MAD of ARHD rSO_2_ (%) | 1.1 (0.91 – 1.74) | 1.44 (1.07 – 1.8) | 1.08 (0.88 – 1.28) | 1.46 (1.09 – 2.65) | 1.3 (1.3 – 1.3) | 1.1 (0.97 – 1.75) |
|  | MAD of ARHD HbO (au) | 14.17 (11.21 – 21.6) | 21.06 (14.44 – 38.21) | 14.17 (12.51 – 17.75) | 21.46 (10.87 – 34.13) | 21.8 (21.8 – 21.8) | 15.67 (12.35 – 26.84) |
|  | MAD of ARHD HHb (au) | 11.68 (7.78 – 16.38) | 11.26 (9.32 – 15.48) | 11.08 (8.61 – 14.56) | 12.74 (8 – 18.01) | 17.21 (17.21 – 17.21) | 11.09 (8.4 – 16.29) |
|  | MAD of ARHD tHb (au) | 23.15 (12.14 – 32.32) | 26.67 (16.08 – 43.53) | 23.15 (13.21 – 30.93) | 26.6 (15.39 – 45.19) | 31.5 (31.5 – 31.5) | 22.61 (13.65 – 34.36) |
|  | MAD of ARHD HbDiff (au) | 15.17 (12.39 – 19.13) | 18.72 (15.96 – 28.64) | 15.28 (12.58 – 16.65) | 19.64 (13.95 – 31.03) | 19.26 (19.26 – 19.26) | 15.64 (13.68 – 20.11) |
| **250 Hz Sampled Data** | | | | | | | |
| Frontal | ARHD of rSO_2_ (%) | 8.06 (6.06 – 9.43) | 6.12 (5.26 – 7.14) | 6.66 (5.77 – 8.54) | 7.9 (5.86 – 9.68) | 4.23 (1.53 – 8.28) | 7.45 (6.02 – 9.29) |
|  | ARHD of HbO (au) | 27.67 (19.17 – 40.55) | 22.24 (13.44 – 26.58) | 25.86 (19.17 – 38.11) | 23.87 (15.37 – 32.43) | 57.63 (26.4 – 420.66) | 25.02 (17.08 – 36.26) |
|  | ARHD of HHb (au) | 34.19 (25.02 – 42.49) | 23.28 (16.61 – 28.25) | 30.68 (22.89 – 38.3) | 30.43 (21.6 – 35.51) | 52.54 (28.63 – 214.78) | 28.13 (22.66 – 37.64) |
|  | ARHD of tHb (au) | 40.33 (27.64 – 54.42) | 29.13 (14.11 – 45.39) | 39.64 (30.25 – 51.85) | 36.59 (25.62 – 45.89) | 109.03 (52.2 – 609.56) | 39.12 (24.74 – 47.32) |
|  | ARHD of HbDiff (au) | 33.53 (25.09 – 44.42) | 21.01 (13.93 – 26.24) | 33.03 (24.55 – 43.42) | 26.69 (23.26 – 31.42) | 28.1 (5.13 – 205.6) | 28.45 (24.3 – 40.62) |
|  | MAD of ARHD rSO_2_ (%) | 1.3 (0.93 – 2.01) | 0.85 (0.77 – 1.17) | 1.1 (0.76 – 1.64) | 1.23 (0.9 – 1.96) | 3.23 (3.23 – 3.23) | 1.21 (0.83 – 1.71) |
|  | MAD of ARHD HbO (au) | 7.35 (5.02 – 16.7) | 6.11 (4.31 – 8.71) | 7.86 (4.75 – 15.17) | 6.8 (4.7 – 10.04) | 56.91 (56.91 – 56.91) | 7.28 (4.7 – 12.88) |
|  | MAD of ARHD HHb (au) | 7.5 (4.03 – 15.69) | 5.18 (2.79 – 7.8) | 6.61 (3.58 – 15.23) | 6.2 (4.11 – 11.79) | 28.89 (28.89 – 28.89) | 6.27 (3.99 – 13.94) |
|  | MAD of ARHD tHb (au) | 12.36 (7.78 – 26.93) | 12.17 (7.33 – 15.13) | 13.16 (8.05 – 27.85) | 11.58 (7.49 – 18.69) | 84.81 (84.81 – 84.81) | 12.3 (7.43 – 24.28) |
|  | MAD of ARHD HbDiff (au) | 6.19 (3.66 – 9.75) | 4.53 (2.98 – 5.29) | 5.47 (3.27 – 10.88) | 5.2 (3.84 – 8.42) | 26.07 (26.07 – 26.07) | 5.13 (3.53 – 8.79) |
| Parietal | ARHD of rSO_2_ (%) | 7.18 (3.67 – 11.28) | 6.72 (3.15 – 10.51) | 6.85 (3.53 – 11.16) | 6.99 (3.43 – 10.87) | 10.06 (5.2 – 15.28) | 6.74 (3.46 – 10.8) |
|  | ARHD of HbO (au) | 52.06 (31.66 – 86.13) | 51.05 (26.63 – 94.59) | 50.46 (28.01 – 87.79) | 60.17 (30.89 – 86.13) | 77.72 (56.14 – 106.31) | 51.7 (30.54 – 85.91) |
|  | ARHD of HHb (au) | 39.99 (20.82 – 67.64) | 51.06 (22.04 – 92.73) | 43.72 (20.82 – 71.84) | 38.89 (21.24 – 73.02) | 24.31 (12.11 – 40.9) | 42.93 (21.06 – 74.54) |
|  | ARHD of tHb (au) | 75.76 (34.37 – 120.39) | 82.5 (46.78 – 128.99) | 75.28 (30.77 – 113.28) | 76.82 (50.25 – 132.33) | 77.75 (54.15 – 109.01) | 75.07 (37.54 – 123.23) |
|  | ARHD of HbDiff (au) | 46.9 (26.04 – 77.1) | 50.81 (26.67 – 106.97) | 50.2 (26.83 – 80.97) | 44.28 (23.22 – 81.9) | 76.12 (40.54 – 112.26) | 42.84 (25.78 – 79.57) |
|  | MAD of ARHD rSO_2_ (%) | 3.81 (1.51 – 4.9) | 3.08 (2.37 – 4.51) | 3.39 (1.47 – 4.62) | 3.39 (1.78 – 4.61) | 5.01 (5.01 – 5.01) | 3.18 (1.62 – 4.57) |
|  | MAD of ARHD HbO (au) | 21.17 (10.58 – 45.42) | 30.45 (17.27 – 46.15) | 22.75 (10.59 – 44.96) | 25.79 (14.94 – 47.78) | 24.34 (24.34 – 24.34) | 24.06 (10.59 – 45.64) |
|  | MAD of ARHD HHb (au) | 19.18 (10.68 – 31.96) | 32.03 (12.85 – 37.59) | 24.85 (10.52 – 34.54) | 20.54 (12.12 – 33.14) | 13.73 (13.73 – 13.73) | 21.73 (10.77 – 34.39) |
|  | MAD of ARHD tHb (au) | 25.35 (18.36 – 46.31) | 43.26 (22.15 – 73.96) | 25.12 (16 – 43.86) | 33.06 (19.9 – 55.64) | 25.9 (25.9 – 25.9) | 28.18 (18.04 – 52.56) |
|  | MAD of ARHD HbDiff (au) | 21.88 (8.72 – 60.99) | 25.18 (10.91 – 51.3) | 23.73 (8.28 – 61.79) | 21.88 (10.6 – 46) | 35.82 (35.82 – 35.82) | 21.54 (9.2 – 61.47) |
| Temporal | ARHD of rSO_2_ (%) | 9.31 (6.07 – 14.05) | 6.76 (4.31 – 11.26) | 8.81 (6.17 – 12.4) | 9.63 (4.75 – 15) | 9.14 (4.84 – 14.02) | 9 (5.05 – 12.93) |
|  | ARHD of HbO (au) | 71.1 (44.68 – 103.56) | 86.42 (56.7 – 147.93) | 61.38 (42.55 – 76.56) | 86.32 (49.77 – 149.82) | 235.73 (142.19 – 332.67) | 82.56 (44.49 – 103.89) |
|  | ARHD of HHb (au) | 45.03 (24.62 – 72.31) | 59.61 (32.72 – 107.63) | 41.3 (24.62 – 59.74) | 61.86 (29.83 – 107.22) | 101.74 (44.69 – 165.39) | 47.21 (27.62 – 81.1) |
|  | ARHD of tHb (au) | 83.24 (42.86 – 122.37) | 130.2 (94.23 – 179.71) | 78.99 (36.35 – 111.54) | 118.72 (77.44 – 152.44) | 353.09 (229.11 – 447.22) | 89.48 (47.92 – 123.19) |
|  | ARHD of HbDiff (au) | 66.28 (46.72 – 90.24) | 71.03 (45.59 – 98.5) | 54.89 (44.44 – 63.55) | 88.6 (50.77 – 157.66) | 134.13 (61.91 – 220.57) | 60.29 (46.41 – 87.79) |
|  | MAD of ARHD rSO_2_ (%) | 3.96 (1.22 – 4.97) | 2.74 (1.28 – 5.96) | 2.5 (1.16 – 4.58) | 4.64 (1.69 – 5.99) | 4.51 (4.51 – 4.51) | 3.67 (1.19 – 5.07) |
|  | MAD of ARHD HbO (au) | 25.9 (9.55 – 53.36) | 29.33 (15.76 – 51.88) | 14.83 (9.02 – 47.79) | 33.95 (20.07 – 55.22) | 95.36 (95.36 – 95.36) | 24.85 (9.78 – 52.32) |
|  | MAD of ARHD HHb (au) | 22.98 (7.05 – 38.59) | 22.58 (11.58 – 39.08) | 15.44 (7.68 – 38.96) | 32.3 (9.65 – 38.59) | 60.31 (60.31 – 60.31) | 21.92 (7.77 – 38.6) |
|  | MAD of ARHD tHb (au) | 25.69 (14.16 – 38.61) | 33.55 (22.36 – 52.19) | 23.05 (15.71 – 36.66) | 32.79 (21.17 – 48.15) | 104.98 (104.98 – 104.98) | 30.83 (16.46 – 39.26) |
|  | MAD of ARHD HbDiff (au) | 27.13 (7.13 – 67.33) | 28.83 (8.02 – 64.4) | 13.83 (6.78 – 60.8) | 51.12 (8.86 – 78.82) | 77.95 (77.95 – 77.95) | 22.9 (7.14 – 65.6) |
| Occipital | ARHD of rSO_2_ (%) | 11.61 (6.82 – 17.63) | 10.57 (6.16 – 17.48) | 10.6 (6.95 – 16.66) | 11.53 (6.38 – 18.33) | 10.25 (5.81 – 16.28) | 11.25 (6.79 – 17.69) |
|  | ARHD of HbO (au) | 329.98 (252.26 – 375) | 334.58 (244.04 – 370.32) | 340.09 (253.83 – 390.04) | 260.98 (172.63 – 336.3) | 225.36 (139.7 – 280.1) | 335.68 (251.55 – 373.26) |
|  | ARHD of HHb (au) | 118.59 (66.67 – 156.27) | 98.75 (49.04 – 136.58) | 134.29 (75.56 – 163.12) | 77.75 (38.42 – 125.26) | 67.64 (34.74 – 100.66) | 117.98 (66.17 – 155.51) |
|  | ARHD of tHb (au) | 426.76 (371.4 – 482.88) | 419.08 (361.44 – 467.92) | 449.74 (397.13 – 495.39) | 312.56 (227.46 – 406.31) | 267.24 (202.55 – 318.64) | 426.82 (369.66 – 478.03) |
|  | ARHD of HbDiff (au) | 207.05 (114.33 – 290.51) | 209.09 (100.02 – 286.92) | 208.91 (113.41 – 292.28) | 202.15 (105.87 – 286.29) | 174.63 (91.52 – 258.1) | 208.48 (114.22 – 292.12) |
|  | MAD of ARHD rSO_2_ (%) | 4.98 (4.32 – 6.09) | 5.69 (4.65 – 6.26) | 4.79 (4.16 – 5.67) | 5.95 (5.09 – 6.68) | 5.04 (5.04 – 5.04) | 5.12 (4.29 – 6.18) |
|  | MAD of ARHD HbO (au) | 55.19 (43.94 – 62.52) | 64.43 (56.85 – 74.71) | 55.47 (43.98 – 61.15) | 59.33 (50.51 – 75.71) | 65.94 (65.94 – 65.94) | 56.47 (46 – 66.9) |
|  | MAD of ARHD HHb (au) | 38.35 (34.5 – 42.35) | 39.57 (35.1 – 43.02) | 40.63 (32.79 – 44.28) | 36.7 (35.2 – 38.87) | 32.95 (32.95 – 32.95) | 38.69 (35.02 – 42.98) |
|  | MAD of ARHD tHb (au) | 42.61 (32.25 – 47.71) | 51.22 (39.93 – 59.8) | 42.46 (34.73 – 47.84) | 46.2 (32.67 – 63.96) | 56.85 (56.85 – 56.85) | 43.45 (33.23 – 57.07) |
|  | MAD of ARHD HbDiff (au) | 83.05 (70.73 – 90.14) | 90.09 (85.11 – 93.14) | 83.05 (70.67 – 90.58) | 86.94 (79.05 – 93.02) | 83.22 (83.22 – 83.22) | 85.1 (71.15 – 92.55) |
| The table shows the perturbation subgrouped absolute regional hemispheric disparity analysis in on four brain lobes of physiologic signals using raw data at 1 Hz and 250 Hz sampling frequencies. *ARHD, absolute regional hemispheric difference; au, arbitrary units; HbDiff, hemoglobin difference; HbO, oxyhemoglobin; HHb, deoxyhemoglobin; IQR, interquartile range; MAD, median absolute deviation; rSO_2_, regional cerebral oxygen saturation; tHb, total hemoglobin.* | | | | | | | |

Appendix S6j: Subgrouped Optimal ARIMA Models Based on AIC of Physiologic Signals and their Hemispheric Disparity

| **Physiologic Variable** | **Brain Lobe** | **Hemisphere** | **Optimal ARIMA Models (Median [IQR]) for Subgroups** | | | | | |
| --- | --- | --- | --- | --- | --- | --- | --- | --- |
|  |  |  | **Age < 40 [n=38]** | **Age 40 – 60 [n=12]** | **Males [n=28]** | **Females [n=22]** | **Left Hand Dominance [n=1]** | **Right Hand Dominance [n=49]** |
| **1 Hz Sampled Data** | | | | | | | | |
| ABP | – | – | (4,1,4) [(2,1,6) – (6,1,3)] | (4,1,7) [(4,1,1) – (5,1,4)] | (4,1,3) [(2,1,6) – (6,1,0)] | (4,1,7) [(3,1,3) – (6,1,6)] | (2,1,5) [(2,1,5) – (2,1,5)] | (4,1,7) [(3,1,3) – (6,1,3)] |
| rSO_2_ | Frontal | Left | (2,1,3) [(1,1,2) – (4,1,3)] | (3,1,0) [(1,1,4) – (7,1,0)] | (3,1,0) [(1,1,5) – (4,1,2)] | (2,1,1) [(1,1,3) – (6,1,1)] | (10,1,0) [(10,1,0) – (10,1,0)] | (2,1,5) [(1,1,3) – (4,1,3)] |
|  |  | Right | (2,1,4) [(1,1,10) – (5,1,4)] | (4,1,6) [(2,1,3) – (5,1,6)] | (2,1,3) [(2,1,1) – (5,1,5)] | (3,1,2) [(2,1,2) – (5,1,6)] | (2,1,4) [(2,1,4) – (2,1,4)] | (3,1,1) [(2,1,1) – (5,1,6)] |
|  | Parietal | Left | (3,1,7) [(2,1,1) – (5,1,3)] | (3,1,3) [(1,1,8) – (6,1,4)] | (4,1,3) [(2,1,1) – (5,1,5)] | (3,1,3) [(1,1,10) – (5,1,5)] | (1,1,0) [(1,1,0) – (1,1,0)] | (3,1,7) [(2,1,0) – (5,1,5)] |
|  |  | Right | (4,1,4) [(2,1,8) – (7,1,8)] | (4,1,8) [(3,1,3) – (6,1,3)] | (4,1,3) [(2,1,8) – (7,1,8)] | (4,1,5) [(3,1,3) – (5,1,5)] | (3,1,7) [(3,1,7) – (3,1,7)] | (4,1,5) [(2,1,8) – (7,1,8)] |
|  | Temporal | Left | (3,1,3) [(2,1,3) – (5,1,4)] | (3,1,6) [(2,1,3) – (4,1,9)] | (3,1,2) [(2,1,1) – (4,1,7)] | (4,1,4) [(2,1,4) – (5,1,6)] | (8,1,1) [(8,1,1) – (8,1,1)] | (3,1,3) [(2,1,3) – (5,1,3)] |
|  |  | Right | (3,1,3) [(1,1,9) – (6,1,5)] | (5,1,1) [(1,1,5) – (6,1,3)] | (4,1,3) [(2,1,1) – (6,1,7)] | (2,1,1) [(1,1,4) – (5,1,8)] | (2,1,3) [(2,1,3) – (2,1,3)] | (3,1,4) [(1,1,5) – (6,1,5)] |
|  | Occipital | Left | (2,1,5) [(1,1,3) – (5,1,1)] | (3,1,4) [(1,1,4) – (3,1,5)] | (4,1,2) [(2,1,2) – (5,1,2)] | (2,1,1) [(1,1,1) – (3,1,4)] | (3,1,2) [(3,1,2) – (3,1,2)] | (2,1,5) [(1,1,3) – (5,1,1)] |
|  |  | Right | (3,1,4) [(2,1,1) – (4,1,10)] | (3,1,5) [(2,1,2) – (6,1,3)] | (3,1,3) [(2,1,1) – (4,1,10)] | (4,1,3) [(2,1,1) – (5,1,5)] | (5,1,1) [(5,1,1) – (5,1,1)] | (3,1,4) [(2,1,1) – (5,1,2)] |
| HbO | Frontal | Left | (3,1,4) [(2,1,0) – (6,1,10)] | (7,1,9) [(3,1,3) – (8,1,6)] | (3,1,3) [(2,1,0) – (5,1,10)] | (5,1,0) [(2,1,4) – (8,1,3)] | (1,1,4) [(1,1,4) – (1,1,4)] | (4,1,3) [(2,1,4) – (7,1,7)] |
|  |  | Right | (3,1,4) [(1,1,6) – (6,1,4)] | (4,1,5) [(3,1,3) – (6,1,6)] | (4,1,5) [(2,1,0) – (6,1,6)] | (3,1,3) [(2,1,2) – (4,1,10)] | (1,1,0) [(1,1,0) – (1,1,0)] | (3,1,5) [(2,1,2) – (6,1,5)] |
|  | Parietal | Left | (3,1,5) [(2,1,2) – (5,1,6)] | (5,1,8) [(2,1,2) – (7,1,1)] | (4,1,4) [(2,1,1) – (5,1,8)] | (3,1,2) [(2,1,2) – (5,1,10)] | (7,1,1) [(7,1,1) – (7,1,1)] | (3,1,6) [(2,1,2) – (5,1,8)] |
|  |  | Right | (5,1,4) [(3,1,2) – (6,1,6)] | (7,1,3) [(2,1,8) – (8,1,8)] | (5,1,6) [(2,1,8) – (6,1,9)] | (5,1,4) [(2,1,4) – (7,1,3)] | (6,1,6) [(6,1,6) – (6,1,6)] | (5,1,5) [(2,1,8) – (6,1,9)] |
|  | Temporal | Left | (3,1,4) [(2,1,1) – (5,1,2)] | (5,1,4) [(1,1,2) – (5,1,6)] | (3,1,8) [(1,1,10) – (5,1,3)] | (3,1,9) [(2,1,1) – (5,1,6)] | (1,1,8) [(1,1,8) – (1,1,8)] | (3,1,9) [(2,1,1) – (5,1,5)] |
|  |  | Right | (3,1,4) [(2,1,2) – (5,1,9)] | (5,1,5) [(3,1,3) – (7,1,3)] | (4,1,5) [(2,1,7) – (6,1,5)] | (3,1,3) [(2,1,2) – (5,1,5)] | (4,1,5) [(4,1,5) – (4,1,5)] | (3,1,5) [(2,1,5) – (6,1,4)] |
|  | Occipital | Left | (4,1,5) [(2,1,1) – (6,1,1)] | (3,1,4) [(2,1,3) – (4,1,5)] | (5,1,3) [(2,1,6) – (6,1,1)] | (2,1,8) [(1,1,4) – (5,1,6)] | (6,1,4) [(6,1,4) – (6,1,4)] | (4,1,3) [(2,1,1) – (5,1,6)] |
|  |  | Right | (4,1,4) [(2,1,1) – (6,1,3)] | (6,1,5) [(4,1,2) – (7,1,7)] | (5,1,1) [(2,1,4) – (6,1,3)] | (4,1,4) [(2,1,8) – (7,1,7)] | (1,1,10) [(1,1,10) – (1,1,10)] | (4,1,9) [(2,1,8) – (6,1,7)] |
| HHb | Frontal | Left | (3,1,3) [(1,1,6) – (5,1,5)] | (6,1,6) [(2,1,3) – (8,1,4)] | (3,1,1) [(2,1,2) – (5,1,9)] | (3,1,5) [(1,1,9) – (8,1,3)] | (1,1,6) [(1,1,6) – (1,1,6)] | (3,1,3) [(2,1,2) – (7,1,4)] |
|  |  | Right | (4,1,0) [(2,1,2) – (6,1,1)] | (3,1,4) [(2,1,1) – (4,1,2)] | (4,1,2) [(2,1,3) – (5,1,8)] | (3,1,4) [(2,1,1) – (4,1,5)] | (1,1,0) [(1,1,0) – (1,1,0)] | (3,1,10) [(2,1,2) – (5,1,8)] |
|  | Parietal | Left | (3,1,4) [(2,1,0) – (5,1,10)] | (2,1,5) [(1,1,4) – (5,1,4)] | (3,1,4) [(1,1,10) – (4,1,5)] | (4,1,7) [(1,1,4) – (6,1,9)] | (3,1,2) [(3,1,2) – (3,1,2)] | (3,1,4) [(1,1,7) – (5,1,10)] |
|  |  | Right | (4,1,3) [(2,1,1) – (7,1,10)] | (3,1,4) [(2,1,1) – (5,1,8)] | (5,1,7) [(2,1,6) – (8,1,2)] | (2,1,8) [(1,1,6) – (4,1,3)] | (8,1,7) [(8,1,7) – (8,1,7)] | (3,1,4) [(2,1,1) – (7,1,7)] |
|  | Temporal | Left | (2,1,5) [(2,1,1) – (4,1,6)] | (4,1,5) [(2,1,3) – (5,1,6)] | (2,1,8) [(2,1,2) – (4,1,6)] | (3,1,1) [(2,1,2) – (5,1,2)] | (3,1,4) [(3,1,4) – (3,1,4)] | (2,1,9) [(2,1,2) – (4,1,9)] |
|  |  | Right | (2,1,10) [(1,1,9) – (6,1,8)] | (5,1,5) [(2,1,3) – (7,1,7)] | (3,1,9) [(2,1,2) – (6,1,8)] | (2,1,9) [(1,1,3) – (7,1,9)] | (4,1,3) [(4,1,3) – (4,1,3)] | (3,1,6) [(1,1,9) – (7,1,5)] |
|  | Occipital | Left | (3,1,5) [(2,1,6) – (5,1,9)] | (3,1,1) [(2,1,3) – (5,1,7)] | (3,1,5) [(2,1,10) – (5,1,7)] | (3,1,4) [(2,1,1) – (6,1,1)] | (5,1,7) [(5,1,7) – (5,1,7)] | (3,1,4) [(2,1,3) – (5,1,9)] |
|  |  | Right | (3,1,6) [(2,1,3) – (6,1,3)] | (6,1,3) [(5,1,7) – (8,1,5)] | (5,1,4) [(3,1,1) – (6,1,3)] | (4,1,9) [(2,1,4) – (8,1,5)] | (7,1,2) [(7,1,2) – (7,1,2)] | (4,1,9) [(2,1,5) – (6,1,6)] |
| tHb | Frontal | Left | (3,1,8) [(2,1,3) – (6,1,7)] | (5,1,7) [(4,1,10) – (6,1,9)] | (4,1,2) [(2,1,3) – (5,1,7)] | (4,1,6) [(3,1,1) – (8,1,3)] | (5,1,2) [(5,1,2) – (5,1,2)] | (4,1,5) [(2,1,4) – (6,1,9)] |
|  |  | Right | (4,1,1) [(1,1,7) – (6,1,5)] | (3,1,4) [(2,1,0) – (4,1,7)] | (4,1,4) [(2,1,3) – (6,1,5)] | (2,1,1) [(1,1,6) – (6,1,1)] | (5,1,4) [(5,1,4) – (5,1,4)] | (3,1,10) [(1,1,7) – (6,1,5)] |
|  | Parietal | Left | (3,1,4) [(1,1,5) – (5,1,6)] | (3,1,7) [(2,1,4) – (4,1,2)] | (3,1,4) [(1,1,5) – (4,1,2)] | (4,1,6) [(2,1,4) – (5,1,9)] | (9,1,8) [(9,1,8) – (9,1,8)] | (3,1,6) [(1,1,7) – (4,1,10)] |
|  |  | Right | (4,1,7) [(2,1,3) – (7,1,9)] | (4,1,10) [(3,1,4) – (7,1,7)] | (4,1,10) [(3,1,5) – (7,1,9)] | (4,1,4) [(2,1,1) – (7,1,7)] | (9,1,10) [(9,1,10) – (9,1,10)] | (4,1,7) [(3,1,3) – (7,1,9)] |
|  | Temporal | Left | (3,1,4) [(1,1,8) – (6,1,0)] | (5,1,4) [(2,1,2) – (6,1,8)] | (4,1,1) [(1,1,9) – (6,1,5)] | (3,1,4) [(1,1,6) – (6,1,3)] | (1,1,8) [(1,1,8) – (1,1,8)] | (4,1,1) [(1,1,9) – (6,1,5)] |
|  |  | Right | (3,1,1) [(1,1,2) – (5,1,3)] | (4,1,8) [(4,1,3) – (7,1,1)] | (4,1,3) [(2,1,3) – (7,1,6)] | (3,1,3) [(1,1,7) – (4,1,4)] | (5,1,3) [(5,1,3) – (5,1,3)] | (3,1,5) [(1,1,10) – (5,1,3)] |
|  | Occipital | Left | (4,1,2) [(2,1,3) – (7,1,8)] | (2,1,10) [(1,1,2) – (4,1,5)] | (4,1,6) [(2,1,0) – (7,1,8)] | (3,1,2) [(2,1,1) – (5,1,1)] | (8,1,10) [(8,1,10) – (8,1,10)] | (3,1,3) [(2,1,1) – (6,1,4)] |
|  |  | Right | (3,1,9) [(2,1,0) – (6,1,5)] | (5,1,1) [(4,1,4) – (5,1,9)] | (3,1,9) [(2,1,0) – (6,1,3)] | (4,1,7) [(2,1,5) – (6,1,5)] | (6,1,5) [(6,1,5) – (6,1,5)] | (4,1,4) [(2,1,2) – (6,1,3)] |
| HbDiff | Frontal | Left | (3,1,10) [(3,1,1) – (5,1,8)] | (5,1,2) [(3,1,1) – (6,1,0)] | (3,1,10) [(3,1,3) – (5,1,7)] | (4,1,6) [(2,1,4) – (6,1,8)] | (6,1,2) [(6,1,2) – (6,1,2)] | (4,1,3) [(3,1,1) – (5,1,10)] |
|  |  | Right | (3,1,4) [(1,1,7) – (5,1,7)] | (4,1,6) [(3,1,2) – (5,1,7)] | (4,1,5) [(2,1,3) – (6,1,7)] | (3,1,3) [(1,1,7) – (4,1,7)] | (2,1,0) [(2,1,0) – (2,1,0)] | (3,1,8) [(2,1,2) – (5,1,7)] |
|  | Parietal | Left | (3,1,4) [(2,1,2) – (7,1,3)] | (4,1,2) [(1,1,5) – (5,1,7)] | (4,1,8) [(2,1,2) – (7,1,8)] | (3,1,3) [(2,1,1) – (5,1,7)] | (1,1,0) [(1,1,0) – (1,1,0)] | (3,1,5) [(2,1,1) – (7,1,1)] |
|  |  | Right | (4,1,10) [(3,1,3) – (7,1,8)] | (4,1,7) [(2,1,7) – (5,1,7)] | (5,1,6) [(2,1,10) – (7,1,8)] | (4,1,6) [(2,1,7) – (7,1,3)] | (3,1,7) [(3,1,7) – (3,1,7)] | (4,1,10) [(2,1,8) – (7,1,8)] |
|  | Temporal | Left | (4,1,4) [(2,1,4) – (7,1,7)] | (4,1,7) [(2,1,3) – (5,1,5)] | (4,1,4) [(2,1,3) – (8,1,1)] | (4,1,6) [(2,1,4) – (5,1,5)] | (8,1,1) [(8,1,1) – (8,1,1)] | (4,1,4) [(2,1,3) – (6,1,3)] |
|  |  | Right | (3,1,3) [(2,1,3) – (5,1,6)] | (4,1,6) [(3,1,1) – (6,1,4)] | (3,1,6) [(2,1,7) – (5,1,6)] | (3,1,3) [(2,1,3) – (6,1,4)] | (5,1,4) [(5,1,4) – (5,1,4)] | (3,1,4) [(2,1,3) – (6,1,4)] |
|  | Occipital | Left | (2,1,10) [(1,1,3) – (5,1,3)] | (3,1,4) [(2,1,2) – (4,1,4)] | (3,1,2) [(1,1,4) – (5,1,10)] | (3,1,4) [(2,1,2) – (4,1,3)] | (6,1,3) [(6,1,3) – (6,1,3)] | (3,1,2) [(1,1,4) – (5,1,1)] |
|  |  | Right | (3,1,8) [(2,1,3) – (6,1,10)] | (3,1,9) [(3,1,7) – (4,1,8)] | (4,1,1) [(2,1,3) – (6,1,10)] | (3,1,7) [(3,1,3) – (5,1,8)] | (2,1,2) [(2,1,2) – (2,1,2)] | (3,1,9) [(2,1,7) – (6,1,8)] |
| COx-a | Frontal | Left | (2,1,1) [(1,1,5) – (3,1,5)] | (2,1,1) [(1,1,7) – (3,1,5)] | (2,1,6) [(1,1,7) – (3,1,6)] | (1,1,10) [(1,1,3) – (2,1,7)] | (5,1,2) [(5,1,2) – (5,1,2)] | (2,1,1) [(1,1,7) – (3,1,5)] |
|  |  | Right | (2,1,10) [(2,1,1) – (4,1,7)] | (3,1,6) [(2,1,9) – (5,1,5)] | (3,1,2) [(2,1,1) – (4,1,9)] | (3,1,2) [(1,1,6) – (5,1,5)] | (2,1,2) [(2,1,2) – (2,1,2)] | (3,1,2) [(2,1,1) – (5,1,2)] |
|  | Parietal | Left | (2,1,1) [(1,1,4) – (3,1,1)] | (3,1,2) [(1,1,4) – (5,1,7)] | (2,1,8) [(1,1,6) – (4,1,3)] | (1,1,9) [(1,1,3) – (3,1,2)] | (1,1,4) [(1,1,4) – (1,1,4)] | (2,1,4) [(1,1,4) – (3,1,7)] |
|  |  | Right | (2,1,9) [(2,1,0) – (4,1,7)] | (2,1,3) [(1,1,7) – (4,1,6)] | (2,1,10) [(2,1,1) – (4,1,6)] | (2,1,3) [(1,1,3) – (6,1,5)] | (3,1,3) [(3,1,3) – (3,1,3)] | (2,1,8) [(1,1,9) – (4,1,7)] |
|  | Temporal | Left | (2,1,6) [(1,1,5) – (4,1,8)] | (3,1,2) [(1,1,8) – (4,1,7)] | (2,1,10) [(1,1,4) – (5,1,0)] | (2,1,6) [(1,1,8) – (3,1,7)] | (2,1,3) [(2,1,3) – (2,1,3)] | (2,1,7) [(1,1,5) – (4,1,8)] |
|  |  | Right | (2,1,6) [(1,1,6) – (4,1,7)] | (2,1,7) [(1,1,7) – (2,1,8)] | (2,1,9) [(1,1,7) – (4,1,7)] | (2,1,4) [(1,1,3) – (3,1,1)] | (1,1,1) [(1,1,1) – (1,1,1)] | (2,1,6) [(1,1,6) – (4,1,3)] |
|  | Occipital | Left | (2,1,2) [(1,1,4) – (3,1,3)] | (2,1,3) [(1,1,1) – (3,1,4)] | (2,1,3) [(1,1,7) – (3,1,8)] | (2,1,1) [(1,1,0) – (3,1,0)] | (2,1,2) [(2,1,2) – (2,1,2)] | (2,1,2) [(1,1,1) – (3,1,4)] |
|  |  | Right | (2,1,6) [(1,1,7) – (5,1,10)] | (3,1,2) [(1,1,6) – (4,1,2)] | (3,1,2) [(1,1,8) – (6,1,5)] | (2,1,0) [(1,1,6) – (4,1,0)] | (1,1,2) [(1,1,2) – (1,1,2)] | (2,1,8) [(1,1,7) – (5,1,4)] |
| HbOx | Frontal | Left | (2,1,4) [(1,1,5) – (4,1,3)] | (5,1,6) [(3,1,3) – (6,1,2)] | (2,1,8) [(1,1,9) – (6,1,3)] | (2,1,2) [(1,1,4) – (4,1,5)] | (2,1,2) [(2,1,2) – (2,1,2)] | (2,1,8) [(1,1,5) – (5,1,5)] |
|  |  | Right | (2,1,1) [(1,1,6) – (4,1,5)] | (2,1,8) [(1,1,7) – (2,1,8)] | (2,1,1) [(1,1,6) – (3,1,2)] | (2,1,8) [(1,1,6) – (5,1,0)] | (1,1,0) [(1,1,0) – (1,1,0)] | (2,1,2) [(1,1,6) – (4,1,4)] |
|  | Parietal | Left | (2,1,9) [(1,1,9) – (4,1,1)] | (4,1,3) [(2,1,0) – (4,1,6)] | (3,1,1) [(2,1,1) – (4,1,3)] | (2,1,7) [(1,1,9) – (4,1,4)] | (7,1,7) [(7,1,7) – (7,1,7)] | (2,1,9) [(1,1,9) – (4,1,3)] |
|  |  | Right | (2,1,1) [(1,1,5) – (3,1,2)] | (4,1,6) [(2,1,7) – (4,1,7)] | (2,1,5) [(1,1,5) – (3,1,2)] | (2,1,4) [(1,1,6) – (5,1,4)] | (1,1,7) [(1,1,7) – (1,1,7)] | (2,1,5) [(1,1,6) – (4,1,6)] |
|  | Temporal | Left | (2,1,8) [(1,1,5) – (4,1,1)] | (3,1,7) [(1,1,9) – (4,1,6)] | (2,1,7) [(1,1,3) – (3,1,7)] | (3,1,3) [(1,1,8) – (5,1,10)] | (1,1,0) [(1,1,0) – (1,1,0)] | (2,1,8) [(1,1,6) – (4,1,4)] |
|  |  | Right | (2,1,2) [(1,1,6) – (4,1,1)] | (1,1,8) [(1,1,3) – (2,1,2)] | (2,1,2) [(1,1,8) – (4,1,1)] | (2,1,1) [(1,1,1) – (2,1,8)] | (2,1,0) [(2,1,0) – (2,1,0)] | (2,1,2) [(1,1,4) – (3,1,4)] |
|  | Occipital | Left | (2,1,9) [(1,1,2) – (6,1,1)] | (1,1,6) [(1,1,1) – (3,1,8)] | (3,1,8) [(2,1,1) – (6,1,7)] | (1,1,4) [(1,1,1) – (3,1,3)] | (2,1,1) [(2,1,1) – (2,1,1)] | (2,1,9) [(1,1,1) – (5,1,5)] |
|  |  | Right | (2,1,6) [(1,1,8) – (4,1,3)] | (2,1,7) [(1,1,9) – (3,1,5)] | (2,1,6) [(1,1,8) – (3,1,8)] | (2,1,7) [(1,1,9) – (4,1,6)] | (1,1,0) [(1,1,0) – (1,1,0)] | (2,1,7) [(1,1,9) – (4,1,3)] |
| HHbx | Frontal | Left | (2,1,10) [(1,1,10) – (6,1,1)] | (4,1,2) [(2,1,4) – (5,1,8)] | (3,1,5) [(1,1,9) – (5,1,3)] | (3,1,0) [(2,1,3) – (6,1,1)] | (1,1,4) [(1,1,4) – (1,1,4)] | (3,1,5) [(2,1,1) – (6,1,1)] |
|  |  | Right | (2,1,1) [(1,1,3) – (3,1,6)] | (5,1,3) [(1,1,8) – (6,1,6)] | (2,1,3) [(1,1,6) – (3,1,10)] | (2,1,1) [(1,1,6) – (5,1,3)] | (1,1,0) [(1,1,0) – (1,1,0)] | (2,1,1) [(1,1,6) – (4,1,7)] |
|  | Parietal | Left | (1,1,7) [(1,1,3) – (2,1,7)] | (2,1,7) [(1,1,5) – (2,1,10)] | (1,1,10) [(1,1,3) – (2,1,6)] | (1,1,8) [(1,1,3) – (3,1,0)] | (1,1,0) [(1,1,0) – (1,1,0)] | (1,1,10) [(1,1,3) – (2,1,9)] |
|  |  | Right | (2,1,3) [(1,1,8) – (4,1,5)] | (2,1,7) [(1,1,7) – (3,1,6)] | (2,1,7) [(1,1,7) – (4,1,3)] | (2,1,5) [(1,1,10) – (4,1,5)] | (2,1,3) [(2,1,3) – (2,1,3)] | (2,1,5) [(1,1,7) – (4,1,5)] |
|  | Temporal | Left | (2,1,4) [(1,1,7) – (3,1,9)] | (2,1,7) [(1,1,6) – (3,1,4)] | (2,1,4) [(1,1,6) – (3,1,7)] | (2,1,5) [(1,1,7) – (3,1,8)] | (1,1,1) [(1,1,1) – (1,1,1)] | (2,1,5) [(1,1,7) – (3,1,8)] |
|  |  | Right | (3,1,0) [(1,1,6) – (4,1,7)] | (2,1,8) [(1,1,5) – (4,1,9)] | (3,1,4) [(1,1,7) – (4,1,6)] | (2,1,8) [(1,1,1) – (4,1,9)] | (4,1,2) [(4,1,2) – (4,1,2)] | (2,1,10) [(1,1,5) – (4,1,9)] |
|  | Occipital | Left | (2,1,0) [(1,1,1) – (3,1,4)] | (2,1,6) [(1,1,5) – (3,1,3)] | (2,1,2) [(1,1,2) – (3,1,4)] | (2,1,0) [(1,1,1) – (3,1,4)] | (2,1,1) [(2,1,1) – (2,1,1)] | (2,1,2) [(1,1,1) – (3,1,4)] |
|  |  | Right | (2,1,0) [(1,1,4) – (4,1,4)] | (2,1,5) [(1,1,10) – (3,1,2)] | (1,1,10) [(1,1,4) – (3,1,3)] | (2,1,2) [(1,1,8) – (4,1,1)] | (1,1,2) [(1,1,2) – (1,1,2)] | (2,1,1) [(1,1,7) – (3,1,6)] |
| tHbx | Frontal | Left | (2,1,8) [(1,1,9) – (5,1,8)] | (3,1,4) [(1,1,7) – (4,1,10)] | (2,1,9) [(2,1,3) – (5,1,2)] | (2,1,9) [(1,1,5) – (6,1,2)] | (3,1,9) [(3,1,9) – (3,1,9)] | (2,1,9) [(1,1,9) – (5,1,2)] |
|  |  | Right | (2,1,7) [(1,1,9) – (4,1,5)] | (4,1,9) [(2,1,8) – (6,1,2)] | (2,1,9) [(1,1,9) – (4,1,3)] | (3,1,3) [(1,1,9) – (6,1,3)] | (2,1,0) [(2,1,0) – (2,1,0)] | (2,1,10) [(1,1,9) – (5,1,1)] |
|  | Parietal | Left | (2,1,1) [(1,1,4) – (3,1,2)] | (3,1,2) [(1,1,8) – (3,1,3)] | (2,1,6) [(1,1,9) – (3,1,3)] | (1,1,7) [(1,1,4) – (3,1,2)] | (2,1,1) [(2,1,1) – (2,1,1)] | (2,1,3) [(1,1,5) – (3,1,3)] |
|  |  | Right | (2,1,8) [(1,1,9) – (5,1,2)] | (2,1,8) [(1,1,10) – (4,1,6)] | (3,1,2) [(1,1,7) – (5,1,2)] | (2,1,8) [(2,1,2) – (4,1,6)] | (1,1,8) [(1,1,8) – (1,1,8)] | (2,1,8) [(1,1,10) – (5,1,1)] |
|  | Temporal | Left | (2,1,10) [(1,1,7) – (3,1,10)] | (3,1,8) [(2,1,5) – (4,1,4)] | (3,1,2) [(1,1,6) – (4,1,0)] | (3,1,0) [(1,1,9) – (3,1,10)] | (3,1,1) [(3,1,1) – (3,1,1)] | (3,1,1) [(1,1,7) – (4,1,0)] |
|  |  | Right | (2,1,4) [(1,1,7) – (5,1,4)] | (2,1,9) [(1,1,5) – (3,1,2)] | (2,1,9) [(1,1,7) – (4,1,1)] | (2,1,4) [(1,1,3) – (5,1,4)] | (1,1,0) [(1,1,0) – (1,1,0)] | (2,1,8) [(1,1,7) – (4,1,6)] |
|  | Occipital | Left | (2,1,7) [(1,1,2) – (3,1,9)] | (3,1,3) [(1,1,7) – (4,1,1)] | (3,1,8) [(1,1,10) – (4,1,4)] | (1,1,10) [(1,1,2) – (3,1,0)] | (1,1,2) [(1,1,2) – (1,1,2)] | (2,1,8) [(1,1,4) – (3,1,10)] |
|  |  | Right | (2,1,2) [(1,1,7) – (3,1,7)] | (3,1,4) [(2,1,1) – (3,1,8)] | (2,1,7) [(1,1,9) – (4,1,3)] | (2,1,1) [(1,1,7) – (3,1,4)] | (1,1,2) [(1,1,2) – (1,1,2)] | (2,1,7) [(1,1,7) – (3,1,8)] |
| HbDiffx | Frontal | Left | (1,1,8) [(1,1,1) – (2,1,9)] | (2,1,6) [(1,1,8) – (5,1,7)] | (1,1,9) [(1,1,1) – (3,1,4)] | (1,1,10) [(1,1,3) – (2,1,5)] | (4,1,8) [(4,1,8) – (4,1,8)] | (1,1,9) [(1,1,2) – (2,1,9)] |
|  |  | Right | (2,1,3) [(1,1,3) – (3,1,9)] | (2,1,10) [(1,1,7) – (5,1,2)] | (2,1,9) [(1,1,6) – (3,1,2)] | (1,1,8) [(1,1,2) – (5,1,4)] | (2,1,3) [(2,1,3) – (2,1,3)] | (2,1,5) [(1,1,4) – (5,1,2)] |
|  | Parietal | Left | (2,1,7) [(1,1,6) – (4,1,4)] | (3,1,2) [(2,1,4) – (5,1,7)] | (2,1,7) [(1,1,3) – (3,1,10)] | (3,1,2) [(2,1,1) – (5,1,4)] | (3,1,3) [(3,1,3) – (3,1,3)] | (2,1,8) [(1,1,8) – (4,1,7)] |
|  |  | Right | (2,1,8) [(1,1,7) – (4,1,2)] | (3,1,4) [(2,1,3) – (4,1,3)] | (2,1,8) [(1,1,7) – (3,1,9)] | (3,1,2) [(2,1,1) – (4,1,5)] | (3,1,3) [(3,1,3) – (3,1,3)] | (2,1,9) [(2,1,1) – (4,1,3)] |
|  | Temporal | Left | (2,1,3) [(1,1,3) – (3,1,4)] | (4,1,4) [(2,1,2) – (4,1,9)] | (2,1,3) [(1,1,10) – (3,1,7)] | (2,1,7) [(1,1,7) – (4,1,4)] | (1,1,0) [(1,1,0) – (1,1,0)] | (2,1,6) [(1,1,10) – (4,1,4)] |
|  |  | Right | (2,1,5) [(1,1,7) – (4,1,8)] | (3,1,1) [(2,1,1) – (3,1,6)] | (2,1,7) [(1,1,7) – (4,1,6)] | (2,1,4) [(1,1,7) – (3,1,8)] | (2,1,2) [(2,1,2) – (2,1,2)] | (2,1,7) [(1,1,7) – (4,1,5)] |
|  | Occipital | Left | (2,1,2) [(1,1,1) – (2,1,10)] | (1,1,3) [(1,1,1) – (4,1,7)] | (2,1,7) [(2,1,0) – (4,1,2)] | (1,1,1) [(1,1,0) – (2,1,8)] | (1,1,0) [(1,1,0) – (1,1,0)] | (2,1,2) [(1,1,1) – (3,1,0)] |
|  |  | Right | (2,1,0) [(1,1,4) – (3,1,4)] | (2,1,3) [(1,1,4) – (3,1,8)] | (2,1,3) [(1,1,6) – (3,1,6)] | (1,1,9) [(1,1,3) – (3,1,9)] | (1,1,1) [(1,1,1) – (1,1,1)] | (2,1,2) [(1,1,4) – (3,1,7)] |
| **250 Hz Sampled Data** | | | | | | | | |
| ABP | – | – | (4,1,10) [(2,1,10) – (7,1,3)] | (5,1,2) [(4,1,3) – (5,1,4)] | (5,1,1) [(3,1,3) – (6,1,3)] | (5,1,2) [(3,1,5) – (7,1,3)] | (6,1,1) [(6,1,1) – (6,1,1)] | (5,1,1) [(3,1,3) – (7,1,1)] |
| rSO_2_ | Frontal | Left | (3,1,4) [(2,1,1) – (5,1,8)] | (5,1,1) [(2,1,9) – (6,1,8)] | (3,1,6) [(1,1,6) – (5,1,7)] | (3,1,5) [(2,1,1) – (6,1,8)] | (9,1,5) [(9,1,5) – (9,1,5)] | (3,1,6) [(2,1,1) – (5,1,8)] |
|  |  | Right | (2,1,9) [(1,1,6) – (5,1,2)] | (3,1,5) [(3,1,3) – (3,1,6)] | (3,1,3) [(2,1,2) – (5,1,6)] | (3,1,3) [(1,1,7) – (4,1,1)] | (7,1,8) [(7,1,8) – (7,1,8)] | (3,1,3) [(2,1,0) – (4,1,1)] |
|  | Parietal | Left | (3,1,3) [(2,1,1) – (5,1,3)] | (2,1,6) [(1,1,1) – (4,1,6)] | (3,1,3) [(1,1,2) – (4,1,5)] | (3,1,3) [(2,1,6) – (5,1,4)] | (1,1,0) [(1,1,0) – (1,1,0)] | (3,1,3) [(2,1,1) – (5,1,3)] |
|  |  | Right | (3,1,8) [(2,1,1) – (6,1,8)] | (3,1,8) [(2,1,10) – (6,1,6)] | (3,1,8) [(2,1,7) – (6,1,8)] | (3,1,8) [(2,1,1) – (6,1,6)] | (3,1,7) [(3,1,7) – (3,1,7)] | (3,1,8) [(2,1,1) – (6,1,8)] |
|  | Temporal | Left | (3,1,4) [(2,1,1) – (6,1,7)] | (3,1,1) [(1,1,6) – (5,1,6)] | (3,1,3) [(2,1,0) – (5,1,3)] | (4,1,8) [(2,1,2) – (6,1,7)] | (1,1,7) [(1,1,7) – (1,1,7)] | (3,1,4) [(2,1,1) – (6,1,7)] |
|  |  | Right | (2,1,2) [(1,1,2) – (4,1,6)] | (3,1,3) [(2,1,2) – (3,1,6)] | (2,1,2) [(1,1,3) – (4,1,5)] | (2,1,5) [(1,1,5) – (4,1,10)] | (1,1,2) [(1,1,2) – (1,1,2)] | (2,1,5) [(1,1,3) – (4,1,6)] |
|  | Occipital | Left | (2,1,3) [(1,1,2) – (4,1,1)] | (3,1,1) [(1,1,3) – (4,1,9)] | (3,1,1) [(2,1,0) – (4,1,9)] | (2,1,1) [(1,1,2) – (3,1,1)] | (4,1,1) [(4,1,1) – (4,1,1)] | (2,1,3) [(1,1,2) – (4,1,2)] |
|  |  | Right | (3,1,6) [(2,1,1) – (5,1,0)] | (4,1,1) [(2,1,4) – (5,1,7)] | (3,1,9) [(2,1,1) – (5,1,2)] | (3,1,6) [(1,1,3) – (4,1,6)] | (1,1,5) [(1,1,5) – (1,1,5)] | (3,1,9) [(2,1,1) – (5,1,1)] |
| HbO | Frontal | Left | (3,1,7) [(2,1,3) – (5,1,7)] | (4,1,2) [(1,1,7) – (6,1,10)] | (3,1,3) [(2,1,0) – (5,1,0)] | (4,1,8) [(2,1,4) – (6,1,10)] | (1,1,4) [(1,1,4) – (1,1,4)] | (4,1,1) [(2,1,2) – (5,1,7)] |
|  |  | Right | (3,1,8) [(1,1,6) – (6,1,7)] | (3,1,3) [(1,1,7) – (4,1,8)] | (3,1,9) [(1,1,5) – (6,1,7)] | (2,1,6) [(1,1,7) – (4,1,4)] | (1,1,0) [(1,1,0) – (1,1,0)] | (3,1,4) [(1,1,7) – (6,1,1)] |
|  | Parietal | Left | (3,1,5) [(1,1,3) – (5,1,8)] | (4,1,4) [(2,1,2) – (5,1,2)] | (4,1,5) [(1,1,5) – (5,1,8)] | (2,1,3) [(1,1,3) – (4,1,7)] | (5,1,4) [(5,1,4) – (5,1,4)] | (3,1,5) [(1,1,4) – (5,1,8)] |
|  |  | Right | (4,1,8) [(3,1,4) – (6,1,8)] | (4,1,0) [(2,1,6) – (5,1,5)] | (4,1,7) [(2,1,8) – (5,1,9)] | (4,1,4) [(3,1,4) – (7,1,3)] | (8,1,9) [(8,1,9) – (8,1,9)] | (4,1,5) [(2,1,8) – (6,1,6)] |
|  | Temporal | Left | (2,1,10) [(2,1,2) – (4,1,9)] | (3,1,4) [(1,1,2) – (4,1,2)] | (2,1,5) [(2,1,2) – (4,1,6)] | (3,1,5) [(1,1,3) – (4,1,7)] | (1,1,8) [(1,1,8) – (1,1,8)] | (2,1,10) [(2,1,2) – (4,1,7)] |
|  |  | Right | (4,1,3) [(2,1,4) – (7,1,8)] | (4,1,3) [(2,1,3) – (6,1,10)] | (4,1,4) [(2,1,5) – (7,1,10)] | (3,1,8) [(1,1,2) – (5,1,1)] | (4,1,4) [(4,1,4) – (4,1,4)] | (4,1,3) [(2,1,3) – (7,1,6)] |
|  | Occipital | Left | (3,1,3) [(1,1,7) – (6,1,1)] | (4,1,3) [(3,1,1) – (5,1,7)] | (3,1,7) [(2,1,1) – (6,1,4)] | (3,1,3) [(1,1,6) – (4,1,3)] | (1,1,5) [(1,1,5) – (1,1,5)] | (3,1,4) [(2,1,1) – (6,1,1)] |
|  |  | Right | (4,1,6) [(1,1,9) – (7,1,9)] | (4,1,10) [(4,1,2) – (5,1,7)] | (4,1,8) [(2,1,3) – (6,1,5)] | (4,1,3) [(2,1,1) – (7,1,9)] | (4,1,6) [(4,1,6) – (4,1,6)] | (4,1,7) [(2,1,1) – (7,1,7)] |
| HHb | Frontal | Left | (4,1,1) [(2,1,4) – (7,1,10)] | (3,1,6) [(1,1,8) – (5,1,1)] | (4,1,1) [(2,1,2) – (6,1,1)] | (4,1,3) [(2,1,4) – (8,1,3)] | (6,1,1) [(6,1,1) – (6,1,1)] | (4,1,1) [(2,1,3) – (7,1,10)] |
|  |  | Right | (3,1,4) [(2,1,2) – (7,1,1)] | (2,1,10) [(2,1,0) – (3,1,4)] | (3,1,3) [(2,1,2) – (7,1,2)] | (3,1,2) [(2,1,1) – (4,1,3)] | (1,1,0) [(1,1,0) – (1,1,0)] | (3,1,3) [(2,1,2) – (6,1,1)] |
|  | Parietal | Left | (3,1,1) [(1,1,9) – (7,1,3)] | (4,1,7) [(2,1,5) – (6,1,7)] | (2,1,9) [(1,1,7) – (6,1,7)] | (4,1,2) [(2,1,3) – (7,1,7)] | (4,1,3) [(4,1,3) – (4,1,3)] | (3,1,3) [(1,1,10) – (7,1,3)] |
|  |  | Right | (5,1,2) [(2,1,5) – (7,1,5)] | (3,1,4) [(1,1,6) – (4,1,8)] | (5,1,2) [(2,1,5) – (7,1,1)] | (3,1,4) [(1,1,10) – (6,1,4)] | (8,1,7) [(8,1,7) – (8,1,7)] | (4,1,1) [(2,1,3) – (6,1,7)] |
|  | Temporal | Left | (3,1,4) [(2,1,1) – (5,1,2)] | (4,1,4) [(2,1,3) – (6,1,6)] | (3,1,3) [(1,1,10) – (5,1,9)] | (3,1,5) [(2,1,3) – (5,1,6)] | (1,1,7) [(1,1,7) – (1,1,7)] | (3,1,4) [(2,1,2) – (5,1,9)] |
|  |  | Right | (3,1,4) [(2,1,2) – (6,1,9)] | (5,1,2) [(2,1,4) – (6,1,8)] | (4,1,9) [(2,1,2) – (7,1,2)] | (3,1,4) [(1,1,7) – (5,1,10)] | (2,1,2) [(2,1,2) – (2,1,2)] | (3,1,5) [(2,1,2) – (6,1,9)] |
|  | Occipital | Left | (2,1,9) [(2,1,1) – (5,1,3)] | (3,1,6) [(1,1,3) – (6,1,2)] | (3,1,10) [(2,1,3) – (5,1,6)] | (2,1,4) [(1,1,2) – (5,1,1)] | (2,1,9) [(2,1,9) – (2,1,9)] | (3,1,6) [(1,1,6) – (5,1,6)] |
|  |  | Right | (4,1,4) [(3,1,3) – (7,1,1)] | (6,1,1) [(4,1,2) – (6,1,9)] | (5,1,9) [(3,1,4) – (7,1,2)] | (4,1,4) [(3,1,3) – (6,1,5)] | (7,1,2) [(7,1,2) – (7,1,2)] | (4,1,7) [(3,1,3) – (6,1,9)] |
| tHb | Frontal | Left | (3,1,3) [(2,1,3) – (5,1,10)] | (3,1,6) [(1,1,9) – (7,1,8)] | (3,1,1) [(2,1,0) – (5,1,1)] | (4,1,2) [(2,1,3) – (7,1,8)] | (5,1,1) [(5,1,1) – (5,1,1)] | (3,1,4) [(2,1,2) – (6,1,8)] |
|  |  | Right | (2,1,4) [(1,1,5) – (6,1,5)] | (3,1,4) [(2,1,1) – (4,1,2)] | (4,1,4) [(2,1,0) – (6,1,8)] | (2,1,1) [(1,1,6) – (4,1,4)] | (1,1,0) [(1,1,0) – (1,1,0)] | (3,1,4) [(1,1,7) – (6,1,5)] |
|  | Parietal | Left | (3,1,4) [(1,1,5) – (5,1,7)] | (6,1,1) [(3,1,2) – (7,1,7)] | (3,1,4) [(2,1,1) – (5,1,9)] | (3,1,4) [(1,1,4) – (7,1,6)] | (3,1,6) [(3,1,6) – (3,1,6)] | (3,1,4) [(2,1,0) – (6,1,6)] |
|  |  | Right | (5,1,5) [(3,1,4) – (7,1,8)] | (3,1,8) [(2,1,0) – (4,1,6)] | (6,1,6) [(3,1,5) – (7,1,8)] | (3,1,9) [(2,1,5) – (6,1,2)] | (8,1,8) [(8,1,8) – (8,1,8)] | (4,1,4) [(2,1,7) – (6,1,10)] |
|  | Temporal | Left | (2,1,10) [(1,1,10) – (5,1,5)] | (4,1,2) [(2,1,4) – (5,1,1)] | (3,1,3) [(2,1,2) – (5,1,1)] | (2,1,10) [(2,1,2) – (5,1,5)] | (1,1,8) [(1,1,8) – (1,1,8)] | (3,1,3) [(2,1,2) – (5,1,5)] |
|  |  | Right | (2,1,4) [(1,1,2) – (4,1,4)] | (5,1,2) [(4,1,1) – (6,1,4)] | (3,1,3) [(1,1,7) – (4,1,4)] | (3,1,3) [(1,1,3) – (5,1,7)] | (3,1,5) [(3,1,5) – (3,1,5)] | (3,1,3) [(1,1,3) – (5,1,3)] |
|  | Occipital | Left | (3,1,7) [(1,1,5) – (7,1,8)] | (3,1,4) [(3,1,1) – (8,1,3)] | (4,1,5) [(2,1,4) – (8,1,3)] | (3,1,1) [(1,1,2) – (4,1,5)] | (4,1,1) [(4,1,1) – (4,1,1)] | (3,1,6) [(1,1,5) – (7,1,8)] |
|  |  | Right | (3,1,5) [(1,1,10) – (6,1,6)] | (4,1,6) [(4,1,2) – (4,1,10)] | (3,1,7) [(2,1,0) – (5,1,8)] | (4,1,4) [(2,1,5) – (8,1,7)] | (2,1,6) [(2,1,6) – (2,1,6)] | (4,1,2) [(2,1,0) – (6,1,5)] |
| HbDiff | Frontal | Left | (3,1,4) [(2,1,3) – (5,1,4)] | (7,1,8) [(4,1,1) – (8,1,5)] | (4,1,3) [(3,1,1) – (6,1,7)] | (3,1,4) [(2,1,4) – (8,1,1)] | (9,1,2) [(9,1,2) – (9,1,2)] | (4,1,1) [(2,1,6) – (6,1,8)] |
|  |  | Right | (3,1,6) [(1,1,6) – (5,1,6)] | (4,1,2) [(1,1,9) – (5,1,2)] | (4,1,10) [(2,1,3) – (5,1,6)] | (2,1,1) [(1,1,5) – (4,1,1)] | (2,1,0) [(2,1,0) – (2,1,0)] | (3,1,7) [(1,1,6) – (5,1,6)] |
|  | Parietal | Left | (3,1,3) [(2,1,2) – (6,1,5)] | (3,1,10) [(1,1,5) – (5,1,10)] | (3,1,5) [(1,1,9) – (5,1,4)] | (4,1,2) [(2,1,2) – (6,1,5)] | (1,1,0) [(1,1,0) – (1,1,0)] | (3,1,5) [(2,1,2) – (6,1,3)] |
|  |  | Right | (4,1,2) [(2,1,4) – (6,1,5)] | (3,1,3) [(2,1,10) – (3,1,9)] | (4,1,3) [(3,1,1) – (6,1,5)] | (3,1,4) [(2,1,4) – (4,1,7)] | (6,1,5) [(6,1,5) – (6,1,5)] | (3,1,9) [(2,1,5) – (5,1,8)] |
|  | Temporal | Left | (3,1,8) [(2,1,3) – (6,1,7)] | (5,1,7) [(2,1,4) – (6,1,10)] | (3,1,8) [(2,1,1) – (5,1,7)] | (4,1,6) [(2,1,4) – (6,1,7)] | (1,1,7) [(1,1,7) – (1,1,7)] | (4,1,6) [(2,1,4) – (6,1,7)] |
|  |  | Right | (3,1,3) [(2,1,2) – (4,1,5)] | (5,1,6) [(4,1,4) – (6,1,3)] | (3,1,4) [(2,1,2) – (4,1,6)] | (3,1,8) [(2,1,6) – (6,1,3)] | (1,1,2) [(1,1,2) – (1,1,2)] | (3,1,6) [(2,1,3) – (5,1,6)] |
|  | Occipital | Left | (2,1,3) [(1,1,2) – (4,1,1)] | (3,1,6) [(1,1,2) – (5,1,3)] | (3,1,4) [(1,1,3) – (5,1,1)] | (2,1,1) [(1,1,2) – (3,1,5)] | (4,1,1) [(4,1,1) – (4,1,1)] | (2,1,3) [(1,1,2) – (4,1,4)] |
|  |  | Right | (3,1,6) [(2,1,1) – (7,1,7)] | (3,1,1) [(2,1,8) – (3,1,9)] | (3,1,1) [(2,1,1) – (7,1,4)] | (4,1,5) [(2,1,9) – (7,1,9)] | (2,1,3) [(2,1,3) – (2,1,3)] | (3,1,6) [(2,1,1) – (7,1,7)] |
| COx-a | Frontal | Left | (2,1,2) [(1,1,2) – (3,1,6)] | (3,1,6) [(2,1,4) – (5,1,3)] | (2,1,7) [(1,1,9) – (3,1,7)] | (2,1,2) [(1,1,1) – (4,1,1)] | (7,1,4) [(7,1,4) – (7,1,4)] | (2,1,5) [(1,1,5) – (3,1,7)] |
|  |  | Right | (2,1,6) [(1,1,9) – (3,1,7)] | (3,1,0) [(2,1,7) – (5,1,1)] | (2,1,8) [(2,1,1) – (3,1,7)] | (2,1,6) [(1,1,4) – (3,1,8)] | (3,1,2) [(3,1,2) – (3,1,2)] | (2,1,7) [(1,1,9) – (3,1,8)] |
|  | Parietal | Left | (2,1,2) [(1,1,7) – (3,1,9)] | (3,1,2) [(1,1,8) – (4,1,6)] | (2,1,1) [(1,1,7) – (3,1,9)] | (2,1,5) [(1,1,9) – (5,1,1)] | (1,1,0) [(1,1,0) – (1,1,0)] | (2,1,5) [(1,1,7) – (4,1,4)] |
|  |  | Right | (2,1,8) [(1,1,10) – (5,1,0)] | (2,1,6) [(2,1,1) – (3,1,1)] | (2,1,8) [(2,1,0) – (3,1,7)] | (2,1,6) [(1,1,7) – (5,1,5)] | (5,1,1) [(5,1,1) – (5,1,1)] | (2,1,6) [(1,1,10) – (4,1,4)] |
|  | Temporal | Left | (2,1,5) [(1,1,6) – (4,1,2)] | (3,1,5) [(2,1,5) – (5,1,1)] | (2,1,7) [(1,1,3) – (4,1,0)] | (2,1,8) [(2,1,1) – (5,1,2)] | (1,1,0) [(1,1,0) – (1,1,0)] | (2,1,8) [(1,1,9) – (4,1,3)] |
|  |  | Right | (2,1,6) [(1,1,3) – (4,1,8)] | (2,1,8) [(1,1,7) – (3,1,2)] | (2,1,4) [(1,1,3) – (3,1,6)] | (2,1,8) [(1,1,7) – (3,1,5)] | (1,1,1) [(1,1,1) – (1,1,1)] | (2,1,7) [(1,1,6) – (3,1,6)] |
|  | Occipital | Left | (2,1,0) [(1,1,0) – (3,1,5)] | (2,1,5) [(1,1,6) – (4,1,0)] | (2,1,2) [(1,1,2) – (3,1,2)] | (1,1,2) [(1,1,0) – (4,1,0)] | (1,1,0) [(1,1,0) – (1,1,0)] | (2,1,1) [(1,1,1) – (4,1,0)] |
|  |  | Right | (2,1,1) [(1,1,1) – (3,1,3)] | (2,1,6) [(1,1,7) – (5,1,2)] | (2,1,9) [(1,1,6) – (3,1,5)] | (1,1,9) [(1,1,0) – (3,1,6)] | (1,1,1) [(1,1,1) – (1,1,1)] | (2,1,2) [(1,1,6) – (3,1,6)] |
| HbOx | Frontal | Left | (3,1,3) [(1,1,7) – (5,1,10)] | (3,1,2) [(1,1,9) – (3,1,9)] | (3,1,4) [(1,1,7) – (5,1,2)] | (3,1,2) [(1,1,6) – (5,1,2)] | (5,1,2) [(5,1,2) – (5,1,2)] | (3,1,2) [(1,1,7) – (5,1,2)] |
|  |  | Right | (2,1,1) [(1,1,7) – (3,1,4)] | (2,1,10) [(2,1,5) – (4,1,2)] | (2,1,1) [(1,1,6) – (2,1,10)] | (2,1,8) [(2,1,1) – (4,1,3)] | (1,1,0) [(1,1,0) – (1,1,0)] | (2,1,5) [(1,1,8) – (3,1,8)] |
|  | Parietal | Left | (2,1,5) [(1,1,7) – (4,1,3)] | (3,1,4) [(1,1,8) – (3,1,6)] | (2,1,8) [(1,1,7) – (3,1,2)] | (2,1,0) [(1,1,7) – (5,1,10)] | (1,1,1) [(1,1,1) – (1,1,1)] | (2,1,7) [(1,1,7) – (4,1,2)] |
|  |  | Right | (2,1,1) [(1,1,2) – (3,1,3)] | (4,1,2) [(2,1,3) – (4,1,6)] | (2,1,3) [(1,1,5) – (3,1,5)] | (2,1,5) [(1,1,2) – (4,1,2)] | (1,1,8) [(1,1,8) – (1,1,8)] | (2,1,3) [(1,1,2) – (4,1,2)] |
|  | Temporal | Left | (2,1,4) [(1,1,7) – (4,1,8)] | (1,1,10) [(1,1,5) – (3,1,7)] | (2,1,1) [(1,1,3) – (3,1,6)] | (3,1,7) [(1,1,7) – (6,1,3)] | (1,1,0) [(1,1,0) – (1,1,0)] | (2,1,3) [(1,1,6) – (4,1,8)] |
|  |  | Right | (2,1,4) [(1,1,6) – (4,1,4)] | (2,1,6) [(1,1,9) – (3,1,8)] | (2,1,4) [(1,1,7) – (4,1,1)] | (2,1,6) [(1,1,7) – (4,1,4)] | (2,1,0) [(2,1,0) – (2,1,0)] | (2,1,6) [(1,1,7) – (4,1,2)] |
|  | Occipital | Left | (2,1,2) [(1,1,1) – (3,1,5)] | (2,1,7) [(1,1,4) – (3,1,2)] | (2,1,7) [(1,1,7) – (3,1,3)] | (1,1,8) [(1,1,1) – (3,1,5)] | (1,1,3) [(1,1,3) – (1,1,3)] | (2,1,5) [(1,1,2) – (3,1,5)] |
|  |  | Right | (2,1,2) [(1,1,6) – (4,1,1)] | (4,1,5) [(2,1,3) – (4,1,9)] | (3,1,6) [(1,1,9) – (4,1,6)] | (2,1,1) [(1,1,6) – (4,1,5)] | (1,1,0) [(1,1,0) – (1,1,0)] | (2,1,7) [(1,1,7) – (4,1,5)] |
| HHbx | Frontal | Left | (2,1,1) [(1,1,7) – (3,1,9)] | (3,1,8) [(1,1,9) – (6,1,0)] | (2,1,7) [(1,1,8) – (4,1,8)] | (2,1,1) [(1,1,7) – (5,1,3)] | (1,1,4) [(1,1,4) – (1,1,4)] | (2,1,3) [(1,1,7) – (5,1,2)] |
|  |  | Right | (2,1,1) [(1,1,7) – (3,1,4)] | (2,1,6) [(2,1,1) – (3,1,8)] | (2,1,2) [(1,1,7) – (3,1,4)] | (2,1,1) [(1,1,9) – (3,1,8)] | (2,1,1) [(2,1,1) – (2,1,1)] | (2,1,2) [(1,1,7) – (3,1,8)] |
|  | Parietal | Left | (2,1,5) [(1,1,7) – (3,1,4)] | (5,1,2) [(2,1,8) – (6,1,6)] | (2,1,2) [(1,1,7) – (3,1,2)] | (3,1,0) [(2,1,5) – (4,1,4)] | (1,1,0) [(1,1,0) – (1,1,0)] | (2,1,7) [(1,1,9) – (4,1,4)] |
|  |  | Right | (2,1,2) [(1,1,2) – (4,1,7)] | (3,1,2) [(2,1,3) – (3,1,10)] | (3,1,2) [(1,1,7) – (4,1,7)] | (2,1,3) [(1,1,5) – (4,1,4)] | (3,1,2) [(3,1,2) – (3,1,2)] | (2,1,5) [(1,1,7) – (4,1,7)] |
|  | Temporal | Left | (2,1,1) [(1,1,6) – (3,1,8)] | (2,1,2) [(1,1,8) – (2,1,5)] | (2,1,2) [(1,1,6) – (3,1,2)] | (2,1,0) [(1,1,7) – (3,1,4)] | (1,1,0) [(1,1,0) – (1,1,0)] | (2,1,2) [(1,1,7) – (3,1,3)] |
|  |  | Right | (2,1,4) [(1,1,6) – (4,1,8)] | (2,1,7) [(1,1,8) – (3,1,8)] | (2,1,6) [(1,1,6) – (4,1,8)] | (2,1,7) [(1,1,7) – (4,1,3)] | (2,1,3) [(2,1,3) – (2,1,3)] | (2,1,7) [(1,1,6) – (4,1,8)] |
|  | Occipital | Left | (2,1,1) [(1,1,1) – (3,1,3)] | (3,1,4) [(1,1,5) – (4,1,0)] | (2,1,3) [(1,1,2) – (4,1,4)] | (1,1,7) [(1,1,1) – (3,1,4)] | (1,1,1) [(1,1,1) – (1,1,1)] | (2,1,2) [(1,1,2) – (4,1,0)] |
|  |  | Right | (2,1,2) [(1,1,4) – (3,1,8)] | (2,1,4) [(1,1,9) – (3,1,7)] | (2,1,2) [(1,1,5) – (3,1,7)] | (2,1,2) [(1,1,0) – (3,1,7)] | (2,1,1) [(2,1,1) – (2,1,1)] | (2,1,2) [(1,1,5) – (3,1,7)] |
| tHbx | Frontal | Left | (2,1,3) [(1,1,7) – (4,1,2)] | (2,1,2) [(1,1,8) – (3,1,8)] | (2,1,3) [(1,1,7) – (3,1,9)] | (2,1,2) [(1,1,9) – (4,1,4)] | (5,1,8) [(5,1,8) – (5,1,8)] | (2,1,2) [(1,1,7) – (3,1,10)] |
|  |  | Right | (2,1,4) [(1,1,9) – (3,1,8)] | (2,1,2) [(1,1,7) – (2,1,7)] | (2,1,4) [(2,1,0) – (3,1,4)] | (2,1,2) [(1,1,5) – (3,1,8)] | (2,1,0) [(2,1,0) – (2,1,0)] | (2,1,4) [(1,1,9) – (3,1,7)] |
|  | Parietal | Left | (2,1,8) [(1,1,6) – (3,1,8)] | (2,1,8) [(1,1,8) – (4,1,2)] | (2,1,8) [(1,1,8) – (3,1,3)] | (2,1,9) [(1,1,4) – (4,1,4)] | (1,1,0) [(1,1,0) – (1,1,0)] | (2,1,8) [(1,1,7) – (4,1,1)] |
|  |  | Right | (2,1,3) [(1,1,7) – (4,1,4)] | (3,1,5) [(2,1,6) – (4,1,0)] | (2,1,8) [(2,1,0) – (4,1,1)] | (2,1,8) [(2,1,0) – (4,1,9)] | (1,1,8) [(1,1,8) – (1,1,8)] | (2,1,8) [(2,1,0) – (4,1,4)] |
|  | Temporal | Left | (3,1,1) [(1,1,6) – (4,1,7)] | (3,1,3) [(1,1,9) – (3,1,7)] | (3,1,1) [(1,1,6) – (3,1,7)] | (3,1,3) [(1,1,9) – (5,1,2)] | (3,1,3) [(3,1,3) – (3,1,3)] | (3,1,1) [(1,1,6) – (4,1,5)] |
|  |  | Right | (2,1,4) [(1,1,6) – (3,1,7)] | (2,1,8) [(1,1,9) – (3,1,3)] | (2,1,8) [(1,1,7) – (3,1,4)] | (2,1,4) [(1,1,1) – (3,1,2)] | (1,1,1) [(1,1,1) – (1,1,1)] | (2,1,6) [(1,1,7) – (3,1,4)] |
|  | Occipital | Left | (2,1,5) [(1,1,7) – (3,1,8)] | (3,1,2) [(1,1,7) – (4,1,1)] | (2,1,8) [(1,1,8) – (3,1,8)] | (2,1,1) [(1,1,3) – (3,1,4)] | (1,1,3) [(1,1,3) – (1,1,3)] | (2,1,7) [(1,1,7) – (3,1,8)] |
|  |  | Right | (2,1,5) [(1,1,6) – (3,1,8)] | (3,1,5) [(2,1,5) – (4,1,1)] | (2,1,6) [(1,1,9) – (4,1,0)] | (2,1,5) [(1,1,7) – (4,1,4)] | (1,1,2) [(1,1,2) – (1,1,2)] | (2,1,6) [(1,1,9) – (4,1,1)] |
| HbDiffx | Frontal | Left | (1,1,10) [(1,1,1) – (3,1,6)] | (2,1,8) [(1,1,7) – (3,1,9)] | (2,1,8) [(1,1,7) – (3,1,10)] | (1,1,5) [(1,1,1) – (3,1,3)] | (4,1,2) [(4,1,2) – (4,1,2)] | (2,1,0) [(1,1,2) – (3,1,6)] |
|  |  | Right | (2,1,3) [(1,1,6) – (4,1,1)] | (2,1,7) [(1,1,5) – (3,1,0)] | (2,1,4) [(1,1,6) – (3,1,5)] | (2,1,6) [(1,1,4) – (3,1,9)] | (2,1,2) [(2,1,2) – (2,1,2)] | (2,1,6) [(1,1,5) – (3,1,9)] |
|  | Parietal | Left | (1,1,10) [(1,1,5) – (4,1,1)] | (2,1,2) [(1,1,7) – (2,1,3)] | (1,1,8) [(1,1,3) – (2,1,5)] | (2,1,3) [(1,1,9) – (4,1,1)] | (1,1,0) [(1,1,0) – (1,1,0)] | (2,1,0) [(1,1,6) – (3,1,5)] |
|  |  | Right | (2,1,1) [(1,1,5) – (4,1,4)] | (3,1,0) [(2,1,0) – (3,1,4)] | (2,1,6) [(1,1,6) – (3,1,7)] | (2,1,2) [(1,1,8) – (4,1,3)] | (1,1,5) [(1,1,5) – (1,1,5)] | (2,1,3) [(1,1,7) – (4,1,3)] |
|  | Temporal | Left | (2,1,0) [(1,1,2) – (4,1,3)] | (2,1,0) [(1,1,5) – (2,1,8)] | (2,1,3) [(1,1,1) – (4,1,4)] | (1,1,10) [(1,1,6) – (2,1,8)] | (1,1,0) [(1,1,0) – (1,1,0)] | (2,1,0) [(1,1,5) – (3,1,9)] |
|  |  | Right | (3,1,3) [(1,1,7) – (6,1,3)] | (3,1,1) [(2,1,2) – (3,1,4)] | (3,1,4) [(2,1,2) – (6,1,1)] | (2,1,8) [(1,1,7) – (5,1,3)] | (2,1,2) [(2,1,2) – (2,1,2)] | (3,1,2) [(1,1,7) – (5,1,5)] |
|  | Occipital | Left | (2,1,0) [(1,1,0) – (3,1,10)] | (2,1,5) [(1,1,7) – (3,1,3)] | (2,1,2) [(1,1,0) – (3,1,10)] | (2,1,0) [(1,1,2) – (3,1,7)] | (1,1,0) [(1,1,0) – (1,1,0)] | (2,1,2) [(1,1,1) – (3,1,10)] |
|  |  | Right | (2,1,5) [(1,1,7) – (3,1,9)] | (2,1,2) [(1,1,5) – (3,1,8)] | (2,1,6) [(1,1,7) – (4,1,2)] | (2,1,0) [(1,1,3) – (3,1,8)] | (2,1,3) [(2,1,3) – (2,1,3)] | (2,1,5) [(1,1,6) – (3,1,9)] |
| The table provides subgrouped median and IQR of optimal ARIMA models based on AIC for physiologic signals using data in 1 Hz and 250 Hz frequencies. *AIC, Akaike Information Criterion; ARIMA, autoregressive integrative moving average; COx-a, cerebral oximetry index with arterial blood pressure; HbDiff, hemoglobin difference; HbDiffx, hemoglobin difference index; HbO, oxyhemoglobin; HbOx, oxyhemoglobin index; HHb, deoxyhemoglobin; HHbx, deoxyhemoglobin index; IQR, interquartile range; MAD, median absolute deviation; rSO_2_, regional cerebral oxygen saturation; tHb, total hemoglobin; tHbx, total hemoglobin index.* | | | | | | | | |

Appendix S6k: Subgrouped Hemispheric Responsiveness using Impulse Response Coefficients of Optimal VARIMA Model

| **Signal Combination** | **Brain Lobe** | **Hemisphere** | **Direction** | **Hemispheric Responsiveness using Impulse Response Coefficients of Optimal VARIMA Model [% (count)] for Subgroups** | | | | | | | | | | | |
| --- | --- | --- | --- | --- | --- | --- | --- | --- | --- | --- | --- | --- | --- | --- | --- |
|  |  |  |  | **Age < 40 [n=38]** | | **Age 40 – 60 [n=12]** | | **Males [n=28]** | | **Females [n=22]** | | **Left Hand Dominance [n=1]** | | **Right Hand Dominance [n=49]** | |
|  |  |  |  | **>0.1%** | **NA** | **>0.1%** | **NA** | **>0.1%** | **NA** | **>0.1%** | **NA** | **>0.1%** | **NA** | **>0.1%** | **NA** |
| **1 Hz Sampled Data** | | | | | | | | | | | | | | | |
| ABP & rSO_2_ | Frontal | Left | ABP ® rSO_2_ | 86.8% (33) | 2.6% (1) | 100% (12) | 0% (0) | 89.3% (25) | 0% (0) | 90.9% (20) | 4.5% (1) | 100% (1) | 0% (0) | 89.8% (44) | 2% (1) |
|  |  |  | rSO_2_ ® ABP | 89.5% (34) | 2.6% (1) | 91.7% (11) | 0% (0) | 89.3% (25) | 0% (0) | 90.9% (20) | 4.5% (1) | 100% (1) | 0% (0) | 89.8% (44) | 2% (1) |
|  |  | Right | ABP ® rSO_2_ | 86.8% (33) | 5.3% (2) | 91.7% (11) | 0% (0) | 78.6% (22) | 7.1% (2) | 100% (22) | 0% (0) | 100% (1) | 0% (0) | 87.8% (43) | 4.1% (2) |
|  |  |  | rSO_2_ ® ABP | 86.8% (33) | 5.3% (2) | 91.7% (11) | 0% (0) | 78.6% (22) | 7.1% (2) | 100% (22) | 0% (0) | 100% (1) | 0% (0) | 87.8% (43) | 4.1% (2) |
|  | Parietal | Left | ABP ® rSO_2_ | 92.1% (35) | 0% (0) | 100% (12) | 0% (0) | 89.3% (25) | 0% (0) | 100% (22) | 0% (0) | 0% (0) | 0% (0) | 95.9% (47) | 0% (0) |
|  |  |  | rSO_2_ ® ABP | 92.1% (35) | 0% (0) | 100% (12) | 0% (0) | 92.9% (26) | 0% (0) | 95.5% (21) | 0% (0) | 100% (1) | 0% (0) | 93.9% (46) | 0% (0) |
|  |  | Right | ABP ® rSO_2_ | 92.1% (35) | 0% (0) | 100% (12) | 0% (0) | 89.3% (25) | 0% (0) | 100% (22) | 0% (0) | 100% (1) | 0% (0) | 93.9% (46) | 0% (0) |
|  |  |  | rSO_2_ ® ABP | 92.1% (35) | 0% (0) | 100% (12) | 0% (0) | 89.3% (25) | 0% (0) | 100% (22) | 0% (0) | 100% (1) | 0% (0) | 93.9% (46) | 0% (0) |
|  | Occipital | Left | ABP ® rSO_2_ | 92.1% (35) | 5.3% (2) | 83.3% (10) | 8.3% (1) | 96.4% (27) | 0% (0) | 81.8% (18) | 13.6% (3) | 100% (1) | 0% (0) | 89.8% (44) | 6.1% (3) |
|  |  |  | rSO_2_ ® ABP | 92.1% (35) | 5.3% (2) | 83.3% (10) | 8.3% (1) | 100% (28) | 0% (0) | 77.3% (17) | 13.6% (3) | 100% (1) | 0% (0) | 89.8% (44) | 6.1% (3) |
|  |  | Right | ABP ® rSO_2_ | 92.1% (35) | 0% (0) | 91.7% (11) | 8.3% (1) | 96.4% (27) | 3.6% (1) | 86.4% (19) | 0% (0) | 100% (1) | 0% (0) | 91.8% (45) | 2% (1) |
|  |  |  | rSO_2_ ® ABP | 92.1% (35) | 0% (0) | 91.7% (11) | 8.3% (1) | 96.4% (27) | 3.6% (1) | 86.4% (19) | 0% (0) | 100% (1) | 0% (0) | 91.8% (45) | 2% (1) |
|  | Temporal | Left | ABP ® rSO_2_ | 89.5% (34) | 0% (0) | 100% (12) | 0% (0) | 92.9% (26) | 0% (0) | 90.9% (20) | 0% (0) | 100% (1) | 0% (0) | 91.8% (45) | 0% (0) |
|  |  |  | rSO_2_ ® ABP | 92.1% (35) | 0% (0) | 91.7% (11) | 0% (0) | 96.4% (27) | 0% (0) | 86.4% (19) | 0% (0) | 100% (1) | 0% (0) | 91.8% (45) | 0% (0) |
|  |  | Right | ABP ® rSO_2_ | 92.1% (35) | 0% (0) | 100% (12) | 0% (0) | 89.3% (25) | 0% (0) | 100% (22) | 0% (0) | 100% (1) | 0% (0) | 93.9% (46) | 0% (0) |
|  |  |  | rSO_2_ ® ABP | 94.7% (36) | 0% (0) | 100% (12) | 0% (0) | 92.9% (26) | 0% (0) | 100% (22) | 0% (0) | 100% (1) | 0% (0) | 95.9% (47) | 0% (0) |
| ABP & HbO | Frontal | Left | ABP ® HbO | 92.1% (35) | 0% (0) | 91.7% (11) | 0% (0) | 96.4% (27) | 0% (0) | 86.4% (19) | 0% (0) | 100% (1) | 0% (0) | 91.8% (45) | 0% (0) |
|  |  |  | HbO ® ABP | 94.7% (36) | 0% (0) | 100% (12) | 0% (0) | 100% (28) | 0% (0) | 90.9% (20) | 0% (0) | 100% (1) | 0% (0) | 95.9% (47) | 0% (0) |
|  |  | Right | ABP ® HbO | 84.2% (32) | 0% (0) | 83.3% (10) | 8.3% (1) | 82.1% (23) | 0% (0) | 86.4% (19) | 4.5% (1) | 100% (1) | 0% (0) | 83.7% (41) | 2% (1) |
|  |  |  | HbO ® ABP | 86.8% (33) | 0% (0) | 83.3% (10) | 8.3% (1) | 85.7% (24) | 0% (0) | 86.4% (19) | 4.5% (1) | 100% (1) | 0% (0) | 85.7% (42) | 2% (1) |
|  | Parietal | Left | ABP ® HbO | 81.6% (31) | 0% (0) | 100% (12) | 0% (0) | 82.1% (23) | 0% (0) | 90.9% (20) | 0% (0) | 0% (0) | 0% (0) | 87.8% (43) | 0% (0) |
|  |  |  | HbO ® ABP | 78.9% (30) | 0% (0) | 100% (12) | 0% (0) | 82.1% (23) | 0% (0) | 86.4% (19) | 0% (0) | 0% (0) | 0% (0) | 85.7% (42) | 0% (0) |
|  |  | Right | ABP ® HbO | 86.8% (33) | 2.6% (1) | 100% (12) | 0% (0) | 85.7% (24) | 3.6% (1) | 95.5% (21) | 0% (0) | 100% (1) | 0% (0) | 89.8% (44) | 2% (1) |
|  |  |  | HbO ® ABP | 86.8% (33) | 2.6% (1) | 100% (12) | 0% (0) | 85.7% (24) | 3.6% (1) | 95.5% (21) | 0% (0) | 100% (1) | 0% (0) | 89.8% (44) | 2% (1) |
|  | Occipital | Left | ABP ® HbO | 89.5% (34) | 2.6% (1) | 75% (9) | 0% (0) | 82.1% (23) | 0% (0) | 90.9% (20) | 4.5% (1) | 100% (1) | 0% (0) | 85.7% (42) | 2% (1) |
|  |  |  | HbO ® ABP | 86.8% (33) | 2.6% (1) | 75% (9) | 0% (0) | 78.6% (22) | 0% (0) | 90.9% (20) | 4.5% (1) | 100% (1) | 0% (0) | 83.7% (41) | 2% (1) |
|  |  | Right | ABP ® HbO | 84.2% (32) | 2.6% (1) | 100% (12) | 0% (0) | 85.7% (24) | 3.6% (1) | 90.9% (20) | 0% (0) | 100% (1) | 0% (0) | 87.8% (43) | 2% (1) |
|  |  |  | HbO ® ABP | 84.2% (32) | 2.6% (1) | 100% (12) | 0% (0) | 85.7% (24) | 3.6% (1) | 90.9% (20) | 0% (0) | 100% (1) | 0% (0) | 87.8% (43) | 2% (1) |
|  | Temporal | Left | ABP ® HbO | 94.7% (36) | 0% (0) | 100% (12) | 0% (0) | 96.4% (27) | 0% (0) | 95.5% (21) | 0% (0) | 100% (1) | 0% (0) | 95.9% (47) | 0% (0) |
|  |  |  | HbO ® ABP | 97.4% (37) | 0% (0) | 100% (12) | 0% (0) | 96.4% (27) | 0% (0) | 100% (22) | 0% (0) | 100% (1) | 0% (0) | 98% (48) | 0% (0) |
|  |  | Right | ABP ® HbO | 86.8% (33) | 5.3% (2) | 100% (12) | 0% (0) | 85.7% (24) | 7.1% (2) | 95.5% (21) | 0% (0) | 100% (1) | 0% (0) | 89.8% (44) | 4.1% (2) |
|  |  |  | HbO ® ABP | 84.2% (32) | 5.3% (2) | 100% (12) | 0% (0) | 82.1% (23) | 7.1% (2) | 95.5% (21) | 0% (0) | 100% (1) | 0% (0) | 87.8% (43) | 4.1% (2) |
| ABP & HHb | Frontal | Left | ABP ® HHb | 94.7% (36) | 0% (0) | 83.3% (10) | 0% (0) | 92.9% (26) | 0% (0) | 90.9% (20) | 0% (0) | 100% (1) | 0% (0) | 91.8% (45) | 0% (0) |
|  |  |  | HHb ® ABP | 92.1% (35) | 0% (0) | 83.3% (10) | 0% (0) | 89.3% (25) | 0% (0) | 90.9% (20) | 0% (0) | 100% (1) | 0% (0) | 89.8% (44) | 0% (0) |
|  |  | Right | ABP ® HHb | 94.7% (36) | 0% (0) | 91.7% (11) | 0% (0) | 96.4% (27) | 0% (0) | 90.9% (20) | 0% (0) | 100% (1) | 0% (0) | 93.9% (46) | 0% (0) |
|  |  |  | HHb ® ABP | 94.7% (36) | 0% (0) | 100% (12) | 0% (0) | 100% (28) | 0% (0) | 90.9% (20) | 0% (0) | 100% (1) | 0% (0) | 95.9% (47) | 0% (0) |
|  | Parietal | Left | ABP ® HHb | 78.9% (30) | 7.9% (3) | 91.7% (11) | 0% (0) | 78.6% (22) | 3.6% (1) | 86.4% (19) | 9.1% (2) | 100% (1) | 0% (0) | 81.6% (40) | 6.1% (3) |
|  |  |  | HHb ® ABP | 81.6% (31) | 7.9% (3) | 83.3% (10) | 0% (0) | 82.1% (23) | 3.6% (1) | 81.8% (18) | 9.1% (2) | 100% (1) | 0% (0) | 81.6% (40) | 6.1% (3) |
|  |  | Right | ABP ® HHb | 81.6% (31) | 2.6% (1) | 91.7% (11) | 0% (0) | 82.1% (23) | 3.6% (1) | 86.4% (19) | 0% (0) | 100% (1) | 0% (0) | 83.7% (41) | 2% (1) |
|  |  |  | HHb ® ABP | 81.6% (31) | 2.6% (1) | 100% (12) | 0% (0) | 85.7% (24) | 3.6% (1) | 86.4% (19) | 0% (0) | 100% (1) | 0% (0) | 85.7% (42) | 2% (1) |
|  | Occipital | Left | ABP ® HHb | 86.8% (33) | 2.6% (1) | 100% (12) | 0% (0) | 92.9% (26) | 0% (0) | 86.4% (19) | 4.5% (1) | 100% (1) | 0% (0) | 89.8% (44) | 2% (1) |
|  |  |  | HHb ® ABP | 86.8% (33) | 2.6% (1) | 100% (12) | 0% (0) | 92.9% (26) | 0% (0) | 86.4% (19) | 4.5% (1) | 100% (1) | 0% (0) | 89.8% (44) | 2% (1) |
|  |  | Right | ABP ® HHb | 86.8% (33) | 0% (0) | 91.7% (11) | 8.3% (1) | 89.3% (25) | 0% (0) | 86.4% (19) | 4.5% (1) | 100% (1) | 0% (0) | 87.8% (43) | 2% (1) |
|  |  |  | HHb ® ABP | 86.8% (33) | 0% (0) | 91.7% (11) | 8.3% (1) | 89.3% (25) | 0% (0) | 86.4% (19) | 4.5% (1) | 100% (1) | 0% (0) | 87.8% (43) | 2% (1) |
|  | Temporal | Left | ABP ® HHb | 89.5% (34) | 2.6% (1) | 100% (12) | 0% (0) | 89.3% (25) | 3.6% (1) | 95.5% (21) | 0% (0) | 100% (1) | 0% (0) | 91.8% (45) | 2% (1) |
|  |  |  | HHb ® ABP | 89.5% (34) | 2.6% (1) | 91.7% (11) | 0% (0) | 89.3% (25) | 3.6% (1) | 90.9% (20) | 0% (0) | 100% (1) | 0% (0) | 89.8% (44) | 2% (1) |
|  |  | Right | ABP ® HHb | 78.9% (30) | 7.9% (3) | 91.7% (11) | 8.3% (1) | 71.4% (20) | 14.3% (4) | 95.5% (21) | 0% (0) | 0% (0) | 0% (0) | 83.7% (41) | 8.2% (4) |
|  |  |  | HHb ® ABP | 81.6% (31) | 7.9% (3) | 91.7% (11) | 8.3% (1) | 75% (21) | 14.3% (4) | 95.5% (21) | 0% (0) | 0% (0) | 0% (0) | 85.7% (42) | 8.2% (4) |
| ABP & tHb | Frontal | Left | ABP ® tHb | 89.5% (34) | 5.3% (2) | 91.7% (11) | 0% (0) | 89.3% (25) | 3.6% (1) | 90.9% (20) | 4.5% (1) | 100% (1) | 0% (0) | 89.8% (44) | 4.1% (2) |
|  |  |  | tHb ® ABP | 92.1% (35) | 5.3% (2) | 91.7% (11) | 0% (0) | 92.9% (26) | 3.6% (1) | 90.9% (20) | 4.5% (1) | 100% (1) | 0% (0) | 91.8% (45) | 4.1% (2) |
|  |  | Right | ABP ® tHb | 78.9% (30) | 2.6% (1) | 91.7% (11) | 0% (0) | 78.6% (22) | 3.6% (1) | 86.4% (19) | 0% (0) | 100% (1) | 0% (0) | 81.6% (40) | 2% (1) |
|  |  |  | tHb ® ABP | 84.2% (32) | 2.6% (1) | 91.7% (11) | 0% (0) | 85.7% (24) | 3.6% (1) | 86.4% (19) | 0% (0) | 100% (1) | 0% (0) | 85.7% (42) | 2% (1) |
|  | Parietal | Left | ABP ® tHb | 84.2% (32) | 2.6% (1) | 91.7% (11) | 0% (0) | 85.7% (24) | 0% (0) | 86.4% (19) | 4.5% (1) | 100% (1) | 0% (0) | 85.7% (42) | 2% (1) |
|  |  |  | tHb ® ABP | 81.6% (31) | 2.6% (1) | 100% (12) | 0% (0) | 92.9% (26) | 0% (0) | 77.3% (17) | 4.5% (1) | 100% (1) | 0% (0) | 85.7% (42) | 2% (1) |
|  |  | Right | ABP ® tHb | 86.8% (33) | 5.3% (2) | 100% (12) | 0% (0) | 89.3% (25) | 7.1% (2) | 90.9% (20) | 0% (0) | 100% (1) | 0% (0) | 89.8% (44) | 4.1% (2) |
|  |  |  | tHb ® ABP | 86.8% (33) | 5.3% (2) | 100% (12) | 0% (0) | 89.3% (25) | 7.1% (2) | 90.9% (20) | 0% (0) | 100% (1) | 0% (0) | 89.8% (44) | 4.1% (2) |
|  | Occipital | Left | ABP ® tHb | 92.1% (35) | 2.6% (1) | 91.7% (11) | 0% (0) | 89.3% (25) | 3.6% (1) | 95.5% (21) | 0% (0) | 100% (1) | 0% (0) | 91.8% (45) | 2% (1) |
|  |  |  | tHb ® ABP | 92.1% (35) | 2.6% (1) | 91.7% (11) | 0% (0) | 89.3% (25) | 3.6% (1) | 95.5% (21) | 0% (0) | 100% (1) | 0% (0) | 91.8% (45) | 2% (1) |
|  |  | Right | ABP ® tHb | 84.2% (32) | 0% (0) | 91.7% (11) | 8.3% (1) | 82.1% (23) | 0% (0) | 90.9% (20) | 4.5% (1) | 100% (1) | 0% (0) | 85.7% (42) | 2% (1) |
|  |  |  | tHb ® ABP | 84.2% (32) | 0% (0) | 91.7% (11) | 8.3% (1) | 82.1% (23) | 0% (0) | 90.9% (20) | 4.5% (1) | 100% (1) | 0% (0) | 85.7% (42) | 2% (1) |
|  | Temporal | Left | ABP ® tHb | 84.2% (32) | 0% (0) | 100% (12) | 0% (0) | 85.7% (24) | 0% (0) | 90.9% (20) | 0% (0) | 100% (1) | 0% (0) | 87.8% (43) | 0% (0) |
|  |  |  | tHb ® ABP | 81.6% (31) | 0% (0) | 91.7% (11) | 0% (0) | 82.1% (23) | 0% (0) | 86.4% (19) | 0% (0) | 100% (1) | 0% (0) | 83.7% (41) | 0% (0) |
|  |  | Right | ABP ® tHb | 94.7% (36) | 0% (0) | 91.7% (11) | 0% (0) | 96.4% (27) | 0% (0) | 90.9% (20) | 0% (0) | 100% (1) | 0% (0) | 93.9% (46) | 0% (0) |
|  |  |  | tHb ® ABP | 89.5% (34) | 0% (0) | 91.7% (11) | 0% (0) | 89.3% (25) | 0% (0) | 90.9% (20) | 0% (0) | 100% (1) | 0% (0) | 89.8% (44) | 0% (0) |
| ABP & HbDiff | Frontal | Left | ABP ® HbDiff | 94.7% (36) | 2.6% (1) | 100% (12) | 0% (0) | 96.4% (27) | 0% (0) | 95.5% (21) | 4.5% (1) | 100% (1) | 0% (0) | 95.9% (47) | 2% (1) |
|  |  |  | HbDiff ® ABP | 94.7% (36) | 2.6% (1) | 100% (12) | 0% (0) | 96.4% (27) | 0% (0) | 95.5% (21) | 4.5% (1) | 100% (1) | 0% (0) | 95.9% (47) | 2% (1) |
|  |  | Right | ABP ® HbDiff | 92.1% (35) | 2.6% (1) | 75% (9) | 8.3% (1) | 89.3% (25) | 0% (0) | 86.4% (19) | 9.1% (2) | 100% (1) | 0% (0) | 87.8% (43) | 4.1% (2) |
|  |  |  | HbDiff ® ABP | 94.7% (36) | 2.6% (1) | 75% (9) | 8.3% (1) | 89.3% (25) | 0% (0) | 90.9% (20) | 9.1% (2) | 100% (1) | 0% (0) | 89.8% (44) | 4.1% (2) |
|  | Parietal | Left | ABP ® HbDiff | 86.8% (33) | 0% (0) | 100% (12) | 0% (0) | 85.7% (24) | 0% (0) | 95.5% (21) | 0% (0) | 0% (0) | 0% (0) | 91.8% (45) | 0% (0) |
|  |  |  | HbDiff ® ABP | 86.8% (33) | 0% (0) | 100% (12) | 0% (0) | 89.3% (25) | 0% (0) | 90.9% (20) | 0% (0) | 0% (0) | 0% (0) | 91.8% (45) | 0% (0) |
|  |  | Right | ABP ® HbDiff | 89.5% (34) | 7.9% (3) | 100% (12) | 0% (0) | 89.3% (25) | 7.1% (2) | 95.5% (21) | 4.5% (1) | 100% (1) | 0% (0) | 91.8% (45) | 6.1% (3) |
|  |  |  | HbDiff ® ABP | 89.5% (34) | 7.9% (3) | 100% (12) | 0% (0) | 89.3% (25) | 7.1% (2) | 95.5% (21) | 4.5% (1) | 100% (1) | 0% (0) | 91.8% (45) | 6.1% (3) |
|  | Occipital | Left | ABP ® HbDiff | 89.5% (34) | 2.6% (1) | 75% (9) | 8.3% (1) | 85.7% (24) | 0% (0) | 86.4% (19) | 9.1% (2) | 100% (1) | 0% (0) | 85.7% (42) | 4.1% (2) |
|  |  |  | HbDiff ® ABP | 86.8% (33) | 2.6% (1) | 91.7% (11) | 8.3% (1) | 89.3% (25) | 0% (0) | 86.4% (19) | 9.1% (2) | 100% (1) | 0% (0) | 87.8% (43) | 4.1% (2) |
|  |  | Right | ABP ® HbDiff | 84.2% (32) | 2.6% (1) | 91.7% (11) | 8.3% (1) | 82.1% (23) | 7.1% (2) | 90.9% (20) | 0% (0) | 0% (0) | 100% (1) | 87.8% (43) | 2% (1) |
|  |  |  | HbDiff ® ABP | 84.2% (32) | 2.6% (1) | 91.7% (11) | 8.3% (1) | 82.1% (23) | 7.1% (2) | 90.9% (20) | 0% (0) | 0% (0) | 100% (1) | 87.8% (43) | 2% (1) |
|  | Temporal | Left | ABP ® HbDiff | 89.5% (34) | 2.6% (1) | 100% (12) | 0% (0) | 92.9% (26) | 0% (0) | 90.9% (20) | 4.5% (1) | 100% (1) | 0% (0) | 91.8% (45) | 2% (1) |
|  |  |  | HbDiff ® ABP | 89.5% (34) | 2.6% (1) | 100% (12) | 0% (0) | 92.9% (26) | 0% (0) | 90.9% (20) | 4.5% (1) | 100% (1) | 0% (0) | 91.8% (45) | 2% (1) |
|  |  | Right | ABP ® HbDiff | 92.1% (35) | 2.6% (1) | 91.7% (11) | 0% (0) | 85.7% (24) | 3.6% (1) | 100% (22) | 0% (0) | 100% (1) | 0% (0) | 91.8% (45) | 2% (1) |
|  |  |  | HbDiff ® ABP | 92.1% (35) | 2.6% (1) | 91.7% (11) | 0% (0) | 85.7% (24) | 3.6% (1) | 100% (22) | 0% (0) | 100% (1) | 0% (0) | 91.8% (45) | 2% (1) |
| **250 Hz Sampled Data** | | | | | | | | | | | | | | | |
| ABP & rSO_2_ | Frontal | Left | ABP ® rSO_2_ | 84.2% (32) | 0% (0) | 75% (9) | 0% (0) | 85.7% (24) | 0% (0) | 77.3% (17) | 0% (0) | 100% (1) | 0% (0) | 81.6% (40) | 0% (0) |
|  |  |  | rSO_2_ ® ABP | 84.2% (32) | 0% (0) | 83.3% (10) | 0% (0) | 85.7% (24) | 0% (0) | 81.8% (18) | 0% (0) | 100% (1) | 0% (0) | 83.7% (41) | 0% (0) |
|  |  | Right | ABP ® rSO_2_ | 94.7% (36) | 2.6% (1) | 100% (12) | 0% (0) | 92.9% (26) | 3.6% (1) | 100% (22) | 0% (0) | 100% (1) | 0% (0) | 95.9% (47) | 2% (1) |
|  |  |  | rSO_2_ ® ABP | 97.4% (37) | 2.6% (1) | 100% (12) | 0% (0) | 96.4% (27) | 3.6% (1) | 100% (22) | 0% (0) | 100% (1) | 0% (0) | 98% (48) | 2% (1) |
|  | Parietal | Left | ABP ® rSO_2_ | 86.8% (33) | 2.6% (1) | 91.7% (11) | 0% (0) | 85.7% (24) | 0% (0) | 90.9% (20) | 4.5% (1) | 100% (1) | 0% (0) | 87.8% (43) | 2% (1) |
|  |  |  | rSO_2_ ® ABP | 89.5% (34) | 2.6% (1) | 91.7% (11) | 0% (0) | 89.3% (25) | 0% (0) | 90.9% (20) | 4.5% (1) | 100% (1) | 0% (0) | 89.8% (44) | 2% (1) |
|  |  | Right | ABP ® rSO_2_ | 89.5% (34) | 2.6% (1) | 91.7% (11) | 8.3% (1) | 89.3% (25) | 3.6% (1) | 90.9% (20) | 4.5% (1) | 100% (1) | 0% (0) | 89.8% (44) | 4.1% (2) |
|  |  |  | rSO_2_ ® ABP | 89.5% (34) | 2.6% (1) | 91.7% (11) | 8.3% (1) | 89.3% (25) | 3.6% (1) | 90.9% (20) | 4.5% (1) | 100% (1) | 0% (0) | 89.8% (44) | 4.1% (2) |
|  | Occipital | Left | ABP ® rSO_2_ | 97.4% (37) | 2.6% (1) | 91.7% (11) | 0% (0) | 96.4% (27) | 3.6% (1) | 95.5% (21) | 0% (0) | 100% (1) | 0% (0) | 95.9% (47) | 2% (1) |
|  |  |  | rSO_2_ ® ABP | 97.4% (37) | 2.6% (1) | 83.3% (10) | 0% (0) | 96.4% (27) | 3.6% (1) | 90.9% (20) | 0% (0) | 100% (1) | 0% (0) | 93.9% (46) | 2% (1) |
|  |  | Right | ABP ® rSO_2_ | 84.2% (32) | 2.6% (1) | 100% (12) | 0% (0) | 96.4% (27) | 0% (0) | 77.3% (17) | 4.5% (1) | 100% (1) | 0% (0) | 87.8% (43) | 2% (1) |
|  |  |  | rSO_2_ ® ABP | 84.2% (32) | 2.6% (1) | 100% (12) | 0% (0) | 96.4% (27) | 0% (0) | 77.3% (17) | 4.5% (1) | 100% (1) | 0% (0) | 87.8% (43) | 2% (1) |
|  | Temporal | Left | ABP ® rSO_2_ | 84.2% (32) | 0% (0) | 91.7% (11) | 0% (0) | 89.3% (25) | 0% (0) | 81.8% (18) | 0% (0) | 100% (1) | 0% (0) | 85.7% (42) | 0% (0) |
|  |  |  | rSO_2_ ® ABP | 84.2% (32) | 0% (0) | 91.7% (11) | 0% (0) | 92.9% (26) | 0% (0) | 77.3% (17) | 0% (0) | 100% (1) | 0% (0) | 85.7% (42) | 0% (0) |
|  |  | Right | ABP ® rSO_2_ | 97.4% (37) | 0% (0) | 83.3% (10) | 16.7% (2) | 92.9% (26) | 3.6% (1) | 95.5% (21) | 4.5% (1) | 100% (1) | 0% (0) | 93.9% (46) | 4.1% (2) |
|  |  |  | rSO_2_ ® ABP | 94.7% (36) | 0% (0) | 83.3% (10) | 16.7% (2) | 92.9% (26) | 3.6% (1) | 90.9% (20) | 4.5% (1) | 100% (1) | 0% (0) | 91.8% (45) | 4.1% (2) |
| ABP & HbO | Frontal | Left | ABP ® HbO | 97.4% (37) | 0% (0) | 100% (12) | 0% (0) | 100% (28) | 0% (0) | 95.5% (21) | 0% (0) | 100% (1) | 0% (0) | 98% (48) | 0% (0) |
|  |  |  | HbO ® ABP | 97.4% (37) | 0% (0) | 100% (12) | 0% (0) | 100% (28) | 0% (0) | 95.5% (21) | 0% (0) | 100% (1) | 0% (0) | 98% (48) | 0% (0) |
|  |  | Right | ABP ® HbO | 92.1% (35) | 0% (0) | 100% (12) | 0% (0) | 96.4% (27) | 0% (0) | 90.9% (20) | 0% (0) | 100% (1) | 0% (0) | 93.9% (46) | 0% (0) |
|  |  |  | HbO ® ABP | 89.5% (34) | 0% (0) | 100% (12) | 0% (0) | 92.9% (26) | 0% (0) | 90.9% (20) | 0% (0) | 100% (1) | 0% (0) | 91.8% (45) | 0% (0) |
|  | Parietal | Left | ABP ® HbO | 84.2% (32) | 2.6% (1) | 91.7% (11) | 0% (0) | 85.7% (24) | 0% (0) | 86.4% (19) | 4.5% (1) | 0% (0) | 0% (0) | 87.8% (43) | 2% (1) |
|  |  |  | HbO ® ABP | 81.6% (31) | 2.6% (1) | 91.7% (11) | 0% (0) | 82.1% (23) | 0% (0) | 86.4% (19) | 4.5% (1) | 0% (0) | 0% (0) | 85.7% (42) | 2% (1) |
|  |  | Right | ABP ® HbO | 94.7% (36) | 2.6% (1) | 100% (12) | 0% (0) | 92.9% (26) | 3.6% (1) | 100% (22) | 0% (0) | 100% (1) | 0% (0) | 95.9% (47) | 2% (1) |
|  |  |  | HbO ® ABP | 94.7% (36) | 2.6% (1) | 100% (12) | 0% (0) | 92.9% (26) | 3.6% (1) | 100% (22) | 0% (0) | 100% (1) | 0% (0) | 95.9% (47) | 2% (1) |
|  | Occipital | Left | ABP ® HbO | 84.2% (32) | 0% (0) | 83.3% (10) | 0% (0) | 82.1% (23) | 0% (0) | 86.4% (19) | 0% (0) | 100% (1) | 0% (0) | 83.7% (41) | 0% (0) |
|  |  |  | HbO ® ABP | 86.8% (33) | 0% (0) | 91.7% (11) | 0% (0) | 85.7% (24) | 0% (0) | 90.9% (20) | 0% (0) | 100% (1) | 0% (0) | 87.8% (43) | 0% (0) |
|  |  | Right | ABP ® HbO | 86.8% (33) | 2.6% (1) | 91.7% (11) | 0% (0) | 96.4% (27) | 0% (0) | 77.3% (17) | 4.5% (1) | 100% (1) | 0% (0) | 87.8% (43) | 2% (1) |
|  |  |  | HbO ® ABP | 89.5% (34) | 2.6% (1) | 91.7% (11) | 0% (0) | 100% (28) | 0% (0) | 77.3% (17) | 4.5% (1) | 100% (1) | 0% (0) | 89.8% (44) | 2% (1) |
|  | Temporal | Left | ABP ® HbO | 81.6% (31) | 2.6% (1) | 83.3% (10) | 0% (0) | 82.1% (23) | 3.6% (1) | 81.8% (18) | 0% (0) | 100% (1) | 0% (0) | 81.6% (40) | 2% (1) |
|  |  |  | HbO ® ABP | 81.6% (31) | 2.6% (1) | 83.3% (10) | 0% (0) | 82.1% (23) | 3.6% (1) | 81.8% (18) | 0% (0) | 100% (1) | 0% (0) | 81.6% (40) | 2% (1) |
|  |  | Right | ABP ® HbO | 94.7% (36) | 2.6% (1) | 100% (12) | 0% (0) | 96.4% (27) | 0% (0) | 95.5% (21) | 4.5% (1) | 100% (1) | 0% (0) | 95.9% (47) | 2% (1) |
|  |  |  | HbO ® ABP | 97.4% (37) | 2.6% (1) | 100% (12) | 0% (0) | 100% (28) | 0% (0) | 95.5% (21) | 4.5% (1) | 100% (1) | 0% (0) | 98% (48) | 2% (1) |
| ABP & HHb | Frontal | Left | ABP ® HHb | 78.9% (30) | 7.9% (3) | 100% (12) | 0% (0) | 78.6% (22) | 7.1% (2) | 90.9% (20) | 4.5% (1) | 100% (1) | 0% (0) | 83.7% (41) | 6.1% (3) |
|  |  |  | HHb ® ABP | 78.9% (30) | 7.9% (3) | 100% (12) | 0% (0) | 78.6% (22) | 7.1% (2) | 90.9% (20) | 4.5% (1) | 100% (1) | 0% (0) | 83.7% (41) | 6.1% (3) |
|  |  | Right | ABP ® HHb | 81.6% (31) | 2.6% (1) | 91.7% (11) | 0% (0) | 85.7% (24) | 0% (0) | 81.8% (18) | 4.5% (1) | 100% (1) | 0% (0) | 83.7% (41) | 2% (1) |
|  |  |  | HHb ® ABP | 81.6% (31) | 2.6% (1) | 91.7% (11) | 0% (0) | 85.7% (24) | 0% (0) | 81.8% (18) | 4.5% (1) | 100% (1) | 0% (0) | 83.7% (41) | 2% (1) |
|  | Parietal | Left | ABP ® HHb | 92.1% (35) | 0% (0) | 100% (12) | 0% (0) | 89.3% (25) | 0% (0) | 100% (22) | 0% (0) | 100% (1) | 0% (0) | 93.9% (46) | 0% (0) |
|  |  |  | HHb ® ABP | 94.7% (36) | 0% (0) | 100% (12) | 0% (0) | 92.9% (26) | 0% (0) | 100% (22) | 0% (0) | 100% (1) | 0% (0) | 95.9% (47) | 0% (0) |
|  |  | Right | ABP ® HHb | 94.7% (36) | 0% (0) | 91.7% (11) | 0% (0) | 92.9% (26) | 0% (0) | 95.5% (21) | 0% (0) | 100% (1) | 0% (0) | 93.9% (46) | 0% (0) |
|  |  |  | HHb ® ABP | 94.7% (36) | 0% (0) | 91.7% (11) | 0% (0) | 92.9% (26) | 0% (0) | 95.5% (21) | 0% (0) | 100% (1) | 0% (0) | 93.9% (46) | 0% (0) |
|  | Occipital | Left | ABP ® HHb | 89.5% (34) | 0% (0) | 66.7% (8) | 0% (0) | 85.7% (24) | 0% (0) | 81.8% (18) | 0% (0) | 100% (1) | 0% (0) | 83.7% (41) | 0% (0) |
|  |  |  | HHb ® ABP | 92.1% (35) | 0% (0) | 66.7% (8) | 0% (0) | 89.3% (25) | 0% (0) | 81.8% (18) | 0% (0) | 100% (1) | 0% (0) | 85.7% (42) | 0% (0) |
|  |  | Right | ABP ® HHb | 94.7% (36) | 0% (0) | 100% (12) | 0% (0) | 96.4% (27) | 0% (0) | 95.5% (21) | 0% (0) | 100% (1) | 0% (0) | 95.9% (47) | 0% (0) |
|  |  |  | HHb ® ABP | 94.7% (36) | 0% (0) | 100% (12) | 0% (0) | 96.4% (27) | 0% (0) | 95.5% (21) | 0% (0) | 100% (1) | 0% (0) | 95.9% (47) | 0% (0) |
|  | Temporal | Left | ABP ® HHb | 81.6% (31) | 5.3% (2) | 75% (9) | 8.3% (1) | 78.6% (22) | 7.1% (2) | 81.8% (18) | 4.5% (1) | 100% (1) | 0% (0) | 79.6% (39) | 6.1% (3) |
|  |  |  | HHb ® ABP | 81.6% (31) | 5.3% (2) | 66.7% (8) | 8.3% (1) | 78.6% (22) | 7.1% (2) | 77.3% (17) | 4.5% (1) | 100% (1) | 0% (0) | 77.6% (38) | 6.1% (3) |
|  |  | Right | ABP ® HHb | 76.3% (29) | 5.3% (2) | 83.3% (10) | 8.3% (1) | 78.6% (22) | 3.6% (1) | 77.3% (17) | 9.1% (2) | 100% (1) | 0% (0) | 77.6% (38) | 6.1% (3) |
|  |  |  | HHb ® ABP | 78.9% (30) | 5.3% (2) | 83.3% (10) | 8.3% (1) | 82.1% (23) | 3.6% (1) | 77.3% (17) | 9.1% (2) | 100% (1) | 0% (0) | 79.6% (39) | 6.1% (3) |
| ABP & tHb | Frontal | Left | ABP ® tHb | 84.2% (32) | 0% (0) | 100% (12) | 0% (0) | 85.7% (24) | 0% (0) | 90.9% (20) | 0% (0) | 0% (0) | 0% (0) | 89.8% (44) | 0% (0) |
|  |  |  | tHb ® ABP | 84.2% (32) | 0% (0) | 100% (12) | 0% (0) | 85.7% (24) | 0% (0) | 90.9% (20) | 0% (0) | 0% (0) | 0% (0) | 89.8% (44) | 0% (0) |
|  |  | Right | ABP ® tHb | 89.5% (34) | 0% (0) | 100% (12) | 0% (0) | 89.3% (25) | 0% (0) | 95.5% (21) | 0% (0) | 100% (1) | 0% (0) | 91.8% (45) | 0% (0) |
|  |  |  | tHb ® ABP | 89.5% (34) | 0% (0) | 100% (12) | 0% (0) | 89.3% (25) | 0% (0) | 95.5% (21) | 0% (0) | 100% (1) | 0% (0) | 91.8% (45) | 0% (0) |
|  | Parietal | Left | ABP ® tHb | 86.8% (33) | 0% (0) | 91.7% (11) | 0% (0) | 92.9% (26) | 0% (0) | 81.8% (18) | 0% (0) | 100% (1) | 0% (0) | 87.8% (43) | 0% (0) |
|  |  |  | tHb ® ABP | 86.8% (33) | 0% (0) | 91.7% (11) | 0% (0) | 92.9% (26) | 0% (0) | 81.8% (18) | 0% (0) | 100% (1) | 0% (0) | 87.8% (43) | 0% (0) |
|  |  | Right | ABP ® tHb | 94.7% (36) | 0% (0) | 83.3% (10) | 0% (0) | 92.9% (26) | 0% (0) | 90.9% (20) | 0% (0) | 100% (1) | 0% (0) | 91.8% (45) | 0% (0) |
|  |  |  | tHb ® ABP | 94.7% (36) | 0% (0) | 83.3% (10) | 0% (0) | 92.9% (26) | 0% (0) | 90.9% (20) | 0% (0) | 100% (1) | 0% (0) | 91.8% (45) | 0% (0) |
|  | Occipital | Left | ABP ® tHb | 89.5% (34) | 5.3% (2) | 91.7% (11) | 0% (0) | 89.3% (25) | 3.6% (1) | 90.9% (20) | 4.5% (1) | 100% (1) | 0% (0) | 89.8% (44) | 4.1% (2) |
|  |  |  | tHb ® ABP | 86.8% (33) | 5.3% (2) | 91.7% (11) | 0% (0) | 89.3% (25) | 3.6% (1) | 86.4% (19) | 4.5% (1) | 100% (1) | 0% (0) | 87.8% (43) | 4.1% (2) |
|  |  | Right | ABP ® tHb | 89.5% (34) | 0% (0) | 91.7% (11) | 0% (0) | 89.3% (25) | 0% (0) | 90.9% (20) | 0% (0) | 100% (1) | 0% (0) | 89.8% (44) | 0% (0) |
|  |  |  | tHb ® ABP | 94.7% (36) | 0% (0) | 91.7% (11) | 0% (0) | 92.9% (26) | 0% (0) | 95.5% (21) | 0% (0) | 100% (1) | 0% (0) | 93.9% (46) | 0% (0) |
|  | Temporal | Left | ABP ® tHb | 89.5% (34) | 5.3% (2) | 75% (9) | 8.3% (1) | 89.3% (25) | 7.1% (2) | 81.8% (18) | 4.5% (1) | 100% (1) | 0% (0) | 85.7% (42) | 6.1% (3) |
|  |  |  | tHb ® ABP | 86.8% (33) | 5.3% (2) | 83.3% (10) | 8.3% (1) | 85.7% (24) | 7.1% (2) | 86.4% (19) | 4.5% (1) | 100% (1) | 0% (0) | 85.7% (42) | 6.1% (3) |
|  |  | Right | ABP ® tHb | 89.5% (34) | 0% (0) | 100% (12) | 0% (0) | 89.3% (25) | 0% (0) | 95.5% (21) | 0% (0) | 100% (1) | 0% (0) | 91.8% (45) | 0% (0) |
|  |  |  | tHb ® ABP | 92.1% (35) | 0% (0) | 91.7% (11) | 0% (0) | 92.9% (26) | 0% (0) | 90.9% (20) | 0% (0) | 100% (1) | 0% (0) | 91.8% (45) | 0% (0) |
| ABP & HbDiff | Frontal | Left | ABP ® HbDiff | 86.8% (33) | 2.6% (1) | 100% (12) | 0% (0) | 89.3% (25) | 3.6% (1) | 90.9% (20) | 0% (0) | 100% (1) | 0% (0) | 89.8% (44) | 2% (1) |
|  |  |  | HbDiff ® ABP | 89.5% (34) | 2.6% (1) | 100% (12) | 0% (0) | 92.9% (26) | 3.6% (1) | 90.9% (20) | 0% (0) | 100% (1) | 0% (0) | 91.8% (45) | 2% (1) |
|  |  | Right | ABP ® HbDiff | 94.7% (36) | 0% (0) | 100% (12) | 0% (0) | 92.9% (26) | 0% (0) | 100% (22) | 0% (0) | 100% (1) | 0% (0) | 95.9% (47) | 0% (0) |
|  |  |  | HbDiff ® ABP | 92.1% (35) | 0% (0) | 100% (12) | 0% (0) | 92.9% (26) | 0% (0) | 95.5% (21) | 0% (0) | 100% (1) | 0% (0) | 93.9% (46) | 0% (0) |
|  | Parietal | Left | ABP ® HbDiff | 86.8% (33) | 0% (0) | 83.3% (10) | 0% (0) | 78.6% (22) | 0% (0) | 95.5% (21) | 0% (0) | 100% (1) | 0% (0) | 85.7% (42) | 0% (0) |
|  |  |  | HbDiff ® ABP | 89.5% (34) | 0% (0) | 83.3% (10) | 0% (0) | 82.1% (23) | 0% (0) | 95.5% (21) | 0% (0) | 100% (1) | 0% (0) | 87.8% (43) | 0% (0) |
|  |  | Right | ABP ® HbDiff | 94.7% (36) | 0% (0) | 100% (12) | 0% (0) | 96.4% (27) | 0% (0) | 95.5% (21) | 0% (0) | 100% (1) | 0% (0) | 95.9% (47) | 0% (0) |
|  |  |  | HbDiff ® ABP | 94.7% (36) | 0% (0) | 100% (12) | 0% (0) | 96.4% (27) | 0% (0) | 95.5% (21) | 0% (0) | 100% (1) | 0% (0) | 95.9% (47) | 0% (0) |
|  | Occipital | Left | ABP ® HbDiff | 92.1% (35) | 2.6% (1) | 83.3% (10) | 0% (0) | 89.3% (25) | 3.6% (1) | 90.9% (20) | 0% (0) | 100% (1) | 0% (0) | 89.8% (44) | 2% (1) |
|  |  |  | HbDiff ® ABP | 92.1% (35) | 2.6% (1) | 83.3% (10) | 0% (0) | 85.7% (24) | 3.6% (1) | 95.5% (21) | 0% (0) | 100% (1) | 0% (0) | 89.8% (44) | 2% (1) |
|  |  | Right | ABP ® HbDiff | 92.1% (35) | 0% (0) | 100% (12) | 0% (0) | 96.4% (27) | 0% (0) | 90.9% (20) | 0% (0) | 100% (1) | 0% (0) | 93.9% (46) | 0% (0) |
|  |  |  | HbDiff ® ABP | 94.7% (36) | 0% (0) | 100% (12) | 0% (0) | 100% (28) | 0% (0) | 90.9% (20) | 0% (0) | 100% (1) | 0% (0) | 95.9% (47) | 0% (0) |
|  | Temporal | Left | ABP ® HbDiff | 86.8% (33) | 5.3% (2) | 91.7% (11) | 0% (0) | 92.9% (26) | 3.6% (1) | 81.8% (18) | 4.5% (1) | 100% (1) | 0% (0) | 87.8% (43) | 4.1% (2) |
|  |  |  | HbDiff ® ABP | 78.9% (30) | 5.3% (2) | 83.3% (10) | 0% (0) | 85.7% (24) | 3.6% (1) | 72.7% (16) | 4.5% (1) | 100% (1) | 0% (0) | 79.6% (39) | 4.1% (2) |
|  |  | Right | ABP ® HbDiff | 94.7% (36) | 0% (0) | 91.7% (11) | 0% (0) | 96.4% (27) | 0% (0) | 90.9% (20) | 0% (0) | 100% (1) | 0% (0) | 93.9% (46) | 0% (0) |
|  |  |  | HbDiff ® ABP | 94.7% (36) | 0% (0) | 91.7% (11) | 0% (0) | 96.4% (27) | 0% (0) | 90.9% (20) | 0% (0) | 100% (1) | 0% (0) | 93.9% (46) | 0% (0) |
| The table shows the hemispheric responsiveness of signals using subgrouped Impulse Response Coefficients of Optimal VARIMA model using 1 Hz and 250 Hz data. *ABP, arterial blood pressure; HbDiff, hemoglobin difference; HbO, oxyhemoglobin; HHb, deoxyhemoglobin; rSO_2_, regional cerebral oxygen saturation; tHb, total hemoglobin; VARIMA, vector autoregressive integrative moving average.* | | | | | | | | | | | | | | | |

Appendix S6l: Subgrouped Granger Causal Directionality Results Based on Greater F-Statistic

| **Signal Combination** | **Brain Lobe** | **Hemisphere** | **Direction** | **Directional Granger Causality [% (count)] for Subgroups** | | | | | |
| --- | --- | --- | --- | --- | --- | --- | --- | --- | --- |
|  |  |  |  | **Age < 40 [n=38]** | **Age 40 – 60 [n=12]** | **Males [n=28]** | **Females [n=22]** | **Left Hand Dominance [n=1]** | **Right Hand Dominance [n=49]** |
| **1 Hz Sampled Data** | | | | | | | | | |
| ABP & rSO_2_ | Frontal | Left | ABP ® rSO_2_ | 42.1% (16) | 50% (6) | 39.3% (11) | 50% (11) | 0% (0) | 44.9% (22) |
|  |  |  | rSO_2_ ® ABP | 57.9% (22) | 50% (6) | 60.7% (17) | 50% (11) | 100% (1) | 55.1% (27) |
|  |  | Right | ABP ® rSO_2_ | 50% (19) | 41.7% (5) | 46.4% (13) | 50% (11) | 0% (0) | 49% (24) |
|  |  |  | rSO_2_ ® ABP | 50% (19) | 58.3% (7) | 53.6% (15) | 50% (11) | 100% (1) | 51% (25) |
|  | Parietal | Left | ABP ® rSO_2_ | 44.7% (17) | 58.3% (7) | 42.9% (12) | 54.5% (12) | 100% (1) | 46.9% (23) |
|  |  |  | rSO_2_ ® ABP | 55.3% (21) | 41.7% (5) | 57.1% (16) | 45.5% (10) | 0% (0) | 53.1% (26) |
|  |  | Right | ABP ® rSO_2_ | 60.5% (23) | 66.7% (8) | 50% (14) | 77.3% (17) | 0% (0) | 63.3% (31) |
|  |  |  | rSO_2_ ® ABP | 39.5% (15) | 33.3% (4) | 50% (14) | 22.7% (5) | 100% (1) | 36.7% (18) |
|  | Occipital | Left | ABP ® rSO_2_ | 63.2% (24) | 58.3% (7) | 64.3% (18) | 59.1% (13) | 100% (1) | 61.2% (30) |
|  |  |  | rSO_2_ ® ABP | 36.8% (14) | 41.7% (5) | 35.7% (10) | 40.9% (9) | 0% (0) | 38.8% (19) |
|  |  | Right | ABP ® rSO_2_ | 42.1% (16) | 41.7% (5) | 46.4% (13) | 36.4% (8) | 100% (1) | 40.8% (20) |
|  |  |  | rSO_2_ ® ABP | 57.9% (22) | 58.3% (7) | 53.6% (15) | 63.6% (14) | 0% (0) | 59.2% (29) |
|  | Temporal | Left | ABP ® rSO_2_ | 44.7% (17) | 25% (3) | 42.9% (12) | 36.4% (8) | 0% (0) | 40.8% (20) |
|  |  |  | rSO_2_ ® ABP | 55.3% (21) | 75% (9) | 57.1% (16) | 63.6% (14) | 100% (1) | 59.2% (29) |
|  |  | Right | ABP ® rSO_2_ | 55.3% (21) | 50% (6) | 53.6% (15) | 54.5% (12) | 0% (0) | 55.1% (27) |
|  |  |  | rSO_2_ ® ABP | 44.7% (17) | 50% (6) | 46.4% (13) | 45.5% (10) | 100% (1) | 44.9% (22) |
| ABP & HbO | Frontal | Left | ABP ® HbO | 47.4% (18) | 33.3% (4) | 39.3% (11) | 50% (11) | 100% (1) | 42.9% (21) |
|  |  |  | HbO ® ABP | 52.6% (20) | 66.7% (8) | 60.7% (17) | 50% (11) | 0% (0) | 57.1% (28) |
|  |  | Right | ABP ® HbO | 68.4% (26) | 41.7% (5) | 60.7% (17) | 63.6% (14) | 100% (1) | 61.2% (30) |
|  |  |  | HbO ® ABP | 31.6% (12) | 58.3% (7) | 39.3% (11) | 36.4% (8) | 0% (0) | 38.8% (19) |
|  | Parietal | Left | ABP ® HbO | 52.6% (20) | 58.3% (7) | 39.3% (11) | 72.7% (16) | 0% (0) | 55.1% (27) |
|  |  |  | HbO ® ABP | 47.4% (18) | 41.7% (5) | 60.7% (17) | 27.3% (6) | 100% (1) | 44.9% (22) |
|  |  | Right | ABP ® HbO | 50% (19) | 66.7% (8) | 53.6% (15) | 54.5% (12) | 100% (1) | 53.1% (26) |
|  |  |  | HbO ® ABP | 50% (19) | 33.3% (4) | 46.4% (13) | 45.5% (10) | 0% (0) | 46.9% (23) |
|  | Occipital | Left | ABP ® HbO | 50% (19) | 66.7% (8) | 50% (14) | 59.1% (13) | 0% (0) | 55.1% (27) |
|  |  |  | HbO ® ABP | 50% (19) | 33.3% (4) | 50% (14) | 40.9% (9) | 100% (1) | 44.9% (22) |
|  |  | Right | ABP ® HbO | 52.6% (20) | 50% (6) | 50% (14) | 54.5% (12) | 0% (0) | 53.1% (26) |
|  |  |  | HbO ® ABP | 47.4% (18) | 50% (6) | 50% (14) | 45.5% (10) | 100% (1) | 46.9% (23) |
|  | Temporal | Left | ABP ® HbO | 50% (19) | 41.7% (5) | 50% (14) | 45.5% (10) | 0% (0) | 49% (24) |
|  |  |  | HbO ® ABP | 50% (19) | 58.3% (7) | 50% (14) | 54.5% (12) | 100% (1) | 51% (25) |
|  |  | Right | ABP ® HbO | 65.8% (25) | 41.7% (5) | 60.7% (17) | 59.1% (13) | 0% (0) | 61.2% (30) |
|  |  |  | HbO ® ABP | 34.2% (13) | 58.3% (7) | 39.3% (11) | 40.9% (9) | 100% (1) | 38.8% (19) |
| ABP & HHb | Frontal | Left | ABP ® HHb | 57.9% (22) | 33.3% (4) | 53.6% (15) | 50% (11) | 100% (1) | 51% (25) |
|  |  |  | HHb ® ABP | 42.1% (16) | 66.7% (8) | 46.4% (13) | 50% (11) | 0% (0) | 49% (24) |
|  |  | Right | ABP ® HHb | 57.9% (22) | 50% (6) | 57.1% (16) | 54.5% (12) | 100% (1) | 55.1% (27) |
|  |  |  | HHb ® ABP | 42.1% (16) | 50% (6) | 42.9% (12) | 45.5% (10) | 0% (0) | 44.9% (22) |
|  | Parietal | Left | ABP ® HHb | 50% (19) | 50% (6) | 46.4% (13) | 54.5% (12) | 100% (1) | 49% (24) |
|  |  |  | HHb ® ABP | 50% (19) | 50% (6) | 53.6% (15) | 45.5% (10) | 0% (0) | 51% (25) |
|  |  | Right | ABP ® HHb | 50% (19) | 66.7% (8) | 50% (14) | 59.1% (13) | 0% (0) | 55.1% (27) |
|  |  |  | HHb ® ABP | 50% (19) | 33.3% (4) | 50% (14) | 40.9% (9) | 100% (1) | 44.9% (22) |
|  | Occipital | Left | ABP ® HHb | 52.6% (20) | 58.3% (7) | 46.4% (13) | 63.6% (14) | 100% (1) | 53.1% (26) |
|  |  |  | HHb ® ABP | 47.4% (18) | 41.7% (5) | 53.6% (15) | 36.4% (8) | 0% (0) | 46.9% (23) |
|  |  | Right | ABP ® HHb | 65.8% (25) | 33.3% (4) | 64.3% (18) | 50% (11) | 100% (1) | 57.1% (28) |
|  |  |  | HHb ® ABP | 34.2% (13) | 66.7% (8) | 35.7% (10) | 50% (11) | 0% (0) | 42.9% (21) |
|  | Temporal | Left | ABP ® HHb | 52.6% (20) | 50% (6) | 57.1% (16) | 45.5% (10) | 0% (0) | 53.1% (26) |
|  |  |  | HHb ® ABP | 47.4% (18) | 50% (6) | 42.9% (12) | 54.5% (12) | 100% (1) | 46.9% (23) |
|  |  | Right | ABP ® HHb | 57.9% (22) | 58.3% (7) | 60.7% (17) | 54.5% (12) | 0% (0) | 59.2% (29) |
|  |  |  | HHb ® ABP | 42.1% (16) | 41.7% (5) | 39.3% (11) | 45.5% (10) | 100% (1) | 40.8% (20) |
| ABP & tHb | Frontal | Left | ABP ® tHb | 55.3% (21) | 33.3% (4) | 50% (14) | 50% (11) | 100% (1) | 49% (24) |
|  |  |  | tHb ® ABP | 44.7% (17) | 66.7% (8) | 50% (14) | 50% (11) | 0% (0) | 51% (25) |
|  |  | Right | ABP ® tHb | 39.5% (15) | 58.3% (7) | 39.3% (11) | 50% (11) | 0% (0) | 44.9% (22) |
|  |  |  | tHb ® ABP | 60.5% (23) | 41.7% (5) | 60.7% (17) | 50% (11) | 100% (1) | 55.1% (27) |
|  | Parietal | Left | ABP ® tHb | 42.1% (16) | 41.7% (5) | 46.4% (13) | 36.4% (8) | 0% (0) | 42.9% (21) |
|  |  |  | tHb ® ABP | 57.9% (22) | 58.3% (7) | 53.6% (15) | 63.6% (14) | 100% (1) | 57.1% (28) |
|  |  | Right | ABP ® tHb | 50% (19) | 50% (6) | 46.4% (13) | 54.5% (12) | 0% (0) | 51% (25) |
|  |  |  | tHb ® ABP | 50% (19) | 50% (6) | 53.6% (15) | 45.5% (10) | 100% (1) | 49% (24) |
|  | Occipital | Left | ABP ® tHb | 44.7% (17) | 58.3% (7) | 39.3% (11) | 59.1% (13) | 0% (0) | 49% (24) |
|  |  |  | tHb ® ABP | 55.3% (21) | 41.7% (5) | 60.7% (17) | 40.9% (9) | 100% (1) | 51% (25) |
|  |  | Right | ABP ® tHb | 52.6% (20) | 50% (6) | 57.1% (16) | 45.5% (10) | 0% (0) | 53.1% (26) |
|  |  |  | tHb ® ABP | 47.4% (18) | 50% (6) | 42.9% (12) | 54.5% (12) | 100% (1) | 46.9% (23) |
|  | Temporal | Left | ABP ® tHb | 47.4% (18) | 58.3% (7) | 50% (14) | 50% (11) | 0% (0) | 51% (25) |
|  |  |  | tHb ® ABP | 52.6% (20) | 41.7% (5) | 50% (14) | 50% (11) | 100% (1) | 49% (24) |
|  |  | Right | ABP ® tHb | 57.9% (22) | 58.3% (7) | 53.6% (15) | 63.6% (14) | 0% (0) | 59.2% (29) |
|  |  |  | tHb ® ABP | 42.1% (16) | 41.7% (5) | 46.4% (13) | 36.4% (8) | 100% (1) | 40.8% (20) |
| ABP & HbDiff | Frontal | Left | ABP ® HbDiff | 47.4% (18) | 41.7% (5) | 46.4% (13) | 45.5% (10) | 100% (1) | 44.9% (22) |
|  |  |  | HbDiff ® ABP | 52.6% (20) | 58.3% (7) | 53.6% (15) | 54.5% (12) | 0% (0) | 55.1% (27) |
|  |  | Right | ABP ® HbDiff | 50% (19) | 33.3% (4) | 46.4% (13) | 45.5% (10) | 100% (1) | 44.9% (22) |
|  |  |  | HbDiff ® ABP | 50% (19) | 66.7% (8) | 53.6% (15) | 54.5% (12) | 0% (0) | 55.1% (27) |
|  | Parietal | Left | ABP ® HbDiff | 50% (19) | 66.7% (8) | 53.6% (15) | 54.5% (12) | 100% (1) | 53.1% (26) |
|  |  |  | HbDiff ® ABP | 50% (19) | 33.3% (4) | 46.4% (13) | 45.5% (10) | 0% (0) | 46.9% (23) |
|  |  | Right | ABP ® HbDiff | 63.2% (24) | 66.7% (8) | 53.6% (15) | 77.3% (17) | 0% (0) | 65.3% (32) |
|  |  |  | HbDiff ® ABP | 36.8% (14) | 33.3% (4) | 46.4% (13) | 22.7% (5) | 100% (1) | 34.7% (17) |
|  | Occipital | Left | ABP ® HbDiff | 44.7% (17) | 58.3% (7) | 39.3% (11) | 59.1% (13) | 100% (1) | 46.9% (23) |
|  |  |  | HbDiff ® ABP | 55.3% (21) | 41.7% (5) | 60.7% (17) | 40.9% (9) | 0% (0) | 53.1% (26) |
|  |  | Right | ABP ® HbDiff | 44.7% (17) | 58.3% (7) | 50% (14) | 45.5% (10) | 100% (1) | 46.9% (23) |
|  |  |  | HbDiff ® ABP | 55.3% (21) | 41.7% (5) | 50% (14) | 54.5% (12) | 0% (0) | 53.1% (26) |
|  | Temporal | Left | ABP ® HbDiff | 52.6% (20) | 25% (3) | 50% (14) | 40.9% (9) | 0% (0) | 46.9% (23) |
|  |  |  | HbDiff ® ABP | 47.4% (18) | 75% (9) | 50% (14) | 59.1% (13) | 100% (1) | 53.1% (26) |
|  |  | Right | ABP ® HbDiff | 50% (19) | 50% (6) | 50% (14) | 50% (11) | 0% (0) | 51% (25) |
|  |  |  | HbDiff ® ABP | 50% (19) | 50% (6) | 50% (14) | 50% (11) | 100% (1) | 49% (24) |
| **250 Hz Sampled Data** | | | | | | | | | |
| ABP & rSO_2_ | Frontal | Left | ABP ® rSO_2_ | 60.5% (23) | 50% (6) | 60.7% (17) | 54.5% (12) | 0% (0) | 59.2% (29) |
|  |  |  | rSO_2_ ® ABP | 39.5% (15) | 50% (6) | 39.3% (11) | 45.5% (10) | 100% (1) | 40.8% (20) |
|  |  | Right | ABP ® rSO_2_ | 52.6% (20) | 58.3% (7) | 50% (14) | 59.1% (13) | 0% (0) | 55.1% (27) |
|  |  |  | rSO_2_ ® ABP | 47.4% (18) | 41.7% (5) | 50% (14) | 40.9% (9) | 100% (1) | 44.9% (22) |
|  | Parietal | Left | ABP ® rSO_2_ | 50% (19) | 66.7% (8) | 46.4% (13) | 63.6% (14) | 0% (0) | 55.1% (27) |
|  |  |  | rSO_2_ ® ABP | 50% (19) | 33.3% (4) | 53.6% (15) | 36.4% (8) | 100% (1) | 44.9% (22) |
|  |  | Right | ABP ® rSO_2_ | 63.2% (24) | 50% (6) | 53.6% (15) | 68.2% (15) | 0% (0) | 61.2% (30) |
|  |  |  | rSO_2_ ® ABP | 36.8% (14) | 50% (6) | 46.4% (13) | 31.8% (7) | 100% (1) | 38.8% (19) |
|  | Occipital | Left | ABP ® rSO_2_ | 57.9% (22) | 58.3% (7) | 57.1% (16) | 59.1% (13) | 100% (1) | 57.1% (28) |
|  |  |  | rSO_2_ ® ABP | 42.1% (16) | 41.7% (5) | 42.9% (12) | 40.9% (9) | 0% (0) | 42.9% (21) |
|  |  | Right | ABP ® rSO_2_ | 55.3% (21) | 66.7% (8) | 53.6% (15) | 63.6% (14) | 100% (1) | 57.1% (28) |
|  |  |  | rSO_2_ ® ABP | 44.7% (17) | 33.3% (4) | 46.4% (13) | 36.4% (8) | 0% (0) | 42.9% (21) |
|  | Temporal | Left | ABP ® rSO_2_ | 52.6% (20) | 25% (3) | 50% (14) | 40.9% (9) | 0% (0) | 46.9% (23) |
|  |  |  | rSO_2_ ® ABP | 47.4% (18) | 75% (9) | 50% (14) | 59.1% (13) | 100% (1) | 53.1% (26) |
|  |  | Right | ABP ® rSO_2_ | 57.9% (22) | 50% (6) | 60.7% (17) | 50% (11) | 0% (0) | 57.1% (28) |
|  |  |  | rSO_2_ ® ABP | 42.1% (16) | 50% (6) | 39.3% (11) | 50% (11) | 100% (1) | 42.9% (21) |
| ABP & HbO | Frontal | Left | ABP ® HbO | 63.2% (24) | 50% (6) | 60.7% (17) | 59.1% (13) | 0% (0) | 61.2% (30) |
|  |  |  | HbO ® ABP | 36.8% (14) | 50% (6) | 39.3% (11) | 40.9% (9) | 100% (1) | 38.8% (19) |
|  |  | Right | ABP ® HbO | 60.5% (23) | 50% (6) | 53.6% (15) | 63.6% (14) | 0% (0) | 59.2% (29) |
|  |  |  | HbO ® ABP | 39.5% (15) | 50% (6) | 46.4% (13) | 36.4% (8) | 100% (1) | 40.8% (20) |
|  | Parietal | Left | ABP ® HbO | 63.2% (24) | 75% (9) | 57.1% (16) | 77.3% (17) | 0% (0) | 67.3% (33) |
|  |  |  | HbO ® ABP | 36.8% (14) | 25% (3) | 42.9% (12) | 22.7% (5) | 100% (1) | 32.7% (16) |
|  |  | Right | ABP ® HbO | 50% (19) | 66.7% (8) | 46.4% (13) | 63.6% (14) | 0% (0) | 55.1% (27) |
|  |  |  | HbO ® ABP | 50% (19) | 33.3% (4) | 53.6% (15) | 36.4% (8) | 100% (1) | 44.9% (22) |
|  | Occipital | Left | ABP ® HbO | 39.5% (15) | 58.3% (7) | 42.9% (12) | 45.5% (10) | 0% (0) | 44.9% (22) |
|  |  |  | HbO ® ABP | 60.5% (23) | 41.7% (5) | 57.1% (16) | 54.5% (12) | 100% (1) | 55.1% (27) |
|  |  | Right | ABP ® HbO | 57.9% (22) | 66.7% (8) | 53.6% (15) | 68.2% (15) | 0% (0) | 61.2% (30) |
|  |  |  | HbO ® ABP | 42.1% (16) | 33.3% (4) | 46.4% (13) | 31.8% (7) | 100% (1) | 38.8% (19) |
|  | Temporal | Left | ABP ® HbO | 57.9% (22) | 66.7% (8) | 64.3% (18) | 54.5% (12) | 100% (1) | 59.2% (29) |
|  |  |  | HbO ® ABP | 42.1% (16) | 33.3% (4) | 35.7% (10) | 45.5% (10) | 0% (0) | 40.8% (20) |
|  |  | Right | ABP ® HbO | 57.9% (22) | 41.7% (5) | 53.6% (15) | 54.5% (12) | 0% (0) | 55.1% (27) |
|  |  |  | HbO ® ABP | 42.1% (16) | 58.3% (7) | 46.4% (13) | 45.5% (10) | 100% (1) | 44.9% (22) |
| ABP & HHb | Frontal | Left | ABP ® HHb | 57.9% (22) | 41.7% (5) | 60.7% (17) | 45.5% (10) | 100% (1) | 53.1% (26) |
|  |  |  | HHb ® ABP | 42.1% (16) | 58.3% (7) | 39.3% (11) | 54.5% (12) | 0% (0) | 46.9% (23) |
|  |  | Right | ABP ® HHb | 60.5% (23) | 50% (6) | 57.1% (16) | 59.1% (13) | 0% (0) | 59.2% (29) |
|  |  |  | HHb ® ABP | 39.5% (15) | 50% (6) | 42.9% (12) | 40.9% (9) | 100% (1) | 40.8% (20) |
|  | Parietal | Left | ABP ® HHb | 50% (19) | 41.7% (5) | 50% (14) | 45.5% (10) | 100% (1) | 46.9% (23) |
|  |  |  | HHb ® ABP | 50% (19) | 58.3% (7) | 50% (14) | 54.5% (12) | 0% (0) | 53.1% (26) |
|  |  | Right | ABP ® HHb | 63.2% (24) | 58.3% (7) | 60.7% (17) | 63.6% (14) | 0% (0) | 63.3% (31) |
|  |  |  | HHb ® ABP | 36.8% (14) | 41.7% (5) | 39.3% (11) | 36.4% (8) | 100% (1) | 36.7% (18) |
|  | Occipital | Left | ABP ® HHb | 60.5% (23) | 66.7% (8) | 46.4% (13) | 81.8% (18) | 100% (1) | 61.2% (30) |
|  |  |  | HHb ® ABP | 39.5% (15) | 33.3% (4) | 53.6% (15) | 18.2% (4) | 0% (0) | 38.8% (19) |
|  |  | Right | ABP ® HHb | 55.3% (21) | 66.7% (8) | 60.7% (17) | 54.5% (12) | 0% (0) | 59.2% (29) |
|  |  |  | HHb ® ABP | 44.7% (17) | 33.3% (4) | 39.3% (11) | 45.5% (10) | 100% (1) | 40.8% (20) |
|  | Temporal | Left | ABP ® HHb | 50% (19) | 50% (6) | 60.7% (17) | 36.4% (8) | 0% (0) | 51% (25) |
|  |  |  | HHb ® ABP | 50% (19) | 50% (6) | 39.3% (11) | 63.6% (14) | 100% (1) | 49% (24) |
|  |  | Right | ABP ® HHb | 50% (19) | 41.7% (5) | 42.9% (12) | 54.5% (12) | 0% (0) | 49% (24) |
|  |  |  | HHb ® ABP | 50% (19) | 58.3% (7) | 57.1% (16) | 45.5% (10) | 100% (1) | 51% (25) |
| ABP & tHb | Frontal | Left | ABP ® tHb | 60.5% (23) | 41.7% (5) | 53.6% (15) | 59.1% (13) | 100% (1) | 55.1% (27) |
|  |  |  | tHb ® ABP | 39.5% (15) | 58.3% (7) | 46.4% (13) | 40.9% (9) | 0% (0) | 44.9% (22) |
|  |  | Right | ABP ® tHb | 52.6% (20) | 50% (6) | 50% (14) | 54.5% (12) | 0% (0) | 53.1% (26) |
|  |  |  | tHb ® ABP | 47.4% (18) | 50% (6) | 50% (14) | 45.5% (10) | 100% (1) | 46.9% (23) |
|  | Parietal | Left | ABP ® tHb | 44.7% (17) | 50% (6) | 42.9% (12) | 50% (11) | 0% (0) | 46.9% (23) |
|  |  |  | tHb ® ABP | 55.3% (21) | 50% (6) | 57.1% (16) | 50% (11) | 100% (1) | 53.1% (26) |
|  |  | Right | ABP ® tHb | 47.4% (18) | 58.3% (7) | 35.7% (10) | 68.2% (15) | 0% (0) | 51% (25) |
|  |  |  | tHb ® ABP | 52.6% (20) | 41.7% (5) | 64.3% (18) | 31.8% (7) | 100% (1) | 49% (24) |
|  | Occipital | Left | ABP ® tHb | 44.7% (17) | 58.3% (7) | 46.4% (13) | 50% (11) | 0% (0) | 49% (24) |
|  |  |  | tHb ® ABP | 55.3% (21) | 41.7% (5) | 53.6% (15) | 50% (11) | 100% (1) | 51% (25) |
|  |  | Right | ABP ® tHb | 65.8% (25) | 83.3% (10) | 67.9% (19) | 72.7% (16) | 0% (0) | 71.4% (35) |
|  |  |  | tHb ® ABP | 34.2% (13) | 16.7% (2) | 32.1% (9) | 27.3% (6) | 100% (1) | 28.6% (14) |
|  | Temporal | Left | ABP ® tHb | 44.7% (17) | 41.7% (5) | 42.9% (12) | 45.5% (10) | 0% (0) | 44.9% (22) |
|  |  |  | tHb ® ABP | 55.3% (21) | 58.3% (7) | 57.1% (16) | 54.5% (12) | 100% (1) | 55.1% (27) |
|  |  | Right | ABP ® tHb | 57.9% (22) | 41.7% (5) | 53.6% (15) | 54.5% (12) | 0% (0) | 55.1% (27) |
|  |  |  | tHb ® ABP | 42.1% (16) | 58.3% (7) | 46.4% (13) | 45.5% (10) | 100% (1) | 44.9% (22) |
| ABP & HbDiff | Frontal | Left | ABP ® HbDiff | 55.3% (21) | 33.3% (4) | 53.6% (15) | 45.5% (10) | 100% (1) | 49% (24) |
|  |  |  | HbDiff ® ABP | 44.7% (17) | 66.7% (8) | 46.4% (13) | 54.5% (12) | 0% (0) | 51% (25) |
|  |  | Right | ABP ® HbDiff | 57.9% (22) | 41.7% (5) | 53.6% (15) | 54.5% (12) | 0% (0) | 55.1% (27) |
|  |  |  | HbDiff ® ABP | 42.1% (16) | 58.3% (7) | 46.4% (13) | 45.5% (10) | 100% (1) | 44.9% (22) |
|  | Parietal | Left | ABP ® HbDiff | 44.7% (17) | 58.3% (7) | 42.9% (12) | 54.5% (12) | 0% (0) | 49% (24) |
|  |  |  | HbDiff ® ABP | 55.3% (21) | 41.7% (5) | 57.1% (16) | 45.5% (10) | 100% (1) | 51% (25) |
|  |  | Right | ABP ® HbDiff | 57.9% (22) | 58.3% (7) | 50% (14) | 68.2% (15) | 0% (0) | 59.2% (29) |
|  |  |  | HbDiff ® ABP | 42.1% (16) | 41.7% (5) | 50% (14) | 31.8% (7) | 100% (1) | 40.8% (20) |
|  | Occipital | Left | ABP ® HbDiff | 50% (19) | 58.3% (7) | 46.4% (13) | 59.1% (13) | 0% (0) | 53.1% (26) |
|  |  |  | HbDiff ® ABP | 50% (19) | 41.7% (5) | 53.6% (15) | 40.9% (9) | 100% (1) | 46.9% (23) |
|  |  | Right | ABP ® HbDiff | 52.6% (20) | 66.7% (8) | 50% (14) | 63.6% (14) | 100% (1) | 55.1% (27) |
|  |  |  | HbDiff ® ABP | 47.4% (18) | 33.3% (4) | 50% (14) | 36.4% (8) | 0% (0) | 44.9% (22) |
|  | Temporal | Left | ABP ® HbDiff | 50% (19) | 41.7% (5) | 50% (14) | 45.5% (10) | 0% (0) | 49% (24) |
|  |  |  | HbDiff ® ABP | 50% (19) | 58.3% (7) | 50% (14) | 54.5% (12) | 100% (1) | 51% (25) |
|  |  | Right | ABP ® HbDiff | 52.6% (20) | 50% (6) | 53.6% (15) | 50% (11) | 0% (0) | 53.1% (26) |
|  |  |  | HbDiff ® ABP | 47.4% (18) | 50% (6) | 46.4% (13) | 50% (11) | 100% (1) | 46.9% (23) |
| The table shows the subgrouped Granger causal directionality results between ABP and NIRS signals using 1 Hz and 250 Hz data. *ABP, arterial blood pressure; HbDiff, hemoglobin difference; HbO, oxyhemoglobin; HHb, deoxyhemoglobin; NIRS, near-infrared spectroscopy; rSO_2_, regional cerebral oxygen saturation; tHb, total hemoglobin.* | | | | | | | | | |
