# Supplementary material for: Detection of regional disparity in cerebrovascular reactivity using a custom whole brain functional near-infrared spectroscopy based mapping system: A prospective observational study
Source: PLOS Digit Health. 2026 Apr 15;5(4):e0001349. doi: 10.1371/journal.pdig.0001349 (PMC13082728; doi:10.1371/journal.pdig.0001349)
Supplement: S7 Appendix — (DOCX) [file pdig.0001349.s007.docx]

**Appendix S7 – Perturbation Subgroup Analysis**

Appendix S7 – Table of Contents

[Appendix S7a: Perturbation Subgrouped Median and IQR of CVR Indices Using 10-Second Decimated Data 2](#_Toc213066637)

[Appendix S7b: Perturbation Subgrouped Median and IQR of Physiologic Signals Using 10-Second Decimated Data 3](#_Toc213066638)

[Appendix S7c: Perturbation Subgrouped Median and IQR of Physiologic Signals Using Raw Data 5](#_Toc213066639)

[Appendix S7d: Perturbation Subgrouped Percent Time Results of rSO_2_ and CVR Indices Using 10-Second Decimated Data 7](#_Toc213066640)

[Appendix S7e: Perturbation Subgrouped Percent Time Results of rSO_2_ Using Raw Data 10](#_Toc213066641)

[Appendix S7f: Perturbation Subgrouped Regional Hemispheric Disparity Analysis on CVR Indices Using 10-Second Decimated Data 11](#_Toc213066642)

[Appendix S7g: Perturbation Subgrouped Regional Hemispheric Disparity Analysis on Physiologic Signals 12](#_Toc213066643)

[Appendix S7h: Perturbation Subgrouped Optimal ARIMA Models Based on AIC of Physiologic Signals and their Hemispheric Disparity 13](#_Toc213066644)

[Appendix S7i: Perturbation Subgrouped Hemispheric Responsiveness using Impulse Response Coefficients of Optimal VARIMA Model 17](#_Toc213066645)

[Appendix S7j: Perturbation Subgrouped Granger Causal Directionality Results Based on Greater F-Statistic 19](#_Toc213066646)

Appendix S7a: Perturbation Subgrouped Median and IQR of CVR Indices Using 10-Second Decimated Data

| **CVR Index** | **Brain Lobe** | **Baseline** | | | **Neurovascular Coupling** | | | **Orthostatic Challenge** | | | **Vascular Chemo-Reactivity** | | |
| --- | --- | --- | --- | --- | --- | --- | --- | --- | --- | --- | --- | --- | --- |
|  |  | **Median (IQR)** | | **p-value** | **Median (IQR)** | | **p-value** | **Median (IQR)** | | **p-value** | **Median (IQR)** | | **p-value** |
|  |  | **Left Hemisphere** | **Right Hemisphere** |  | **Left Hemisphere** | **Right Hemisphere** |  | **Left Hemisphere** | **Right Hemisphere** |  | **Left Hemisphere** | **Right Hemisphere** |  |
| **1 Hz Sampled Data** | | | | | | | | | | | | | |
| COx-a (au) | Frontal | 0.04 (-0.16 – 0.17) | 0.04 (-0.12 – 0.15) | 0.5602 | -0.03 (-0.2 – 0.16) | -0.03 (-0.2 – 0.16) | 0.6172 | 0 (-0.23 – 0.18) | 0 (-0.22 – 0.22) | 0.2597 | 0 (-0.15 – 0.13) | 0.01 (-0.12 – 0.14) | 0.2317 |
|  | Parietal | 0 (-0.16 – 0.17) | -0.03 (-0.17 – 0.16) | 0.6867 | -0.01 (-0.18 – 0.16) | -0.04 (-0.18 – 0.18) | 0.18 | -0.03 (-0.21 – 0.18) | -0.01 (-0.21 – 0.23) | 0.4755 | 0.04 (-0.11 – 0.16) | -0.02 (-0.13 – 0.15) | 0.1777 |
|  | Temporal | 0.04 (-0.06 – 0.18) | -0.02 (-0.17 – 0.15) | 0.0672 | -0.01 (-0.17 – 0.18) | 0.03 (-0.17 – 0.2) | 0.4971 | -0.01 (-0.2 – 0.19) | 0 (-0.2 – 0.19) | 0.7485 | 0.03 (-0.14 – 0.15) | 0 (-0.11 – 0.13) | 0.3158 |
|  | Occipital | 0.02 (-0.09 – 0.14) | 0.04 (-0.12 – 0.2) | 0.8931 | 0.01 (-0.11 – 0.14) | 0.01 (-0.17 – 0.2) | 0.4023 | 0 (-0.15 – 0.14) | 0.01 (-0.18 – 0.22) | 0.1891 | -0.02 (-0.1 – 0.1) | -0.01 (-0.17 – 0.11) | 0.9808 |
| HbOx (au) | Frontal | 0.04 (-0.19 – 0.15) | 0 (-0.14 – 0.15) | 0.9533 | -0.01 (-0.2 – 0.15) | -0.02 (-0.21 – 0.2) | 0.8281 | -0.03 (-0.23 – 0.18) | 0.02 (-0.2 – 0.24) | **0.039** | 0 (-0.15 – 0.15) | 0.02 (-0.13 – 0.16) | 0.7277 |
|  | Parietal | 0 (-0.14 – 0.16) | 0.04 (-0.18 – 0.17) | 0.9478 | 0 (-0.16 – 0.17) | 0 (-0.17 – 0.19) | 0.8876 | -0.01 (-0.23 – 0.23) | 0.03 (-0.18 – 0.26) | 0.2034 | -0.02 (-0.15 – 0.13) | -0.03 (-0.16 – 0.14) | 0.467 |
|  | Temporal | 0 (-0.15 – 0.16) | -0.03 (-0.17 – 0.15) | 0.3538 | 0 (-0.16 – 0.18) | -0.01 (-0.19 – 0.19) | 0.5147 | -0.01 (-0.21 – 0.21) | 0.02 (-0.16 – 0.23) | 0.4628 | 0.01 (-0.1 – 0.19) | -0.03 (-0.17 – 0.09) | 0.2426 |
|  | Occipital | 0.04 (-0.11 – 0.16) | -0.01 (-0.17 – 0.17) | 0.0291 | 0.03 (-0.12 – 0.15) | 0 (-0.19 – 0.17) | 0.8931 | 0 (-0.16 – 0.16) | 0.03 (-0.17 – 0.25) | 0.5742 | -0.02 (-0.12 – 0.09) | -0.02 (-0.17 – 0.1) | 0.812 |
| HHbx (au) | Frontal | -0.01 (-0.15 – 0.2) | -0.06 (-0.19 – 0.13) | 0.3538 | 0.02 (-0.15 – 0.2) | 0.04 (-0.18 – 0.2) | 0.5742 | 0.03 (-0.18 – 0.23) | 0.01 (-0.2 – 0.24) | 0.8335 | -0.01 (-0.14 – 0.14) | 0.01 (-0.14 – 0.13) | 0.904 |
|  | Parietal | -0.04 (-0.21 – 0.14) | 0 (-0.19 – 0.19) | 0.5837 | -0.01 (-0.18 – 0.2) | 0.01 (-0.21 – 0.19) | 0.7381 | 0.01 (-0.19 – 0.2) | 0.04 (-0.17 – 0.23) | 0.2482 | -0.02 (-0.18 – 0.13) | -0.04 (-0.19 – 0.13) | 0.7226 |
|  | Temporal | 0 (-0.18 – 0.15) | 0.04 (-0.14 – 0.17) | 0.4462 | 0.02 (-0.16 – 0.18) | -0.03 (-0.21 – 0.17) | 0.2317 | -0.04 (-0.21 – 0.21) | 0.04 (-0.18 – 0.23) | 0.1411 | 0 (-0.09 – 0.15) | -0.01 (-0.14 – 0.12) | 0.7538 |
|  | Occipital | -0.02 (-0.13 – 0.13) | 0 (-0.17 – 0.15) | 0.8931 | 0 (-0.12 – 0.14) | 0 (-0.17 – 0.18) | 0.467 | 0.03 (-0.12 – 0.17) | 0 (-0.19 – 0.23) | 0.3192 | 0.02 (-0.1 – 0.09) | 0.02 (-0.11 – 0.18) | 0.855 |
| tHbx (au) | Frontal | 0.04 (-0.17 – 0.18) | -0.01 (-0.16 – 0.21) | 0.4586 | 0.02 (-0.21 – 0.2) | 0 (-0.18 – 0.19) | 0.9259 | 0 (-0.23 – 0.19) | 0.02 (-0.2 – 0.24) | 0.2263 | -0.03 (-0.16 – 0.14) | 0 (-0.13 – 0.13) | 0.5464 |
|  | Parietal | -0.02 (-0.14 – 0.1) | 0.01 (-0.17 – 0.14) | 0.9313 | 0 (-0.16 – 0.21) | -0.02 (-0.16 – 0.2) | 0.6867 | 0.01 (-0.2 – 0.23) | 0.03 (-0.18 – 0.24) | 0.2185 | -0.04 (-0.17 – 0.12) | -0.03 (-0.19 – 0.14) | 0.7226 |
|  | Temporal | 0.02 (-0.13 – 0.17) | -0.01 (-0.15 – 0.17) | 0.8605 | -0.01 (-0.17 – 0.18) | -0.03 (-0.2 – 0.19) | 0.434 | 0 (-0.2 – 0.22) | 0.03 (-0.18 – 0.28) | 0.2211 | 0.02 (-0.1 – 0.17) | -0.02 (-0.15 – 0.11) | 0.2776 |
|  | Occipital | 0.04 (-0.1 – 0.19) | 0 (-0.16 – 0.18) | 0.1506 | 0.01 (-0.13 – 0.18) | 0 (-0.17 – 0.17) | 0.9643 | 0 (-0.14 – 0.2) | 0.02 (-0.18 – 0.25) | 0.7123 | -0.03 (-0.14 – 0.09) | -0.01 (-0.14 – 0.13) | 0.3467 |
| HbDiffx (au) | Frontal | 0.04 (-0.16 – 0.18) | 0.02 (-0.14 – 0.15) | 0.6817 | -0.03 (-0.19 – 0.18) | 0 (-0.2 – 0.16) | 0.9917 | 0 (-0.23 – 0.17) | 0.02 (-0.18 – 0.24) | 0.0842 | 0 (-0.15 – 0.13) | 0.01 (-0.1 – 0.14) | 0.3757 |
|  | Parietal | 0 (-0.16 – 0.16) | -0.05 (-0.17 – 0.17) | 0.7748 | -0.02 (-0.18 – 0.15) | -0.03 (-0.18 – 0.17) | 0.2482 | -0.01 (-0.2 – 0.21) | 0 (-0.21 – 0.23) | 0.5837 | 0.02 (-0.11 – 0.15) | 0 (-0.14 – 0.17) | 0.3647 |
|  | Temporal | 0.04 (-0.06 – 0.19) | -0.02 (-0.17 – 0.15) | 0.083 | -0.02 (-0.17 – 0.19) | 0.03 (-0.15 – 0.2) | 0.2656 | -0.01 (-0.21 – 0.2) | 0.01 (-0.19 – 0.2) | 0.8767 | 0.05 (-0.11 – 0.18) | -0.01 (-0.15 – 0.11) | 0.0672 |
|  | Occipital | 0.02 (-0.1 – 0.16) | 0.03 (-0.12 – 0.19) | 0.4299 | 0.02 (-0.1 – 0.14) | 0.01 (-0.17 – 0.2) | 0.4586 | 0 (-0.14 – 0.17) | 0.01 (-0.19 – 0.24) | 0.4219 | 0 (-0.1 – 0.09) | -0.01 (-0.18 – 0.12) | 0.9533 |
| MAD of COx-a (au) | Frontal | 0.12 (0.07 – 0.19) | 0.12 (0.07 – 0.18) | 0.9478 | 0.18 (0.14 – 0.22) | 0.17 (0.14 – 0.2) | 0.8174 | 0.22 (0.19 – 0.25) | 0.23 (0.19 – 0.25) | 0.6368 | 0.12 (0.08 – 0.15) | 0.11 (0.09 – 0.15) | 0.7695 |
|  | Parietal | 0.14 (0.1 – 0.18) | 0.15 (0.1 – 0.22) | 0.414 | 0.15 (0.11 – 0.19) | 0.16 (0.13 – 0.22) | 0.1045 | 0.2 (0.16 – 0.25) | 0.21 (0.18 – 0.23) | 0.5556 | 0.12 (0.09 – 0.17) | 0.12 (0.09 – 0.16) | 0.9313 |
|  | Temporal | 0.11 (0.08 – 0.17) | 0.12 (0.08 – 0.18) | 0.7854 | 0.17 (0.12 – 0.21) | 0.14 (0.12 – 0.19) | 0.3432 | 0.2 (0.17 – 0.23) | 0.2 (0.16 – 0.24) | 0.7433 | 0.12 (0.08 – 0.18) | 0.1 (0.08 – 0.14) | 0.4884 |
|  | Occipital | 0.1 (0.07 – 0.14) | 0.13 (0.09 – 0.2) | **0.0048** | 0.11 (0.08 – 0.14) | 0.17 (0.13 – 0.2) | **<0.001** | 0.14 (0.12 – 0.18) | 0.2 (0.17 – 0.24) | **<0.001** | 0.08 (0.06 – 0.1) | 0.11 (0.08 – 0.16) | **0.0013** |
| MAD of HbOx (au) | Frontal | 0.14 (0.1 – 0.2) | 0.11 (0.08 – 0.15) | **0.0276** | 0.19 (0.13 – 0.23) | 0.17 (0.13 – 0.21) | 0.3984 | 0.21 (0.18 – 0.23) | 0.22 (0.19 – 0.25) | 0.0693 | 0.12 (0.08 – 0.16) | 0.12 (0.09 – 0.16) | 0.8605 |
|  | Parietal | 0.12 (0.09 – 0.17) | 0.15 (0.09 – 0.19) | 0.2454 | 0.15 (0.13 – 0.2) | 0.17 (0.14 – 0.21) | 0.3125 | 0.22 (0.19 – 0.24) | 0.22 (0.17 – 0.26) | 0.9588 | 0.12 (0.1 – 0.16) | 0.12 (0.08 – 0.2) | 0.9368 |
|  | Temporal | 0.13 (0.1 – 0.18) | 0.13 (0.1 – 0.19) | 0.8174 | 0.16 (0.11 – 0.22) | 0.16 (0.14 – 0.2) | 0.372 | 0.21 (0.17 – 0.24) | 0.19 (0.15 – 0.25) | 0.3225 | 0.12 (0.09 – 0.17) | 0.12 (0.09 – 0.16) | 0.8442 |
|  | Occipital | 0.1 (0.07 – 0.17) | 0.14 (0.09 – 0.2) | **0.0271** | 0.14 (0.1 – 0.16) | 0.17 (0.12 – 0.2) | **<0.001** | 0.17 (0.14 – 0.22) | 0.21 (0.17 – 0.24) | **0.0102** | 0.1 (0.07 – 0.13) | 0.12 (0.09 – 0.18) | **0.0035** |
| MAD of HHbx (au) | Frontal | 0.14 (0.09 – 0.2) | 0.12 (0.08 – 0.19) | 0.132 | 0.17 (0.13 – 0.2) | 0.17 (0.14 – 0.21) | 0.4755 | 0.21 (0.19 – 0.23) | 0.22 (0.17 – 0.27) | 0.2745 | 0.13 (0.1 – 0.18) | 0.13 (0.09 – 0.16) | 0.9862 |
|  | Parietal | 0.13 (0.09 – 0.16) | 0.16 (0.1 – 0.21) | 0.0932 | 0.15 (0.12 – 0.2) | 0.16 (0.13 – 0.23) | 0.1356 | 0.2 (0.18 – 0.24) | 0.21 (0.18 – 0.25) | 0.9698 | 0.14 (0.09 – 0.2) | 0.13 (0.09 – 0.18) | 0.6466 |
|  | Temporal | 0.14 (0.09 – 0.18) | 0.13 (0.08 – 0.21) | 0.9533 | 0.17 (0.12 – 0.21) | 0.18 (0.13 – 0.23) | 0.3158 | 0.2 (0.16 – 0.24) | 0.19 (0.16 – 0.23) | 0.6075 | 0.13 (0.07 – 0.16) | 0.11 (0.08 – 0.16) | 0.6466 |
|  | Occipital | 0.1 (0.07 – 0.15) | 0.14 (0.09 – 0.19) | **0.0137** | 0.12 (0.09 – 0.15) | 0.16 (0.13 – 0.21) | **<0.001** | 0.15 (0.13 – 0.19) | 0.21 (0.17 – 0.24) | **<0.001** | 0.08 (0.07 – 0.11) | 0.13 (0.08 – 0.18) | **0.0044** |
| MAD of tHbx (au) | Frontal | 0.15 (0.1 – 0.2) | 0.13 (0.09 – 0.19) | 0.3092 | 0.19 (0.13 – 0.23) | 0.17 (0.13 – 0.2) | 0.1105 | 0.21 (0.18 – 0.23) | 0.21 (0.18 – 0.25) | 0.2371 | 0.12 (0.09 – 0.17) | 0.11 (0.09 – 0.16) | 0.702 |
|  | Parietal | 0.11 (0.08 – 0.16) | 0.15 (0.08 – 0.21) | 0.0973 | 0.16 (0.13 – 0.21) | 0.17 (0.14 – 0.21) | 0.8388 | 0.21 (0.19 – 0.24) | 0.21 (0.19 – 0.26) | 0.3869 | 0.13 (0.1 – 0.17) | 0.13 (0.08 – 0.19) | 0.9972 |
|  | Temporal | 0.13 (0.08 – 0.17) | 0.13 (0.1 – 0.19) | 0.3794 | 0.17 (0.13 – 0.21) | 0.18 (0.14 – 0.23) | 0.3574 | 0.22 (0.18 – 0.27) | 0.2 (0.17 – 0.26) | 0.2685 | 0.1 (0.08 – 0.17) | 0.12 (0.09 – 0.15) | 0.2568 |
|  | Occipital | 0.1 (0.08 – 0.16) | 0.14 (0.11 – 0.21) | **0.0094** | 0.14 (0.11 – 0.18) | 0.17 (0.12 – 0.21) | 0.0652 | 0.19 (0.15 – 0.22) | 0.21 (0.17 – 0.24) | 0.0584 | 0.1 (0.07 – 0.15) | 0.13 (0.09 – 0.17) | **0.0445** |
| MAD of HbDiffx (au) | Frontal | 0.14 (0.07 – 0.19) | 0.11 (0.07 – 0.17) | 0.6075 | 0.18 (0.13 – 0.21) | 0.17 (0.14 – 0.21) | 0.8335 | 0.22 (0.2 – 0.24) | 0.22 (0.18 – 0.26) | 0.8013 | 0.11 (0.08 – 0.16) | 0.11 (0.09 – 0.18) | 0.7381 |
|  | Parietal | 0.14 (0.1 – 0.19) | 0.15 (0.11 – 0.21) | 0.5237 | 0.15 (0.11 – 0.19) | 0.16 (0.13 – 0.22) | 0.0714 | 0.2 (0.16 – 0.25) | 0.21 (0.17 – 0.24) | 0.9313 | 0.12 (0.09 – 0.17) | 0.12 (0.09 – 0.16) | 0.8013 |
|  | Temporal | 0.12 (0.09 – 0.17) | 0.14 (0.09 – 0.2) | 0.6918 | 0.15 (0.12 – 0.2) | 0.15 (0.12 – 0.18) | 0.5742 | 0.19 (0.16 – 0.22) | 0.2 (0.17 – 0.24) | 0.4023 | 0.12 (0.08 – 0.15) | 0.11 (0.08 – 0.15) | 0.6666 |
|  | Occipital | 0.1 (0.08 – 0.14) | 0.13 (0.09 – 0.21) | **0.0106** | 0.11 (0.09 – 0.15) | 0.16 (0.12 – 0.2) | **<0.001** | 0.16 (0.12 – 0.19) | 0.2 (0.16 – 0.23) | **<0.001** | 0.09 (0.07 – 0.11) | 0.11 (0.08 – 0.17) | **0.0029** |
| **250 Hz Sampled Data** | | | | | | | | | | | | | |
| COx-a (au) | Frontal | 0.02 (-0.16 – 0.18) | 0.05 (-0.13 – 0.17) | 0.438 | -0.02 (-0.2 – 0.17) | -0.03 (-0.21 – 0.18) | 0.5649 | -0.03 (-0.23 – 0.17) | 0 (-0.21 – 0.22) | 0.1755 | 0 (-0.16 – 0.15) | 0.03 (-0.13 – 0.14) | 0.251 |
|  | Parietal | -0.02 (-0.16 – 0.16) | -0.04 (-0.16 – 0.16) | 0.5602 | -0.01 (-0.18 – 0.16) | -0.03 (-0.19 – 0.18) | 0.1448 | -0.03 (-0.22 – 0.18) | 0 (-0.21 – 0.22) | 0.3683 | 0.04 (-0.12 – 0.16) | -0.01 (-0.14 – 0.16) | 0.2237 |
|  | Temporal | 0.06 (-0.09 – 0.17) | -0.02 (-0.2 – 0.15) | 0.0189 | -0.02 (-0.17 – 0.15) | 0.02 (-0.17 – 0.2) | 0.2806 | -0.01 (-0.19 – 0.21) | 0 (-0.2 – 0.18) | 0.9533 | 0.01 (-0.14 – 0.15) | -0.02 (-0.13 – 0.08) | 0.361 |
|  | Occipital | 0.03 (-0.11 – 0.14) | 0.03 (-0.14 – 0.2) | 0.5103 | 0 (-0.12 – 0.12) | 0.01 (-0.17 – 0.2) | 0.5147 | 0 (-0.15 – 0.15) | 0 (-0.19 – 0.21) | 0.3907 | -0.04 (-0.12 – 0.1) | -0.02 (-0.15 – 0.09) | 0.6172 |
| HbOx (au) | Frontal | 0.04 (-0.17 – 0.16) | 0.02 (-0.13 – 0.16) | 0.9478 | -0.01 (-0.2 – 0.15) | -0.02 (-0.2 – 0.2) | 0.8767 | -0.03 (-0.22 – 0.19) | 0.02 (-0.21 – 0.25) | 0.037 | 0.01 (-0.14 – 0.15) | 0 (-0.12 – 0.15) | 0.8174 |
|  | Parietal | 0.01 (-0.15 – 0.16) | 0.02 (-0.18 – 0.17) | 0.812 | 0.01 (-0.17 – 0.18) | 0.01 (-0.17 – 0.19) | 0.8822 | -0.01 (-0.24 – 0.23) | 0.03 (-0.18 – 0.26) | 0.18 | -0.01 (-0.14 – 0.14) | -0.03 (-0.17 – 0.14) | 0.3125 |
|  | Temporal | 0.01 (-0.15 – 0.18) | -0.04 (-0.17 – 0.13) | 0.2539 | -0.01 (-0.17 – 0.17) | -0.01 (-0.19 – 0.2) | 0.3647 | 0 (-0.21 – 0.2) | 0.01 (-0.17 – 0.22) | 0.4462 | -0.01 (-0.1 – 0.14) | -0.04 (-0.18 – 0.06) | 0.2995 |
|  | Occipital | 0.03 (-0.1 – 0.19) | -0.01 (-0.17 – 0.15) | **0.049** | 0.01 (-0.11 – 0.15) | 0 (-0.19 – 0.16) | 0.9094 | 0 (-0.15 – 0.18) | 0.04 (-0.17 – 0.25) | 0.6616 | -0.03 (-0.13 – 0.1) | -0.02 (-0.14 – 0.1) | 0.812 |
| HHbx (au) | Frontal | 0.01 (-0.16 – 0.22) | -0.06 (-0.17 – 0.15) | 0.3328 | 0.03 (-0.16 – 0.2) | 0.02 (-0.18 – 0.2) | 0.6075 | 0.02 (-0.18 – 0.22) | 0 (-0.2 – 0.23) | 0.8822 | 0 (-0.14 – 0.14) | 0 (-0.14 – 0.13) | 0.9588 |
|  | Parietal | -0.01 (-0.21 – 0.12) | 0.03 (-0.18 – 0.18) | 0.5015 | 0 (-0.16 – 0.2) | 0.01 (-0.2 – 0.22) | 0.6516 | 0.03 (-0.19 – 0.21) | 0.04 (-0.17 – 0.23) | 0.2482 | -0.01 (-0.18 – 0.12) | -0.03 (-0.21 – 0.13) | 0.9862 |
|  | Temporal | 0 (-0.17 – 0.14) | 0.04 (-0.15 – 0.2) | 0.3574 | 0.03 (-0.15 – 0.19) | -0.03 (-0.22 – 0.16) | 0.0842 | -0.03 (-0.21 – 0.2) | 0.02 (-0.19 – 0.22) | 0.2211 | 0.01 (-0.1 – 0.17) | 0 (-0.12 – 0.1) | 0.551 |
|  | Occipital | -0.02 (-0.15 – 0.11) | 0 (-0.18 – 0.16) | 0.5464 | 0 (-0.1 – 0.15) | 0.01 (-0.16 – 0.17) | 0.5742 | 0.03 (-0.14 – 0.17) | 0 (-0.2 – 0.23) | 0.5327 | 0 (-0.12 – 0.12) | 0.02 (-0.1 – 0.15) | 0.3907 |
| tHbx (au) | Frontal | 0.05 (-0.16 – 0.18) | -0.03 (-0.16 – 0.2) | 0.4841 | 0.02 (-0.2 – 0.21) | -0.02 (-0.19 – 0.2) | 0.8713 | 0 (-0.22 – 0.19) | 0.02 (-0.21 – 0.23) | 0.2482 | -0.02 (-0.14 – 0.15) | 0.01 (-0.13 – 0.13) | 0.7643 |
|  | Parietal | -0.02 (-0.15 – 0.12) | 0 (-0.15 – 0.15) | 0.9862 | 0.01 (-0.15 – 0.2) | -0.02 (-0.16 – 0.2) | 0.6666 | 0.01 (-0.2 – 0.23) | 0.02 (-0.19 – 0.24) | 0.2995 | -0.04 (-0.18 – 0.14) | -0.04 (-0.18 – 0.13) | 0.6221 |
|  | Temporal | 0.01 (-0.12 – 0.17) | 0 (-0.18 – 0.19) | 0.7854 | -0.01 (-0.16 – 0.18) | -0.03 (-0.21 – 0.19) | 0.3946 | 0 (-0.21 – 0.21) | 0.02 (-0.18 – 0.26) | 0.2371 | 0.03 (-0.08 – 0.15) | -0.03 (-0.16 – 0.11) | 0.1268 |
|  | Occipital | 0.02 (-0.1 – 0.17) | 0 (-0.17 – 0.16) | 0.1938 | 0.02 (-0.13 – 0.18) | 0.01 (-0.17 – 0.17) | 0.5789 | 0 (-0.14 – 0.19) | 0.01 (-0.18 – 0.25) | 0.6817 | -0.01 (-0.14 – 0.07) | 0.01 (-0.13 – 0.14) | 0.3467 |
| HbDiffx (au) | Frontal | 0.03 (-0.15 – 0.19) | 0.02 (-0.13 – 0.15) | 0.7433 | -0.02 (-0.19 – 0.18) | 0 (-0.2 – 0.16) | 0.9423 | -0.01 (-0.24 – 0.17) | 0.01 (-0.17 – 0.24) | 0.0523 | 0 (-0.16 – 0.15) | 0.03 (-0.11 – 0.14) | 0.3946 |
|  | Parietal | -0.03 (-0.17 – 0.16) | -0.05 (-0.16 – 0.16) | 0.4928 | -0.01 (-0.18 – 0.15) | -0.03 (-0.19 – 0.18) | 0.2317 | -0.02 (-0.22 – 0.2) | 0.01 (-0.21 – 0.23) | 0.6172 | 0.04 (-0.12 – 0.15) | 0.01 (-0.15 – 0.17) | 0.414 |
|  | Temporal | 0.06 (-0.07 – 0.17) | -0.02 (-0.22 – 0.15) | **0.0257** | -0.02 (-0.18 – 0.16) | 0.02 (-0.17 – 0.22) | 0.1429 | -0.01 (-0.19 – 0.2) | 0 (-0.19 – 0.2) | 0.8931 | 0.05 (-0.1 – 0.16) | -0.02 (-0.14 – 0.07) | 0.0854 |
|  | Occipital | 0.03 (-0.1 – 0.17) | 0.02 (-0.14 – 0.2) | 0.2059 | 0.01 (-0.12 – 0.13) | 0.01 (-0.17 – 0.19) | 0.6027 | 0 (-0.13 – 0.16) | 0.02 (-0.19 – 0.24) | 0.7485 | -0.03 (-0.11 – 0.1) | -0.02 (-0.17 – 0.1) | 0.7854 |
| MAD of COx-a (au) | Frontal | 0.12 (0.09 – 0.19) | 0.11 (0.08 – 0.19) | 0.7748 | 0.17 (0.13 – 0.22) | 0.17 (0.14 – 0.21) | 0.8876 | 0.21 (0.18 – 0.25) | 0.23 (0.19 – 0.25) | 0.4586 | 0.12 (0.08 – 0.15) | 0.13 (0.08 – 0.16) | 0.8713 |
|  | Parietal | 0.14 (0.1 – 0.19) | 0.15 (0.1 – 0.23) | 0.5884 | 0.16 (0.11 – 0.19) | 0.16 (0.13 – 0.23) | 0.2568 | 0.2 (0.16 – 0.24) | 0.21 (0.17 – 0.24) | 0.3467 | 0.13 (0.09 – 0.17) | 0.12 (0.09 – 0.18) | 0.8496 |
|  | Temporal | 0.11 (0.08 – 0.18) | 0.13 (0.08 – 0.19) | 0.467 | 0.16 (0.12 – 0.2) | 0.15 (0.12 – 0.19) | 0.4299 | 0.2 (0.16 – 0.23) | 0.19 (0.16 – 0.23) | 0.9149 | 0.11 (0.08 – 0.18) | 0.11 (0.07 – 0.15) | 0.5418 |
|  | Occipital | 0.1 (0.07 – 0.16) | 0.12 (0.09 – 0.2) | 0.0622 | 0.12 (0.08 – 0.14) | 0.18 (0.12 – 0.21) | **<0.001** | 0.14 (0.12 – 0.18) | 0.2 (0.16 – 0.24) | **<0.001** | 0.08 (0.07 – 0.13) | 0.11 (0.08 – 0.15) | **0.0302** |
| MAD of HbOx (au) | Frontal | 0.15 (0.1 – 0.2) | 0.1 (0.09 – 0.16) | **0.037** | 0.18 (0.13 – 0.22) | 0.16 (0.13 – 0.21) | 0.3397 | 0.21 (0.18 – 0.24) | 0.23 (0.19 – 0.26) | 0.0584 | 0.12 (0.1 – 0.17) | 0.12 (0.09 – 0.16) | 0.6027 |
|  | Parietal | 0.12 (0.09 – 0.16) | 0.15 (0.09 – 0.19) | 0.1733 | 0.17 (0.14 – 0.19) | 0.18 (0.14 – 0.21) | 0.3757 | 0.22 (0.19 – 0.24) | 0.22 (0.17 – 0.26) | 0.8442 | 0.11 (0.09 – 0.16) | 0.13 (0.09 – 0.19) | 0.7174 |
|  | Temporal | 0.13 (0.09 – 0.18) | 0.13 (0.1 – 0.18) | 0.8985 | 0.16 (0.12 – 0.22) | 0.17 (0.14 – 0.21) | 0.3467 | 0.21 (0.18 – 0.24) | 0.19 (0.14 – 0.25) | 0.2685 | 0.12 (0.08 – 0.17) | 0.11 (0.08 – 0.15) | 0.8442 |
|  | Occipital | 0.11 (0.07 – 0.14) | 0.13 (0.09 – 0.18) | 0.0584 | 0.14 (0.1 – 0.16) | 0.18 (0.12 – 0.21) | **<0.001** | 0.17 (0.13 – 0.21) | 0.21 (0.17 – 0.24) | **0.0068** | 0.09 (0.06 – 0.13) | 0.12 (0.09 – 0.18) | **0.0117** |
| MAD of HHbx (au) | Frontal | 0.13 (0.1 – 0.2) | 0.12 (0.07 – 0.18) | 0.1467 | 0.16 (0.13 – 0.2) | 0.17 (0.14 – 0.21) | 0.5059 | 0.21 (0.18 – 0.23) | 0.23 (0.18 – 0.27) | 0.2134 | 0.12 (0.09 – 0.16) | 0.14 (0.09 – 0.18) | 0.6027 |
|  | Parietal | 0.13 (0.09 – 0.17) | 0.16 (0.09 – 0.2) | 0.2482 | 0.15 (0.12 – 0.2) | 0.17 (0.14 – 0.22) | 0.1566 | 0.21 (0.18 – 0.24) | 0.21 (0.18 – 0.26) | 0.9972 | 0.13 (0.09 – 0.19) | 0.13 (0.08 – 0.18) | 0.7538 |
|  | Temporal | 0.14 (0.07 – 0.18) | 0.12 (0.08 – 0.22) | 0.796 | 0.17 (0.11 – 0.21) | 0.17 (0.14 – 0.23) | 0.3125 | 0.19 (0.17 – 0.23) | 0.19 (0.16 – 0.23) | 0.7907 | 0.12 (0.07 – 0.16) | 0.11 (0.08 – 0.14) | 0.8174 |
|  | Occipital | 0.11 (0.09 – 0.15) | 0.13 (0.09 – 0.19) | 0.0531 | 0.12 (0.1 – 0.15) | 0.15 (0.14 – 0.21) | **<0.001** | 0.15 (0.13 – 0.2) | 0.21 (0.17 – 0.24) | **<0.001** | 0.09 (0.06 – 0.14) | 0.12 (0.08 – 0.18) | **0.0329** |
| MAD of tHbx (au) | Frontal | 0.15 (0.1 – 0.21) | 0.12 (0.09 – 0.19) | 0.2482 | 0.19 (0.13 – 0.23) | 0.17 (0.13 – 0.21) | 0.1136 | 0.2 (0.17 – 0.23) | 0.21 (0.17 – 0.26) | 0.229 | 0.12 (0.08 – 0.18) | 0.11 (0.08 – 0.17) | 0.7174 |
|  | Parietal | 0.11 (0.08 – 0.16) | 0.15 (0.09 – 0.2) | 0.1669 | 0.16 (0.13 – 0.22) | 0.17 (0.14 – 0.21) | 0.5192 | 0.2 (0.17 – 0.24) | 0.22 (0.19 – 0.26) | 0.4259 | 0.13 (0.09 – 0.17) | 0.13 (0.09 – 0.19) | 0.8013 |
|  | Temporal | 0.13 (0.09 – 0.17) | 0.12 (0.09 – 0.19) | 0.4586 | 0.16 (0.13 – 0.21) | 0.18 (0.15 – 0.24) | 0.1777 | 0.21 (0.19 – 0.27) | 0.2 (0.17 – 0.26) | 0.2626 | 0.09 (0.08 – 0.16) | 0.12 (0.09 – 0.15) | 0.1526 |
|  | Occipital | 0.12 (0.08 – 0.16) | 0.13 (0.12 – 0.2) | 0.0531 | 0.14 (0.12 – 0.17) | 0.16 (0.12 – 0.21) | 0.088 | 0.19 (0.15 – 0.22) | 0.21 (0.17 – 0.25) | **0.043** | 0.1 (0.07 – 0.16) | 0.13 (0.09 – 0.17) | 0.0613 |
| MAD of HbDiffx (au) | Frontal | 0.14 (0.07 – 0.19) | 0.11 (0.08 – 0.19) | 0.5418 | 0.18 (0.13 – 0.21) | 0.17 (0.14 – 0.22) | 0.6466 | 0.22 (0.19 – 0.24) | 0.23 (0.17 – 0.25) | 0.5418 | 0.12 (0.08 – 0.16) | 0.13 (0.08 – 0.18) | 0.5979 |
|  | Parietal | 0.15 (0.1 – 0.19) | 0.14 (0.1 – 0.21) | 0.9094 | 0.15 (0.11 – 0.2) | 0.16 (0.13 – 0.23) | 0.1448 | 0.2 (0.16 – 0.25) | 0.21 (0.17 – 0.24) | 0.627 | 0.12 (0.09 – 0.17) | 0.12 (0.09 – 0.17) | 0.6766 |
|  | Temporal | 0.12 (0.08 – 0.18) | 0.14 (0.08 – 0.18) | 0.5327 | 0.15 (0.12 – 0.21) | 0.15 (0.13 – 0.18) | 0.6666 | 0.19 (0.16 – 0.22) | 0.2 (0.17 – 0.23) | 0.4545 | 0.11 (0.08 – 0.16) | 0.11 (0.08 – 0.15) | 0.8496 |
|  | Occipital | 0.11 (0.08 – 0.14) | 0.12 (0.08 – 0.2) | 0.1985 | 0.12 (0.08 – 0.14) | 0.16 (0.11 – 0.21) | **<0.001** | 0.15 (0.12 – 0.2) | 0.2 (0.17 – 0.23) | **<0.001** | 0.08 (0.07 – 0.13) | 0.12 (0.08 – 0.15) | **0.0235** |
| The p-values in the table are derived using Mann-Whitney U test between the bilateral signals for the perturbation subgroups. *COx-a, cerebral oximetry index with arterial blood pressure; CVR, cerebrovascular reactivity index; HbDiffx, hemoglobin difference index; HbOx, oxyhemoglobin index; HHbx, deoxyhemoglobin index; IQR, interquartile range; MAD, median absolute deviation; tHbx, total hemoglobin index.* | | | | | | | | | | | | | |

Appendix S7b: Perturbation Subgrouped Median and IQR of Physiologic Signals Using 10-Second Decimated Data

| **Physiologic Signal** | **Brain Lobe** | **Baseline** | | | **Neurovascular Coupling** | | | **Orthostatic Challenge** | | | **Vascular Chemo-Reactivity** | | |
| --- | --- | --- | --- | --- | --- | --- | --- | --- | --- | --- | --- | --- | --- |
|  |  | **Median (IQR)** | | **p-value** | **Median (IQR)** | | **p-value** | **Median (IQR)** | | **p-value** | **Median (IQR)** | | **p-value** |
|  |  | **Left Hemisphere** | **Right Hemisphere** |  | **Left Hemisphere** | **Right Hemisphere** |  | **Left Hemisphere** | **Right Hemisphere** |  | **Left Hemisphere** | **Right Hemisphere** |  |
| **1 Hz Sampled Data** | | | | | | | | | | | | | |
| ABP (mmHg) | – | 95.47 (90.04 – 100.81) | | – | 97.35 (91.55 – 100.78) | | – | 95.33 (89.2 – 100.7) | | – | 95.3 (91.95 – 99.92) | | – |
| EtCO₂ (mmHg) | – | 35.84 (34.23 – 36.95) | | – | 35.86 (35 – 36.97) | | – | 35.56 (34.52 – 37.12) | | – | 28.89 (26.13 – 31.23) | | – |
| RR (bpm) | – | 18 (14 – 19.9) | | – | 18.61 (15.75 – 20) | | – | 17.47 (14.81 – 19.5) | | – | 14.02 (9.92 – 24.74) | | – |
| rSO_2_ (%) | Frontal | 45.62 (44.99 – 46.45) | 40.37 (39.45 – 40.91) | **<0.001** | 46.23 (45.78 – 46.45) | 40.32 (39.99 – 40.83) | **<0.001** | 45.28 (44.76 – 45.95) | 40.46 (39.37 – 41.23) | **<0.001** | 46.79 (46.24 – 47.1) | 40.69 (40.38 – 40.83) | **<0.001** |
|  | Parietal | 49.08 (47.44 – 50.78) | 46.03 (45.09 – 47.21) | **0.0067** | 48.61 (48.13 – 49.98) | 46.46 (46 – 46.87) | 0.0632 | 48.95 (47.29 – 50.57) | 45.25 (43.92 – 47.09) | **<0.001** | 48.81 (48.37 – 49.25) | 44.74 (44.44 – 45.8) | **<0.001** |
|  | Temporal | 40.11 (38.81 – 41.26) | 48 (46.93 – 48.7) | **<0.001** | 40.03 (39.33 – 40.26) | 48.33 (47.69 – 48.6) | **<0.001** | 40.55 (39.17 – 41.92) | 47.77 (47.17 – 48.59) | **<0.001** | 40.21 (40.05 – 40.45) | 47.45 (47.16 – 47.77) | **<0.001** |
|  | Occipital | 35.89 (35.66 – 36.27) | 47.33 (45.83 – 48.13) | **<0.001** | 36.07 (35.73 – 36.35) | 47.59 (47.2 – 47.92) | **<0.001** | 35.84 (35.38 – 36.33) | 46.66 (45.82 – 47.66) | **<0.001** | 35.93 (35.55 – 36.17) | 47.03 (46.64 – 47.65) | **<0.001** |
| HbO (au) | Frontal | -117.22  (-128.87 – -114.11) | -133.26  (-137.48 – -123.28) | **0.0179** | -117.21  (-120.42 – -116.64) | -132.58  (-134.02 – -131.48) | **0.0122** | -121.06  (-125.09 – -117.44) | -126.43  (-135.96 – -124.43) | 0.106 | -120.56  (-122.71 – -119.3) | -125.83  (-128.38 – -125.13) | 0.1448 |
|  | Parietal | -165.48  (-180.64 – -158.37) | -176.22  (-202.78 – -171.11) | 0.1546 | -168.7  (-174.99 – -163.38) | -175.37  (-176.73 – -174.05) | 0.251 | -174.68  (-185.16 – -162.86) | -190.74  (-205.82 – -177.11) | 0.1822 | -171.39  (-172.21 – -168.22) | -186.75  (-188.28 – -183.4) | 0.1285 |
|  | Temporal | -208.75  (-220.8 – -204.19) | -147.83  (-155.57 – -141.27) | **0.0096** | -208.02  (-211.81 – -207.17) | -148.94  (-149.57 – -146.15) | **0.0154** | -210.74  (-221.75 – -199.27) | -155.92  (-162.95 – -150.21) | **0.0302** | -216.23  (-219.99 – -215.39) | -155.09  (-155.98 – -154.23) | **0.0334** |
|  | Occipital | -501.82  (-507.49 – -481.72) | -189.72  (-198.93 – -177.03) | **<0.001** | -499.18  (-501.38 – -494.77) | -174.39  (-174.87 – -170.11) | **<0.001** | -500.15  (-506.22 – -491.06) | -194.44  (-214.95 – -181.56) | **<0.001** | -505.33  (-507.78 – -503.38) | -193.76  (-197.03 – -192.43) | **<0.001** |
| HHb (au) | Frontal | -109.87  (-116.17 – -105.73) | -91.67  (-100.19 – -79.66) | 0.132 | -108.27  (-110.97 – -107.28) | -93  (-95.01 – -91.14) | 0.1733 | -109.24  (-111.23 – -106.38) | -82.18  (-89.67 – -80.77) | 0.0714 | -110.88  (-111.61 – -110.08) | -86.86  (-91.11 – -82.7) | 0.0523 |
|  | Parietal | -176.52  (-195 – -157.72) | -178.74  (-187.93 – -152.24) | 0.8335 | -177.73  (-187.93 – -172.83) | -175.64  (-177.29 – -171.77) | 0.8713 | -164.24  (-175.98 – -158.45) | -157.7  (-176.69 – -150.43) | 0.5884 | -160.56  (-161.26 – -158.04) | -149.92  (-151.46 – -149.49) | 0.4928 |
|  | Temporal | -140.16  (-160.36 – -134.09) | -146.76  (-167.4 – -135.65) | 0.5556 | -144.74  (-149.78 – -139.31) | -147.18  (-151.37 – -143.34) | 0.5418 | -144.38  (-158.57 – -134.82) | -145.57  (-151.98 – -137.8) | 0.6319 | -152.96  (-162.18 – -149.89) | -149.45  (-150.35 – -147.1) | 0.5742 |
|  | Occipital | -274.58  (-280.96 – -269.63) | -160.18  (-168.99 – -155.16) | **<0.001** | -276.8  (-279.11 – -274.44) | -157.63  (-160.17 – -155.26) | **<0.001** | -273.82  (-279.71 – -269.46) | -174.59  (-186.81 – -159.36) | **<0.001** | -275.92  (-278.86 – -273.76) | -172.91  (-173.12 – -172.25) | **<0.001** |
| tHb (au) | Frontal | -221.77  (-238.14 – -216.12) | -225.86  (-243.38 – -202.85) | 0.9533 | -221.88  (-235.64 – -220.39) | -223.46  (-226.66 – -222.15) | 0.8227 | -228.06  (-238.82 – -221.51) | -209.71  (-231.89 – -205.2) | 0.5837 | -225.18  (-227.32 – -221.8) | -213.3  (-216.25 – -212.67) | 0.4545 |
|  | Parietal | -340.17  (-378.18 – -319.49) | -342.62  (-385.16 – -329.01) | 0.5237 | -344.31  (-360.58 – -334.21) | -347.27  (-349.7 – -340.2) | 0.5837 | -335.7  (-353.95 – -325.76) | -349.18  (-372.06 – -332.83) | 0.5464 | -332.75  (-333.78 – -330.97) | -341.77  (-343.24 – -334.72) | 0.5103 |
|  | Temporal | -352.38  (-381.33 – -339.64) | -297.35  (-325.68 – -277.8) | 0.2398 | -350.9  (-354.91 – -349.8) | -294.79  (-300.24 – -288.77) | 0.2776 | -360.41  (-382.8 – -342.2) | -308.82  (-319.89 – -295.73) | 0.3293 | -365.2  (-375.19 – -364.56) | -306.93  (-308.03 – -305.38) | 0.3293 |
|  | Occipital | -779.3  (-794.91 – -755.39) | -353.43  (-367.77 – -336.49) | **<0.001** | -774.34  (-778.34 – -771.4) | -330.94  (-338.62 – -324.07) | **<0.001** | -771.73  (-784.94 – -761.03) | -374.21  (-403.37 – -344.33) | **<0.001** | -786.41  (-791.64 – -783.48) | -363.09  (-365.36 – -361.21) | **<0.001** |
| HbDiff (au) | Frontal | -19.79  (-21.98 – -15.88) | -43.39  (-47.47 – -38.43) | **<0.001** | -17.49  (-18.31 – -16.51) | -43.44  (-45.27 – -41.53) | **<0.001** | -19.8  (-21.61 – -16.17) | -44.56  (-48.54 – -41.53) | **<0.001** | -15.7  (-17.04 – -14.65) | -43.09  (-43.5 – -41.38) | **<0.001** |
|  | Parietal | -5.79  (-10.57 – 4.65) | -22.61  (-30.98 – -17.78) | **0.0132** | -7.73  (-8.26 – -0.29) | -22.1  (-23.11 – -19.1) | 0.103 | -7.38  (-15.87 – 3.61) | -31.65  (-39 – -19.13) | **<0.001** | -8.49  (-9.44 – -4.2) | -30.28  (-32.53 – -27.55) | **<0.001** |
|  | Temporal | -54.2  (-72.59 – -51.15) | -12.64  (-17.2 – -6) | **<0.001** | -59.35  (-65.35 – -56.58) | -10.87  (-13.47 – -10.2) | **<0.001** | -60.83  (-67 – -54.94) | -10.63  (-16.32 – -5.88) | **<0.001** | -58.98  (-60.58 – -55.07) | -12.21  (-12.74 – -11.47) | **<0.001** |
|  | Occipital | -216.07  (-223.63 – -209.44) | -18.83  (-25.41 – -11.08) | **<0.001** | -215.26  (-220.23 – -211.78) | -15.77  (-18.51 – -13.81) | **<0.001** | -223.71  (-229.1 – -212.13) | -20.79  (-28.5 – -16.59) | **<0.001** | -224.96  (-230.13 – -220.07) | -19.89  (-22.51 – -18.83) | **<0.001** |
| MAD of ABP (mmHg) | – | 4.2 (3.13 – 6.12) | | – | 4.3 (3.49 – 5.17) | | – | 5.38 (4.61 – 6.41) | | – | 3.73 (2.85 – 4.83) | | – |
| MAD of EtCO₂ (mmHg) | – | 1.01 (0.83 – 1.39) | | – | 0.85 (0.61 – 1) | | – | 1.18 (0.93 – 1.44) | | – | 1.77 (1.1 – 2.67) | | – |
| MAD of RR (bpm) | – | 2 (1.11 – 2.68) | | – | 1.67 (1.09 – 2.27) | | – | 2.02 (1.78 – 2.73) | | – | 5.18 (2.13 – 6.03) | | – |
| MAD of rSO_2_ (%) | Frontal | 0.39 (0.23 – 0.81) | 0.54 (0.34 – 1.2) | 0.1184 | 0.21 (0.13 – 0.41) | 0.21 (0.14 – 0.5) | 0.6918 | 0.5 (0.36 – 0.75) | 0.8 (0.5 – 1.13) | **0.0027** | 0.13 (0.08 – 0.28) | 0.21 (0.08 – 0.45) | 0.1251 |
|  | Parietal | 0.49 (0.33 – 1.14) | 0.59 (0.23 – 1.39) | 0.8605 | 0.38 (0.19 – 0.61) | 0.33 (0.13 – 0.61) | 0.5602 | 1.01 (0.64 – 1.52) | 1.18 (0.66 – 1.73) | 0.5837 | 0.19 (0.13 – 0.41) | 0.17 (0.1 – 0.38) | 0.434 |
|  | Temporal | 0.5 (0.17 – 0.88) | 0.47 (0.31 – 0.82) | 0.5418 | 0.31 (0.15 – 0.47) | 0.27 (0.15 – 0.38) | 0.4798 | 0.71 (0.49 – 1.09) | 0.67 (0.45 – 0.94) | 0.4798 | 0.16 (0.08 – 0.32) | 0.25 (0.13 – 0.39) | 0.1338 |
|  | Occipital | 0.31 (0.24 – 0.37) | 0.46 (0.27 – 0.73) | **0.0032** | 0.26 (0.2 – 0.3) | 0.31 (0.14 – 0.5) | 0.2398 | 0.35 (0.28 – 0.45) | 0.66 (0.44 – 1.42) | **<0.001** | 0.3 (0.19 – 0.35) | 0.19 (0.13 – 0.33) | 0.0613 |
| MAD of HbO (au) | Frontal | 2.27 (1.53 – 3.85) | 3.26 (1.83 – 5.61) | 0.109 | 1.13 (0.76 – 2.17) | 1.38 (0.69 – 2.33) | 0.6867 | 3.46 (2.55 – 6.03) | 3.46 (2.65 – 7.49) | 0.5649 | 0.69 (0.36 – 0.99) | 0.9 (0.49 – 1.7) | 0.1374 |
|  | Parietal | 4.87 (2.2 – 13.95) | 3.94 (2.09 – 6.59) | 0.2426 | 2.02 (0.88 – 4.32) | 1.7 (0.73 – 3.6) | 0.4884 | 7.87 (3.44 – 17.85) | 10.73 (5.63 – 18.34) | 0.2931 | 1.21 (0.59 – 3.14) | 1.42 (0.64 – 2.48) | 1 |
|  | Temporal | 4.92 (1.38 – 8.58) | 3.33 (2.27 – 9.1) | 0.8985 | 1.95 (0.83 – 3.9) | 2.3 (0.95 – 3.33) | 0.9862 | 6.72 (3.33 – 12.34) | 4.97 (3.28 – 11.6) | 0.4421 | 1.43 (0.48 – 2.74) | 1.51 (0.75 – 2.98) | 0.3647 |
|  | Occipital | 3.67 (2.97 – 5.32) | 4.15 (2.44 – 9.65) | 0.3907 | 2.65 (2.28 – 3.2) | 2.06 (1.03 – 3.86) | 0.0566 | 5.57 (3.48 – 7.8) | 10.88 (6.07 – 17.28) | **<0.001** | 3 (2.32 – 3.58) | 1.78 (0.72 – 3.59) | **0.01** |
| MAD of HHb (au) | Frontal | 1.59 (0.82 – 3.74) | 2.22 (1.35 – 6.91) | 0.0959 | 0.69 (0.39 – 1.35) | 0.88 (0.37 – 1.86) | 0.796 | 2.49 (1.25 – 4.28) | 3.23 (2.27 – 6.47) | **0.0172** | 0.49 (0.23 – 0.69) | 0.55 (0.22 – 1.53) | 0.4628 |
|  | Parietal | 4.26 (2.04 – 11.71) | 4.66 (1.63 – 8.95) | 0.5103 | 1.77 (0.77 – 3.57) | 1.49 (0.81 – 3.72) | 0.7329 | 7.62 (3.01 – 13.83) | 9.07 (4.35 – 14.93) | 0.3259 | 1 (0.41 – 2.47) | 1.2 (0.6 – 2.66) | 0.5695 |
|  | Temporal | 3.68 (1.57 – 7.34) | 3.78 (2.01 – 6.9) | 0.7643 | 1.87 (0.67 – 4.05) | 1.7 (0.54 – 2.65) | 0.3794 | 4.98 (2.56 – 9.25) | 4.35 (2.42 – 9.18) | 0.8067 | 1.14 (0.37 – 2.17) | 1.42 (0.51 – 2.58) | 0.3158 |
|  | Occipital | 2.46 (1.92 – 3.39) | 3.11 (1.72 – 7.62) | 0.2109 | 1.94 (1.7 – 2.2) | 1.82 (0.68 – 3.71) | 0.5932 | 2.99 (2.26 – 3.7) | 8.93 (4.49 – 15.07) | **<0.001** | 2.11 (1.78 – 2.41) | 1.43 (0.41 – 3.2) | 0.1184 |
| MAD of tHb (au) | Frontal | 3 (1.67 – 6.14) | 5.92 (2.3 – 9.65) | 0.0893 | 1.52 (0.93 – 3.01) | 1.57 (0.78 – 3.41) | 0.9478 | 5.61 (3.43 – 11.13) | 6.4 (3.4 – 13.43) | 0.5556 | 0.8 (0.43 – 1.63) | 0.97 (0.45 – 1.91) | 0.6666 |
|  | Parietal | 7.69 (2.04 – 21.66) | 4.87 (1.69 – 11.86) | 0.1586 | 2.33 (1.05 – 5.28) | 1.61 (0.72 – 4.64) | 0.2185 | 12.5 (6.82 – 27.98) | 14.29 (9.54 – 32.18) | 0.3907 | 1.15 (0.56 – 2.33) | 1.65 (0.88 – 2.91) | 0.2626 |
|  | Temporal | 5.93 (2.2 – 14.24) | 6.63 (2.42 – 14.19) | 0.7538 | 1.76 (1.05 – 6.28) | 1.98 (1.27 – 4.75) | 0.8442 | 10.25 (5.86 – 16.54) | 8.14 (4.28 – 18.96) | 0.467 | 1.45 (0.54 – 3.09) | 1.85 (1.19 – 2.93) | 0.2237 |
|  | Occipital | 3.14 (2.07 – 5.76) | 5.76 (3.13 – 17.87) | **0.0013** | 1.82 (1.51 – 2.42) | 2.8 (1.47 – 7.84) | **0.0423** | 5.36 (3.24 – 8.44) | 19.74 (9.41 – 32.39) | **<0.001** | 1.95 (1.59 – 2.37) | 2.09 (0.88 – 5.23) | 0.9478 |
| MAD of HbDiff (au) | Frontal | 1.87 (1.02 – 4.2) | 3.05 (1.61 – 4.94) | 0.1566 | 0.96 (0.64 – 1.86) | 1.27 (0.66 – 3.01) | 0.2568 | 2.55 (1.7 – 4.16) | 3.27 (2.48 – 4.96) | **0.0262** | 0.58 (0.44 – 1.2) | 0.99 (0.44 – 1.84) | 0.1234 |
|  | Parietal | 3.98 (2.13 – 8.83) | 3.25 (2.09 – 10.73) | 1 | 2.22 (1.16 – 5.71) | 2.82 (0.92 – 4.16) | 0.6666 | 5.41 (4.04 – 11.66) | 7.8 (4.22 – 15.53) | 0.2931 | 1.18 (0.68 – 3.73) | 1.39 (0.51 – 3.71) | 0.8335 |
|  | Temporal | 3.77 (1.28 – 7.02) | 3.86 (1.47 – 6.86) | 0.702 | 2.26 (1.01 – 4.62) | 1.99 (0.83 – 4.29) | 0.3574 | 4.6 (2.85 – 9.87) | 4.99 (3.43 – 8.29) | 0.8985 | 1.2 (0.47 – 2.88) | 1.72 (0.72 – 4.64) | 0.1487 |
|  | Occipital | 4.81 (3.92 – 5.94) | 3.3 (1.34 – 6.34) | **0.0291** | 4.06 (3.4 – 4.91) | 2.15 (0.89 – 4.29) | **<0.001** | 5.7 (4.97 – 8.66) | 5.22 (3.36 – 11.22) | 0.2837 | 4.81 (3.57 – 5.73) | 1.28 (0.78 – 3.75) | **<0.001** |
| **250 Hz Sampled Data** | | | | | | | | | | | | | |
| ABP (mmHg) | – | 95.13 (90.2 – 100.43) | | – | 97.3 (91.69 – 100.99) | | – | 95.18 (89.31 – 100.79) | | – | 95.42 (91.95 – 99.66) | | – |
| EtCO₂ (mmHg) | – | 35.68 (34.17 – 36.92) | | – | 35.85 (35 – 36.95) | | – | 35.46 (34.52 – 37.12) | | – | 28.99 (26.39 – 31.16) | | – |
| RR (bpm) | – | 17.91 (13.94 – 19.84) | | – | 18.57 (15.69 – 20) | | – | 17.41 (14.89 – 19.52) | | – | 14.23 (10.01 – 24.89) | | – |
| rSO_2_ (%) | Frontal | 45.83 (45.06 – 46.43) | 40.37 (39.44 – 40.91) | **<0.001** | 46.23 (45.78 – 46.45) | 40.33 (39.99 – 40.83) | **<0.001** | 45.27 (44.76 – 45.95) | 40.46 (39.37 – 41.21) | **<0.001** | 46.8 (46.26 – 47.09) | 40.68 (40.38 – 40.82) | **<0.001** |
|  | Parietal | 49.09 (47.5 – 50.62) | 46.05 (45.1 – 47.21) | **0.0064** | 48.6 (48.13 – 50.01) | 46.45 (46 – 46.86) | 0.0652 | 48.99 (47.29 – 50.56) | 45.23 (43.93 – 47.07) | **0.001** | 48.97 (48.38 – 49.28) | 44.74 (44.44 – 45.79) | **<0.001** |
|  | Temporal | 40.1 (38.84 – 41.27) | 48.01 (46.88 – 48.7) | **<0.001** | 40.04 (39.41 – 40.43) | 48.33 (47.69 – 48.59) | **<0.001** | 40.56 (39.23 – 42.02) | 47.77 (47.18 – 48.59) | **<0.001** | 40.31 (40.06 – 40.57) | 47.44 (47.16 – 47.78) | **<0.001** |
|  | Occipital | 36.13 (35.91 – 36.54) | 47.33 (45.77 – 47.99) | **<0.001** | 36.35 (36.01 – 36.59) | 47.6 (47.2 – 47.92) | **<0.001** | 36.08 (35.65 – 36.61) | 46.66 (45.84 – 47.66) | **<0.001** | 36.16 (35.77 – 36.43) | 47.04 (46.66 – 47.69) | **<0.001** |
| HbO (au) | Frontal | -117.3  (-128.87 – -114.1) | -133.55  (-137.51 – -123.28) | **0.0143** | -117.24  (-120.42 – -116.62) | -132.54  (-134.03 – -131.52) | **0.0125** | -121.08  (-125.29 – -117.44) | -126.42  (-135.83 – -124.46) | 0.1075 | -120.48  (-122.62 – -119.31) | -125.83  (-128.37 – -125.15) | 0.1448 |
|  | Parietal | -165.17  (-180.61 – -158.23) | -176.21  (-202.33 – -169.76) | 0.1822 | -168.69  (-174.99 – -163.39) | -175.37  (-176.72 – -174.07) | 0.2539 | -174.63  (-185.4 – -163.07) | -190.7  (-205.57 – -177.07) | 0.18 | -171.34  (-172.2 – -168.18) | -187.28  (-188.33 – -183.25) | 0.1302 |
|  | Temporal | -208.66  (-220.73 – -204.1) | -148.61  (-156.45 – -141.32) | **0.0098** | -207.99  (-211.81 – -207.17) | -148.95  (-149.57 – -146.17) | **0.0154** | -210.83  (-221.77 – -199.38) | -155.93  (-163.17 – -150.33) | **0.0296** | -216.12  (-220.15 – -215.36) | -155.09  (-155.95 – -154.22) | **0.0329** |
|  | Occipital | -501.41  (-507.4 – -481.81) | -187  (-194.6 – -176.66) | **<0.001** | -499.1  (-501.49 – -494.58) | -174.39  (-174.89 – -170.11) | **<0.001** | -500.39  (-506.51 – -491.11) | -194.36  (-214.87 – -181.76) | **<0.001** | -505.36  (-507.72 – -503.5) | -193.65  (-197.24 – -192.41) | **<0.001** |
| HHb (au) | Frontal | -109.85  (-116.18 – -105.73) | -91.66  (-100.23 – -79.26) | 0.132 | -108.29  (-110.98 – -107.28) | -93.01  (-95.01 – -91.14) | 0.1733 | -109.31  (-111.2 – -106.38) | -82.18  (-89.53 – -80.75) | 0.0714 | -110.89  (-111.61 – -110.23) | -86.87  (-91.1 – -82.72) | 0.0531 |
|  | Parietal | -176.39  (-195.01 – -159.08) | -178.82  (-187.94 – -147.84) | 0.8174 | -177.72  (-187.83 – -172.79) | -175.62  (-177.34 – -171.73) | 0.8767 | -164.14  (-175.85 – -158.37) | -157.71  (-176.81 – -150.58) | 0.5932 | -160.58  (-161.31 – -158.08) | -149.93  (-151.43 – -149.49) | 0.4971 |
|  | Temporal | -140.14  (-160.32 – -131.77) | -146.57  (-167.39 – -135.83) | 0.5372 | -144.75  (-149.78 – -139.71) | -147.19  (-150.7 – -143.35) | 0.5418 | -144.18  (-158.52 – -134.82) | -145.47  (-152.06 – -137.97) | 0.627 | -152.98  (-162.34 – -149.88) | -149.45  (-150.31 – -147.07) | 0.5742 |
|  | Occipital | -274.83  (-280.64 – -269.73) | -159.58  (-168.98 – -155.1) | **<0.001** | -276.82  (-279.09 – -274.19) | -157.65  (-160.22 – -155.31) | **<0.001** | -273.71  (-279.74 – -269.47) | -174.55  (-186.73 – -159.36) | **<0.001** | -275.87  (-278.89 – -273.66) | -172.92  (-173.12 – -172.33) | **<0.001** |
| tHb (au) | Frontal | -221.89  (-238.08 – -216.03) | -226.25  (-243.49 – -202.88) | 0.9588 | -221.9  (-235.68 – -220.39) | -223.49  (-226.67 – -222.12) | 0.8227 | -227.99  (-238.34 – -221.71) | -209.73  (-231.51 – -205.2) | 0.5979 | -225.16  (-227.5 – -222.14) | -213.35  (-216.29 – -212.64) | 0.4503 |
|  | Parietal | -340.24  (-378.08 – -319.41) | -342.9  (-387.8 – -326.79) | 0.5979 | -344.34  (-360.56 – -334.19) | -347.28  (-349.7 – -340.21) | 0.5837 | -336.2  (-353.96 – -325.77) | -349.25  (-372.75 – -332.87) | 0.5418 | -332.76  (-333.75 – -330.86) | -341.84  (-343.21 – -334.78) | 0.5103 |
|  | Temporal | -352.2  (-381.13 – -339.07) | -297.34  (-325.66 – -277.77) | 0.2482 | -350.89  (-354.93 – -349.79) | -294.74  (-300.2 – -288.82) | 0.2806 | -360.32  (-382.64 – -342.16) | -308.72  (-319.91 – -295.6) | 0.3328 | -365.25  (-375.49 – -364.44) | -306.91  (-308.02 – -305.49) | 0.3328 |
|  | Occipital | -779.05  (-795.52 – -755.21) | -345.59  (-367.74 – -336.17) | **<0.001** | -774.39  (-778.46 – -771.43) | -330.91  (-338.64 – -324.76) | **<0.001** | -771.8  (-785.09 – -760.97) | -373.68  (-403.69 – -344.94) | **<0.001** | -786.93  (-792.08 – -783.24) | -362.97  (-365.25 – -361.14) | **<0.001** |
| HbDiff (au) | Frontal | -19.93  (-22.18 – -15.9) | -43.41  (-47.5 – -38.24) | **<0.001** | -17.5  (-18.29 – -16.53) | -43.34  (-45.21 – -41.56) | **<0.001** | -19.79  (-21.6 – -16.18) | -44.61  (-48.46 – -41.52) | **<0.001** | -15.66  (-17.03 – -14.71) | -43.2  (-43.5 – -41.58) | **<0.001** |
|  | Parietal | -5.88  (-13.48 – 3.1) | -22.6  (-30.82 – -17.77) | **0.0125** | -7.78  (-8.22 – -0.18) | -22.09  (-23.09 – -19.11) | 0.1016 | -8.33  (-15.98 – 3.69) | -31.75  (-38.86 – -19.26) | **<0.001** | -8.5  (-9.51 – -4) | -30.29  (-32.66 – -27.48) | **<0.001** |
|  | Temporal | -54.21  (-72.56 – -50.93) | -12.59  (-17.21 – -5.99) | **<0.001** | -59.26  (-65.38 – -56.74) | -10.89  (-13.47 – -10.24) | **<0.001** | -60.8  (-66.96 – -55.03) | -10.58  (-16.25 – -5.87) | **<0.001** | -59.51  (-60.58 – -55.23) | -12.15  (-12.8 – -11.51) | **<0.001** |
|  | Occipital | -215.97  (-223.82 – -210.98) | -18.82  (-25.31 – -11.67) | **<0.001** | -215.01  (-219.74 – -211.72) | -15.82  (-18.55 – -13.82) | **<0.001** | -223.03  (-229.27 – -212.32) | -20.74  (-28.46 – -16.38) | **<0.001** | -224.59  (-229.69 – -221.19) | -20.07  (-22.85 – -18.82) | **<0.001** |
| MAD of ABP (mmHg) | – | 4.64 (3.27 – 5.85) | | – | 4.44 (3.5 – 5.06) | | – | 5.29 (4.59 – 6.53) | | – | 3.54 (2.79 – 4.67) | | – |
| MAD of EtCO₂ (mmHg) | – | 1.03 (0.81 – 1.38) | | – | 0.84 (0.6 – 1) | | – | 1.18 (0.94 – 1.42) | | – | 1.72 (1.2 – 2.57) | | – |
| MAD of RR (bpm) | – | 2.01 (1.17 – 2.8) | | – | 1.69 (1.07 – 2.22) | | – | 2.08 (1.75 – 2.71) | | – | 5.28 (2.03 – 6.37) | | – |
| MAD of rSO_2_ (%) | Frontal | 0.43 (0.22 – 0.79) | 0.5 (0.33 – 1.22) | 0.1712 | 0.2 (0.13 – 0.4) | 0.21 (0.14 – 0.51) | 0.6969 | 0.5 (0.36 – 0.76) | 0.79 (0.49 – 1.07) | **0.0031** | 0.13 (0.08 – 0.3) | 0.2 (0.09 – 0.44) | 0.1733 |
|  | Parietal | 0.51 (0.31 – 1.11) | 0.61 (0.26 – 1.4) | **0.9808** | 0.37 (0.2 – 0.59) | 0.32 (0.13 – 0.6) | **0.5282** | 1.02 (0.65 – 1.48) | 1.18 (0.66 – 1.75) | 0.6027 | 0.19 (0.14 – 0.44) | 0.17 (0.09 – 0.4) | 0.4023 |
|  | Temporal | 0.51 (0.22 – 0.86) | 0.45 (0.31 – 0.82) | 0.7381 | 0.3 (0.16 – 0.47) | 0.26 (0.15 – 0.41) | 0.627 | 0.74 (0.49 – 1.09) | 0.69 (0.45 – 0.94) | 0.5327 | 0.17 (0.08 – 0.36) | 0.25 (0.13 – 0.36) | 0.1627 |
|  | Occipital | 0.32 (0.26 – 0.37) | 0.46 (0.26 – 0.74) | **0.008** | 0.27 (0.22 – 0.32) | 0.3 (0.14 – 0.5) | **0.3683** | 0.36 (0.3 – 0.45) | 0.66 (0.44 – 1.44) | **<0.001** | 0.3 (0.2 – 0.36) | 0.19 (0.13 – 0.33) | **0.0207** |
| MAD of HbO (au) | Frontal | 2.3 (1.44 – 3.83) | 3.07 (1.96 – 5.78) | 0.1566 | 1.1 (0.79 – 2.15) | 1.36 (0.68 – 2.35) | 0.7071 | 3.4 (2.55 – 6.25) | 3.52 (2.68 – 7.41) | 0.5147 | 0.67 (0.38 – 1.02) | 0.91 (0.49 – 1.77) | 0.1712 |
|  | Parietal | 4.83 (2.28 – 14.04) | 3.57 (1.94 – 6.67) | **0.2083** | 2.07 (0.9 – 4.38) | 1.66 (0.68 – 3.53) | **0.3946** | 7.87 (3.47 – 17.92) | 10.75 (5.66 – 18.3) | 0.2837 | 1.24 (0.6 – 2.83) | 1.34 (0.72 – 3.07) | 0.9533 |
|  | Temporal | 4.9 (1.41 – 8.49) | 3.29 (2.26 – 8.37) | 0.9588 | 1.95 (0.83 – 3.69) | 2.3 (0.98 – 3.37) | 0.9478 | 6.71 (3.29 – 12.43) | 4.98 (3.31 – 11.57) | 0.467 | 1.39 (0.5 – 2.75) | 1.47 (0.75 – 2.96) | 0.3192 |
|  | Occipital | 3.64 (2.7 – 5.34) | 4.06 (2.43 – 9.98) | **0.2626** | 2.67 (2.22 – 3.1) | 2.02 (1 – 3.95) | **0.0893** | 5.44 (3.38 – 7.81) | 10.96 (5.97 – 17.15) | **<0.001** | 2.81 (2.33 – 3.34) | 1.83 (0.63 – 3.67) | **0.0204** |
| MAD of HHb (au) | Frontal | 1.67 (0.73 – 3.77) | 2.32 (1.3 – 6.45) | 0.1184 | 0.66 (0.39 – 1.46) | 0.87 (0.37 – 1.82) | 0.8067 | 2.5 (1.28 – 4.1) | 3.25 (2.21 – 6.46) | 0.0189 | 0.49 (0.23 – 0.66) | 0.53 (0.21 – 1.47) | 0.5372 |
|  | Parietal | 4.59 (1.93 – 11.3) | 4.6 (1.82 – 9.08) | 0.6417 | 1.77 (0.77 – 3.65) | 1.67 (0.8 – 3.69) | **0.7748** | 7.6 (3 – 13.78) | 8.84 (4.22 – 14.93) | 0.3397 | 0.98 (0.44 – 2.7) | 1.19 (0.6 – 2.92) | 0.6172 |
|  | Temporal | 3.7 (1.52 – 8.23) | 3.93 (1.84 – 6.78) | 0.812 | 2.02 (0.65 – 4.05) | 1.76 (0.54 – 2.66) | 0.372 | 4.95 (2.63 – 9.35) | 4.41 (2.5 – 9.09) | 0.8767 | 1.08 (0.38 – 2.34) | 1.41 (0.48 – 2.6) | 0.4219 |
|  | Occipital | 2.52 (1.91 – 3.32) | 3.15 (1.75 – 7.93) | **0.1891** | 1.91 (1.75 – 2.21) | 1.78 (0.66 – 3.81) | **0.5015** | 3 (2.26 – 3.69) | 8.88 (4.46 – 14.46) | **<0.001** | 2.09 (1.78 – 2.34) | 1.39 (0.38 – 3.33) | 0.1606 |
| MAD of tHb (au) | Frontal | 2.94 (1.65 – 7.06) | 5.4 (2.11 – 10.28) | 0.1627 | 1.52 (0.93 – 2.99) | 1.59 (0.78 – 3.43) | 0.9808 | 5.63 (3.42 – 11.05) | 6.42 (3.44 – 13.44) | 0.5059 | 0.75 (0.43 – 1.49) | 0.92 (0.42 – 1.95) | 0.7433 |
|  | Parietal | 7.55 (2.27 – 22.1) | 4.31 (1.59 – 12.8) | **0.1392** | 2.27 (1.02 – 5.13) | 1.61 (0.73 – 4.62) | **0.2482** | 12.37 (6.75 – 28.32) | 14.19 (9.45 – 32.3) | 0.3502 | 1.14 (0.53 – 2.57) | 1.64 (0.96 – 2.87) | 0.2371 |
|  | Temporal | 5.56 (2.48 – 15.18) | 6.51 (2.43 – 13.93) | 0.702 | 1.76 (1.02 – 6.15) | 2.01 (1.35 – 4.77) | 0.8822 | 10.03 (5.94 – 16.73) | 8.13 (4.3 – 18.94) | 0.4586 | 1.45 (0.53 – 3.36) | 1.8 (1.18 – 2.87) | 0.2685 |
|  | Occipital | 3.09 (2.08 – 5.66) | 5.95 (3.27 – 16.77) | **<0.001** | 1.8 (1.57 – 2.46) | 2.84 (1.45 – 7.74) | **0.046** | 5.48 (3.08 – 8.3) | 19.61 (9.05 – 32.08) | **<0.001** | 1.96 (1.51 – 2.52) | 1.91 (0.88 – 5.61) | 0.9588 |
| MAD of HbDiff (au) | Frontal | 1.83 (1.06 – 4.22) | 2.98 (1.61 – 5.11) | 0.18 | 0.98 (0.63 – 1.85) | 1.28 (0.65 – 3.03) | 0.2454 | 2.54 (1.65 – 4.12) | 3.29 (2.5 – 4.91) | **0.0276** | 0.56 (0.44 – 1.22) | 0.97 (0.46 – 1.89) | 0.132 |
|  | Parietal | 4.05 (1.74 – 9.75) | 3.89 (2.11 – 10.14) | **0.8876** | 2.21 (1.13 – 5.75) | 2.76 (0.97 – 4.2) | **0.759** | 5.45 (4.24 – 11.57) | 7.83 (4.21 – 15.61) | 0.3027 | 1.23 (0.66 – 3.73) | 1.41 (0.58 – 3.83) | 0.812 |
|  | Temporal | 3.51 (1.28 – 7.47) | 3.8 (1.42 – 7.52) | 0.6918 | 2.3 (1.01 – 4.52) | 1.97 (0.8 – 4.54) | 0.4023 | 4.52 (2.89 – 9.85) | 4.79 (3.38 – 8.51) | 0.9259 | 1.2 (0.49 – 2.97) | 1.67 (0.73 – 4.27) | 0.169 |
|  | Occipital | 4.68 (3.79 – 5.85) | 3.38 (1.43 – 6) | **0.0111** | 4.19 (3.59 – 4.8) | 2.17 (0.94 – 4.39) | **<0.001** | 5.61 (5.03 – 8.39) | 5.27 (3.38 – 11.25) | **0.2776** | 4.71 (3.64 – 5.45) | 1.33 (0.79 – 3.4) | **<0.001** |
| The p-values in the table are derived using Mann-Whitney U test between the bilateral signals. *ABP, arterial blood pressure; bpm, beats per minute; EtCO_2_, end-tidal carbon dioxide; HbDiff, hemoglobin difference; HbO, oxyhemoglobin; HHb, deoxyhemoglobin; IQR, interquartile range; MAD, median absolute deviation; mmHg, millimeters of mercury; RR, respiratory rate; rSO_2_, regional cerebral oxygen saturation; tHb, total hemoglobin.* | | | | | | | | | | | | | |

Appendix S7c: Perturbation Subgrouped Median and IQR of Physiologic Signals Using Raw Data3549

| **Physiologic Signal** | **Brain Lobe** | **Baseline** | | | **Neurovascular Coupling** | | | **Orthostatic Challenge** | | | **Vascular Chemo-Reactivity** | | |
| --- | --- | --- | --- | --- | --- | --- | --- | --- | --- | --- | --- | --- | --- |
|  |  | **Median (IQR)** | | **p-value** | **Median (IQR)** | | **p-value** | **Median (IQR)** | | **p-value** | **Median (IQR)** | | **p-value** |
|  |  | **Left Hemisphere** | **Right Hemisphere** |  | **Left Hemisphere** | **Right Hemisphere** |  | **Left Hemisphere** | **Right Hemisphere** |  | **Left Hemisphere** | **Right Hemisphere** |  |
| **1 Hz Sampled Data** | | | | | | | | | | | | | |
| ABP (mmHg) | – | 94.08 (88.73 – 100.69) | | – | 96.37 (89.92 – 100.71) | | – | 94.42 (87.92 – 101.09) | | – | 94.13 (89.76 – 99.23) | | – |
| EtCO₂ (mmHg) | – | 36 (33.94 – 37) | | – | 36 (35 – 37) | | – | 35.5 (34.51 – 37) | | – | 29 (26 – 31.5) | | – |
| RR (bpm) | – | 18 (14 – 20) | | – | 18.65 (15.5 – 20) | | – | 17.5 (14.7 – 19.5) | | – | 13 (10 – 25) | | – |
| rSO_2_ (%) | Frontal | 45.72 (45.06 – 46.45) | 40.36 (39.45 – 40.92) | **<0.001** | 46.19 (45.46 – 46.44) | 40.49 (39.97 – 40.85) | **<0.001** | 45.33 (44.76 – 45.95) | 40.46 (39.39 – 41.22) | **<0.001** | 46.81 (46.26 – 47.08) | 40.67 (40.39 – 40.82) | **<0.001** |
|  | Parietal | 49.19 (47.55 – 50.62) | 46.01 (45.15 – 47.22) | **0.0055** | 48.62 (48.11 – 49.96) | 46.47 (46.01 – 46.9) | 0.0531 | 48.96 (47.21 – 50.58) | 45.33 (43.91 – 47.02) | **0.001** | 49 (48.26 – 49.38) | 44.83 (44.42 – 45.83) | **<0.001** |
|  | Temporal | 40.09 (38.81 – 41.31) | 48 (46.67 – 48.71) | **<0.001** | 40.04 (39.41 – 40.46) | 48.33 (47.69 – 48.62) | **<0.001** | 40.55 (39.18 – 41.94) | 47.77 (47.15 – 48.59) | **<0.001** | 40.21 (40.05 – 40.57) | 47.43 (47.13 – 47.76) | **<0.001** |
|  | Occipital | 35.89 (35.11 – 36.68) | 47.31 (45.8 – 48.08) | **<0.001** | 36.05 (35.24 – 36.64) | 47.6 (47.17 – 47.92) | **<0.001** | 35.84 (34.9 – 36.84) | 46.65 (45.77 – 47.66) | **<0.001** | 35.88 (34.98 – 36.73) | 47.05 (46.63 – 47.6) | **<0.001** |
| HbO (au) | Frontal | -117.41  (-128.89 – -114.09) | -133.59  (-137.18 – -123.24) | **0.0169** | -117.25  (-120.41 – -116.59) | -132.7  (-134.59 – -130.92) | **0.0127** | -121  (-125.49 – -117.33) | -126.4  (-135.79 – -124.39) | 0.1105 | -120.44  (-122.77 – -119.33) | -125.64  (-128.38 – -125.19) | 0.1448 |
|  | Parietal | -164.1  (-181.01 – -157.86) | -176.24  (-200.77 – -168.35) | 0.1627 | -168.68  (-175.41 – -163.34) | -175.33  (-176.87 – -174) | 0.2482 | -174.65  (-185.35 – -162.08) | -190.12  (-206.24 – -176.85) | 0.1733 | -171.32  (-172.16 – -168.37) | -187.22  (-188.35 – -182.52) | 0.1251 |
|  | Temporal | -208.7  (-220.24 – -203.4) | -147.4  (-154.97 – -141.39) | **0.0102** | -208.11  (-211.9 – -207.13) | -148.98  (-149.57 – -146.24) | **0.0148** | -210.78  (-221.35 – -199.15) | -155.96  (-163.12 – -150.38) | **0.0291** | -216.1  (-220.16 – -215.18) | -155.1  (-155.89 – -154.12) | **0.0329** |
|  | Occipital | -497.23  (-507.19 – -478.77) | -186.99  (-194.67 – -174.62) | **<0.001** | -499.09  (-504.83 – -490.9) | -174.31  (-175.08 – -170.15) | **<0.001** | -498.74  (-510.74 – -487.67) | -195.11  (-216.16 – -181.02) | **<0.001** | -505.45  (-510.61 – -499.5) | -193.74  (-198.31 – -192.33) | **<0.001** |
| HHb (au) | Frontal | -109.98  (-116.17 – -105.67) | -91.66  (-100.24 – -79.68) | 0.1338 | -108.31  (-110.98 – -107.23) | -92.73  (-95.01 – -91.13) | 0.1712 | -109.28  (-111.29 – -105.91) | -82.13  (-89.62 – -80.71) | 0.0704 | -110.88  (-111.66 – -110.14) | -87.34  (-91.51 – -82.71) | 0.0557 |
|  | Parietal | -176.94  (-195.87 – -157.55) | -179.35  (-188.29 – -147.72) | 0.7643 | -177  (-188.25 – -172.85) | -175.7  (-177.44 – -171.71) | 0.8605 | -163.76  (-174.71 – -157.68) | -157.85  (-177.2 – -150.28) | 0.5884 | -160.71  (-162.29 – -159.08) | -149.97  (-152.04 – -149.44) | 0.4971 |
|  | Temporal | -140.05  (-160 – -131.29) | -148.12  (-163.05 – -135.62) | 0.5742 | -144.73  (-149.74 – -139.27) | -147.18  (-150.1 – -143.34) | 0.5418 | -144.13  (-158.99 – -134.76) | -145.53  (-152.01 – -137.36) | 0.6221 | -152.86  (-162.21 – -149.98) | -149.57  (-150.35 – -147.1) | 0.5695 |
|  | Occipital | -274.43  (-281.92 – -266.59) | -160.17  (-169 – -155.17) | **<0.001** | -276.9  (-281.87 – -270.88) | -157.53  (-160.25 – -155.27) | **<0.001** | -273.3  (-281.05 – -267.33) | -174.56  (-186.34 – -158.96) | **<0.001** | -276.28  (-281.33 – -271.66) | -172.85  (-173.14 – -171.89) | **<0.001** |
| tHb (au) | Frontal | -221.67  (-238.28 – -216.27) | -226.55  (-243.08 – -202.23) | 0.9533 | -221.86  (-235.66 – -220.31) | -223.34  (-226.62 – -221.91) | 0.8335 | -227.78  (-238.5 – -221.63) | -209.78  (-232.94 – -205.09) | 0.5837 | -225.19  (-227.38 – -222.19) | -213.33  (-216.46 – -212.57) | 0.4586 |
|  | Parietal | -340.22  (-378.85 – -319.29) | -342.46  (-388.48 – -323.91) | 0.5418 | -341.84  (-360.85 – -333.59) | -347.24  (-349.73 – -339.91) | 0.5742 | -335.28  (-354.16 – -325.61) | -349.59  (-372.79 – -332.74) | 0.5237 | -332.92  (-334.09 – -331.01) | -341.72  (-343.28 – -334.8) | 0.5192 |
|  | Temporal | -352.49  (-381.18 – -338.74) | -301.64  (-320.19 – -277.71) | 0.2398 | -350.93  (-354.88 – -349.83) | -294.83  (-300.06 – -289.05) | 0.2776 | -360.4  (-382.55 – -342.12) | -308.63  (-319.98 – -295.38) | 0.3432 | -365.31  (-375.42 – -364.26) | -307.01  (-308.14 – -305.21) | 0.3362 |
|  | Occipital | -777.18  (-796.25 – -751.72) | -346.8  (-367.94 – -334.85) | **<0.001** | -774.85  (-779.23 – -770.43) | -330.93  (-338.63 – -324.46) | **<0.001** | -771.52  (-784.7 – -759.89) | -376.97  (-403.94 – -344.56) | **<0.001** | -787.63  (-793.51 – -781.28) | -362.98  (-365.03 – -361.15) | **<0.001** |
| HbDiff (au) | Frontal | -19.9  (-22.14 – -15.7) | -43.42  (-47.5 – -38.18) | **<0.001** | -17.47  (-18.28 – -15.12) | -42.98  (-45.21 – -40.92) | **<0.001** | -19.8  (-21.58 – -16.17) | -44.6  (-48.7 – -41.48) | **<0.001** | -15.63  (-17.06 – -14.63) | -42.26  (-43.62 – -41.63) | **<0.001** |
|  | Parietal | -5.24  (-14.85 – 3.14) | -22.68  (-31 – -17.83) | **0.0151** | -7.77  (-8.93 – -0.41) | -21.86  (-23.16 – -19.15) | 0.0959 | -8.2  (-16.24 – 4.04) | -30.62  (-39.31 – -19.33) | **0.001** | -7.7  (-10.17 – -3.47) | -30.09  (-33.18 – -26.6) | **<0.001** |
|  | Temporal | -54.24  (-72.54 – -50.52) | -12.65  (-17.36 – -5.94) | **<0.001** | -59.67  (-66.35 – -55.99) | -10.96  (-13.48 – -10.21) | **<0.001** | -60.82  (-66.96 – -55.5) | -10.84  (-16.29 – -5.76) | **<0.001** | -58.92  (-60.51 – -54.77) | -12.15  (-12.81 – -10.37) | **<0.001** |
|  | Occipital | -217.27  (-228.2 – -204.13) | -17.74  (-25.89 – -11.03) | **<0.001** | -215.52  (-226.77 – -204.11) | -15.73  (-18.7 – -13.62) | **<0.001** | -222.28  (-234.45 – -205.03) | -20.65  (-28.85 – -16.55) | **<0.001** | -225.53  (-239.72 – -211.59) | -19.81  (-23.02 – -18.81) | **<0.001** |
| MAD of ABP (mmHg) | – | 5.2 (4.3 – 7.6) | | – | 5.15 (4.57 – 6.04) | | – | 6.17 (5.37 – 7.19) | | – | 4.79 (3.92 – 5.56) | | – |
| MAD of EtCO₂ (mmHg) | – | 1 (1 – 1.69) | | – | 1 (1 – 1) | | – | 1 (1 – 2) | | – | 2 (1 – 2.67) | | – |
| MAD of RR (bpm) | – | 2 (1.09 – 3) | | – | 2 (1 – 2) | | – | 2 (2 – 3) | | – | 5 (2 – 6) | | – |
| MAD of rSO_2_ (%) | Frontal | 0.43 (0.23 – 0.97) | 0.76 (0.37 – 1.19) | 0.1268 | 0.2 (0.14 – 0.45) | 0.24 (0.15 – 0.57) | 0.7381 | 0.53 (0.39 – 0.84) | 0.83 (0.51 – 1.17) | **0.0034** | 0.15 (0.09 – 0.29) | 0.22 (0.09 – 0.53) | 0.1755 |
|  | Parietal | 0.84 (0.44 – 1.67) | 0.85 (0.37 – 1.49) | 0.5372 | 0.63 (0.34 – 1.04) | 0.48 (0.17 – 0.72) | 0.1001 | 1.16 (0.81 – 1.74) | 1.24 (0.77 – 1.78) | 0.904 | 0.38 (0.19 – 0.84) | 0.27 (0.11 – 0.71) | 0.1001 |
|  | Temporal | 0.68 (0.35 – 1.43) | 0.83 (0.36 – 1.28) | 0.6368 | 0.39 (0.22 – 0.92) | 0.4 (0.16 – 0.86) | 0.7329 | 0.82 (0.59 – 1.39) | 0.9 (0.58 – 1.15) | 0.7485 | 0.24 (0.1 – 0.53) | 0.28 (0.13 – 0.86) | 0.2263 |
|  | Occipital | 0.81 (0.59 – 0.91) | 0.56 (0.31 – 1.09) | 0.1962 | 0.76 (0.55 – 0.86) | 0.4 (0.17 – 0.82) | **0.0016** | 0.87 (0.64 – 1.04) | 0.82 (0.5 – 1.58) | 0.9588 | 0.9 (0.57 – 1.06) | 0.25 (0.15 – 0.69) | **<0.001** |
| MAD of HbO (au) | Frontal | 2.54 (1.51 – 4.36) | 3.58 (1.89 – 6.32) | 0.2745 | 1.15 (0.86 – 2.31) | 1.59 (0.69 – 2.75) | 0.6969 | 3.49 (2.6 – 7.14) | 3.9 (2.71 – 7.71) | 0.5932 | 0.79 (0.42 – 1.13) | 1 (0.57 – 1.87) | 0.2134 |
|  | Parietal | 6.07 (3.73 – 16.64) | 4.86 (2.64 – 7.83) | 0.1121 | 3.3 (0.98 – 7.46) | 2.61 (0.78 – 4.52) | 0.1712 | 8.72 (5.68 – 18.57) | 10.9 (5.93 – 18.22) | 0.3647 | 1.89 (0.77 – 4.15) | 1.65 (0.73 – 4.7) | 0.5695 |
|  | Temporal | 6.11 (1.67 – 10.67) | 6.21 (2.36 – 9.84) | 0.8388 | 2.1 (0.9 – 8.32) | 2.76 (0.99 – 6.11) | 0.904 | 8.26 (4.28 – 13.26) | 8.04 (3.71 – 12.17) | 0.7071 | 1.63 (0.61 – 3.83) | 1.64 (0.76 – 4.96) | 0.4798 |
|  | Occipital | 8.72 (7.42 – 9.7) | 5 (3.27 – 11.97) | **0.0231** | 7.91 (5.68 – 8.43) | 2.78 (1.22 – 5.5) | **<0.001** | 10.2 (8.94 – 11.85) | 11.27 (6.85 – 19.31) | 0.4628 | 9.17 (5.53 – 10.12) | 2.11 (0.81 – 5.69) | **<0.001** |
| MAD of HHb (au) | Frontal | 1.9 (0.8 – 4.73) | 2.47 (1.25 – 6.94) | 0.1669 | 0.71 (0.4 – 1.39) | 0.87 (0.39 – 2.13) | 0.8013 | 2.69 (1.3 – 4.97) | 3.31 (2.4 – 6.63) | **0.0383** | 0.52 (0.28 – 0.97) | 0.57 (0.25 – 2.13) | 0.4971 |
|  | Parietal | 6.13 (2.86 – 11.94) | 5.57 (2.11 – 9.19) | 0.4061 | 2.87 (1.09 – 5.85) | 2.77 (0.84 – 5.72) | 0.4755 | 8.37 (5.27 – 13.81) | 9.34 (5.54 – 15.02) | 0.4462 | 1.99 (0.84 – 4.67) | 1.45 (0.61 – 3.91) | 0.6221 |
|  | Temporal | 5.89 (1.72 – 11.15) | 5.58 (3.04 – 7.96) | 0.7329 | 3.23 (0.87 – 6.06) | 3.19 (0.62 – 5.73) | 0.414 | 6.83 (3.65 – 10.02) | 6.4 (3.85 – 9.93) | 0.7277 | 1.79 (0.47 – 4.27) | 1.47 (0.55 – 5.8) | 0.5837 |
|  | Occipital | 6.17 (5.46 – 6.69) | 4.76 (1.97 – 9.07) | 0.2109 | 5.75 (4.9 – 6) | 2.22 (0.74 – 5.97) | **<0.001** | 6.83 (6.31 – 7.76) | 9.44 (5.03 – 15.88) | **0.0044** | 6.66 (5.57 – 7.15) | 2.41 (0.48 – 5.45) | **<0.001** |
| MAD of tHb (au) | Frontal | 3.21 (1.8 – 7.03) | 5.94 (2.5 – 10.8) | 0.106 | 1.69 (0.99 – 3.12) | 1.78 (0.82 – 3.78) | 0.9917 | 5.87 (3.57 – 11.38) | 6.32 (3.64 – 13.52) | 0.6124 | 0.86 (0.5 – 1.77) | 0.98 (0.51 – 2.44) | 0.812 |
|  | Parietal | 8.44 (3.37 – 21.9) | 4.92 (1.91 – 14.18) | 0.0867 | 3.01 (1.38 – 6.15) | 2.29 (0.76 – 5.77) | 0.2426 | 12.95 (6.98 – 27.64) | 14.27 (9.79 – 31.08) | 0.3538 | 1.84 (0.74 – 4.07) | 1.97 (0.98 – 4.26) | 0.7643 |
|  | Temporal | 7.24 (2.97 – 14.22) | 7.35 (4.45 – 13.39) | 0.9862 | 4.61 (1.3 – 6.61) | 3.44 (1.38 – 5.49) | 0.5932 | 11.45 (6.52 – 17.62) | 8.33 (5.55 – 19.44) | 0.4023 | 2.05 (0.63 – 5.02) | 2.45 (1.23 – 4.97) | 0.3794 |
|  | Occipital | 6.26 (4.95 – 8.27) | 7.31 (4.07 – 17.76) | 0.434 | 4.81 (3.52 – 5.1) | 3.64 (1.61 – 7.87) | 0.1487 | 8.12 (5.59 – 10.96) | 19.53 (9.1 – 31.69) | **<0.001** | 5.59 (3.88 – 6.03) | 3.26 (0.92 – 6.36) | **0.0307** |
| MAD of HbDiff (au) | Frontal | 1.89 (1.13 – 4.39) | 3.13 (1.58 – 5.46) | 0.2211 | 1 (0.72 – 2.24) | 1.29 (0.66 – 3.18) | 0.2344 | 2.54 (1.7 – 4.15) | 3.44 (2.62 – 5.3) | **0.0302** | 0.67 (0.5 – 1.34) | 1.05 (0.56 – 2.27) | 0.1302 |
|  | Parietal | 6.46 (2.76 – 13.03) | 6.67 (2.36 – 12.32) | 0.6516 | 4.61 (1.34 – 9.82) | 3.72 (1.16 – 6.93) | 0.2837 | 7.81 (4.66 – 12.8) | 9.23 (4.74 – 15.7) | 0.5837 | 2.09 (0.93 – 6.26) | 1.88 (0.59 – 4.7) | 0.2656 |
|  | Temporal | 4.92 (1.39 – 13.62) | 6.15 (1.59 – 13.61) | 0.7381 | 2.53 (1.18 – 12.54) | 2.39 (0.87 – 9.34) | 0.4219 | 5.36 (3.1 – 14) | 5 (3.38 – 14.72) | 0.8876 | 1.26 (0.68 – 5.69) | 1.78 (0.79 – 8.62) | 0.3907 |
|  | Occipital | 13.36 (10.5 – 14.54) | 4.71 (1.57 – 8.16) | **<0.001** | 12.76 (9.82 – 13.48) | 2.57 (0.97 – 7.52) | **<0.001** | 14.94 (12.55 – 16.09) | 6.07 (3.64 – 14.09) | **<0.001** | 14.54 (10.39 – 16.32) | 1.81 (0.81 – 6.15) | **<0.001** |
| **250 Hz Sampled Data** | | | | | | | | | | | | | |
| ABP (mmHg) | – | 92.51 (81.02 – 107.57) | | – | 92.83 (81.89 – 109.1) | | – | 92.45 (80.76 – 108.06) | | – | 91.77 (81.31 – 107.32) | | – |
| EtCO₂ (mmHg) | – | 36 (33.5 – 37) | | – | 36 (35 – 37) | | – | 35.5 (34.5 – 37) | | – | 29 (26 – 31.5) | | – |
| RR (bpm) | – | 18 (14 – 20) | | – | 19 (15.5 – 20) | | – | 17.5 (15 – 19.5) | | – | 13 (10 – 25) | | – |
| rSO_2_ (%) | Frontal | 45.81 (44.91 – 46.47) | 40.31 (39.39 – 41.36) | **<0.001** | 46.19 (45.4 – 46.46) | 40.48 (39.59 – 40.95) | **<0.001** | 45.36 (44.33 – 46.02) | 40.47 (39.43 – 41.45) | **<0.001** | 46.81 (45.93 – 47.12) | 40.66 (40.38 – 41) | **<0.001** |
|  | Parietal | 49.48 (46.56 – 51.9) | 46.04 (44.76 – 47.38) | **0.0034** | 48.55 (46.85 – 51.39) | 46.34 (45.69 – 47.16) | 0.0566 | 48.64 (46.49 – 51.76) | 45.28 (43.65 – 47.27) | **<0.001** | 49.31 (46.82 – 51.5) | 45.03 (44.09 – 46.11) | **<0.001** |
|  | Temporal | 40.13 (38.35 – 42.69) | 47.91 (45.99 – 48.73) | **<0.001** | 40.04 (38.25 – 42.81) | 48.43 (46.95 – 48.92) | **<0.001** | 40.41 (38.99 – 43.02) | 47.72 (46.24 – 48.57) | **<0.001** | 40.21 (39.87 – 42.05) | 47.39 (46.9 – 47.85) | **<0.001** |
|  | Occipital | 36.38 (30.42 – 41.59) | 47.24 (45.69 – 48.73) | **<0.001** | 36.39 (30.26 – 41.24) | 47.6 (46.52 – 48.37) | **<0.001** | 36.45 (30.09 – 41.36) | 46.67 (45.35 – 47.75) | **<0.001** | 36.41 (30.29 – 40.91) | 47.17 (45.94 – 48.02) | **<0.001** |
| HbO (au) | Frontal | -117.43  (-128.86 – -114.05) | -133.55  (-139.97 – -123.24) | **0.0151** | -117.29  (-120.42 – -116.55) | -133.89  (-136.51 – -130.11) | **0.0127** | -120.98  (-127.68 – -117.35) | -126.41  (-136.03 – -124.39) | 0.1121 | -120.42  (-122.77 – -119.24) | -125.62  (-129.72 – -125.22) | 0.1448 |
|  | Parietal | -166.17  (-181.61 – -154.43) | -176.42  (-201.02 – -168.39) | 0.1733 | -167.05  (-177.78 – -159.17) | -174.68  (-180.82 – -171.9) | 0.2626 | -173.32  (-186.1 – -160.73) | -189.74  (-206.1 – -176.82) | 0.1755 | -171.1  (-176.42 – -166.59) | -186.17  (-189.93 – -181.37) | 0.1302 |
|  | Temporal | -208.57  (-220.79 – -200.23) | -147.03  (-156.39 – -141.28) | **0.016** | -208.27  (-218.29 – -206.92) | -148.99  (-154.87 – -144.56) | **0.0145** | -210.56  (-223.2 – -198.15) | -156.02  (-163.15 – -149.35) | **0.0318** | -216.33  (-220.94 – -214.43) | -155.05  (-156.07 – -154.04) | **0.0445** |
|  | Occipital | -510.14  (-542.55 – -431.14) | -189.67  (-198.66 – -171.66) | **<0.001** | -516.53  (-544.84 – -434.68) | -173.85  (-179.94 – -169.13) | **<0.001** | -515.55  (-548.6 – -444.37) | -195.85  (-216.25 – -180.44) | **<0.001** | -524.78  (-555.4 – -450.85) | -193.8  (-198.99 – -187.16) | **<0.001** |
| HHb (au) | Frontal | -110.01  (-116.12 – -105.61) | -91.61  (-100.27 – -79.48) | 0.1302 | -108.31  (-111.05 – -106.27) | -92.78  (-95.03 – -91.08) | 0.1712 | -109.86  (-111.35 – -104.86) | -82.18  (-89.59 – -80.68) | 0.0704 | -110.88  (-113.18 – -110.11) | -87.31  (-91.69 – -82.65) | 0.0549 |
|  | Parietal | -179.61  (-198.51 – -157.97) | -179.48  (-188.85 – -147.59) | 0.6616 | -178.36  (-194.14 – -172.4) | -175.73  (-179.98 – -171.93) | 0.8822 | -166.87  (-183.18 – -153.99) | -158.04  (-177.39 – -149.62) | 0.5649 | -162.45  (-167.08 – -156.93) | -150.33  (-152.57 – -147.25) | 0.4928 |
|  | Temporal | -139.95  (-162.24 – -131.98) | -147.45  (-163.29 – -135.33) | 0.5059 | -144.91  (-149.68 – -139.01) | -146.92  (-154.5 – -143.31) | 0.4971 | -144.19  (-160.64 – -134.65) | -145.62  (-153.01 – -137.39) | 0.5979 | -153.14  (-161.96 – -143.32) | -149.52  (-150.42 – -147.15) | 0.5648 |
|  | Occipital | -288.73  (-310.5 – -229.2) | -160.6  (-170.58 – -154.19) | **<0.001** | -292.21  (-311.29 – -231.5) | -157.99  (-167.77 – -153.3) | **<0.001** | -286.84  (-311.58 – -228.8) | -174.51  (-186.69 – -158.73) | **<0.001** | -290.26  (-311.46 – -234.35) | -172.82  (-173.7 – -170.46) | **<0.001** |
| tHb (au) | Frontal | -221.12  (-238.27 – -216.28) | -226.49  (-243.09 – -202.13) | 0.9204 | -221.85  (-235.73 – -220.33) | -223.37  (-226.65 – -221.92) | 0.8227 | -227.72  (-238.39 – -221.57) | -209.74  (-232.89 – -205.08) | 0.5789 | -225.15  (-227.41 – -222.14) | -213.32  (-216.51 – -212.56) | 0.4421 |
|  | Parietal | -341.25  (-375.6 – -311.95) | -342.87  (-390.13 – -323.74) | 0.551 | -346.47  (-363.75 – -333.51) | -346.83  (-350.21 – -340.01) | 0.5695 | -335.39  (-354.28 – -325.43) | -349.71  (-372.95 – -332.83) | 0.5464 | -332.92  (-336.03 – -329.59) | -341.23  (-343.45 – -334.76) | 0.5237 |
|  | Temporal | -353.4  (-381.12 – -338.71) | -296.12  (-319.71 – -277.67) | 0.2837 | -351.43  (-356.14 – -349.26) | -295.11  (-299.88 – -289.56) | 0.29 | -361.46  (-382.58 – -341.84) | -308.66  (-319.98 – -295.37) | 0.3574 | -365.89  (-378.33 – -362.02) | -306.99  (-308.14 – -305.21) | 0.3502 |
|  | Occipital | -770.75  (-810.68 – -721.81) | -354.26  (-367.86 – -334.95) | **<0.001** | -780.88  (-812.26 – -739.51) | -329.46  (-339.39 – -324.33) | **<0.001** | -779.01  (-816.46 – -732.56) | -376.98  (-403.99 – -344.71) | **<0.001** | -794.41  (-826.8 – -753.63) | -363.24  (-365.85 – -360.28) | **<0.001** |
| HbDiff (au) | Frontal | -19.82  (-22.83 – -15.59) | -43.72  (-47.63 – -38) | **<0.001** | -17.42  (-18.51 – -14.93) | -43.01  (-46.02 – -40.55) | **<0.001** | -19.47  (-23.39 – -16.04) | -44.46  (-49.91 – -40.35) | **<0.001** | -15.6  (-18.84 – -14.37) | -42.54  (-44.07 – -39.95) | **<0.001** |
|  | Parietal | -4  (-18.21 – 13.64) | -24.62  (-34.9 – -15.04) | **0.0091** | -7.09  (-15.88 – 8.7) | -21.86  (-31.89 – -17.37) | 0.109 | -10.34  (-23.42 – 10.69) | -31.53  (-41.43 – -17.09) | **0.001** | -4.71  (-19.83 – 7.99) | -30.14  (-36.66 – -24.37) | **<0.001** |
|  | Temporal | -58.92  (-72.37 – -46.15) | -12.89  (-21.95 – -6.03) | **<0.001** | -61.09  (-68.77 – -50.48) | -11.9  (-18.57 – -5.05) | **<0.001** | -60.86  (-71.32 – -47.39) | -11.61  (-25.52 – -5.71) | **<0.001** | -56.01  (-68.71 – -53.65) | -13.02  (-19.82 – -10.26) | **<0.001** |
|  | Occipital | -226.87  (-303.88 – -125.29) | -16.41  (-29.03 – -8.19) | **<0.001** | -223.51  (-302.48 – -126.4) | -15.77  (-22.34 – -9.58) | **<0.001** | -230.94  (-309.12 – -129.01) | -20.99  (-32.27 – -12.13) | **<0.001** | -234.82  (-310.15 – -135.34) | -22.43  (-28.15 – -12.93) | **<0.001** |
| MAD of ABP (mmHg) | – | 12.77 (11.45 – 14.81) | | – | 12.6 (11.15 – 13.6) | | – | 12.85 (11.48 – 14.17) | | – | 12.15 (11.06 – 13.8) | | – |
| MAD of EtCO₂ (mmHg) | – | 1 (1 – 2) | | – | 1 (1 – 1) | | – | 1 (1 – 2) | | – | 2 (1 – 3) | | – |
| MAD of RR (bpm) | – | 2 (1 – 3) | | – | 2 (1 – 2) | | – | 2 (2 – 3) | | – | 5 (2 – 6) | | – |
| MAD of rSO_2_ (%) | Frontal | 0.47 (0.31 – 1.1) | 0.88 (0.4 – 1.53) | 0.1168 | 0.29 (0.18 – 0.49) | 0.38 (0.18 – 0.79) | 0.5695 | 0.55 (0.4 – 0.93) | 0.88 (0.62 – 1.37) | **0.0043** | 0.23 (0.14 – 0.38) | 0.32 (0.18 – 0.67) | 0.0932 |
|  | Parietal | 3.18 (1.02 – 5.06) | 1.38 (0.54 – 3.86) | **0.0056** | 3.57 (0.7 – 5.35) | 0.89 (0.35 – 3.9) | **0.006** | 2.6 (1.19 – 4.97) | 1.63 (1.01 – 4.07) | 0.0622 | 1.62 (0.42 – 4.76) | 0.48 (0.22 – 2.85) | **0.0037** |
|  | Temporal | 1.36 (0.53 – 4.97) | 1.47 (0.48 – 5.15) | 0.9368 | 0.79 (0.29 – 4.92) | 0.72 (0.25 – 5.02) | 0.7329 | 1.2 (0.69 – 5.1) | 0.97 (0.68 – 5.08) | 0.5884 | 0.54 (0.21 – 3.23) | 0.4 (0.2 – 5.04) | 0.855 |
|  | Occipital | 5.57 (4.45 – 6.26) | 1.1 (0.5 – 4.71) | **<0.001** | 5.04 (4.35 – 6.29) | 0.96 (0.4 – 4.29) | **<0.001** | 5.55 (4.29 – 6.23) | 1.45 (0.64 – 5.17) | **<0.001** | 5.39 (4.33 – 6.28) | 0.67 (0.23 – 4.78) | **<0.001** |
| MAD of HbO (au) | Frontal | 2.55 (1.63 – 4.55) | 4.08 (1.93 – 6.95) | 0.1985 | 1.31 (0.92 – 2.61) | 1.84 (0.8 – 3.39) | 0.4462 | 3.48 (2.62 – 7.19) | 4.07 (2.81 – 8.6) | 0.4545 | 0.94 (0.58 – 1.62) | 1.14 (0.71 – 2.63) | 0.1487 |
|  | Parietal | 15.44 (5.75 – 40.9) | 7.04 (3.96 – 25.14) | **0.0438** | 14.73 (2.83 – 36.09) | 4.11 (1.39 – 19.25) | **0.0204** | 15.01 (9.26 – 36.84) | 14.18 (6.92 – 30.9) | 0.627 | 5.34 (1.82 – 24.92) | 2.61 (1.14 – 9.99) | 0.1016 |
|  | Temporal | 8.72 (2.98 – 34.73) | 9.4 (2.89 – 35.15) | 0.8659 | 4.76 (1.22 – 37.6) | 3.77 (1.26 – 28.65) | 0.9698 | 9.72 (4.93 – 40.11) | 11.03 (3.73 – 31.36) | 0.6817 | 3.07 (1.02 – 11.78) | 2.43 (1.01 – 23.55) | 0.8227 |
|  | Occipital | 57.5 (43.39 – 59.96) | 8.07 (4.24 – 26.37) | **<0.001** | 57.67 (44.69 – 59.4) | 4.74 (1.9 – 20.92) | **<0.001** | 58.53 (44.32 – 60.05) | 13.35 (7.86 – 33.82) | **<0.001** | 57.93 (44.17 – 60.2) | 3.91 (1.04 – 20.11) | **<0.001** |
| MAD of HHb (au) | Frontal | 2.18 (0.86 – 4.72) | 3.72 (1.25 – 8.99) | 0.1217 | 0.81 (0.45 – 1.7) | 1.21 (0.43 – 2.63) | 0.5932 | 3.02 (1.35 – 5.64) | 3.51 (2.46 – 6.91) | 0.0747 | 0.63 (0.35 – 1.3) | 0.94 (0.34 – 2.53) | 0.4339 |
|  | Parietal | 14.34 (6.12 – 32.23) | 8.28 (2.98 – 26.54) | 0.1201 | 11.97 (2.67 – 31.31) | 4.76 (1.31 – 16.3) | **0.0416** | 14.72 (8.26 – 30.89) | 12.9 (5.92 – 24.72) | 0.5695 | 5.98 (2.07 – 18.33) | 2.92 (0.89 – 11.09) | 0.0725 |
|  | Temporal | 10.79 (3.27 – 34.39) | 8.85 (3.77 – 36.95) | 0.9588 | 5.65 (1.19 – 29.04) | 4.31 (1.14 – 33.41) | 0.7801 | 11.3 (4.69 – 38.31) | 9.03 (3.96 – 32.56) | 0.4421 | 2.84 (0.76 – 13.15) | 2.18 (0.74 – 30.32) | 0.8876 |
|  | Occipital | 40.82 (34.16 – 42.88) | 8.36 (3.05 – 24.37) | **<0.001** | 40.93 (35.35 – 42.72) | 4.47 (1.55 – 16.73) | **<0.001** | 41.08 (34.81 – 42.68) | 13.25 (7 – 29.7) | **<0.001** | 41.07 (33.69 – 42.74) | 3.5 (1.03 – 17.55) | **<0.001** |
| MAD of tHb (au) | Frontal | 3.26 (1.71 – 7.67) | 6.09 (2.6 – 12.33) | 0.0893 | 1.73 (1.05 – 3.32) | 1.85 (0.81 – 4.21) | 0.9259 | 6.12 (3.58 – 13.01) | 6.37 (3.67 – 14.33) | 0.6716 | 0.99 (0.63 – 1.84) | 1.09 (0.62 – 4.46) | 0.759 |
|  | Parietal | 20.15 (7.64 – 43.02) | 11.72 (2.64 – 24.71) | **0.037** | 9.64 (2.35 – 23.67) | 5.62 (0.99 – 13.94) | **0.0475** | 18.49 (10.12 – 32.19) | 19.74 (11.64 – 34.89) | 0.796 | 4.27 (1.68 – 15.91) | 2.7 (1.24 – 11.33) | 0.2568 |
|  | Temporal | 16.17 (5.41 – 34) | 14.9 (5.73 – 30.75) | 0.9094 | 6.19 (1.43 – 26.95) | 4.69 (1.73 – 20.45) | 0.6616 | 17.4 (9.2 – 33.42) | 16.71 (6.79 – 33.32) | 0.5602 | 3.62 (1.09 – 11.05) | 3.02 (1.25 – 12.67) | 0.7123 |
|  | Occipital | 34.62 (26.82 – 38.46) | 14.2 (4.8 – 32.23) | **<0.001** | 34.43 (27.55 – 36.54) | 4.59 (2.34 – 16.65) | **<0.001** | 34.78 (28.22 – 38.2) | 22.69 (11.35 – 38.76) | **0.013** | 34.73 (27.41 – 36.75) | 5.14 (1.1 – 16.32) | **<0.001** |
| MAD of HbDiff (au) | Frontal | 2.45 (1.27 – 4.33) | 3.91 (1.76 – 5.78) | 0.1566 | 1.39 (0.83 – 2.94) | 1.76 (0.84 – 3.9) | 0.3869 | 2.58 (1.77 – 4.76) | 4.16 (2.59 – 5.57) | **0.0271** | 1.02 (0.58 – 1.78) | 1.33 (0.86 – 2.74) | 0.0793 |
|  | Parietal | 21.49 (5.44 – 42.93) | 9.07 (3.52 – 35.81) | **0.0498** | 22.52 (3.94 – 62.4) | 5.46 (2.19 – 32.96) | **0.0302** | 17.36 (6.37 – 48.57) | 11.02 (5.41 – 36.7) | 0.2745 | 9.15 (3.12 – 38.19) | 3 (1.36 – 20.13) | **0.0204** |
|  | Temporal | 6.5 (2.43 – 56.67) | 6.46 (2.25 – 60.63) | 0.8335 | 4.39 (2.07 – 62.44) | 3.99 (1.3 – 62.93) | 0.4299 | 7.86 (3.52 – 45.12) | 5.51 (3.36 – 56.85) | 0.7329 | 3.67 (1.24 – 25) | 2.66 (1.11 – 45.98) | 0.8876 |
|  | Occipital | 88.54 (70.86 – 93.95) | 8.09 (2.81 – 36.76) | **<0.001** | 85.54 (72.31 – 94.7) | 6.1 (2 – 33.48) | **<0.001** | 89.1 (72.01 – 94.31) | 10.14 (4.21 – 34.68) | **<0.001** | 87.77 (72.48 – 94.18) | 4.46 (1.53 – 34.94) | **<0.001** |
| The p-values in the table are derived using Mann-Whitney U test between the bilateral signals. *ABP, arterial blood pressure; bpm, beats per minute; EtCO_2_, end-tidal carbon dioxide; HbDiff, hemoglobin difference; HbO, oxyhemoglobin; HHb, deoxyhemoglobin; IQR, interquartile range; MAD, median absolute deviation; mmHg, millimeters of mercury; RR, respiratory rate; rSO_2_, regional cerebral oxygen saturation; tHb, total hemoglobin.* | | | | | | | | | | | | | |

Appendix S7d: Perturbation Subgrouped Percent Time Results of rSO_2_ and CVR Indices Using 10-Second Decimated Data

| **Physiologic Variable** | **Brain Lobe** | **Baseline** | | | **Neurovascular Coupling** | | | **Orthostatic Challenge** | | | **Vascular Chemo-Reactivity** | | |
| --- | --- | --- | --- | --- | --- | --- | --- | --- | --- | --- | --- | --- | --- |
|  |  | **Median (IQR; MAD)** | | **p-value** | **Median (IQR; MAD)** | | **p-value** | **Median (IQR; MAD)** | | **p-value** | **Median (IQR; MAD)** | | **p-value** |
|  |  | **Left Hemisphere** | **Right Hemisphere** |  | **Left Hemisphere** | **Right Hemisphere** |  | **Left Hemisphere** | **Right Hemisphere** |  | **Left Hemisphere** | **Right Hemisphere** |  |
| **1 Hz Sampled Data** | | | | | | | | | | | | | |
| % time rSO_2_ > 30% | Frontal | 100 (100 – 100; 0) | 100 (100 – 100; 0) | **0.0123** | 100 (100 – 100; 0) | 100 (100 – 100; 0) | 1 | 100 (100 – 100; 0) | 100 (100 – 100; 0) | **0.0433** | 100 (100 – 100; 0) | 100 (100 – 100; 0) | 0.1594 |
|  | Parietal | 100 (100 – 100; 0) | 100 (100 – 100; 0) | 0.3271 | 100 (100 – 100; 0) | 100 (100 – 100; 0) | 0.5676 | 100 (100 – 100; 0) | 100 (100 – 100; 0) | 0.1739 | 100 (100 – 100; 0) | 100 (100 – 100; 0) | 1 |
|  | Temporal | 100 (100 – 100; 0) | 100 (100 – 100; 0) | 0.3271 | 100 (100 – 100; 0) | 100 (100 – 100; 0) | 1 | 100 (100 – 100; 0) | 100 (100 – 100; 0) | 0.5836 | 100 (100 – 100; 0) | 100 (100 – 100; 0) | 0.3271 |
|  | Occipital | 100 (100 – 100; 0) | 100 (100 – 100; 0) | 0.3271 | 100 (100 – 100; 0) | 100 (100 – 100; 0) | 1 | 100 (100 – 100; 0) | 100 (100 – 100; 0) | 0.3271 | 100 (100 – 100; 0) | 100 (100 – 100; 0) | 1 |
| % time rSO_2_ > 40% | Frontal | 100 (100 – 100; 0) | 57.04 (1.75 – 100; 42.96) | **<0.001** | 100 (100 – 100; 0) | 72.12 (0 – 100; 27.88) | **<0.001** | 100 (100 – 100; 0) | 64.03 (0.08 – 100; 35.97) | **<0.001** | 100 (100 – 100; 0) | 97.34 (0 – 100; 2.66) | **<0.001** |
|  | Parietal | 100 (70.66 – 100; 0) | 100 (83.59 – 100; 0) | 0.6949 | 100 (56.69 – 100; 0) | 100 (100 – 100; 0) | 0.4408 | 100 (88.82 – 100; 0) | 100 (88.12 – 100; 0) | 0.6021 | 100 (100 – 100; 0) | 100 (100 – 100; 0) | 0.5246 |
|  | Temporal | 58.5 (0 – 100; 41.5) | 100 (100 – 100; 0) | **<0.001** | 44.25 (0 – 100; 44.25) | 100 (100 – 100; 0) | **<0.001** | 59.87 (4.3 – 99.23; 40.13) | 100 (100 – 100; 0) | **<0.001** | 77.61 (0 – 100; 22.39) | 100 (100 – 100; 0) | **0.0015** |
|  | Occipital | 0 (0 – 0; 0) | 100 (100 – 100; 0) | **<0.001** | 0 (0 – 0; 0) | 100 (100 – 100; 0) | **<0.001** | 0 (0 – 0; 0) | 100 (92.8 – 100; 0) | **<0.001** | 0 (0 – 0; 0) | 100 (100 – 100; 0) | **<0.001** |
| % time rSO_2_ > 50% | Frontal | 0 (0 – 64.98; 0) | 0 (0 – 0; 0) | **0.0051** | 0 (0 – 97.62; 0) | 0 (0 – 0; 0) | **<0.001** | 0 (0 – 66.51; 0) | 0 (0 – 0; 0) | **<0.001** | 0 (0 – 100; 0) | 0 (0 – 0; 0) | **<0.001** |
|  | Parietal | 36.69 (0 – 97.72; 36.69) | 0 (0 – 36.24; 0) | **<0.001** | 25.45 (0 – 100; 25.45) | 0 (0 – 36.17; 0) | **0.0201** | 35.38 (0 – 83.7; 35.38) | 0 (0 – 6.66; 0) | **<0.001** | 5.54 (0 – 100; 5.54) | 0 (0 – 0; 0) | **<0.001** |
|  | Temporal | 0 (0 – 0; 0) | 0 (0 – 40.73; 0) | **<0.001** | 0 (0 – 0; 0) | 0 (0 – 44.57; 0) | **<0.001** | 0 (0 – 0; 0) | 2.12 (0 – 47.83; 2.12) | **<0.001** | 0 (0 – 0; 0) | 0 (0 – 29.27; 0) | **<0.001** |
|  | Occipital | 0 (0 – 0; 0) | 0 (0 – 23.92; 0) | **<0.001** | 0 (0 – 0; 0) | 0 (0 – 27.9; 0) | **<0.001** | 0 (0 – 0; 0) | 0 (0 – 6.36; 0) | **<0.001** | 0 (0 – 0; 0) | 0 (0 – 0; 0) | **<0.001** |
| % time rSO_2_ > 60% | Frontal | 0 (0 – 0; 0) | 0 (0 – 0; 0) | 1 | 0 (0 – 0; 0) | 0 (0 – 0; 0) | 0.3271 | 0 (0 – 0; 0) | 0 (0 – 0; 0) | 0.3271 | 0 (0 – 0; 0) | 0 (0 – 0; 0) | 1 |
|  | Parietal | 0 (0 – 0; 0) | 0 (0 – 0; 0) | 0.6416 | 0 (0 – 0; 0) | 0 (0 – 0; 0) | 0.3004 | 0 (0 – 0; 0) | 0 (0 – 0; 0) | 0.1797 | 0 (0 – 0; 0) | 0 (0 – 0; 0) | 1 |
|  | Temporal | 0 (0 – 0; 0) | 0 (0 – 0; 0) | **0.0433** | 0 (0 – 0; 0) | 0 (0 – 0; 0) | 0.1594 | 0 (0 – 0; 0) | 0 (0 – 0; 0) | 0.5519 | 0 (0 – 0; 0) | 0 (0 – 0; 0) | 0.3271 |
|  | Occipital | 0 (0 – 0; 0) | 0 (0 – 0; 0) | 0.3271 | 0 (0 – 0; 0) | 0 (0 – 0; 0) | 0.1594 | 0 (0 – 0; 0) | 0 (0 – 0; 0) | **0.0822** | 0 (0 – 0; 0) | 0 (0 – 0; 0) | 1 |
| % time COx-a > 0 | Frontal | 55.84  (33.33 – 67.56; 17.92) | 57.28  (45.11 – 75.23; 17.32) | 0.8013 | 46.02  (38.95 – 60.76; 10.87) | 44.4  (30.49 – 55.44; 13.76) | 0.3832 | 49.4  (36.68 – 58.91; 11.04) | 49.91  (42.71 – 59.09; 8.56) | 0.2317 | 50.42  (30.29 – 60.63; 16.06) | 53.21  (37.4 – 70.83; 16.93) | 0.1383 |
|  | Parietal | 49.23  (30.77 – 69.7; 18.78) | 43.3  (32.2 – 61.32; 12.39) | 0.6516 | 47.96  (34.98 – 62.68; 14.58) | 45.5  (34.54 – 57.23; 11.59) | 0.3813 | 45.93  (40.38 – 57.45; 9.59) | 48.48  (41.93 – 58.58; 7.86) | 0.7329 | 57.13  (39.53 – 67.82; 12.68) | 46.46  (29.85 – 64.8; 17.97) | 0.1234 |
|  | Temporal | 58.85  (36.09 – 74.88; 22.12) | 48.91  (32.19 – 65.06; 16.54) | 0.1059 | 49.59  (36.29 – 61.66; 13.07) | 52.24  (38.34 – 70.1; 16.59) | 0.1733 | 48.51  (41.96 – 60.51; 10.71) | 50.58  (41.54 – 58.61; 9.03) | 0.9808 | 54.5  (35.87 – 70.16; 17.14) | 50.42  (34.54 – 63.32; 15.25) | 0.3225 |
|  | Occipital | 57.69  (37.47 – 73.21; 16.65) | 57.64  (38.13 – 74.43; 17.5) | 0.9533 | 51.52  (36.41 – 65.05; 14.53) | 51.83  (42.75 – 65.67; 11.78) | 0.7433 | 49.51  (36.72 – 55.76; 10.15) | 50.65  (42.97 – 62.31; 11.04) | 0.1365 | 46.62  (23.54 – 74.48; 25.78) | 46.55  (30.37 – 63.35; 16.88) | 0.6124 |
| % time COx-a > 0.2 | Frontal | 19.72  (5.97 – 46.41; 19.72) | 18.71  (7.1 – 40.88; 14.36) | 0.8627 | 18.47  (10.76 – 31.97; 10.05) | 21.98  (8.27 – 34; 12.63) | 0.9588 | 22.59  (13.81 – 31.9; 9.07) | 27.46  (22.09 – 36.29; 6.72) | **0.0329** | 14.7  (0 – 31.05; 14.7) | 16.54  (2.73 – 38.86; 15.43) | 0.3509 |
|  | Parietal | 20.45  (5.24 – 39; 15.55) | 23.3  (8.08 – 46.72; 16.41) | 0.6187 | 18.82  (11.96 – 31.91; 9.43) | 21.95  (16.44 – 31.56; 8.12) | 0.467 | 23.24  (15.11 – 31.82; 8.49) | 28.23  (16.11 – 33.59; 10.52) | 0.4586 | 17.47  (0.3 – 35.35; 17.47) | 15.42  (5.19 – 35.03; 13.48) | 0.8137 |
|  | Temporal | 23.11  (4.89 – 42.17; 18.46) | 15.54  (3.57 – 31.14; 12.86) | 0.1462 | 23.22  (9.25 – 33.4; 12.72) | 24.7  (12.22 – 37.7; 12.84) | 0.3397 | 24.47  (15.04 – 31.83; 9.36) | 22.82  (14.49 – 32.46; 9.19) | 0.9588 | 15.38  (3.85 – 33.81; 15.38) | 11.89  (0 – 27.44; 11.89) | 0.2298 |
|  | Occipital | 15.69  (3 – 36.78; 15.22) | 25  (6.13 – 38.63; 15.72) | 0.3257 | 16.1  (5.94 – 23.13; 8.25) | 25.88  (12.86 – 35.63; 10.98) | **0.0015** | 19.17  (11.33 – 25.61; 7.37) | 27.24  (18.61 – 40.56; 11.94) | **<0.001** | 7.76  (1.21 – 19.83; 7.76) | 13.44  (2.35 – 20.77; 9.59) | 0.3392 |
| % time COx-a > 0.3 | Frontal | 14  (0 – 29.13; 14) | 7.92  (0 – 30.01; 7.92) | 0.575 | 10.03  (3.46 – 20.25; 8.38) | 13.27  (3.76 – 21.78; 9.42) | 0.6614 | 12.62  (8.55 – 19.09; 5.32) | 17.4  (12.64 – 24.44; 5.56) | **0.037** | 3.2  (0 – 15.61; 3.2) | 6.92  (0 – 22.76; 6.92) | 0.4067 |
|  | Parietal | 10.1  (0.49 – 27.17; 10.1) | 13.96  (0 – 30.2; 13.96) | 0.7617 | 10.94  (5.12 – 16.24; 5.75) | 13.69  (5.02 – 19.87; 7.98) | 0.3534 | 15.54  (9.43 – 20.98; 5.9) | 17.15  (7.7 – 26.09; 9.29) | 0.6893 | 5.17  (0 – 19.93; 5.17) | 4.85  (0 – 17.2; 4.85) | 0.7373 |
|  | Temporal | 10.73  (0 – 27.51; 10.73) | 6.82  (0 – 17.04; 6.82) | 0.3061 | 9.27  (3.14 – 17.5; 7.55) | 13.28  (4.95 – 23.59; 8.56) | 0.1804 | 13.73  (6.81 – 22.65; 8.73) | 13.12  (6.56 – 23.43; 8.58) | 1 | 2.69  (0 – 18.06; 2.69) | 0.96  (0 – 9.85; 0.96) | 0.2701 |
|  | Occipital | 0  (0 – 12.29; 0) | 11.68  (0 – 26.65; 11.68) | **0.0283** | 4.28  (0 – 11.5; 4.28) | 13.1  (4.28 – 24.48; 10.91) | **<0.001** | 8.15  (4.19 – 15.04; 5.68) | 18  (9.25 – 27.81; 9.66) | **<0.001** | 0  (0 – 4.14; 0) | 1.98  (0 – 10.12; 1.98) | **0.0247** |
| % time HbOx > 0 | Frontal | 53.59  (32.5 – 61.94; 12.57) | 51.72  (28.14 – 67.34; 18.19) | 0.6269 | 47.37  (40.39 – 62.87; 10.18) | 47.79  (39.06 – 62.86; 12.81) | 0.9122 | 45.42  (33.53 – 59.37; 12.02) | 52.4  (42.91 – 62.65; 10.21) | **0.037** | 50.56  (32.12 – 67.27; 17.49) | 53.28  (32.49 – 67.27; 17.99) | 0.7511 |
|  | Parietal | 50.62  (36.14 – 70.51; 17.45) | 54.52  (33.62 – 67.1; 18.16) | 0.9122 | 51.59  (38.2 – 63.56; 13.36) | 50.6  (37.29 – 61.49; 13.29) | 0.8254 | 47.49  (41.17 – 57.03; 7.88) | 54.06  (43.62 – 61.44; 8.56) | 0.3592 | 47.84  (34.69 – 69.17; 16.77) | 44.94  (32.83 – 65.56; 16.44) | 0.3794 |
|  | Temporal | 50  (34.91 – 70.44; 19.34) | 45.36  (32.11 – 63.21; 15.94) | 0.301 | 50.38  (34.54 – 59.9; 12.58) | 47.47  (38.61 – 64.12; 11.73) | 0.3467 | 48.66  (43.88 – 58.26; 7.63) | 52.15  (41.84 – 65.11; 11.88) | 0.6075 | 54.14  (35.63 – 75.6; 20.86) | 43.13  (29.41 – 62.29; 14.28) | 0.0994 |
|  | Occipital | 54.25  (38.42 – 72.86; 17.09) | 49.51  (37.28 – 60; 12.16) | 0.1302 | 55.33  (38.83 – 63.53; 12.78) | 49.78  (38 – 61.52; 12.01) | 0.72 | 49.92  (43.53 – 60.47; 7.69) | 54.63  (41.98 – 64.13; 12.45) | 0.5464 | 42.99  (29.47 – 67.06; 19.18) | 44.38  (28.57 – 64.98; 19.5) | 0.8931 |
| % time HbOx > 0.2 | Frontal | 18.41  (8.28 – 35.96; 15.19) | 18.71  (6.25 – 34.87; 15.05) | 0.9641 | 19.88  (14 – 31.68; 6.93) | 25.47  (15.41 – 38.46; 12.08) | 0.3984 | 23.39  (12.45 – 31.5; 10.29) | 29.3  (21.25 – 36.3; 7.43) | **0.0094** | 13.09  (3.74 – 27.12; 11.94) | 16.08  (5.39 – 31.83; 12.5) | 0.2487 |
|  | Parietal | 19.09  (5 – 42.81; 16.08) | 20.54  (8.46 – 43.71; 14.5) | 0.6763 | 22.95  (15.64 – 37.04; 9.87) | 23.32  (15.81 – 36.63; 11.45) | 0.7748 | 27.66  (18.14 – 35.16; 7.94) | 31.25  (18.58 – 38.33; 9.67) | 0.3984 | 18.57  (2.16 – 37.2; 16.63) | 17.86  (5.45 – 30.87; 12.6) | 0.7903 |
|  | Temporal | 16.67  (5.19 – 38.05; 12.83) | 16.32  (3.76 – 28.19; 12.51) | 0.5273 | 22.85  (11.15 – 29.98; 9.83) | 24.74  (15.44 – 35.18; 9.44) | 0.2553 | 26.01  (15.75 – 32.14; 9.42) | 28.2  (19.24 – 38.06; 9.48) | 0.2806 | 23.15  (0 – 34.39; 18.11) | 9.35  (2.51 – 24.16; 9.35) | 0.2578 |
|  | Occipital | 20  (5.14 – 41.91; 19.83) | 21.9  (6.92 – 36.3; 14.78) | 0.6483 | 20.64  (8.21 – 25.91; 7.84) | 21.37  (14.73 – 35.01; 9.96) | 0.1636 | 20.98  (15.41 – 32.97; 9.39) | 31.6  (18.6 – 39.99; 10.95) | 0.0566 | 6.81  (0 – 20.63; 6.81) | 10.01  (2.13 – 38.79; 10) | 0.1189 |
| % time HbOx > 0.3 | Frontal | 10.07  (0 – 19.31; 10.07) | 9.17  (0 – 25.99; 9.17) | 0.8463 | 11.76  (8.09 – 21.94; 5.62) | 14.74  (6.5 – 28.94; 10.11) | 0.4627 | 13.48  (5.15 – 22.32; 8.81) | 17.87  (12.83 – 25.22; 6.02) | **0.0111** | 0.6  (0 – 14.01; 0.6) | 3.43  (0 – 19.65; 3.43) | 0.1304 |
|  | Parietal | 10.43  (0 – 22.86; 10.43) | 9.48  (0.45 – 28.67; 9.48) | 0.9029 | 14.43  (4.97 – 20.99; 9.02) | 15.79  (6.38 – 25.94; 9.95) | 0.4667 | 19.74  (11.34 – 24.6; 5.84) | 20.44  (11.52 – 28.39; 8.65) | 0.5742 | 6.15  (0 – 16.9; 6.15) | 5.31  (0 – 17.05; 5.31) | 0.8255 |
|  | Temporal | 4.95  (0 – 23.49; 4.95) | 6.39  (0 – 18.11; 6.39) | 0.6835 | 10.67  (2.01 – 21.05; 9.41) | 13.73  (6.65 – 19.76; 6.75) | 0.364 | 15.93  (8.6 – 22.68; 7.27) | 17.4  (9.62 – 25.83; 8.24) | 0.4649 | 8.11  (0 – 21.53; 8.11) | 1.92  (0 – 9.93; 1.92) | 0.1192 |
|  | Occipital | 5.56  (0 – 18.97; 5.56) | 4.29  (0 – 24; 4.29) | 0.7816 | 7.64  (1.63 – 13.94; 6.34) | 11.88  (5.91 – 21.08; 7.9) | **0.0119** | 11.84  (8.02 – 22.17; 6.75) | 20.55  (11.05 – 29.14; 8.73) | **0.0399** | 0  (0 – 4.91; 0) | 1.94  (0 – 19.59; 1.94) | **0.0149** |
| % time HHbx > 0 | Frontal | 50.68  (30.77 – 67.67; 19.91) | 44.73  (22.26 – 61.31; 19.98) | 0.4061 | 53.8  (42.01 – 61.01; 11.31) | 56.75  (42.52 – 65.99; 13.24) | 0.3467 | 54.88  (38.56 – 62.49; 11.34) | 51.3  (39.73 – 59.05; 9.18) | 0.7512 | 47.32  (36.12 – 64.74; 13.77) | 52.22  (28.34 – 64.06; 17.35) | 0.8147 |
|  | Parietal | 44.04  (24.69 – 65.52; 20.99) | 50.76  (26.59 – 68.83; 21.11) | 0.3945 | 49.41  (39.92 – 64.53; 12.24) | 51.12  (39.62 – 59.72; 9.98) | 0.9341 | 51.84  (42.57 – 59.06; 8.35) | 54.87  (45.72 – 61.42; 7.2) | 0.3851 | 45.33  (30.28 – 61.16; 15.61) | 44.44  (28.45 – 67.71; 21.01) | 0.8985 |
|  | Temporal | 49.14  (31.86 – 69.56; 18.95) | 56.62  (37.08 – 68.19; 15.2) | 0.5081 | 53.74  (35.16 – 59.94; 10.55) | 47.06  (37.69 – 57.89; 10.05) | 0.2979 | 45.64  (39.98 – 57.91; 8.92) | 54.01  (42.29 – 63.34; 10.35) | 0.1392 | 51  (35.99 – 66.9; 15.81) | 48.22  (32.24 – 62.68; 15.55) | 0.7097 |
|  | Occipital | 44.5  (33.93 – 56.48; 11.94) | 49.27  (27.93 – 64.15; 17.96) | 0.7616 | 49.55  (38.22 – 61.03; 11.57) | 49.19  (36.24 – 59.6; 11.7) | 0.6172 | 55.31  (46.03 – 66.09; 9.94) | 50.26  (40.57 – 61.24; 10.46) | 0.1638 | 53.53  (29.9 – 72.01; 21.13) | 53.15  (32.8 – 72.54; 19.49) | 0.8281 |
| % time HHbx > 0.2 | Frontal | 26.12  (7.02 – 43.43; 18.35) | 17.32  (4.84 – 32.76; 14.05) | 0.2482 | 25  (14.7 – 33.39; 9.11) | 24.91  (17.05 – 38.38; 10.39) | 0.5015 | 28.7  (16.35 – 35.84; 9) | 29.28  (22.56 – 36.12; 7.29) | 0.4061 | 14.3  (3.96 – 28.51; 12.62) | 15.18  (3.61 – 32.55; 13.52) | 0.9228 |
|  | Parietal | 19.71  (4.69 – 33.11; 13.81) | 22.54  (10.32 – 37.93; 15.18) | 0.585 | 25.02  (13.98 – 34.22; 10.05) | 24.35  (13.48 – 35.39; 11.09) | 0.7801 | 24.87  (19.45 – 35.41; 8.98) | 30.27  (21.65 – 41.28; 8.94) | 0.2134 | 14.64  (5.36 – 30.69; 12.17) | 16.11  (2.05 – 31.86; 14.97) | 0.7928 |
|  | Temporal | 15.83  (4.76 – 43.58; 15.83) | 22.91  (10.22 – 39.96; 14.9) | 0.4247 | 21.23  (14.91 – 31.89; 9.63) | 20.71  (9.5 – 31.48; 11.38) | 0.517 | 25.66  (16.64 – 31.69; 8.99) | 28.11  (19.6 – 38.31; 9.36) | 0.2776 | 12.27  (3.62 – 29.74; 12.27) | 13.38  (2.45 – 24.53; 11.12) | 0.5705 |
|  | Occipital | 15.5  (2.65 – 26.74; 12.76) | 19.72  (3.23 – 29.54; 13.78) | 0.4589 | 17.42  (5.89 – 30.52; 11.93) | 23.13  (14.7 – 30.71; 8.22) | 0.0946 | 20.58  (14.02 – 32.04; 9.19) | 28.5  (19.9 – 37.01; 8.62) | 0.0589 | 9.44  (0 – 21.47; 9.44) | 20.7  (5.51 – 35.85; 15.24) | **0.0191** |
| % time HHbx > 0.3 | Frontal | 11.77  (0 – 25.51; 11.77) | 7.49  (0 – 19.81; 7.49) | 0.5396 | 13.9  (6.85 – 24.84; 8.33) | 16.4  (7.58 – 27.86; 9.9) | 0.5147 | 16.17  (10.97 – 25.04; 7.27) | 18.82  (13.26 – 25.94; 5.77) | 0.27 | 8.5  (0 – 19.91; 8.5) | 4.9  (0 – 16.27; 4.9) | 0.8169 |
|  | Parietal | 10.31  (0 – 21.96; 10.31) | 11.6  (0 – 26.25; 11.6) | 0.3785 | 12.4  (5.45 – 21.13; 8.26) | 12.44  (4.67 – 23.07; 8.64) | 0.674 | 18.25  (11.54 – 24.18; 6.17) | 18.45  (12.24 – 28.58; 6.72) | 0.5081 | 4.11  (0 – 15.39; 4.11) | 4.38  (0 – 20.03; 4.38) | 0.983 |
|  | Temporal | 5.9  (0 – 20.1; 5.9) | 8.54  (0 – 23.66; 8.54) | 0.6463 | 12.06  (3.31 – 23.41; 9.54) | 12.62  (0.97 – 21.88; 11.01) | 0.6559 | 15.59  (9.7 – 22.6; 6.64) | 18.18  (12.24 – 28.29; 8.13) | 0.1938 | 2.02  (0 – 12.38; 2.02) | 0.66  (0 – 13.42; 0.66) | 0.7185 |
|  | Occipital | 3.58  (0 – 12.96; 3.58) | 6.51  (0 – 16.33; 6.51) | 0.2175 | 5.67  (0.78 – 13.75; 5.67) | 14.4  (4.41 – 22.03; 8.17) | **0.0025** | 11.39  (5.72 – 17.34; 5.91) | 16.7  (11.07 – 27.67; 8.17) | **0.0119** | 0  (0 – 6.8; 0) | 6.49  (0 – 21.19; 6.49) | **0.0039** |
| % time tHbx > 0 | Frontal | 52.61  (32.64 – 63.43; 15.1) | 50  (25.25 – 71.79; 23.57) | 0.6994 | 52.07  (37.51 – 61.03; 11.37) | 49.63  (38.93 – 61.47; 10.97) | 0.8605 | 49.68  (35.03 – 60.69; 12.71) | 52.96  (44.22 – 60.74; 8.4) | 0.372 | 45.78  (36.01 – 61.1; 13.26) | 50.66  (29.58 – 65.56; 18.67) | 0.8577 |
|  | Parietal | 46.16  (29.87 – 67.39; 20.92) | 51.95  (33.7 – 64.93; 15.73) | 0.8794 | 51.12  (36.1 – 66.67; 15.55) | 46.93  (36 – 64.91; 16.03) | 0.8958 | 51.61  (38.35 – 59.97; 10.64) | 54.42  (45.31 – 62.16; 8.44) | 0.2963 | 44.67  (31.23 – 69.71; 18.85) | 43.74  (34.02 – 62.03; 15.05) | 0.9725 |
|  | Temporal | 50  (40.1 – 72.86; 16.09) | 49.27  (35.84 – 66.67; 14.64) | 0.3537 | 47.56  (41.91 – 59.5; 8.83) | 46.08  (37.62 – 58.24; 8.9) | 0.5487 | 50  (40.72 – 61.88; 10.38) | 52.31  (45.43 – 65.38; 10.95) | 0.29 | 56.09  (32.18 – 70.21; 22.73) | 46.97  (37.46 – 63.5; 14.83) | 0.3869 |
|  | Occipital | 58.57  (38.45 – 70.96; 16.39) | 50  (36.29 – 61.92; 12.68) | 0.12 | 52.85  (37.72 – 59.43; 10.04) | 50.42  (35.18 – 61.05; 12.86) | 0.7538 | 50.95  (45.59 – 61.63; 8.24) | 51.72  (43.48 – 64.47; 9.96) | 0.9588 | 43.28  (30.07 – 67.01; 19.65) | 48.33  (34.37 – 64.31; 15.87) | 0.5059 |
| % time tHbx > 0.2 | Frontal | 23.33  (7.06 – 37.78; 16.01) | 26.2  (8.17 – 34.95; 13.02) | 0.8166 | 25.49  (17.12 – 37.92; 10.35) | 24.9  (15.74 – 36; 10.68) | 0.7695 | 23.28  (13.48 – 35.08; 10.74) | 28.33  (23.29 – 35.9; 7) | 0.0531 | 11.58  (3.58 – 30.55; 11.58) | 14.41  (1.93 – 28.57; 13.82) | 0.9917 |
|  | Parietal | 16.25  (8.38 – 35.12; 11.25) | 16.67  (8.63 – 34.78; 13.53) | 0.9176 | 26.33  (14.82 – 35.97; 11.12) | 24.73  (10.66 – 41.39; 14.88) | 0.8876 | 27.55  (17.53 – 36.16; 9.46) | 30.06  (22.44 – 38.79; 8.16) | 0.2761 | 18.6  (6.06 – 33.01; 12.99) | 13.21  (4.51 – 25.24; 10.31) | 0.1981 |
|  | Temporal | 20.07  (11.46 – 33.39; 10.35) | 21.54  (3.68 – 33.33; 14.68) | 0.8547 | 22.45  (12.07 – 32.89; 10.59) | 23.85  (15.83 – 30.68; 7.49) | 0.874 | 26.28  (20.71 – 35.3; 6.76) | 31.08  (21.44 – 39.04; 9.3) | 0.2995 | 20.86  (1.95 – 36.29; 18.78) | 10.68  (3.49 – 25.4; 8.87) | 0.3161 |
|  | Occipital | 23.9  (9.92 – 39.38; 15.35) | 23.11  (9.48 – 37.03; 13.98) | 0.8146 | 22.41  (12.66 – 29.42; 8.66) | 21.37  (12.39 – 35.81; 11.72) | 0.3832 | 25.33  (16.64 – 33.32; 8.33) | 30.21  (18.35 – 40.55; 10.86) | 0.2083 | 5.98  (0 – 23.69; 5.98) | 17.61  (7.34 – 39.88; 15.31) | **0.0028** |
| % time tHbx > 0.3 | Frontal | 9.04  (0 – 25.14; 9.04) | 12.92  (0.54 – 25.3; 12.7) | 0.5553 | 14.38  (6.02 – 27.93; 9.7) | 15.51  (8.73 – 24.69; 8.23) | 0.8281 | 15.16  (8.04 – 23.56; 7.45) | 18.84  (15.6 – 25.98; 6.44) | **0.0119** | 0.55  (0 – 14.43; 0.55) | 3.32  (0 – 15.34; 3.32) | 0.6205 |
|  | Parietal | 10.25  (0 – 22.22; 10.25) | 10.56  (0 – 19.94; 10.56) | 0.9112 | 16.41  (6.18 – 25.49; 9.45) | 15.2  (2.76 – 27.86; 12.75) | 0.8684 | 17.99  (10.33 – 25; 7.71) | 17.95  (12.9 – 30.94; 8.15) | 0.586 | 7.45  (0.29 – 15.66; 7.45) | 4.14  (0 – 16.13; 4.14) | 0.2621 |
|  | Temporal | 5.8  (0 – 18.77; 5.8) | 9.07  (0 – 21.2; 9.07) | 0.8247 | 12.72  (4.04 – 22.92; 9.49) | 12.01  (7.07 – 18.62; 6.27) | 0.8767 | 18.49  (10.19 – 28.03; 8.73) | 21.62  (12.18 – 27.28; 9.07) | 0.3869 | 6.79  (0 – 21.53; 6.79) | 0  (0 – 14.02; 0) | 0.0937 |
|  | Occipital | 11.11  (0 – 29.33; 11.11) | 9.58  (1.84 – 24.7; 9.58) | 0.9473 | 11.99  (3.96 – 19.48; 7.95) | 14.01  (4.93 – 22.29; 9.04) | 0.3223 | 15.24  (7.35 – 23.49; 8.12) | 19.54  (11.48 – 30.46; 8.99) | 0.1617 | 0  (0 – 7.03; 0) | 6.31  (0 – 23.33; 6.31) | **0.0073** |
| % time HbDiffx > 0 | Frontal | 55.84  (33.82 – 69.74; 16.77) | 57.65  (40.51 – 73.06; 17.1) | 0.6318 | 46.52  (38.72 – 62.4; 11.05) | 48.35  (34.42 – 58.19; 10.83) | 0.7616 | 49.92  (37.5 – 56.85; 9.84) | 52.36  (44.8 – 60.56; 8.03) | 0.0642 | 51.28  (30.96 – 62.92; 18.08) | 51.89  (38.42 – 70.42; 13.99) | 0.3092 |
|  | Parietal | 47.69  (31.26 – 67.67; 18.06) | 42.12  (31.57 – 64.83; 13.35) | 0.7538 | 46.61  (34.17 – 63.74; 14.96) | 46.06  (35.9 – 56.8; 10.65) | 0.7046 | 48.39  (40.47 – 59.18; 8.87) | 49.84  (41.67 – 56.84; 8.15) | 0.9478 | 53.45  (41.63 – 67.82; 13.43) | 49.2  (33.12 – 66.9; 18) | 0.2317 |
|  | Temporal | 61.82  (38.89 – 78.37; 22.05) | 50  (27.53 – 63; 18.99) | **0.0179** | 47.58  (35.28 – 57.32; 11.52) | 52.19  (39.03 – 69.27; 15.85) | 0.1121 | 48.74  (41.03 – 61.59; 10.01) | 51.88  (42.44 – 58.23; 8.69) | 0.9862 | 56.9  (39.75 – 79.92; 19) | 48.55  (36.04 – 60.21; 12.46) | 0.0549 |
|  | Occipital | 54.25  (39.41 – 72.51; 17.4) | 57.07  (35.25 – 72.4; 19.23) | 0.8013 | 54.3  (34.06 – 64.01; 12.11) | 51.64  (41.92 – 63.59; 11.82) | 0.8227 | 49.9  (42.33 – 57.28; 7.58) | 51.52  (42.08 – 66.22; 11.95) | 0.3907 | 49.94  (27.72 – 70.94; 22.15) | 46.24  (29.04 – 62.18; 16.96) | 0.6027 |
| % time HbDiffx > 0.2 | Frontal | 21.69  (9.73 – 42.95; 16.75) | 15.46  (4.82 – 43.49; 15.46) | 0.4723 | 23.08  (11.43 – 32.48; 10.27) | 21.74  (13.8 – 35.33; 10.92) | 0.6417 | 22.09  (14.79 – 32.43; 8.9) | 31.42  (21.6 – 37.19; 8.59) | **0.0154** | 13.21  (0 – 30.49; 13.21) | 17.19  (4.4 – 33.4; 13.66) | 0.4111 |
|  | Parietal | 18.96  (5.14 – 39.46; 16.39) | 22.56  (6.9 – 44.46; 18.85) | 0.7687 | 18.51  (12.35 – 30.5; 9.14) | 21.4  (16.48 – 32.02; 7.06) | 0.4159 | 26.33  (14.8 – 32.96; 9.2) | 27.95  (15.01 – 35.06; 10.2) | 0.5649 | 14.2  (0.3 – 34.85; 14.2) | 18.21  (5.55 – 34.52; 14.06) | 0.5772 |
|  | Temporal | 23.27  (7.45 – 38.54; 15.68) | 15.13  (3.36 – 31.25; 12.72) | 0.1322 | 24.15  (10.16 – 30.96; 10.51) | 25.78  (12.15 – 35.38; 12.35) | 0.3043 | 24.42  (14.87 – 31.45; 8.96) | 24.97  (14.8 – 33.22; 9.89) | 0.6842 | 15.97  (2.99 – 33.6; 15.63) | 12.63  (0.42 – 27.54; 12.63) | 0.326 |
|  | Occipital | 16  (2.61 – 35.75; 16) | 22.71  (5.14 – 37.62; 15.74) | 0.8137 | 15.72  (8.79 – 23.61; 7.88) | 26.32  (11.65 – 34.53; 10.83) | **0.008** | 21.11  (12.68 – 32.1; 8.83) | 27.85  (18.4 – 39.88; 11.57) | **0.0163** | 6.65  (0 – 19.59; 6.65) | 13.53  (2.03 – 20.67; 9.96) | 0.2209 |
| % time HbDiffx > 0.3 | Frontal | 13.94  (0 – 28.23; 13.94) | 7.8  (0 – 23.06; 7.8) | 0.3595 | 12.48  (4.15 – 20.49; 8.36) | 14.81  (4.81 – 24.4; 10.3) | 0.5812 | 12.56  (8.53 – 22.53; 5.95) | 18.21  (12.41 – 25.32; 6.09) | **0.0262** | 2.35  (0 – 15.32; 2.35) | 6.07  (0 – 17.67; 6.07) | 0.3284 |
|  | Parietal | 11.56  (0.49 – 26.75; 11.56) | 14.09  (0 – 31.86; 14.09) | 0.9806 | 10.01  (5.36 – 16.65; 5.51) | 13.9  (5.77 – 21.41; 7.73) | 0.2479 | 16.77  (10.1 – 22.86; 6.54) | 18.14  (8.06 – 25.44; 9.34) | 0.9698 | 4.05  (0 – 21.45; 4.05) | 5.36  (0 – 17.24; 5.36) | 0.7467 |
|  | Temporal | 10.36  (0 – 27.91; 10.36) | 7.88  (0 – 17.98; 7.88) | 0.1727 | 10.63  (2.99 – 18.68; 7.69) | 13.18  (4.81 – 20.81; 8.28) | 0.4211 | 14.44  (6.6 – 22.7; 8.02) | 16.72  (6.77 – 23.44; 7.67) | 0.8469 | 2.57  (0 – 16.42; 2.57) | 2.04  (0 – 9.9; 2.04) | 0.3985 |
|  | Occipital | 0  (0 – 15.34; 0) | 11.4  (0 – 25.98; 11.4) | 0.108 | 5.55  (0 – 12.65; 5.55) | 12.98  (2.88 – 24.31; 11.19) | **0.0014** | 11.2  (5.56 – 18.15; 6.82) | 19.36  (10.59 – 29; 9.05) | **0.0024** | 0  (0 – 4.2; 0) | 1.81  (0 – 9.62; 1.81) | **0.0391** |
| **250 Hz Sampled Data** | | | | | | | | | | | | | |
| % time rSO_2_ > 30% | Frontal | 100 (100 – 100; 0) | 100 (100 – 100; 0) | **0.0123** | 100 (100 – 100; 0) | 100 (100 – 100; 0) | 0.1594 | 100 (100 – 100; 0) | 100 (100 – 100; 0) | 0.1682 | 100 (100 – 100; 0) | 100 (100 – 100; 0) | 0.0822 |
|  | Parietal | 100 (100 – 100; 0) | 100 (100 – 100; 0) | 0.1594 | 100 (100 – 100; 0) | 100 (100 – 100; 0) | 0.5836 | 100 (100 – 100; 0) | 100 (100 – 100; 0) | 0.1739 | 100 (100 – 100; 0) | 100 (100 – 100; 0) | 0.3271 |
|  | Temporal | 100 (100 – 100; 0) | 100 (100 – 100; 0) | 0.3271 | 100 (100 – 100; 0) | 100 (100 – 100; 0) | 1 | 100 (100 – 100; 0) | 100 (100 – 100; 0) | 0.3297 | 100 (100 – 100; 0) | 100 (100 – 100; 0) | 0.3271 |
|  | Occipital | 100 (100 – 100; 0) | 100 (100 – 100; 0) | 0.3271 | 100 (100 – 100; 0) | 100 (100 – 100; 0) | 1 | 100 (100 – 100; 0) | 100 (100 – 100; 0) | 0.3271 | 100 (100 – 100; 0) | 100 (100 – 100; 0) | 1 |
| % time rSO_2_ > 40% | Frontal | 100 (100 – 100; 0) | 56.19 (1.7 – 100; 43.81) | **<0.001** | 100 (100 – 100; 0) | 71.7 (0 – 100; 28.3) | **<0.001** | 100 (100 – 100; 0) | 64.03 (0.41 – 100; 35.97) | **<0.001** | 100 (100 – 100; 0) | 97.31 (0 – 100; 2.69) | **<0.001** |
|  | Parietal | 100 (69.19 – 100; 0) | 100 (90.32 – 100; 0) | 0.7046 | 100 (57.66 – 100; 0) | 100 (100 – 100; 0) | 0.3263 | 100 (89.37 – 100; 0) | 100 (88.05 – 100; 0) | 0.6131 | 100 (100 – 100; 0) | 100 (100 – 100; 0) | 0.6646 |
|  | Temporal | 59.1 (0 – 100; 40.9) | 100 (100 – 100; 0) | **<0.001** | 48.22 (0 – 100; 48.22) | 100 (100 – 100; 0) | **<0.001** | 59.81 (4.3 – 99.14; 40.19) | 100 (100 – 100; 0) | **<0.001** | 81.02 (0 – 100; 18.98) | 100 (100 – 100; 0) | **<0.001** |
|  | Occipital | 0 (0 – 0; 0) | 100 (100 – 100; 0) | **<0.001** | 0 (0 – 0; 0) | 100 (100 – 100; 0) | **<0.001** | 0 (0 – 0; 0) | 100 (93.4 – 100; 0) | **<0.001** | 0 (0 – 0; 0) | 100 (100 – 100; 0) | **<0.001** |
| % time rSO_2_ > 50% | Frontal | 0 (0 – 65.2; 0) | 0 (0 – 0; 0) | **0.0047** | 0 (0 – 97.62; 0) | 0 (0 – 0; 0) | **0.0017** | 0 (0 – 66.78; 0) | 0 (0 – 0; 0) | **<0.001** | 0 (0 – 100; 0) | 0 (0 – 0; 0) | **0.001** |
|  | Parietal | 36.69 (0 – 97.26; 36.69) | 0 (0 – 34.9; 0) | **<0.001** | 26.04 (0 – 100; 26.04) | 0 (0 – 37.56; 0) | **0.0205** | 35.38 (0 – 82.88; 35.38) | 0 (0 – 7.24; 0) | **<0.001** | 4.86 (0 – 100; 4.86) | 0 (0 – 0; 0) | **<0.001** |
|  | Temporal | 0 (0 – 0; 0) | 0 (0 – 37.45; 0) | **<0.001** | 0 (0 – 0; 0) | 0 (0 – 44.65; 0) | **<0.001** | 0 (0 – 0; 0) | 2.12 (0 – 47.25; 2.12) | **<0.001** | 0 (0 – 0; 0) | 0 (0 – 29.31; 0) | **<0.001** |
|  | Occipital | 0 (0 – 0; 0) | 0 (0 – 24.53; 0) | **<0.001** | 0 (0 – 0; 0) | 0 (0 – 27.38; 0) | **<0.001** | 0 (0 – 0; 0) | 0 (0 – 6.36; 0) | **<0.001** | 0 (0 – 0; 0) | 0 (0 – 0; 0) | **<0.001** |
| % time rSO_2_ > 60% | Frontal | 0 (0 – 0; 0) | 0 (0 – 0; 0) | 0.5519 | 0 (0 – 0; 0) | 0 (0 – 0; 0) | 0.3271 | 0 (0 – 0; 0) | 0 (0 – 0; 0) | 0.3271 | 0 (0 – 0; 0) | 0 (0 – 0; 0) | 0.3271 |
|  | Parietal | 0 (0 – 0; 0) | 0 (0 – 0; 0) | 0.6416 | 0 (0 – 0; 0) | 0 (0 – 0; 0) | 0.3004 | 0 (0 – 0; 0) | 0 (0 – 0; 0) | 0.1007 | 0 (0 – 0; 0) | 0 (0 – 0; 0) | 1 |
|  | Temporal | 0 (0 – 0; 0) | 0 (0 – 0; 0) | **0.0433** | 0 (0 – 0; 0) | 0 (0 – 0; 0) | 0.0822 | 0 (0 – 0; 0) | 0 (0 – 0; 0) | 0.5519 | 0 (0 – 0; 0) | 0 (0 – 0; 0) | 0.3271 |
|  | Occipital | 0 (0 – 0; 0) | 0 (0 – 0; 0) | 0.3271 | 0 (0 – 0; 0) | 0 (0 – 0; 0) | 0.1594 | 0 (0 – 0; 0) | 0 (0 – 0; 0) | 0.0822 | 0 (0 – 0; 0) | 0 (0 – 0; 0) | 1 |
| % time COx-a > 0 | Frontal | 52.4  (33.5 – 68.14; 18) | 58.38  (40.24 – 73.89; 16.35) | 0.4906 | 45.77  (39.91 – 60.72; 12.07) | 45.34  (30.36 – 57.09; 14.26) | 0.4199 | 45.66  (37.42 – 57.23; 9.72) | 50.42  (43.65 – 58.8; 7.58) | 0.18 | 49.42  (31.42 – 63.08; 17.44) | 55.13  (38.07 – 70.84; 16.85) | 0.2426 |
|  | Parietal | 44.7  (31.87 – 67.85; 20.15) | 44.38  (31.37 – 62.42; 13.61) | 0.7277 | 48.8  (36.16 – 62.16; 13.44) | 46  (34.66 – 57.98; 11.89) | 0.4339 | 45.34  (39.3 – 58.06; 8.94) | 49.94  (41.57 – 58.33; 8.66) | 0.517 | 57.38  (42.98 – 69.06; 12.03) | 49.63  (34.53 – 65.94; 16.15) | 0.1016 |
|  | Temporal | 62.53  (40 – 77.57; 19.43) | 47.83  (29.18 – 66.03; 18.75) | **0.0309** | 47.66  (40.08 – 61.61; 10.06) | 52.04  (40.01 – 70.05; 16.24) | 0.1184 | 48.71  (42.4 – 62.13; 11.26) | 50  (40.65 – 60.14; 10.09) | 0.9972 | 51.58  (34.15 – 71.72; 19.34) | 44.92  (34.1 – 63.14; 14.17) | 0.2963 |
|  | Occipital | 58.02  (40.59 – 74; 17.04) | 55.96  (37.5 – 73; 18.46) | 0.7538 | 51.26  (37.85 – 65.2; 14.4) | 50.89  (43.6 – 65.4; 13.51) | 0.9698 | 49.18  (39.5 – 57.97; 9.56) | 49.92  (42.69 – 61.24; 10.97) | 0.4777 | 43  (31.69 – 74.04; 21.53) | 44.79  (30.64 – 63.91; 14.82) | 0.6867 |
| % time COx-a > 0.2 | Frontal | 19.61  (6.96 – 44.14; 19.61) | 21.11  (7.14 – 38.86; 16.13) | 0.9641 | 19.01  (10.54 – 32.59; 9.66) | 22.89  (8.27 – 30.88; 12.64) | 0.9643 | 22.1  (13.48 – 34.02; 8.91) | 27.63  (21.84 – 36.49; 7.51) | **0.0205** | 16.99  (2.28 – 33.57; 16.23) | 15.38  (4.28 – 39.52; 14.71) | 0.3815 |
|  | Parietal | 18.14  (5.64 – 42.43; 16.46) | 23.59  (8.44 – 43; 15.92) | 0.5572 | 19.64  (13.4 – 30.14; 9.48) | 23.03  (15.67 – 32.99; 9.73) | 0.5372 | 23.09  (15.2 – 32.2; 8.46) | 27.19  (15.58 – 34.7; 10.19) | 0.5015 | 16.46  (2.13 – 36.78; 16.45) | 17.81  (5.71 – 32.46; 14.36) | 0.9448 |
|  | Temporal | 21.98  (5.88 – 42.09; 16.1) | 16.67  (3.74 – 27.82; 12.51) | 0.1229 | 19.19  (9.84 – 32.74; 10.39) | 25.85  (14.61 – 36.42; 11.51) | 0.1168 | 25.65  (15.92 – 33.01; 9.66) | 23.46  (15.63 – 32.68; 8.34) | 0.9808 | 13.36  (2.92 – 30.87; 12.73) | 9.25  (1.96 – 28.9; 9.25) | 0.3336 |
|  | Occipital | 16.5  (3.45 – 37.67; 14.41) | 25.46  (6.26 – 38.81; 16.11) | 0.6404 | 16.18  (6.98 – 21.76; 8.38) | 25.81  (13.82 – 35.63; 10.64) | **<0.001** | 18.26  (13.15 – 26.66; 7.18) | 26.79  (17.46 – 40.33; 10.55) | **0.0044** | 5.95  (0 – 27.9; 5.95) | 9.16  (2.57 – 19.29; 8.27) | 0.5021 |
| % time COx-a > 0.3 | Frontal | 13.38  (0 – 29.93; 13.38) | 11.27  (0 – 31.58; 11.27) | 0.8255 | 11.04  (3.71 – 20.58; 7.87) | 13.33  (2.56 – 22.12; 10.38) | 0.8306 | 12.31  (8.12 – 19.24; 5.42) | 18.04  (11.18 – 24.37; 6.65) | **0.0396** | 4.13  (0 – 16.35; 4.13) | 3.55  (0 – 21.35; 3.55) | 0.6289 |
|  | Parietal | 6.86  (0 – 28.85; 6.86) | 14.16  (0 – 28.09; 14.16) | 0.8419 | 10.78  (5.81 – 17.07; 6.27) | 13.96  (4.63 – 20.5; 7.69) | 0.5785 | 14.4  (9.36 – 21.17; 6.26) | 17.44  (7.3 – 25.58; 8.51) | 0.6148 | 3.61  (0 – 22.13; 3.61) | 4.01  (0 – 17.68; 4.01) | 0.8319 |
|  | Temporal | 8.21  (0 – 27.82; 8.21) | 7.7  (0 – 17.68; 7.7) | 0.3111 | 5.81  (2.04 – 17.5; 5.81) | 13.4  (6.39 – 23.93; 8.14) | 0.0637 | 13.74  (7.95 – 22.07; 7.26) | 12.94  (7.91 – 23.82; 8.46) | 0.9176 | 2.62  (0 – 13.31; 2.62) | 0  (0 – 9.5; 0) | 0.5961 |
|  | Occipital | 2.44  (0 – 16.45; 2.44) | 7.57  (0 – 25; 7.57) | 0.3584 | 4.39  (0 – 11.23; 4.39) | 12.23  (4.93 – 24.16; 10.57) | **<0.001** | 7.68  (4.72 – 13.74; 4.42) | 17.19  (9.22 – 27.57; 8.57) | **<0.001** | 0  (0 – 6.31; 0) | 1.44  (0 – 8.54; 1.44) | 0.5569 |
| % time HbOx > 0 | Frontal | 53.81  (31.79 – 61.77; 11.86) | 55.52  (26.02 – 68.84; 20.74) | 0.4971 | 47.83  (40.33 – 61.07; 10.64) | 48.1  (38.71 – 62.47; 13.02) | 0.9368 | 45.81  (33.51 – 57.84; 12.58) | 52.54  (42.27 – 61.19; 10.15) | **0.0244** | 51.52  (35.18 – 67.18; 16.67) | 50.53  (31.68 – 67.28; 16.95) | 0.9615 |
|  | Parietal | 50.62  (35.98 – 69.89; 17.45) | 52.38  (35.05 – 67.98; 16.09) | 0.7801 | 51.54  (39.5 – 61.34; 11.46) | 50.83  (36.46 – 64; 14.35) | 0.7123 | 48.08  (40.68 – 58.95; 8.88) | 53.68  (44.01 – 61.7; 9.56) | 0.3043 | 49.07  (34.25 – 68.32; 17.34) | 44.54  (33.32 – 64.98; 15.34) | 0.331 |
|  | Temporal | 52.09  (35.18 – 70.44; 18.2) | 44.72  (28.16 – 58.7; 15.87) | 0.225 | 49.2  (35.88 – 58.12; 11.56) | 48.78  (40.4 – 63.76; 12.16) | 0.2108 | 49.49  (43.79 – 56.84; 6.44) | 52.38  (42.05 – 62.98; 10.46) | 0.6691 | 47.46  (33.11 – 69.49; 19.87) | 41.32  (29.15 – 62.72; 12.51) | 0.1712 |
|  | Occipital | 53.39  (39.35 – 73.98; 16.82) | 49.06  (35 – 62.85; 14.81) | 0.1467 | 52.76  (40.13 – 62.28; 10.74) | 49.63  (38.79 – 59.99; 11.2) | 0.5214 | 50.57  (45.15 – 59.44; 6.41) | 54.53  (41.35 – 64.52; 12.4) | 0.6196 | 42.82  (22.58 – 69.54; 26.34) | 46.66  (30.19 – 65.37; 17.35) | 0.8469 |
| % time HbOx > 0.2 | Frontal | 19.31  (9.37 – 39.48; 15.32) | 18.18  (6.69 – 35.83; 14.14) | 0.8956 | 19.82  (14.61 – 31.41; 8.03) | 25.02  (16.01 – 39.26; 12.06) | 0.3328 | 23.81  (12.31 – 31.22; 9.98) | 30.23  (21.92 – 36.12; 7.91) | **0.0074** | 12.06  (1.56 – 30.6; 12.06) | 20.14  (4.42 – 31.22; 14.82) | 0.3128 |
|  | Parietal | 20.01  (6.02 – 42.81; 15.01) | 19.85  (10.15 – 43.29; 14.89) | 0.785 | 22.34  (15.65 – 34.03; 8.02) | 23.82  (14.96 – 36.18; 11.58) | 0.7433 | 28  (18.47 – 34.28; 7.71) | 31.59  (19.39 – 38.94; 10.36) | 0.2947 | 17.1  (2.79 – 34.41; 15.82) | 15.54  (5.45 – 28.78; 12.31) | 0.9835 |
|  | Temporal | 20.76  (5 – 35.61; 15.75) | 16.15  (3.86 – 31.03; 12.89) | 0.616 | 22.45  (9.17 – 29.64; 9.51) | 23.69  (15.97 – 34.38; 8.75) | 0.1225 | 25.13  (17.01 – 32.91; 8.19) | 27.03  (18.99 – 37.19; 8.7) | 0.3502 | 15.79  (1.38 – 31.91; 15.52) | 8.11  (1.66 – 22.65; 8.11) | 0.3487 |
|  | Occipital | 22.47  (6.51 – 44.5; 18.79) | 21.2  (5.32 – 37.92; 16.56) | 0.2908 | 21.47  (9.3 – 27.9; 8.25) | 21.24  (14.73 – 33.38; 10.36) | 0.4198 | 20.98  (14.06 – 33.07; 9.88) | 31.98  (19.08 – 40.37; 10.83) | 0.0603 | 5.65  (0 – 21; 5.65) | 9.46  (3.77 – 38.45; 9.46) | 0.1226 |
| % time HbOx > 0.3 | Frontal | 8.92  (0 – 19.32; 8.92) | 8.59  (0 – 28.64; 8.59) | 0.6311 | 11.81  (8.09 – 21.55; 5.14) | 15.12  (6.64 – 29.13; 10.45) | 0.5035 | 12.76  (6.25 – 20.83; 7.96) | 18.43  (12.47 – 25.75; 6.4) | **0.0095** | 2.26  (0 – 13.47; 2.26) | 3.67  (0 – 17.8; 3.67) | 0.1757 |
|  | Parietal | 6  (0 – 24.01; 6) | 11.01  (0 – 29.28; 11.01) | 0.7124 | 14.41  (7.03 – 20.02; 7.3) | 16.49  (4.86 – 26.15; 10.54) | 0.5191 | 19.8  (11.03 – 23.47; 5.41) | 19.59  (11.31 – 28.82; 8.45) | 0.6051 | 6.35  (0 – 17.76; 6.35) | 5.79  (0 – 18.47; 5.79) | 0.7574 |
|  | Temporal | 4.22  (0 – 22.99; 4.22) | 5.64  (0 – 20.79; 5.64) | 0.9319 | 10.91  (2.01 – 18.85; 8.75) | 14.39  (7.97 – 20.65; 6.47) | 0.1361 | 15.25  (8.16 – 22.25; 7.28) | 15.64  (10.32 – 25.36; 8.39) | 0.3965 | 5.44  (0 – 17.71; 5.44) | 0  (0 – 9.19; 0) | 0.1171 |
|  | Occipital | 8.96  (0 – 22.99; 8.96) | 3.36  (0 – 21.77; 3.36) | 0.3708 | 7.39  (1.12 – 13.8; 6.44) | 12.57  (5.12 – 21.07; 7.63) | **0.0182** | 12.18  (7.6 – 23.97; 7.68) | 20.68  (10.85 – 28.9; 9.42) | 0.0544 | 0  (0 – 6.32; 0) | 3.14  (0 – 19.64; 3.14) | **0.033** |
| % time HHbx > 0 | Frontal | 51.84  (31.77 – 70.67; 20.38) | 45.21  (22.21 – 60.74; 21.26) | 0.1822 | 54.4  (40.89 – 61.52; 10.68) | 54.93  (42.78 – 67.25; 12.32) | 0.4777 | 53.1  (38.74 – 61.75; 11.91) | 50.49  (40.08 – 58.84; 9.32) | 0.8174 | 50.06  (37.71 – 67.2; 16.28) | 50.26  (29.37 – 62.92; 19.42) | 0.6969 |
|  | Parietal | 49.24  (29.88 – 65.05; 17.33) | 53.88  (26.69 – 68.83; 19.54) | 0.5578 | 49.18  (37.67 – 64.14; 14.05) | 50.53  (40.85 – 60.98; 10.77) | 0.9917 | 52.8  (42.12 – 61.11; 9.66) | 55.62  (45.87 – 63.41; 8.89) | 0.438 | 46.86  (29.57 – 59.49; 16.81) | 44.69  (28.31 – 65.4; 18.18) | 0.967 |
|  | Temporal | 49.24  (30.08 – 66.67; 18.82) | 57.36  (33.24 – 70.21; 15.78) | 0.4119 | 56.28  (35.18 – 60.86; 12.31) | 48.92  (37.1 – 56.02; 10.18) | 0.1251 | 47.39  (39.33 – 57.61; 9.78) | 53.77  (40.71 – 63.16; 10.64) | 0.2496 | 53.31  (31.57 – 68.59; 16.94) | 50.27  (29.51 – 62.5; 16.53) | 0.5813 |
|  | Occipital | 43.3  (34.13 – 56.02; 11.65) | 50.24  (24.86 – 64.15; 17.73) | 0.8334 | 51.55  (40.8 – 61.43; 10.66) | 50.7  (36.25 – 61.05; 11.89) | 0.6148 | 55.3  (44.29 – 64.63; 10) | 50.2  (41.88 – 62.18; 10.11) | 0.3813 | 50.51  (29.29 – 71.06; 21.58) | 50.96  (36.09 – 68.39; 17.32) | 0.8577 |
| % time HHbx > 0.2 | Frontal | 26.39  (10.03 – 41.11; 15.13) | 17.32  (3.97 – 35.46; 14.49) | 0.2946 | 24.74  (14.53 – 34.01; 9.74) | 25.12  (17.43 – 39.14; 10.46) | 0.5418 | 28.74  (19.13 – 35.61; 8.55) | 29.08  (22.39 – 35.95; 7.01) | 0.4586 | 15.47  (2.63 – 30.22; 13.2) | 14.42  (1.93 – 32.7; 13.04) | 0.989 |
|  | Parietal | 18.8  (6.25 – 33.33; 14.53) | 21.53  (10.67 – 38.61; 15.11) | 0.5709 | 25.32  (13.4 – 36.59; 12.18) | 26.54  (13.77 – 33.85; 11.66) | 0.9725 | 25.32  (17.54 – 37.13; 9.72) | 28.66  (20.44 – 41.42; 9.12) | 0.2553 | 12.5  (4.04 – 26.12; 10.75) | 13.52  (2.36 – 31.89; 13.52) | 0.9697 |
|  | Temporal | 18  (4.36 – 42.15; 15.56) | 26.16  (10.22 – 40.25; 14.88) | 0.3582 | 24.27  (15.14 – 33.51; 9.4) | 21.06  (9.03 – 30.84; 10.8) | 0.3449 | 24.51  (16.72 – 32.77; 8.22) | 27.83  (19.42 – 38.13; 9.22) | 0.2096 | 16.5  (2.28 – 31.03; 14.47) | 10.68  (1.73 – 24.13; 10.68) | 0.3106 |
|  | Occipital | 12.42  (3.88 – 28.96; 11.55) | 18.63  (4.09 – 29.96; 14.46) | 0.4311 | 17.78  (7.23 – 24.89; 9.76) | 21.96  (13.71 – 30.2; 8.68) | 0.109 | 20.5  (13.07 – 30.03; 8.19) | 28.71  (21.18 – 38.13; 9.33) | **0.0233** | 11.01  (0 – 21.92; 11.01) | 15.32  (4.47 – 36.17; 13.74) | 0.0563 |
| % time HHbx > 0.3 | Frontal | 12.02  (0 – 26.22; 12.02) | 7.37  (0.49 – 20.79; 7.37) | 0.6988 | 14.05  (6.9 – 24.69; 8.86) | 16.75  (7.78 – 27.16; 9.81) | 0.5509 | 15.7  (11.1 – 25; 6.74) | 19.21  (12.67 – 26.35; 7.11) | 0.2626 | 6.02  (0 – 17.55; 6.02) | 2.78  (0 – 16.15; 2.78) | 0.7448 |
|  | Parietal | 10.27  (0 – 21.42; 10.27) | 11.92  (0.64 – 25.37; 11.92) | 0.4182 | 12.48  (6.09 – 20.17; 7.52) | 12.05  (7.63 – 24.67; 8.65) | 0.6565 | 17.33  (10.24 – 24.21; 7.06) | 18.31  (12.2 – 32.5; 6.73) | 0.2931 | 3.78  (0 – 15.14; 3.78) | 2.37  (0 – 22.41; 2.37) | 0.7685 |
|  | Temporal | 5.28  (0 – 21.32; 5.28) | 10.23  (0 – 24.3; 10.23) | 0.5736 | 12.11  (3.53 – 23.37; 9.43) | 11.97  (1.16 – 22.1; 10.59) | 0.5619 | 14.02  (8.43 – 22.6; 6.86) | 17.52  (11.19 – 28.24; 8.83) | 0.18 | 2.99  (0 – 15.41; 2.99) | 0  (0 – 12.77; 0) | 0.4777 |
|  | Occipital | 0  (0 – 9.04; 0) | 7.63  (0 – 16.55; 7.63) | 0.0467 | 4.91  (0.83 – 12.56; 4.91) | 13.89  (6 – 21.76; 7.98) | **0.0013** | 12.26  (4.06 – 17.71; 7.14) | 17.41  (11.02 – 28.01; 8.75) | **0.0091** | 0  (0 – 4.13; 0) | 4.18  (0 – 21.62; 4.18) | **<0.001** |
| % time tHbx > 0 | Frontal | 53.33  (32.51 – 63.81; 16.19) | 47.68  (27.69 – 70.67; 21.14) | 0.6491 | 52.4  (36.92 – 60.36; 13.27) | 48.12  (38.74 – 62.21; 12.05) | 0.812 | 49.68  (34.92 – 60.03; 13.02) | 52.47  (44.91 – 59.15; 7.44) | 0.3345 | 47.03  (38.19 – 63.21; 14.6) | 50.95  (26.09 – 65.93; 19.57) | 0.8388 |
|  | Parietal | 45.66  (31.77 – 68.59; 18.3) | 50.58  (32.15 – 65.62; 17.65) | 0.9395 | 51.38  (36.71 – 65.98; 14.98) | 47.34  (38.95 – 64.74; 14.2) | 0.9313 | 51.56  (38.78 – 59.69; 11.59) | 54.17  (44.74 – 60.88; 8.44) | 0.3683 | 43.12  (30.39 – 67.82; 16.26) | 43.87  (34.48 – 64.11; 13.32) | 0.945 |
|  | Temporal | 50  (41.78 – 74.02; 17.03) | 49.37  (30.55 – 66.67; 17.3) | 0.2611 | 48.43  (42 – 59.15; 7.62) | 46.61  (38.32 – 55.93; 8.67) | 0.4586 | 49.29  (41.91 – 58.77; 7.59) | 51.56  (43.58 – 64.22; 9.54) | 0.3965 | 59.5  (34.48 – 73.23; 20.97) | 44  (35.03 – 61.2; 15.37) | 0.1669 |
|  | Occipital | 56.39  (38.22 – 68.31; 14.07) | 50  (34.29 – 64.27; 14.93) | 0.2468 | 53.1  (40.18 – 65.8; 12.99) | 51.57  (34.39 – 59.48; 11.07) | 0.3775 | 50  (43.63 – 59.83; 7.5) | 52.21  (42.64 – 63.97; 11.6) | 0.8281 | 43.88  (24.18 – 60.66; 19.08) | 50.45  (30.23 – 68.08; 20) | 0.3208 |
| % time tHbx > 0.2 | Frontal | 24.57  (9.37 – 39.85; 15.45) | 25.54  (10.68 – 35.13; 12.26) | 0.9807 | 25.84  (16.45 – 38.08; 10.45) | 24.54  (17.11 – 34.76; 9.08) | 0.6817 | 24.42  (14.2 – 35.55; 10.91) | 27.99  (22.7 – 35.78; 7.27) | 0.0523 | 12.57  (2 – 32.09; 12.57) | 15.04  (1.88 – 29.73; 13.38) | 0.887 |
|  | Parietal | 17.91  (10.84 – 33.33; 12.33) | 17.16  (9.14 – 35.22; 15.5) | 0.8875 | 25.06  (15.4 – 34.42; 9.68) | 24.6  (11.15 – 41.05; 14.47) | 0.9396 | 28.05  (17.95 – 34.99; 9.14) | 29.17  (21.96 – 39.33; 9.2) | 0.3158 | 18.15  (5.87 – 32.29; 12.46) | 14.72  (3.96 – 26.82; 11.28) | 0.4709 |
|  | Temporal | 20.29  (10.53 – 34.71; 13.27) | 21.96  (4.5 – 35; 14.88) | 0.8143 | 21.63  (12.7 – 32.92; 10.62) | 23.61  (14.9 – 29.93; 7.35) | 0.9231 | 26.4  (20.08 – 35.12; 7.25) | 31.19  (20.85 – 39.35; 9.2) | 0.3965 | 17.99  (0.47 – 32.47; 16.7) | 9.11  (1.94 – 22.34; 8.4) | 0.2735 |
|  | Occipital | 20.82  (5.14 – 39.8; 16.56) | 23.11  (8.75 – 35.29; 13) | 0.8903 | 22.72  (13.54 – 32.02; 9.44) | 21.86  (12.53 – 35.92; 12.52) | 0.7355 | 24.28  (15.67 – 32.86; 8.8) | 29.74  (18.96 – 40.7; 11.06) | 0.1962 | 5.48  (0 – 27.96; 5.48) | 16.37  (5.92 – 39.51; 12.52) | **0.008** |
| % time tHbx > 0.3 | Frontal | 8.44  (0 – 25.99; 8.44) | 12.31  (2.56 – 25.34; 12.31) | 0.6165 | 14.45  (5.37 – 28.43; 10.41) | 15.14  (8.94 – 25.57; 7.88) | 0.8523 | 14.53  (7.67 – 22.54; 7.73) | 18.59  (14.38 – 25.93; 6.33) | **0.0155** | 1.49  (0 – 13.44; 1.49) | 4.52  (0 – 15.01; 4.52) | 0.4921 |
|  | Parietal | 7.22  (0 – 20.77; 7.22) | 10  (0.45 – 22.86; 10) | 0.6597 | 16.01  (5.9 – 23.97; 9.61) | 15.53  (2.57 – 27.68; 12.9) | 0.9505 | 18.26  (11.06 – 24.59; 7.19) | 17.07  (12.75 – 31.29; 8.25) | 0.6417 | 5.75  (0.29 – 14.74; 5.75) | 4.26  (0 – 14.82; 4.26) | 0.5128 |
|  | Temporal | 5.56  (0 – 17.81; 5.56) | 11.25  (0 – 21.46; 11.25) | 0.4671 | 12.33  (5.07 – 22.11; 8.73) | 12.91  (6.9 – 19.05; 6.15) | 0.9149 | 17.21  (10.23 – 28.05; 7.43) | 21.52  (12.24 – 27.95; 8.63) | 0.352 | 2.76  (0 – 20.38; 2.76) | 0  (0 – 12.39; 0) | 0.2383 |
|  | Occipital | 11.81  (0 – 30.18; 11.81) | 7.8  (0 – 22.55; 7.8) | 0.6321 | 12.63  (4.1 – 19.31; 8.5) | 13.62  (4.93 – 22; 8.65) | 0.5555 | 15.66  (7.77 – 24.42; 8.22) | 19.83  (11.44 – 30.56; 9.28) | 0.1302 | 0  (0 – 10.11; 0) | 3.98  (0 – 25.65; 3.98) | **0.017** |
| % time HbDiffx > 0 | Frontal | 52.74  (36.46 – 70.13; 17.34) | 55.56  (39.65 – 73.18; 16.72) | 0.5719 | 46.93  (39.42 – 61.4; 11.04) | 49.2  (34.17 – 58.01; 13.02) | 0.7277 | 49.01  (37.08 – 57.16; 11.33) | 51.5  (45.06 – 60.14; 8.4) | 0.0714 | 50.34  (30.31 – 65.33; 18.93) | 53.09  (38.39 – 69.65; 16.33) | 0.4906 |
|  | Parietal | 43.84  (31.61 – 66.67; 20.94) | 41.14  (31.53 – 60.83; 13.78) | 0.7097 | 48.24  (38.57 – 62.11; 12.9) | 46.31  (36.04 – 58.69; 11.82) | 0.6442 | 46.65  (39.75 – 60.02; 9.62) | 50.34  (41.94 – 56.86; 7.88) | 0.8713 | 54.88  (43.01 – 68.49; 13.48) | 51.28  (37.45 – 63.35; 13.33) | 0.2761 |
|  | Temporal | 64.72  (40.29 – 79.61; 19.54) | 45.34  (28.96 – 64.44; 17.17) | **0.0097** | 45.71  (38.83 – 56.64; 10.42) | 51.56  (41.38 – 69.27; 15.26) | 0.0946 | 48.67  (40.82 – 61.95; 10.44) | 50.87  (41.01 – 59.47; 8.82) | 0.9533 | 58.99  (37.99 – 77.74; 19.09) | 44.92  (32.85 – 60.62; 13.11) | **0.0413** |
|  | Occipital | 58.02  (39.52 – 73.33; 15.86) | 56.38  (32.82 – 74.03; 20.19) | 0.5464 | 53.57  (37.06 – 63.27; 12.49) | 51.21  (38.68 – 63.47; 12.75) | 0.8903 | 50.13  (42.99 – 58.79; 8.14) | 53.23  (41.72 – 63.01; 11.33) | 0.7407 | 44.96  (27.89 – 74.04; 20.94) | 44.1  (29.1 – 63.82; 16.56) | 0.6442 |
| % time HbDiffx > 0.2 | Frontal | 22.55  (9.73 – 43.42; 17.15) | 17.91  (4.47 – 43.16; 17.24) | 0.5452 | 22.95  (11.13 – 31.99; 10.94) | 24.16  (13.06 – 33.59; 10.02) | 0.5672 | 20.99  (14.49 – 33.09; 9.33) | 31.19  (22.8 – 36.73; 7.65) | **0.014** | 18.27  (2.78 – 30.06; 13.56) | 15.02  (4.28 – 38.05; 12.36) | 0.6986 |
|  | Parietal | 19.33  (6.23 – 42.27; 14.89) | 23.33  (6.27 – 41.13; 17.37) | 0.8011 | 19.29  (11.72 – 28.24; 8.47) | 22.27  (15.41 – 32.63; 9.21) | 0.4139 | 25.14  (14.52 – 32.7; 10.02) | 27.73  (15.79 – 35.45; 9.31) | 0.5125 | 14.97  (2.52 – 34.2; 14.97) | 18.39  (6.71 – 30.53; 12.1) | 0.8113 |
|  | Temporal | 21.68  (4.78 – 37.07; 16.57) | 16.11  (3.36 – 30; 13.44) | 0.1857 | 20.06  (10.53 – 30.95; 10.2) | 27.34  (13.34 – 33.69; 11.62) | 0.1383 | 25.47  (15.83 – 31.36; 8.72) | 25.3  (17.27 – 33.14; 8.07) | 0.6817 | 15.24  (2.49 – 26.7; 12.64) | 8.43  (0 – 28.39; 8.43) | 0.3049 |
|  | Occipital | 21.53  (2.46 – 42.42; 19.57) | 23.93  (2.74 – 37.51; 15.92) | 0.7843 | 16.35  (7.74 – 25.72; 9.48) | 25.1  (11.65 – 33.95; 11.06) | **0.0167** | 19.74  (13.81 – 32.08; 8.12) | 28.07  (17.23 – 39.21; 11.19) | **0.0271** | 6.58  (0 – 22.47; 6.58) | 8.19  (1.94 – 18.56; 8.19) | 0.495 |
| % time HbDiffx > 0.3 | Frontal | 12.7  (0 – 25.8; 12.7) | 7.49  (0 – 26.67; 7.49) | 0.4781 | 12.83  (4.02 – 21.75; 8.87) | 16.59  (4.8 – 22.45; 10.82) | 0.5717 | 12.28  (7.66 – 22.57; 6.19) | 18.06  (12.27 – 25.48; 6.61) | **0.0145** | 2.48  (0 – 15.45; 2.48) | 4.02  (0 – 18.71; 4.02) | 0.3549 |
|  | Parietal | 8.09  (2.09 – 28.54; 8.09) | 8.53  (0 – 32.5; 8.53) | 0.7721 | 11.54  (6.13 – 16.34; 5.22) | 13.67  (5.53 – 21.18; 8.13) | 0.3204 | 15.46  (8.72 – 23.17; 7.42) | 17.15  (7.42 – 25.72; 9.1) | 0.8388 | 1.72  (0 – 21.11; 1.72) | 6.58  (0 – 17.66; 6.58) | 0.557 |
|  | Temporal | 8.81  (0 – 27.91; 8.81) | 6.94  (0 – 17.58; 6.94) | 0.2445 | 10.11  (2.1 – 18.8; 8.34) | 14.24  (6.49 – 21.37; 7.55) | 0.1322 | 15.14  (7.15 – 22.05; 7.22) | 14.68  (8.29 – 23.4; 8.01) | 0.8067 | 1.68  (0 – 11.3; 1.68) | 0  (0 – 11.07; 0) | 0.5637 |
|  | Occipital | 3.48  (0 – 20.38; 3.48) | 6.07  (0 – 23.46; 6.07) | 0.9031 | 5.07  (0.2 – 12.14; 5.07) | 12.65  (1.86 – 24.21; 11.03) | **0.002** | 10.45  (4.01 – 19.52; 6.81) | 19.18  (10.24 – 30.29; 9.82) | **0.003** | 0  (0 – 4.38; 0) | 0.66  (0 – 9.45; 0.66) | 0.2365 |
| The p-values in the table are derived using Mann-Whitney U test between the bilateral signals for the perturbation subgroups. *COx-a, cerebral oximetry index with arterial blood pressure; CVR, cerebrovascular reactivity index; HbDiffx, hemoglobin difference index; HbOx, oxyhemoglobin index; HHbx, deoxyhemoglobin index; IQR, interquartile range; MAD, median absolute deviation; rSO_2_, regional cerebral oxygen saturation; tHbx, total hemoglobin index.* | | | | | | | | | | | | | |

Appendix S7e: Perturbation Subgrouped Percent Time Results of rSO_2_ Using Raw Data

| **Physiologic Variable** | **Brain Lobe** | **Baseline** | | | **Neurovascular Coupling** | | | **Orthostatic Challenge** | | | **Vascular Chemo-Reactivity** | | |
| --- | --- | --- | --- | --- | --- | --- | --- | --- | --- | --- | --- | --- | --- |
|  |  | **Median (IQR)** | | **p-value** | **Median (IQR)** | | **p-value** | **Median (IQR)** | | **p-value** | **Median (IQR)** | | **p-value** |
|  |  | **Left Hemisphere** | **Right Hemisphere** |  | **Left Hemisphere** | **Right Hemisphere** |  | **Left Hemisphere** | **Right Hemisphere** |  | **Left Hemisphere** | **Right Hemisphere** |  |
| **1 Hz Sampled Data** | | | | | | | | | | | | | |
| % time rSO_2_ > 30% | Frontal | 100 (100 – 100; 0) | 100 (100 – 100; 0) | **0.0123** | 100 (100 – 100; 0) | 100 (100 – 100; 0) | 0.3198 | 100 (100 – 100; 0) | 100 (100 – 100; 0) | 0.1626 | 100 (100 – 100; 0) | 100 (100 – 100; 0) | 0.1653 |
|  | Parietal | 100 (100 – 100; 0) | 100 (100 – 100; 0) | 0.3812 | 100 (100 – 100; 0) | 100 (100 – 100; 0) | 0.2818 | 100 (100 – 100; 0) | 100 (100 – 100; 0) | 0.3846 | 100 (100 – 100; 0) | 100 (100 – 100; 0) | 0.4624 |
|  | Temporal | 100 (100 – 100; 0) | 100 (100 – 100; 0) | 0.1594 | 100 (100 – 100; 0) | 100 (100 – 100; 0) | 0.1594 | 100 (100 – 100; 0) | 100 (100 – 100; 0) | 0.1688 | 100 (100 – 100; 0) | 100 (100 – 100; 0) | 0.6416 |
|  | Occipital | 100 (100 – 100; 0) | 100 (100 – 100; 0) | **0.0172** | 100 (100 – 100; 0) | 100 (100 – 100; 0) | 0.0822 | 99.93 (99.81 – 100; 0.07) | 100 (100 – 100; 0) | **<0.001** | 100 (99.8 – 100; 0) | 100 (100 – 100; 0) | **<0.001** |
| % time rSO_2_ > 40% | Frontal | 100 (100 – 100; 0) | 57.7 (1.86 – 100; 42.3) | **<0.001** | 100 (100 – 100; 0) | 72.67 (0 – 100; 27.33) | **<0.001** | 100 (100 – 100; 0) | 63.63 (0.37 – 99.86; 36.37) | **<0.001** | 100 (100 – 100; 0) | 95.5 (0 – 100; 4.5) | **<0.001** |
|  | Parietal | 100 (69.14 – 100; 0) | 100 (83.46 – 100; 0) | 0.5873 | 100 (56.77 – 100; 0) | 100 (100 – 100; 0) | 0.2781 | 100 (88.01 – 100; 0) | 100 (86.95 – 100; 0) | 0.4595 | 100 (100 – 100; 0) | 100 (100 – 100; 0) | 0.9924 |
|  | Temporal | 57.48 (1.96 – 99.2; 42.44) | 100 (100 – 100; 0) | **<0.001** | 45.93 (0.18 – 100; 45.93) | 100 (100 – 100; 0) | **<0.001** | 59.78 (5.51 – 98.23; 40.22) | 100 (100 – 100; 0) | **<0.001** | 76.74 (0.59 – 100; 23.26) | 100 (100 – 100; 0) | **<0.001** |
|  | Occipital | 0.14 (0 – 0.91; 0.14) | 100 (100 – 100; 0) | **<0.001** | 0.04 (0 – 0.44; 0.04) | 100 (100 – 100; 0) | **<0.001** | 0.44 (0 – 2.46; 0.44) | 100 (91.64 – 100; 0) | **<0.001** | 0.4 (0 – 2.04; 0.4) | 100 (100 – 100; 0) | **<0.001** |
| % time rSO_2_ > 50% | Frontal | 0 (0 – 64.28; 0) | 0 (0 – 0; 0) | **0.0043** | 0 (0 – 96.8; 0) | 0 (0 – 0; 0) | **0.0022** | 0 (0 – 64.52; 0) | 0 (0 – 0; 0) | **<0.001** | 0 (0 – 99.54; 0) | 0 (0 – 0; 0) | **<0.001** |
|  | Parietal | 34.8 (4.97 – 95.97; 34.8) | 0 (0 – 35.62; 0) | **<0.001** | 23.67 (0 – 100; 23.67) | 0 (0 – 36.44; 0) | **0.0036** | 35.43 (0.28 – 80.69; 35.43) | 0.17 (0 – 7.21; 0.17) | **<0.001** | 15.7 (0 – 98.58; 15.7) | 0 (0 – 0; 0) | **<0.001** |
|  | Temporal | 0 (0 – 0; 0) | 0 (0 – 39.9; 0) | **<0.001** | 0 (0 – 0; 0) | 0 (0 – 44.3; 0) | **<0.001** | 0 (0 – 0; 0) | 2.21 (0 – 47.05; 2.21) | **<0.001** | 0 (0 – 0; 0) | 0 (0 – 27.64; 0) | **0.0013** |
|  | Occipital | 0 (0 – 0; 0) | 0.15 (0 – 18.7; 0.15) | **<0.001** | 0 (0 – 0; 0) | 0 (0 – 34.33; 0) | **<0.001** | 0 (0 – 0; 0) | 0.71 (0 – 6.85; 0.71) | **<0.001** | 0 (0 – 0; 0) | 0 (0 – 1.3; 0) | **<0.001** |
| % time rSO_2_ > 60% | Frontal | 0 (0 – 0; 0) | 0 (0 – 0; 0) | 0.6416 | 0 (0 – 0; 0) | 0 (0 – 0; 0) | 0.5676 | 0 (0 – 0; 0) | 0 (0 – 0; 0) | 0.6947 | 0 (0 – 0; 0) | 0 (0 – 0; 0) | 0.5598 |
|  | Parietal | 0 (0 – 0; 0) | 0 (0 – 0; 0) | **0.0164** | 0 (0 – 0; 0) | 0 (0 – 0; 0) | **0.0286** | 0 (0 – 0; 0) | 0 (0 – 0; 0) | **0.014** | 0 (0 – 0; 0) | 0 (0 – 0; 0) | 0.3198 |
|  | Temporal | 0 (0 – 0; 0) | 0 (0 – 0; 0) | **0.0231** | 0 (0 – 0; 0) | 0 (0 – 0; 0) | **0.0822** | 0 (0 – 0; 0) | 0 (0 – 0; 0) | 0.0973 | 0 (0 – 0; 0) | 0 (0 – 0; 0) | **0.0433** |
|  | Occipital | 0 (0 – 0; 0) | 0 (0 – 0; 0) | 0.0822 | 0 (0 – 0; 0) | 0 (0 – 0; 0) | **0.0822** | 0 (0 – 0; 0) | 0 (0 – 0; 0) | **0.0065** | 0 (0 – 0; 0) | 0 (0 – 0; 0) | 0.3271 |
| **250 Hz Sampled Data** | | | | | | | | | | | | | |
| % time rSO_2_ > 30% | Frontal | 100 (100 – 100; 0) | 100 (100 – 100; 0) | 0.0984 | 100 (100 – 100; 0) | 100 (100 – 100; 0) | 0.4084 | 100 (100 – 100; 0) | 100 (100 – 100; 0) | 0.0601 | 100 (100 – 100; 0) | 100 (100 – 100; 0) | 0.2881 |
|  | Parietal | 99.98 (92.32 – 100; 0.02) | 100 (99.99 – 100; 0) | **0.0311** | 100 (95.7 – 100; 0) | 100 (100 – 100; 0) | **0.0479** | 99.99 (96.45 – 100; 0.01) | 100 (99.36 – 100; 0) | 0.1186 | 100 (99.74 – 100; 0) | 100 (100 – 100; 0) | **0.0489** |
|  | Temporal | 100 (88.31 – 100; 0) | 100 (98.52 – 100; 0) | 0.729 | 100 (92.7 – 100; 0) | 100 (99.16 – 100; 0) | 0.2533 | 100 (95.46 – 100; 0) | 100 (99.95 – 100; 0) | 0.1657 | 100 (99.98 – 100; 0) | 100 (99.97 – 100; 0) | 0.9824 |
|  | Occipital | 75.65 (73.35 – 76.8; 1.63) | 100 (97.84 – 100; 0) | **<0.001** | 75.37 (73.1 – 77.14; 2.05) | 100 (99.85 – 100; 0) | **<0.001** | 75.11 (73.59 – 77.09; 1.86) | 100 (97.48 – 100; 0) | **<0.001** | 75.39 (73.32 – 77.14; 1.88) | 100 (100 – 100; 0) | **<0.001** |
| % time rSO_2_ > 40% | Frontal | 100 (100 – 100; 0) | 59.24 (2.38 – 99.48; 40.68) | **<0.001** | 100 (100 – 100; 0) | 64.43 (0.06 – 100; 35.57) | **<0.001** | 100 (99.96 – 100; 0) | 67.64 (0.37 – 99.14; 32.36) | **<0.001** | 100 (100 – 100; 0) | 92.93 (0 – 100; 7.07) | **<0.001** |
|  | Parietal | 97.48 (72.17 – 99.99; 2.52) | 99.87 (83.36 – 100; 0.13) | 0.09 | 98.05 (61.1 – 100; 1.95) | 100 (93.4 – 100; 0) | **0.0094** | 97.45 (78.33 – 99.95; 2.55) | 99.53 (79.33 – 100; 0.47) | 0.4328 | 99.44 (90.16 – 100; 0.56) | 100 (87.22 – 100; 0) | 0.1188 |
|  | Temporal | 51.85 (32.26 – 94.16; 28.84) | 100 (89.26 – 100; 0) | **<0.001** | 49.38 (27.79 – 98.92; 44.3) | 100 (89.31 – 100; 0) | **<0.001** | 60.41 (32.83 – 89.25; 28.16) | 100 (95.12 – 100; 0) | **<0.001** | 66.82 (27.99 – 99.3; 33.18) | 100 (93.34 – 100; 0) | **<0.001** |
|  | Occipital | 29.29 (22.62 – 33.59; 5.86) | 99.78 (83.07 – 100; 0.22) | **<0.001** | 29.26 (22.68 – 34.02; 5.86) | 99.98 (92.74 – 100; 0.02) | **<0.001** | 28.97 (23.38 – 33.7; 5.26) | 98.98 (81.98 – 100; 1.02) | **<0.001** | 28.82 (23.42 – 34.99; 6) | 99.99 (96.26 – 100; 0.01) | **<0.001** |
| % time rSO_2_ > 50% | Frontal | 0 (0 – 64.77; 0) | 0 (0 – 0.16; 0) | **0.0123** | 0 (0 – 80.4; 0) | 0 (0 – 0; 0) | **0.0037** | 0 (0 – 61.48; 0) | 0 (0 – 0.03; 0) | **0.0072** | 0 (0 – 90.96; 0) | 0 (0 – 0; 0) | **0.0022** |
|  | Parietal | 45.82 (17.78 – 77.55; 29.62) | 7.57 (0.03 – 33.45; 7.57) | **<0.001** | 36.54 (8.04 – 76.6; 34.08) | 4.6 (0 – 44.59; 4.6) | **0.0014** | 41.59 (6.55 – 65.21; 30.17) | 2.7 (0.15 – 16.74; 2.7) | **<0.001** | 43.22 (3.08 – 74.41; 38.72) | 0 (0 – 6.7; 0) | **<0.001** |
|  | Temporal | 0.03 (0 – 13.46; 0.03) | 16.02 (0.01 – 38.92; 16.02) | **<0.001** | 0 (0 – 12.53; 0) | 13.33 (0.1 – 44.38; 13.33) | **<0.001** | 0.06 (0 – 13.43; 0.06) | 13.39 (0.05 – 47.48; 13.39) | **<0.001** | 0 (0 – 8.35; 0) | 6.28 (0 – 41.52; 6.28) | **0.0105** |
|  | Occipital | 7.54 (2.84 – 10.13; 4.4) | 15.08 (0 – 39.45; 15.08) | 0.1316 | 7.45 (3.24 – 10.57; 4.14) | 13.87 (0 – 41.33; 13.87) | 0.3562 | 7.19 (2.39 – 10.89; 4.48) | 7.6 (0.13 – 23.44; 7.6) | 0.8659 | 6.48 (2.14 – 12; 4.65) | 0.58 (0 – 18.44; 0.58) | 0.2218 |
| % time rSO_2_ > 60% | Frontal | 0 (0 – 0; 0) | 0 (0 – 0; 0) | 0.7587 | 0 (0 – 0; 0) | 0 (0 – 0; 0) | 0.7772 | 0 (0 – 0; 0) | 0 (0 – 0; 0) | 0.9635 | 0 (0 – 0; 0) | 0 (0 – 0; 0) | 0.7025 |
|  | Parietal | 1.54 (0.01 – 7.77; 1.54) | 0 (0 – 0.9; 0) | **<0.001** | 0.46 (0 – 6.21; 0.46) | 0 (0 – 0.68; 0) | **0.0033** | 0.7 (0 – 10.29; 0.7) | 0 (0 – 0.73; 0) | **0.0012** | 0 (0 – 6.68; 0) | 0 (0 – 0.04; 0) | **0.0087** |
|  | Temporal | 0 (0 – 0.86; 0) | 0 (0 – 3.36; 0) | 0.1547 | 0 (0 – 0.26; 0) | 0 (0 – 2.43; 0) | 0.2639 | 0 (0 – 1.28; 0) | 0 (0 – 2.63; 0) | 0.6934 | 0 (0 – 0.11; 0) | 0 (0 – 2.13; 0) | 0.3934 |
|  | Occipital | 0.03 (0 – 0.71; 0.03) | 0 (0 – 3.36; 0) | 0.3414 | 0.01 (0 – 0.65; 0.01) | 0 (0 – 3.59; 0) | 0.7772 | 0.03 (0 – 1.05; 0.03) | 0.01 (0 – 2.45; 0.01) | 0.9082 | 0 (0 – 1.09; 0) | 0 (0 – 2.52; 0) | 0.8414 |
| The p-values in the table are derived using Mann-Whitney U test between the bilateral signals for the perturbation subgroups. *IQR, interquartile range; MAD, median absolute deviation; rSO_2_, regional cerebral oxygen saturation.* | | | | | | | | | | | | | |

Appendix S7f: Perturbation Subgrouped Regional Hemispheric Disparity Analysis on CVR Indices Using 10-Second Decimated Data

| **Brain Lobe** | **Physiologic Variable** | **Median (IQR)** | | | |
| --- | --- | --- | --- | --- | --- |
|  |  | **Baseline** | **Neurovascular Coupling** | **Orthostatic Challenge** | **Vascular Chemo-Reactivity** |
| **1 Hz Sampled Data** | | | | | |
| Frontal | ARHD of COx-a (au) | 0.23 (0.13 – 0.36) | 0.21 (0.1 – 0.36) | 0.25 (0.11 – 0.45) | 0.18 (0.1 – 0.3) |
|  | ARHD of HbOx (au) | 0.26 (0.15 – 0.39) | 0.24 (0.1 – 0.4) | 0.22 (0.1 – 0.42) | 0.21 (0.1 – 0.36) |
|  | ARHD of HHbx (au) | 0.24 (0.11 – 0.41) | 0.22 (0.1 – 0.36) | 0.24 (0.09 – 0.46) | 0.19 (0.09 – 0.32) |
|  | ARHD of tHbx (au) | 0.24 (0.14 – 0.43) | 0.26 (0.12 – 0.44) | 0.2 (0.1 – 0.41) | 0.2 (0.1 – 0.34) |
|  | ARHD of HbDiffx (au) | 0.21 (0.13 – 0.34) | 0.22 (0.1 – 0.37) | 0.24 (0.11 – 0.46) | 0.17 (0.1 – 0.31) |
|  | MAD of ARHD COx-a (au) | 0.09 (0.07 – 0.15) | 0.13 (0.1 – 0.15) | 0.15 (0.13 – 0.19) | 0.08 (0.06 – 0.12) |
|  | MAD of ARHD HbOx (au) | 0.1 (0.08 – 0.15) | 0.13 (0.1 – 0.17) | 0.14 (0.11 – 0.21) | 0.1 (0.08 – 0.14) |
|  | MAD of ARHD HHbx (au) | 0.11 (0.07 – 0.13) | 0.12 (0.1 – 0.15) | 0.16 (0.12 – 0.21) | 0.1 (0.06 – 0.15) |
|  | MAD of ARHD tHbx (au) | 0.12 (0.07 – 0.16) | 0.15 (0.11 – 0.18) | 0.13 (0.11 – 0.16) | 0.1 (0.07 – 0.15) |
|  | MAD of ARHD HbDiffx (au) | 0.1 (0.07 – 0.16) | 0.12 (0.1 – 0.15) | 0.15 (0.12 – 0.18) | 0.08 (0.06 – 0.11) |
| Parietal | ARHD of COx-a (au) | 0.26 (0.12 – 0.42) | 0.23 (0.11 – 0.41) | 0.25 (0.11 – 0.47) | 0.2 (0.1 – 0.36) |
|  | ARHD of HbOx (au) | 0.23 (0.12 – 0.36) | 0.22 (0.1 – 0.4) | 0.22 (0.1 – 0.47) | 0.19 (0.1 – 0.32) |
|  | ARHD of HHbx (au) | 0.24 (0.13 – 0.45) | 0.26 (0.12 – 0.44) | 0.22 (0.1 – 0.47) | 0.25 (0.12 – 0.38) |
|  | ARHD of tHbx (au) | 0.24 (0.13 – 0.41) | 0.23 (0.1 – 0.42) | 0.23 (0.1 – 0.47) | 0.2 (0.08 – 0.32) |
|  | ARHD of HbDiffx (au) | 0.26 (0.12 – 0.4) | 0.23 (0.11 – 0.4) | 0.24 (0.11 – 0.45) | 0.2 (0.1 – 0.33) |
|  | MAD of ARHD COx-a (au) | 0.12 (0.09 – 0.15) | 0.14 (0.11 – 0.17) | 0.15 (0.12 – 0.18) | 0.1 (0.07 – 0.13) |
|  | MAD of ARHD HbOx (au) | 0.1 (0.07 – 0.16) | 0.13 (0.09 – 0.17) | 0.15 (0.11 – 0.19) | 0.09 (0.06 – 0.15) |
|  | MAD of ARHD HHbx (au) | 0.12 (0.08 – 0.16) | 0.14 (0.1 – 0.18) | 0.14 (0.12 – 0.19) | 0.11 (0.08 – 0.16) |
|  | MAD of ARHD tHbx (au) | 0.12 (0.09 – 0.15) | 0.13 (0.1 – 0.17) | 0.15 (0.12 – 0.2) | 0.1 (0.08 – 0.16) |
|  | MAD of ARHD HbDiffx (au) | 0.12 (0.09 – 0.15) | 0.12 (0.1 – 0.18) | 0.15 (0.12 – 0.19) | 0.1 (0.07 – 0.14) |
| Temporal | ARHD of COx-a (au) | 0.22 (0.12 – 0.38) | 0.23 (0.1 – 0.38) | 0.23 (0.1 – 0.42) | 0.17 (0.09 – 0.27) |
|  | ARHD of HbOx (au) | 0.21 (0.11 – 0.39) | 0.22 (0.09 – 0.39) | 0.25 (0.11 – 0.45) | 0.21 (0.11 – 0.36) |
|  | ARHD of HHbx (au) | 0.2 (0.11 – 0.35) | 0.24 (0.11 – 0.4) | 0.22 (0.1 – 0.42) | 0.19 (0.09 – 0.32) |
|  | ARHD of tHbx (au) | 0.22 (0.1 – 0.37) | 0.23 (0.11 – 0.43) | 0.24 (0.1 – 0.47) | 0.22 (0.11 – 0.36) |
|  | ARHD of HbDiffx (au) | 0.24 (0.13 – 0.37) | 0.22 (0.11 – 0.4) | 0.23 (0.11 – 0.42) | 0.18 (0.1 – 0.29) |
|  | MAD of ARHD COx-a (au) | 0.11 (0.08 – 0.15) | 0.12 (0.1 – 0.16) | 0.14 (0.11 – 0.17) | 0.09 (0.06 – 0.14) |
|  | MAD of ARHD HbOx (au) | 0.11 (0.07 – 0.16) | 0.13 (0.1 – 0.17) | 0.16 (0.12 – 0.2) | 0.11 (0.09 – 0.14) |
|  | MAD of ARHD HHbx (au) | 0.1 (0.07 – 0.13) | 0.13 (0.1 – 0.16) | 0.13 (0.11 – 0.18) | 0.1 (0.07 – 0.13) |
|  | MAD of ARHD tHbx (au) | 0.12 (0.08 – 0.16) | 0.15 (0.11 – 0.18) | 0.16 (0.12 – 0.19) | 0.1 (0.07 – 0.14) |
|  | MAD of ARHD HbDiffx (au) | 0.11 (0.08 – 0.16) | 0.12 (0.09 – 0.15) | 0.14 (0.12 – 0.18) | 0.09 (0.06 – 0.13) |
| Occipital | ARHD of COx-a (au) | 0.23 (0.12 – 0.34) | 0.23 (0.12 – 0.39) | 0.26 (0.12 – 0.47) | 0.2 (0.1 – 0.3) |
|  | ARHD of HbOx (au) | 0.22 (0.11 – 0.4) | 0.23 (0.12 – 0.38) | 0.24 (0.11 – 0.43) | 0.21 (0.1 – 0.33) |
|  | ARHD of HHbx (au) | 0.21 (0.11 – 0.35) | 0.25 (0.13 – 0.42) | 0.23 (0.1 – 0.42) | 0.19 (0.09 – 0.3) |
|  | ARHD of tHbx (au) | 0.23 (0.12 – 0.41) | 0.23 (0.1 – 0.4) | 0.23 (0.1 – 0.42) | 0.22 (0.12 – 0.34) |
|  | ARHD of HbDiffx (au) | 0.2 (0.12 – 0.36) | 0.22 (0.11 – 0.37) | 0.24 (0.12 – 0.44) | 0.21 (0.1 – 0.31) |
|  | MAD of ARHD COx-a (au) | 0.1 (0.07 – 0.13) | 0.12 (0.1 – 0.14) | 0.15 (0.12 – 0.17) | 0.09 (0.07 – 0.13) |
|  | MAD of ARHD HbOx (au) | 0.11 (0.08 – 0.15) | 0.12 (0.1 – 0.14) | 0.15 (0.11 – 0.18) | 0.11 (0.08 – 0.15) |
|  | MAD of ARHD HHbx (au) | 0.1 (0.07 – 0.15) | 0.12 (0.09 – 0.15) | 0.14 (0.11 – 0.17) | 0.09 (0.07 – 0.12) |
|  | MAD of ARHD tHbx (au) | 0.11 (0.09 – 0.15) | 0.13 (0.09 – 0.15) | 0.15 (0.12 – 0.19) | 0.11 (0.08 – 0.14) |
|  | MAD of ARHD HbDiffx (au) | 0.1 (0.07 – 0.13) | 0.12 (0.1 – 0.15) | 0.14 (0.12 – 0.17) | 0.09 (0.08 – 0.14) |
| **250 Hz Sampled Data** | | | | | |
| Frontal | ARHD of COx-a (au) | 0.21 (0.13 – 0.34) | 0.21 (0.1 – 0.36) | 0.25 (0.11 – 0.45) | 0.18 (0.1 – 0.29) |
|  | ARHD of HbOx (au) | 0.26 (0.15 – 0.37) | 0.23 (0.11 – 0.4) | 0.23 (0.1 – 0.41) | 0.19 (0.09 – 0.34) |
|  | ARHD of HHbx (au) | 0.25 (0.12 – 0.41) | 0.21 (0.09 – 0.37) | 0.23 (0.09 – 0.46) | 0.19 (0.09 – 0.3) |
|  | ARHD of tHbx (au) | 0.23 (0.14 – 0.42) | 0.26 (0.12 – 0.44) | 0.22 (0.1 – 0.43) | 0.2 (0.11 – 0.38) |
|  | ARHD of HbDiffx (au) | 0.21 (0.14 – 0.33) | 0.22 (0.1 – 0.37) | 0.24 (0.11 – 0.44) | 0.18 (0.09 – 0.3) |
|  | MAD of ARHD COx-a (au) | 0.09 (0.07 – 0.14) | 0.13 (0.1 – 0.15) | 0.15 (0.13 – 0.19) | 0.08 (0.07 – 0.11) |
|  | MAD of ARHD HbOx (au) | 0.11 (0.07 – 0.14) | 0.13 (0.09 – 0.17) | 0.14 (0.11 – 0.2) | 0.1 (0.07 – 0.14) |
|  | MAD of ARHD HHbx (au) | 0.1 (0.06 – 0.15) | 0.12 (0.1 – 0.15) | 0.16 (0.12 – 0.2) | 0.1 (0.06 – 0.14) |
|  | MAD of ARHD tHbx (au) | 0.11 (0.07 – 0.15) | 0.14 (0.11 – 0.17) | 0.14 (0.11 – 0.16) | 0.1 (0.08 – 0.15) |
|  | MAD of ARHD HbDiffx (au) | 0.1 (0.08 – 0.15) | 0.12 (0.1 – 0.16) | 0.15 (0.12 – 0.18) | 0.08 (0.07 – 0.12) |
| Parietal | ARHD of COx-a (au) | 0.24 (0.14 – 0.4) | 0.23 (0.1 – 0.41) | 0.24 (0.11 – 0.48) | 0.2 (0.1 – 0.32) |
|  | ARHD of HbOx (au) | 0.21 (0.1 – 0.35) | 0.22 (0.1 – 0.42) | 0.23 (0.1 – 0.46) | 0.2 (0.1 – 0.35) |
|  | ARHD of HHbx (au) | 0.24 (0.13 – 0.45) | 0.25 (0.12 – 0.45) | 0.23 (0.1 – 0.48) | 0.23 (0.11 – 0.37) |
|  | ARHD of tHbx (au) | 0.25 (0.13 – 0.42) | 0.23 (0.1 – 0.41) | 0.23 (0.11 – 0.47) | 0.2 (0.1 – 0.33) |
|  | ARHD of HbDiffx (au) | 0.26 (0.12 – 0.4) | 0.23 (0.11 – 0.41) | 0.24 (0.1 – 0.45) | 0.2 (0.1 – 0.31) |
|  | MAD of ARHD COx-a (au) | 0.11 (0.08 – 0.15) | 0.13 (0.11 – 0.16) | 0.15 (0.12 – 0.18) | 0.1 (0.06 – 0.14) |
|  | MAD of ARHD HbOx (au) | 0.09 (0.07 – 0.16) | 0.12 (0.09 – 0.17) | 0.15 (0.12 – 0.19) | 0.1 (0.07 – 0.16) |
|  | MAD of ARHD HHbx (au) | 0.11 (0.08 – 0.15) | 0.14 (0.1 – 0.17) | 0.15 (0.12 – 0.19) | 0.1 (0.08 – 0.15) |
|  | MAD of ARHD tHbx (au) | 0.13 (0.09 – 0.15) | 0.13 (0.1 – 0.17) | 0.15 (0.11 – 0.19) | 0.1 (0.07 – 0.15) |
|  | MAD of ARHD HbDiffx (au) | 0.12 (0.08 – 0.16) | 0.12 (0.11 – 0.16) | 0.15 (0.11 – 0.18) | 0.1 (0.07 – 0.14) |
| Temporal | ARHD of COx-a (au) | 0.24 (0.13 – 0.36) | 0.23 (0.1 – 0.38) | 0.24 (0.1 – 0.42) | 0.16 (0.08 – 0.27) |
|  | ARHD of HbOx (au) | 0.24 (0.12 – 0.38) | 0.22 (0.1 – 0.38) | 0.25 (0.12 – 0.48) | 0.22 (0.11 – 0.36) |
|  | ARHD of HHbx (au) | 0.22 (0.12 – 0.36) | 0.23 (0.11 – 0.39) | 0.23 (0.1 – 0.44) | 0.19 (0.1 – 0.3) |
|  | ARHD of tHbx (au) | 0.23 (0.09 – 0.38) | 0.23 (0.11 – 0.43) | 0.25 (0.11 – 0.49) | 0.22 (0.11 – 0.35) |
|  | ARHD of HbDiffx (au) | 0.25 (0.15 – 0.37) | 0.22 (0.11 – 0.39) | 0.24 (0.1 – 0.42) | 0.17 (0.08 – 0.28) |
|  | MAD of ARHD COx-a (au) | 0.1 (0.07 – 0.17) | 0.13 (0.1 – 0.17) | 0.13 (0.11 – 0.17) | 0.08 (0.07 – 0.13) |
|  | MAD of ARHD HbOx (au) | 0.12 (0.08 – 0.16) | 0.13 (0.1 – 0.17) | 0.16 (0.12 – 0.2) | 0.1 (0.07 – 0.14) |
|  | MAD of ARHD HHbx (au) | 0.1 (0.07 – 0.13) | 0.12 (0.1 – 0.17) | 0.14 (0.11 – 0.19) | 0.1 (0.07 – 0.14) |
|  | MAD of ARHD tHbx (au) | 0.11 (0.08 – 0.16) | 0.15 (0.11 – 0.17) | 0.15 (0.12 – 0.19) | 0.1 (0.07 – 0.14) |
|  | MAD of ARHD HbDiffx (au) | 0.12 (0.07 – 0.16) | 0.13 (0.1 – 0.15) | 0.14 (0.12 – 0.18) | 0.09 (0.07 – 0.13) |
| Occipital | ARHD of COx-a (au) | 0.23 (0.12 – 0.32) | 0.24 (0.12 – 0.4) | 0.26 (0.12 – 0.44) | 0.19 (0.1 – 0.31) |
|  | ARHD of HbOx (au) | 0.23 (0.1 – 0.39) | 0.24 (0.12 – 0.4) | 0.24 (0.11 – 0.42) | 0.22 (0.09 – 0.29) |
|  | ARHD of HHbx (au) | 0.2 (0.11 – 0.35) | 0.26 (0.13 – 0.41) | 0.24 (0.11 – 0.43) | 0.19 (0.1 – 0.3) |
|  | ARHD of tHbx (au) | 0.24 (0.11 – 0.39) | 0.22 (0.1 – 0.41) | 0.23 (0.1 – 0.42) | 0.21 (0.11 – 0.35) |
|  | ARHD of HbDiffx (au) | 0.23 (0.1 – 0.34) | 0.23 (0.12 – 0.39) | 0.25 (0.12 – 0.45) | 0.21 (0.09 – 0.32) |
|  | MAD of ARHD COx-a (au) | 0.1 (0.07 – 0.15) | 0.12 (0.1 – 0.16) | 0.14 (0.13 – 0.17) | 0.09 (0.07 – 0.12) |
|  | MAD of ARHD HbOx (au) | 0.11 (0.08 – 0.16) | 0.12 (0.1 – 0.15) | 0.14 (0.12 – 0.18) | 0.1 (0.08 – 0.15) |
|  | MAD of ARHD HHbx (au) | 0.1 (0.07 – 0.14) | 0.13 (0.1 – 0.16) | 0.14 (0.11 – 0.17) | 0.08 (0.07 – 0.1) |
|  | MAD of ARHD tHbx (au) | 0.12 (0.08 – 0.15) | 0.12 (0.1 – 0.15) | 0.15 (0.12 – 0.19) | 0.11 (0.08 – 0.13) |
|  | MAD of ARHD HbDiffx (au) | 0.1 (0.08 – 0.15) | 0.12 (0.1 – 0.15) | 0.14 (0.12 – 0.17) | 0.09 (0.07 – 0.12) |
| The table shows the perturbation subgrouped absolute regional hemispheric disparity analysis in on four brain lobes of the five NIRS-derived CVR indices using 10-second decimated data at 1 Hz and 250 Hz sampling frequencies. *ARHD, absolute regional hemispheric difference; au, arbitrary units; COx-a, cerebral oximetry index with arterial blood pressure; CVR, cerebrovascular reactivity index; HbDiffx, hemoglobin difference index; HbOx, oxyhemoglobin index; HHbx, deoxyhemoglobin index; IQR, interquartile range; MAD, median absolute deviation; tHbx, total hemoglobin index.* | | | | | |

Appendix S7g: Perturbation Subgrouped Regional Hemispheric Disparity Analysis on Physiologic Signals

| **Brain Lobe** | **Physiologic Variable** | **Median (IQR)** | | | | | | | |
| --- | --- | --- | --- | --- | --- | --- | --- | --- | --- |
|  |  | **Baseline** | | **Neurovascular Coupling** | | **Orthostatic Challenge** | | **Vascular Chemo-Reactivity** | |
|  |  | **Raw Data** | **10-Second Decimated Data** | **Raw Data** | **10-Second Decimated Data** | **Raw Data** | **10-Second Decimated Data** | **Raw Data** | **10-Second Decimated Data** |
| **1 Hz Sampled Data** | | | | | | | | | |
| Frontal | ARHD of rSO_2_ (au) | 6.37 (4.71 – 8.42) | 6.34 (4.79 – 8.55) | 7.33 (6.5 – 8.42) | 7.35 (6.51 – 8.39) | 7.18 (5.9 – 8.27) | 7.2 (5.9 – 8.23) | 7.49 (6.99 – 8) | 7.52 (7.07 – 7.98) |
|  | ARHD of HbO (au) | 28.01 (18.81 – 32.58) | 27.98 (19.34 – 32.23) | 25.87 (23.34 – 30.91) | 26.48 (23.33 – 30.09) | 24.44 (17.99 – 30.05) | 24.43 (17.51 – 29.43) | 23.72 (21.78 – 28.98) | 23.6 (21.87 – 26.86) |
|  | ARHD of HHb (au) | 29.29 (20.81 – 37.8) | 29.33 (20.67 – 36.75) | 25.87 (25.1 – 30.63) | 25.88 (25.07 – 30.66) | 29.45 (23.89 – 37.67) | 29.36 (24.08 – 37.5) | 33.73 (32.63 – 35.87) | 34.15 (32.72 – 35.8) |
|  | ARHD of tHb (au) | 40.87 (28.91 – 56.23) | 40.96 (30.9 – 54.08) | 36.14 (32.1 – 40.86) | 36.17 (32.32 – 39.86) | 40.03 (30.54 – 50.37) | 39.99 (31.27 – 50.1) | 40 (37.42 – 44.03) | 39.95 (37.69 – 44.07) |
|  | ARHD of HbDiff (au) | 30 (22.13 – 33.94) | 29.85 (20.31 – 33.93) | 26.07 (25.34 – 33.75) | 26.04 (25.39 – 30.45) | 27.39 (23.26 – 37.06) | 27.63 (23.26 – 35.71) | 33.44 (27.83 – 35.42) | 33.36 (27.8 – 34.58) |
|  | MAD of ARHD rSO_2_ (au) | 0.77 (0.41 – 1.63) | 0.69 (0.41 – 1.39) | 0.39 (0.22 – 0.76) | 0.36 (0.2 – 0.63) | 0.95 (0.73 – 1.51) | 0.93 (0.67 – 1.46) | 0.26 (0.14 – 0.53) | 0.25 (0.14 – 0.48) |
|  | MAD of ARHD HbO (au) | 4.12 (2.21 – 8.24) | 4.05 (2.06 – 7.11) | 2.1 (1.28 – 3.85) | 2.06 (1.2 – 3.63) | 5.32 (3.73 – 10.94) | 4.93 (3.62 – 10.1) | 1.27 (0.77 – 2.7) | 1.07 (0.64 – 2.4) |
|  | MAD of ARHD HHb (au) | 3.87 (1.68 – 10.28) | 3.72 (1.63 – 9.77) | 1.5 (0.68 – 3.37) | 1.49 (0.67 – 2.75) | 5.34 (2.81 – 10.04) | 4.17 (2.77 – 9.89) | 0.92 (0.34 – 2.91) | 0.82 (0.34 – 2.34) |
|  | MAD of ARHD tHb (au) | 7.34 (3.3 – 14.92) | 7.16 (3.05 – 15.13) | 2.77 (1.61 – 5) | 2.58 (1.7 – 3.95) | 8.32 (5.74 – 15.81) | 8.04 (5.36 – 15.89) | 1.54 (0.92 – 4.11) | 1.4 (0.77 – 3.51) |
|  | MAD of ARHD HbDiff (au) | 4.26 (2.16 – 7.6) | 3.85 (2.19 – 6.57) | 2.25 (1.02 – 4.28) | 2.24 (0.98 – 3.9) | 5.02 (2.88 – 7.42) | 4.96 (2.81 – 6.93) | 1.3 (0.74 – 3.14) | 1.18 (0.67 – 2.48) |
| Parietal | ARHD of rSO_2_ (au) | 5.3 (3.4 – 6.93) | 5.49 (3.59 – 6.9) | 4.27 (3.91 – 5.87) | 4.29 (3.88 – 5.89) | 4.86 (3.17 – 6.92) | 4.74 (3.17 – 6.76) | 5.27 (4.58 – 6.29) | 5.61 (4.75 – 5.99) |
|  | ARHD of HbO (au) | 42.25 (22.95 – 67.29) | 42.4 (24 – 66.77) | 47.01 (38.8 – 60.57) | 46.94 (38.52 – 60.5) | 43.64 (25.62 – 69) | 43.44 (26.14 – 68.54) | 41.88 (38.23 – 45.57) | 41.99 (41.46 – 44.87) |
|  | ARHD of HHb (au) | 34.66 (18.64 – 44.82) | 32.99 (19.36 – 44.17) | 29.88 (23.73 – 42.45) | 29.87 (23.81 – 42.7) | 27.52 (12.63 – 38.94) | 27.45 (13.29 – 38.73) | 25.45 (21.16 – 27.89) | 24.85 (20.97 – 27.43) |
|  | ARHD of tHb (au) | 70.83 (46.28 – 102.64) | 71.47 (47.02 – 104.06) | 89.02 (75.83 – 96.84) | 88.49 (74.77 – 95.69) | 69.7 (30.68 – 109.01) | 71.33 (30.21 – 108.78) | 61.03 (55.31 – 63.29) | 58.63 (56.92 – 62.85) |
|  | ARHD of HbDiff (au) | 33.58 (24.81 – 49.5) | 33.49 (21.91 – 44.82) | 27.9 (23.49 – 39.59) | 27.68 (22.71 – 39.49) | 31.4 (23.01 – 46.48) | 31.06 (23.98 – 44.43) | 32.88 (30.42 – 39.29) | 32.94 (31.34 – 33.85) |
|  | MAD of ARHD rSO_2_ (au) | 1.3 (0.74 – 2.14) | 1.11 (0.63 – 1.66) | 0.84 (0.47 – 1.29) | 0.48 (0.32 – 0.84) | 1.41 (1 – 2.2) | 1.31 (0.92 – 1.96) | 0.54 (0.28 – 1.07) | 0.3 (0.17 – 0.64) |
|  | MAD of ARHD HbO (au) | 9.65 (5.05 – 20.96) | 8.73 (3.56 – 15.1) | 4.91 (2.14 – 9.74) | 3.07 (1.62 – 6.37) | 13.65 (8.3 – 22.97) | 12.99 (8.19 – 24.07) | 3.11 (1.33 – 7.56) | 2.13 (0.81 – 5.27) |
|  | MAD of ARHD HHb (au) | 7.19 (3.96 – 13.16) | 6.1 (2.91 – 11.95) | 4.69 (2.22 – 7.43) | 3.64 (1.47 – 4.92) | 11.33 (6.45 – 17.97) | 10.7 (6.02 – 17.05) | 3.01 (1.35 – 5.98) | 2.04 (0.94 – 3.56) |
|  | MAD of ARHD tHb (au) | 13.31 (5.05 – 30.94) | 12.19 (4.65 – 28.33) | 5.62 (2.07 – 10.47) | 4.14 (1.62 – 8.75) | 23.39 (12.69 – 43.13) | 23.97 (12.27 – 42.29) | 3.14 (1.33 – 8.46) | 2.55 (0.84 – 7.35) |
|  | MAD of ARHD HbDiff (au) | 10.11 (5.91 – 16.81) | 8.68 (3.29 – 14.35) | 5.67 (3.23 – 12.63) | 3.92 (2.32 – 7.43) | 10.36 (6.97 – 21.67) | 8.56 (5.62 – 18.65) | 3.79 (1.55 – 9.63) | 2.35 (1.09 – 5.73) |
| Temporal | ARHD of rSO_2_ (au) | 7.56 (5.63 – 9.35) | 7.57 (5.82 – 9.49) | 7.87 (7.35 – 8.59) | 7.9 (7.41 – 8.53) | 8.14 (6.41 – 9.45) | 8.19 (6.53 – 9.27) | 8 (7.3 – 8.54) | 8.01 (7.74 – 8.3) |
|  | ARHD of HbO (au) | 53.71 (42.42 – 66.38) | 52.52 (43 – 64.82) | 47.67 (46.33 – 52.04) | 47.65 (46.38 – 51.43) | 56.49 (50.54 – 63.22) | 56.89 (52.25 – 63.13) | 61.76 (52.48 – 71.11) | 61.91 (53.01 – 71.29) |
|  | ARHD of HHb (au) | 35.31 (17.28 – 51.62) | 35.04 (17.86 – 52.28) | 35.25 (31.85 – 41.21) | 35.33 (32.22 – 41.17) | 31.79 (22.13 – 42.79) | 31.62 (22.1 – 43.33) | 27.84 (23.87 – 32.2) | 28.25 (23.46 – 30.07) |
|  | ARHD of tHb (au) | 75.42 (52.93 – 125.17) | 75.77 (53.84 – 120.61) | 79.8 (73.03 – 84.01) | 80.83 (73.31 – 83.39) | 101.9 (71.61 – 138.41) | 104.83 (80.54 – 137.22) | 105.57 (101.96 – 110.88) | 105.67 (102.07 – 109.38) |
|  | ARHD of HbDiff (au) | 47.75 (42.45 – 66.82) | 47.8 (43.2 – 66.95) | 50.2 (47.67 – 54.23) | 50.22 (47.72 – 52.31) | 54 (45.86 – 64.26) | 53.76 (45.68 – 62.85) | 50.36 (49.08 – 54.17) | 50.45 (49.07 – 52.26) |
|  | MAD of ARHD rSO_2_ (au) | 1.16 (0.57 – 1.59) | 0.68 (0.4 – 1.36) | 0.69 (0.33 – 1.05) | 0.42 (0.27 – 0.65) | 1.36 (0.92 – 1.74) | 1.14 (0.72 – 1.57) | 0.48 (0.2 – 1.01) | 0.32 (0.17 – 0.44) |
|  | MAD of ARHD HbO (au) | 9.03 (4.95 – 15.8) | 6.11 (3.84 – 12.94) | 4.21 (1.57 – 9.44) | 3.16 (1.51 – 5.15) | 11.8 (7.3 – 17.55) | 11.19 (5.79 – 14.21) | 3.86 (1.33 – 7.05) | 2.38 (1.22 – 4) |
|  | MAD of ARHD HHb (au) | 7.58 (4.38 – 11.57) | 5.96 (2.42 – 9.94) | 4.79 (1.68 – 7.12) | 2.7 (1.45 – 4.74) | 9.43 (6.14 – 16.04) | 8.09 (4.27 – 13.39) | 3.02 (0.86 – 6.57) | 2.12 (0.83 – 3.34) |
|  | MAD of ARHD tHb (au) | 10.78 (6.21 – 19.15) | 11.03 (4.11 – 18.19) | 5.29 (2.64 – 9.86) | 4.14 (2.13 – 8.79) | 17.18 (8.75 – 26.68) | 17.03 (8.22 – 26.1) | 4.66 (1.98 – 6.43) | 2.76 (1.57 – 5.43) |
|  | MAD of ARHD HbDiff (au) | 11.78 (3.34 – 17.55) | 6.78 (3.01 – 11.39) | 5.89 (1.97 – 14) | 3.47 (1.54 – 6.54) | 12.06 (5.61 – 17.46) | 8.65 (5.3 – 13.8) | 5.22 (0.96 – 10.51) | 2.64 (0.83 – 5.07) |
| Occipital | ARHD of rSO_2_ (au) | 11.57 (9.72 – 12.93) | 11.34 (9.82 – 12.9) | 12.16 (11.11 – 12.78) | 12.09 (11.48 – 12.43) | 11.04 (9.75 – 12.03) | 11.16 (10 – 11.71) | 11.55 (10.7 – 12.18) | 11.55 (11.25 – 11.82) |
|  | ARHD of HbO (au) | 317.28 (301.75 – 330.43) | 318.93 (303.11 – 329.18) | 318.98 (309.96 – 327.56) | 318.89 (315.08 – 323.03) | 312.12 (292.97 – 335.13) | 312.25 (297.94 – 331.08) | 317.34 (311.1 – 323.99) | 318.01 (314.37 – 320.08) |
|  | ARHD of HHb (au) | 113.79 (102.66 – 124.49) | 114.34 (102.78 – 123.54) | 114.66 (108.55 – 120.61) | 115.02 (112.46 – 117.09) | 106.41 (87.55 – 121.16) | 106.31 (90.19 – 119.76) | 112.5 (105.25 – 120.38) | 112.63 (109.9 – 115.62) |
|  | ARHD of tHb (au) | 436.29 (402.76 – 454.05) | 438.31 (405.01 – 451.73) | 432.96 (425.42 – 440.64) | 430.88 (426.14 – 439.04) | 433.05 (388.86 – 459.35) | 432.61 (391.17 – 460.38) | 443.7 (438.94 – 448.02) | 443.58 (441.79 – 445.19) |
|  | ARHD of HbDiff (au) | 201.63 (181.26 – 217.66) | 204.34 (188.14 – 216.04) | 206.4 (191.32 – 218.01) | 204.47 (195.89 – 213.68) | 199.23 (183.57 – 215.31) | 199.49 (189.29 – 208.51) | 199.64 (185.84 – 216.86) | 199.57 (194.55 – 207.32) |
|  | MAD of ARHD rSO_2_ (au) | 0.97 (0.82 – 1.3) | 0.61 (0.42 – 0.8) | 0.88 (0.68 – 1.12) | 0.4 (0.28 – 0.59) | 1.16 (0.98 – 1.82) | 0.79 (0.62 – 1.55) | 0.92 (0.68 – 1.16) | 0.36 (0.29 – 0.47) |
|  | MAD of ARHD HbO (au) | 11.97 (9.29 – 16.12) | 7.21 (4.63 – 13.21) | 8.12 (6.71 – 9.83) | 3.61 (2.67 – 6.32) | 16.75 (13.44 – 24.57) | 13.71 (8.17 – 22.81) | 9.74 (6.21 – 10.91) | 3.69 (2.72 – 4.84) |
|  | MAD of ARHD HHb (au) | 7.77 (6.13 – 10.42) | 4.01 (2.93 – 7.34) | 6.17 (5.52 – 6.8) | 2.69 (2.26 – 4.59) | 12.09 (8.8 – 15.75) | 9.92 (5.41 – 14.6) | 6.98 (5.68 – 7.93) | 2.67 (2.16 – 3.84) |
|  | MAD of ARHD tHb (au) | 11.14 (7.5 – 18.78) | 8.24 (4.47 – 17.89) | 5.97 (4.67 – 10.12) | 3.78 (2.07 – 9.48) | 21.79 (13.88 – 33.36) | 21.66 (11.85 – 33.19) | 6.05 (4.53 – 8.48) | 2.8 (1.88 – 6.48) |
|  | MAD of ARHD HbDiff (au) | 14.57 (12.62 – 17.02) | 6.47 (5.2 – 10.81) | 13.04 (10.09 – 14.69) | 5.28 (3.84 – 6.9) | 16.8 (14.15 – 20.73) | 9.02 (7.18 – 13.43) | 15.73 (10.49 – 17.19) | 5.48 (3.86 – 6.79) |
| **250 Hz Sampled Data** | | | | | | | | | |
| Frontal | ARHD of rSO_2_ (au) | 7.25 (5.22 – 9.22) | 6.41 (4.73 – 8.34) | 7.62 (5.75 – 8.78) | 7.38 (6.5 – 8.3) | 7.5 (5.67 – 8.86) | 7.19 (5.91 – 8.23) | 7.78 (6.8 – 8.31) | 7.51 (7.02 – 7.98) |
|  | ARHD of HbO (au) | 28.9 (19.88 – 35.49) | 27.93 (18.6 – 32.55) | 27.76 (23.29 – 31.8) | 26.49 (23.32 – 30.1) | 24.9 (18.58 – 30.57) | 24.42 (17.65 – 29.4) | 25.51 (21.73 – 28.98) | 23.61 (21.88 – 26.86) |
|  | ARHD of HHb (au) | 30.11 (23.2 – 38.42) | 29.18 (20.76 – 36.77) | 28.1 (26.31 – 31.78) | 25.9 (25.08 – 30.65) | 29.4 (23.79 – 38.36) | 29.37 (24.04 – 37.54) | 35.04 (31.75 – 36.6) | 34.22 (32.72 – 35.79) |
|  | ARHD of tHb (au) | 41.71 (29.46 – 59.55) | 41.11 (30.85 – 53.47) | 37.35 (30.93 – 40.98) | 36.2 (32.2 – 39.87) | 40.67 (30.54 – 51.44) | 40.04 (31.24 – 48.63) | 43.06 (37.46 – 45.06) | 39.92 (37.65 – 44.05) |
|  | ARHD of HbDiff (au) | 31.74 (24.43 – 41.2) | 28.91 (19.99 – 34.04) | 29.48 (25.13 – 36.43) | 26.06 (25.37 – 30.45) | 29.13 (23.65 – 41.1) | 27.64 (23.3 – 35.7) | 34.5 (27.83 – 36.63) | 33.39 (27.78 – 34.58) |
|  | MAD of ARHD rSO_2_ (au) | 0.83 (0.5 – 1.82) | 0.67 (0.37 – 1.38) | 0.5 (0.3 – 0.84) | 0.35 (0.21 – 0.62) | 1.02 (0.76 – 1.62) | 0.93 (0.69 – 1.46) | 0.39 (0.24 – 0.8) | 0.23 (0.13 – 0.46) |
|  | MAD of ARHD HbO (au) | 5.3 (2.47 – 9.62) | 4.04 (2.14 – 7.35) | 2.35 (1.37 – 4.97) | 2.06 (1.19 – 3.65) | 5.59 (3.68 – 12.11) | 4.83 (3.59 – 10.1) | 1.52 (0.84 – 3.24) | 1.15 (0.63 – 2.38) |
|  | MAD of ARHD HHb (au) | 4.12 (1.72 – 10.96) | 3.64 (1.57 – 9.79) | 1.67 (0.72 – 3.88) | 1.51 (0.67 – 2.62) | 5.48 (2.86 – 12.32) | 4.26 (2.77 – 9.87) | 1.47 (0.5 – 3.88) | 0.81 (0.34 – 2.44) |
|  | MAD of ARHD tHb (au) | 7.79 (3.34 – 18.13) | 7.3 (2.98 – 14.64) | 3.04 (1.68 – 6.24) | 2.64 (1.7 – 3.98) | 10.14 (5.74 – 18.43) | 8.17 (5.41 – 15.94) | 1.85 (1.05 – 4.61) | 1.3 (0.76 – 3.08) |
|  | MAD of ARHD HbDiff (au) | 4.49 (2.38 – 9.02) | 3.88 (2.08 – 6.66) | 2.46 (1.18 – 5.07) | 2.22 (0.97 – 3.74) | 4.99 (3.12 – 7.35) | 5.01 (2.77 – 6.89) | 1.99 (1.04 – 5) | 1.18 (0.71 – 2.5) |
| Parietal | ARHD of rSO_2_ (au) | 7.1 (3.94 – 10.24) | 5.43 (3.48 – 6.93) | 7.18 (3.73 – 11.78) | 4.28 (3.9 – 5.9) | 6.78 (3.42 – 10.49) | 4.79 (3.03 – 6.74) | 6.9 (4.22 – 9.98) | 5.4 (4.7 – 5.83) |
|  | ARHD of HbO (au) | 49.19 (30.89 – 80.18) | 42.89 (22.85 – 66.75) | 62.38 (38.82 – 85.4) | 46.86 (38.49 – 61.17) | 53.34 (28.34 – 83.34) | 43.04 (26.15 – 68.5) | 48.59 (35.29 – 52) | 41.92 (41.44 – 44.81) |
|  | ARHD of HHb (au) | 46.13 (21.16 – 68.97) | 32.72 (19.52 – 43.98) | 50.35 (28.21 – 70.29) | 29.69 (23.84 – 42.59) | 40.84 (22.43 – 67.6) | 27.2 (13.09 – 38.95) | 34.84 (22.79 – 43.09) | 25.31 (21.07 – 27.62) |
|  | ARHD of tHb (au) | 68.85 (46.57 – 107.56) | 71.48 (47.15 – 104.2) | 88.35 (70.47 – 100.07) | 88.5 (75 – 95.6) | 70.47 (31.27 – 109) | 70.77 (30.07 – 108.72) | 60.4 (49.54 – 71.59) | 59.82 (56.71 – 62.61) |
|  | ARHD of HbDiff (au) | 45.39 (28.12 – 80.82) | 33.48 (23.4 – 44.47) | 51.67 (30.14 – 87.57) | 27.06 (22.72 – 39.52) | 45.46 (23.47 – 67.17) | 31.25 (23.92 – 44.81) | 53.82 (29.52 – 72.91) | 32.94 (31.12 – 33.88) |
|  | MAD of ARHD rSO_2_ (au) | 3.28 (1.2 – 4.67) | 1.08 (0.64 – 1.77) | 3.18 (1.17 – 5.04) | 0.48 (0.32 – 0.82) | 3.09 (1.52 – 4.64) | 1.35 (0.95 – 1.99) | 1.66 (0.62 – 4.44) | 0.29 (0.16 – 0.63) |
|  | MAD of ARHD HbO (au) | 20.43 (8.21 – 34.32) | 8.85 (3.44 – 15.78) | 16.3 (4.93 – 37.8) | 2.99 (1.64 – 6.11) | 22.91 (11.07 – 38.73) | 13.45 (8.2 – 22.61) | 7.11 (3.62 – 28.59) | 2.19 (0.86 – 5.57) |
|  | MAD of ARHD HHb (au) | 17.52 (6.64 – 31.57) | 6.09 (2.98 – 11.74) | 12.44 (5.52 – 31.8) | 3.57 (1.43 – 5.16) | 17.57 (8.63 – 33.55) | 10.49 (6.27 – 17.19) | 7.31 (3.45 – 23.47) | 2.03 (0.96 – 3.67) |
|  | MAD of ARHD tHb (au) | 20.18 (9.34 – 44.3) | 11.43 (4.46 – 29.56) | 13.58 (3.71 – 26.83) | 3.99 (1.64 – 8.53) | 27.85 (17.14 – 45.1) | 23.86 (12.39 – 42.22) | 6.6 (3.2 – 20.35) | 2.36 (0.89 – 6.28) |
|  | MAD of ARHD HbDiff (au) | 24.44 (9.04 – 52.96) | 8.74 (3.44 – 14.94) | 22.05 (6.1 – 62.31) | 3.95 (2.23 – 7) | 19.54 (9.53 – 50.42) | 8.67 (5.81 – 19.81) | 12.06 (3.79 – 42.32) | 2.19 (1.11 – 5.76) |
| Temporal | ARHD of rSO_2_ (au) | 9.5 (5.47 – 12.44) | 7.56 (5.68 – 9.43) | 9.58 (5.12 – 12.56) | 7.77 (7.36 – 8.34) | 8.73 (5.04 – 12.48) | 8.07 (6.39 – 9.01) | 9.09 (6.04 – 11.21) | 7.93 (7.61 – 8.24) |
|  | ARHD of HbO (au) | 84.8 (42.56 – 104.75) | 52.32 (42.88 – 65.57) | 77.79 (46.2 – 84.23) | 47.7 (46.38 – 51.45) | 78.25 (48 – 107.61) | 56.79 (52.31 – 63.46) | 84.28 (52.71 – 98.68) | 61.96 (52.96 – 71.22) |
|  | ARHD of HHb (au) | 52.53 (28.86 – 77.2) | 35.21 (17.86 – 52.3) | 53.59 (29.14 – 73.55) | 35.31 (32.23 – 40.95) | 55.34 (27.81 – 84.75) | 31.64 (22.1 – 42.83) | 44.11 (28.37 – 57.47) | 28.18 (23.63 – 30.08) |
|  | ARHD of tHb (au) | 86.36 (49.1 – 124.89) | 75.09 (48.63 – 125.22) | 79.47 (66.4 – 99.75) | 80.85 (73.19 – 83.37) | 103.02 (69.09 – 138.09) | 104.69 (80.45 – 136.89) | 110.21 (99.22 – 116.22) | 105.72 (102.31 – 109.49) |
|  | ARHD of HbDiff (au) | 68.69 (47.63 – 126.59) | 49.73 (43.49 – 66.8) | 70.59 (52.1 – 94.74) | 50.21 (47.72 – 52.45) | 64 (45.35 – 87.23) | 53.8 (45.88 – 62.98) | 66.5 (47.16 – 87.96) | 50.42 (49.05 – 52.27) |
|  | MAD of ARHD rSO_2_ (au) | 3.59 (0.93 – 5.54) | 0.71 (0.42 – 1.29) | 4.21 (0.48 – 5.17) | 0.43 (0.26 – 0.68) | 3.53 (1.26 – 5.07) | 1.14 (0.73 – 1.54) | 2.02 (0.29 – 4.81) | 0.32 (0.18 – 0.45) |
|  | MAD of ARHD HbO (au) | 23.8 (6.48 – 53.72) | 5.93 (4.14 – 13.52) | 16.04 (2.43 – 52.26) | 3.22 (1.52 – 5.09) | 25.32 (10.49 – 52.98) | 11.03 (5.73 – 14.22) | 10.54 (1.77 – 44.72) | 2.46 (1.12 – 4.12) |
|  | MAD of ARHD HHb (au) | 26.09 (6.89 – 37.62) | 5.29 (2.6 – 9.79) | 16.09 (2.21 – 37.58) | 2.77 (1.46 – 4.82) | 28.14 (8.12 – 38.52) | 7.77 (4.32 – 13.53) | 10.17 (1.58 – 34.26) | 1.98 (0.85 – 3.64) |
|  | MAD of ARHD tHb (au) | 24.14 (12.41 – 36.2) | 11.02 (4.21 – 21.28) | 13.29 (4.26 – 32.32) | 4.02 (2.13 – 8.72) | 28.29 (17.02 – 42.83) | 17.12 (8.35 – 26.27) | 9.72 (2.33 – 26.45) | 2.97 (1.59 – 5.25) |
|  | MAD of ARHD HbDiff (au) | 27.61 (4.81 – 75.35) | 6.79 (3.09 – 11.37) | 25.24 (2.96 – 74.38) | 3.19 (1.54 – 6.64) | 26.16 (5.85 – 65.27) | 8.53 (5.33 – 13.36) | 17.8 (1.65 – 55.74) | 2.83 (0.83 – 4.93) |
| Occipital | ARHD of rSO_2_ (au) | 11.63 (6.68 – 18.21) | 11.35 (9.55 – 12.69) | 11.3 (7.34 – 18.07) | 11.84 (11.31 – 12.29) | 10.9 (6.69 – 17.23) | 10.99 (9.89 – 11.51) | 11.11 (6.93 – 17.14) | 11.4 (11.12 – 11.69) |
|  | ARHD of HbO (au) | 333.95 (250.6 – 372.25) | 319.48 (302.35 – 329.69) | 340.23 (254.3 – 375.44) | 318.39 (315.2 – 323.04) | 328.16 (248.98 – 370.78) | 312.52 (297.19 – 331.21) | 335.4 (257.77 – 370.4) | 317.6 (314.9 – 319.93) |
|  | ARHD of HHb (au) | 123.89 (67.39 – 153.67) | 114.11 (104.27 – 123.61) | 134.64 (68.02 – 154.69) | 114.8 (112.46 – 117.14) | 116.95 (59.31 – 143.91) | 106.23 (90.37 – 119.65) | 129.66 (64.53 – 153.58) | 112.27 (110.04 – 115.33) |
|  | ARHD of tHb (au) | 435.89 (382.23 – 474.6) | 438.69 (404.48 – 451.53) | 446.33 (390.79 – 477.91) | 430.92 (425.96 – 439.48) | 433.45 (370.79 – 470.53) | 433.3 (390.92 – 460.32) | 453.77 (407.95 – 479.23) | 443.44 (441.53 – 445.42) |
|  | ARHD of HbDiff (au) | 209.11 (114.19 – 291.86) | 201.28 (187.37 – 215.24) | 213.75 (117.38 – 291.92) | 204.27 (196.91 – 213.49) | 205.53 (113.91 – 287.67) | 199.29 (189.71 – 207.99) | 213.06 (110.99 – 289.75) | 199.94 (194.63 – 207.16) |
|  | MAD of ARHD rSO_2_ (au) | 5.06 (4.36 – 6.26) | 0.62 (0.42 – 0.83) | 5.13 (4.33 – 6.29) | 0.41 (0.27 – 0.58) | 5.02 (4.23 – 6.01) | 0.75 (0.62 – 1.51) | 4.93 (4.18 – 5.92) | 0.37 (0.29 – 0.48) |
|  | MAD of ARHD HbO (au) | 56.24 (46.61 – 62.32) | 6.55 (4.39 – 13.36) | 55.97 (44.92 – 59.51) | 3.73 (2.84 – 6.59) | 56.26 (48.7 – 63.71) | 13.67 (8.19 – 23.19) | 56.02 (44.97 – 60.41) | 3.44 (2.54 – 4.72) |
|  | MAD of ARHD HHb (au) | 37.84 (33.27 – 42.31) | 4 (2.78 – 6.87) | 36.73 (31.79 – 40.85) | 2.76 (2.17 – 4.71) | 38.09 (34.48 – 43.46) | 10.01 (5.31 – 14.05) | 36.96 (30.52 – 41.2) | 2.71 (2.23 – 3.78) |
|  | MAD of ARHD tHb (au) | 39.59 (30.51 – 46.87) | 8.01 (4.98 – 17.96) | 33.32 (27.64 – 38.06) | 3.81 (2.16 – 9.06) | 43.5 (35.69 – 50.6) | 21.23 (12.07 – 33.29) | 33.5 (28.41 – 37.82) | 3.08 (1.84 – 6.17) |
|  | MAD of ARHD HbDiff (au) | 86.68 (74.63 – 91.92) | 6.29 (5.19 – 9.26) | 85.34 (72.24 – 92.08) | 4.93 (3.91 – 6.75) | 84.43 (72.43 – 91.59) | 9.19 (7.03 – 13.41) | 84.56 (72.56 – 91.55) | 5.34 (3.79 – 6.4) |
| The table shows the perturbation subgrouped absolute regional hemispheric disparity analysis in on four brain lobes of physiologic signals using raw and 10-second decimated data at 1 Hz and 250 Hz sampling frequencies. *ARHD, absolute regional hemispheric difference; au, arbitrary units; HbDiff, hemoglobin difference; HbO, oxyhemoglobin; HHb, deoxyhemoglobin; IQR, interquartile range; MAD, median absolute deviation; mmHg, millimeters of mercury; rSO_2_, regional cerebral oxygen saturation; tHb, total hemoglobin.* | | | | | | | | | |

Appendix S7h: Perturbation Subgrouped Optimal ARIMA Models Based on AIC of Physiologic Signals and their Hemispheric Disparity

| **Physiologic Signal** | **Brain Lobe** | **Hemisphere** | **Optimal ARIMA Models (Median [IQR]) for Perturbation Subgroups** | | | |
| --- | --- | --- | --- | --- | --- | --- |
|  |  |  | **Baseline** | **Neurovascular Coupling** | **Orthostatic Challenge** | **Vascular Chemo-Reactivity** |
| **1 Hz Sampled Data** | | | | | | |
| ABP | – | – | (1,1,3) [(1,1,1) – (2,1,5)] | (3,1,9) [(2,1,5) – (5,1,5)] | (4,1,3) [(2,1,3) – (5,1,10)] | (2,1,2) [(1,1,1) – (2,1,7)] |
| rSO_2_ | Frontal | Left | (1,1,7) [(1,1,1) – (2,1,3)] | (2,1,0) [(1,1,2) – (3,1,0)] | (2,1,3) [(1,1,3) – (4,1,1)] | (1,1,3) [(1,1,1) – (2,1,2)] |
|  |  | Right | (1,1,2) [(1,1,0) – (2,1,3)] | (2,1,1) [(1,1,7) – (3,1,2)] | (3,1,3) [(1,1,2) – (4,1,2)] | (1,1,2) [(1,1,1) – (2,1,2)] |
|  | Parietal | Left | (1,1,2) [(1,1,1) – (2,1,2)] | (2,1,2) [(1,1,1) – (3,1,3)] | (2,1,1) [(1,1,2) – (4,1,2)] | (1,1,5) [(1,1,1) – (2,1,2)] |
|  |  | Right | (1,1,3) [(1,1,1) – (2,1,2)] | (2,1,1) [(1,1,1) – (3,1,0)] | (2,1,3) [(1,1,3) – (4,1,3)] | (1,1,4) [(1,1,1) – (2,1,2)] |
|  | Temporal | Left | (1,1,3) [(1,1,1) – (2,1,2)] | (2,1,1) [(1,1,1) – (2,1,6)] | (2,1,4) [(1,1,5) – (4,1,4)] | (1,1,1) [(1,1,1) – (2,1,1)] |
|  |  | Right | (1,1,1) [(1,1,0) – (2,1,2)] | (2,1,2) [(1,1,1) – (3,1,1)] | (2,1,4) [(1,1,5) – (4,1,3)] | (1,1,4) [(1,1,1) – (2,1,4)] |
|  | Occipital | Left | (1,1,1) [(1,1,1) – (2,1,4)] | (1,1,2) [(1,1,1) – (2,1,3)] | (2,1,3) [(1,1,2) – (4,1,10)] | (1,1,5) [(1,1,1) – (2,1,6)] |
|  |  | Right | (1,1,3) [(1,1,1) – (2,1,1)] | (1,1,10) [(1,1,1) – (2,1,8)] | (2,1,2) [(1,1,2) – (3,1,9)] | (1,1,1) [(1,1,1) – (2,1,3)] |
| HbO | Frontal | Left | (1,1,5) [(1,1,1) – (2,1,2)] | (2,1,4) [(1,1,8) – (3,1,3)] | (2,1,4) [(1,1,9) – (4,1,5)] | (2,1,1) [(1,1,1) – (2,1,6)] |
|  |  | Right | (1,1,5) [(1,1,1) – (2,1,3)] | (2,1,0) [(1,1,1) – (3,1,4)] | (3,1,2) [(1,1,2) – (5,1,3)] | (1,1,4) [(1,1,1) – (2,1,9)] |
|  | Parietal | Left | (1,1,3) [(1,1,1) – (2,1,1)] | (2,1,2) [(1,1,1) – (3,1,3)] | (2,1,3) [(2,1,0) – (4,1,3)] | (1,1,3) [(1,1,1) – (2,1,5)] |
|  |  | Right | (1,1,2) [(1,1,1) – (2,1,1)] | (2,1,3) [(1,1,2) – (3,1,4)] | (2,1,1) [(1,1,2) – (6,1,1)] | (1,1,8) [(1,1,1) – (2,1,3)] |
|  | Temporal | Left | (1,1,2) [(1,1,0) – (2,1,1)] | (2,1,2) [(1,1,2) – (3,1,4)] | (2,1,10) [(1,1,7) – (5,1,1)] | (1,1,3) [(1,1,1) – (2,1,4)] |
|  |  | Right | (2,1,0) [(1,1,1) – (3,1,0)] | (2,1,1) [(1,1,2) – (3,1,2)] | (3,1,1) [(1,1,4) – (4,1,3)] | (1,1,3) [(1,1,1) – (3,1,0)] |
|  | Occipital | Left | (1,1,2) [(1,1,1) – (2,1,3)] | (2,1,1) [(1,1,1) – (2,1,5)] | (2,1,4) [(1,1,6) – (4,1,7)] | (1,1,3) [(1,1,1) – (2,1,3)] |
|  |  | Right | (1,1,2) [(1,1,1) – (2,1,1)] | (2,1,0) [(1,1,1) – (3,1,0)] | (3,1,3) [(1,1,9) – (5,1,8)] | (1,1,3) [(1,1,1) – (2,1,1)] |
| HHb | Frontal | Left | (1,1,2) [(1,1,1) – (2,1,1)] | (2,1,2) [(1,1,2) – (3,1,3)] | (2,1,3) [(1,1,2) – (4,1,3)] | (1,1,3) [(1,1,1) – (2,1,5)] |
|  |  | Right | (1,1,5) [(1,1,0) – (3,1,0)] | (1,1,5) [(1,1,1) – (3,1,3)] | (2,1,2) [(1,1,3) – (4,1,4)] | (1,1,2) [(1,1,1) – (2,1,2)] |
|  | Parietal | Left | (1,1,3) [(1,1,1) – (2,1,10)] | (2,1,1) [(1,1,1) – (3,1,2)] | (2,1,7) [(2,1,0) – (3,1,5)] | (1,1,1) [(1,1,1) – (2,1,2)] |
|  |  | Right | (1,1,1) [(1,1,0) – (2,1,2)] | (2,1,1) [(1,1,1) – (2,1,4)] | (3,1,1) [(1,1,10) – (4,1,7)] | (2,1,0) [(1,1,1) – (2,1,6)] |
|  | Temporal | Left | (1,1,2) [(1,1,1) – (2,1,3)] | (2,1,1) [(1,1,2) – (3,1,5)] | (2,1,4) [(1,1,2) – (3,1,5)] | (1,1,5) [(1,1,1) – (2,1,3)] |
|  |  | Right | (1,1,2) [(1,1,1) – (2,1,2)] | (2,1,0) [(1,1,1) – (2,1,8)] | (3,1,3) [(2,1,0) – (6,1,6)] | (1,1,4) [(1,1,1) – (2,1,4)] |
|  | Occipital | Left | (1,1,1) [(1,1,1) – (2,1,5)] | (2,1,1) [(1,1,1) – (2,1,8)] | (2,1,8) [(2,1,1) – (4,1,6)] | (2,1,1) [(1,1,2) – (3,1,3)] |
|  |  | Right | (1,1,7) [(1,1,1) – (2,1,5)] | (1,1,7) [(1,1,1) – (3,1,1)] | (3,1,3) [(1,1,4) – (4,1,3)] | (1,1,10) [(1,1,1) – (2,1,6)] |
| tHb | Frontal | Left | (1,1,2) [(1,1,1) – (2,1,2)] | (2,1,1) [(1,1,2) – (3,1,2)] | (4,1,2) [(1,1,3) – (6,1,6)] | (1,1,9) [(1,1,1) – (2,1,4)] |
|  |  | Right | (1,1,6) [(1,1,0) – (4,1,0)] | (2,1,1) [(1,1,2) – (2,1,8)] | (3,1,2) [(1,1,5) – (5,1,7)] | (1,1,7) [(1,1,1) – (3,1,3)] |
|  | Parietal | Left | (1,1,2) [(1,1,1) – (2,1,1)] | (2,1,0) [(1,1,1) – (3,1,3)] | (2,1,5) [(1,1,4) – (3,1,10)] | (1,1,1) [(1,1,1) – (2,1,2)] |
|  |  | Right | (2,1,0) [(1,1,1) – (2,1,6)] | (1,1,2) [(1,1,1) – (2,1,6)] | (3,1,4) [(1,1,10) – (5,1,4)] | (1,1,2) [(1,1,1) – (2,1,2)] |
|  | Temporal | Left | (1,1,1) [(1,1,1) – (2,1,3)] | (2,1,0) [(1,1,1) – (3,1,0)] | (3,1,1) [(1,1,8) – (5,1,5)] | (1,1,5) [(1,1,1) – (2,1,4)] |
|  |  | Right | (1,1,6) [(1,1,1) – (3,1,0)] | (2,1,0) [(1,1,1) – (3,1,0)] | (2,1,3) [(1,1,2) – (4,1,4)] | (1,1,7) [(1,1,1) – (2,1,4)] |
|  | Occipital | Left | (1,1,1) [(1,1,1) – (2,1,2)] | (2,1,3) [(1,1,1) – (2,1,8)] | (3,1,3) [(2,1,1) – (4,1,6)] | (1,1,2) [(1,1,1) – (2,1,3)] |
|  |  | Right | (2,1,0) [(1,1,1) – (2,1,3)] | (2,1,2) [(1,1,1) – (3,1,0)] | (3,1,2) [(1,1,7) – (4,1,10)] | (1,1,3) [(1,1,1) – (2,1,4)] |
| HbDiff | Frontal | Left | (2,1,0) [(1,1,1) – (2,1,8)] | (2,1,3) [(1,1,2) – (4,1,3)] | (3,1,3) [(1,1,3) – (5,1,7)] | (2,1,1) [(1,1,1) – (3,1,3)] |
|  |  | Right | (1,1,6) [(1,1,1) – (2,1,3)] | (2,1,1) [(1,1,2) – (3,1,4)] | (3,1,6) [(2,1,2) – (6,1,6)] | (2,1,0) [(1,1,1) – (2,1,5)] |
|  | Parietal | Left | (1,1,4) [(1,1,1) – (2,1,5)] | (2,1,2) [(1,1,1) – (3,1,3)] | (2,1,5) [(2,1,0) – (5,1,4)] | (1,1,10) [(1,1,1) – (2,1,6)] |
|  |  | Right | (1,1,10) [(1,1,1) – (2,1,3)] | (2,1,4) [(2,1,1) – (4,1,3)] | (2,1,10) [(1,1,9) – (4,1,4)] | (1,1,4) [(1,1,1) – (2,1,3)] |
|  | Temporal | Left | (1,1,3) [(1,1,1) – (2,1,3)] | (2,1,0) [(1,1,1) – (2,1,4)] | (3,1,2) [(2,1,1) – (5,1,1)] | (1,1,5) [(1,1,1) – (2,1,4)] |
|  |  | Right | (1,1,1) [(1,1,0) – (2,1,3)] | (2,1,3) [(1,1,2) – (3,1,4)] | (2,1,7) [(1,1,8) – (4,1,4)] | (1,1,5) [(1,1,1) – (2,1,6)] |
|  | Occipital | Left | (1,1,3) [(1,1,1) – (3,1,0)] | (2,1,1) [(1,1,1) – (3,1,1)] | (3,1,4) [(2,1,1) – (5,1,5)] | (1,1,4) [(1,1,1) – (2,1,4)] |
|  |  | Right | (1,1,7) [(1,1,1) – (2,1,3)] | (2,1,3) [(1,1,1) – (3,1,3)] | (2,1,3) [(1,1,3) – (4,1,6)] | (1,1,4) [(1,1,1) – (3,1,2)] |
| COx-a | Frontal | Left | (1,1,1) [(1,1,0) – (3,1,0)] | (1,1,7) [(1,1,0) – (2,1,4)] | (2,1,1) [(1,1,3) – (3,1,4)] | (1,1,0) [(1,1,0) – (1,1,3)] |
|  |  | Right | (1,1,2) [(1,1,0) – (3,1,0)] | (1,1,3) [(1,1,0) – (2,1,2)] | (2,1,2) [(1,1,3) – (3,1,9)] | (1,1,3) [(1,1,0) – (2,1,2)] |
|  | Parietal | Left | (1,1,1) [(1,1,0) – (2,1,1)] | (2,1,0) [(1,1,1) – (3,1,1)] | (2,1,2) [(1,1,1) – (4,1,1)] | (1,1,1) [(1,1,0) – (2,1,1)] |
|  |  | Right | (1,1,1) [(1,1,0) – (2,1,2)] | (2,1,0) [(1,1,0) – (2,1,3)] | (2,1,0) [(1,1,1) – (3,1,4)] | (1,1,1) [(1,1,0) – (2,1,2)] |
|  | Temporal | Left | (1,1,1) [(1,1,0) – (2,1,1)] | (1,1,7) [(1,1,0) – (2,1,6)] | (1,1,5) [(1,1,1) – (3,1,4)] | (1,1,3) [(1,1,0) – (2,1,2)] |
|  |  | Right | (1,1,2) [(1,1,0) – (2,1,2)] | (2,1,1) [(1,1,0) – (4,1,4)] | (1,1,10) [(1,1,1) – (4,1,2)] | (1,1,2) [(1,1,0) – (2,1,2)] |
|  | Occipital | Left | (1,1,1) [(1,1,0) – (1,1,5)] | (1,1,4) [(1,1,0) – (2,1,2)] | (2,1,0) [(1,1,0) – (3,1,2)] | (1,1,1) [(1,1,0) – (2,1,0)] |
|  |  | Right | (1,1,1) [(1,1,0) – (2,1,1)] | (1,1,5) [(1,1,0) – (3,1,0)] | (2,1,1) [(1,1,0) – (3,1,2)] | (1,1,1) [(1,1,0) – (2,1,2)] |
| HbOx | Frontal | Left | (1,1,1) [(1,1,0) – (2,1,3)] | (2,1,1) [(1,1,0) – (2,1,4)] | (2,1,2) [(1,1,1) – (4,1,0)] | (1,1,0) [(1,1,0) – (2,1,1)] |
|  |  | Right | (1,1,1) [(1,1,0) – (2,1,2)] | (2,1,0) [(1,1,0) – (3,1,5)] | (2,1,0) [(1,1,0) – (4,1,2)] | (1,1,1) [(1,1,0) – (2,1,1)] |
|  | Parietal | Left | (1,1,1) [(1,1,0) – (2,1,1)] | (2,1,1) [(1,1,1) – (3,1,0)] | (2,1,2) [(1,1,1) – (3,1,2)] | (1,1,1) [(1,1,0) – (2,1,2)] |
|  |  | Right | (1,1,3) [(1,1,0) – (3,1,0)] | (1,1,3) [(1,1,0) – (2,1,2)] | (2,1,1) [(1,1,2) – (3,1,2)] | (1,1,1) [(1,1,0) – (2,1,2)] |
|  | Temporal | Left | (1,1,2) [(1,1,0) – (2,1,0)] | (1,1,1) [(1,1,0) – (2,1,2)] | (2,1,3) [(1,1,2) – (5,1,1)] | (1,1,3) [(1,1,0) – (2,1,3)] |
|  |  | Right | (1,1,1) [(1,1,0) – (2,1,2)] | (2,1,2) [(1,1,0) – (4,1,2)] | (2,1,1) [(1,1,3) – (3,1,1)] | (1,1,1) [(1,1,0) – (2,1,1)] |
|  | Occipital | Left | (1,1,2) [(1,1,0) – (2,1,1)] | (1,1,2) [(1,1,0) – (2,1,2)] | (2,1,1) [(1,1,1) – (3,1,5)] | (1,1,1) [(1,1,0) – (2,1,1)] |
|  |  | Right | (1,1,0) [(1,1,0) – (2,1,1)] | (1,1,4) [(1,1,0) – (3,1,1)] | (2,1,1) [(1,1,4) – (3,1,2)] | (1,1,9) [(1,1,0) – (2,1,4)] |
| HHbx | Frontal | Left | (1,1,0) [(1,1,0) – (2,1,0)] | (2,1,0) [(1,1,0) – (2,1,9)] | (2,1,2) [(1,1,6) – (3,1,3)] | (1,1,0) [(1,1,0) – (1,1,8)] |
|  |  | Right | (1,1,1) [(1,1,0) – (2,1,1)] | (1,1,2) [(1,1,0) – (2,1,3)] | (2,1,1) [(1,1,0) – (3,1,3)] | (1,1,0) [(1,1,0) – (2,1,1)] |
|  | Parietal | Left | (1,1,1) [(1,1,0) – (2,1,0)] | (2,1,0) [(1,1,0) – (3,1,2)] | (2,1,0) [(1,1,0) – (3,1,1)] | (1,1,0) [(1,1,0) – (2,1,1)] |
|  |  | Right | (1,1,2) [(1,1,0) – (2,1,2)] | (2,1,0) [(1,1,0) – (3,1,2)] | (2,1,3) [(1,1,3) – (3,1,7)] | (1,1,4) [(1,1,0) – (2,1,2)] |
|  | Temporal | Left | (1,1,1) [(1,1,0) – (2,1,0)] | (1,1,7) [(1,1,0) – (3,1,0)] | (2,1,1) [(1,1,3) – (4,1,0)] | (1,1,3) [(1,1,0) – (2,1,0)] |
|  |  | Right | (1,1,0) [(1,1,0) – (2,1,5)] | (1,1,8) [(1,1,0) – (2,1,3)] | (2,1,1) [(1,1,1) – (3,1,0)] | (1,1,3) [(1,1,0) – (2,1,2)] |
|  | Occipital | Left | (1,1,1) [(1,1,0) – (2,1,1)] | (1,1,4) [(1,1,0) – (2,1,2)] | (1,1,4) [(1,1,0) – (3,1,1)] | (1,1,1) [(1,1,0) – (2,1,2)] |
|  |  | Right | (1,1,2) [(1,1,0) – (2,1,2)] | (2,1,1) [(1,1,0) – (3,1,0)] | (2,1,0) [(1,1,2) – (3,1,0)] | (1,1,4) [(1,1,0) – (2,1,2)] |
| tHbx | Frontal | Left | (1,1,0) [(1,1,0) – (1,1,4)] | (1,1,7) [(1,1,0) – (3,1,2)] | (2,1,2) [(1,1,2) – (4,1,6)] | (1,1,1) [(1,1,0) – (2,1,2)] |
|  |  | Right | (1,1,1) [(1,1,0) – (2,1,2)] | (1,1,2) [(1,1,0) – (2,1,4)] | (1,1,10) [(1,1,1) – (3,1,4)] | (1,1,1) [(1,1,0) – (2,1,1)] |
|  | Parietal | Left | (1,1,1) [(1,1,0) – (2,1,1)] | (1,1,5) [(1,1,0) – (2,1,2)] | (2,1,1) [(1,1,1) – (3,1,2)] | (1,1,3) [(1,1,0) – (2,1,2)] |
|  |  | Right | (1,1,1) [(1,1,0) – (2,1,2)] | (1,1,1) [(1,1,0) – (2,1,0)] | (2,1,1) [(1,1,2) – (3,1,6)] | (1,1,1) [(1,1,0) – (2,1,2)] |
|  | Temporal | Left | (1,1,1) [(1,1,0) – (2,1,2)] | (2,1,2) [(1,1,1) – (3,1,1)] | (2,1,3) [(1,1,4) – (4,1,0)] | (1,1,6) [(1,1,0) – (2,1,2)] |
|  |  | Right | (1,1,0) [(1,1,0) – (2,1,1)] | (2,1,0) [(1,1,0) – (3,1,0)] | (2,1,0) [(1,1,1) – (3,1,2)] | (1,1,2) [(1,1,0) – (2,1,2)] |
|  | Occipital | Left | (1,1,2) [(1,1,0) – (2,1,2)] | (2,1,0) [(1,1,0) – (3,1,3)] | (1,1,7) [(1,1,1) – (3,1,2)] | (1,1,1) [(1,1,0) – (2,1,0)] |
|  |  | Right | (1,1,1) [(1,1,0) – (2,1,1)] | (2,1,1) [(1,1,0) – (2,1,9)] | (2,1,0) [(1,1,3) – (3,1,1)] | (2,1,0) [(1,1,0) – (3,1,0)] |
| HbDiffx | Frontal | Left | (1,1,1) [(1,1,0) – (2,1,2)] | (1,1,4) [(1,1,0) – (3,1,0)] | (2,1,1) [(1,1,1) – (3,1,2)] | (1,1,1) [(1,1,0) – (1,1,3)] |
|  |  | Right | (1,1,5) [(1,1,0) – (3,1,0)] | (2,1,0) [(1,1,0) – (2,1,2)] | (1,1,7) [(1,1,1) – (3,1,2)] | (1,1,1) [(1,1,0) – (2,1,1)] |
|  | Parietal | Left | (1,1,1) [(1,1,0) – (2,1,0)] | (2,1,1) [(1,1,2) – (3,1,0)] | (2,1,6) [(1,1,7) – (5,1,2)] | (1,1,1) [(1,1,0) – (2,1,2)] |
|  |  | Right | (1,1,0) [(1,1,0) – (2,1,2)] | (1,1,3) [(1,1,0) – (2,1,2)] | (1,1,8) [(1,1,1) – (2,1,6)] | (1,1,2) [(1,1,0) – (2,1,1)] |
|  | Temporal | Left | (1,1,1) [(1,1,0) – (3,1,0)] | (1,1,7) [(1,1,0) – (3,1,0)] | (2,1,0) [(1,1,2) – (4,1,3)] | (1,1,2) [(1,1,0) – (2,1,2)] |
|  |  | Right | (1,1,1) [(1,1,0) – (2,1,1)] | (1,1,7) [(1,1,0) – (4,1,0)] | (1,1,10) [(1,1,1) – (3,1,5)] | (1,1,2) [(1,1,0) – (2,1,0)] |
|  | Occipital | Left | (1,1,1) [(1,1,0) – (2,1,0)] | (1,1,7) [(1,1,0) – (2,1,3)] | (2,1,0) [(1,1,1) – (3,1,3)] | (1,1,1) [(1,1,0) – (2,1,2)] |
|  |  | Right | (1,1,1) [(1,1,0) – (2,1,1)] | (1,1,3) [(1,1,0) – (2,1,10)] | (2,1,0) [(1,1,1) – (3,1,2)] | (1,1,2) [(1,1,0) – (2,1,2)] |
| **250 Hz Sampled Data** | | | | | | |
| ABP | – | – | (1,1,4) [(1,1,1) – (3,1,2)] | (4,1,5) [(2,1,5) – (7,1,2)] | (3,1,4) [(2,1,3) – (5,1,3)] | (2,1,3) [(1,1,5) – (4,1,1)] |
| rSO_2_ | Frontal | Left | (1,1,3) [(1,1,1) – (2,1,2)] | (2,1,0) [(1,1,1) – (3,1,3)] | (2,1,3) [(1,1,2) – (3,1,7)] | (1,1,7) [(1,1,1) – (2,1,2)] |
|  |  | Right | (1,1,5) [(1,1,1) – (2,1,2)] | (2,1,1) [(1,1,6) – (3,1,5)] | (2,1,2) [(1,1,3) – (3,1,6)] | (1,1,5) [(1,1,1) – (2,1,3)] |
|  | Parietal | Left | (1,1,3) [(1,1,0) – (2,1,2)] | (1,1,6) [(1,1,1) – (2,1,6)] | (3,1,1) [(1,1,4) – (4,1,1)] | (1,1,5) [(1,1,1) – (2,1,3)] |
|  |  | Right | (1,1,2) [(1,1,1) – (2,1,2)] | (2,1,1) [(1,1,2) – (3,1,0)] | (3,1,1) [(1,1,3) – (4,1,4)] | (1,1,1) [(1,1,1) – (2,1,0)] |
|  | Temporal | Left | (1,1,1) [(1,1,1) – (2,1,1)] | (1,1,5) [(1,1,1) – (2,1,4)] | (2,1,4) [(1,1,6) – (5,1,2)] | (1,1,2) [(1,1,1) – (2,1,1)] |
|  |  | Right | (1,1,1) [(1,1,0) – (1,1,9)] | (2,1,0) [(1,1,2) – (2,1,7)] | (3,1,0) [(1,1,2) – (3,1,8)] | (2,1,1) [(1,1,2) – (3,1,0)] |
|  | Occipital | Left | (1,1,2) [(1,1,1) – (2,1,3)] | (1,1,2) [(1,1,1) – (2,1,3)] | (2,1,3) [(1,1,2) – (4,1,1)] | (1,1,3) [(1,1,1) – (3,1,1)] |
|  |  | Right | (1,1,3) [(1,1,1) – (2,1,0)] | (2,1,0) [(1,1,1) – (3,1,0)] | (1,1,7) [(1,1,1) – (3,1,3)] | (1,1,2) [(1,1,1) – (2,1,3)] |
| HbO | Frontal | Left | (1,1,2) [(1,1,1) – (2,1,1)] | (2,1,3) [(1,1,2) – (3,1,3)] | (2,1,4) [(1,1,3) – (4,1,7)] | (2,1,0) [(1,1,1) – (2,1,4)] |
|  |  | Right | (1,1,7) [(1,1,1) – (2,1,4)] | (2,1,0) [(1,1,1) – (2,1,5)] | (2,1,3) [(1,1,2) – (4,1,6)] | (1,1,2) [(1,1,1) – (3,1,0)] |
|  | Parietal | Left | (1,1,2) [(1,1,0) – (2,1,3)] | (2,1,1) [(1,1,1) – (3,1,1)] | (3,1,2) [(2,1,0) – (4,1,7)] | (1,1,4) [(1,1,1) – (2,1,4)] |
|  |  | Right | (1,1,2) [(1,1,1) – (2,1,2)] | (2,1,3) [(1,1,1) – (4,1,2)] | (3,1,3) [(1,1,6) – (5,1,6)] | (1,1,9) [(1,1,1) – (2,1,1)] |
|  | Temporal | Left | (1,1,1) [(1,1,0) – (2,1,1)] | (2,1,2) [(1,1,1) – (3,1,4)] | (3,1,1) [(2,1,2) – (4,1,5)] | (1,1,3) [(1,1,1) – (3,1,2)] |
|  |  | Right | (1,1,2) [(1,1,1) – (2,1,5)] | (2,1,2) [(1,1,1) – (3,1,3)] | (3,1,1) [(1,1,4) – (4,1,4)] | (1,1,6) [(1,1,1) – (3,1,0)] |
|  | Occipital | Left | (1,1,1) [(1,1,1) – (2,1,3)] | (2,1,3) [(1,1,2) – (3,1,4)] | (3,1,5) [(1,1,5) – (5,1,4)] | (1,1,2) [(1,1,1) – (2,1,3)] |
|  |  | Right | (1,1,3) [(1,1,1) – (2,1,3)] | (2,1,0) [(1,1,1) – (3,1,5)] | (3,1,8) [(2,1,3) – (6,1,3)] | (1,1,3) [(1,1,1) – (2,1,3)] |
| HHb | Frontal | Left | (1,1,3) [(1,1,1) – (2,1,2)] | (2,1,2) [(1,1,1) – (3,1,1)] | (3,1,1) [(1,1,4) – (5,1,1)] | (2,1,0) [(1,1,1) – (2,1,4)] |
|  |  | Right | (1,1,5) [(1,1,0) – (2,1,4)] | (2,1,0) [(1,1,1) – (2,1,5)] | (2,1,5) [(1,1,6) – (5,1,6)] | (2,1,0) [(1,1,1) – (3,1,2)] |
|  | Parietal | Left | (1,1,2) [(1,1,0) – (2,1,2)] | (2,1,3) [(1,1,1) – (2,1,7)] | (2,1,3) [(1,1,4) – (4,1,1)] | (1,1,2) [(1,1,1) – (2,1,2)] |
|  |  | Right | (1,1,2) [(1,1,0) – (2,1,3)] | (2,1,2) [(1,1,1) – (3,1,0)] | (3,1,3) [(2,1,2) – (5,1,1)] | (1,1,4) [(1,1,1) – (2,1,5)] |
|  | Temporal | Left | (1,1,6) [(1,1,1) – (2,1,3)] | (2,1,2) [(1,1,2) – (3,1,7)] | (2,1,6) [(1,1,2) – (4,1,3)] | (2,1,0) [(1,1,1) – (2,1,3)] |
|  |  | Right | (1,1,6) [(1,1,1) – (2,1,2)] | (2,1,0) [(1,1,1) – (3,1,0)] | (2,1,9) [(1,1,6) – (4,1,7)] | (1,1,8) [(1,1,1) – (2,1,9)] |
|  | Occipital | Left | (1,1,2) [(1,1,1) – (2,1,2)] | (2,1,1) [(1,1,1) – (2,1,4)] | (2,1,3) [(1,1,2) – (4,1,6)] | (2,1,1) [(1,1,1) – (3,1,8)] |
|  |  | Right | (1,1,3) [(1,1,0) – (2,1,5)] | (2,1,0) [(1,1,1) – (2,1,4)] | (3,1,3) [(1,1,9) – (4,1,4)] | (1,1,8) [(1,1,1) – (2,1,4)] |
| tHb | Frontal | Left | (1,1,2) [(1,1,0) – (2,1,2)] | (2,1,1) [(1,1,2) – (3,1,3)] | (2,1,6) [(1,1,4) – (5,1,9)] | (2,1,0) [(1,1,1) – (2,1,4)] |
|  |  | Right | (1,1,3) [(1,1,1) – (2,1,2)] | (2,1,1) [(1,1,3) – (3,1,0)] | (2,1,3) [(1,1,6) – (4,1,5)] | (2,1,1) [(1,1,1) – (3,1,0)] |
|  | Parietal | Left | (1,1,1) [(1,1,0) – (2,1,2)] | (1,1,8) [(1,1,1) – (2,1,4)] | (3,1,2) [(2,1,0) – (4,1,3)] | (1,1,7) [(1,1,1) – (2,1,2)] |
|  |  | Right | (1,1,3) [(1,1,0) – (2,1,3)] | (2,1,1) [(1,1,1) – (3,1,3)] | (3,1,4) [(1,1,10) – (4,1,9)] | (2,1,0) [(1,1,1) – (2,1,4)] |
|  | Temporal | Left | (1,1,1) [(1,1,0) – (2,1,1)] | (2,1,0) [(1,1,1) – (3,1,2)] | (2,1,9) [(1,1,8) – (4,1,6)] | (1,1,3) [(1,1,1) – (2,1,1)] |
|  |  | Right | (1,1,8) [(1,1,1) – (3,1,0)] | (2,1,0) [(1,1,1) – (2,1,5)] | (2,1,10) [(1,1,3) – (4,1,4)] | (1,1,5) [(1,1,1) – (2,1,4)] |
|  | Occipital | Left | (1,1,3) [(1,1,1) – (2,1,3)] | (1,1,8) [(1,1,1) – (3,1,1)] | (3,1,1) [(1,1,9) – (4,1,3)] | (1,1,5) [(1,1,1) – (2,1,6)] |
|  |  | Right | (1,1,3) [(1,1,1) – (2,1,1)] | (2,1,1) [(1,1,1) – (3,1,0)] | (3,1,5) [(2,1,2) – (4,1,4)] | (1,1,3) [(1,1,1) – (2,1,3)] |
| HbDiff | Frontal | Left | (1,1,2) [(1,1,1) – (2,1,3)] | (2,1,2) [(1,1,1) – (3,1,3)] | (3,1,2) [(1,1,10) – (5,1,1)] | (2,1,0) [(1,1,1) – (3,1,0)] |
|  |  | Right | (1,1,5) [(1,1,1) – (2,1,6)] | (2,1,0) [(1,1,2) – (3,1,3)] | (2,1,3) [(2,1,0) – (5,1,5)] | (2,1,2) [(1,1,2) – (3,1,3)] |
|  | Parietal | Left | (1,1,1) [(1,1,0) – (1,1,6)] | (2,1,2) [(1,1,1) – (3,1,3)] | (2,1,6) [(1,1,9) – (3,1,7)] | (1,1,2) [(1,1,1) – (2,1,3)] |
|  |  | Right | (1,1,6) [(1,1,1) – (2,1,2)] | (2,1,3) [(1,1,2) – (3,1,5)] | (2,1,4) [(1,1,3) – (4,1,3)] | (1,1,1) [(1,1,1) – (2,1,1)] |
|  | Temporal | Left | (1,1,1) [(1,1,1) – (2,1,3)] | (2,1,2) [(1,1,2) – (3,1,4)] | (3,1,4) [(2,1,1) – (5,1,1)] | (2,1,0) [(1,1,1) – (3,1,0)] |
|  |  | Right | (1,1,2) [(1,1,0) – (2,1,2)] | (2,1,3) [(1,1,6) – (3,1,2)] | (2,1,5) [(1,1,3) – (4,1,3)] | (2,1,2) [(1,1,2) – (4,1,0)] |
|  | Occipital | Left | (1,1,2) [(1,1,1) – (2,1,3)] | (2,1,3) [(1,1,1) – (3,1,1)] | (3,1,10) [(2,1,1) – (5,1,6)] | (1,1,2) [(1,1,1) – (2,1,3)] |
|  |  | Right | (1,1,5) [(1,1,1) – (2,1,1)] | (2,1,3) [(1,1,2) – (3,1,3)] | (3,1,1) [(1,1,3) – (5,1,4)] | (1,1,4) [(1,1,1) – (2,1,4)] |
| COx-a | Frontal | Left | (1,1,2) [(1,1,0) – (2,1,3)] | (2,1,0) [(1,1,0) – (3,1,1)] | (2,1,1) [(1,1,1) – (4,1,2)] | (1,1,1) [(1,1,0) – (1,1,9)] |
|  |  | Right | (1,1,2) [(1,1,0) – (2,1,2)] | (1,1,9) [(1,1,0) – (2,1,2)] | (2,1,2) [(1,1,3) – (3,1,4)] | (1,1,1) [(1,1,0) – (2,1,1)] |
|  | Parietal | Left | (1,1,2) [(1,1,0) – (2,1,2)] | (2,1,1) [(1,1,1) – (3,1,1)] | (2,1,4) [(1,1,3) – (3,1,6)] | (1,1,2) [(1,1,0) – (2,1,2)] |
|  |  | Right | (1,1,2) [(1,1,0) – (2,1,1)] | (1,1,6) [(1,1,0) – (2,1,8)] | (2,1,0) [(1,1,1) – (4,1,2)] | (1,1,5) [(1,1,0) – (2,1,2)] |
|  | Temporal | Left | (1,1,1) [(1,1,0) – (2,1,1)] | (1,1,7) [(1,1,0) – (3,1,0)] | (1,1,8) [(1,1,0) – (3,1,0)] | (1,1,1) [(1,1,0) – (2,1,0)] |
|  |  | Right | (1,1,3) [(1,1,0) – (2,1,2)] | (2,1,2) [(1,1,1) – (3,1,3)] | (1,1,10) [(1,1,1) – (3,1,2)] | (1,1,3) [(1,1,0) – (2,1,2)] |
|  | Occipital | Left | (1,1,1) [(1,1,0) – (2,1,1)] | (1,1,4) [(1,1,0) – (2,1,2)] | (2,1,0) [(1,1,0) – (3,1,3)] | (1,1,1) [(1,1,0) – (2,1,2)] |
|  |  | Right | (1,1,1) [(1,1,0) – (2,1,1)] | (2,1,0) [(1,1,0) – (2,1,10)] | (2,1,0) [(1,1,0) – (3,1,0)] | (1,1,3) [(1,1,0) – (2,1,1)] |
| HbOx | Frontal | Left | (1,1,1) [(1,1,0) – (2,1,4)] | (1,1,7) [(1,1,0) – (2,1,4)] | (2,1,3) [(1,1,5) – (3,1,5)] | (1,1,2) [(1,1,0) – (2,1,2)] |
|  |  | Right | (1,1,1) [(1,1,0) – (2,1,2)] | (1,1,5) [(1,1,0) – (2,1,6)] | (1,1,4) [(1,1,0) – (2,1,8)] | (1,1,2) [(1,1,0) – (2,1,0)] |
|  | Parietal | Left | (1,1,2) [(1,1,0) – (2,1,1)] | (1,1,2) [(1,1,0) – (2,1,2)] | (1,1,7) [(1,1,1) – (3,1,1)] | (1,1,2) [(1,1,0) – (2,1,0)] |
|  |  | Right | (1,1,2) [(1,1,0) – (2,1,1)] | (1,1,2) [(1,1,0) – (2,1,6)] | (2,1,1) [(1,1,1) – (3,1,0)] | (1,1,3) [(1,1,0) – (2,1,2)] |
|  | Temporal | Left | (1,1,2) [(1,1,0) – (2,1,1)] | (1,1,6) [(1,1,0) – (2,1,3)] | (2,1,1) [(1,1,1) – (4,1,1)] | (1,1,7) [(1,1,0) – (2,1,6)] |
|  |  | Right | (1,1,1) [(1,1,0) – (4,1,0)] | (2,1,2) [(1,1,0) – (3,1,3)] | (1,1,9) [(1,1,2) – (3,1,2)] | (1,1,2) [(1,1,0) – (2,1,2)] |
|  | Occipital | Left | (1,1,3) [(1,1,0) – (2,1,3)] | (2,1,0) [(1,1,1) – (2,1,3)] | (2,1,0) [(1,1,1) – (3,1,3)] | (1,1,1) [(1,1,0) – (2,1,2)] |
|  |  | Right | (1,1,0) [(1,1,0) – (1,1,6)] | (1,1,2) [(1,1,0) – (3,1,3)] | (1,1,8) [(1,1,0) – (3,1,2)] | (1,1,2) [(1,1,0) – (2,1,2)] |
| HHbx | Frontal | Left | (1,1,1) [(1,1,0) – (2,1,0)] | (1,1,7) [(1,1,0) – (2,1,3)] | (2,1,2) [(1,1,3) – (3,1,8)] | (1,1,1) [(1,1,0) – (2,1,0)] |
|  |  | Right | (1,1,1) [(1,1,0) – (2,1,0)] | (1,1,3) [(1,1,0) – (3,1,0)] | (1,1,9) [(1,1,0) – (3,1,6)] | (1,1,2) [(1,1,0) – (2,1,2)] |
|  | Parietal | Left | (1,1,1) [(1,1,0) – (2,1,0)] | (2,1,1) [(1,1,1) – (2,1,2)] | (2,1,1) [(1,1,1) – (3,1,3)] | (1,1,1) [(1,1,0) – (2,1,1)] |
|  |  | Right | (1,1,2) [(1,1,0) – (3,1,0)] | (1,1,3) [(1,1,0) – (2,1,2)] | (1,1,8) [(1,1,0) – (4,1,2)] | (1,1,6) [(1,1,0) – (2,1,2)] |
|  | Temporal | Left | (1,1,1) [(1,1,0) – (2,1,1)] | (2,1,2) [(1,1,1) – (2,1,3)] | (2,1,1) [(1,1,3) – (3,1,7)] | (1,1,2) [(1,1,0) – (2,1,2)] |
|  |  | Right | (1,1,0) [(1,1,0) – (2,1,2)] | (1,1,5) [(1,1,0) – (3,1,0)] | (2,1,0) [(1,1,2) – (3,1,4)] | (1,1,2) [(1,1,0) – (2,1,0)] |
|  | Occipital | Left | (1,1,0) [(1,1,0) – (2,1,3)] | (1,1,10) [(1,1,0) – (2,1,2)] | (1,1,1) [(1,1,0) – (3,1,0)] | (1,1,2) [(1,1,0) – (2,1,2)] |
|  |  | Right | (1,1,2) [(1,1,0) – (2,1,1)] | (2,1,0) [(1,1,0) – (2,1,5)] | (1,1,10) [(1,1,0) – (2,1,7)] | (1,1,5) [(1,1,0) – (2,1,1)] |
| tHbx | Frontal | Left | (1,1,1) [(1,1,0) – (2,1,0)] | (1,1,3) [(1,1,0) – (2,1,2)] | (2,1,0) [(1,1,0) – (4,1,3)] | (1,1,1) [(1,1,0) – (2,1,2)] |
|  |  | Right | (1,1,1) [(1,1,0) – (2,1,1)] | (1,1,6) [(1,1,0) – (2,1,4)] | (2,1,0) [(1,1,2) – (3,1,0)] | (1,1,1) [(1,1,0) – (2,1,2)] |
|  | Parietal | Left | (1,1,1) [(1,1,0) – (2,1,2)] | (1,1,5) [(1,1,0) – (2,1,2)] | (2,1,1) [(1,1,3) – (3,1,2)] | (1,1,1) [(1,1,0) – (2,1,1)] |
|  |  | Right | (1,1,3) [(1,1,0) – (2,1,2)] | (1,1,2) [(1,1,0) – (3,1,5)] | (2,1,1) [(1,1,1) – (4,1,0)] | (1,1,1) [(1,1,0) – (2,1,1)] |
|  | Temporal | Left | (1,1,4) [(1,1,0) – (3,1,1)] | (2,1,2) [(1,1,1) – (3,1,1)] | (2,1,4) [(1,1,1) – (3,1,5)] | (1,1,6) [(1,1,0) – (3,1,0)] |
|  |  | Right | (1,1,1) [(1,1,0) – (2,1,1)] | (2,1,0) [(1,1,1) – (3,1,0)] | (2,1,0) [(1,1,0) – (2,1,7)] | (1,1,2) [(1,1,0) – (2,1,1)] |
|  | Occipital | Left | (1,1,1) [(1,1,0) – (2,1,1)] | (1,1,3) [(1,1,0) – (2,1,2)] | (1,1,7) [(1,1,1) – (2,1,4)] | (1,1,2) [(1,1,0) – (2,1,5)] |
|  |  | Right | (1,1,1) [(1,1,0) – (2,1,2)] | (2,1,0) [(1,1,0) – (3,1,0)] | (1,1,8) [(1,1,2) – (3,1,0)] | (2,1,0) [(1,1,0) – (2,1,2)] |
| HbDiffx | Frontal | Left | (1,1,4) [(1,1,0) – (2,1,2)] | (2,1,0) [(1,1,0) – (3,1,1)] | (2,1,1) [(1,1,0) – (3,1,2)] | (1,1,1) [(1,1,0) – (1,1,4)] |
|  |  | Right | (2,1,1) [(1,1,0) – (5,1,0)] | (2,1,0) [(1,1,1) – (2,1,2)] | (2,1,3) [(1,1,1) – (4,1,3)] | (1,1,1) [(1,1,0) – (2,1,2)] |
|  | Parietal | Left | (1,1,2) [(1,1,0) – (2,1,2)] | (2,1,2) [(1,1,2) – (3,1,1)] | (2,1,3) [(1,1,6) – (3,1,7)] | (1,1,1) [(1,1,0) – (2,1,1)] |
|  |  | Right | (1,1,2) [(1,1,0) – (2,1,2)] | (2,1,0) [(1,1,0) – (2,1,2)] | (1,1,7) [(1,1,1) – (4,1,0)] | (1,1,1) [(1,1,0) – (2,1,2)] |
|  | Temporal | Left | (1,1,1) [(1,1,0) – (2,1,1)] | (1,1,6) [(1,1,0) – (2,1,2)] | (1,1,6) [(1,1,0) – (4,1,5)] | (1,1,2) [(1,1,0) – (2,1,1)] |
|  |  | Right | (1,1,4) [(1,1,0) – (2,1,3)] | (2,1,2) [(1,1,1) – (3,1,4)] | (1,1,7) [(1,1,1) – (2,1,4)] | (1,1,2) [(1,1,0) – (2,1,2)] |
|  | Occipital | Left | (1,1,1) [(1,1,0) – (2,1,1)] | (1,1,2) [(1,1,0) – (2,1,2)] | (1,1,2) [(1,1,1) – (3,1,2)] | (1,1,2) [(1,1,0) – (2,1,2)] |
|  |  | Right | (1,1,1) [(1,1,0) – (2,1,0)] | (1,1,3) [(1,1,0) – (2,1,10)] | (2,1,5) [(1,1,1) – (3,1,6)] | (1,1,2) [(1,1,0) – (2,1,2)] |
| The table provides perturbation subgrouped median and IQR of optimal ARIMA models based on AIC for physiologic signals using data in 1 Hz and 250 Hz frequencies. *AIC, Akaike Information Criterion; ARIMA, autoregressive integrative moving average; COx-a, cerebral oximetry index with arterial blood pressure; HbDiff, hemoglobin difference; HbDiffx, hemoglobin difference index; HbO, oxyhemoglobin; HbOx, oxyhemoglobin index; HHb, deoxyhemoglobin; HHbx, deoxyhemoglobin index; IQR, interquartile range; MAD, median absolute deviation; rSO_2_, regional cerebral oxygen saturation; tHb, total hemoglobin; tHbx, total hemoglobin index.* | | | | | | |

Appendix S7i: Perturbation Subgrouped Hemispheric Responsiveness using Impulse Response Coefficients of Optimal VARIMA Model

| **Signal Combination** | **Brain Lobe** | **Hemisphere** | **Direction** | **Hemispheric Responsiveness using Impulse Response Coefficients of Optimal VARIMA Model [% (count)] for Perturbation Subgroups** | | | | | | | |
| --- | --- | --- | --- | --- | --- | --- | --- | --- | --- | --- | --- |
|  |  |  |  | **Baseline** | | **Neurovascular Coupling** | | **Orthostatic Challenge** | | **Vascular Chemo-Reactivity** | |
|  |  |  |  | **>0.1%** | **NA** | **>0.1%** | **NA** | **>0.1%** | **NA** | **>0.1%** | **NA** |
| **1 Hz Sampled Data** | | | | | | | | | | | |
| ABP & rSO_2_ | Frontal | Left | ABP ® rSO_2_ | 62% (31) | 12% (6) | 80% (40) | 2% (1) | 86% (43) | 2% (1) | 80% (40) | 2% (1) |
|  |  |  | rSO_2_ ® ABP | 58% (29) | 12% (6) | 80% (40) | 2% (1) | 86% (43) | 2% (1) | 80% (40) | 2% (1) |
|  |  | Right | ABP ® rSO_2_ | 68% (34) | 8% (4) | 88% (44) | 2% (1) | 84% (42) | 6% (3) | 64% (32) | 2% (1) |
|  |  |  | rSO_2_ ® ABP | 68% (34) | 8% (4) | 92% (46) | 2% (1) | 88% (44) | 6% (3) | 68% (34) | 2% (1) |
|  | Parietal | Left | ABP ® rSO_2_ | 60% (30) | 6% (3) | 94% (47) | 2% (1) | 86% (43) | 2% (1) | 80% (40) | 0% (0) |
|  |  |  | rSO_2_ ® ABP | 62% (31) | 6% (3) | 92% (46) | 2% (1) | 84% (42) | 2% (1) | 82% (41) | 0% (0) |
|  |  | Right | ABP ® rSO_2_ | 66% (33) | 8% (4) | 84% (42) | 6% (3) | 88% (44) | 0% (0) | 72% (36) | 6% (3) |
|  |  |  | rSO_2_ ® ABP | 68% (34) | 8% (4) | 84% (42) | 6% (3) | 90% (45) | 0% (0) | 70% (35) | 6% (3) |
|  | Occipital | Left | ABP ® rSO_2_ | 76% (38) | 6% (3) | 96% (48) | 0% (0) | 88% (44) | 2% (1) | 78% (39) | 2% (1) |
|  |  |  | rSO_2_ ® ABP | 68% (34) | 6% (3) | 92% (46) | 0% (0) | 88% (44) | 2% (1) | 76% (38) | 2% (1) |
|  |  | Right | ABP ® rSO_2_ | 60% (30) | 4% (2) | 88% (44) | 0% (0) | 92% (46) | 0% (0) | 68% (34) | 6% (3) |
|  |  |  | rSO_2_ ® ABP | 58% (29) | 4% (2) | 92% (46) | 0% (0) | 90% (45) | 0% (0) | 76% (38) | 6% (3) |
|  | Temporal | Left | ABP ® rSO_2_ | 60% (30) | 12% (6) | 84% (42) | 2% (1) | 92% (46) | 2% (1) | 92% (46) | 4% (2) |
|  |  |  | rSO_2_ ® ABP | 64% (32) | 12% (6) | 80% (40) | 2% (1) | 92% (46) | 2% (1) | 82% (41) | 4% (2) |
|  |  | Right | ABP ® rSO_2_ | 74% (37) | 6% (3) | 82% (41) | 4% (2) | 88% (44) | 0% (0) | 78% (39) | 0% (0) |
|  |  |  | rSO_2_ ® ABP | 70% (35) | 6% (3) | 82% (41) | 4% (2) | 88% (44) | 0% (0) | 76% (38) | 0% (0) |
| ABP & HbO | Frontal | Left | ABP ® HbO | 70% (35) | 6% (3) | 88% (44) | 4% (2) | 90% (45) | 0% (0) | 74% (37) | 6% (3) |
|  |  |  | HbO ® ABP | 66% (33) | 6% (3) | 86% (43) | 4% (2) | 90% (45) | 0% (0) | 78% (39) | 6% (3) |
|  |  | Right | ABP ® HbO | 70% (35) | 4% (2) | 90% (45) | 2% (1) | 94% (47) | 0% (0) | 74% (37) | 2% (1) |
|  |  |  | HbO ® ABP | 70% (35) | 4% (2) | 88% (44) | 2% (1) | 92% (46) | 0% (0) | 68% (34) | 2% (1) |
|  | Parietal | Left | ABP ® HbO | 82% (41) | 2% (1) | 94% (47) | 2% (1) | 84% (42) | 2% (1) | 86% (43) | 2% (1) |
|  |  |  | HbO ® ABP | 74% (37) | 2% (1) | 90% (45) | 2% (1) | 84% (42) | 2% (1) | 80% (40) | 2% (1) |
|  |  | Right | ABP ® HbO | 66% (33) | 4% (2) | 88% (44) | 6% (3) | 76% (38) | 4% (2) | 74% (37) | 4% (2) |
|  |  |  | HbO ® ABP | 60% (30) | 4% (2) | 88% (44) | 6% (3) | 72% (36) | 4% (2) | 80% (40) | 4% (2) |
|  | Occipital | Left | ABP ® HbO | 70% (35) | 2% (1) | 82% (41) | 6% (3) | 78% (39) | 4% (2) | 78% (39) | 2% (1) |
|  |  |  | HbO ® ABP | 68% (34) | 2% (1) | 82% (41) | 6% (3) | 86% (43) | 4% (2) | 76% (38) | 2% (1) |
|  |  | Right | ABP ® HbO | 72% (36) | 10% (5) | 86% (43) | 0% (0) | 92% (46) | 0% (0) | 86% (43) | 4% (2) |
|  |  |  | HbO ® ABP | 74% (37) | 10% (5) | 88% (44) | 0% (0) | 90% (45) | 0% (0) | 80% (40) | 4% (2) |
|  | Temporal | Left | ABP ® HbO | 66% (33) | 6% (3) | 84% (42) | 2% (1) | 90% (45) | 0% (0) | 88% (44) | 4% (2) |
|  |  |  | HbO ® ABP | 68% (34) | 6% (3) | 84% (42) | 2% (1) | 90% (45) | 0% (0) | 82% (41) | 4% (2) |
|  |  | Right | ABP ® HbO | 70% (35) | 8% (4) | 84% (42) | 4% (2) | 88% (44) | 0% (0) | 76% (38) | 2% (1) |
|  |  |  | HbO ® ABP | 64% (32) | 8% (4) | 84% (42) | 4% (2) | 94% (47) | 0% (0) | 78% (39) | 2% (1) |
| ABP & HHb | Frontal | Left | ABP ® HHb | 76% (38) | 2% (1) | 88% (44) | 2% (1) | 86% (43) | 0% (0) | 78% (39) | 4% (2) |
|  |  |  | HHb ® ABP | 72% (36) | 2% (1) | 86% (43) | 2% (1) | 88% (44) | 0% (0) | 82% (41) | 4% (2) |
|  |  | Right | ABP ® HHb | 70% (35) | 10% (5) | 84% (42) | 4% (2) | 84% (42) | 4% (2) | 64% (32) | 6% (3) |
|  |  |  | HHb ® ABP | 72% (36) | 10% (5) | 82% (41) | 4% (2) | 82% (41) | 4% (2) | 70% (35) | 6% (3) |
|  | Parietal | Left | ABP ® HHb | 62% (31) | 8% (4) | 90% (45) | 4% (2) | 88% (44) | 0% (0) | 76% (38) | 0% (0) |
|  |  |  | HHb ® ABP | 64% (32) | 8% (4) | 84% (42) | 4% (2) | 90% (45) | 0% (0) | 80% (40) | 0% (0) |
|  |  | Right | ABP ® HHb | 58% (29) | 6% (3) | 90% (45) | 4% (2) | 92% (46) | 0% (0) | 72% (36) | 4% (2) |
|  |  |  | HHb ® ABP | 60% (30) | 6% (3) | 88% (44) | 4% (2) | 88% (44) | 0% (0) | 66% (33) | 4% (2) |
|  | Occipital | Left | ABP ® HHb | 76% (38) | 6% (3) | 90% (45) | 6% (3) | 88% (44) | 2% (1) | 76% (38) | 6% (3) |
|  |  |  | HHb ® ABP | 72% (36) | 6% (3) | 88% (44) | 6% (3) | 92% (46) | 2% (1) | 74% (37) | 6% (3) |
|  |  | Right | ABP ® HHb | 70% (35) | 8% (4) | 92% (46) | 0% (0) | 94% (47) | 0% (0) | 82% (41) | 2% (1) |
|  |  |  | HHb ® ABP | 76% (38) | 8% (4) | 92% (46) | 0% (0) | 92% (46) | 0% (0) | 86% (43) | 2% (1) |
|  | Temporal | Left | ABP ® HHb | 78% (39) | 6% (3) | 86% (43) | 0% (0) | 94% (47) | 0% (0) | 84% (42) | 8% (4) |
|  |  |  | HHb ® ABP | 76% (38) | 6% (3) | 86% (43) | 0% (0) | 94% (47) | 0% (0) | 78% (39) | 8% (4) |
|  |  | Right | ABP ® HHb | 68% (34) | 10% (5) | 84% (42) | 2% (1) | 82% (41) | 6% (3) | 82% (41) | 2% (1) |
|  |  |  | HHb ® ABP | 70% (35) | 10% (5) | 84% (42) | 2% (1) | 80% (40) | 6% (3) | 82% (41) | 2% (1) |
| ABP & tHb | Frontal | Left | ABP ® tHb | 80% (40) | 2% (1) | 84% (42) | 8% (4) | 92% (46) | 2% (1) | 86% (43) | 2% (1) |
|  |  |  | tHb ® ABP | 78% (39) | 2% (1) | 88% (44) | 8% (4) | 92% (46) | 2% (1) | 84% (42) | 2% (1) |
|  |  | Right | ABP ® tHb | 68% (34) | 4% (2) | 90% (45) | 0% (0) | 86% (43) | 6% (3) | 76% (38) | 2% (1) |
|  |  |  | tHb ® ABP | 72% (36) | 4% (2) | 92% (46) | 0% (0) | 84% (42) | 6% (3) | 80% (40) | 2% (1) |
|  | Parietal | Left | ABP ® tHb | 64% (32) | 6% (3) | 90% (45) | 0% (0) | 86% (43) | 0% (0) | 78% (39) | 0% (0) |
|  |  |  | tHb ® ABP | 68% (34) | 6% (3) | 90% (45) | 0% (0) | 88% (44) | 0% (0) | 78% (39) | 0% (0) |
|  |  | Right | ABP ® tHb | 68% (34) | 6% (3) | 88% (44) | 2% (1) | 94% (47) | 2% (1) | 62% (31) | 8% (4) |
|  |  |  | tHb ® ABP | 70% (35) | 6% (3) | 90% (45) | 2% (1) | 92% (46) | 2% (1) | 64% (32) | 8% (4) |
|  | Occipital | Left | ABP ® tHb | 68% (34) | 12% (6) | 86% (43) | 2% (1) | 80% (40) | 2% (1) | 66% (33) | 4% (2) |
|  |  |  | tHb ® ABP | 60% (30) | 12% (6) | 86% (43) | 2% (1) | 88% (44) | 2% (1) | 72% (36) | 4% (2) |
|  |  | Right | ABP ® tHb | 76% (38) | 6% (3) | 88% (44) | 0% (0) | 82% (41) | 2% (1) | 78% (39) | 6% (3) |
|  |  |  | tHb ® ABP | 74% (37) | 6% (3) | 88% (44) | 0% (0) | 84% (42) | 2% (1) | 72% (36) | 6% (3) |
|  | Temporal | Left | ABP ® tHb | 76% (38) | 2% (1) | 84% (42) | 2% (1) | 92% (46) | 0% (0) | 82% (41) | 2% (1) |
|  |  |  | tHb ® ABP | 72% (36) | 2% (1) | 84% (42) | 2% (1) | 90% (45) | 0% (0) | 74% (37) | 2% (1) |
|  |  | Right | ABP ® tHb | 68% (34) | 4% (2) | 88% (44) | 2% (1) | 88% (44) | 2% (1) | 78% (39) | 0% (0) |
|  |  |  | tHb ® ABP | 66% (33) | 4% (2) | 88% (44) | 2% (1) | 88% (44) | 2% (1) | 84% (42) | 0% (0) |
| ABP & HbDiff | Frontal | Left | ABP ® HbDiff | 58% (29) | 14% (7) | 88% (44) | 4% (2) | 88% (44) | 0% (0) | 76% (38) | 6% (3) |
|  |  |  | HbDiff ® ABP | 66% (33) | 14% (7) | 84% (42) | 4% (2) | 90% (45) | 0% (0) | 74% (37) | 6% (3) |
|  |  | Right | ABP ® HbDiff | 60% (30) | 12% (6) | 88% (44) | 4% (2) | 92% (46) | 2% (1) | 74% (37) | 2% (1) |
|  |  |  | HbDiff ® ABP | 64% (32) | 12% (6) | 88% (44) | 4% (2) | 90% (45) | 2% (1) | 74% (37) | 2% (1) |
|  | Parietal | Left | ABP ® HbDiff | 66% (33) | 4% (2) | 94% (47) | 4% (2) | 84% (42) | 4% (2) | 86% (43) | 2% (1) |
|  |  |  | HbDiff ® ABP | 76% (38) | 4% (2) | 92% (46) | 4% (2) | 88% (44) | 4% (2) | 84% (42) | 2% (1) |
|  |  | Right | ABP ® HbDiff | 64% (32) | 12% (6) | 84% (42) | 8% (4) | 90% (45) | 0% (0) | 72% (36) | 4% (2) |
|  |  |  | HbDiff ® ABP | 52% (26) | 12% (6) | 84% (42) | 8% (4) | 92% (46) | 0% (0) | 74% (37) | 4% (2) |
|  | Occipital | Left | ABP ® HbDiff | 68% (34) | 6% (3) | 86% (43) | 2% (1) | 96% (48) | 2% (1) | 82% (41) | 4% (2) |
|  |  |  | HbDiff ® ABP | 62% (31) | 6% (3) | 90% (45) | 2% (1) | 96% (48) | 2% (1) | 84% (42) | 4% (2) |
|  |  | Right | ABP ® HbDiff | 60% (30) | 4% (2) | 84% (42) | 6% (3) | 90% (45) | 0% (0) | 74% (37) | 8% (4) |
|  |  |  | HbDiff ® ABP | 60% (30) | 4% (2) | 86% (43) | 6% (3) | 90% (45) | 0% (0) | 76% (38) | 8% (4) |
|  | Temporal | Left | ABP ® HbDiff | 68% (34) | 8% (4) | 90% (45) | 0% (0) | 92% (46) | 2% (1) | 88% (44) | 4% (2) |
|  |  |  | HbDiff ® ABP | 68% (34) | 8% (4) | 92% (46) | 0% (0) | 92% (46) | 2% (1) | 78% (39) | 4% (2) |
|  |  | Right | ABP ® HbDiff | 80% (40) | 4% (2) | 80% (40) | 6% (3) | 88% (44) | 0% (0) | 78% (39) | 2% (1) |
|  |  |  | HbDiff ® ABP | 74% (37) | 4% (2) | 80% (40) | 6% (3) | 84% (42) | 0% (0) | 80% (40) | 2% (1) |
| **250 Hz Sampled Data** | | | | | | | | | | | |
| ABP & rSO_2_ | Frontal | Left | ABP ® rSO_2_ | 78% (39) | 0% (0) | 88% (44) | 4% (2) | 76% (38) | 0% (0) | 84% (42) | 4% (2) |
|  |  |  | rSO_2_ ® ABP | 76% (38) | 0% (0) | 90% (45) | 4% (2) | 78% (39) | 0% (0) | 82% (41) | 4% (2) |
|  |  | Right | ABP ® rSO_2_ | 80% (40) | 2% (1) | 88% (44) | 0% (0) | 86% (43) | 0% (0) | 74% (37) | 4% (2) |
|  |  |  | rSO_2_ ® ABP | 84% (42) | 2% (1) | 94% (47) | 0% (0) | 88% (44) | 0% (0) | 80% (40) | 4% (2) |
|  | Parietal | Left | ABP ® rSO_2_ | 80% (40) | 4% (2) | 92% (46) | 6% (3) | 80% (40) | 2% (1) | 88% (44) | 4% (2) |
|  |  |  | rSO_2_ ® ABP | 80% (40) | 4% (2) | 90% (45) | 6% (3) | 84% (42) | 2% (1) | 84% (42) | 4% (2) |
|  |  | Right | ABP ® rSO_2_ | 68% (34) | 8% (4) | 86% (43) | 6% (3) | 80% (40) | 2% (1) | 72% (36) | 2% (1) |
|  |  |  | rSO_2_ ® ABP | 72% (36) | 8% (4) | 84% (42) | 6% (3) | 76% (38) | 2% (1) | 78% (39) | 2% (1) |
|  | Occipital | Left | ABP ® rSO_2_ | 72% (36) | 4% (2) | 90% (45) | 4% (2) | 80% (40) | 2% (1) | 80% (40) | 2% (1) |
|  |  |  | rSO_2_ ® ABP | 70% (35) | 4% (2) | 86% (43) | 4% (2) | 78% (39) | 2% (1) | 72% (36) | 2% (1) |
|  |  | Right | ABP ® rSO_2_ | 64% (32) | 4% (2) | 90% (45) | 0% (0) | 90% (45) | 2% (1) | 78% (39) | 4% (2) |
|  |  |  | rSO_2_ ® ABP | 68% (34) | 4% (2) | 90% (45) | 0% (0) | 84% (42) | 2% (1) | 78% (39) | 4% (2) |
|  | Temporal | Left | ABP ® rSO_2_ | 64% (32) | 8% (4) | 82% (41) | 2% (1) | 88% (44) | 0% (0) | 78% (39) | 8% (4) |
|  |  |  | rSO_2_ ® ABP | 68% (34) | 8% (4) | 82% (41) | 2% (1) | 88% (44) | 0% (0) | 72% (36) | 8% (4) |
|  |  | Right | ABP ® rSO_2_ | 72% (36) | 2% (1) | 82% (41) | 2% (1) | 84% (42) | 0% (0) | 70% (35) | 8% (4) |
|  |  |  | rSO_2_ ® ABP | 70% (35) | 2% (1) | 84% (42) | 2% (1) | 86% (43) | 0% (0) | 66% (33) | 8% (4) |
| ABP & HbO | Frontal | Left | ABP ® HbO | 78% (39) | 4% (2) | 84% (42) | 6% (3) | 86% (43) | 2% (1) | 80% (40) | 6% (3) |
|  |  |  | HbO ® ABP | 80% (40) | 4% (2) | 84% (42) | 6% (3) | 86% (43) | 2% (1) | 78% (39) | 6% (3) |
|  |  | Right | ABP ® HbO | 68% (34) | 6% (3) | 94% (47) | 0% (0) | 82% (41) | 0% (0) | 84% (42) | 6% (3) |
|  |  |  | HbO ® ABP | 68% (34) | 6% (3) | 88% (44) | 0% (0) | 84% (42) | 0% (0) | 80% (40) | 6% (3) |
|  | Parietal | Left | ABP ® HbO | 78% (39) | 2% (1) | 84% (42) | 6% (3) | 86% (43) | 0% (0) | 78% (39) | 4% (2) |
|  |  |  | HbO ® ABP | 84% (42) | 2% (1) | 86% (43) | 6% (3) | 86% (43) | 0% (0) | 78% (39) | 4% (2) |
|  |  | Right | ABP ® HbO | 66% (33) | 4% (2) | 86% (43) | 6% (3) | 80% (40) | 6% (3) | 72% (36) | 2% (1) |
|  |  |  | HbO ® ABP | 64% (32) | 4% (2) | 88% (44) | 6% (3) | 76% (38) | 6% (3) | 78% (39) | 2% (1) |
|  | Occipital | Left | ABP ® HbO | 76% (38) | 4% (2) | 84% (42) | 2% (1) | 96% (48) | 0% (0) | 80% (40) | 2% (1) |
|  |  |  | HbO ® ABP | 76% (38) | 4% (2) | 84% (42) | 2% (1) | 98% (49) | 0% (0) | 78% (39) | 2% (1) |
|  |  | Right | ABP ® HbO | 68% (34) | 8% (4) | 94% (47) | 2% (1) | 80% (40) | 2% (1) | 78% (39) | 10% (5) |
|  |  |  | HbO ® ABP | 68% (34) | 8% (4) | 94% (47) | 2% (1) | 88% (44) | 2% (1) | 78% (39) | 10% (5) |
|  | Temporal | Left | ABP ® HbO | 60% (30) | 10% (5) | 88% (44) | 2% (1) | 88% (44) | 0% (0) | 86% (43) | 0% (0) |
|  |  |  | HbO ® ABP | 64% (32) | 10% (5) | 84% (42) | 2% (1) | 90% (45) | 0% (0) | 88% (44) | 0% (0) |
|  |  | Right | ABP ® HbO | 68% (34) | 6% (3) | 86% (43) | 8% (4) | 82% (41) | 4% (2) | 78% (39) | 2% (1) |
|  |  |  | HbO ® ABP | 74% (37) | 6% (3) | 84% (42) | 8% (4) | 84% (42) | 4% (2) | 72% (36) | 2% (1) |
| ABP & HHb | Frontal | Left | ABP ® HHb | 68% (34) | 6% (3) | 80% (40) | 6% (3) | 86% (43) | 0% (0) | 74% (37) | 8% (4) |
|  |  |  | HHb ® ABP | 74% (37) | 6% (3) | 80% (40) | 6% (3) | 86% (43) | 0% (0) | 76% (38) | 8% (4) |
|  |  | Right | ABP ® HHb | 70% (35) | 6% (3) | 82% (41) | 0% (0) | 90% (45) | 4% (2) | 80% (40) | 6% (3) |
|  |  |  | HHb ® ABP | 66% (33) | 6% (3) | 84% (42) | 0% (0) | 86% (43) | 4% (2) | 76% (38) | 6% (3) |
|  | Parietal | Left | ABP ® HHb | 60% (30) | 8% (4) | 88% (44) | 2% (1) | 86% (43) | 0% (0) | 78% (39) | 4% (2) |
|  |  |  | HHb ® ABP | 64% (32) | 8% (4) | 86% (43) | 2% (1) | 86% (43) | 0% (0) | 80% (40) | 4% (2) |
|  |  | Right | ABP ® HHb | 56% (28) | 10% (5) | 90% (45) | 4% (2) | 86% (43) | 2% (1) | 78% (39) | 4% (2) |
|  |  |  | HHb ® ABP | 62% (31) | 10% (5) | 86% (43) | 4% (2) | 90% (45) | 2% (1) | 84% (42) | 4% (2) |
|  | Occipital | Left | ABP ® HHb | 70% (35) | 12% (6) | 84% (42) | 8% (4) | 90% (45) | 0% (0) | 80% (40) | 4% (2) |
|  |  |  | HHb ® ABP | 76% (38) | 12% (6) | 84% (42) | 8% (4) | 88% (44) | 0% (0) | 82% (41) | 4% (2) |
|  |  | Right | ABP ® HHb | 72% (36) | 6% (3) | 86% (43) | 2% (1) | 78% (39) | 8% (4) | 78% (39) | 2% (1) |
|  |  |  | HHb ® ABP | 78% (39) | 6% (3) | 88% (44) | 2% (1) | 78% (39) | 8% (4) | 84% (42) | 2% (1) |
|  | Temporal | Left | ABP ® HHb | 84% (42) | 6% (3) | 90% (45) | 0% (0) | 82% (41) | 0% (0) | 86% (43) | 8% (4) |
|  |  |  | HHb ® ABP | 76% (38) | 6% (3) | 88% (44) | 0% (0) | 82% (41) | 0% (0) | 86% (43) | 8% (4) |
|  |  | Right | ABP ® HHb | 72% (36) | 8% (4) | 82% (41) | 4% (2) | 86% (43) | 0% (0) | 76% (38) | 10% (5) |
|  |  |  | HHb ® ABP | 72% (36) | 8% (4) | 84% (42) | 4% (2) | 86% (43) | 0% (0) | 78% (39) | 10% (5) |
| ABP & tHb | Frontal | Left | ABP ® tHb | 74% (37) | 6% (3) | 84% (42) | 6% (3) | 88% (44) | 0% (0) | 80% (40) | 6% (3) |
|  |  |  | tHb ® ABP | 72% (36) | 6% (3) | 84% (42) | 6% (3) | 90% (45) | 0% (0) | 78% (39) | 6% (3) |
|  |  | Right | ABP ® tHb | 66% (33) | 6% (3) | 92% (46) | 0% (0) | 86% (43) | 0% (0) | 86% (43) | 6% (3) |
|  |  |  | tHb ® ABP | 78% (39) | 6% (3) | 88% (44) | 0% (0) | 88% (44) | 0% (0) | 86% (43) | 6% (3) |
|  | Parietal | Left | ABP ® tHb | 78% (39) | 4% (2) | 88% (44) | 2% (1) | 90% (45) | 2% (1) | 86% (43) | 2% (1) |
|  |  |  | tHb ® ABP | 78% (39) | 4% (2) | 86% (43) | 2% (1) | 90% (45) | 2% (1) | 84% (42) | 2% (1) |
|  |  | Right | ABP ® tHb | 72% (36) | 4% (2) | 84% (42) | 0% (0) | 88% (44) | 2% (1) | 76% (38) | 8% (4) |
|  |  |  | tHb ® ABP | 76% (38) | 4% (2) | 84% (42) | 0% (0) | 90% (45) | 2% (1) | 78% (39) | 8% (4) |
|  | Occipital | Left | ABP ® tHb | 68% (34) | 6% (3) | 88% (44) | 4% (2) | 82% (41) | 0% (0) | 76% (38) | 2% (1) |
|  |  |  | tHb ® ABP | 70% (35) | 6% (3) | 86% (43) | 4% (2) | 84% (42) | 0% (0) | 80% (40) | 2% (1) |
|  |  | Right | ABP ® tHb | 72% (36) | 8% (4) | 88% (44) | 2% (1) | 86% (43) | 0% (0) | 90% (45) | 2% (1) |
|  |  |  | tHb ® ABP | 70% (35) | 8% (4) | 92% (46) | 2% (1) | 90% (45) | 0% (0) | 86% (43) | 2% (1) |
|  | Temporal | Left | ABP ® tHb | 74% (37) | 2% (1) | 82% (41) | 4% (2) | 90% (45) | 2% (1) | 88% (44) | 0% (0) |
|  |  |  | tHb ® ABP | 78% (39) | 2% (1) | 80% (40) | 4% (2) | 90% (45) | 2% (1) | 88% (44) | 0% (0) |
|  |  | Right | ABP ® tHb | 60% (30) | 10% (5) | 80% (40) | 6% (3) | 92% (46) | 0% (0) | 80% (40) | 2% (1) |
|  |  |  | tHb ® ABP | 64% (32) | 10% (5) | 74% (37) | 6% (3) | 94% (47) | 0% (0) | 80% (40) | 2% (1) |
| ABP & HbDiff | Frontal | Left | ABP ® HbDiff | 76% (38) | 6% (3) | 92% (46) | 2% (1) | 90% (45) | 2% (1) | 88% (44) | 6% (3) |
|  |  |  | HbDiff ® ABP | 78% (39) | 6% (3) | 92% (46) | 2% (1) | 90% (45) | 2% (1) | 84% (42) | 6% (3) |
|  |  | Right | ABP ® HbDiff | 76% (38) | 4% (2) | 92% (46) | 2% (1) | 90% (45) | 0% (0) | 86% (43) | 4% (2) |
|  |  |  | HbDiff ® ABP | 78% (39) | 4% (2) | 94% (47) | 2% (1) | 92% (46) | 0% (0) | 86% (43) | 4% (2) |
|  | Parietal | Left | ABP ® HbDiff | 68% (34) | 2% (1) | 90% (45) | 6% (3) | 82% (41) | 0% (0) | 88% (44) | 2% (1) |
|  |  |  | HbDiff ® ABP | 64% (32) | 2% (1) | 88% (44) | 6% (3) | 86% (43) | 0% (0) | 90% (45) | 2% (1) |
|  |  | Right | ABP ® HbDiff | 70% (35) | 6% (3) | 84% (42) | 10% (5) | 88% (44) | 0% (0) | 76% (38) | 4% (2) |
|  |  |  | HbDiff ® ABP | 76% (38) | 6% (3) | 82% (41) | 10% (5) | 88% (44) | 0% (0) | 82% (41) | 4% (2) |
|  | Occipital | Left | ABP ® HbDiff | 68% (34) | 8% (4) | 90% (45) | 2% (1) | 86% (43) | 4% (2) | 74% (37) | 6% (3) |
|  |  |  | HbDiff ® ABP | 72% (36) | 8% (4) | 90% (45) | 2% (1) | 86% (43) | 4% (2) | 68% (34) | 6% (3) |
|  |  | Right | ABP ® HbDiff | 62% (31) | 4% (2) | 84% (42) | 6% (3) | 84% (42) | 2% (1) | 78% (39) | 8% (4) |
|  |  |  | HbDiff ® ABP | 64% (32) | 4% (2) | 84% (42) | 6% (3) | 88% (44) | 2% (1) | 78% (39) | 8% (4) |
|  | Temporal | Left | ABP ® HbDiff | 66% (33) | 8% (4) | 90% (45) | 0% (0) | 94% (47) | 0% (0) | 84% (42) | 4% (2) |
|  |  |  | HbDiff ® ABP | 68% (34) | 8% (4) | 90% (45) | 0% (0) | 92% (46) | 0% (0) | 78% (39) | 4% (2) |
|  |  | Right | ABP ® HbDiff | 76% (38) | 8% (4) | 84% (42) | 4% (2) | 90% (45) | 2% (1) | 74% (37) | 6% (3) |
|  |  |  | HbDiff ® ABP | 78% (39) | 8% (4) | 84% (42) | 4% (2) | 94% (47) | 2% (1) | 74% (37) | 6% (3) |
| The table shows the hemispheric responsiveness of signals using perturbation subgrouped Impulse Response Coefficients of Optimal VARIMA model using 1 Hz and 250 Hz data. *ABP, arterial blood pressure; HbDiff, hemoglobin difference; HbO, oxyhemoglobin; HHb, deoxyhemoglobin; rSO_2_, regional cerebral oxygen saturation; tHb, total hemoglobin; VARIMA, vector autoregressive integrative moving average.* | | | | | | | | | | | |

Appendix S7j: Perturbation Subgrouped Granger Causal Directionality Results Based on Greater F-Statistic

| **Signal Combination** | **Brain Lobe** | **Hemisphere** | **Direction** | **Directional Granger Causality [% (count)] for Perturbation Subgroups** | | | |
| --- | --- | --- | --- | --- | --- | --- | --- |
|  |  |  |  | **Baseline** | **Neurovascular Coupling** | **Orthostatic Challenge** | **Vascular Chemo-Reactivity** |
| **1 Hz Sampled Data** | | | | | | | |
| ABP & rSO_2_ | Frontal | Left | ABP ® rSO_2_ | 46% (23) | 54% (27) | 64% (32) | 60% (30) |
|  |  |  | rSO_2_ ® ABP | 42% (21) | 46% (23) | 36% (18) | 38% (19) |
|  |  |  | NA | 12% (6) | 0% (0) | 0% (0) | 2% (1) |
|  |  | Right | ABP ® rSO_2_ | 54% (27) | 54% (27) | 44% (22) | 50% (25) |
|  |  |  | rSO_2_ ® ABP | 40% (20) | 44% (22) | 56% (28) | 48% (24) |
|  |  |  | NA | 6% (3) | 2% (1) | 0% (0) | 2% (1) |
|  | Parietal | Left | ABP ® rSO_2_ | 52% (26) | 54% (27) | 52% (26) | 66% (33) |
|  |  |  | rSO_2_ ® ABP | 44% (22) | 46% (23) | 48% (24) | 34% (17) |
|  |  |  | NA | 4% (2) | 0% (0) | 0% (0) | 0% (0) |
|  |  | Right | ABP ® rSO_2_ | 42% (21) | 56% (28) | 54% (27) | 50% (25) |
|  |  |  | rSO_2_ ® ABP | 50% (25) | 38% (19) | 46% (23) | 44% (22) |
|  |  |  | NA | 8% (4) | 6% (3) | 0% (0) | 6% (3) |
|  | Occipital | Left | ABP ® rSO_2_ | 46% (23) | 40% (20) | 50% (25) | 56% (28) |
|  |  |  | rSO_2_ ® ABP | 48% (24) | 60% (30) | 50% (25) | 42% (21) |
|  |  |  | NA | 6% (3) | 0% (0) | 0% (0) | 2% (1) |
|  |  | Right | ABP ® rSO_2_ | 46% (23) | 66% (33) | 52% (26) | 44% (22) |
|  |  |  | rSO_2_ ® ABP | 50% (25) | 34% (17) | 48% (24) | 50% (25) |
|  |  |  | NA | 4% (2) | 0% (0) | 0% (0) | 6% (3) |
|  | Temporal | Left | ABP ® rSO_2_ | 46% (23) | 50% (25) | 46% (23) | 44% (22) |
|  |  |  | rSO_2_ ® ABP | 42% (21) | 48% (24) | 54% (27) | 54% (27) |
|  |  |  | NA | 12% (6) | 2% (1) | 0% (0) | 2% (1) |
|  |  | Right | ABP ® rSO_2_ | 56% (28) | 62% (31) | 58% (29) | 46% (23) |
|  |  |  | rSO_2_ ® ABP | 38% (19) | 38% (19) | 42% (21) | 54% (27) |
|  |  |  | NA | 6% (3) | 0% (0) | 0% (0) | 0% (0) |
| ABP & HbO | Frontal | Left | ABP ® HbO | 50% (25) | 60% (30) | 60% (30) | 54% (27) |
|  |  |  | HbO ® ABP | 46% (23) | 36% (18) | 40% (20) | 40% (20) |
|  |  |  | NA | 4% (2) | 4% (2) | 0% (0) | 6% (3) |
|  |  | Right | ABP ® HbO | 54% (27) | 50% (25) | 64% (32) | 52% (26) |
|  |  |  | HbO ® ABP | 42% (21) | 48% (24) | 36% (18) | 46% (23) |
|  |  |  | NA | 4% (2) | 2% (1) | 0% (0) | 2% (1) |
|  | Parietal | Left | ABP ® HbO | 50% (25) | 48% (24) | 54% (27) | 64% (32) |
|  |  |  | HbO ® ABP | 48% (24) | 50% (25) | 46% (23) | 34% (17) |
|  |  |  | NA | 2% (1) | 2% (1) | 0% (0) | 2% (1) |
|  |  | Right | ABP ® HbO | 60% (30) | 50% (25) | 58% (29) | 64% (32) |
|  |  |  | HbO ® ABP | 36% (18) | 44% (22) | 42% (21) | 32% (16) |
|  |  |  | NA | 4% (2) | 6% (3) | 0% (0) | 4% (2) |
|  | Occipital | Left | ABP ® HbO | 48% (24) | 46% (23) | 42% (21) | 50% (25) |
|  |  |  | HbO ® ABP | 50% (25) | 48% (24) | 58% (29) | 48% (24) |
|  |  |  | NA | 2% (1) | 6% (3) | 0% (0) | 2% (1) |
|  |  | Right | ABP ® HbO | 42% (21) | 56% (28) | 58% (29) | 42% (21) |
|  |  |  | HbO ® ABP | 48% (24) | 44% (22) | 42% (21) | 54% (27) |
|  |  |  | NA | 10% (5) | 0% (0) | 0% (0) | 4% (2) |
|  | Temporal | Left | ABP ® HbO | 54% (27) | 56% (28) | 56% (28) | 40% (20) |
|  |  |  | HbO ® ABP | 42% (21) | 44% (22) | 44% (22) | 56% (28) |
|  |  |  | NA | 4% (2) | 0% (0) | 0% (0) | 4% (2) |
|  |  | Right | ABP ® HbO | 46% (23) | 56% (28) | 56% (28) | 52% (26) |
|  |  |  | HbO ® ABP | 46% (23) | 42% (21) | 44% (22) | 46% (23) |
|  |  |  | NA | 8% (4) | 2% (1) | 0% (0) | 2% (1) |
| ABP & HHb | Frontal | Left | ABP ® HHb | 56% (28) | 56% (28) | 48% (24) | 46% (23) |
|  |  |  | HHb ® ABP | 42% (21) | 42% (21) | 52% (26) | 50% (25) |
|  |  |  | NA | 2% (1) | 2% (1) | 0% (0) | 4% (2) |
|  |  | Right | ABP ® HHb | 58% (29) | 50% (25) | 46% (23) | 50% (25) |
|  |  |  | HHb ® ABP | 34% (17) | 50% (25) | 54% (27) | 44% (22) |
|  |  |  | NA | 8% (4) | 0% (0) | 0% (0) | 6% (3) |
|  | Parietal | Left | ABP ® HHb | 38% (19) | 52% (26) | 50% (25) | 64% (32) |
|  |  |  | HHb ® ABP | 54% (27) | 44% (22) | 50% (25) | 36% (18) |
|  |  |  | NA | 8% (4) | 4% (2) | 0% (0) | 0% (0) |
|  |  | Right | ABP ® HHb | 50% (25) | 46% (23) | 58% (29) | 46% (23) |
|  |  |  | HHb ® ABP | 44% (22) | 50% (25) | 42% (21) | 50% (25) |
|  |  |  | NA | 6% (3) | 4% (2) | 0% (0) | 4% (2) |
|  | Occipital | Left | ABP ® HHb | 52% (26) | 46% (23) | 58% (29) | 42% (21) |
|  |  |  | HHb ® ABP | 42% (21) | 52% (26) | 42% (21) | 52% (26) |
|  |  |  | NA | 6% (3) | 2% (1) | 0% (0) | 6% (3) |
|  |  | Right | ABP ® HHb | 42% (21) | 58% (29) | 54% (27) | 42% (21) |
|  |  |  | HHb ® ABP | 50% (25) | 42% (21) | 46% (23) | 56% (28) |
|  |  |  | NA | 8% (4) | 0% (0) | 0% (0) | 2% (1) |
|  | Temporal | Left | ABP ® HHb | 48% (24) | 64% (32) | 44% (22) | 44% (22) |
|  |  |  | HHb ® ABP | 46% (23) | 36% (18) | 56% (28) | 48% (24) |
|  |  |  | NA | 6% (3) | 0% (0) | 0% (0) | 8% (4) |
|  |  | Right | ABP ® HHb | 42% (21) | 52% (26) | 62% (31) | 46% (23) |
|  |  |  | HHb ® ABP | 48% (24) | 46% (23) | 36% (18) | 52% (26) |
|  |  |  | NA | 10% (5) | 2% (1) | 2% (1) | 2% (1) |
| ABP & tHb | Frontal | Left | ABP ® tHb | 54% (27) | 48% (24) | 64% (32) | 46% (23) |
|  |  |  | tHb ® ABP | 44% (22) | 46% (23) | 36% (18) | 52% (26) |
|  |  |  | NA | 2% (1) | 6% (3) | 0% (0) | 2% (1) |
|  |  | Right | ABP ® tHb | 50% (25) | 52% (26) | 54% (27) | 60% (30) |
|  |  |  | tHb ® ABP | 46% (23) | 48% (24) | 46% (23) | 38% (19) |
|  |  |  | NA | 4% (2) | 0% (0) | 0% (0) | 2% (1) |
|  | Parietal | Left | ABP ® tHb | 52% (26) | 50% (25) | 44% (22) | 44% (22) |
|  |  |  | tHb ® ABP | 44% (22) | 50% (25) | 56% (28) | 56% (28) |
|  |  |  | NA | 4% (2) | 0% (0) | 0% (0) | 0% (0) |
|  |  | Right | ABP ® tHb | 48% (24) | 50% (25) | 56% (28) | 48% (24) |
|  |  |  | tHb ® ABP | 46% (23) | 48% (24) | 44% (22) | 44% (22) |
|  |  |  | NA | 6% (3) | 2% (1) | 0% (0) | 8% (4) |
|  | Occipital | Left | ABP ® tHb | 48% (24) | 42% (21) | 48% (24) | 44% (22) |
|  |  |  | tHb ® ABP | 40% (20) | 58% (29) | 52% (26) | 52% (26) |
|  |  |  | NA | 12% (6) | 0% (0) | 0% (0) | 4% (2) |
|  |  | Right | ABP ® tHb | 50% (25) | 60% (30) | 48% (24) | 40% (20) |
|  |  |  | tHb ® ABP | 44% (22) | 40% (20) | 52% (26) | 54% (27) |
|  |  |  | NA | 6% (3) | 0% (0) | 0% (0) | 6% (3) |
|  | Temporal | Left | ABP ® tHb | 62% (31) | 50% (25) | 50% (25) | 56% (28) |
|  |  |  | tHb ® ABP | 36% (18) | 50% (25) | 50% (25) | 44% (22) |
|  |  |  | NA | 2% (1) | 0% (0) | 0% (0) | 0% (0) |
|  |  | Right | ABP ® tHb | 42% (21) | 48% (24) | 58% (29) | 42% (21) |
|  |  |  | tHb ® ABP | 54% (27) | 50% (25) | 42% (21) | 58% (29) |
|  |  |  | NA | 4% (2) | 2% (1) | 0% (0) | 0% (0) |
| ABP & HbDiff | Frontal | Left | ABP ® HbDiff | 36% (18) | 52% (26) | 60% (30) | 58% (29) |
|  |  |  | HbDiff ® ABP | 52% (26) | 46% (23) | 40% (20) | 36% (18) |
|  |  |  | NA | 12% (6) | 2% (1) | 0% (0) | 6% (3) |
|  |  | Right | ABP ® HbDiff | 48% (24) | 60% (30) | 46% (23) | 48% (24) |
|  |  |  | HbDiff ® ABP | 42% (21) | 38% (19) | 54% (27) | 50% (25) |
|  |  |  | NA | 10% (5) | 2% (1) | 0% (0) | 2% (1) |
|  | Parietal | Left | ABP ® HbDiff | 60% (30) | 48% (24) | 48% (24) | 58% (29) |
|  |  |  | HbDiff ® ABP | 36% (18) | 48% (24) | 52% (26) | 40% (20) |
|  |  |  | NA | 4% (2) | 4% (2) | 0% (0) | 2% (1) |
|  |  | Right | ABP ® HbDiff | 40% (20) | 58% (29) | 58% (29) | 58% (29) |
|  |  |  | HbDiff ® ABP | 48% (24) | 34% (17) | 42% (21) | 38% (19) |
|  |  |  | NA | 12% (6) | 8% (4) | 0% (0) | 4% (2) |
|  | Occipital | Left | ABP ® HbDiff | 48% (24) | 38% (19) | 50% (25) | 56% (28) |
|  |  |  | HbDiff ® ABP | 46% (23) | 60% (30) | 50% (25) | 40% (20) |
|  |  |  | NA | 6% (3) | 2% (1) | 0% (0) | 4% (2) |
|  |  | Right | ABP ® HbDiff | 52% (26) | 62% (31) | 48% (24) | 44% (22) |
|  |  |  | HbDiff ® ABP | 46% (23) | 34% (17) | 52% (26) | 48% (24) |
|  |  |  | NA | 2% (1) | 4% (2) | 0% (0) | 8% (4) |
|  | Temporal | Left | ABP ® HbDiff | 46% (23) | 52% (26) | 40% (20) | 48% (24) |
|  |  |  | HbDiff ® ABP | 46% (23) | 48% (24) | 60% (30) | 48% (24) |
|  |  |  | NA | 8% (4) | 0% (0) | 0% (0) | 4% (2) |
|  |  | Right | ABP ® HbDiff | 54% (27) | 68% (34) | 48% (24) | 44% (22) |
|  |  |  | HbDiff ® ABP | 42% (21) | 30% (15) | 52% (26) | 54% (27) |
|  |  |  | NA | 4% (2) | 2% (1) | 0% (0) | 2% (1) |
| **250 Hz Sampled Data** | | | | | | | |
| ABP & rSO_2_ | Frontal | Left | ABP ® rSO_2_ | 64% (32) | 54% (27) | 64% (32) | 54% (27) |
|  |  |  | rSO_2_ ® ABP | 36% (18) | 44% (22) | 36% (18) | 42% (21) |
|  |  |  | NA | 0% (0) | 2% (1) | 0% (0) | 4% (2) |
|  |  | Right | ABP ® rSO_2_ | 48% (24) | 42% (21) | 48% (24) | 38% (19) |
|  |  |  | rSO_2_ ® ABP | 50% (25) | 58% (29) | 52% (26) | 58% (29) |
|  |  |  | NA | 2% (1) | 0% (0) | 0% (0) | 4% (2) |
|  | Parietal | Left | ABP ® rSO_2_ | 48% (24) | 42% (21) | 52% (26) | 40% (20) |
|  |  |  | rSO_2_ ® ABP | 48% (24) | 58% (29) | 48% (24) | 58% (29) |
|  |  |  | NA | 4% (2) | 0% (0) | 0% (0) | 2% (1) |
|  |  | Right | ABP ® rSO_2_ | 42% (21) | 50% (25) | 48% (24) | 44% (22) |
|  |  |  | rSO_2_ ® ABP | 50% (25) | 44% (22) | 52% (26) | 54% (27) |
|  |  |  | NA | 8% (4) | 6% (3) | 0% (0) | 2% (1) |
|  | Occipital | Left | ABP ® rSO_2_ | 54% (27) | 54% (27) | 56% (28) | 48% (24) |
|  |  |  | rSO_2_ ® ABP | 42% (21) | 44% (22) | 44% (22) | 50% (25) |
|  |  |  | NA | 4% (2) | 2% (1) | 0% (0) | 2% (1) |
|  |  | Right | ABP ® rSO_2_ | 56% (28) | 60% (30) | 66% (33) | 38% (19) |
|  |  |  | rSO_2_ ® ABP | 40% (20) | 40% (20) | 34% (17) | 58% (29) |
|  |  |  | NA | 4% (2) | 0% (0) | 0% (0) | 4% (2) |
|  | Temporal | Left | ABP ® rSO_2_ | 44% (22) | 54% (27) | 40% (20) | 48% (24) |
|  |  |  | rSO_2_ ® ABP | 48% (24) | 46% (23) | 60% (30) | 44% (22) |
|  |  |  | NA | 8% (4) | 0% (0) | 0% (0) | 8% (4) |
|  |  | Right | ABP ® rSO_2_ | 56% (28) | 52% (26) | 52% (26) | 40% (20) |
|  |  |  | rSO_2_ ® ABP | 42% (21) | 48% (24) | 48% (24) | 52% (26) |
|  |  |  | NA | 2% (1) | 0% (0) | 0% (0) | 8% (4) |
| ABP & HbO | Frontal | Left | ABP ® HbO | 70% (35) | 52% (26) | 56% (28) | 44% (22) |
|  |  |  | HbO ® ABP | 28% (14) | 42% (21) | 44% (22) | 50% (25) |
|  |  |  | NA | 2% (1) | 6% (3) | 0% (0) | 6% (3) |
|  |  | Right | ABP ® HbO | 50% (25) | 58% (29) | 62% (31) | 52% (26) |
|  |  |  | HbO ® ABP | 44% (22) | 42% (21) | 38% (19) | 42% (21) |
|  |  |  | NA | 6% (3) | 0% (0) | 0% (0) | 6% (3) |
|  | Parietal | Left | ABP ® HbO | 54% (27) | 44% (22) | 60% (30) | 42% (21) |
|  |  |  | HbO ® ABP | 44% (22) | 52% (26) | 40% (20) | 54% (27) |
|  |  |  | NA | 2% (1) | 4% (2) | 0% (0) | 4% (2) |
|  |  | Right | ABP ® HbO | 52% (26) | 36% (18) | 54% (27) | 54% (27) |
|  |  |  | HbO ® ABP | 44% (22) | 58% (29) | 46% (23) | 44% (22) |
|  |  |  | NA | 4% (2) | 6% (3) | 0% (0) | 2% (1) |
|  | Occipital | Left | ABP ® HbO | 48% (24) | 42% (21) | 58% (29) | 52% (26) |
|  |  |  | HbO ® ABP | 48% (24) | 56% (28) | 42% (21) | 46% (23) |
|  |  |  | NA | 4% (2) | 2% (1) | 0% (0) | 2% (1) |
|  |  | Right | ABP ® HbO | 50% (25) | 52% (26) | 60% (30) | 42% (21) |
|  |  |  | HbO ® ABP | 42% (21) | 48% (24) | 40% (20) | 48% (24) |
|  |  |  | NA | 8% (4) | 0% (0) | 0% (0) | 10% (5) |
|  | Temporal | Left | ABP ® HbO | 40% (20) | 52% (26) | 48% (24) | 54% (27) |
|  |  |  | HbO ® ABP | 50% (25) | 46% (23) | 52% (26) | 46% (23) |
|  |  |  | NA | 10% (5) | 2% (1) | 0% (0) | 0% (0) |
|  |  | Right | ABP ® HbO | 54% (27) | 46% (23) | 46% (23) | 48% (24) |
|  |  |  | HbO ® ABP | 40% (20) | 46% (23) | 54% (27) | 50% (25) |
|  |  |  | NA | 6% (3) | 8% (4) | 0% (0) | 2% (1) |
| ABP & HHb | Frontal | Left | ABP ® HHb | 48% (24) | 52% (26) | 52% (26) | 50% (25) |
|  |  |  | HHb ® ABP | 46% (23) | 44% (22) | 48% (24) | 44% (22) |
|  |  |  | NA | 6% (3) | 4% (2) | 0% (0) | 6% (3) |
|  |  | Right | ABP ® HHb | 48% (24) | 46% (23) | 58% (29) | 56% (28) |
|  |  |  | HHb ® ABP | 46% (23) | 54% (27) | 42% (21) | 38% (19) |
|  |  |  | NA | 6% (3) | 0% (0) | 0% (0) | 6% (3) |
|  | Parietal | Left | ABP ® HHb | 52% (26) | 58% (29) | 46% (23) | 64% (32) |
|  |  |  | HHb ® ABP | 40% (20) | 40% (20) | 54% (27) | 32% (16) |
|  |  |  | NA | 8% (4) | 2% (1) | 0% (0) | 4% (2) |
|  |  | Right | ABP ® HHb | 54% (27) | 50% (25) | 50% (25) | 46% (23) |
|  |  |  | HHb ® ABP | 36% (18) | 48% (24) | 50% (25) | 50% (25) |
|  |  |  | NA | 10% (5) | 2% (1) | 0% (0) | 4% (2) |
|  | Occipital | Left | ABP ® HHb | 44% (22) | 32% (16) | 58% (29) | 44% (22) |
|  |  |  | HHb ® ABP | 44% (22) | 64% (32) | 42% (21) | 52% (26) |
|  |  |  | NA | 12% (6) | 4% (2) | 0% (0) | 4% (2) |
|  |  | Right | ABP ® HHb | 44% (22) | 52% (26) | 54% (27) | 48% (24) |
|  |  |  | HHb ® ABP | 50% (25) | 48% (24) | 46% (23) | 50% (25) |
|  |  |  | NA | 6% (3) | 0% (0) | 0% (0) | 2% (1) |
|  | Temporal | Left | ABP ® HHb | 52% (26) | 50% (25) | 44% (22) | 48% (24) |
|  |  |  | HHb ® ABP | 46% (23) | 50% (25) | 56% (28) | 44% (22) |
|  |  |  | NA | 2% (1) | 0% (0) | 0% (0) | 8% (4) |
|  |  | Right | ABP ® HHb | 56% (28) | 46% (23) | 62% (31) | 46% (23) |
|  |  |  | HHb ® ABP | 36% (18) | 50% (25) | 38% (19) | 46% (23) |
|  |  |  | NA | 8% (4) | 4% (2) | 0% (0) | 8% (4) |
| ABP & tHb | Frontal | Left | ABP ® tHb | 60% (30) | 38% (19) | 54% (27) | 50% (25) |
|  |  |  | tHb ® ABP | 36% (18) | 56% (28) | 46% (23) | 44% (22) |
|  |  |  | NA | 4% (2) | 6% (3) | 0% (0) | 6% (3) |
|  |  | Right | ABP ® tHb | 54% (27) | 44% (22) | 52% (26) | 50% (25) |
|  |  |  | tHb ® ABP | 42% (21) | 56% (28) | 48% (24) | 44% (22) |
|  |  |  | NA | 4% (2) | 0% (0) | 0% (0) | 6% (3) |
|  | Parietal | Left | ABP ® tHb | 64% (32) | 44% (22) | 44% (22) | 42% (21) |
|  |  |  | tHb ® ABP | 34% (17) | 54% (27) | 56% (28) | 56% (28) |
|  |  |  | NA | 2% (1) | 2% (1) | 0% (0) | 2% (1) |
|  |  | Right | ABP ® tHb | 60% (30) | 46% (23) | 54% (27) | 48% (24) |
|  |  |  | tHb ® ABP | 36% (18) | 54% (27) | 44% (22) | 44% (22) |
|  |  |  | NA | 4% (2) | 0% (0) | 2% (1) | 8% (4) |
|  | Occipital | Left | ABP ® tHb | 54% (27) | 46% (23) | 54% (27) | 40% (20) |
|  |  |  | tHb ® ABP | 40% (20) | 54% (27) | 46% (23) | 58% (29) |
|  |  |  | NA | 6% (3) | 0% (0) | 0% (0) | 2% (1) |
|  |  | Right | ABP ® tHb | 48% (24) | 62% (31) | 64% (32) | 36% (18) |
|  |  |  | tHb ® ABP | 46% (23) | 36% (18) | 36% (18) | 62% (31) |
|  |  |  | NA | 6% (3) | 2% (1) | 0% (0) | 2% (1) |
|  | Temporal | Left | ABP ® tHb | 46% (23) | 46% (23) | 56% (28) | 64% (32) |
|  |  |  | tHb ® ABP | 52% (26) | 50% (25) | 42% (21) | 36% (18) |
|  |  |  | NA | 2% (1) | 4% (2) | 2% (1) | 0% (0) |
|  |  | Right | ABP ® tHb | 52% (26) | 44% (22) | 48% (24) | 44% (22) |
|  |  |  | tHb ® ABP | 38% (19) | 52% (26) | 52% (26) | 54% (27) |
|  |  |  | NA | 10% (5) | 4% (2) | 0% (0) | 2% (1) |
| ABP & HbDiff | Frontal | Left | ABP ® HbDiff | 54% (27) | 46% (23) | 54% (27) | 54% (27) |
|  |  |  | HbDiff ® ABP | 42% (21) | 52% (26) | 46% (23) | 40% (20) |
|  |  |  | NA | 4% (2) | 2% (1) | 0% (0) | 6% (3) |
|  |  | Right | ABP ® HbDiff | 44% (22) | 62% (31) | 50% (25) | 48% (24) |
|  |  |  | HbDiff ® ABP | 54% (27) | 38% (19) | 50% (25) | 48% (24) |
|  |  |  | NA | 2% (1) | 0% (0) | 0% (0) | 4% (2) |
|  | Parietal | Left | ABP ® HbDiff | 42% (21) | 52% (26) | 50% (25) | 46% (23) |
|  |  |  | HbDiff ® ABP | 56% (28) | 46% (23) | 50% (25) | 52% (26) |
|  |  |  | NA | 2% (1) | 2% (1) | 0% (0) | 2% (1) |
|  |  | Right | ABP ® HbDiff | 42% (21) | 46% (23) | 48% (24) | 52% (26) |
|  |  |  | HbDiff ® ABP | 52% (26) | 46% (23) | 52% (26) | 46% (23) |
|  |  |  | NA | 6% (3) | 8% (4) | 0% (0) | 2% (1) |
|  | Occipital | Left | ABP ® HbDiff | 50% (25) | 44% (22) | 52% (26) | 48% (24) |
|  |  |  | HbDiff ® ABP | 44% (22) | 54% (27) | 48% (24) | 46% (23) |
|  |  |  | NA | 6% (3) | 2% (1) | 0% (0) | 6% (3) |
|  |  | Right | ABP ® HbDiff | 60% (30) | 56% (28) | 50% (25) | 40% (20) |
|  |  |  | HbDiff ® ABP | 36% (18) | 38% (19) | 50% (25) | 52% (26) |
|  |  |  | NA | 4% (2) | 6% (3) | 0% (0) | 8% (4) |
|  | Temporal | Left | ABP ® HbDiff | 50% (25) | 46% (23) | 50% (25) | 46% (23) |
|  |  |  | HbDiff ® ABP | 42% (21) | 54% (27) | 50% (25) | 50% (25) |
|  |  |  | NA | 8% (4) | 0% (0) | 0% (0) | 4% (2) |
|  |  | Right | ABP ® HbDiff | 56% (28) | 44% (22) | 60% (30) | 48% (24) |
|  |  |  | HbDiff ® ABP | 36% (18) | 54% (27) | 40% (20) | 46% (23) |
|  |  |  | NA | 8% (4) | 2% (1) | 0% (0) | 6% (3) |
| The table shows the subgrouped Granger causal directionality results between ABP and NIRS signals using 1 Hz and 250 Hz data. *ABP, arterial blood pressure; HbDiff, hemoglobin difference; HbO, oxyhemoglobin; HHb, deoxyhemoglobin; NIRS, near-infrared spectroscopy; rSO_2_, regional cerebral oxygen saturation; tHb, total hemoglobin.* | | | | | | | |
